# Supplementary material for: Accelerating the prediction and discovery of peptide hydrogels with human-in-the-loop
Source: Nat Commun. 2023 Jun 30;14:3880. doi: 10.1038/s41467-023-39648-2 (PMC10313671; doi:10.1038/s41467-023-39648-2)
Supplement: Supplementary file 1 — Supplementary Information [file 41467_2023_39648_MOESM1_ESM.pdf]

Supplementary Information

Accelerating Prediction and Discovery of Peptide Hydrogel with Human-in-the-Loop

Tengyan Xu[1,2#], Jiaqi Wang[3,4,5#], Shuang Zhao[5#], Dinghao Chen[1#], Hongyue Zhang[1],

Yu Fang[1], Nan Kong[1], Ziao Zhou[1], Wenbin Li[3,4,5]*, Huaimin Wang[1,2,3]*

Affiliations:

[1]Department of Chemistry, School of Science, Westlake University, 18 Shilongshan Road, Hangzhou 310024, Zhejiang Province, China.

[2]Institute of Natural Sciences, Westlake Institute for Advanced Study, 18 Shilongshan Road, Hangzhou 310024, Zhejiang Province, China.

[3]Research Center for the Industries of the Future, Westlake University, No. 600 Dunyu Road, Sandun Town, Xihu District, Hangzhou 310030, Zhejiang Province, China.

[4]Institute of Advanced Technology, Westlake Institute for Advanced Study, 18 Shilongshan Road, Hangzhou 310024, Zhejiang Province, China.

[5]School of Engineering, Westlake University, 18 Shilongshan Road, Hangzhou 310024, Zhejiang Province, China.

[#]These authors contributed equally to this work.
*Correspondence to: Email: liwenbin@westlake.edu.cn;
wanghuaimin@westlake.edu.cn

Supplementary Figures

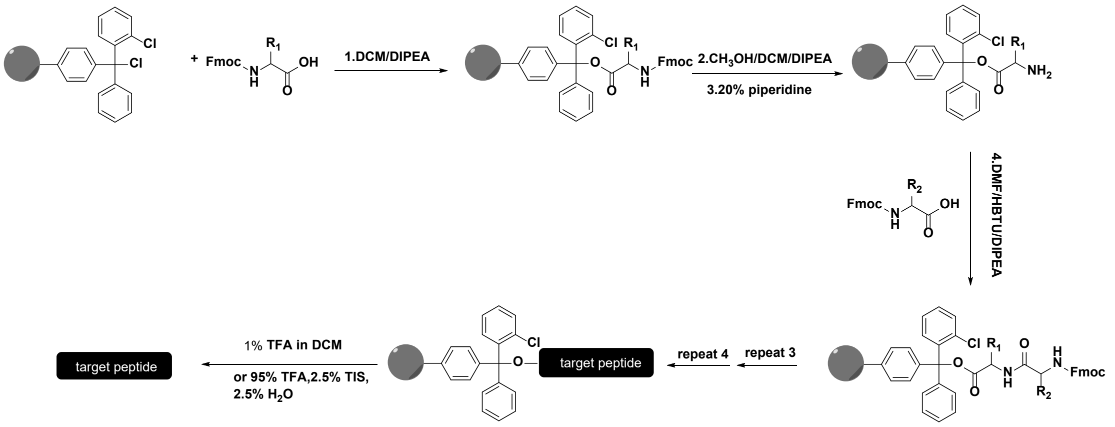

Supplementary Figure 1. The synthetic route of tetrapeptides.

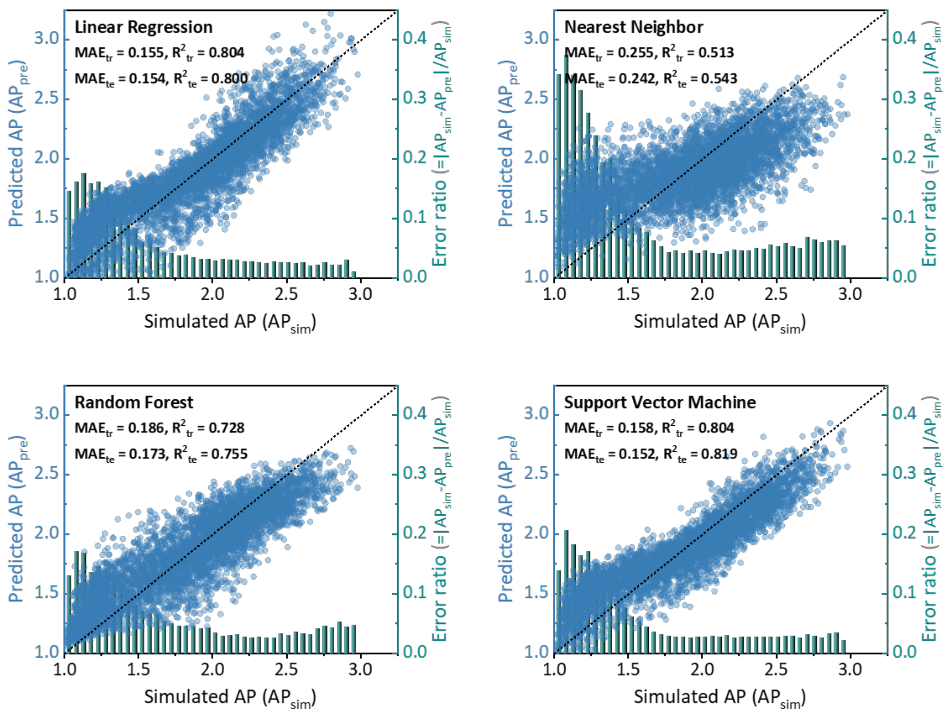

Supplementary Figure 2. Training (MAE_{tr}, R^2_{tr}) and testing (MAE_{te}, R^2_{te}) performance with **1,000** training datasets with 80-bit representation, tested by 5,000 data.

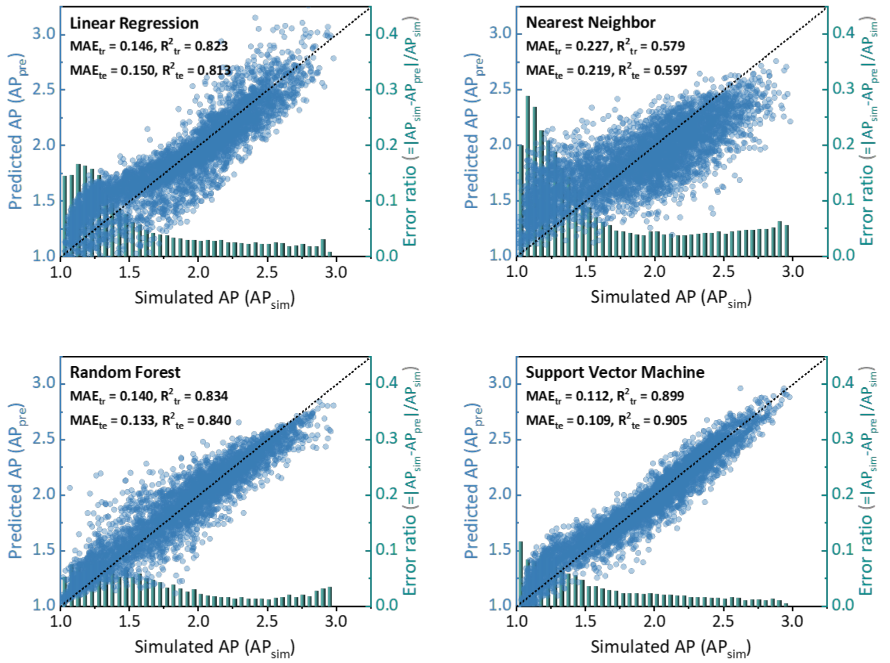

Supplementary Figure 3. Training (MAE_{tr}, R^2_{tr}) and testing (MAE_{te}, R^2_{te}) performance with **5,000** training datasets with 80-bit representation, tested by 5,000 data.

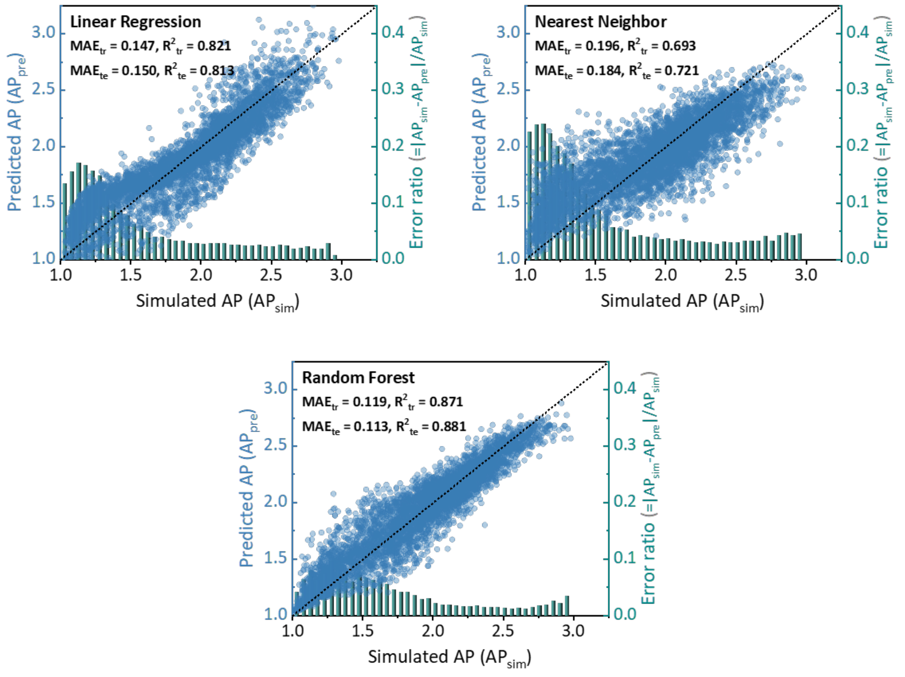

Supplementary Figure 4. Training (MAE_{tr}, R^2_{tr}) and testing (MAE_{te}, R^2_{te}) performance with **10,000** training datasets with 80-bit representation, tested by 5,000 data. It should be noted that, since the SVM model is the optimal one chosen for predicting the AP values of 160,000 tetrapeptides, the model performance is thus presented in main text as Fig. 2b, while not here for avoiding repeatability.

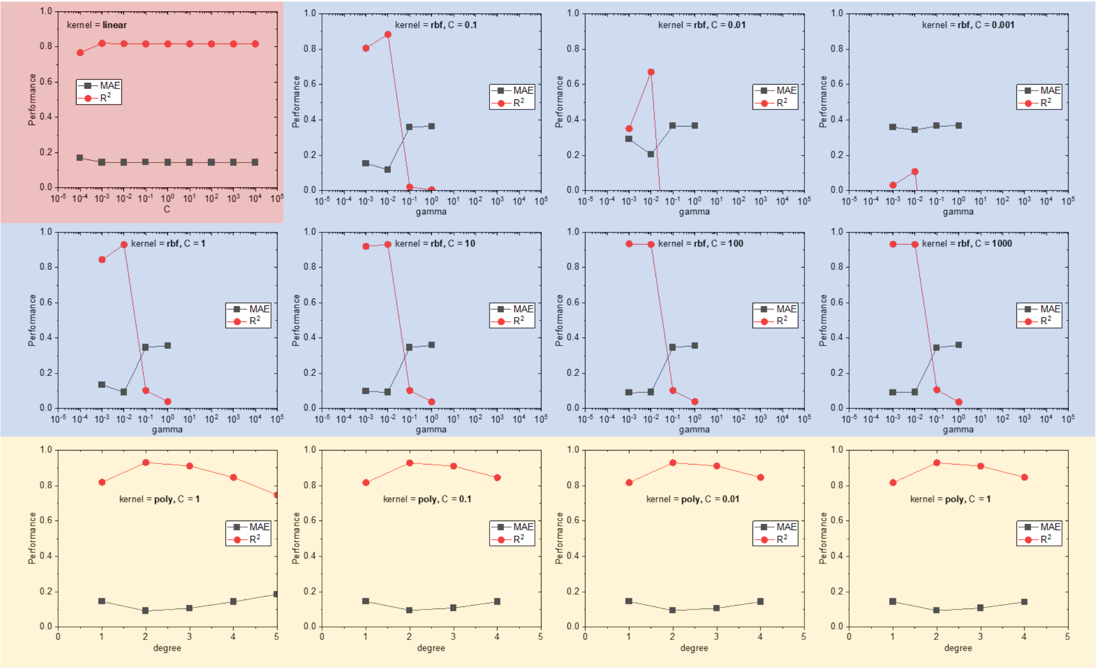

Supplementary Figure 5. Performance of SVM model with different kernels and hyperparameters, trained with 10,000 data represented by 80-bit one-hot representation.

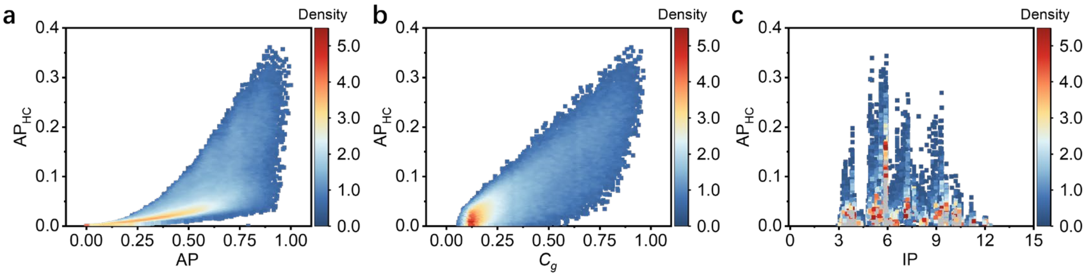

Supplementary Figure 6. Correlation between a) AP_{HC} and AP, b) AP_{HC} and C_g, c) AP_{HC} and isoelectric points (IP). The IP is calculated by the online tool: Isoelectric Point Calculator 2.0 (IPC 2.0 - Isoelectric point and pKa prediction for proteins and peptides using deep learning (mimuw.edu.pl)).

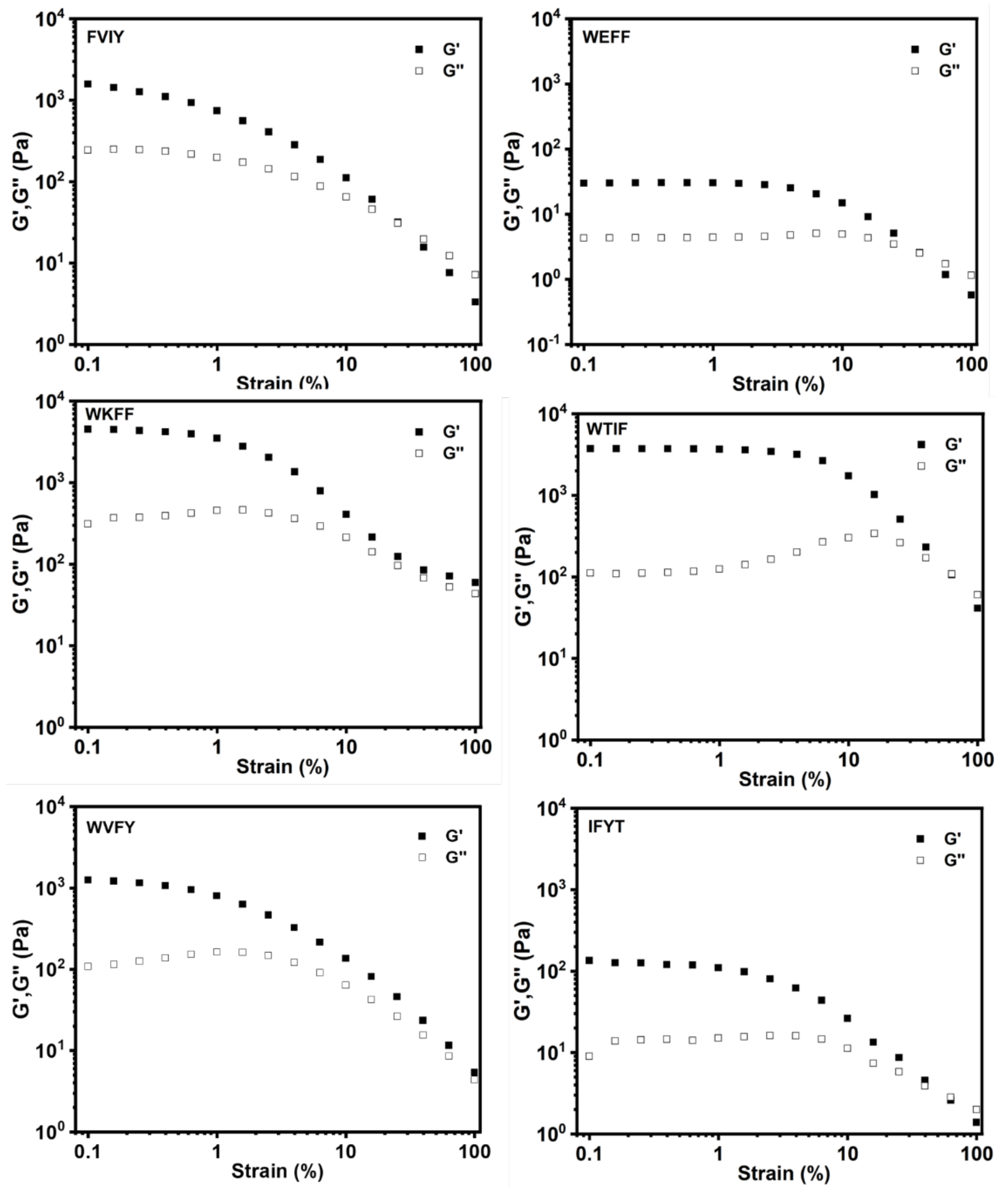

Supplementary Figure 7. Dynamic strain sweeps of 6 representative tetrapeptide hydrogels at frequency of 1 Hz.

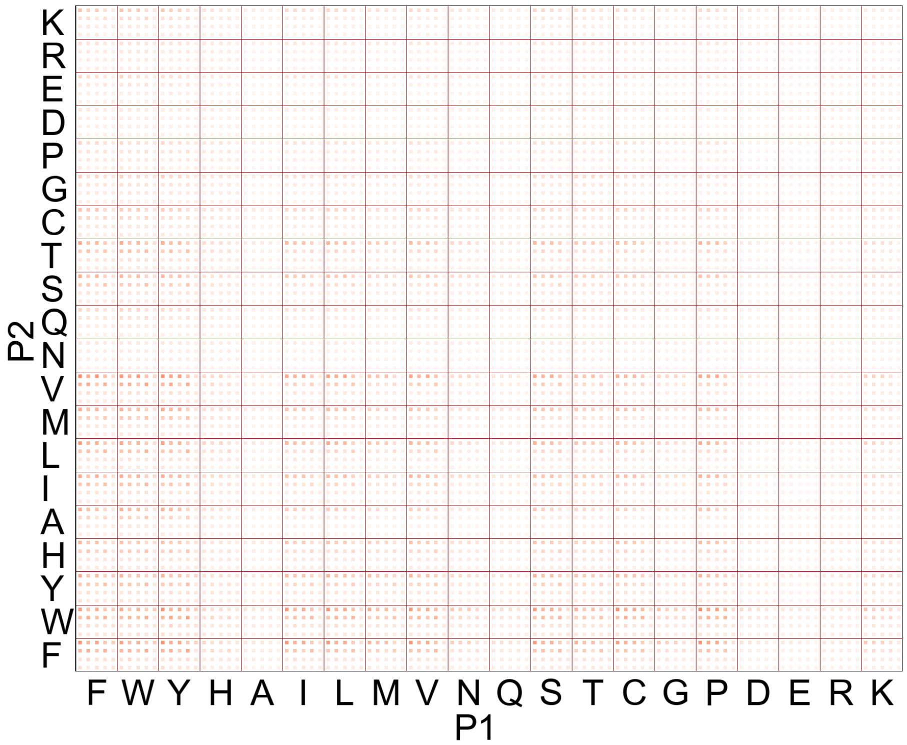

Supplementary Figure 8. Distribution of 8000 AP_{HC} with A fixed at C termini (P4). The x-axis is P1 (N-terminus), the y-axis is P2, and the third position is illustrated in the rectangular box.

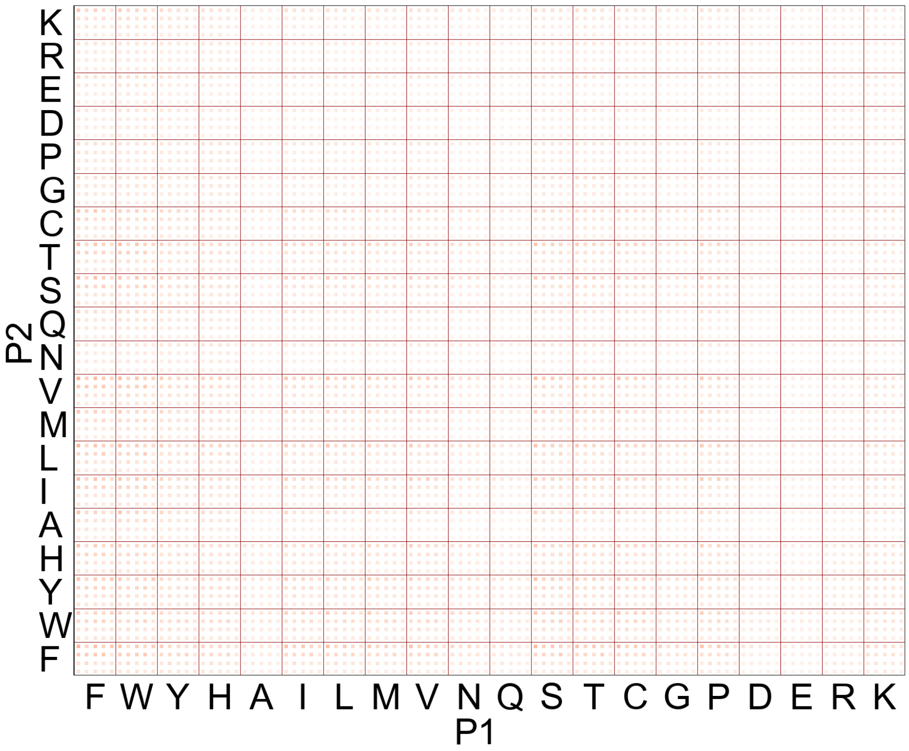

Supplementary Figure 9. Distribution of 8000 AP_{HC} with C fixed at C termini (P4).

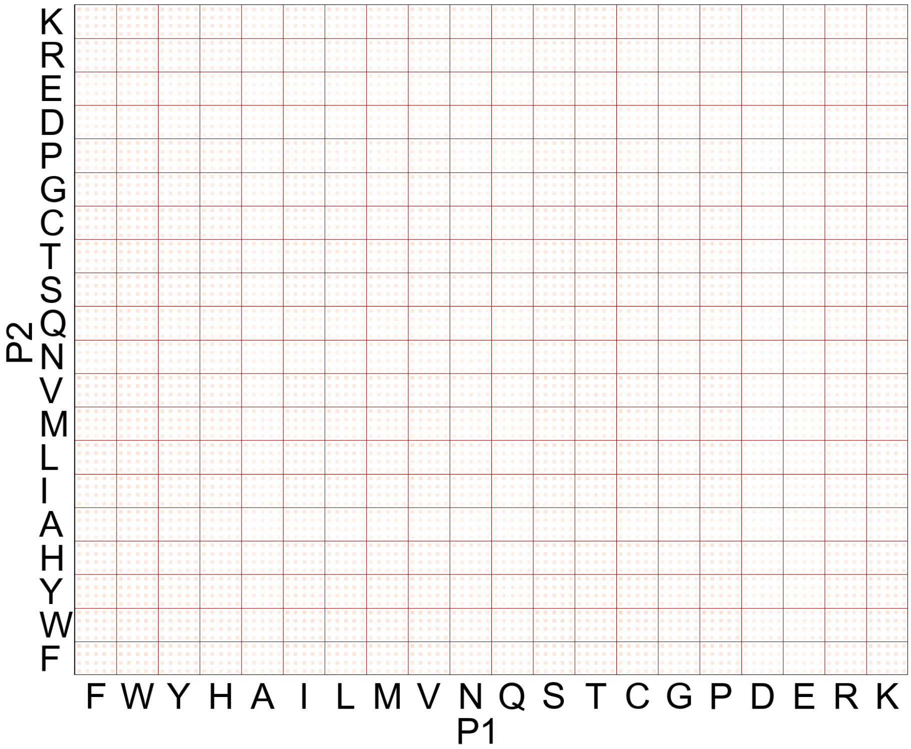

Supplementary Figure 10. Distribution of 8000 AP_{HC} with D fixed at C termini (P4).

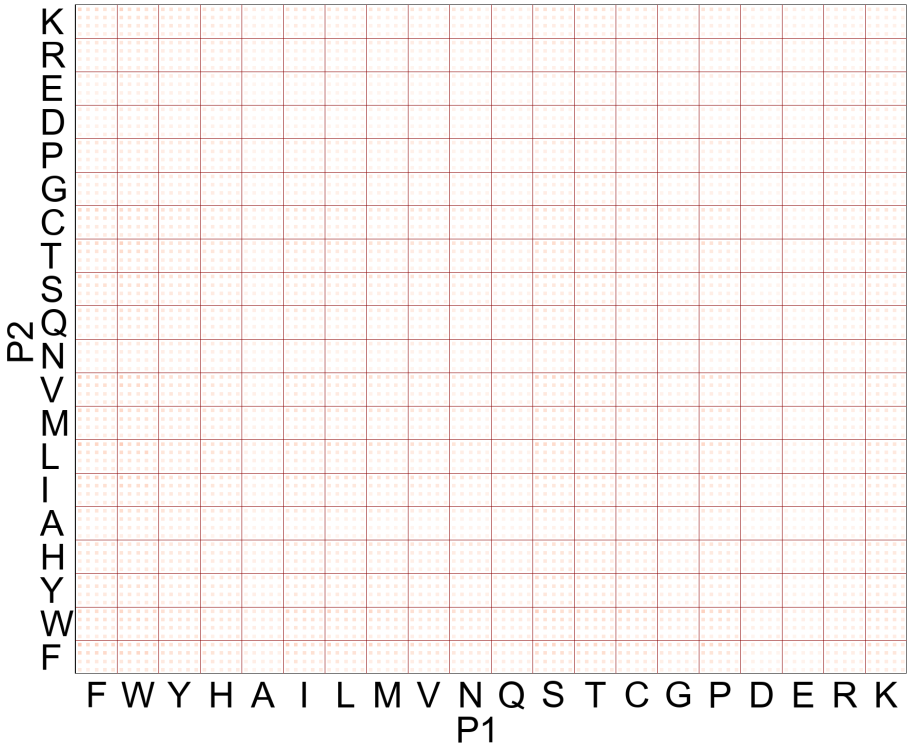

Supplementary Figure 11. Distribution of 8000 AP_{HC} with E fixed at C termini (P4).

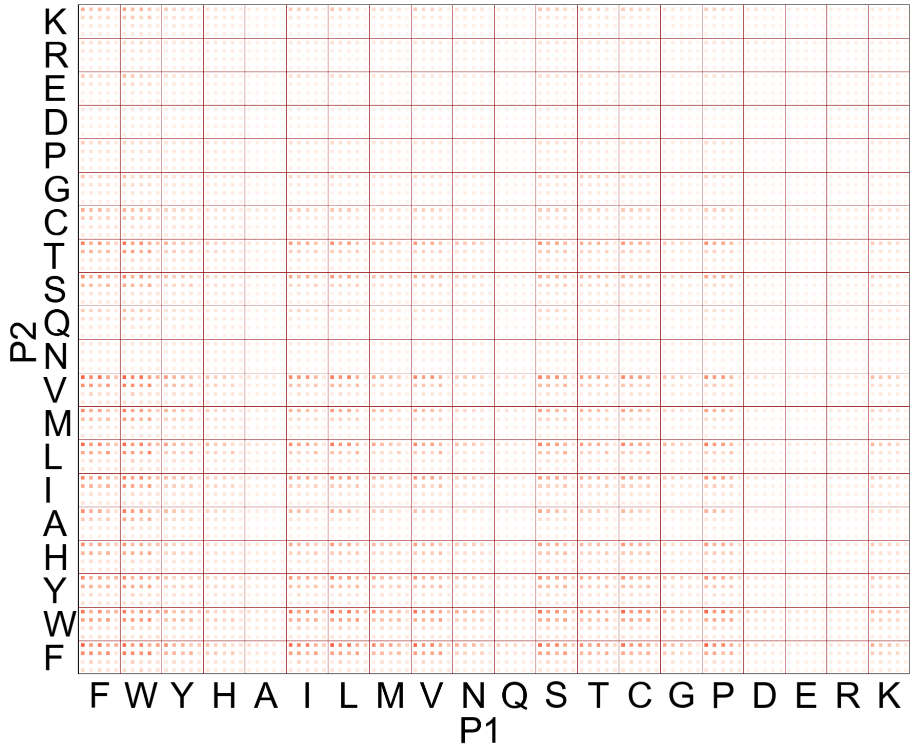

Supplementary Figure 12. Distribution of 8000 AP_{HC} with G fixed at C termini (P4).

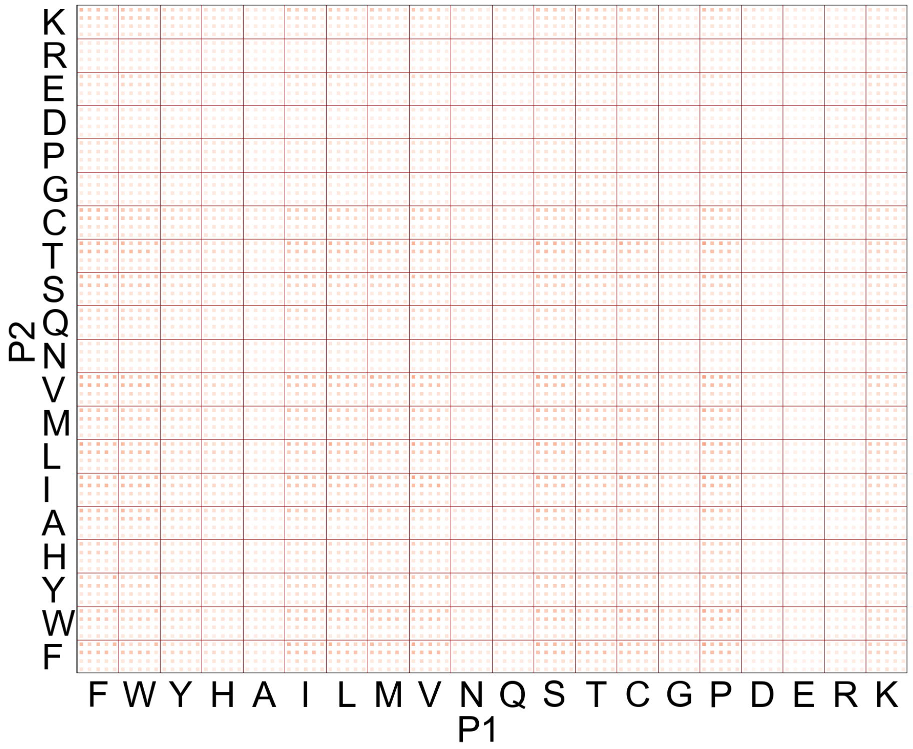

Supplementary Figure 13. Distribution of 8000 AP_{HC} with H fixed at C termini (P4).

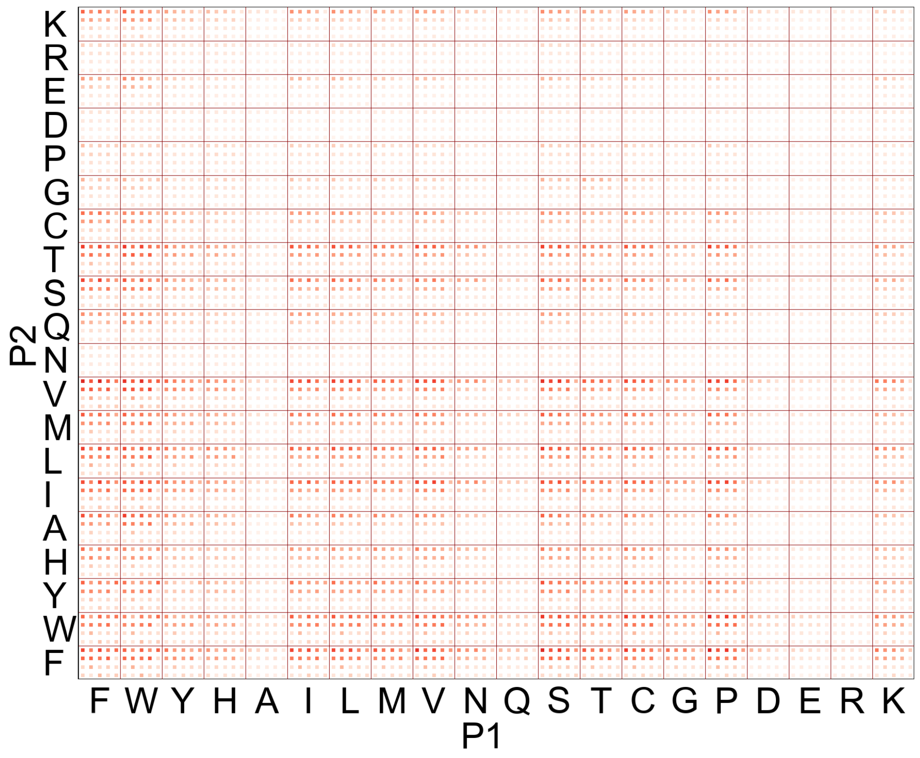

Supplementary Figure 14. Distribution of 8000 AP_{HC} with I fixed at C termini (P4).

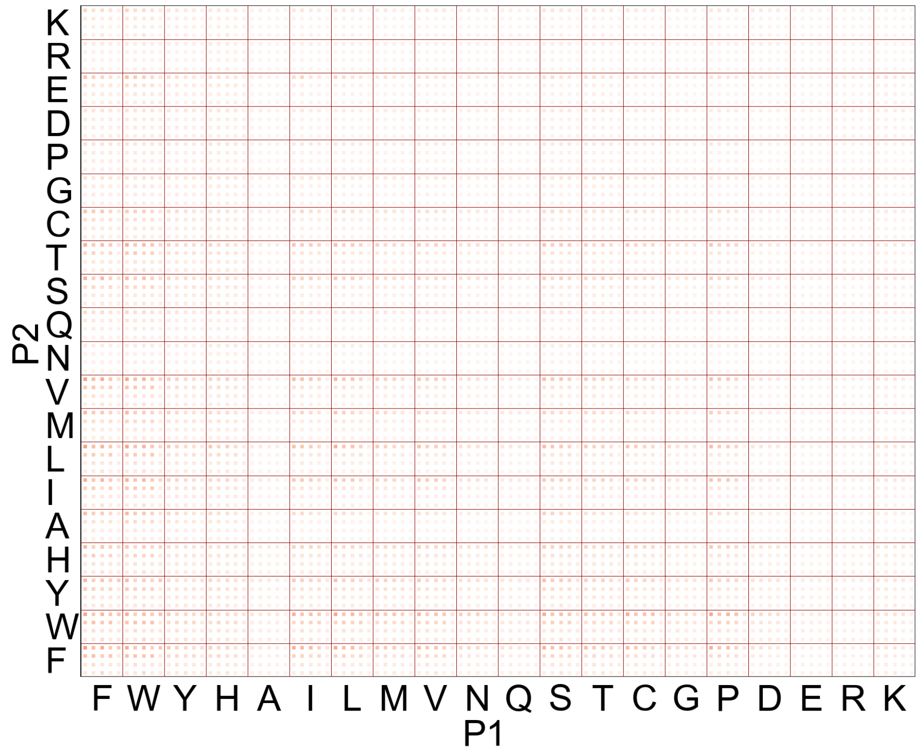

Supplementary Figure 15. Distribution of 8000 AP_{HC} with K fixed at C termini (P4).

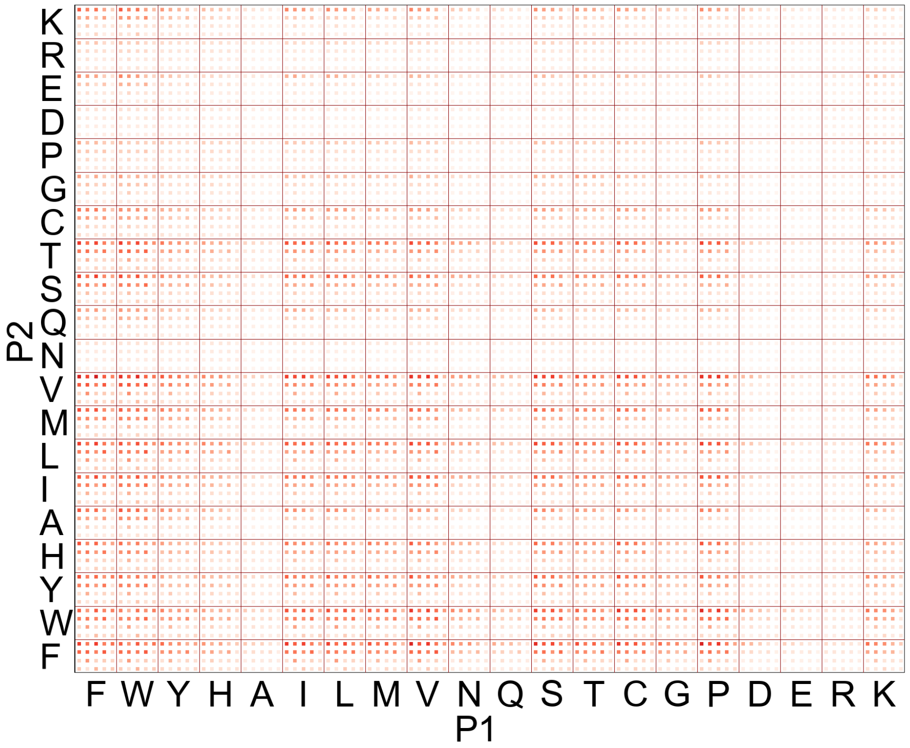

Supplementary Figure 16. Distribution of 8000 AP_{HC} with L fixed at C termini (P4).

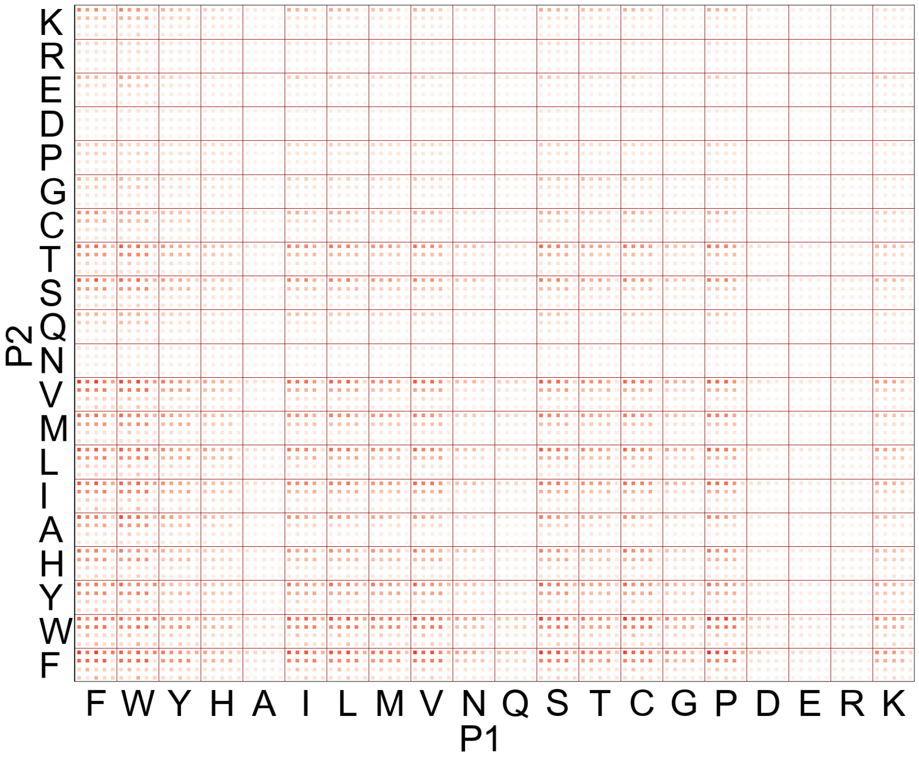

Supplementary Figure 17. Distribution of 8000 AP_{HC} with M fixed at C termini (P4).

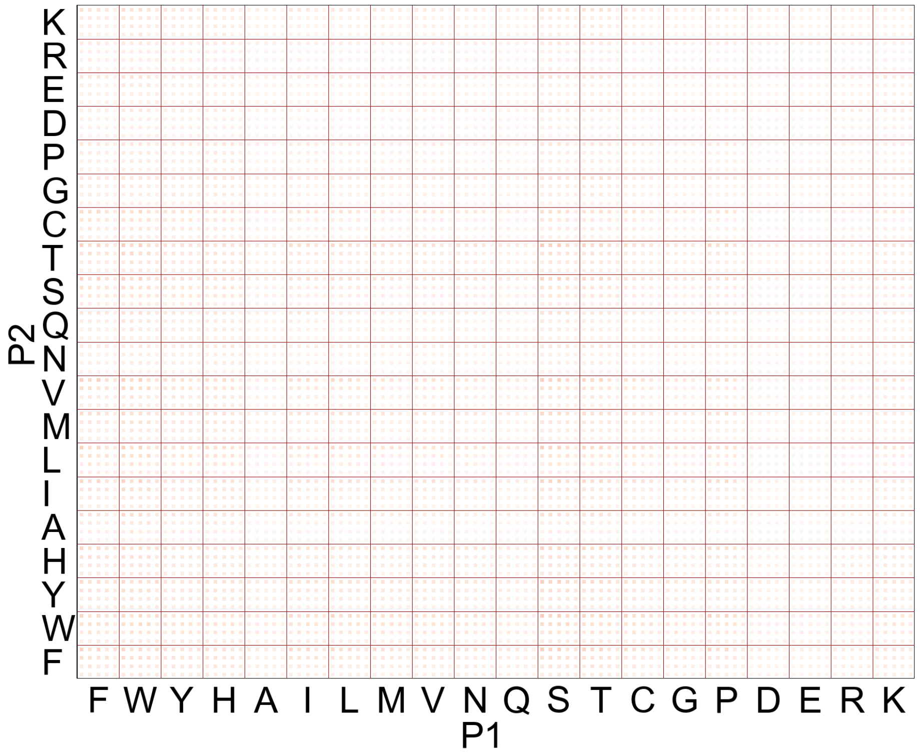

Supplementary Figure 18. Distribution of 8000 AP_{HC} with N fixed at C termini (P4).

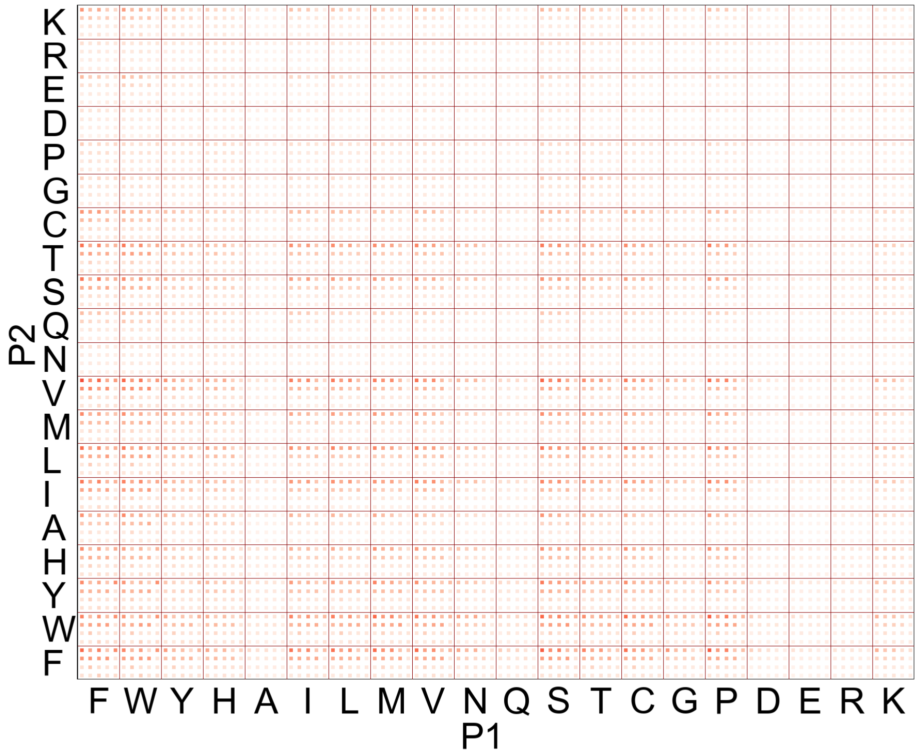

Supplementary Figure 19. Distribution of 8000 AP_{HC} with P fixed at C termini (P4).

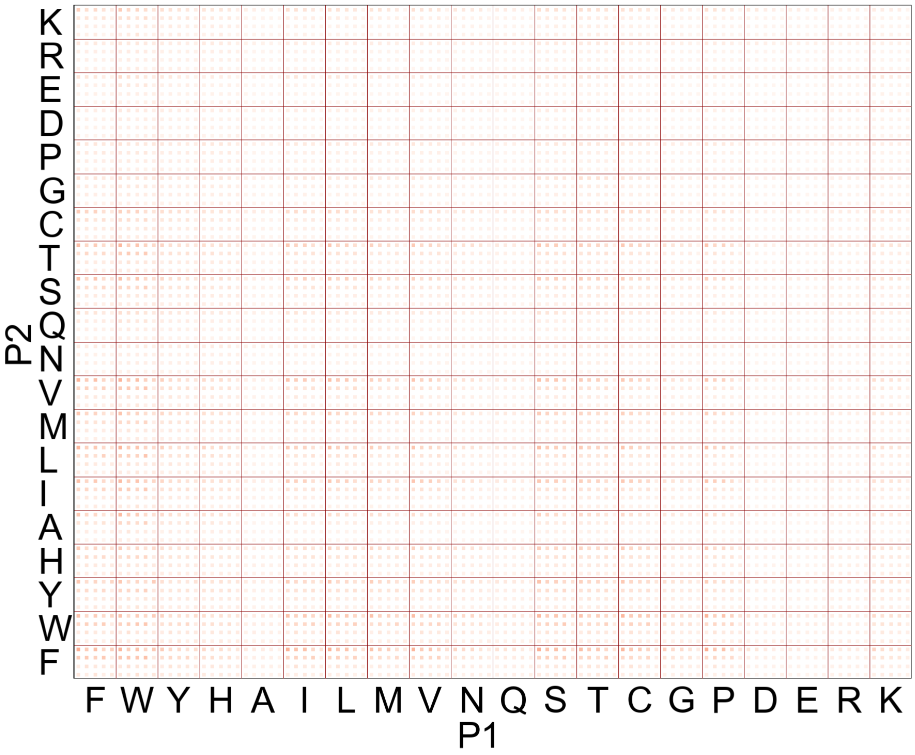

Supplementary Figure 20. Distribution of 8000 AP_{HC} with Q fixed at C termini (P4).

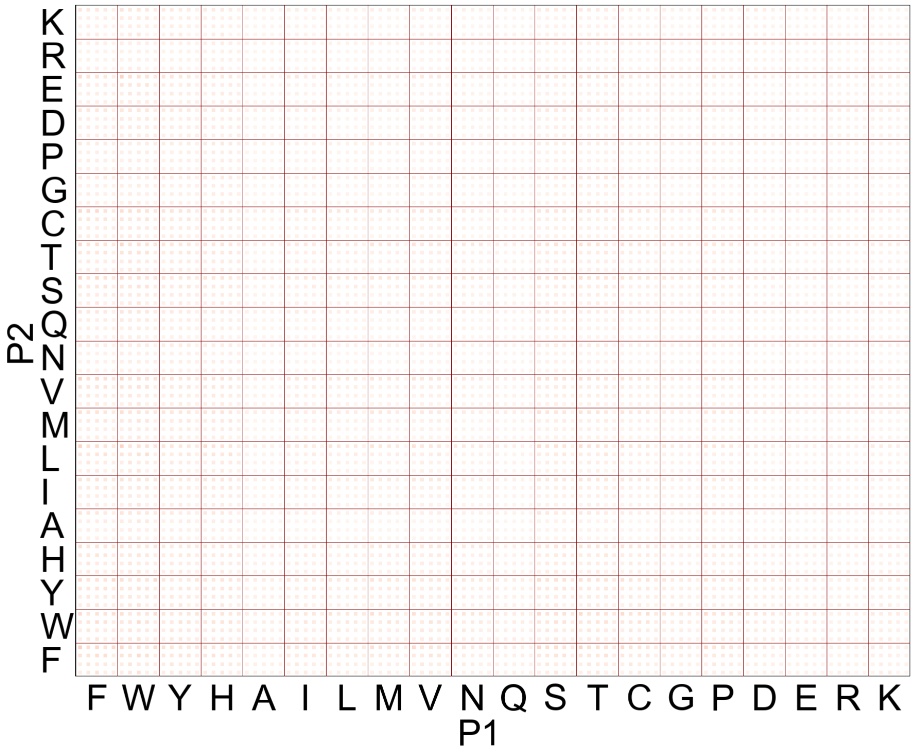

Supplementary Figure 21. Distribution of 8000 AP_{HC} with R fixed at C termini (P4).

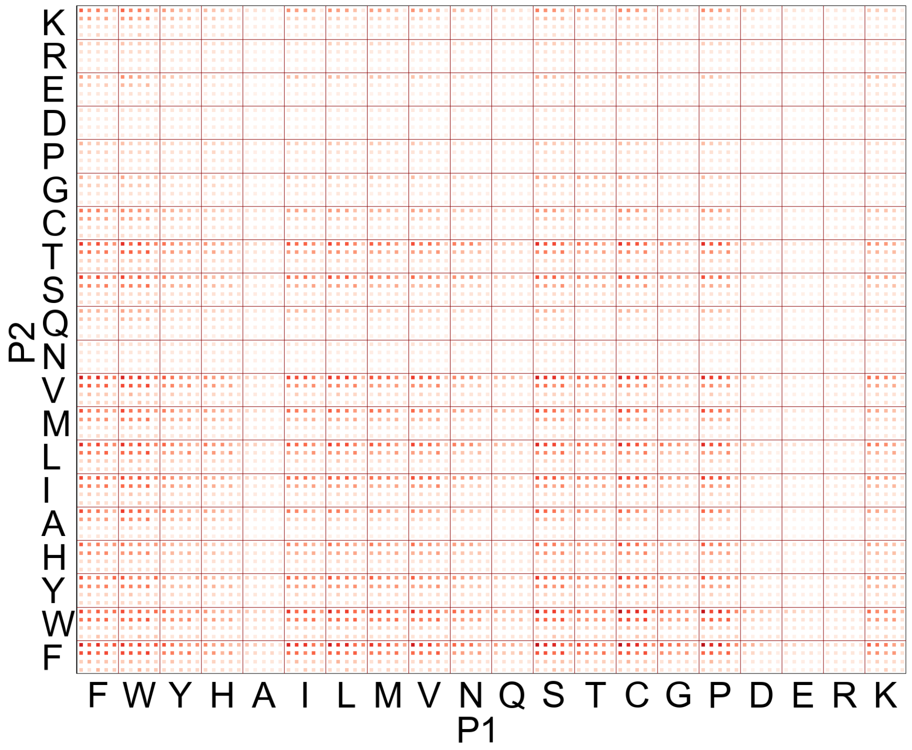

Supplementary Figure 22. Distribution of 8000 AP_{HC} with S fixed at C termini (P4).

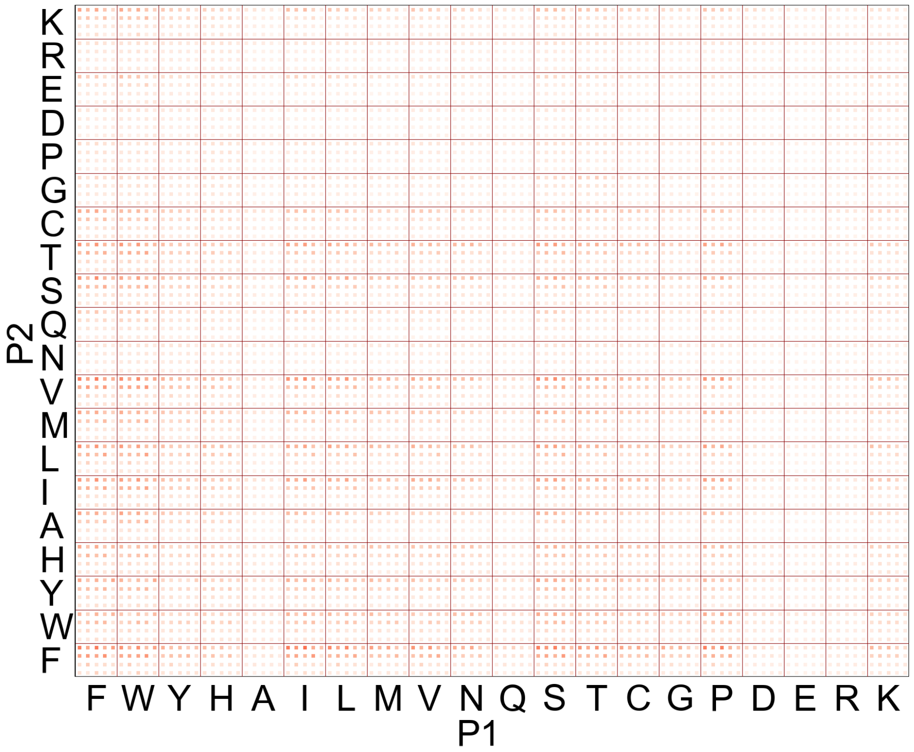

Supplementary Figure 23. Distribution of 8000 AP_{HC} with T fixed at C termini (P4).

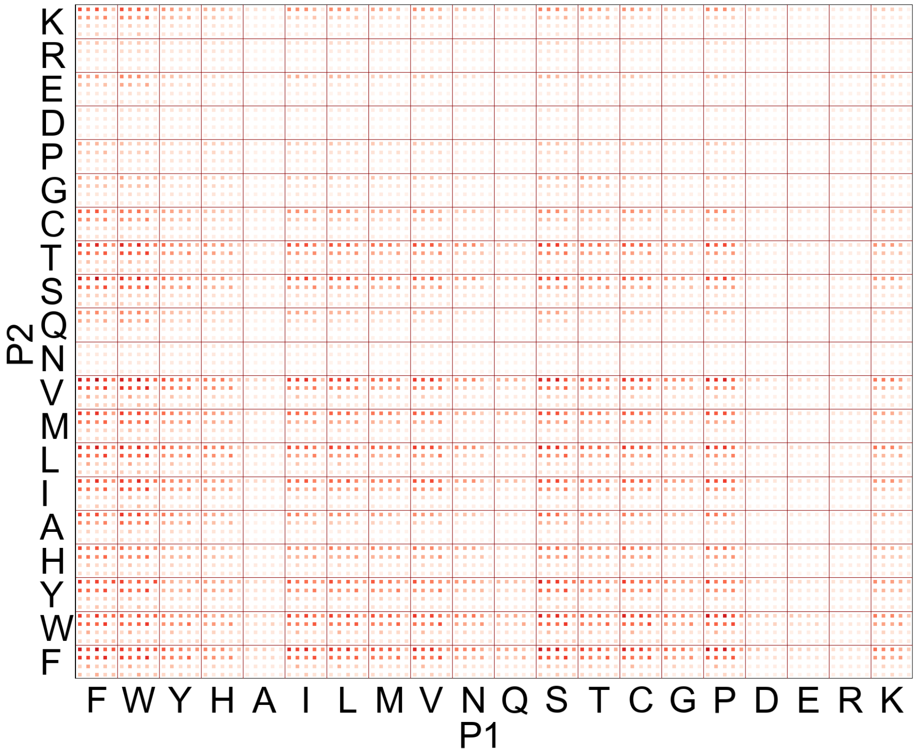

Supplementary Figure 24. Distribution of 8000 AP_{HC} with V fixed at C termini (P4).

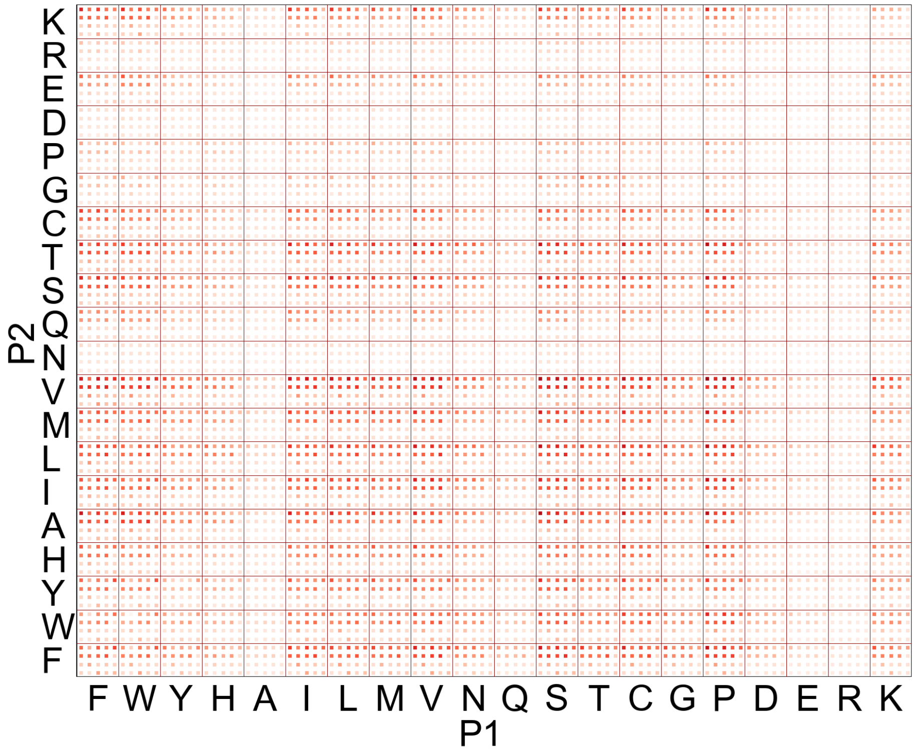

Supplementary Figure 25. Distribution of 8000 AP_{HC} with W fixed at C termini (P4).

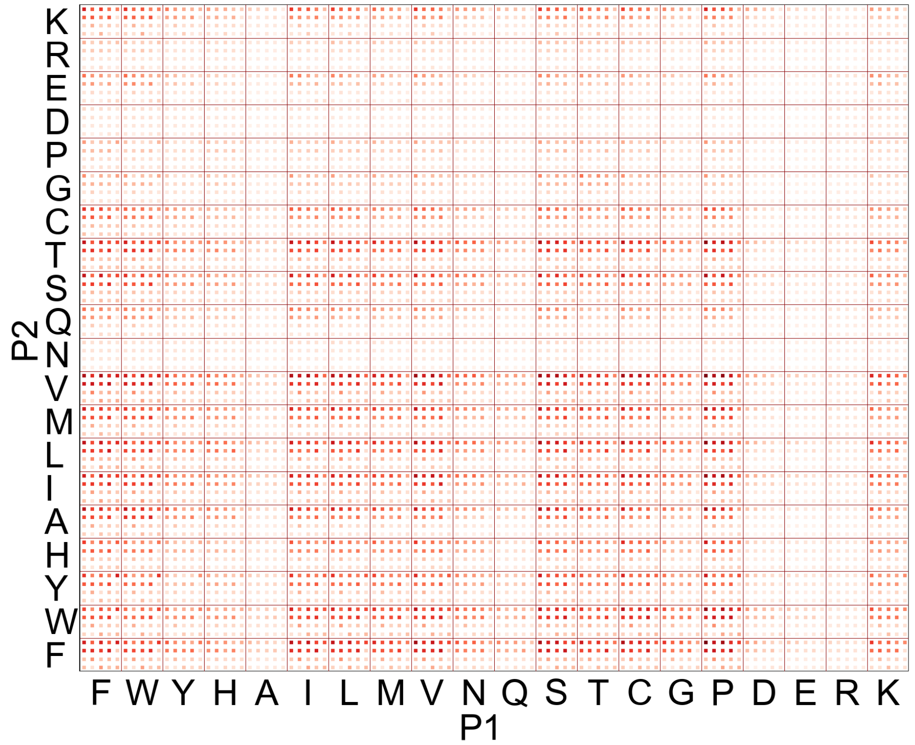

Supplementary Figure 26. Distribution of 8000 AP_{HC} with Y fixed at C termini (P4).

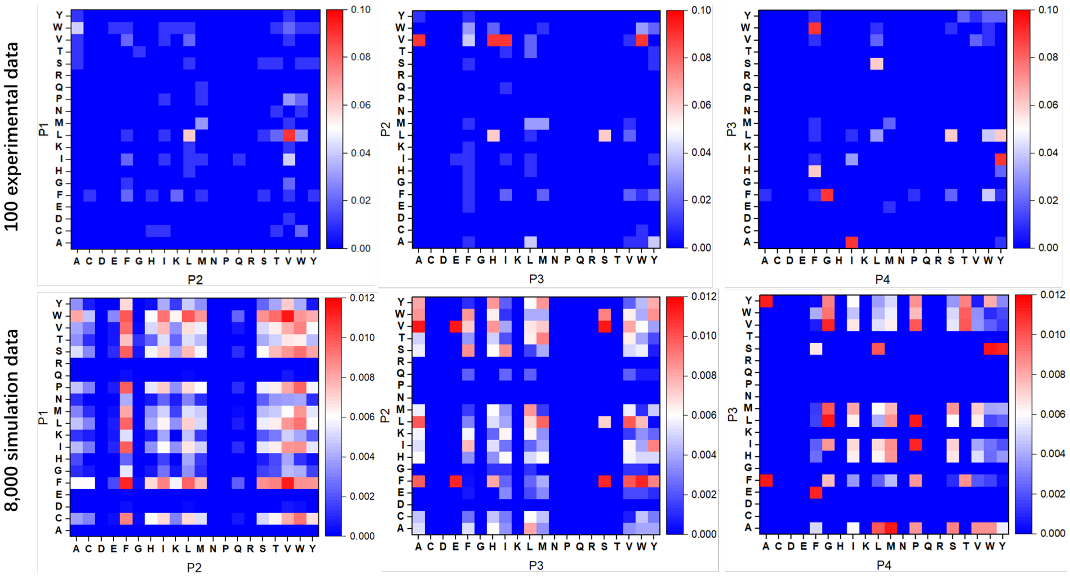

Supplementary Figure 27. Percentage of amino acid pairs (*i.e.*, dipeptide) of 100 hydrogel-forming peptides and 8,000 peptides with top AP_{HC} score in simulations.

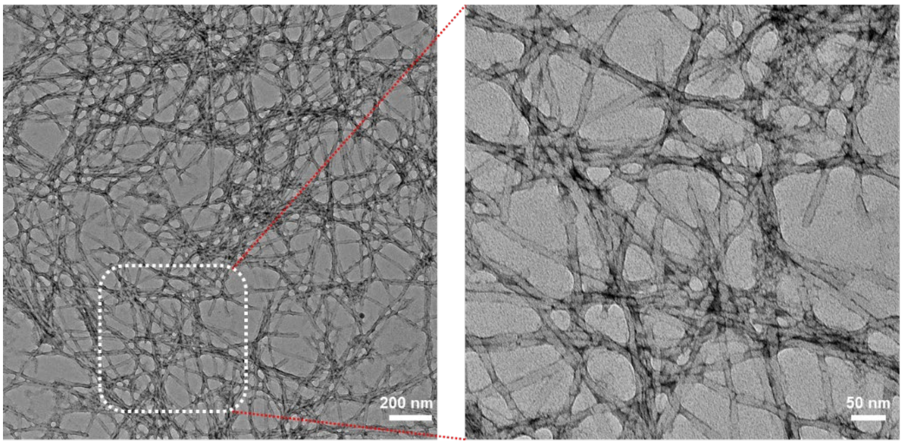

Supplementary Figure 28. TEM images of the hydrogel of YAWF (60 mM).

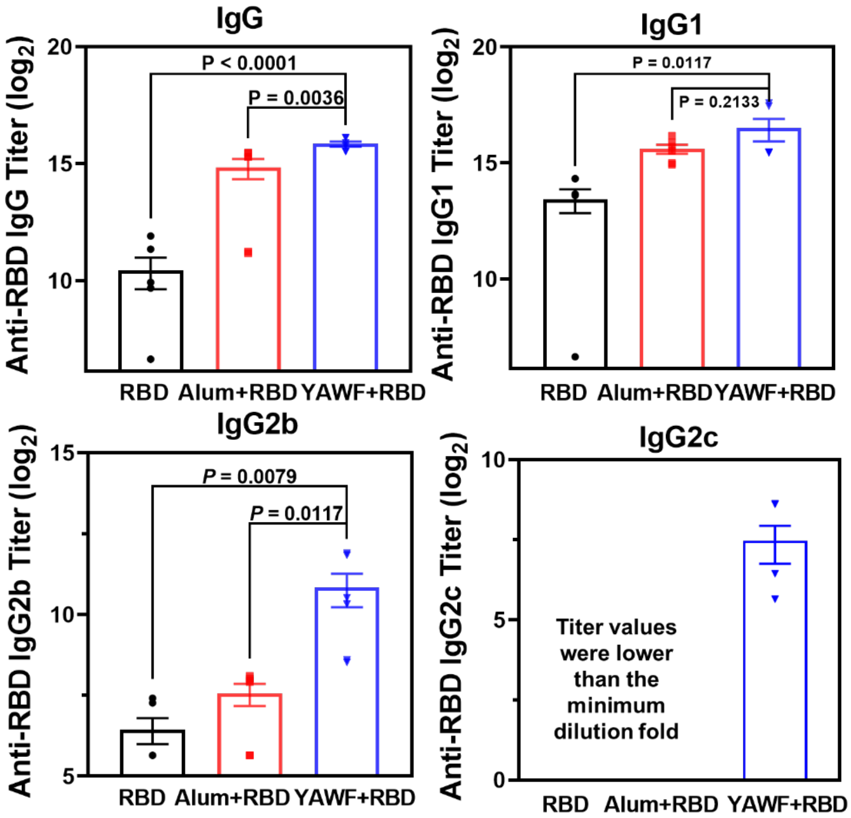

Supplementary Figure 29. The titer of RBD-specific IgG, IgG1, IgG2b, and IgG2c antibodies in serum samples on day 21 were quantified by enzyme-linked immunosorbent assay (ELISA). The data were shown as the mean ± SEM (n=6 biologically independent mice), and differences between RBD and other treatments were determined using one-way ANOVA test.

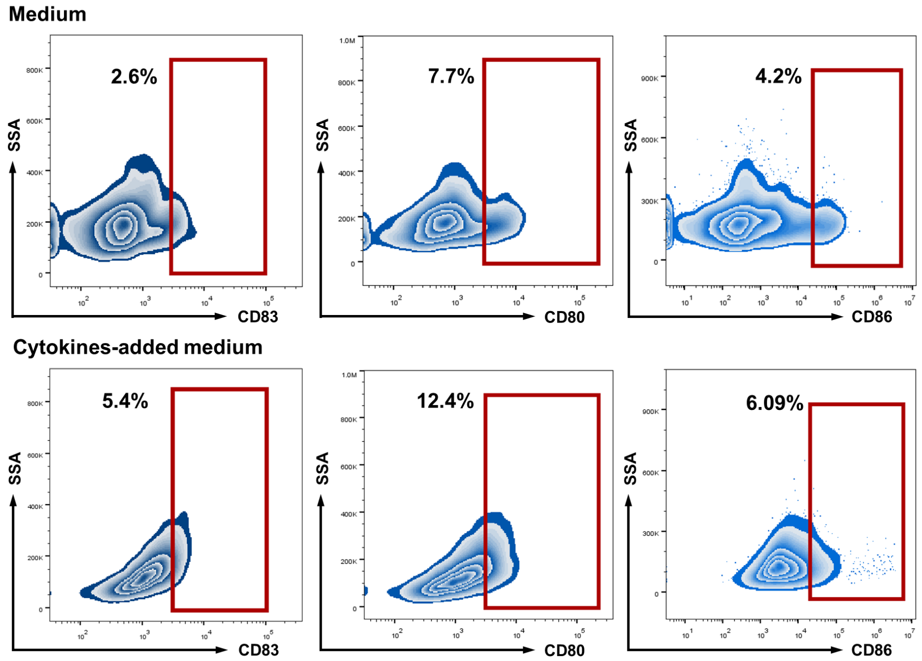

Supplementary Figure 30. Flow cytometry analysis of BMDCs of medium group expressing CD83, CD80, and CD86.

Supplementary Tables

Supplementary Table 1. HPLC elution gradient

Time (min)	Flow (mL/min)	Water% (A)	Acetonitrile% (B)
0	10.0	95	5
11	10.0	20	80
13	10.0	0	100
16	10.0	0	100
17	10.0	95	5
20	10.0	95	5

Supplementary Table 2. Training (MAE_{tr} and R^2_{tr}) and testing (MAE_{te} and R^2_{te}) performance of different ML algorithms and number of training data, with 80-bit data representation with amino acid composition and 4-integer representation of peptides. Since the 4-integer representation yields much worse training performance than the 80-bit approach, it was abandoned immediately and thus was not proceeded with testing. The training performance of MAE_{tr} and R^2_{tr} with 80-bit representation are averaged results over ten parallel ML experiment, shown in Supplementary Data 1.

Peptide Representation	# Training data	Algorithm	MAE_{tr}	R^2_{tr}	MAE_{te}	R^2_{te}
**80-bit representation (one-hot representation)**	**1,000**	LR	0.155	0.804	0.154	0.800
		NN	0.255	0.513	0.242	0.543
		RF	0.186	0.728	0.173	0.755
		SVM	0.158	0.804	0.152	0.819
	**5,000**	LR	0.146	0.823	0.150	0.813
		NN	0.227	0.579	0.219	0.597
		RF	0.140	0.834	0.133	0.840
		SVM	0.112	0.899	0.109	0.905
	**10,000**	LR	0.147	0.821	0.150	0.813
		NN	0.196	0.693	0.184	0.721
		RF	0.119	0.871	0.113	0.881
		**SVM**	**0.095**	**0.928**	**0.092**	**0.933**
**4-integer representation**	**1,000**	LR	0.332	0.142	-	-
		NN	0.319	0.186	-	-
		RF	0.229	0.564	-	-
		SVM	0.306	0.240	-	-
	**5,000**	LR	0.339	0.147	-	-
		NN	0.280	0.380	-	-
		RF	0.192	0.701	-	-
		SVM	0.290	0.357	-	-
	**10,000**	LR	0.336	0.147	-	-
		NN	0.263	0.434	-	-
		RF	0.168	0.764	-	-
		SVM	0.281	0.382	-	-

Supplementary Table 3. Representation of amino acid sequence of a tetrapeptide (taking EHNT as an example) by two approaches. First row is the single-letter representation of 20 amino acids, second row is the corresponding integer for each amino acid, and fourth row is the 4-integer representation of tetrapeptide EHNT, while the fifth row is the 80-bit one-hot representation of EHNT with amino acid composition encoding.

A	C	D	E	F	G	H	I	K	L	M	N	P	Q	R	S	T	V	W	Y
1	2	3	4	5	6	7	8	9	10	11	12	13	14	15	16	17	18	19	20

Position 1	Position 2	Position 3	Position 4
**4**	**7**	**12**	**19**
000**1**00000…00000000<br>Forth bit	000000**1**00…00000000<br>Seventh bit	00000000000**1**…0000<br>Twelfth bit	0000…00000000000**1**0<br>Nineteenth bit

Supplementary Table 4. Training (MAE_{tr} and R^2_{tr}) and testing (MAE_{te} and R^2_{te}) performance of four algorithms with three different number of training data and four training algorithms. The training data is represented with one-hot approach using 1200-bit converted from dipeptide composition.

Peptide Representation	# Training data	Algorithm	MAE_{tr}	R^2_{tr}	MAE_{te}	R^2_{te}
1200-bit representation (one-hot representation)	1,000	LR	>100	< 0	-	-
		NN	0.318	0.216	0.280	0.374
		RF	0.280	0.384	0.275	0.380
		SVM	0.296	0.362	0.282	0.400
	5,000	LR	0.147	0.819	0.140	0.832
		NN	0.258	0.470	0.250	0.487
		RF	0.193	0.689	0.185	0.704
		SVM	0.184	0.742	0.176	0.764
	10,000	LR	0.132	0.855	0.129	0.858
		NN	0.250	0.501	0.242	0.522
		RF	0.168	0.756	0.161	0.769
		SVM	0.147	0.832	0.140	0.846

Supplementary Table 5. Default hyperparameters and values for each algorithm.

**Algorithm**	**Hyperparameters**	**Values**
**LR**	fit_intercept	True
	positive	False
	copy_X	True
	n_jobs	None
**RF**	scaler_option	StandardScaler
	n_estimators	100
	max_features	Auto
	max_depth	None
	min_samples_split	2
	min_samples_leaf	1
	bootstrap	True
	criterion	Mse
	min_weight_fraction_leaf	0
	max_leaf_nodes	None
	min_impurity_decrease	0
**NN**	n_neighbors	5
	weights	Uniform
	algorithm	Auto
	leaf_size	30
	p	2
	metric	Minkowski
	metric_params	None
**SVM**	kernel	rbf
	degree	3
	coef0	0.0
	tol	1e-3
	C	1.0
	epsilon	0.1
	shrinking	True
	gamma	auto

Supplementary Table 6. Kernels and associated parameters and their tuning range for SVM.

**Kernel**	**Parameter and range**
linear	C (0.0001, 0.001, 0.01, 0.1, 1, 10, 100, 1000)
poly	C (0.001, 0.01, 0.1, 1, 10, 100, 1000); degree (1, 2, 3, 4)
rbf	C (0.001, 0.01, 0.1, 1, 10, 100, 1000); gamma (0.001, 0.01, 0.1, 1)
