# Supplementary material for: Accelerating the prediction and discovery of peptide hydrogels with human-in-the-loop
Source: Nat Commun. 2023 Jun 30;14:3880. doi: 10.1038/s41467-023-39648-2 (PMC10313671; doi:10.1038/s41467-023-39648-2)
Supplement: Supplementary file 3 — Description of Additional Supplementary Files [file 41467_2023_39648_MOESM3_ESM.pdf]

Description of Additional Supplementary Files

Supplementary Data 1: Training performance (MAE_{tr} and R^2_{tr}) of ten parallel ML experiments, trained based on three different number of datasets and four algorithms with 80-bit one-hot representation.

Supplementary Data 2: The top 8,000 AP_{HC} of tetrapeptides.

Supplementary Data 3: The top 8,000 C_g of tetrapeptides.

Supplementary Data 4: Mass spectrometry of 165 synthetic tetrapeptides.

Supplementary Data 5: Nuclear magnetic resonance spectroscopy and peak location and associated information of 165 synthetic tetrapeptides.

Supplementary Data 6: Transmission electron microscope morphologies of 165 synthetic tetrapeptide hydrogels/solutions.

Supplementary Data 7: Statistical results of gelation experiments of 165 synthetic tetrapeptides
