# Supplementary material for: Accelerating the prediction and discovery of peptide hydrogels with human-in-the-loop
Source: Nat Commun. 2023 Jun 30;14:3880. doi: 10.1038/s41467-023-39648-2 (PMC10313671; doi:10.1038/s41467-023-39648-2)
Supplement: Supplementary file 7 — Supplementary Data 4 [file 41467_2023_39648_MOESM7_ESM.pdf]

1. Compound **LWFW**: obsvd. [M+H$^+$] = 651.4

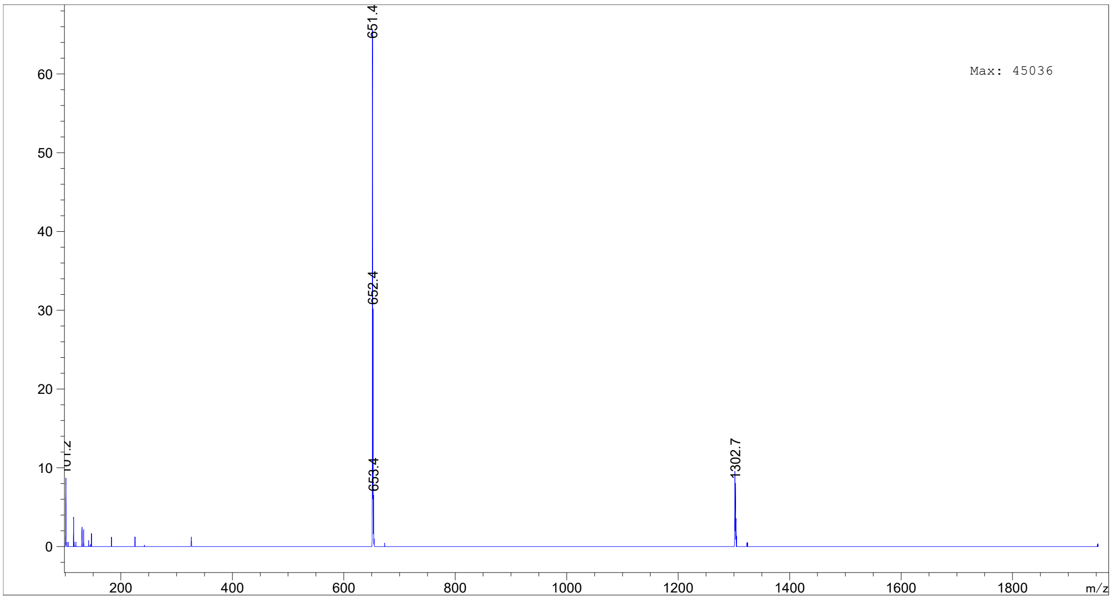

2. Compound **WVFY:** obsvd. [M+H$^+$] = 614.3

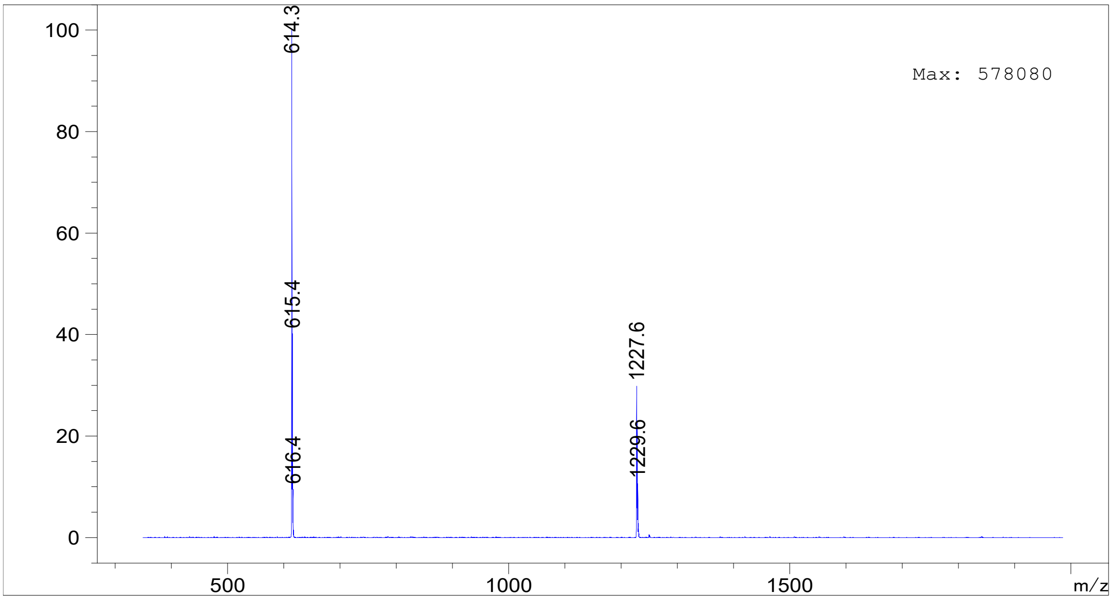

3. Compound **PVFY:** obsvd. [M+H$^+$] = 525.3

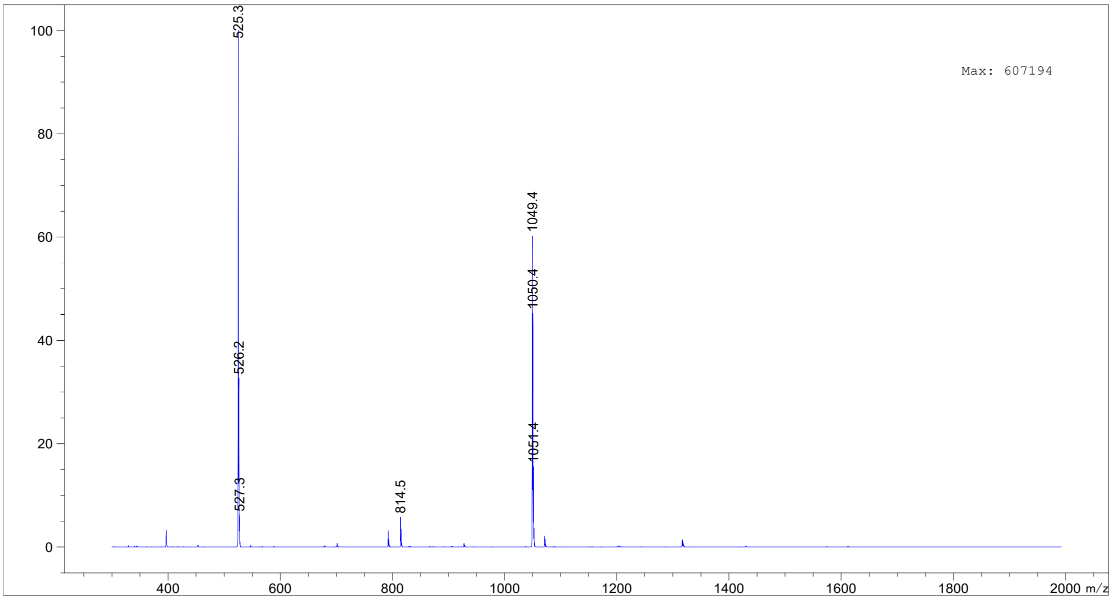

4. Compound **WAYF:** obsvd. [M+H$^+$] = 586.2

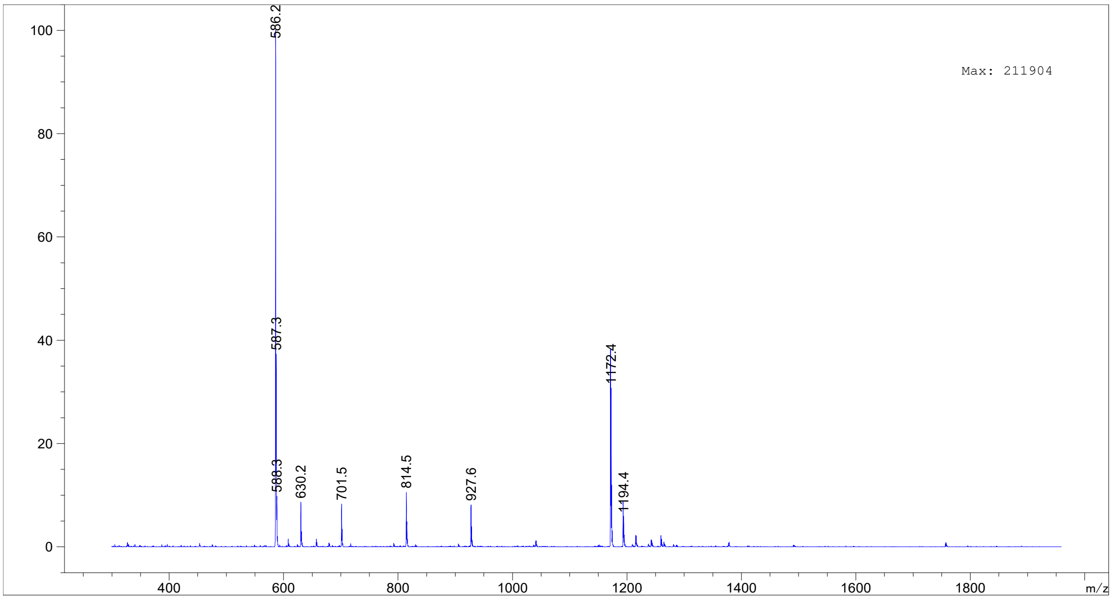

5. Compound **WEFF:** obsvd. [M+H$^+$] = 628.2

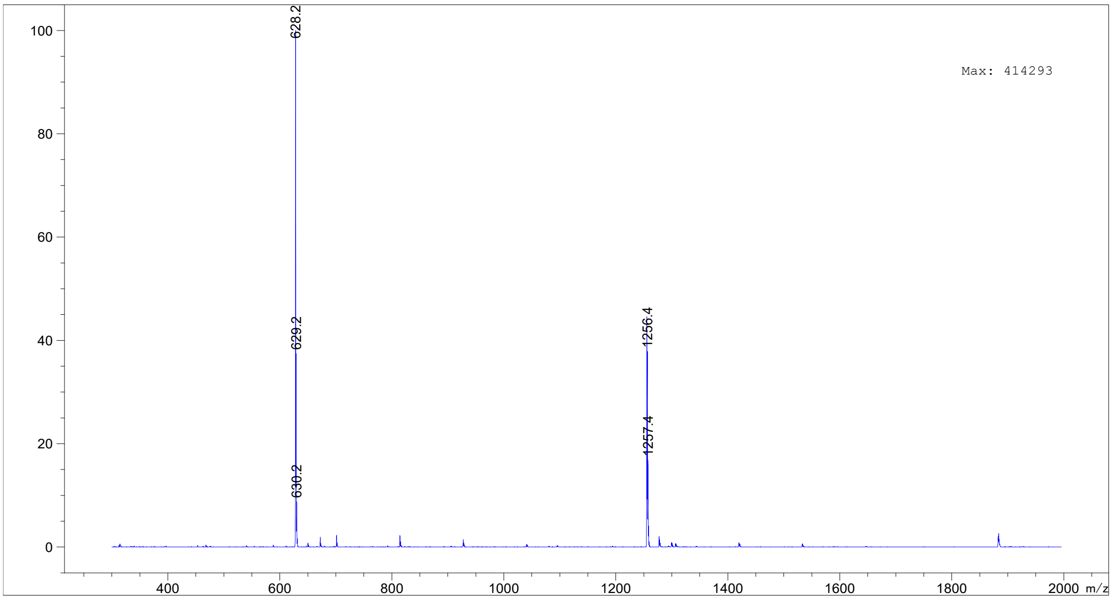

6. Compound **FKFF:** obsvd. [M+H$^+$] = 588.3

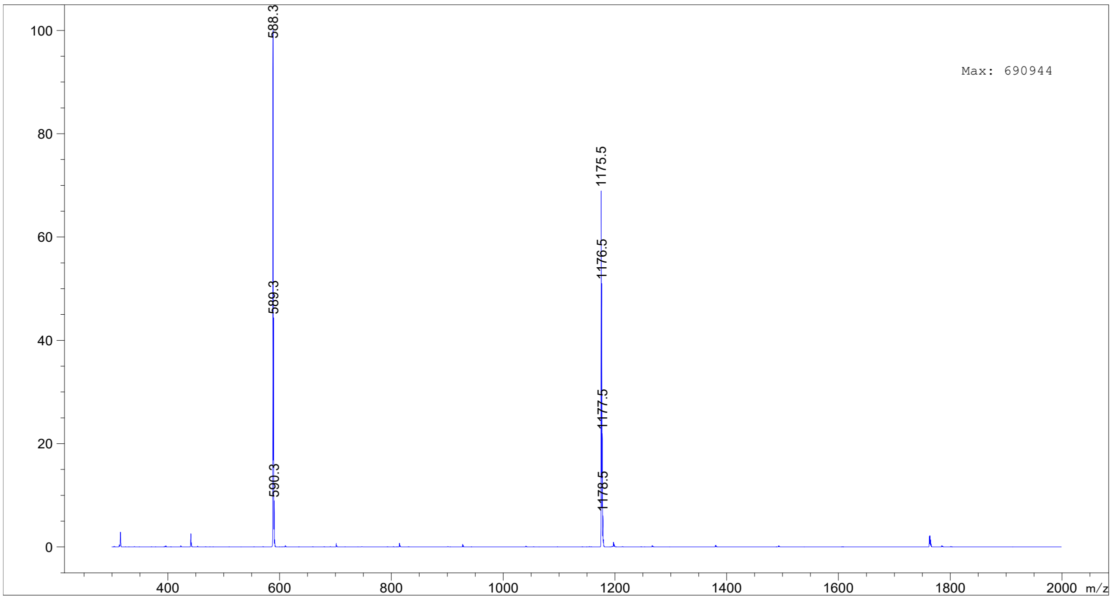

7. Compound **FKFW:** obsvd. [M+TFA$^-$] = 739.3

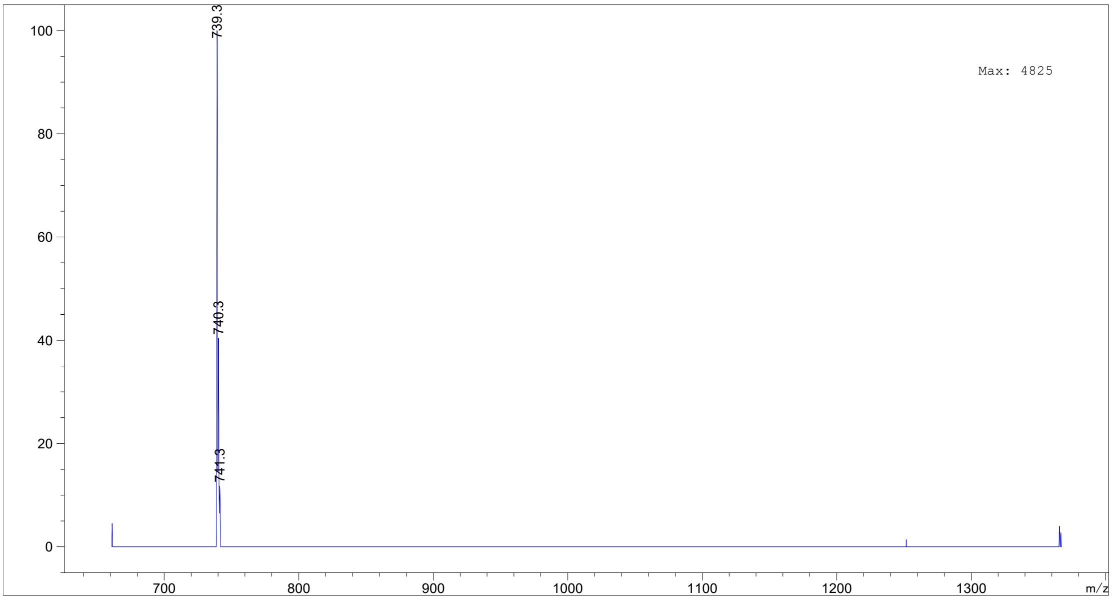

8. Compound **WKFF:** obsvd. [M+ H^+] = 627.3

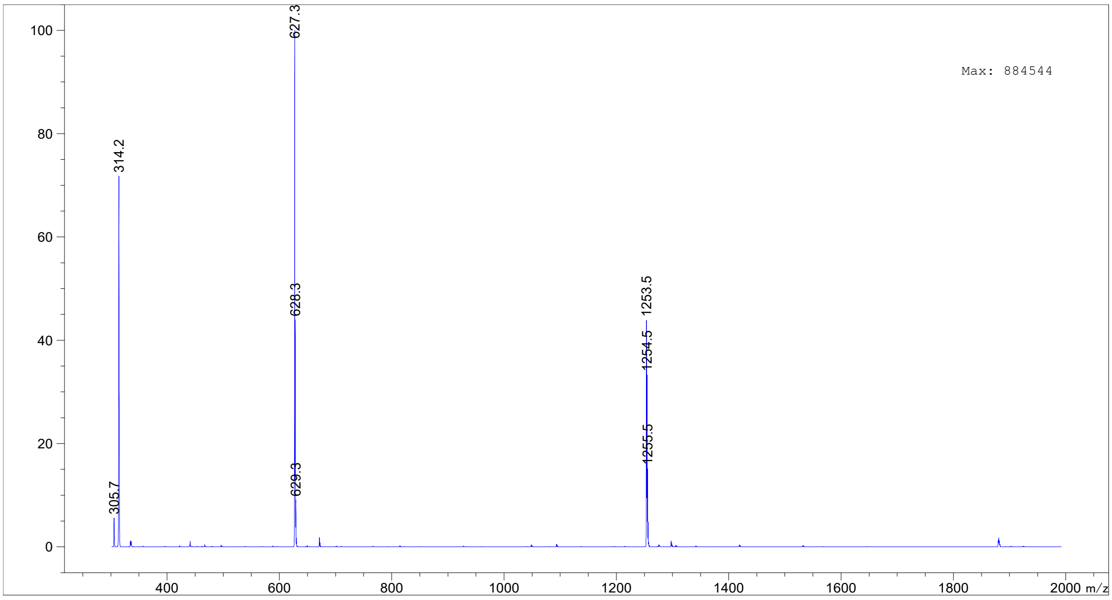

9. Compound **YAWF:** obsvd. [M+ H^+] = 586.2

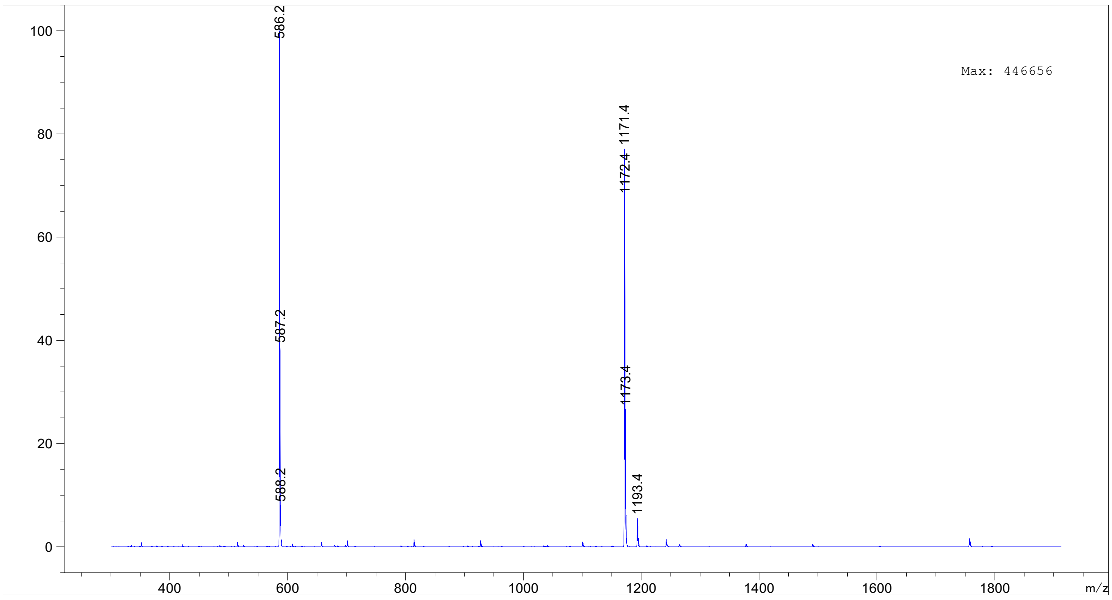

10. Compound **FFFF:** obsvd. [M+ H$^+$] = 607.4

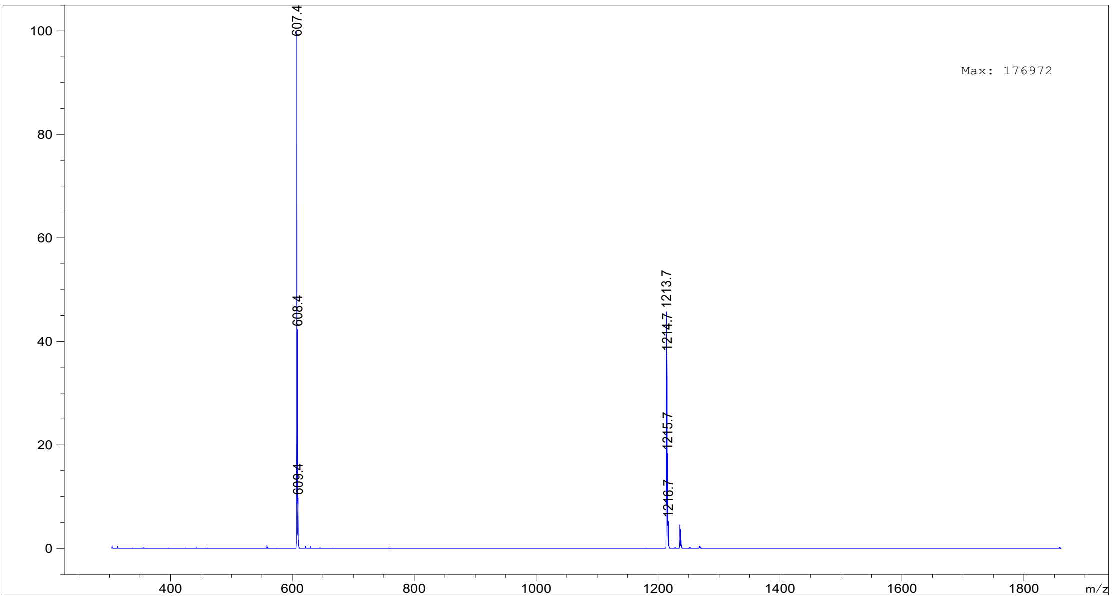

11. Compound **IIII:** obsvd. [M+ H$^+$] = 471.5

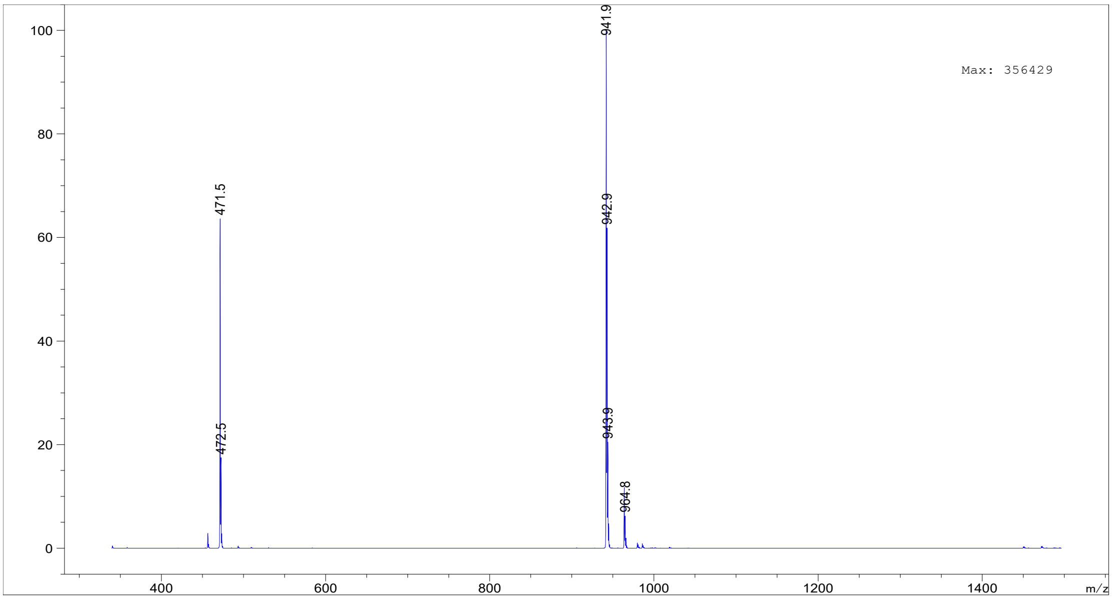

12. Compound **LLLL:** obsvd. $[M+ H^{+}] = 471.4$

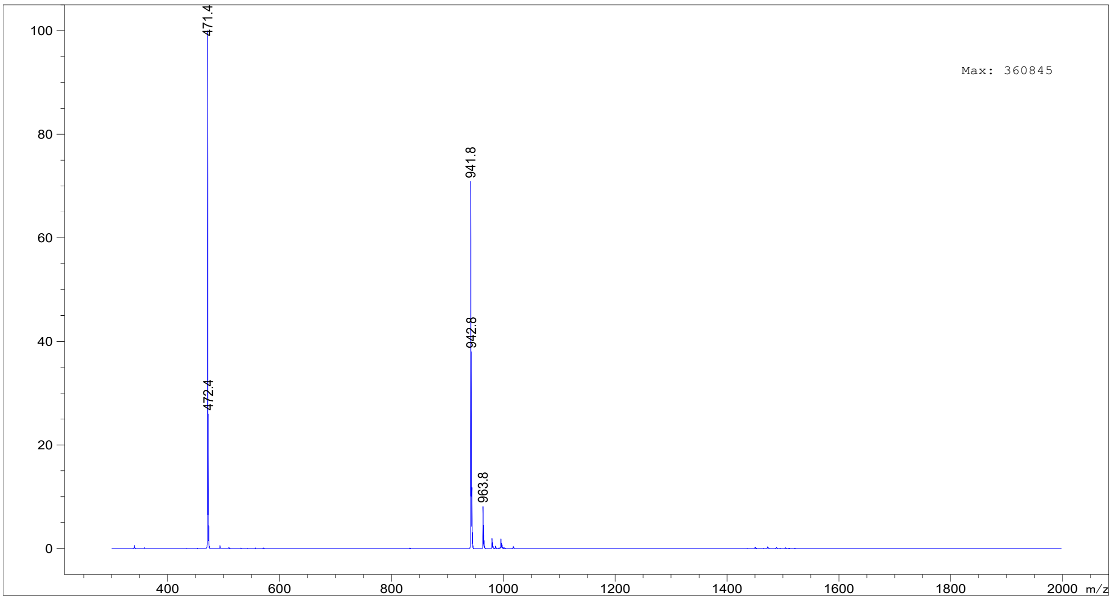

13. Compound **VVVV:** obsvd. $[M+ H^{+}] = 415.4$

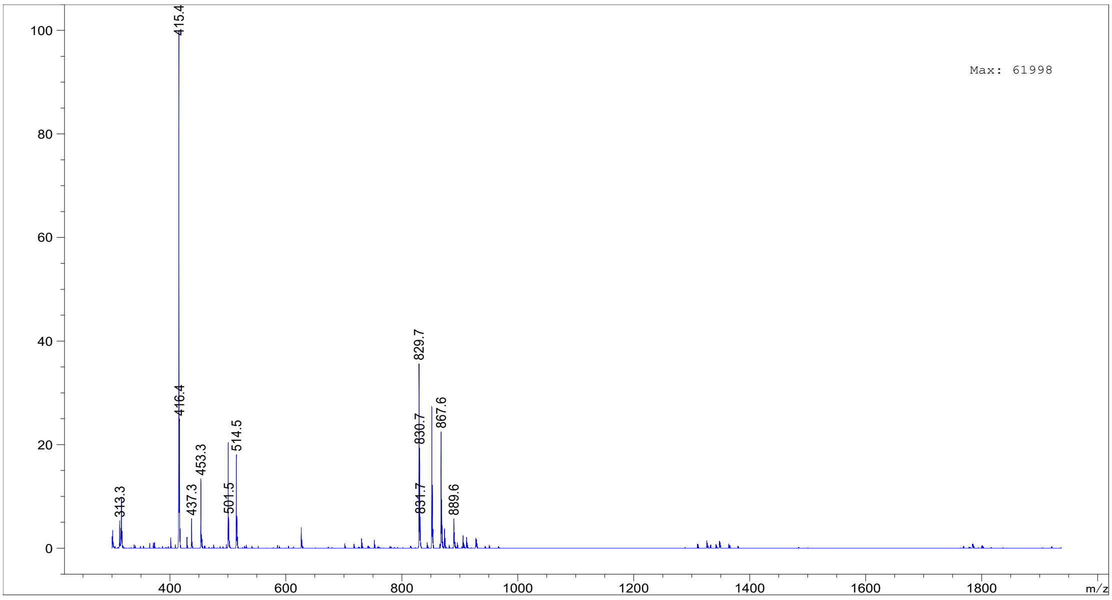

14. Compound **MMMM:** obsvd. [M+ H$^+$] = 543.1

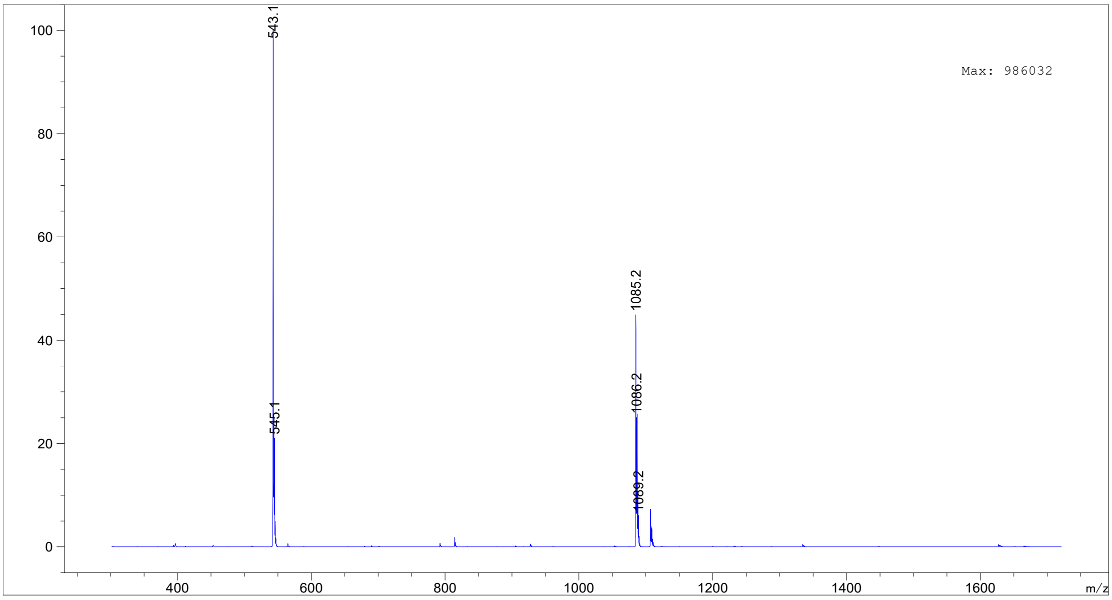

15. Compound **CHFW:** obsvd. [M+ H$^+$] = 592.2

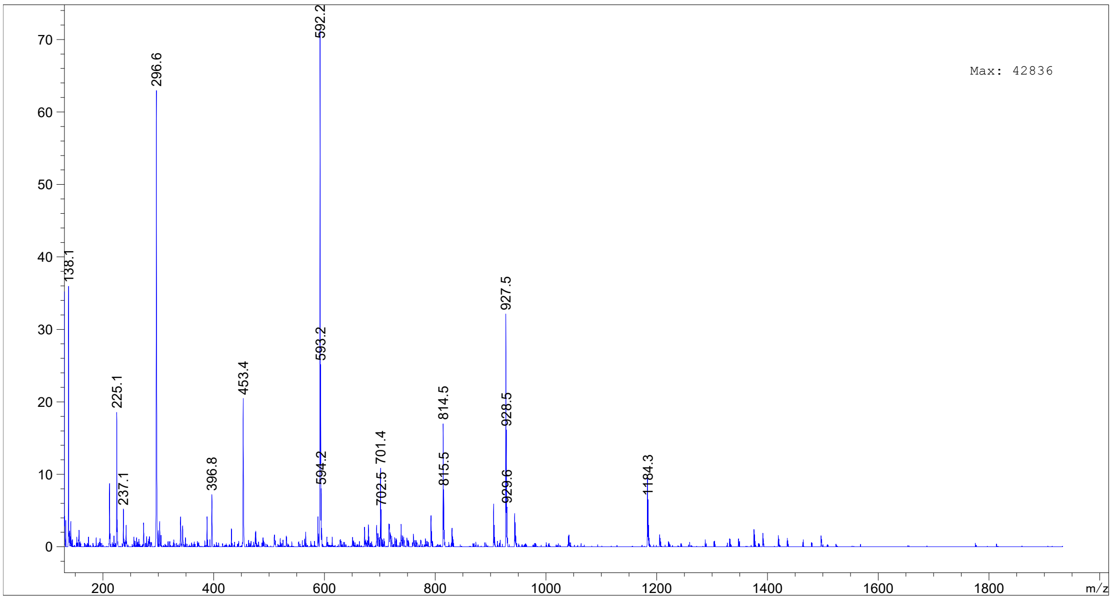

16. Compound **DVFW:** obsvd. [M+ H$^+$] = 566.0

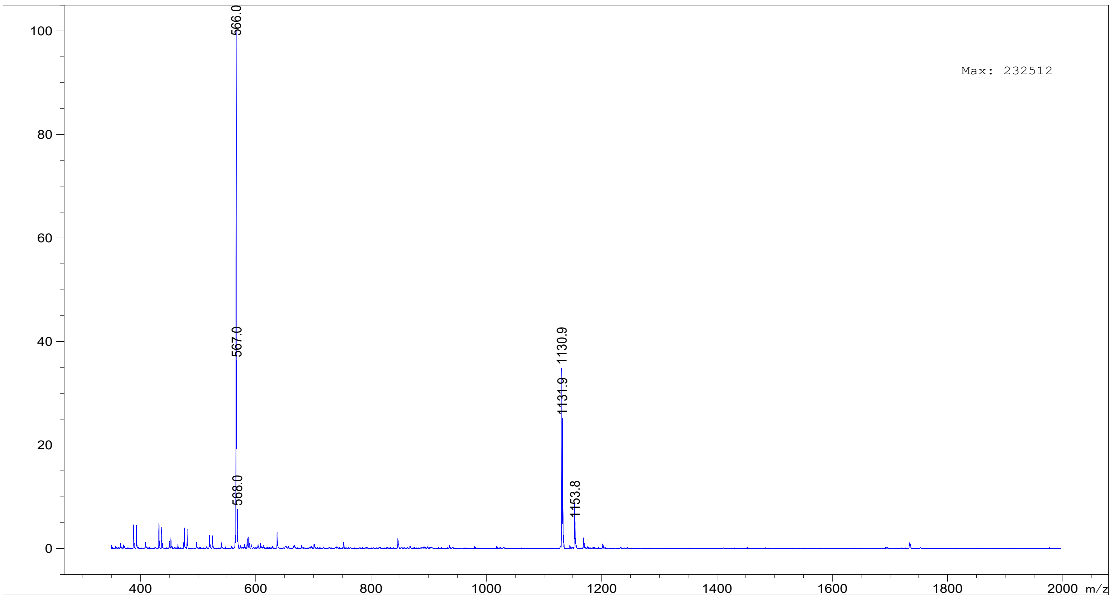

17. Compound **NWWF:** obsvd. [M+ H$^+$] = 551.9

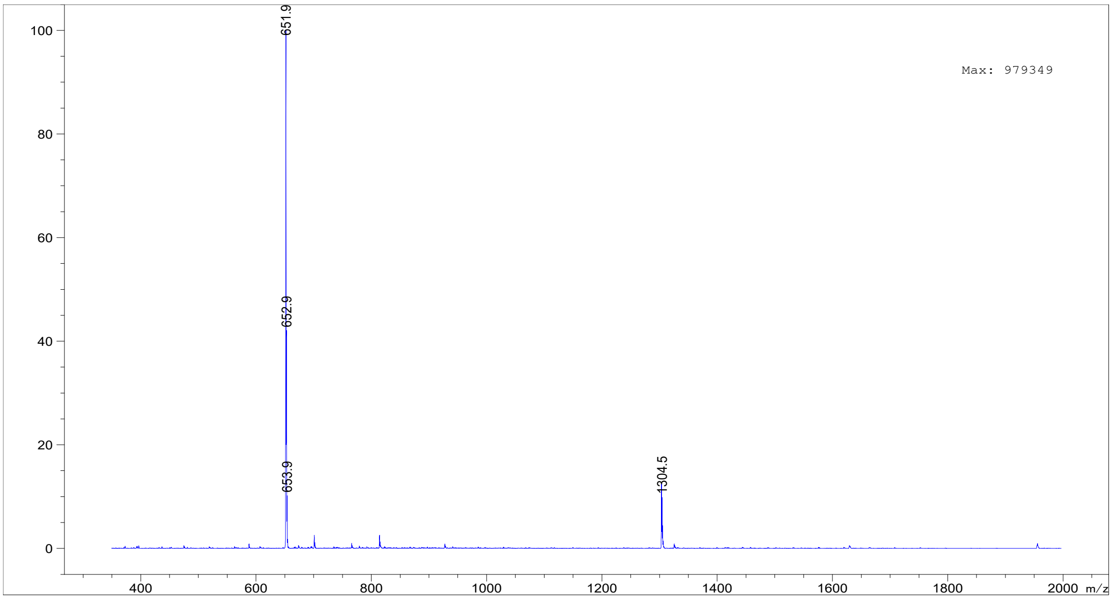

18. Compound **LSFW:** obsvd. [M+ H^{+}] = 552.2

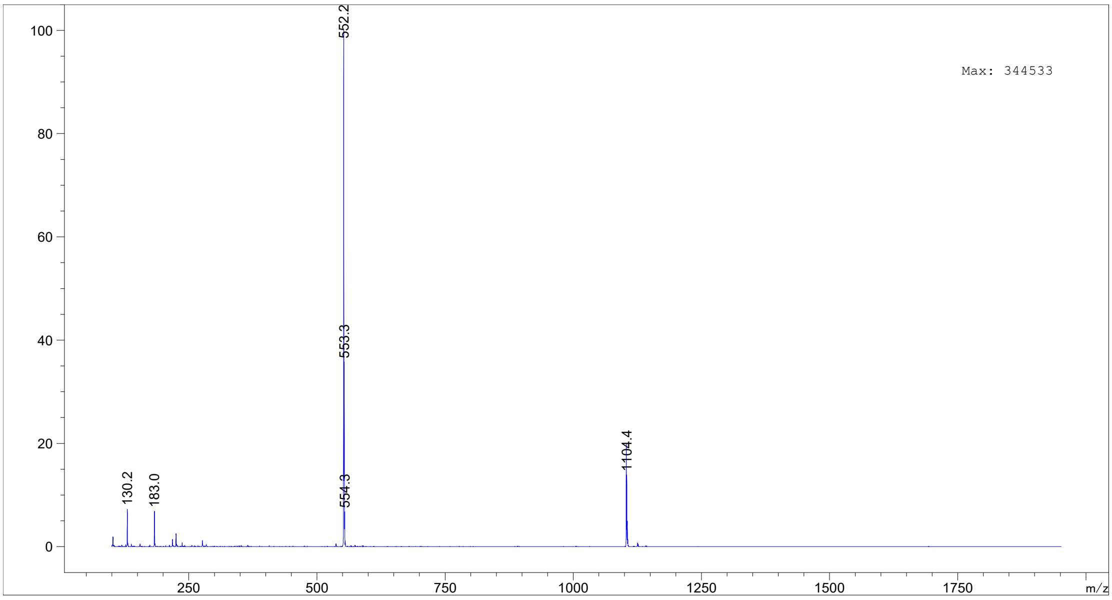

19. Compound **LFWS:** obsvd. [M+ H^{+}] = 552.2

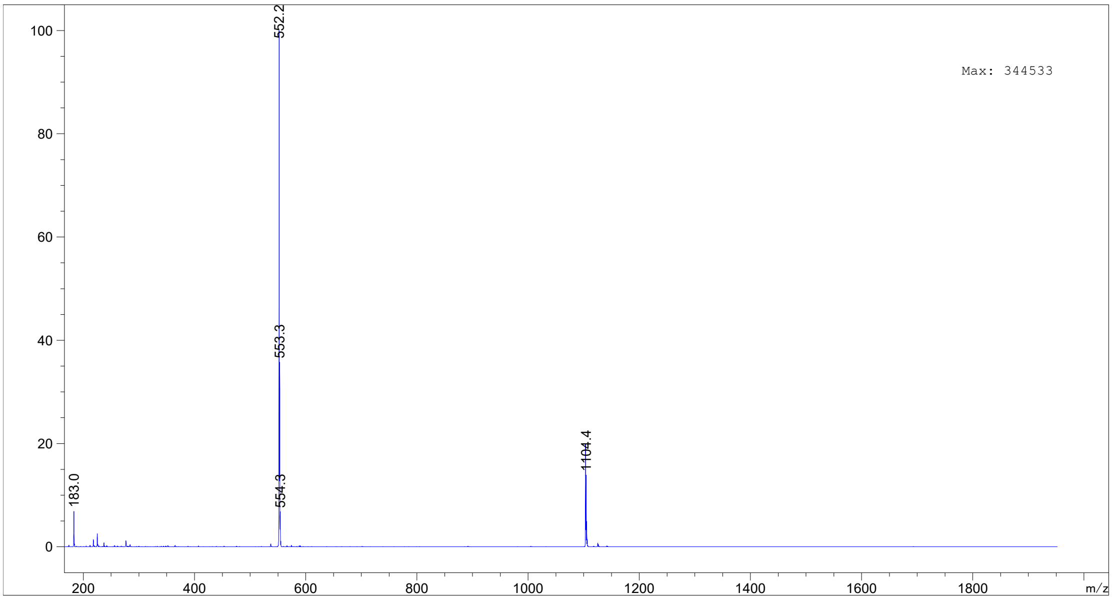

20. Compound **CWHY:** obsvd. [M+ H^+] = 608.2

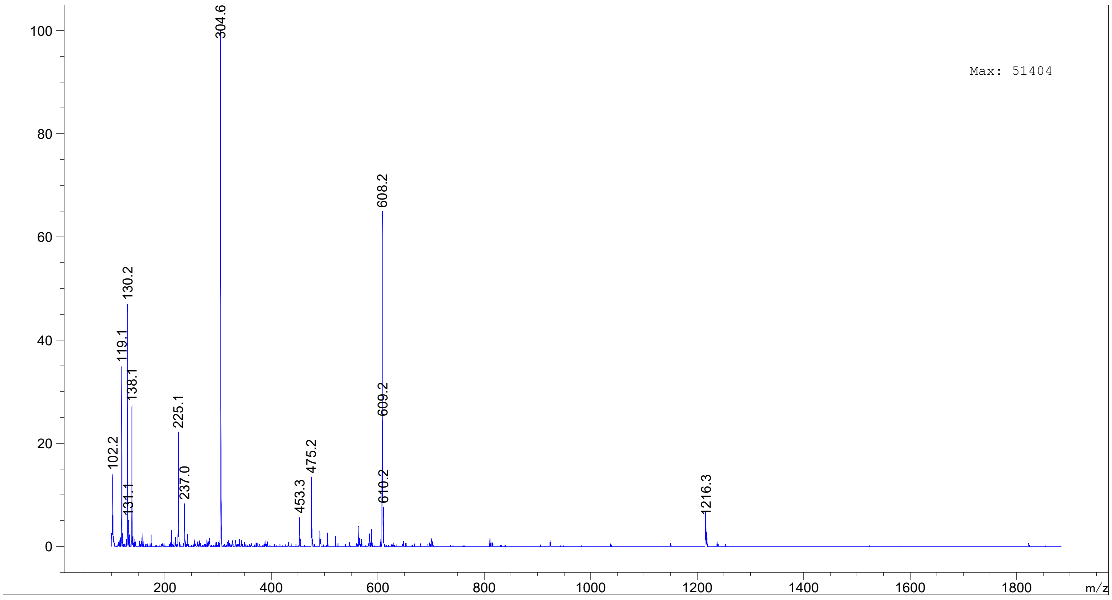

21. Compound **WALW:** obsvd. [M+ H^+] = 575.3

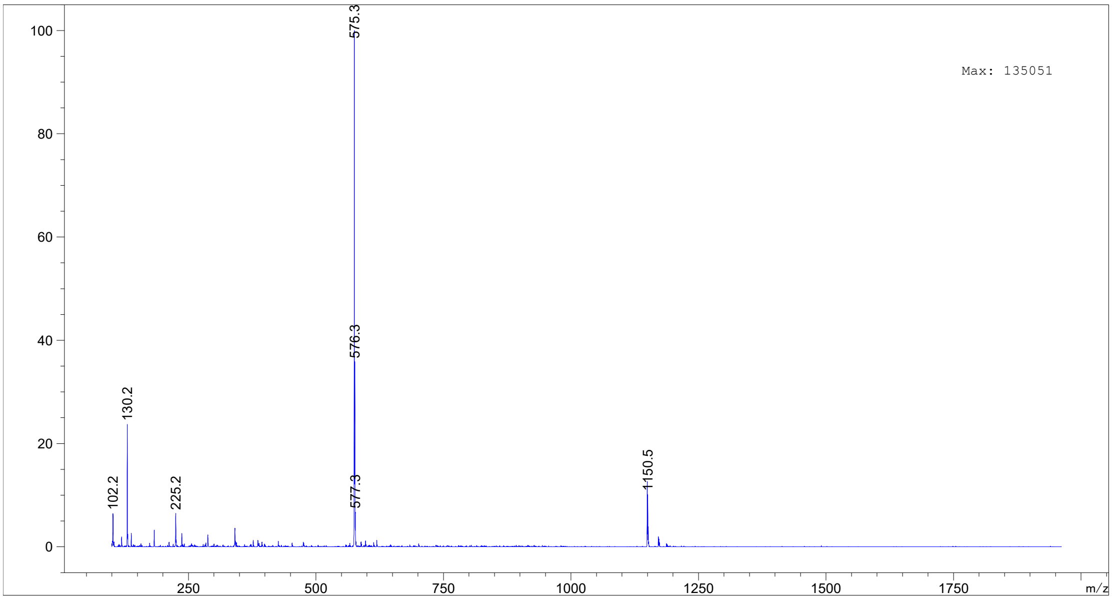

22. Compound **FFYY:** obsvd. [M+ TFA$^-$] = 751.1

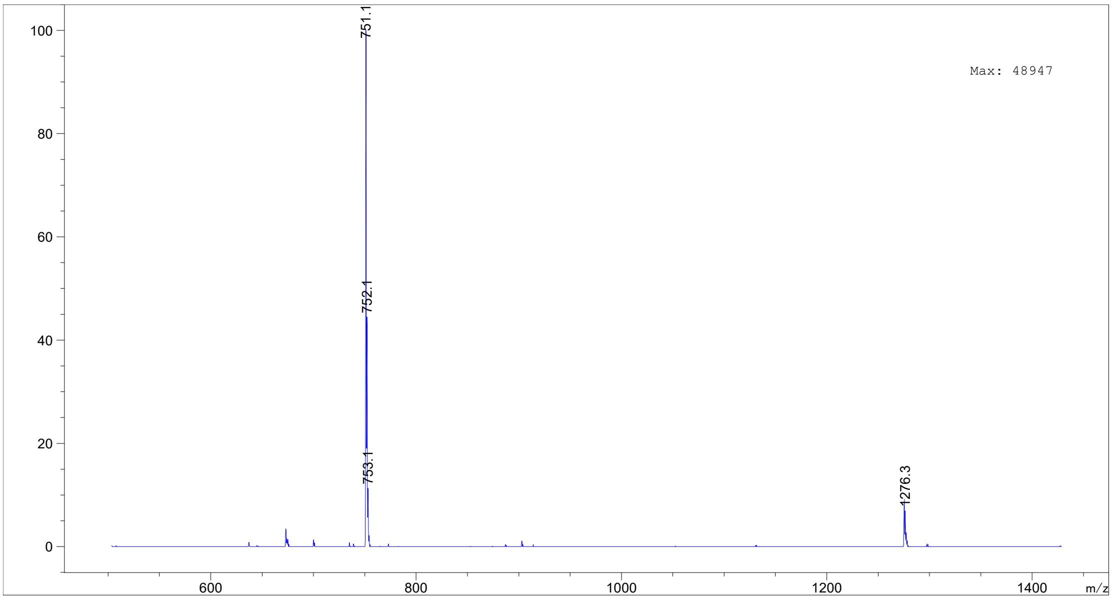

23. Compound **WYFF:** obsvd. [M+ TFA$^-$] = 774.1

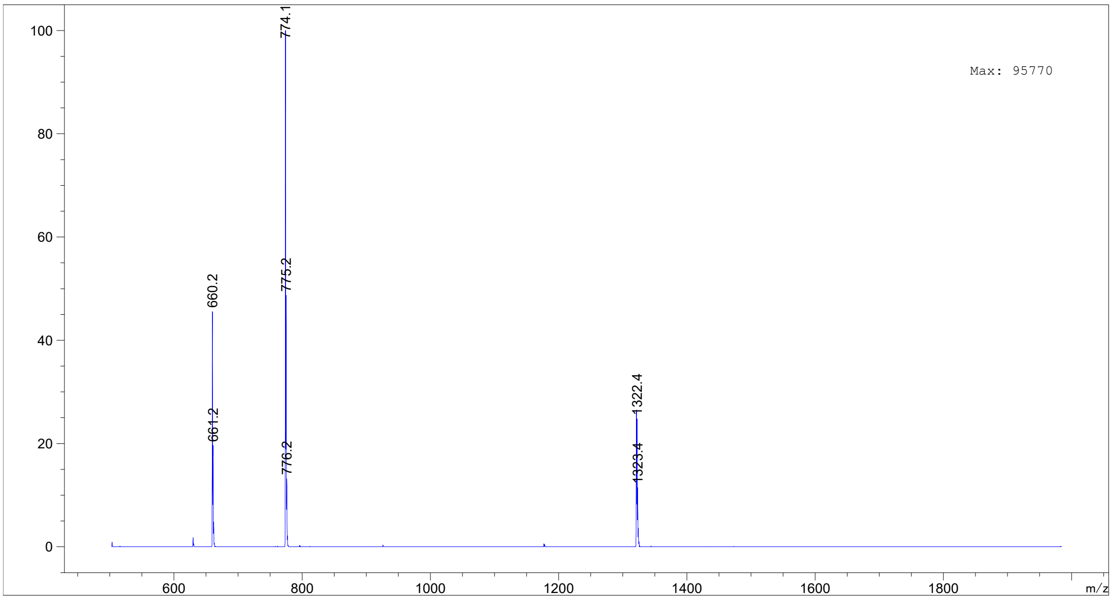

24. Compound **LVFF:** obsvd. [M+ TFA$^-$] = 637.2

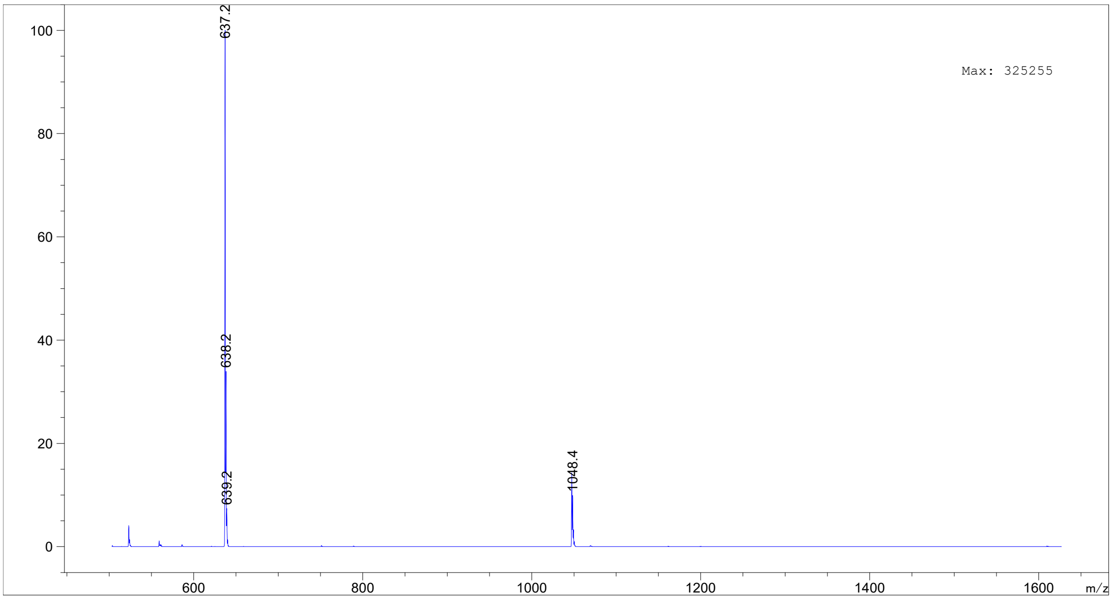

25. Compound **GFFY:** obsvd. [M+ TFA$^-$] = 645.1

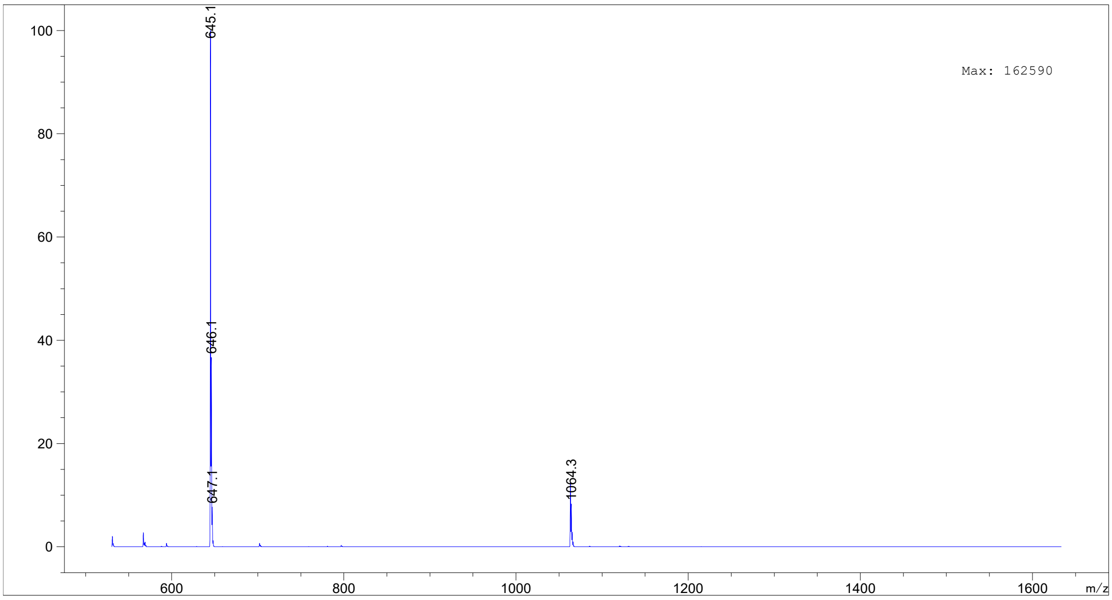

26. Compound **TAVF:** obsvd. [M+ H^+] = 437.1

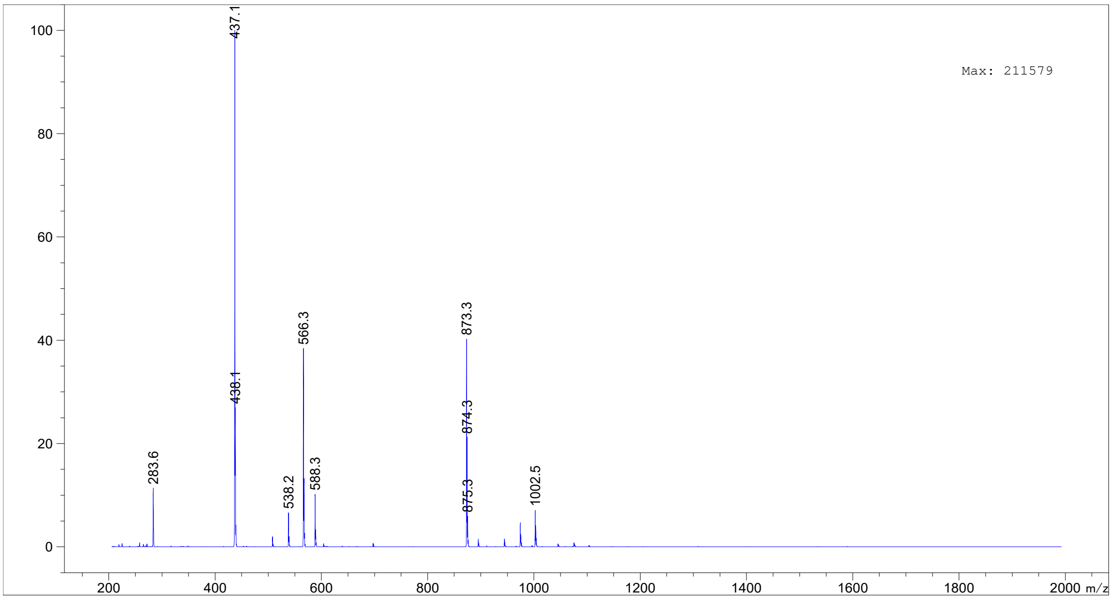

27. Compound **WFMM:** obsvd. [M+ H^+] = 527.2

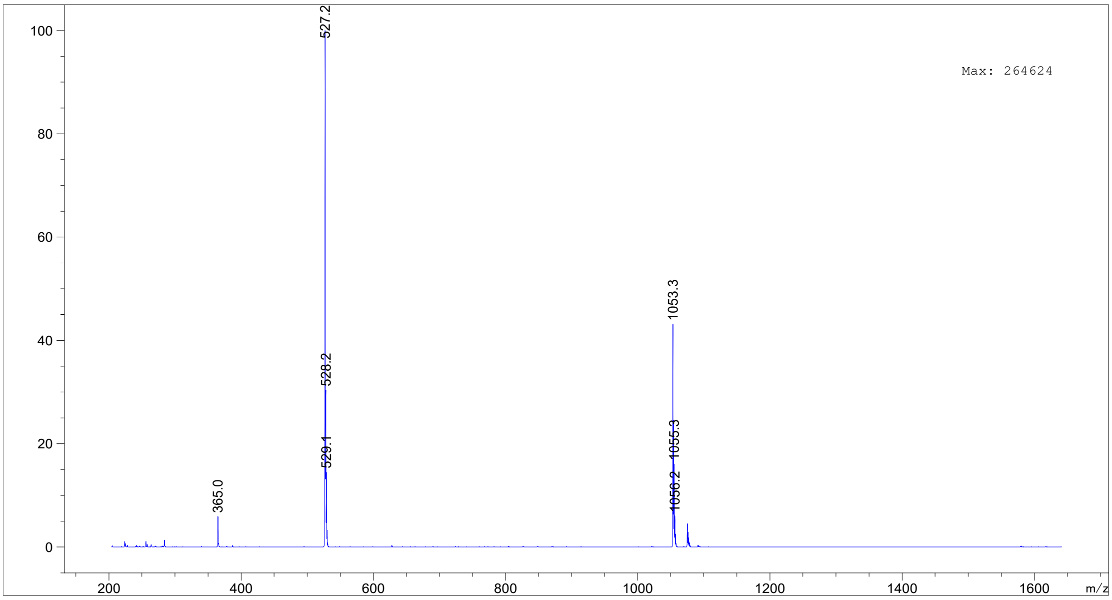

28. Compound **YVFA:** obsvd. [M+ H$^+$] = 499.2

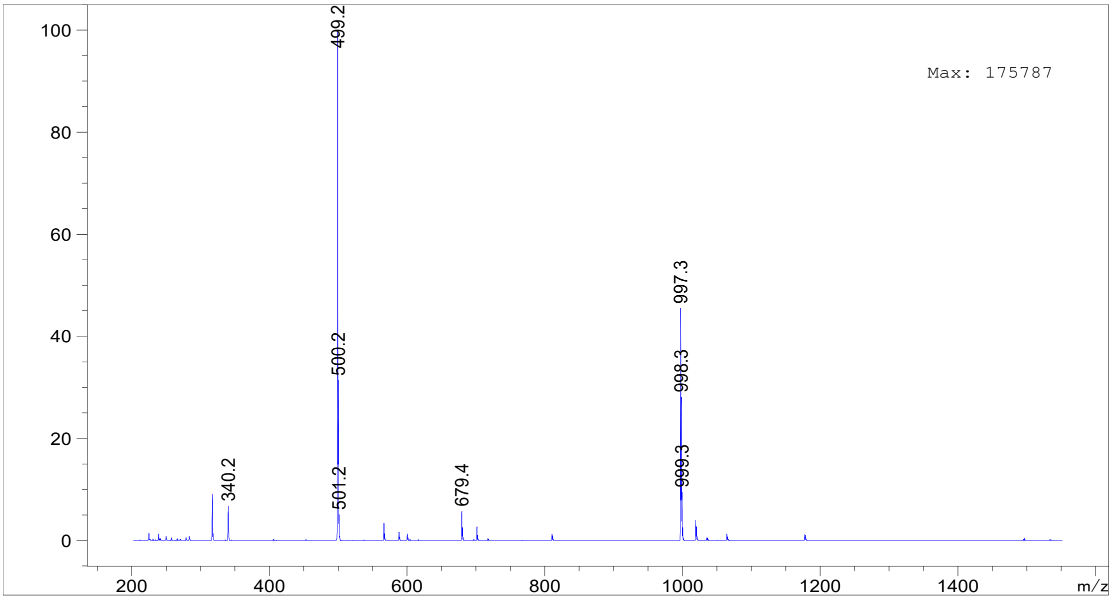

29. Compound **LVFG:** obsvd. [M+ H$^+$] = 435.2

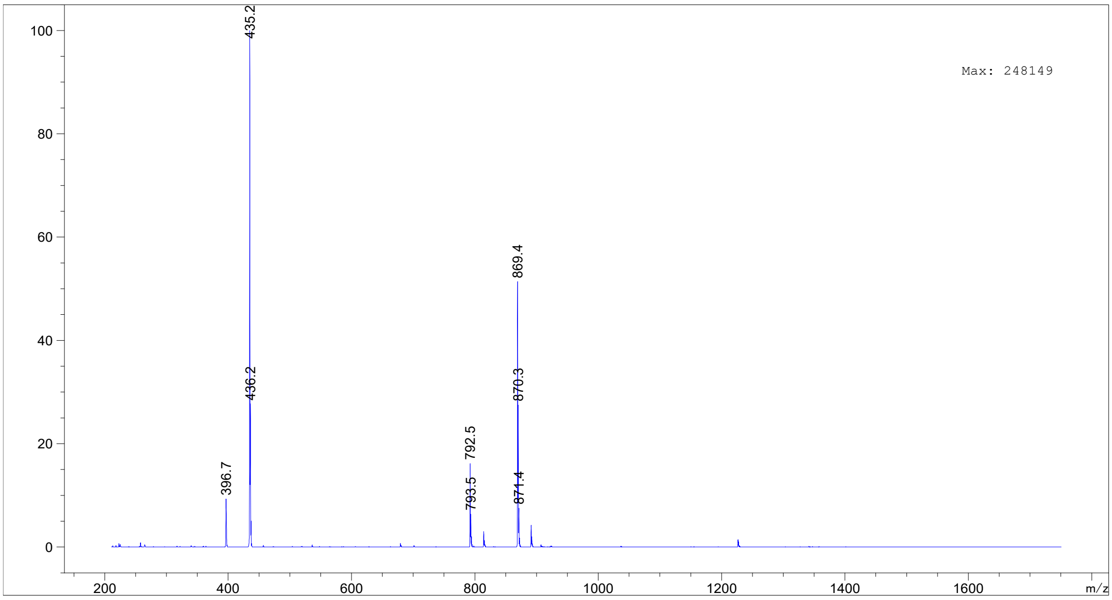

30. Compound **MVFP:** obsvd. [M+ H^+] = 493.2

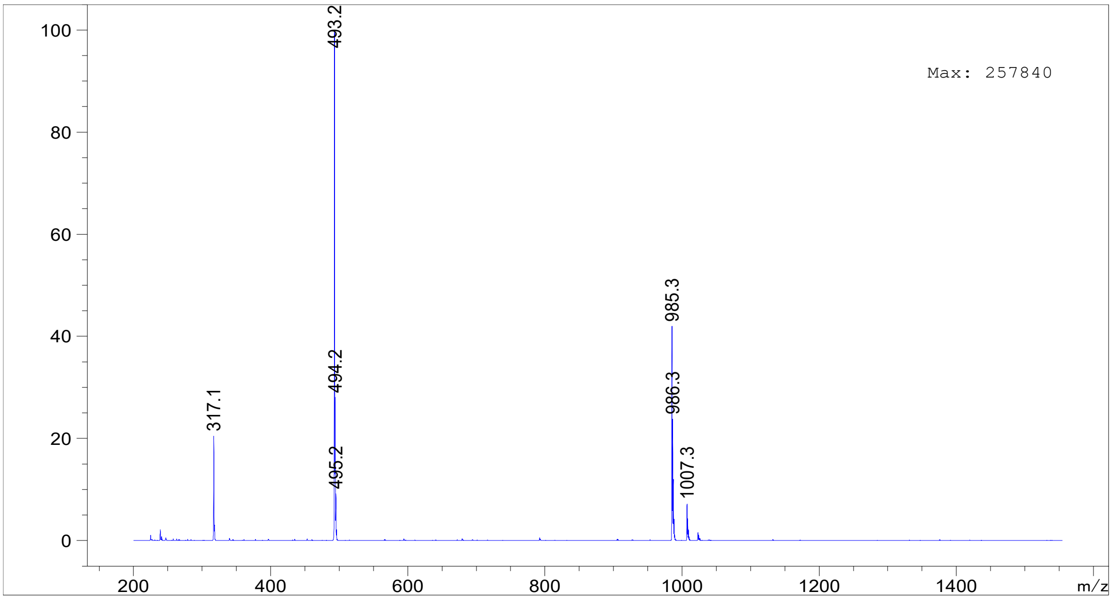

31. Compound **FMLF:** obsvd. [M+ H^+] = 557.2

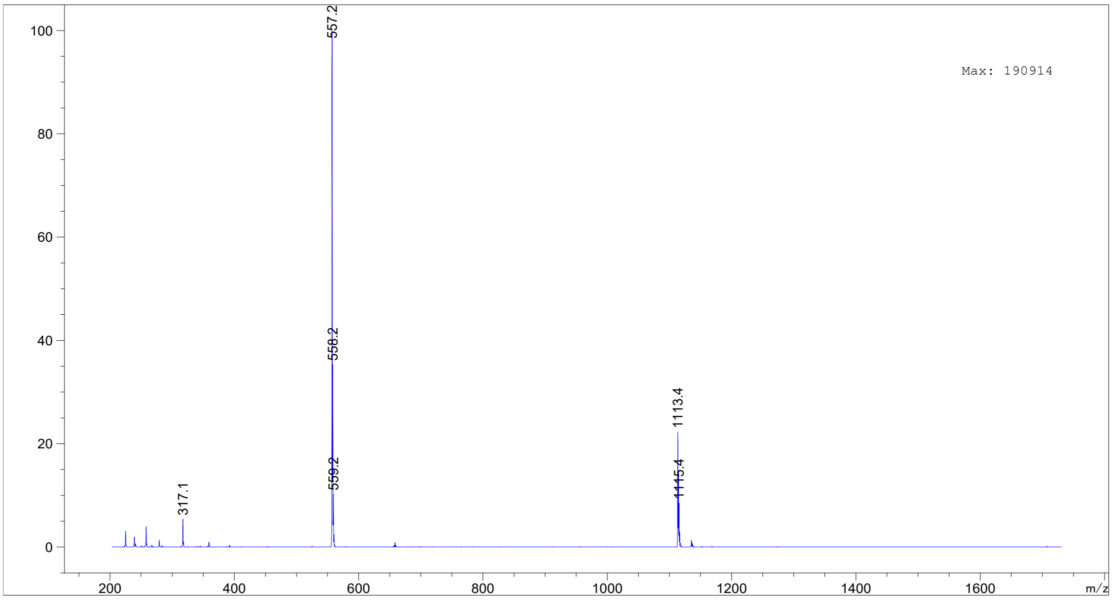

32. Compound **LLLS:** obsvd. [M+ H^+] = 445.2

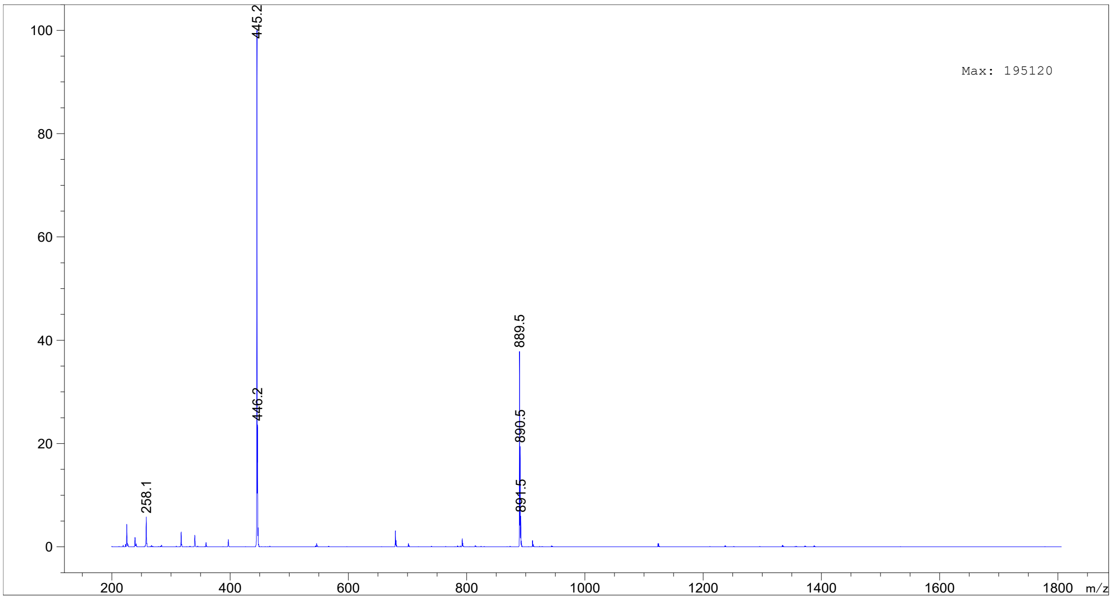

33. Compound **IFYT:** obsvd. [M+ H^+] = 543.2

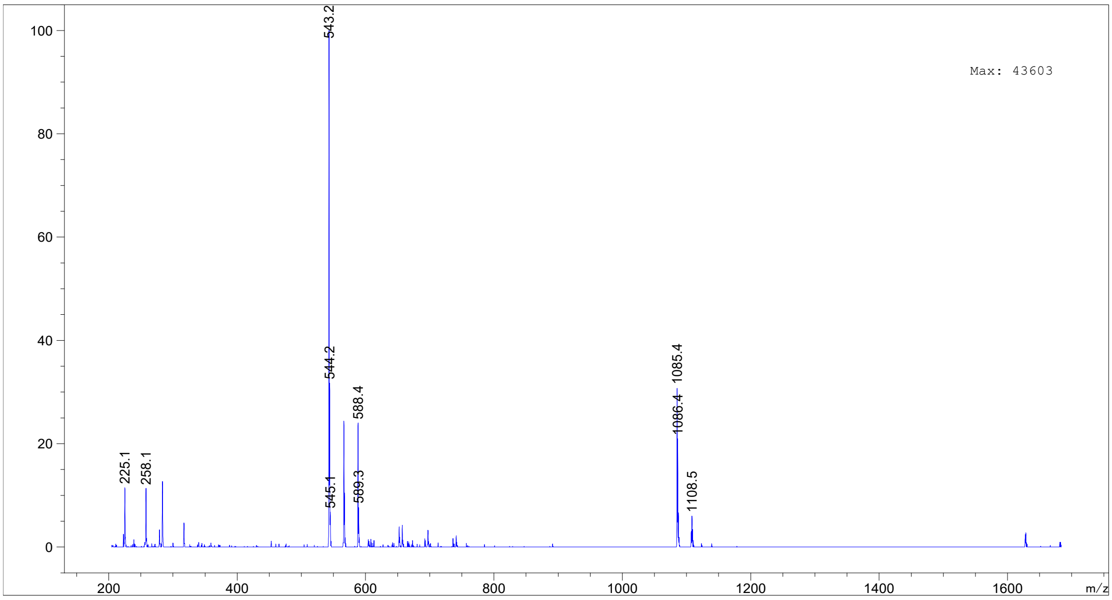

34. Compound **LLSL:** obsvd. $[M+ H^{+}] = 445.3$

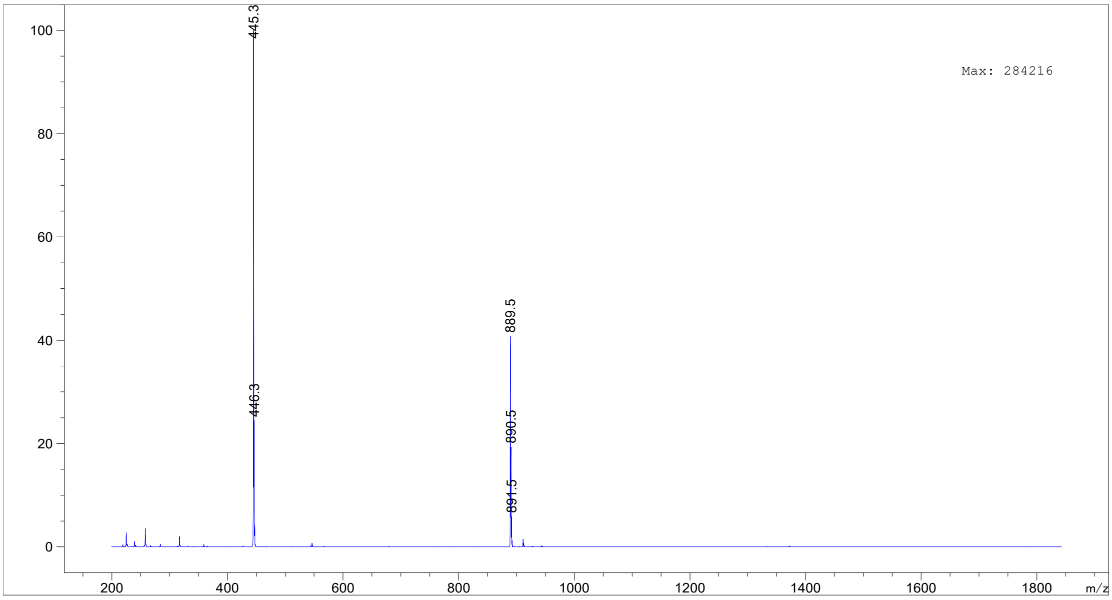

35. Compound **WLVW:** obsvd. $[M+ H^{+}] = 603.3$

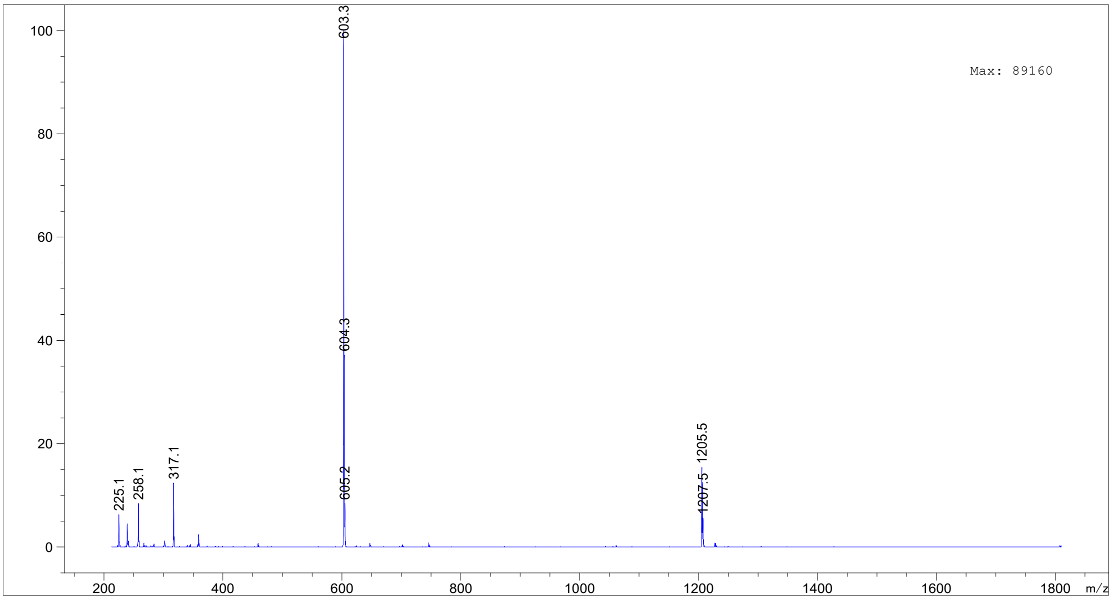

36. Compound **LWWF:** obsvd. $[M+ H^{+}] = 651.3$

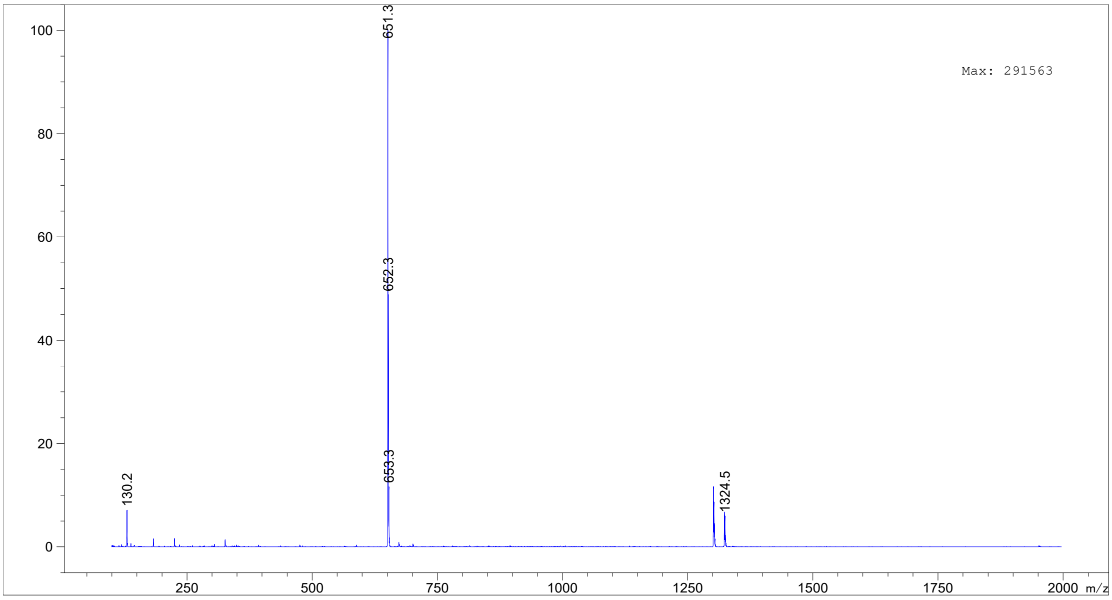

37. Compound **VFVF:** obsvd. $[M+TFA^{-}] = 622.5$

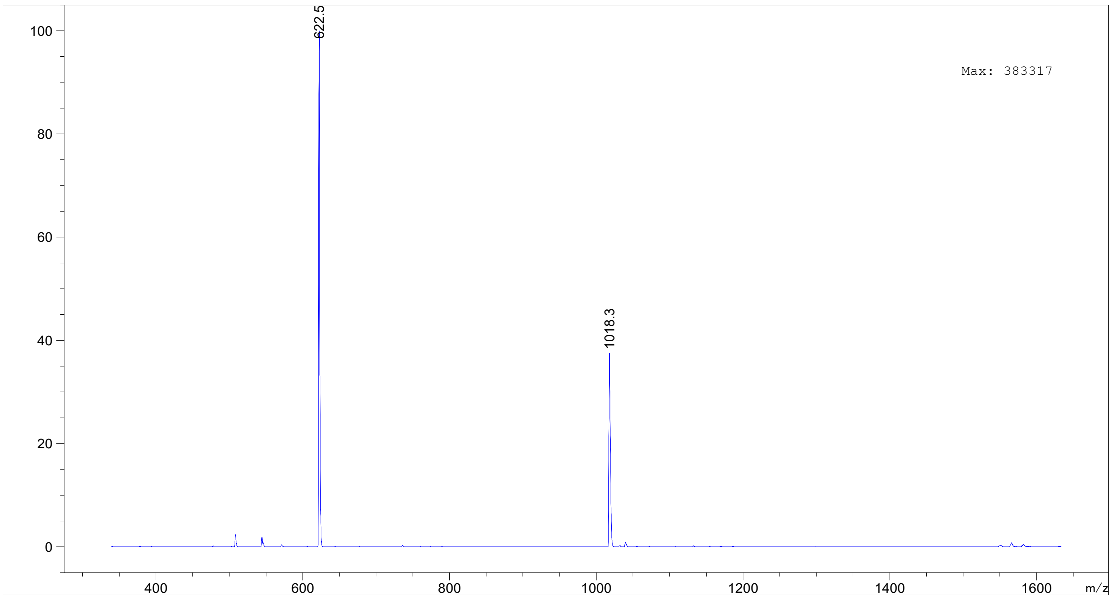

38. Compound **MMVV:** obsvd. [M+TFA$^-$] = 590.4

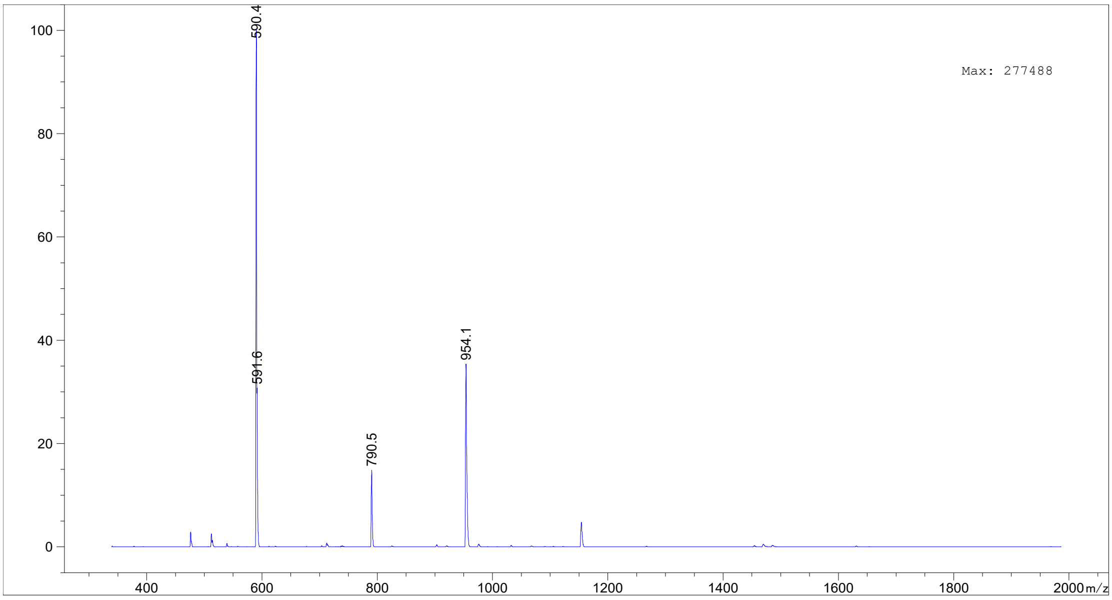

39. Compound **LVAI:** obsvd. [M+TFA$^-$] = 526.5

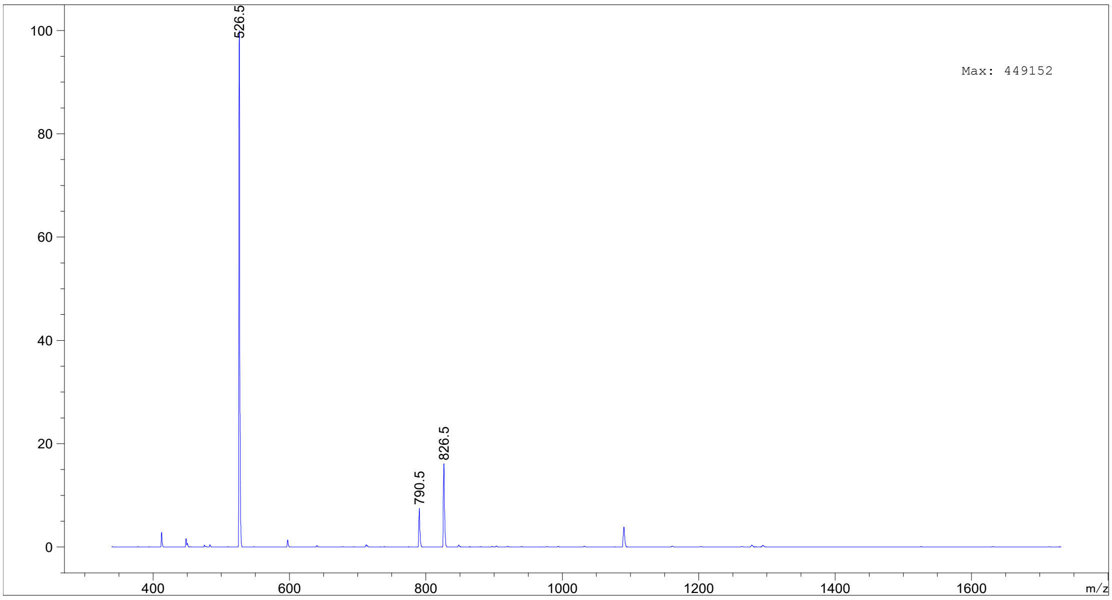

40. Compound **WAVV:** obsvd. [M+ H^{+}] = 474.5

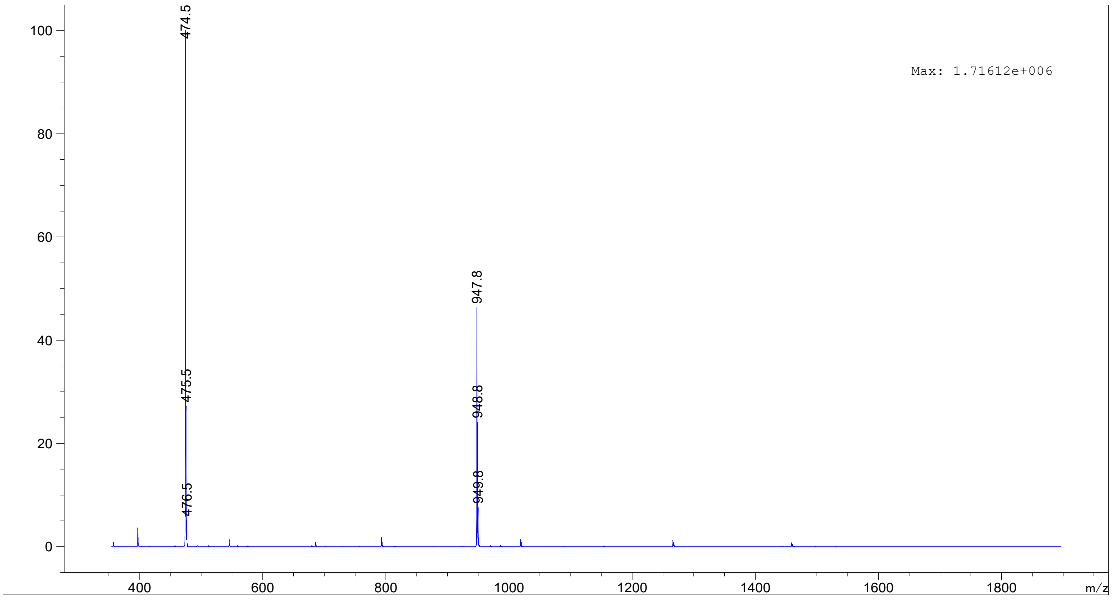

41. Compound **MMLL:** obsvd. [M+TFA^{-}] = 618.5

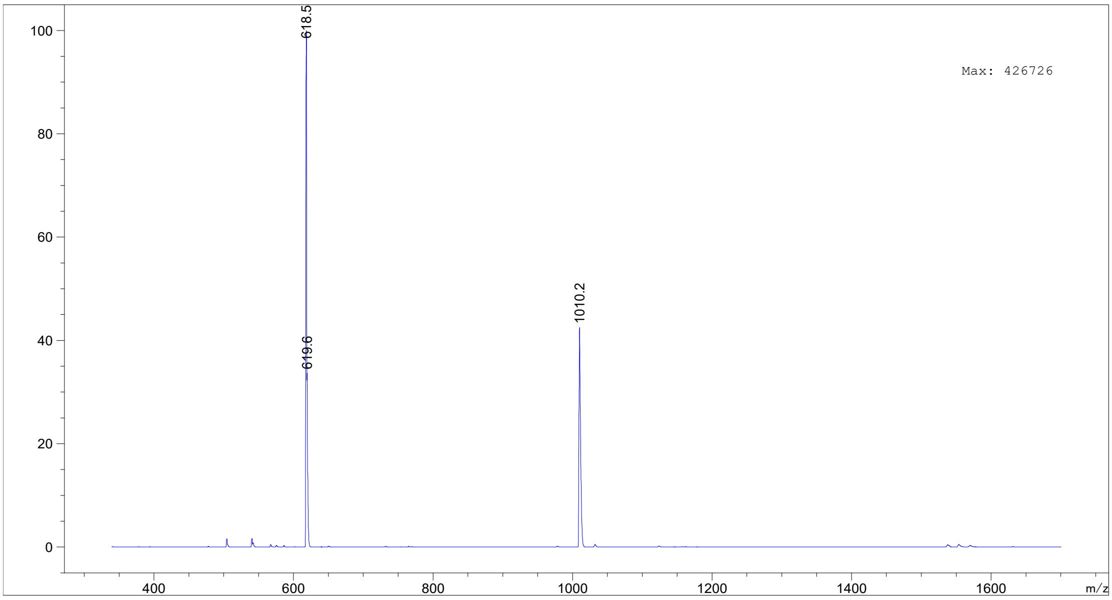

42. Compound **SWYF:** obsvd. [M+ H$^+$] = 602.7

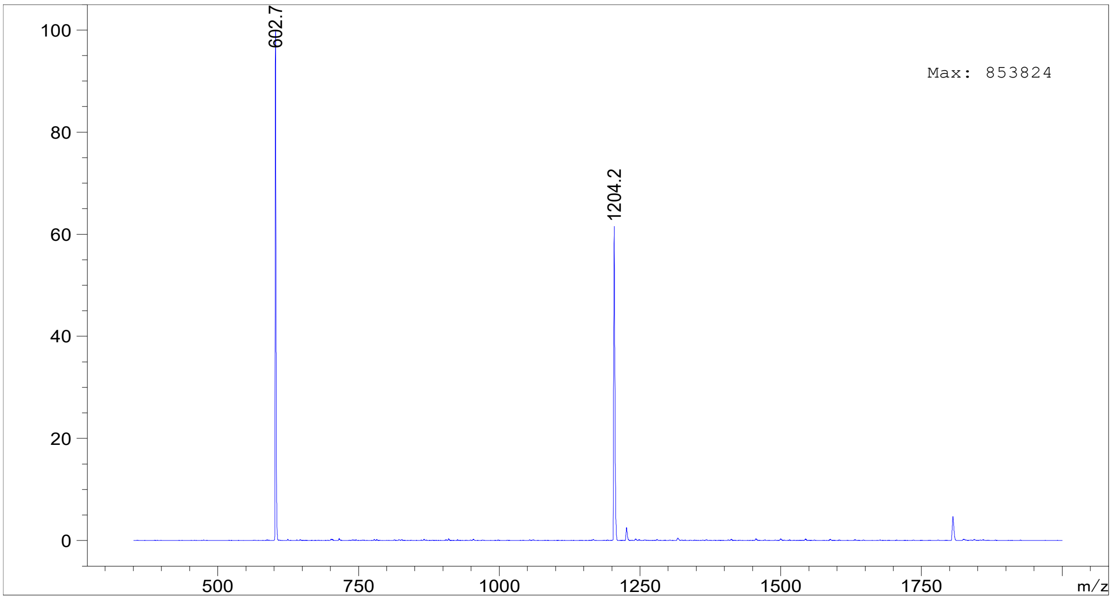

43. Compound **WWMF:** obsvd. [M+ H$^+$] = 668.9

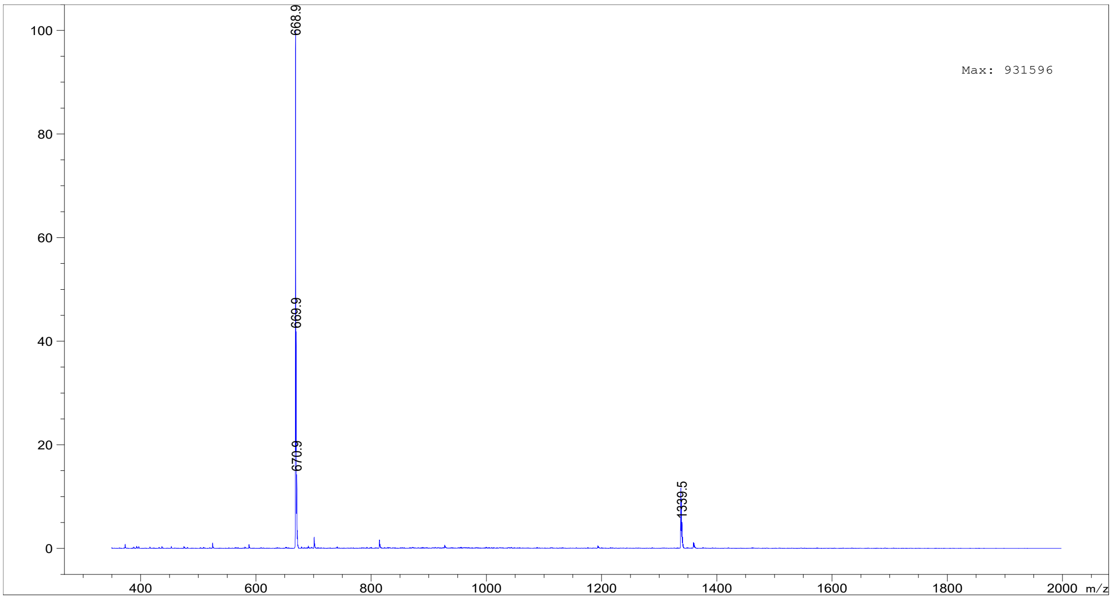

44. Compound **SYFF:** obsvd. [M+ H^+] = 563.5

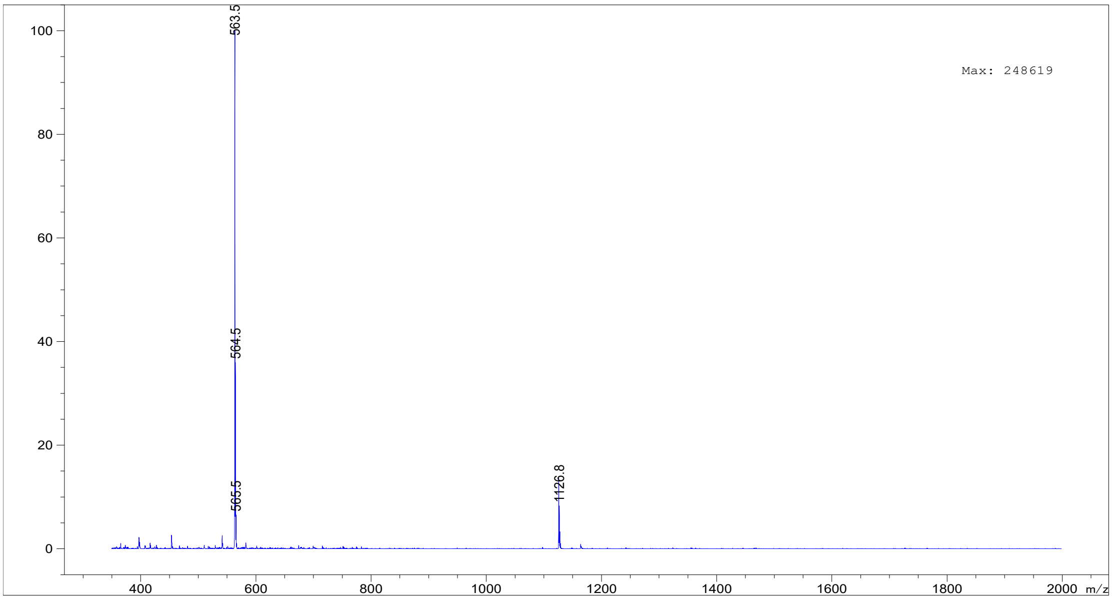

45. Compound **WFMM:** obsvd. [M+ H^+] = 613.8

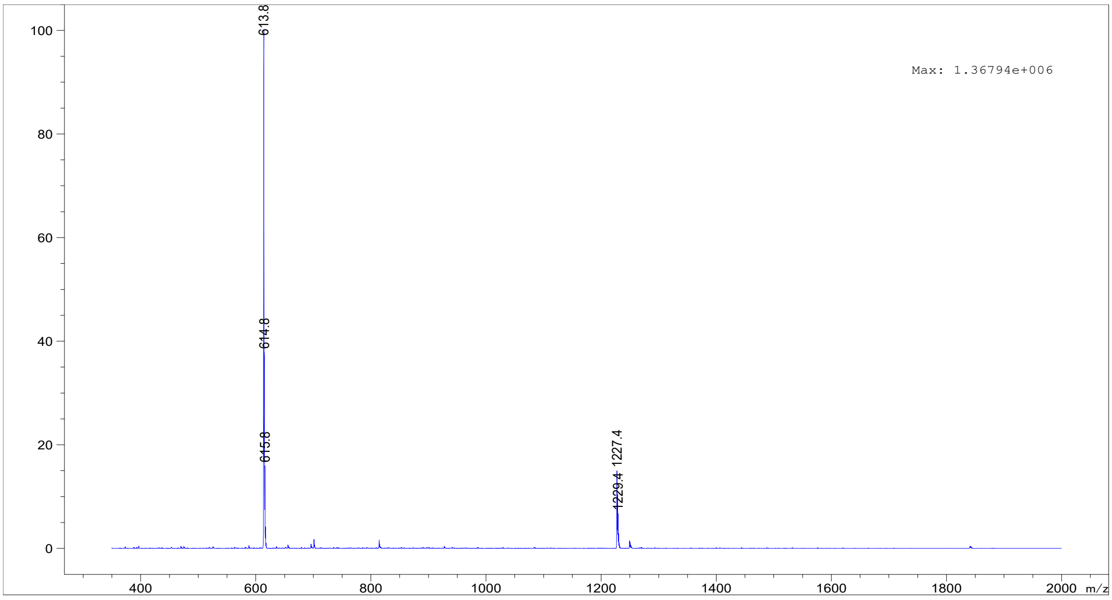

46. Compound **CWFS:** obsvd. [M+ H$^+$] = 541.9

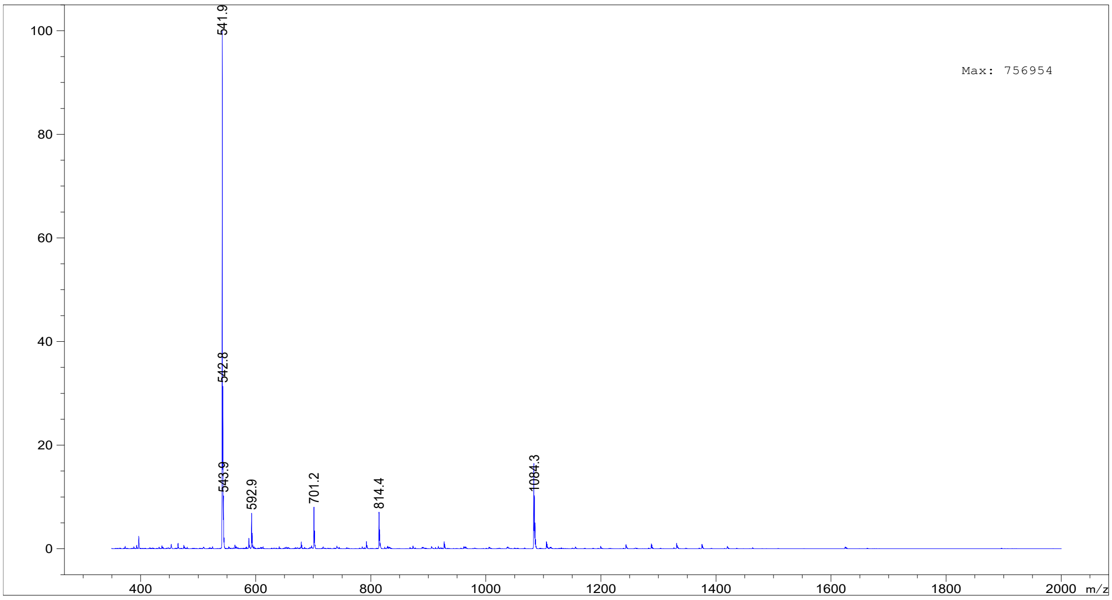

47. Compound **PWYW:** obsvd. [M+ H$^+$] = 651.4

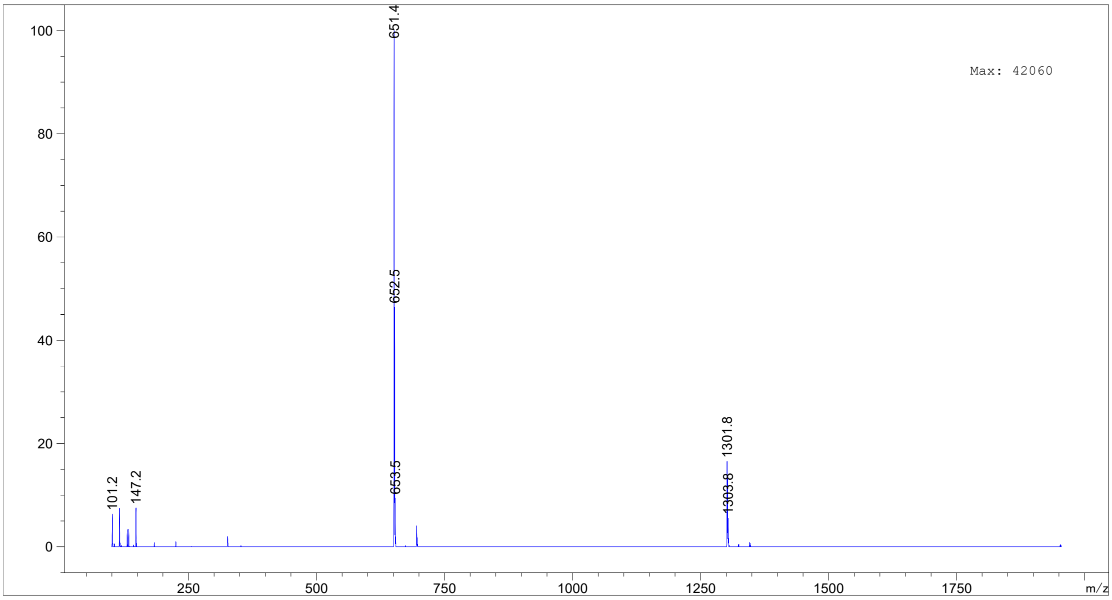

48. Compound **KVWL:** obsvd. [M+ H$^+$] = 545.0

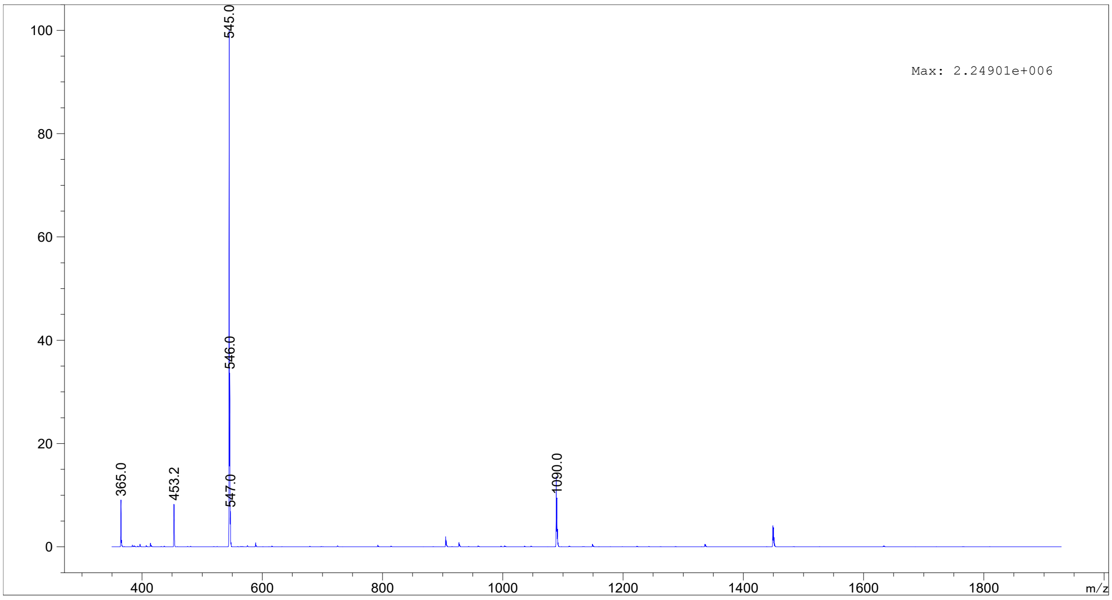

49. Compound **FYAY:** obsvd. [M+ H$^+$] = 562.9

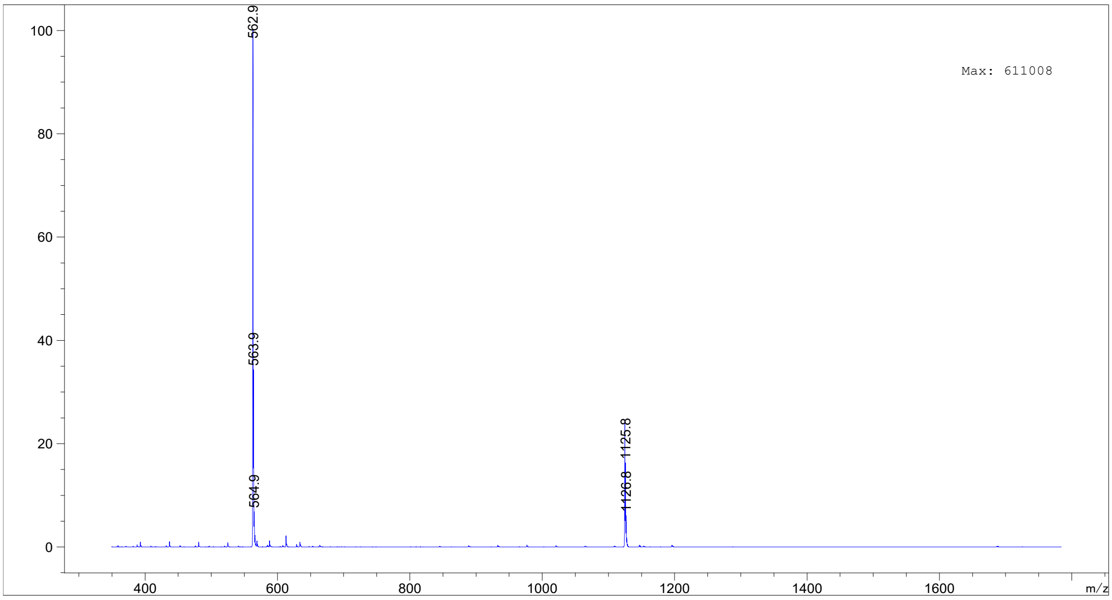

50. Compound **KLLF:** obsvd. [M+ H^+] = 520.0

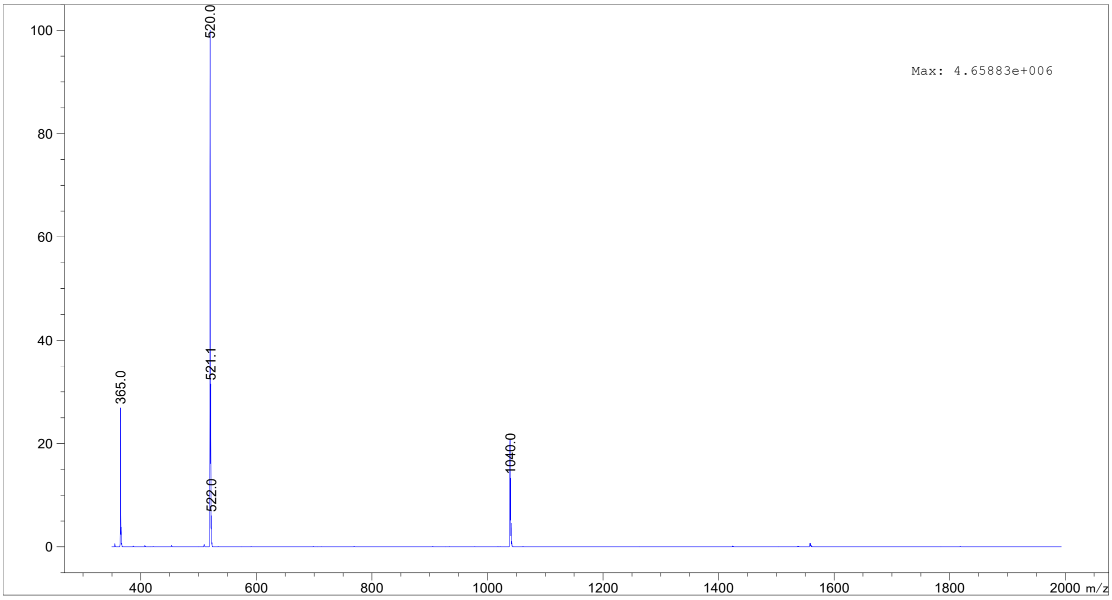

51. Compound **SAWF:** obsvd. [M+ H^+] = 509.9

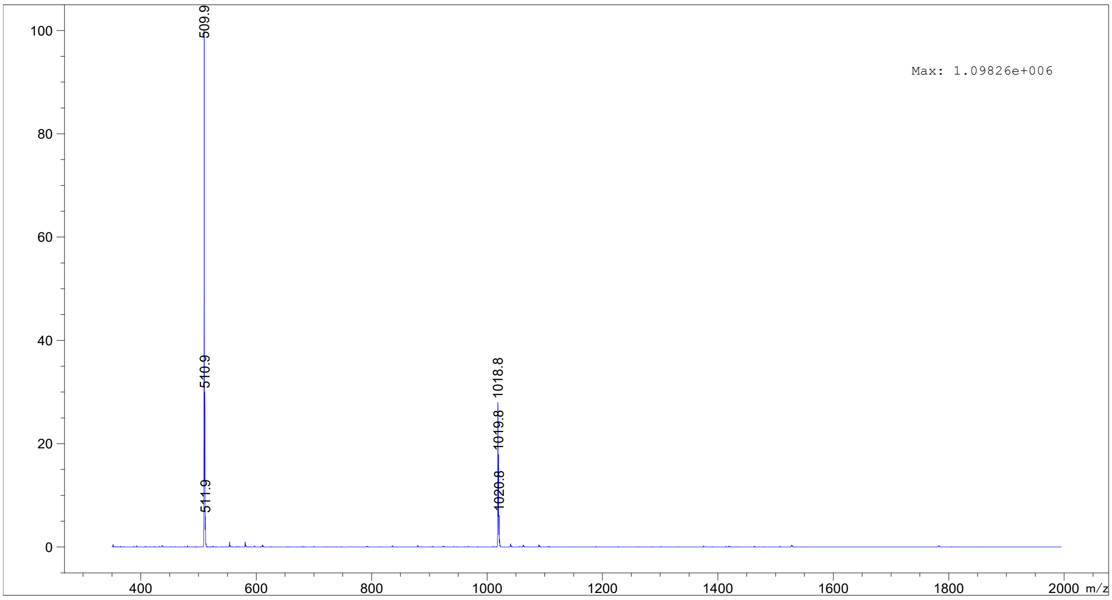

52. Compound **TGFF:** obsvd. $[M+ H^{+}] = 471.0$

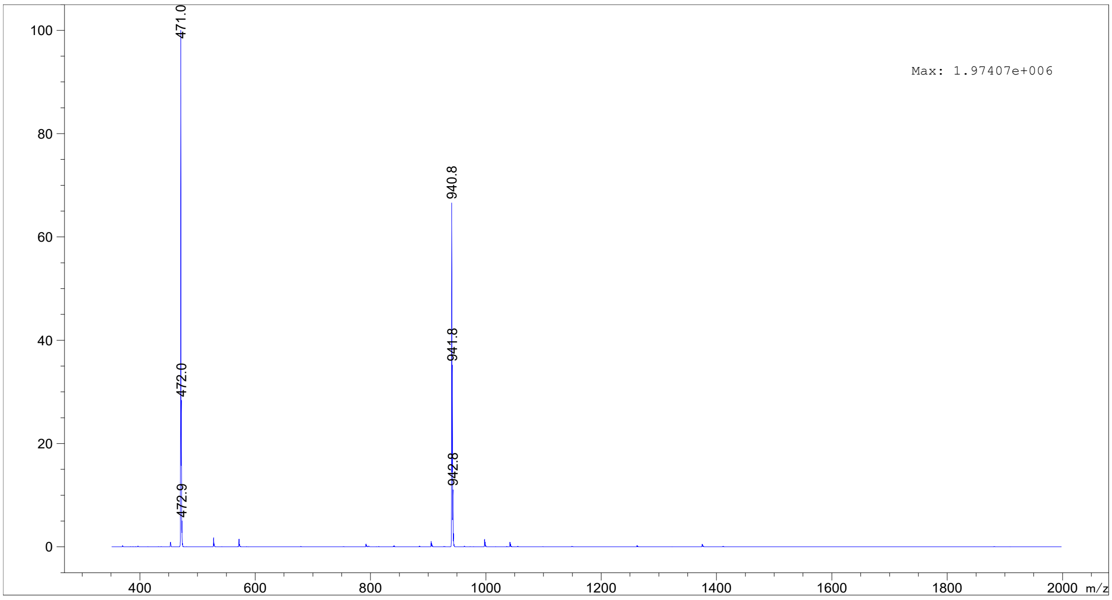

53. Compound **VAFY:** obsvd. $[M+ H^{+}] = 499.0$

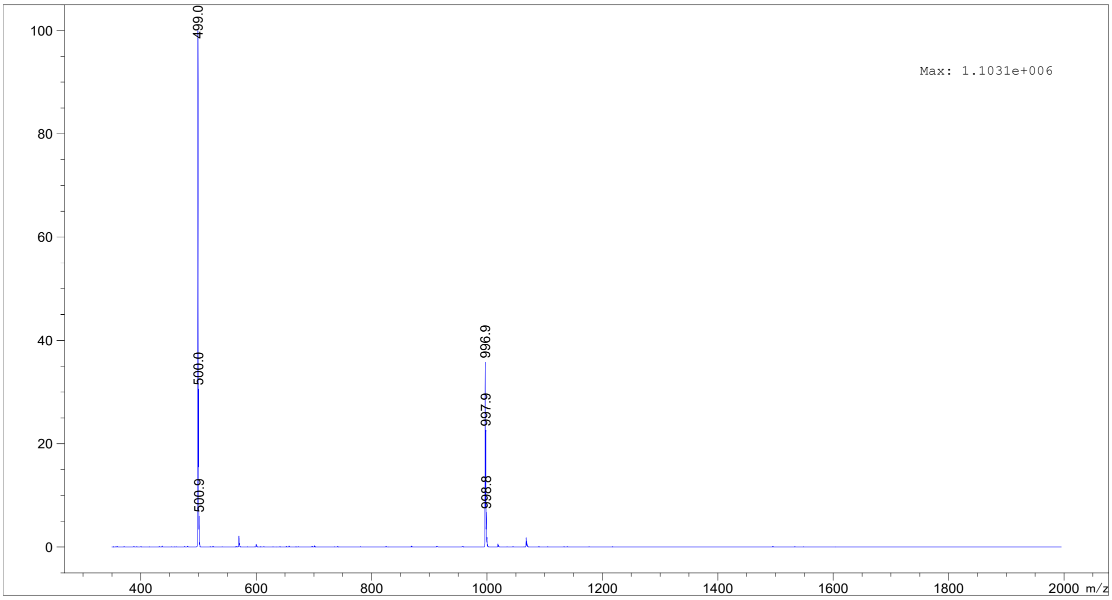

54. Compound **SSFF:** obsvd. [M+ H$^+$] = 487.0

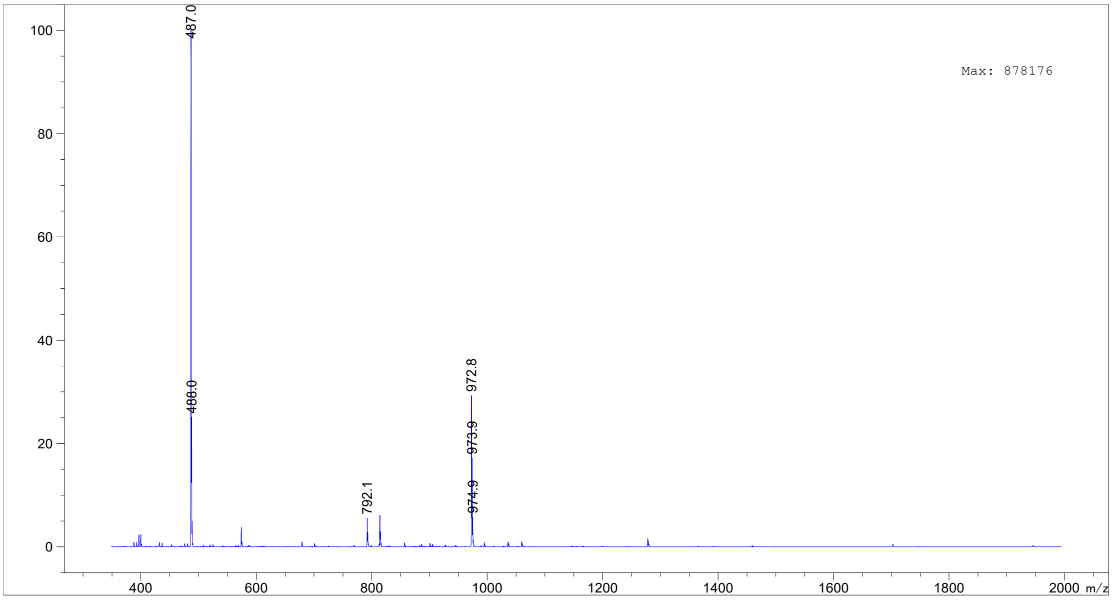

55. Compound **CTYF:** obsvd. [M+ H$^+$] = 532.9

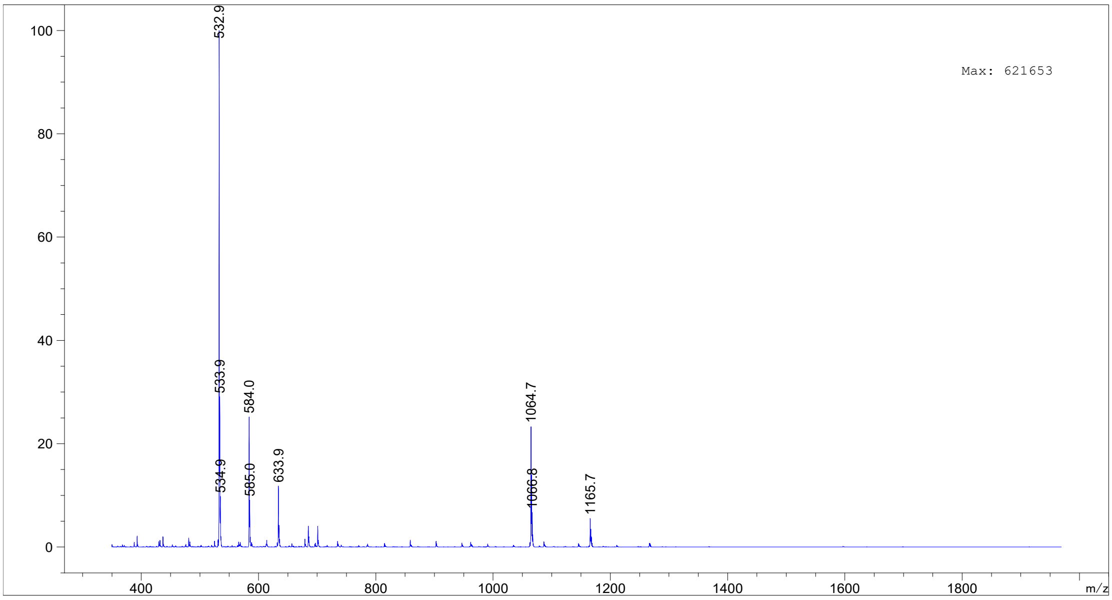

56. Compound **FSYV:** obsvd. [M+ H$^+$] = 515.0

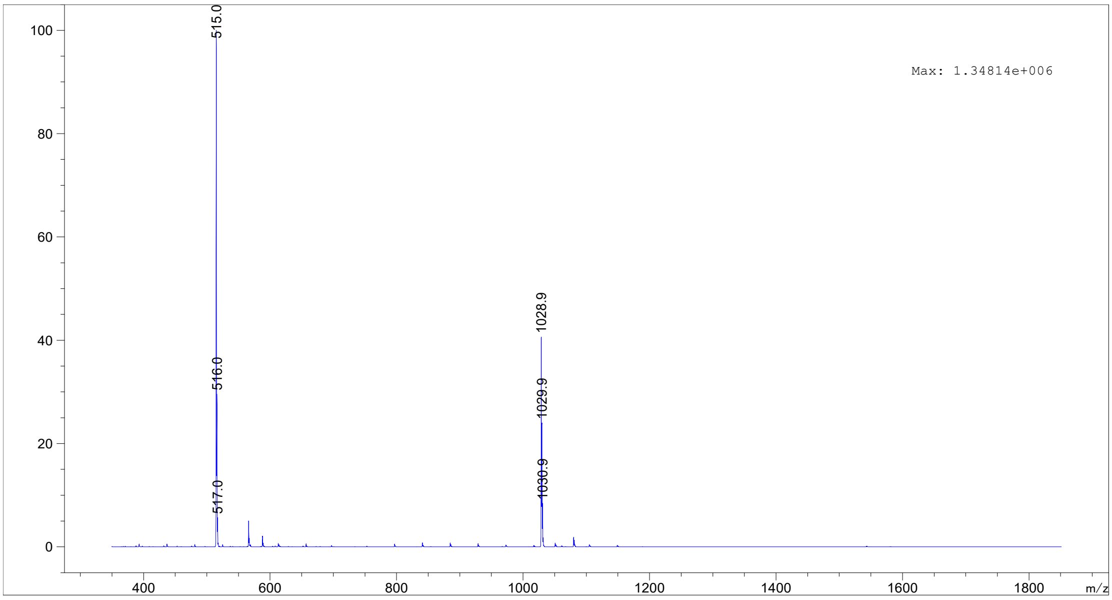

57. Compound **GVLF:** obsvd. [M+ H$^+$] = 435.1

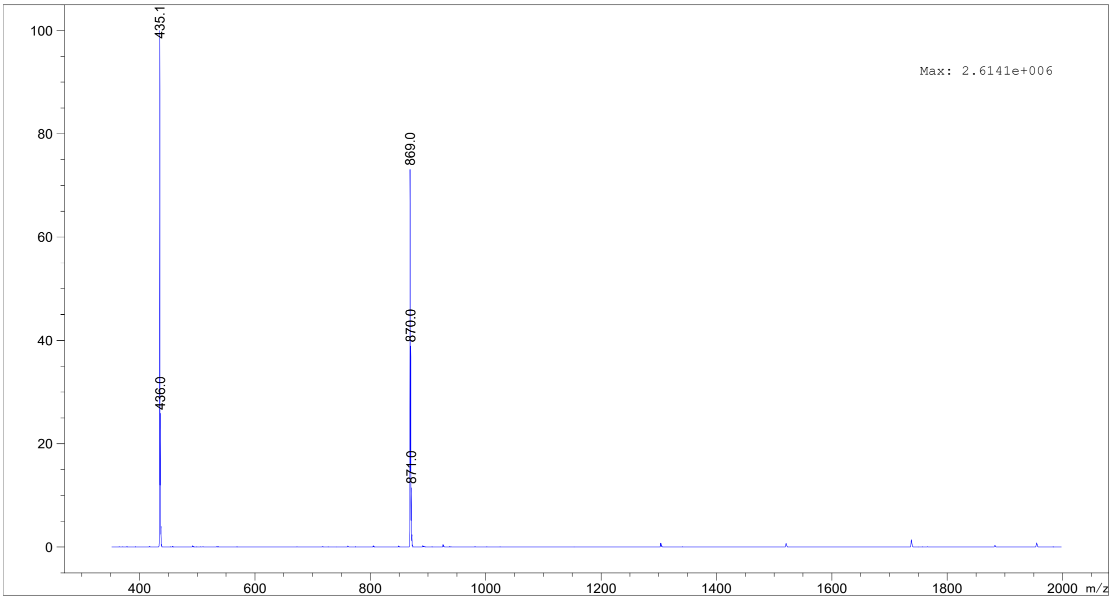

58. Compound **STYF:** obsvd. [M+ H$^+$] = 517.0

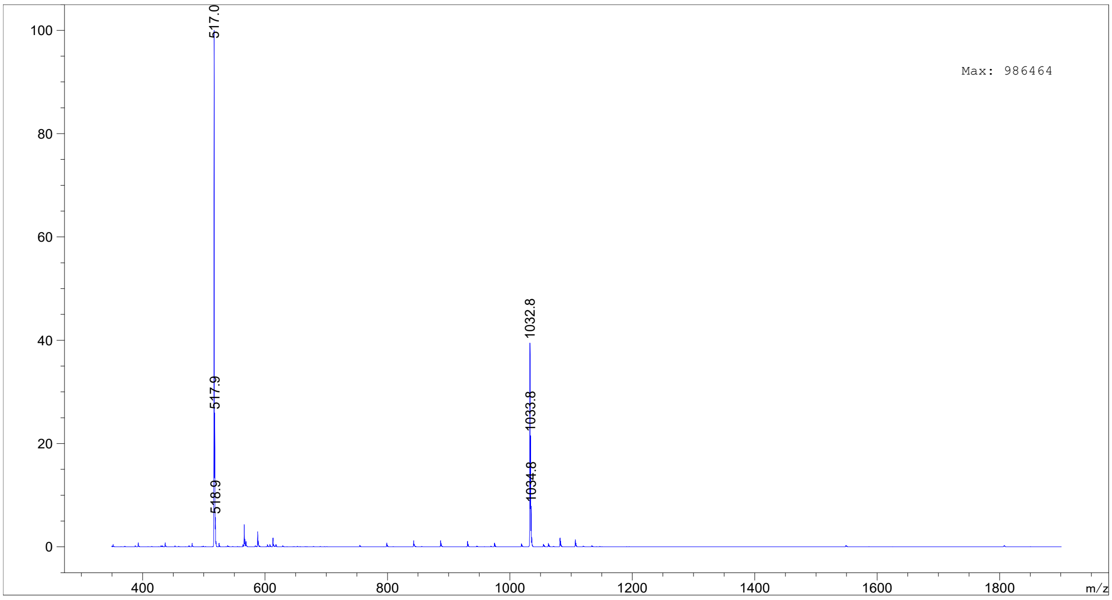

59. Compound **ILVV:** obsvd. [M+ H$^+$] = 442.6

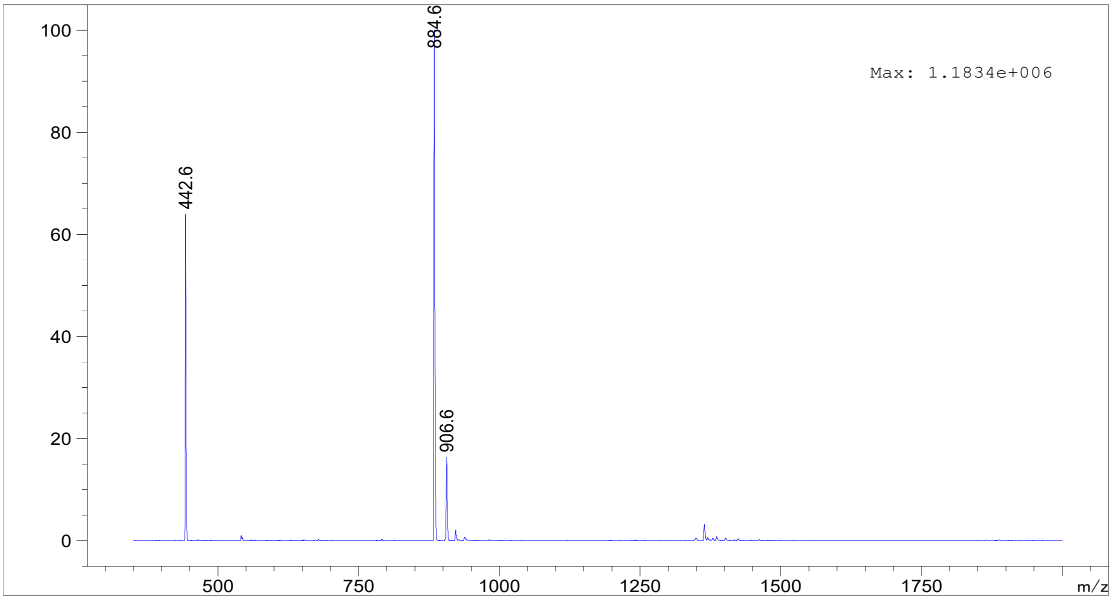

60. Compound **IVII:** obsvd. [M+ H$^+$] = 456.6

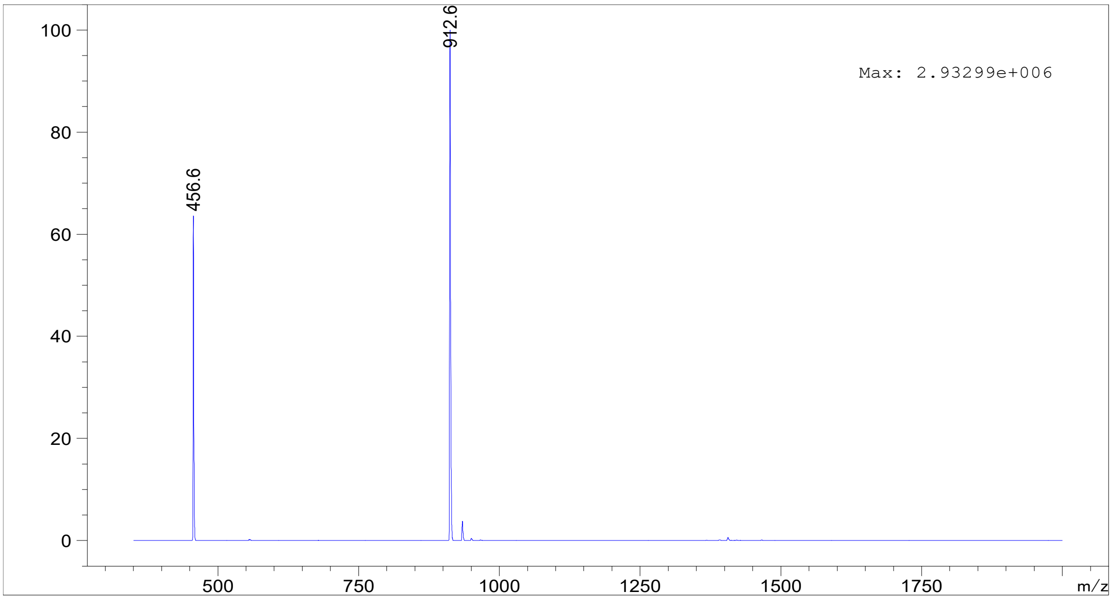

61. Compound **LVII:** obsvd. [M+ H$^+$] = 456.6

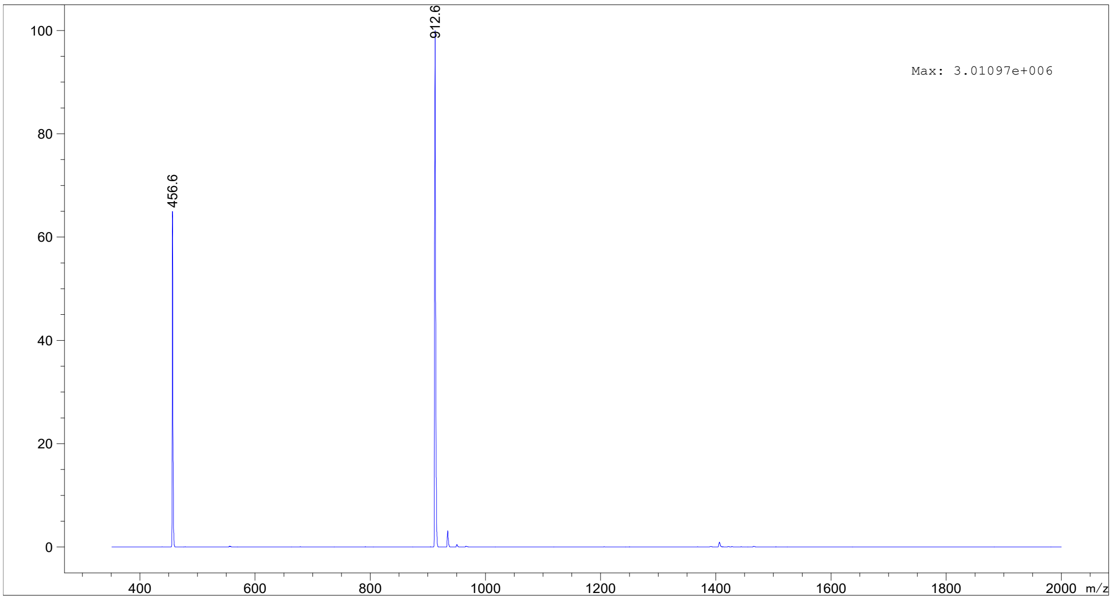

62. Compound **LVLI:** obsvd. [M+ H$^+$] = 456.6

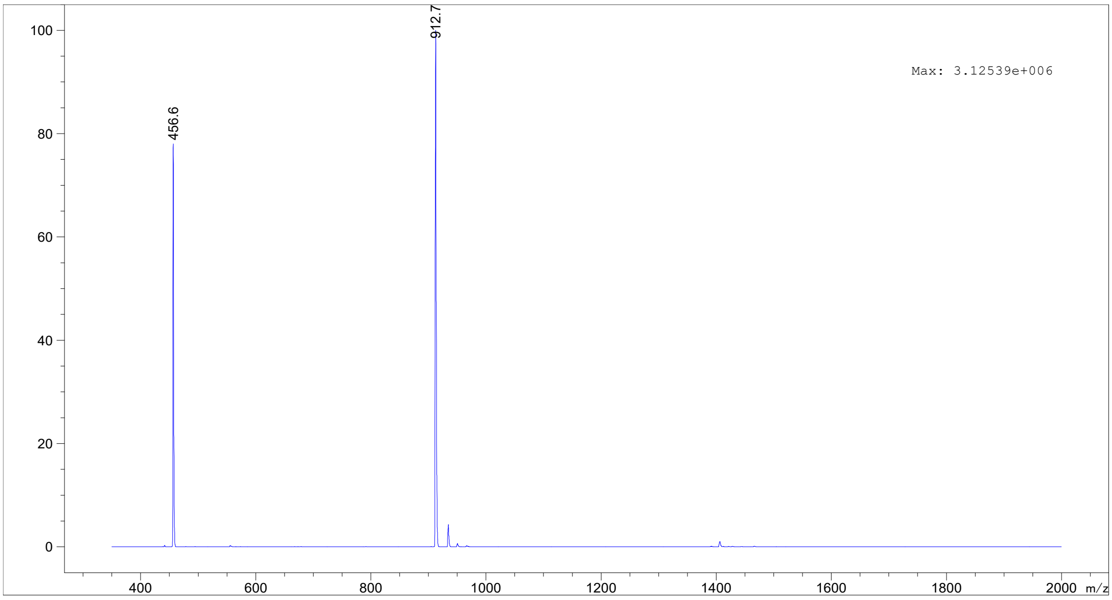

63. Compound **SLVV:** obsvd. [M+ H$^+$] = 416.5

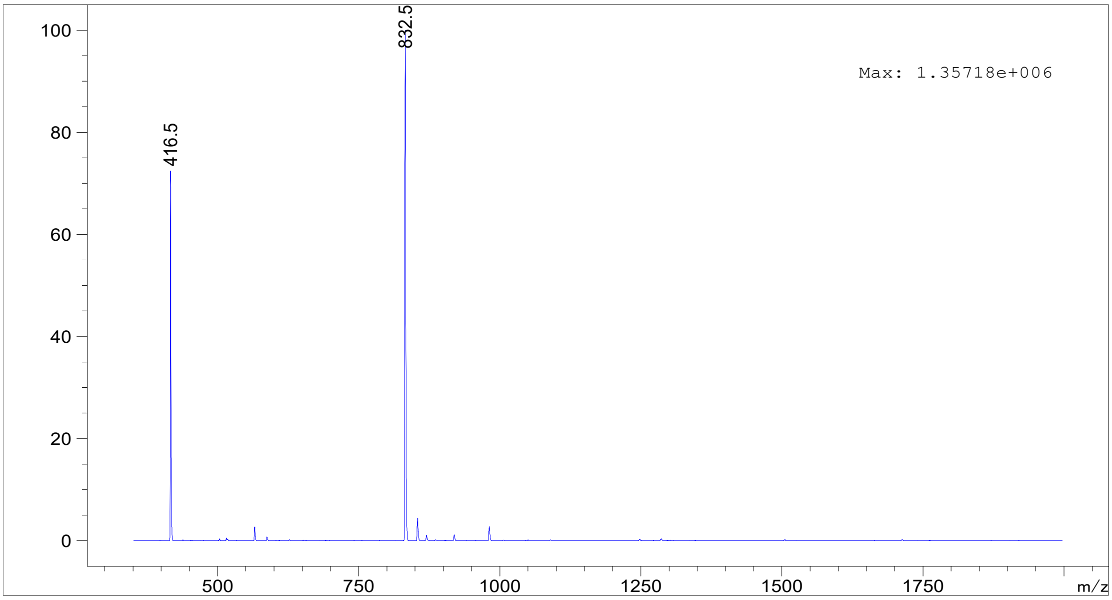

64. Compound **VILI:** obsvd. $[M+ H^{+}] = 456.5$

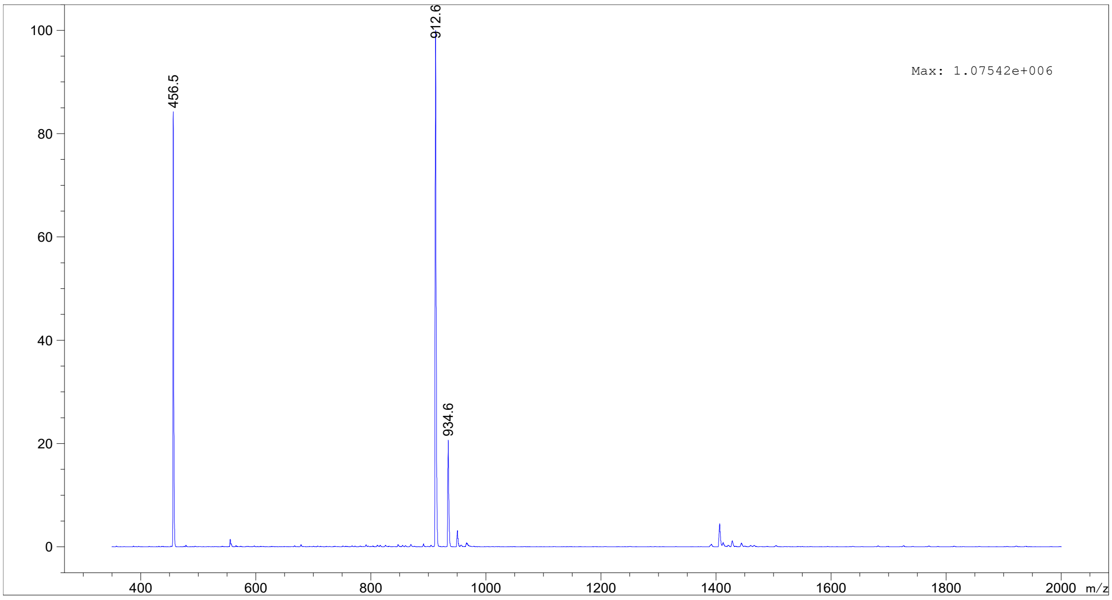

65. Compound **VLVL:** obsvd. $[M+ H^{+}] = 442.5$

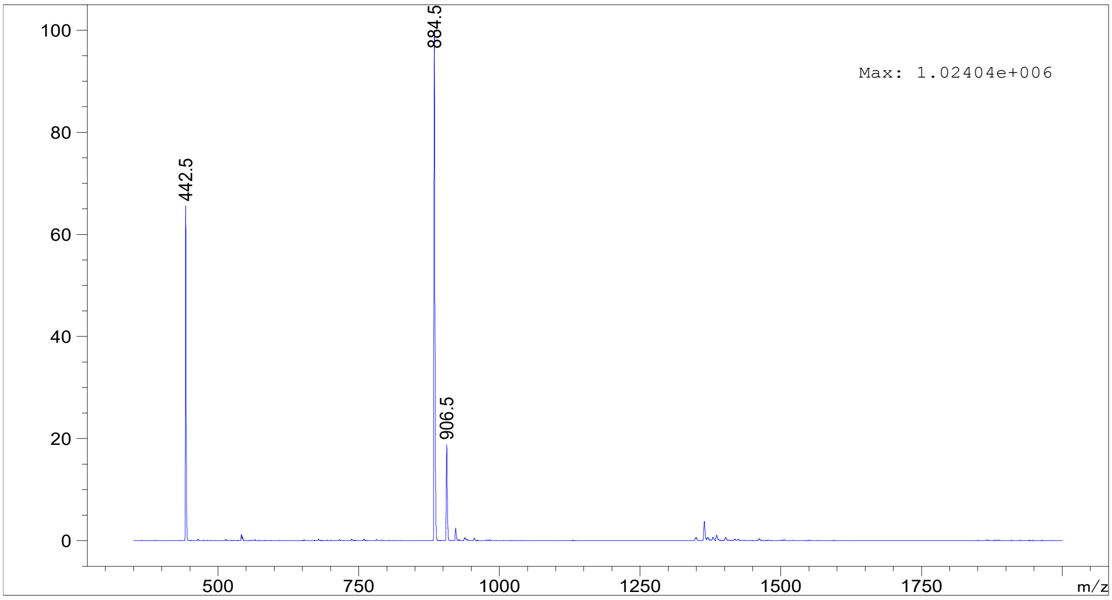

66. Compound **GVFY:** obsvd. [M+ H$^+$] = 485.4

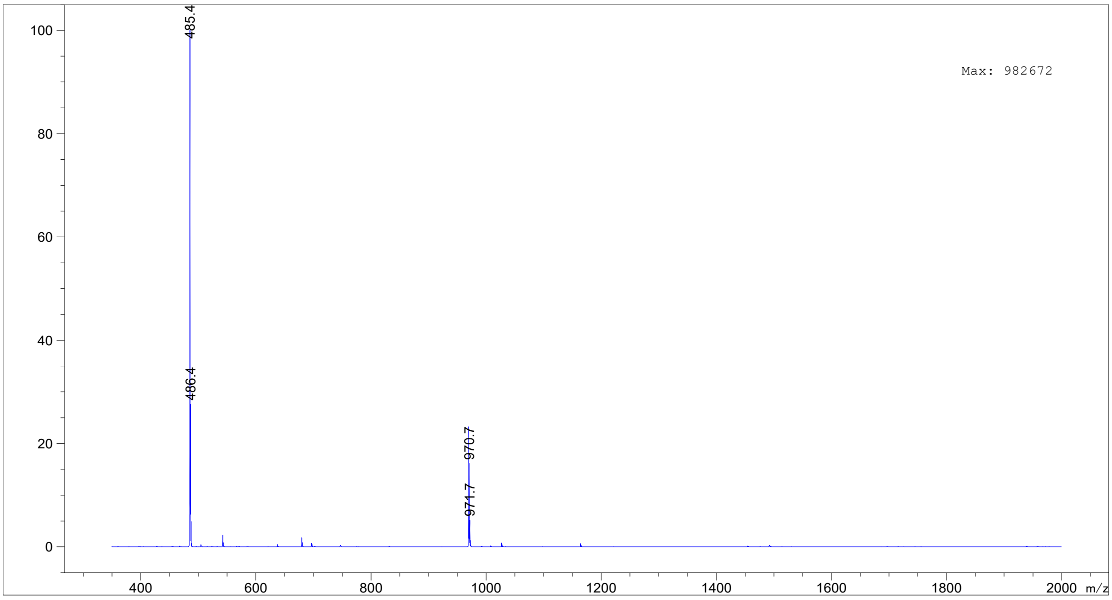

67. Compound **IMVV:** obsvd. [M+ H$^+$] = 461.1

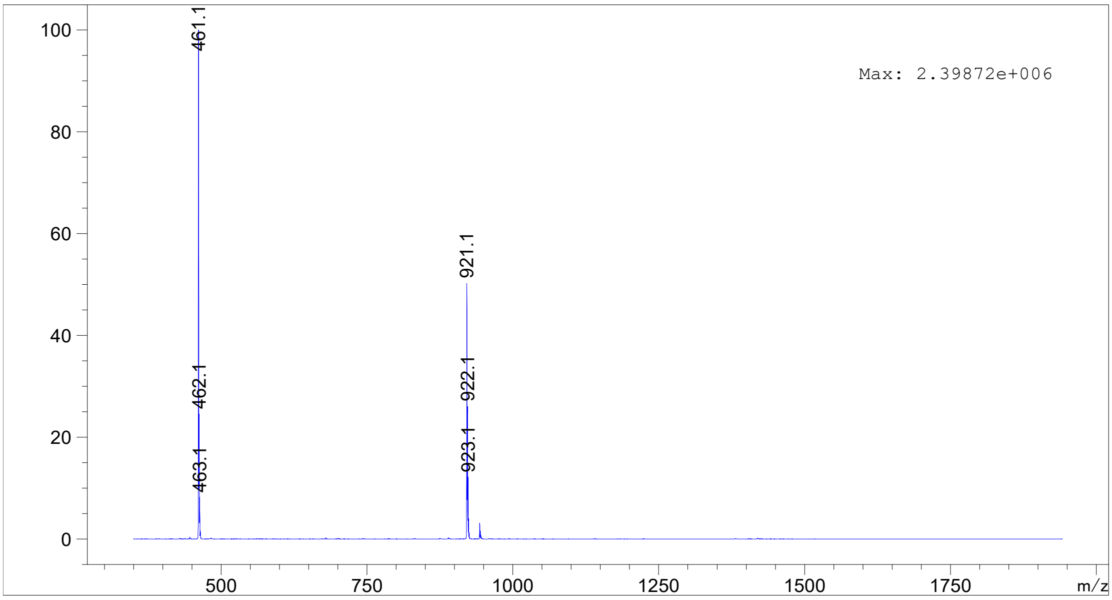

68. Compound **LTLF:** obsvd. [M+ H^{+}] = 493.3

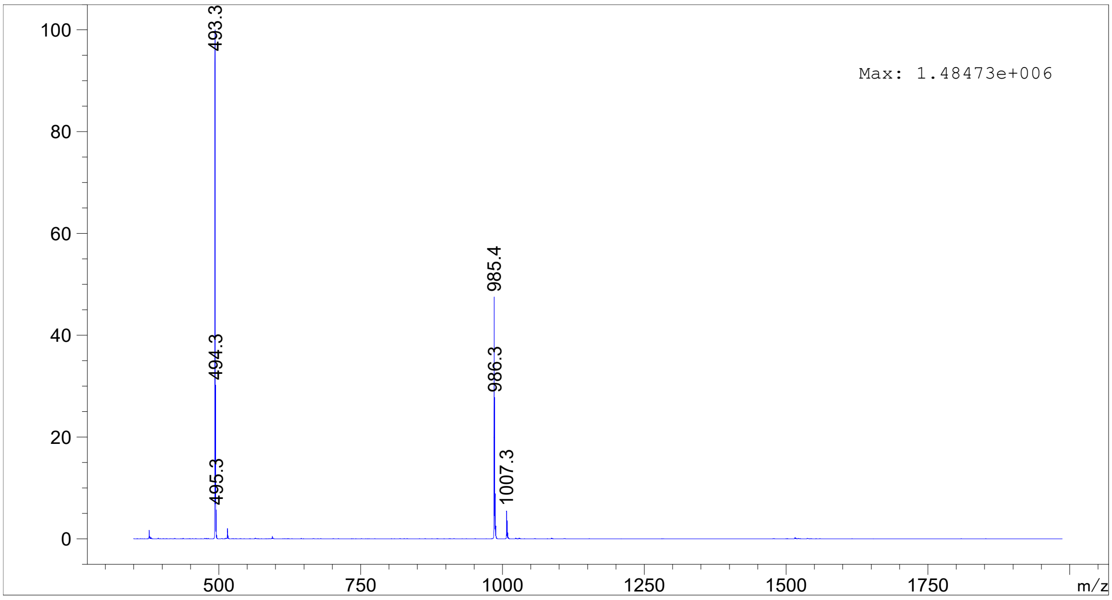

69. Compound **LTLI:** obsvd. [M+ H^{+}] = 459.3

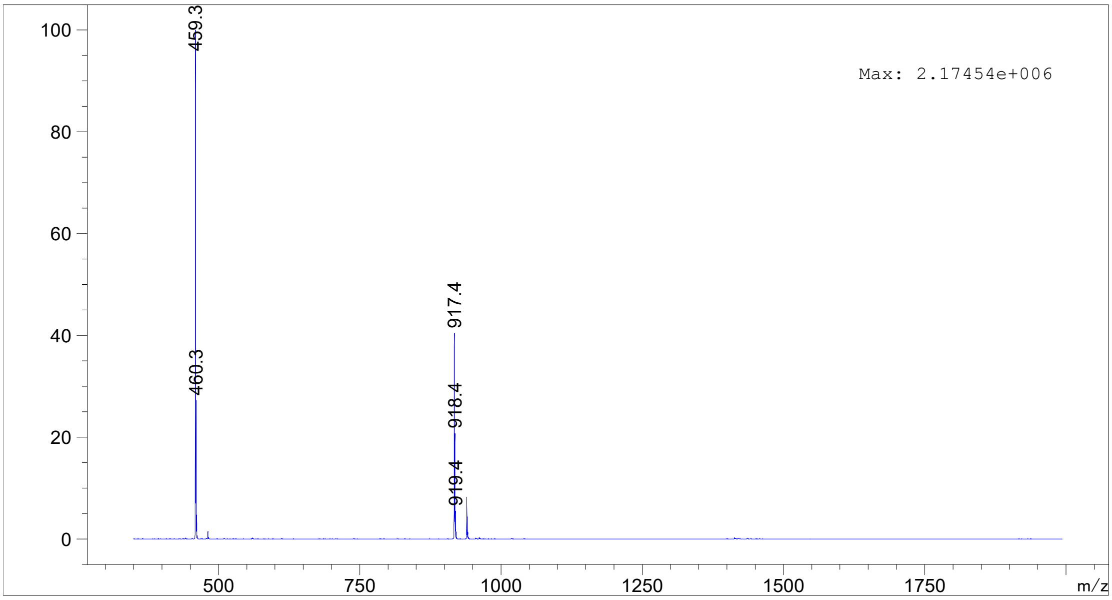

70. Compound **LWFF:** obsvd. $[M+H^{+}] = 611.9$

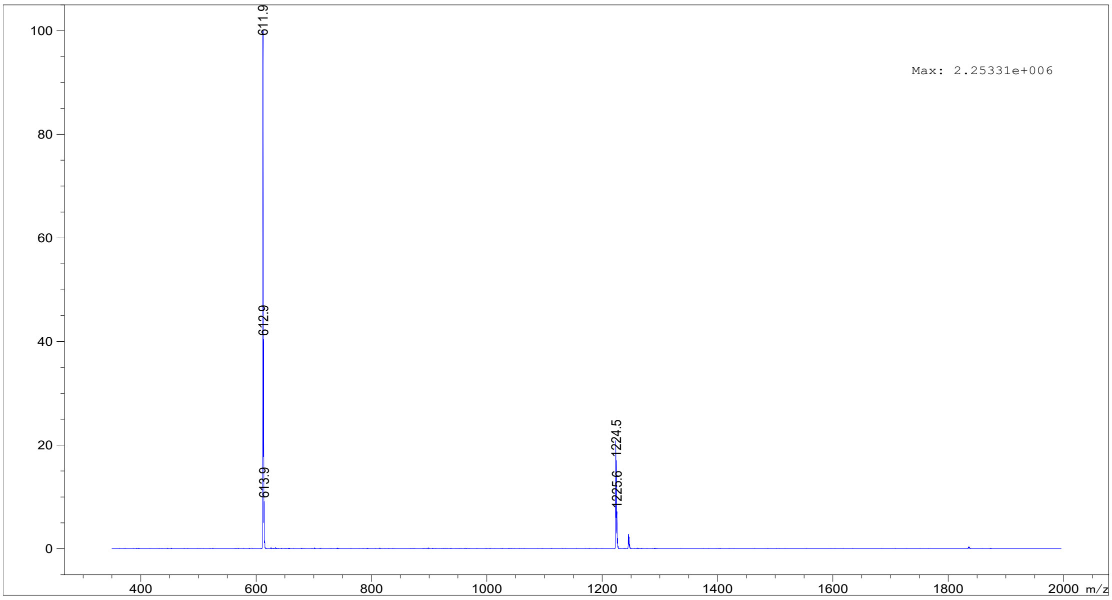

71. Compound **IQII:** obsvd. $[M+H^{+}] = 486.2$

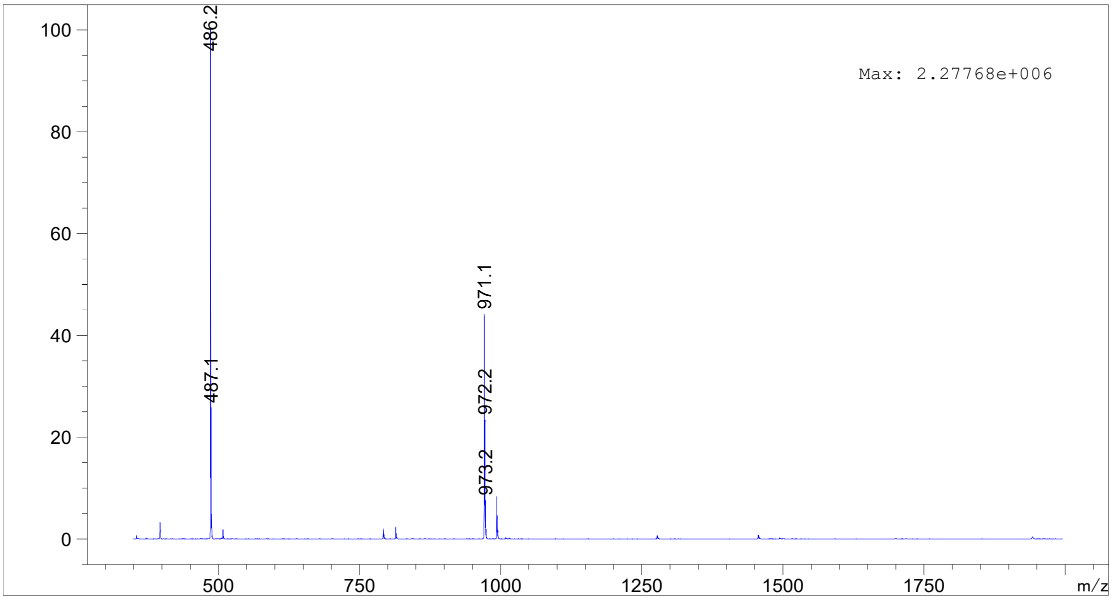

72. Compound **LVIF:** obsvd. [M+ H$^+$] = 491.3

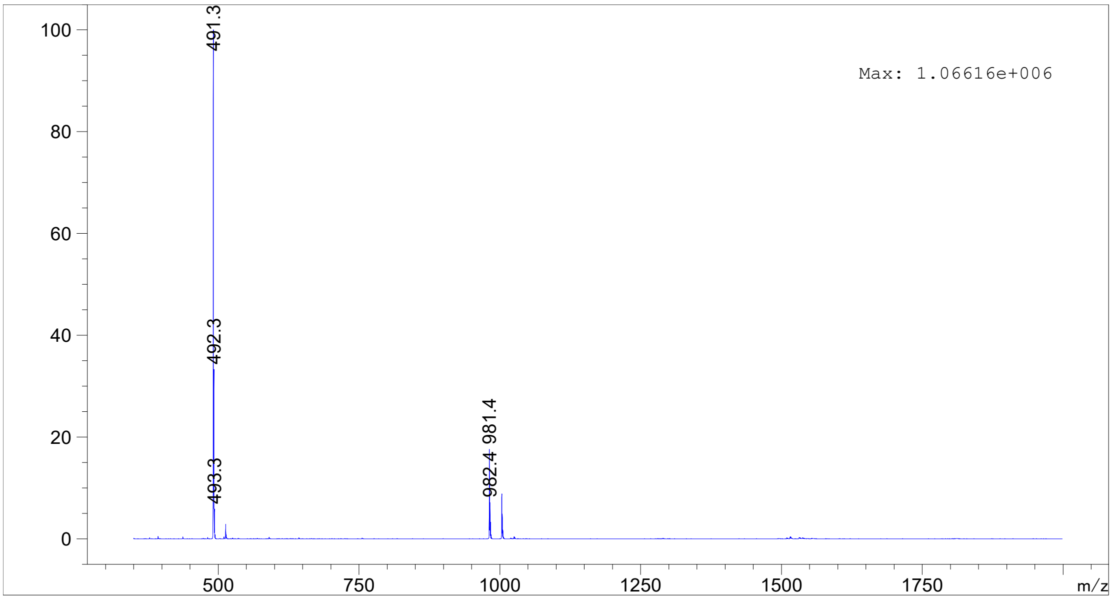

73. Compound **LVWF:** obsvd. [M+ H$^+$] = 564.3

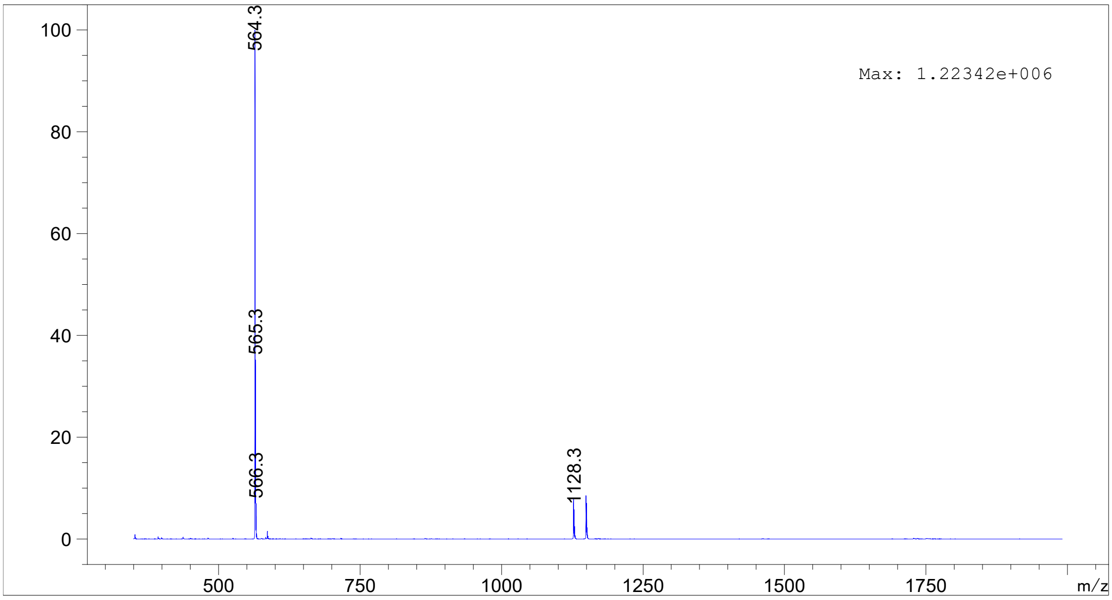

74. Compound **WVII:** obsvd. [M+ H$^+$] = 530.3

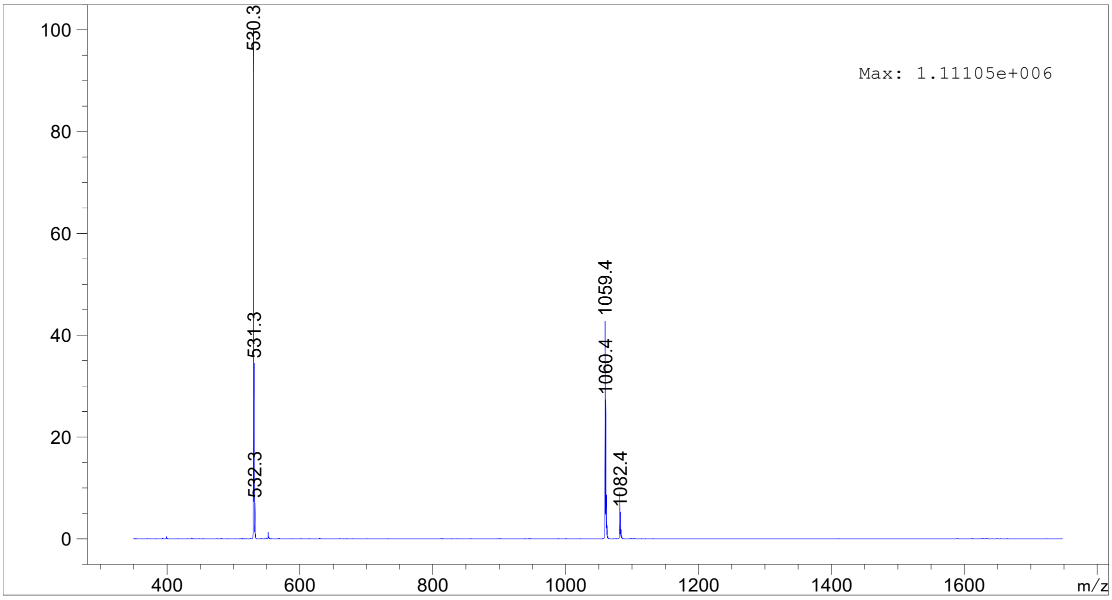

75. Compound **LVIY:** obsvd. [M+ H$^+$] = 507.3

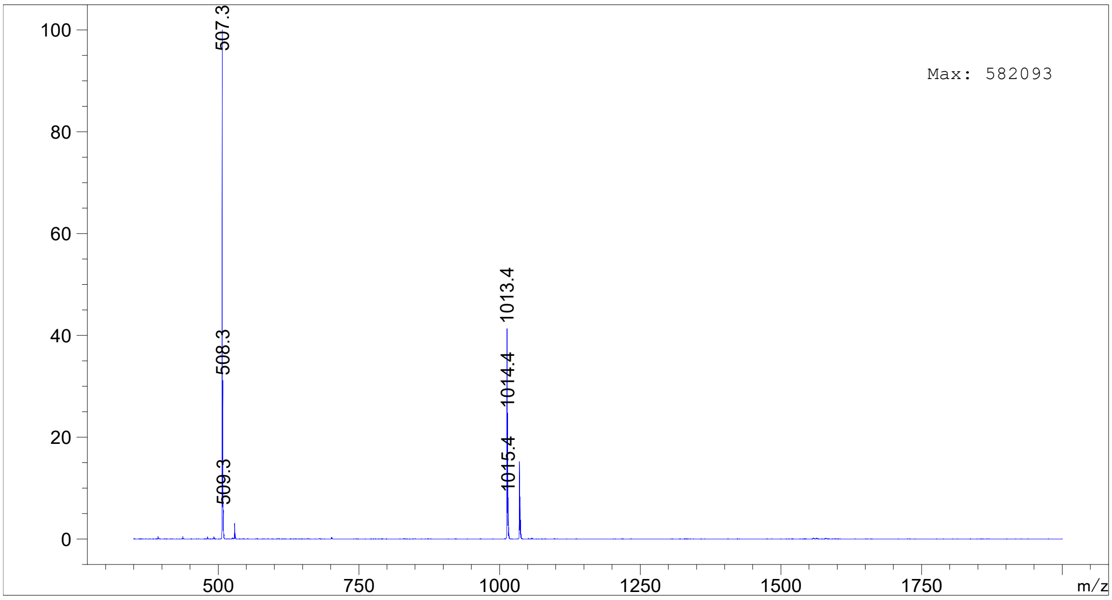

76. Compound **QMVV:** obsvd. [M+ H$^+$] = 476.0

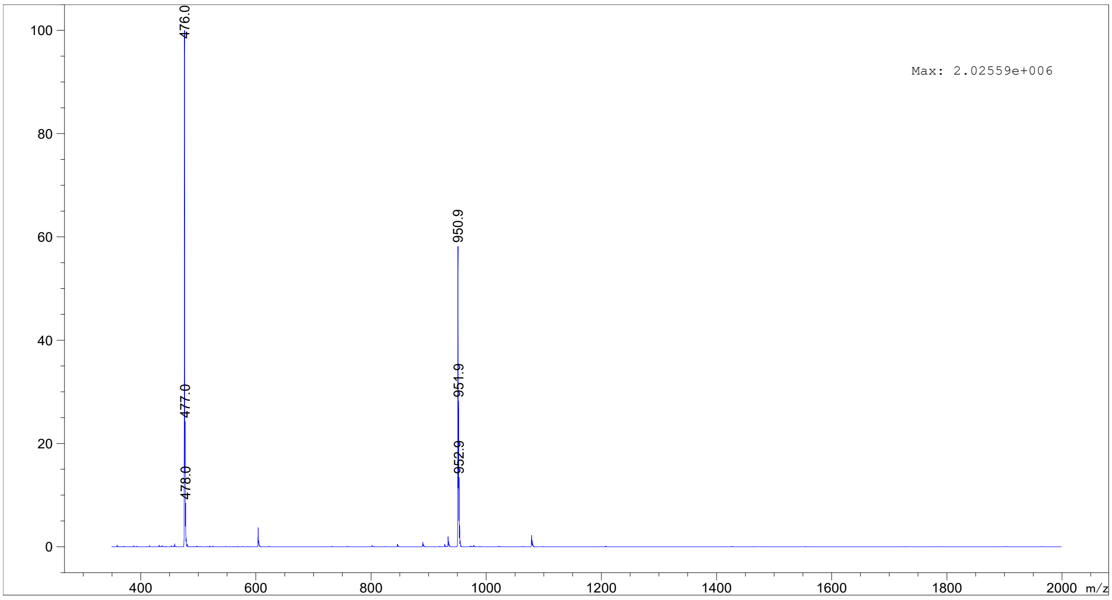

77. Compound **WTIF:** obsvd. [M+ H$^+$] = 566.0

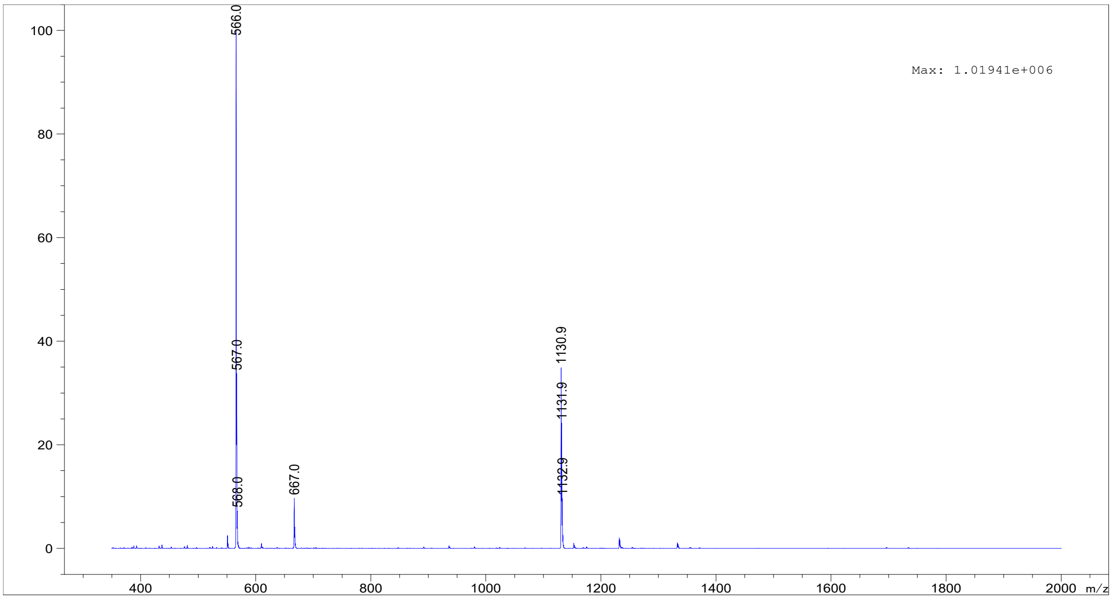

78. Compound **IVIY:** obsvd. [M+ H^+] = 507.0

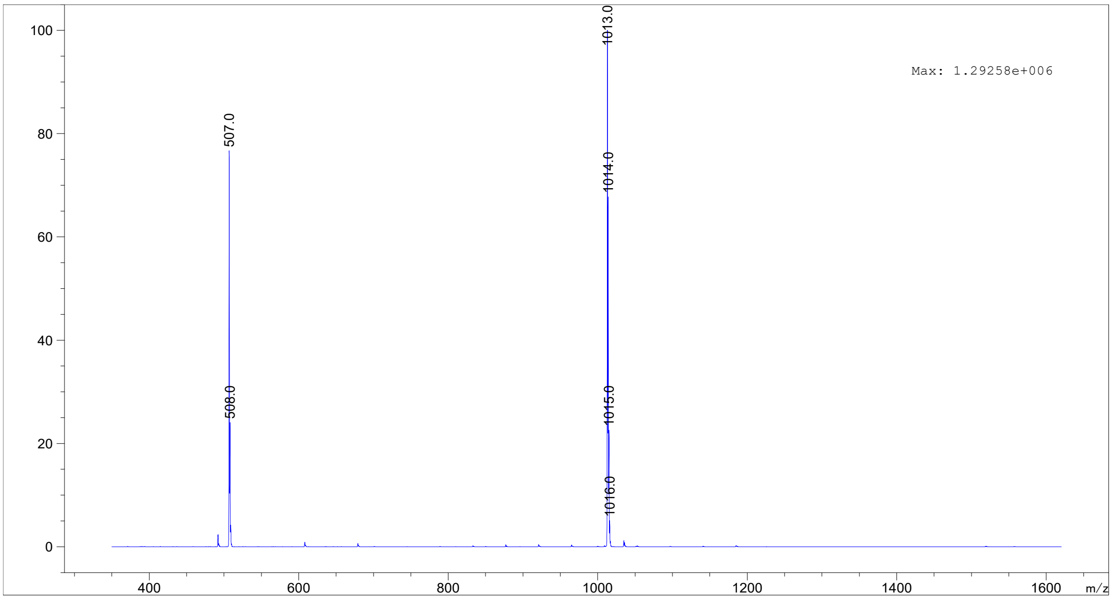

79. Compound **NTIF:** obsvd. [M+ H^+] = 494.0

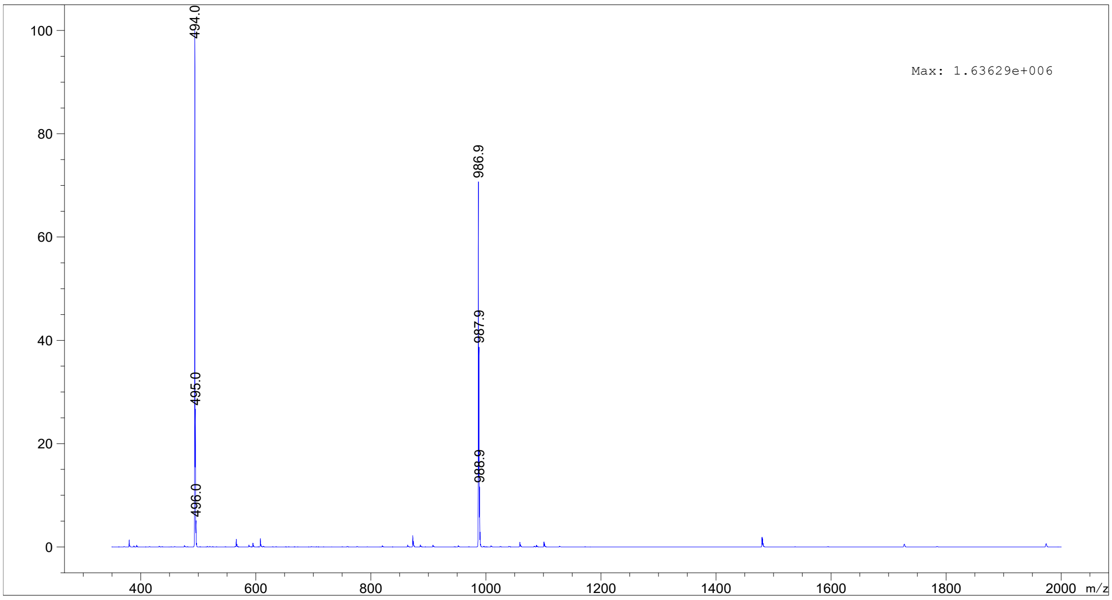

80. Compound **FVIY:** obsvd. [M+ H^+] = 541.0

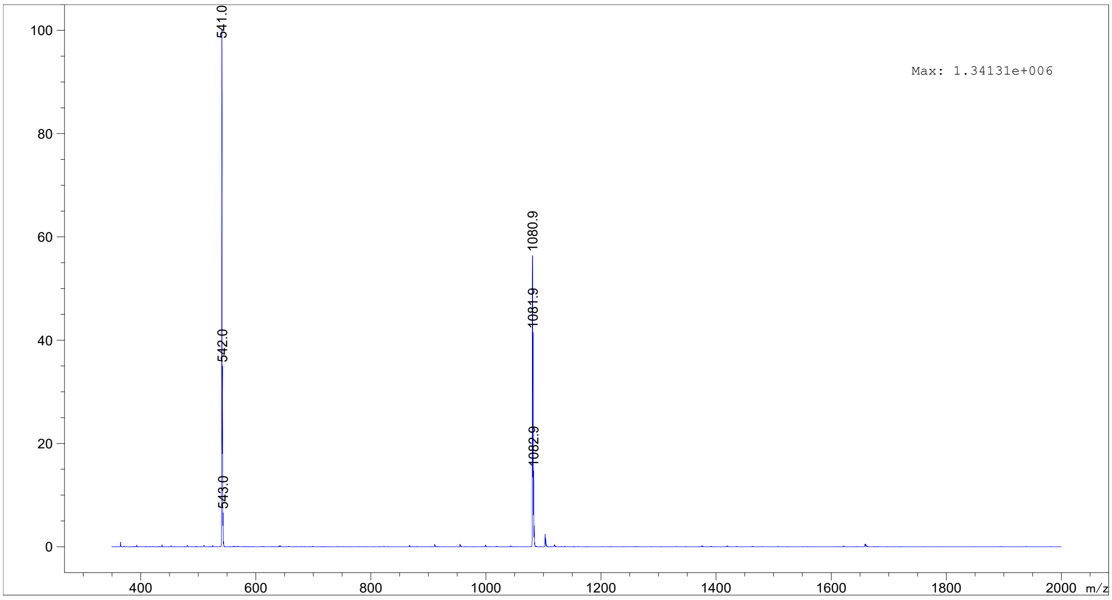

81. Compound **LLLF:** obsvd. [M+ H^+] = 505.0

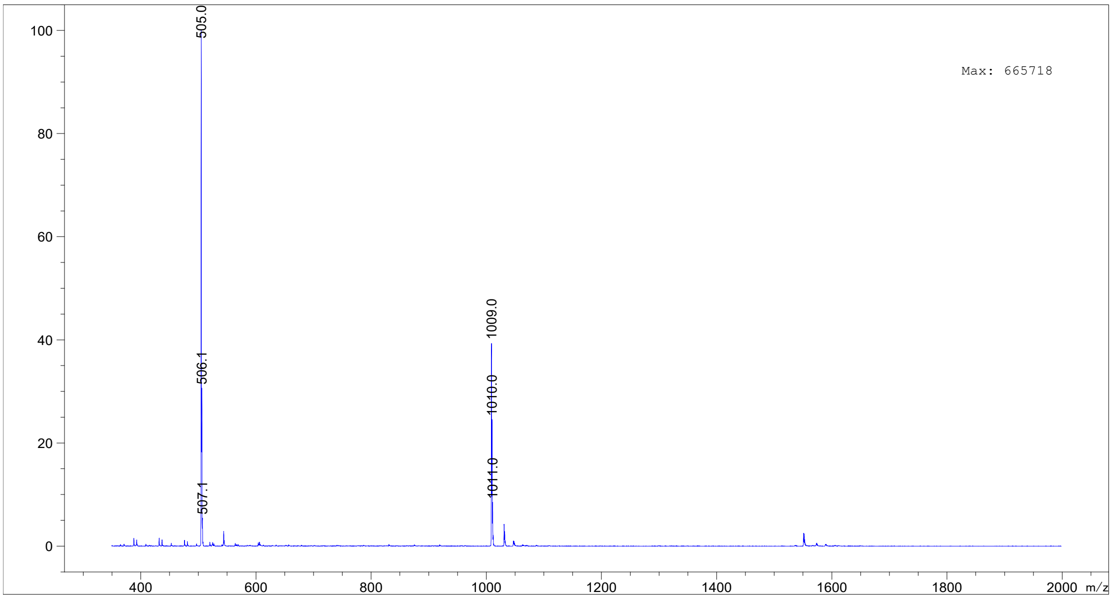

82. Compound **LVHF:** obsvd. [M+ H$^+$] = 515.0

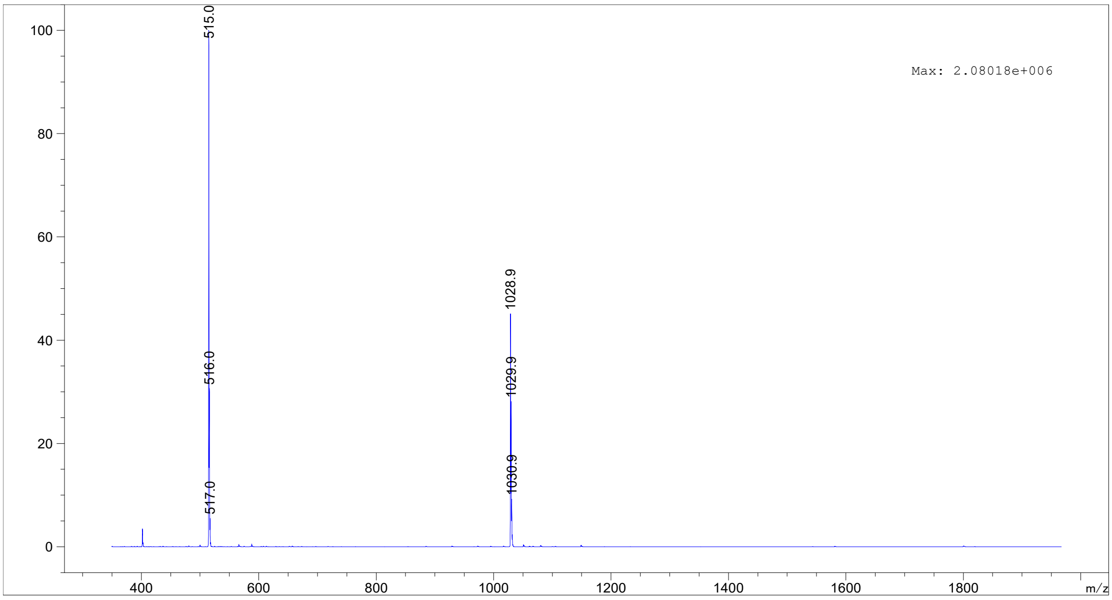

83. Compound **PIFY:** obsvd. [M+ H$^+$] = 539.0

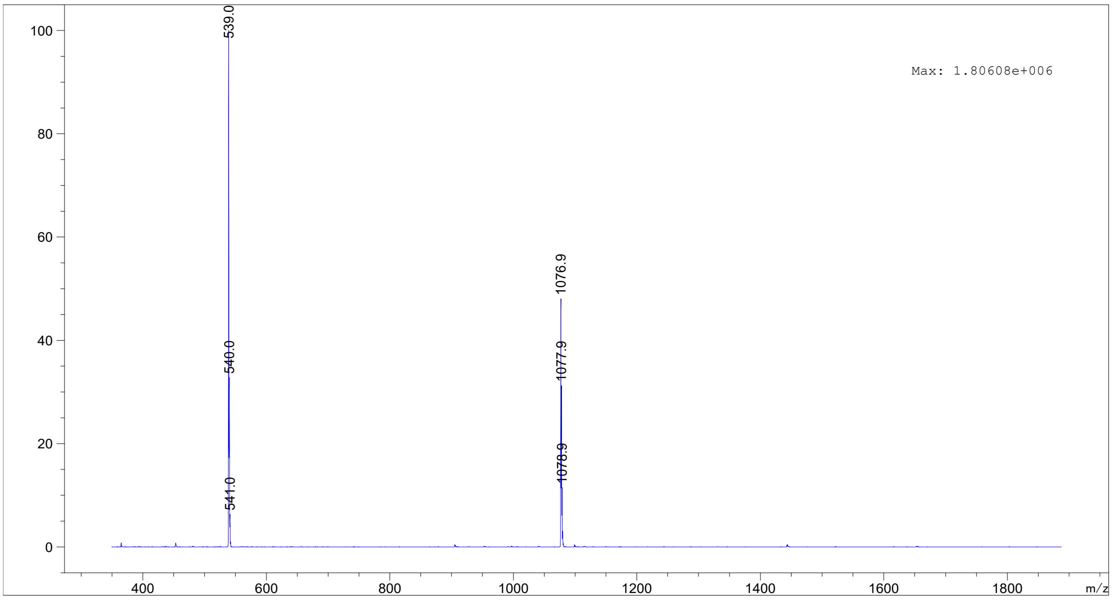

84. Compound **WIIF:** obsvd. [M+ H^+] = 578.0

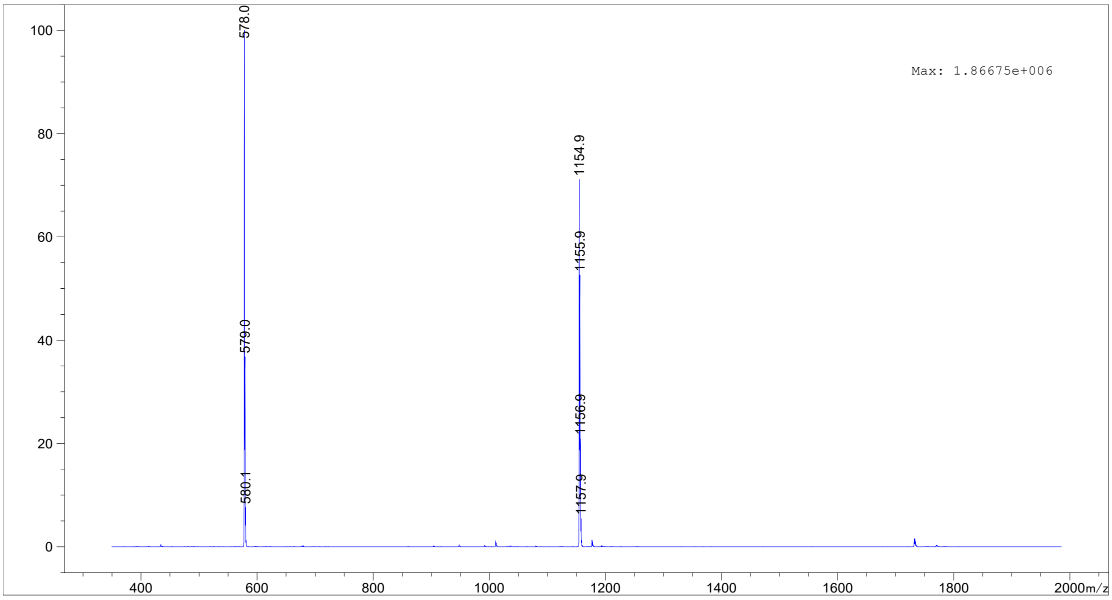

85. Compound **IFIY:** obsvd. [M+ H^+] = 555.0

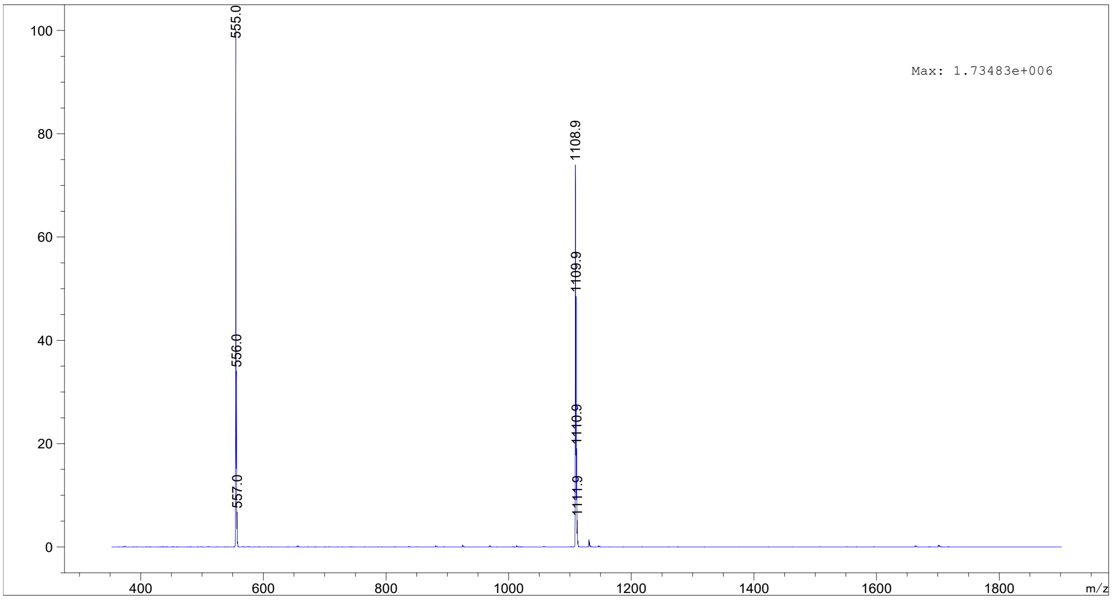

86. Compound **IVFW:** obsvd. [M+ H^+] = 564.0

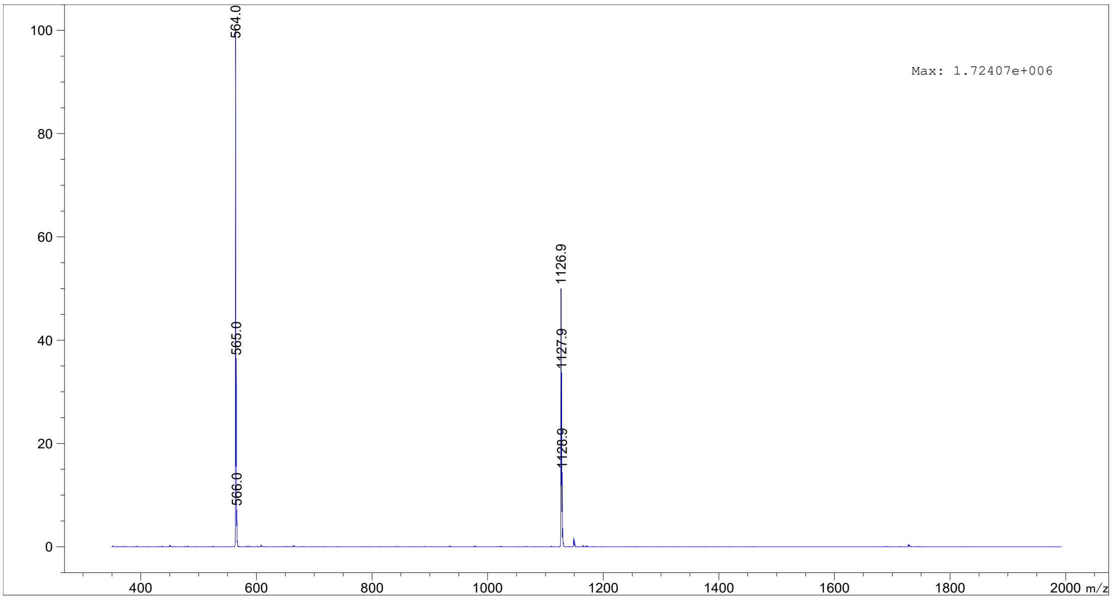

87. Compound **VLVV:** obsvd. [M+ H^+] = 428.6

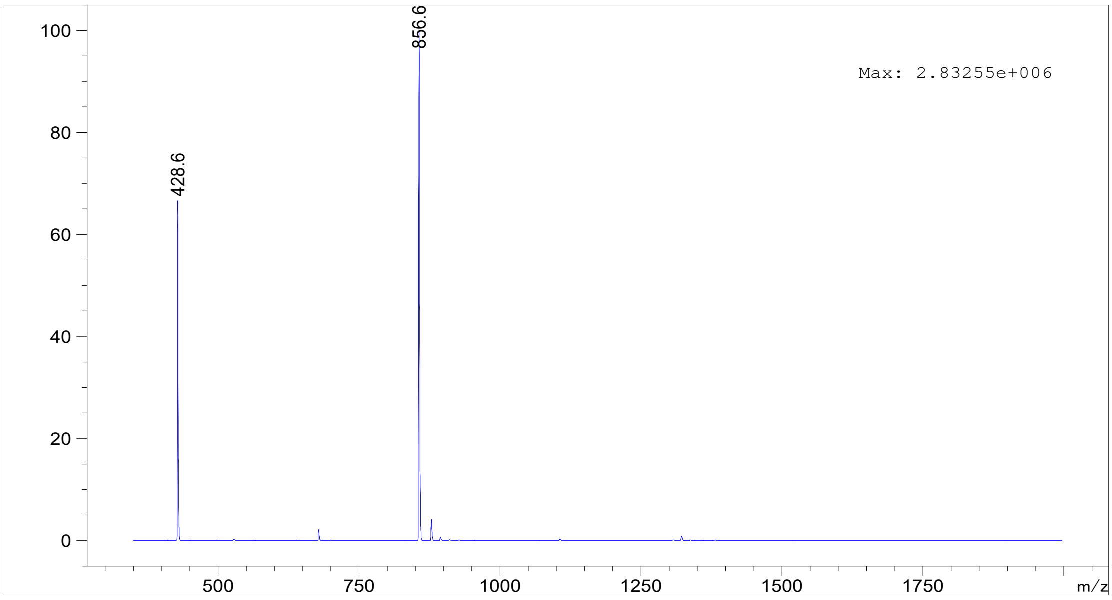

88. Compound **HLLF:** obsvd. $[M+H^{+}]$ = 529.3

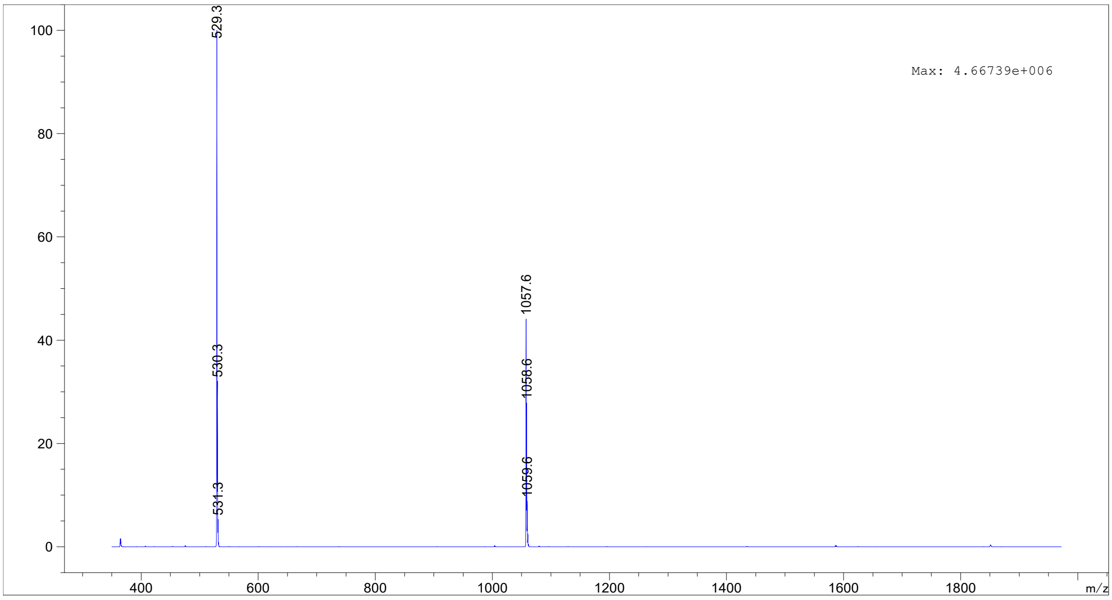

89. Compound **IVIF:** obsvd. $[M+H^{+}]$ = 491.4

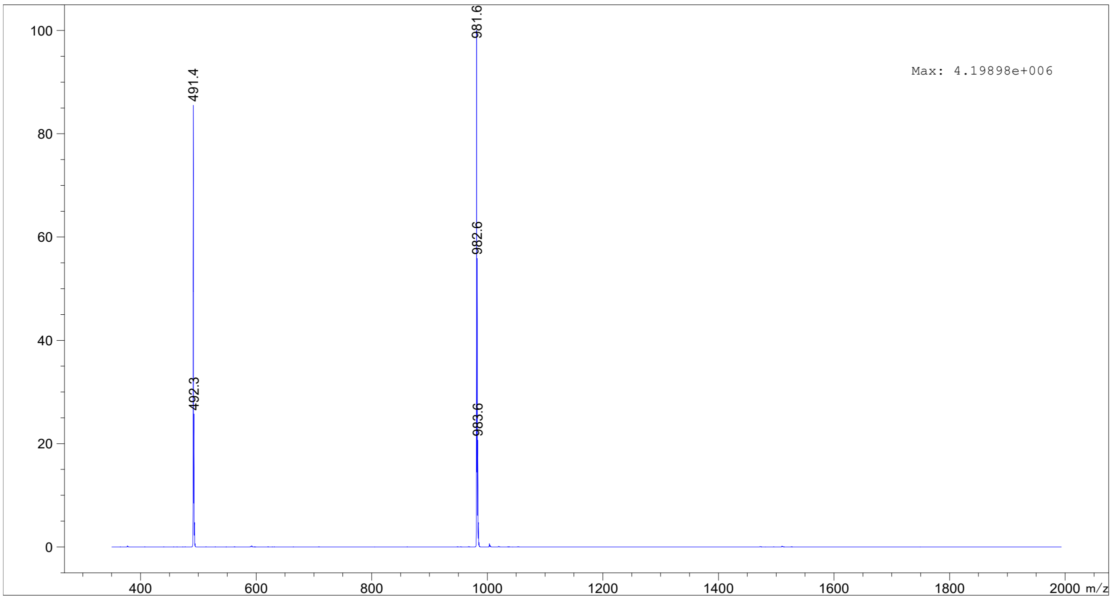

90. Compound **LLHF:** obsvd. [M+ H^+] = 529.3

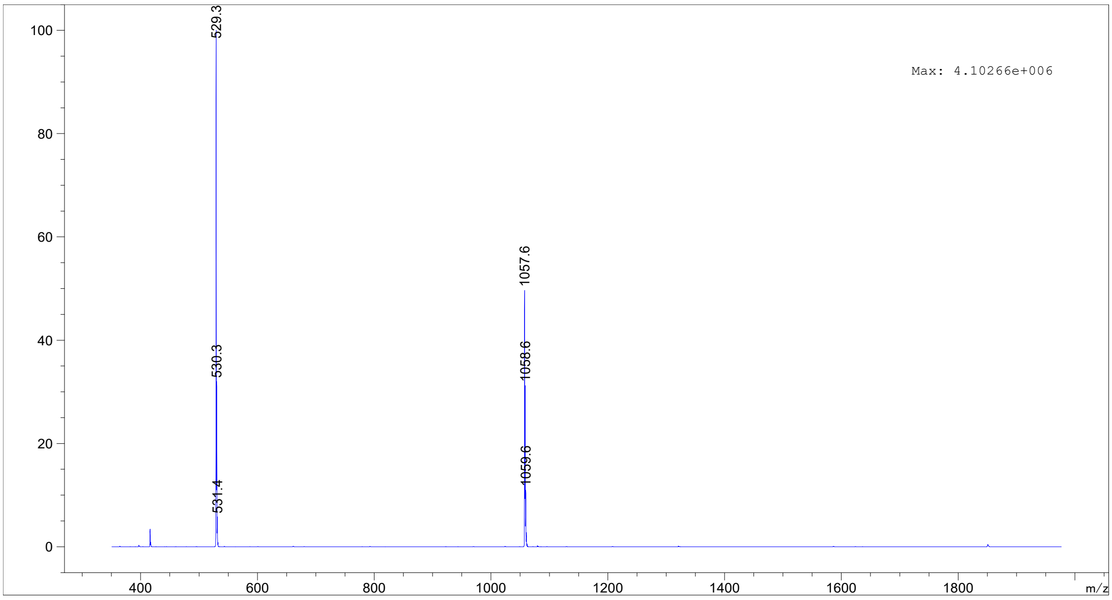

91. Compound **LLLY:** obsvd. [M+ H^+] = 521.4

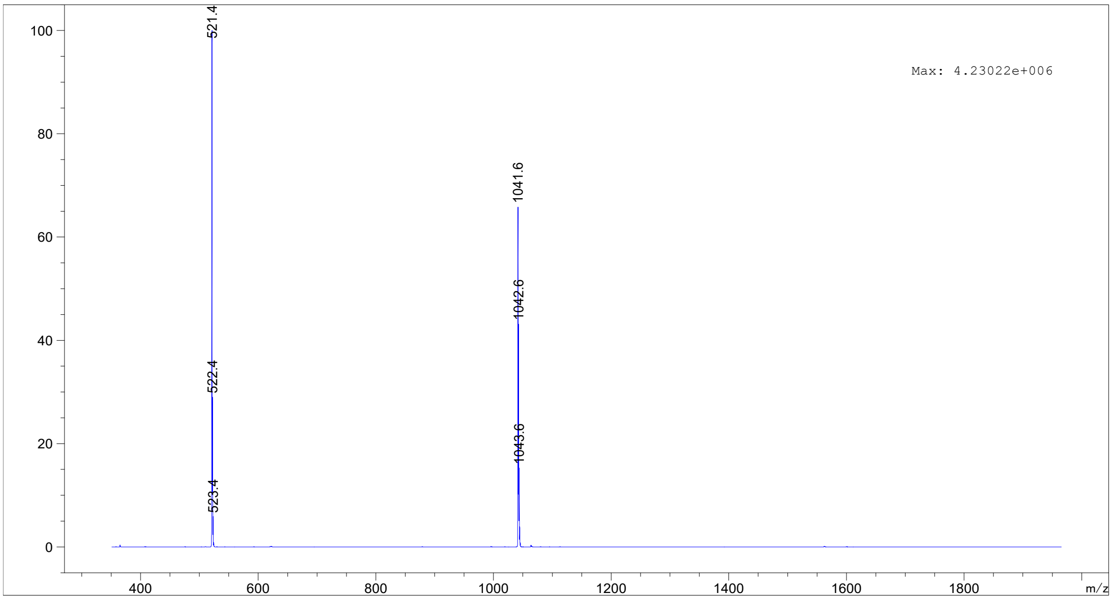

92. Compound **CIYF:** obsvd. [M+ H$^+$] = 545.3

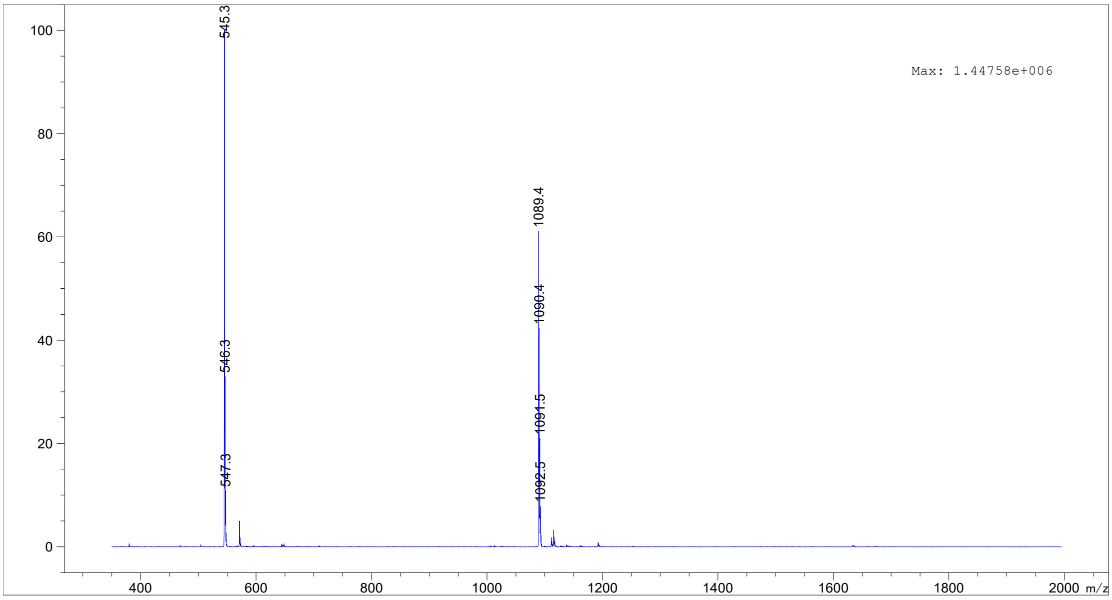

93. Compound **PVFW:** obsvd. [M+ H$^+$] = 548.3

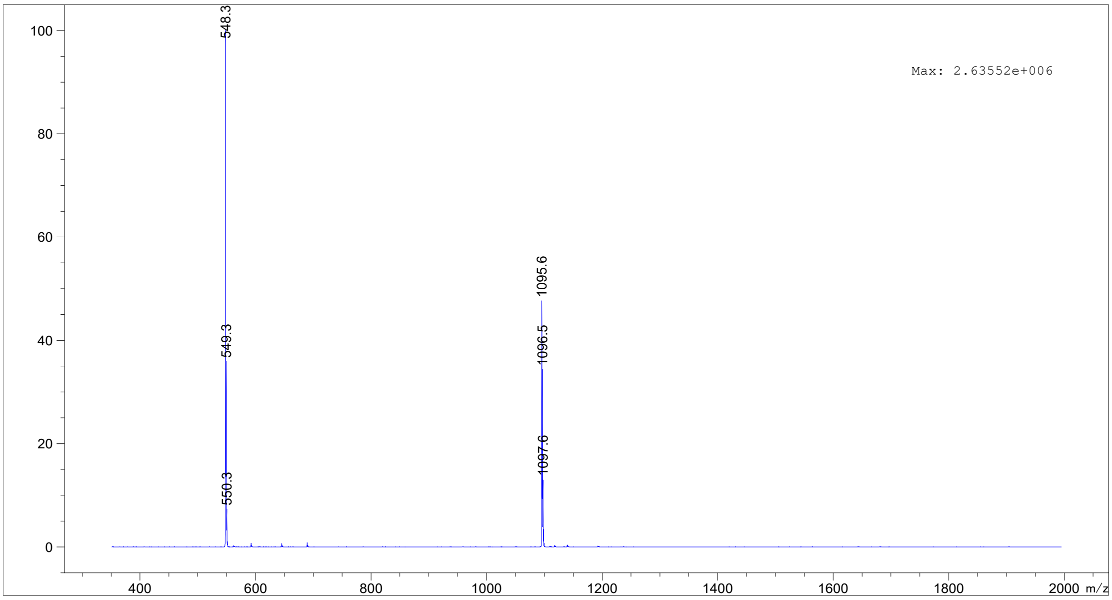

94. Compound **PVII:** obsvd. [M+ H$^+$] = 441.3

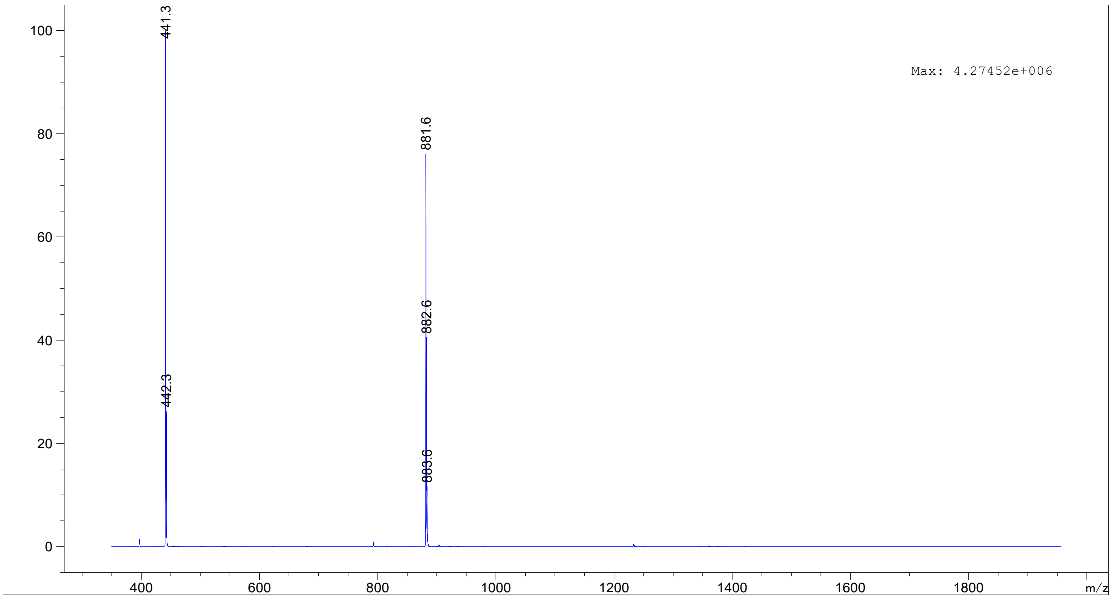

95. Compound **PWYY:** obsvd. [M+ H$^+$] = 628.4

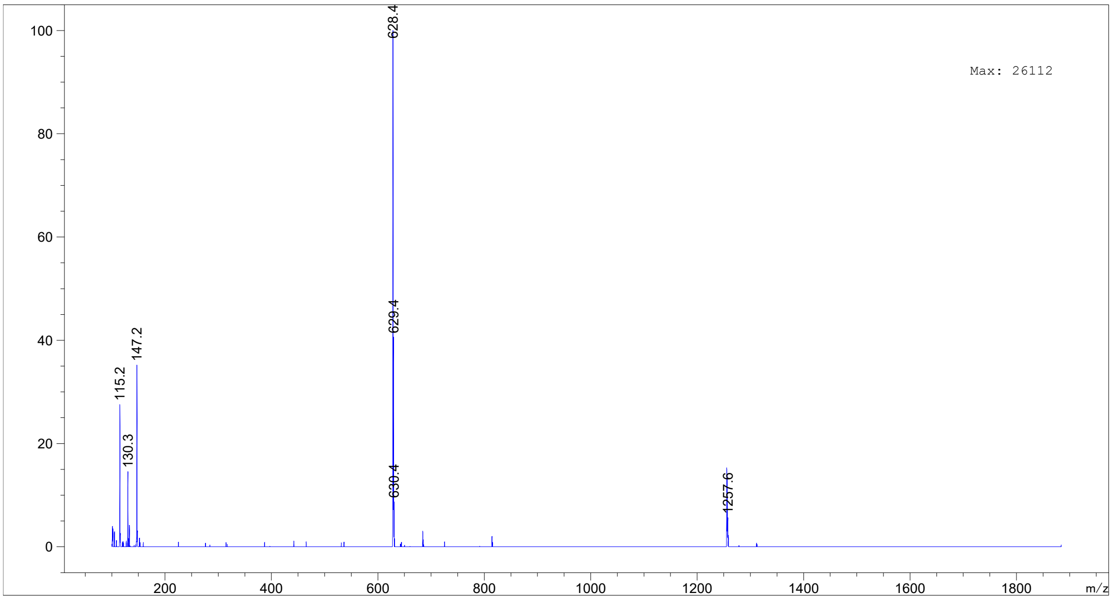

96. Compound **WAFY:** obsvd. [M+ H^{+}] = 586.5

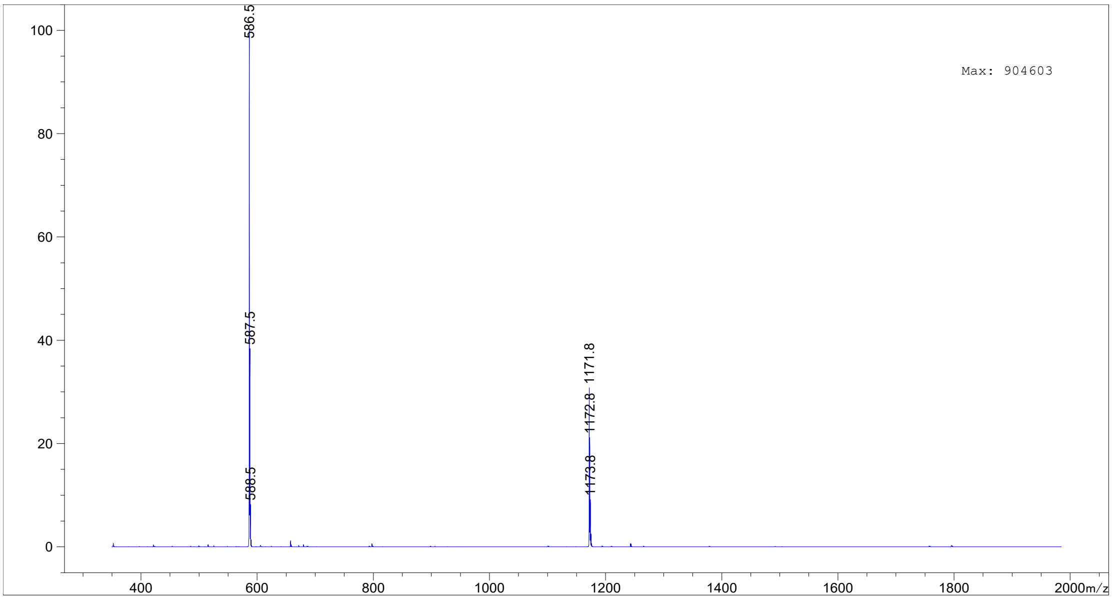

97. Compound **FHLF:** obsvd. [M+ H^{+}] = 563.1

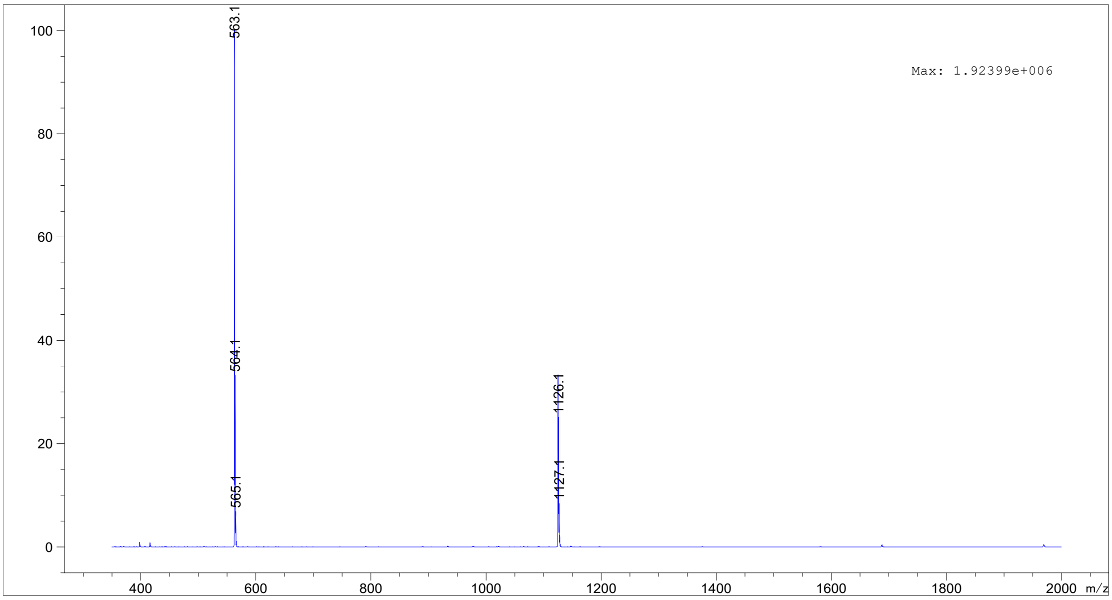

98. Compound **LIEM:** obsvd. [M+ H$^+$] = 505.0

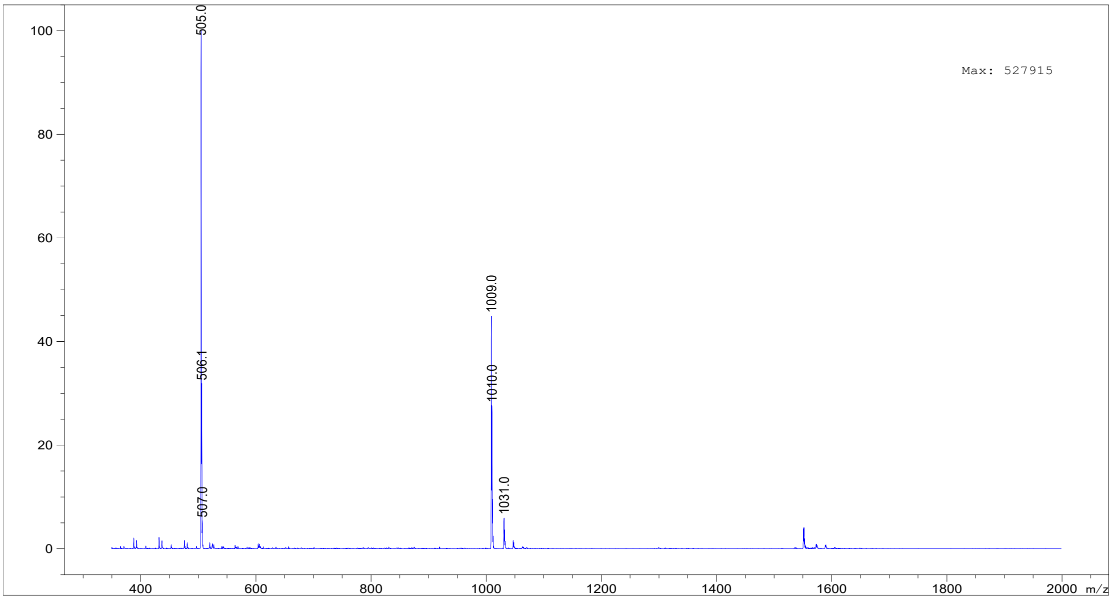

99. Compound **PMFY:** obsvd. [M+ H$^+$] = 557.3

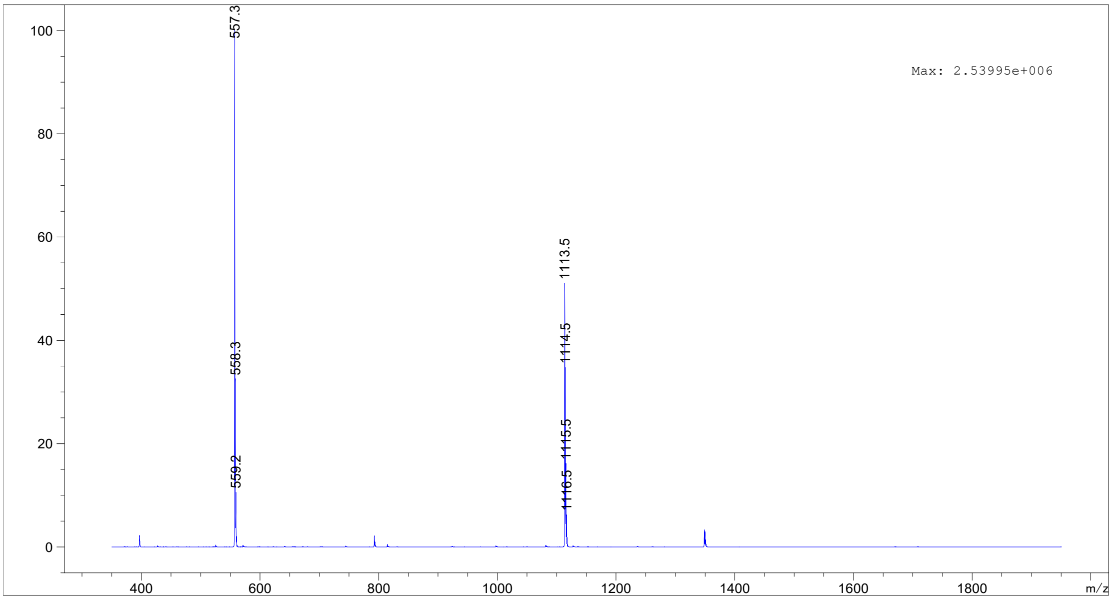

100.Compound **FCWW:** obsvd. [M+ H$^+$] = 640.9

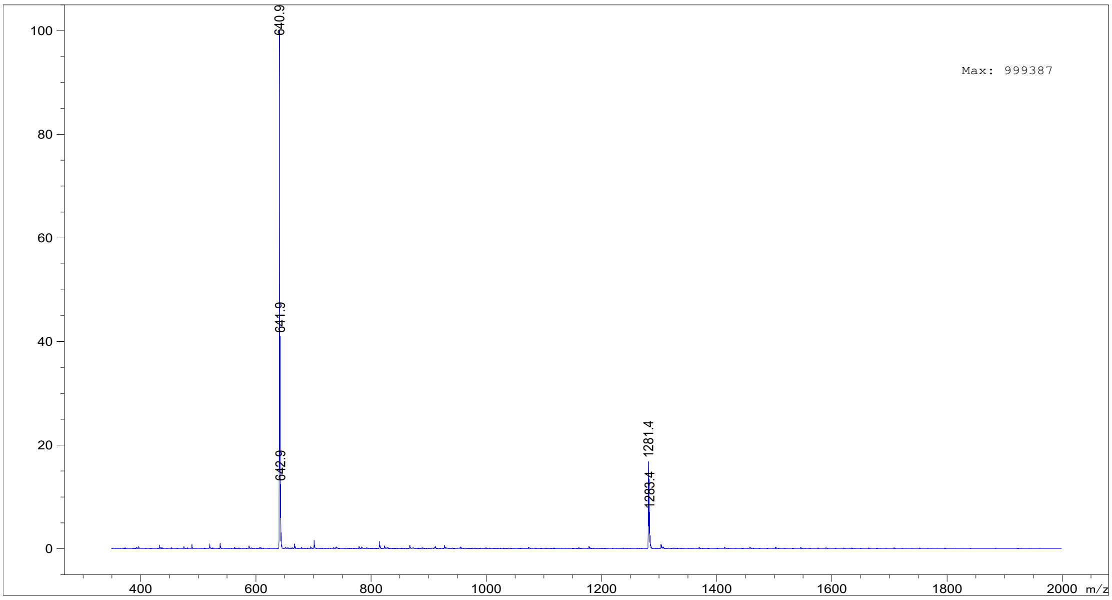

101.Compound **FGWW:** obsvd. [M+ H$^+$] = 595.2

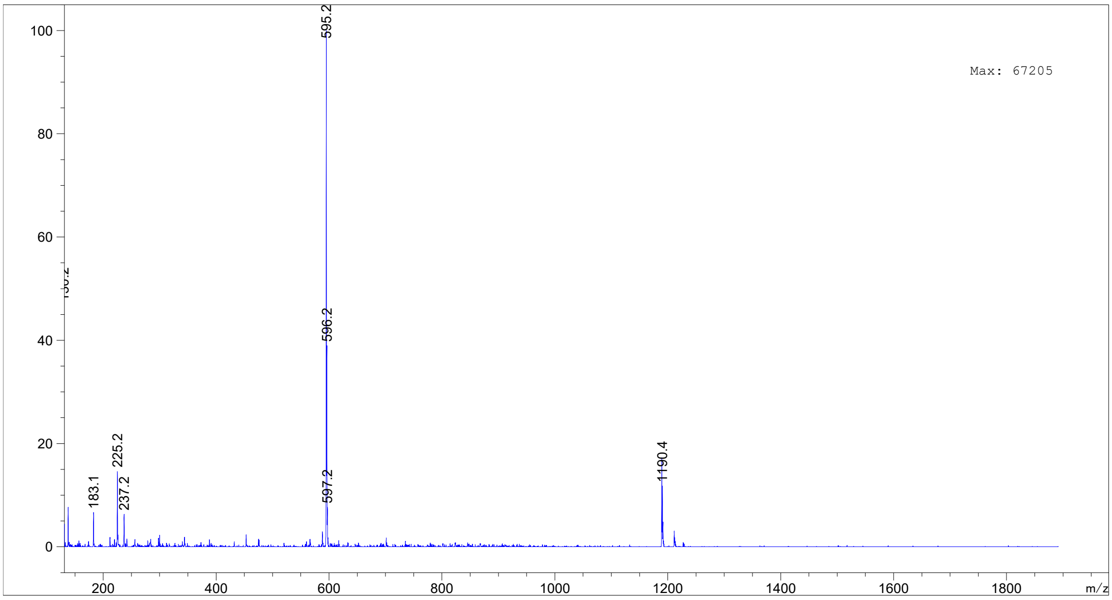

102.Compound **VWIT:** obsvd. [M+ H^+] = 518.2

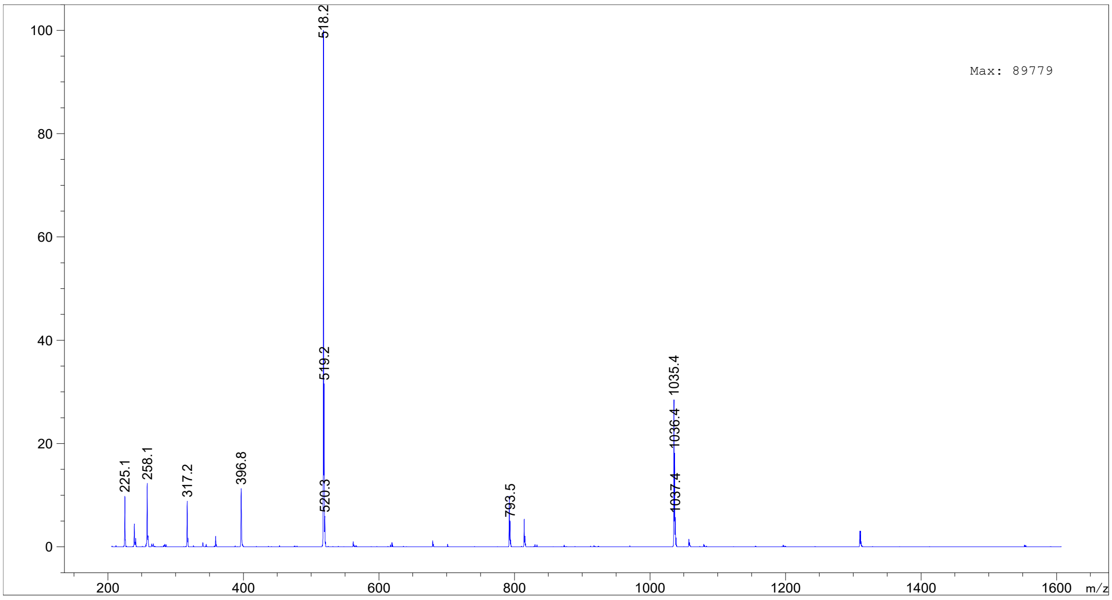

103.Compound **WWCP:** obsvd. [M+ H^+] = 590.8

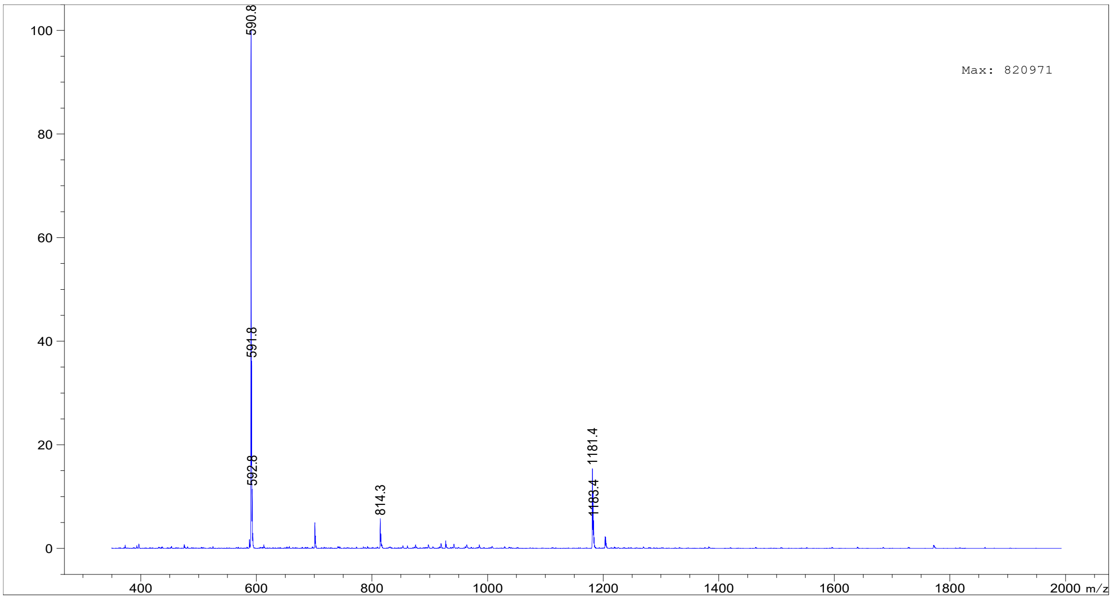

104.Compound **IWPW:** obsvd. [M+ H$^+$] = 600.9

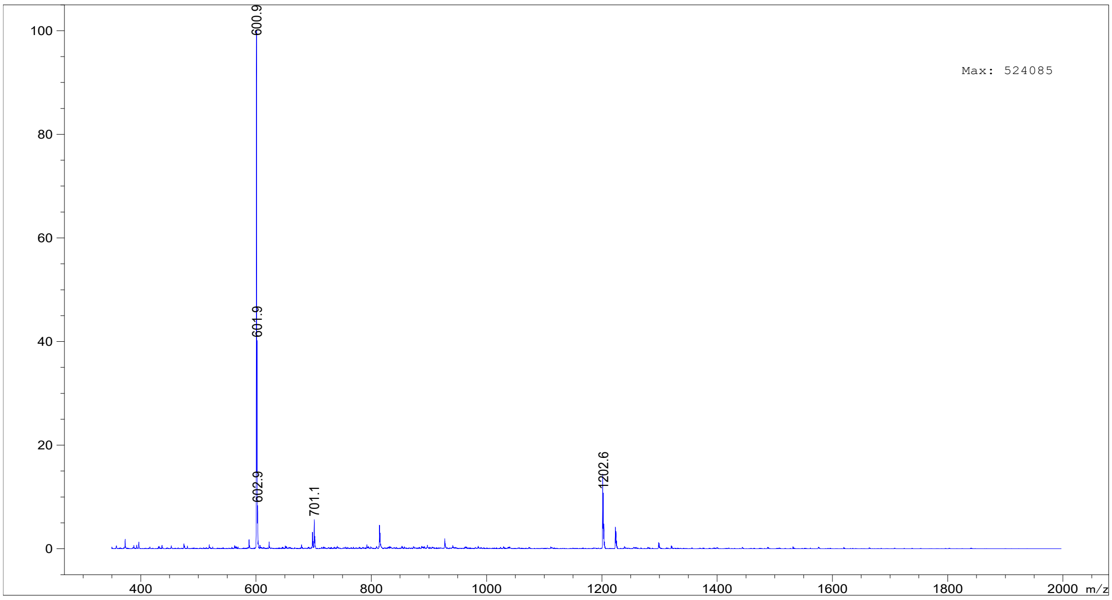

105.Compound **WPYY:** obsvd. [M+ H$^+$] = 628.3

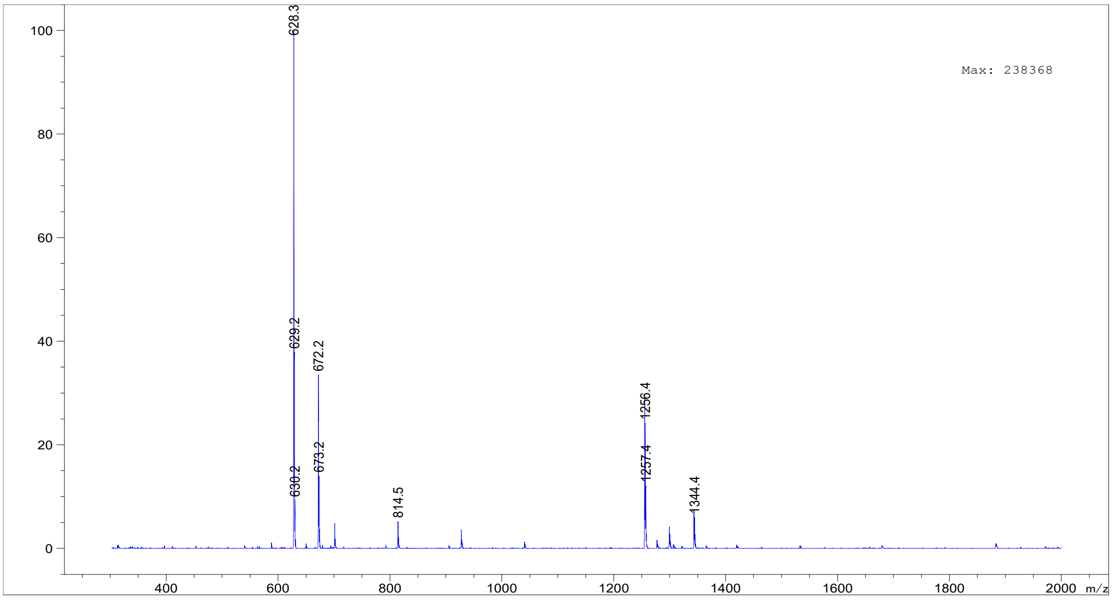

106.Compound **VSWY:** obsvd. [M+ H^{+}] = 553.9

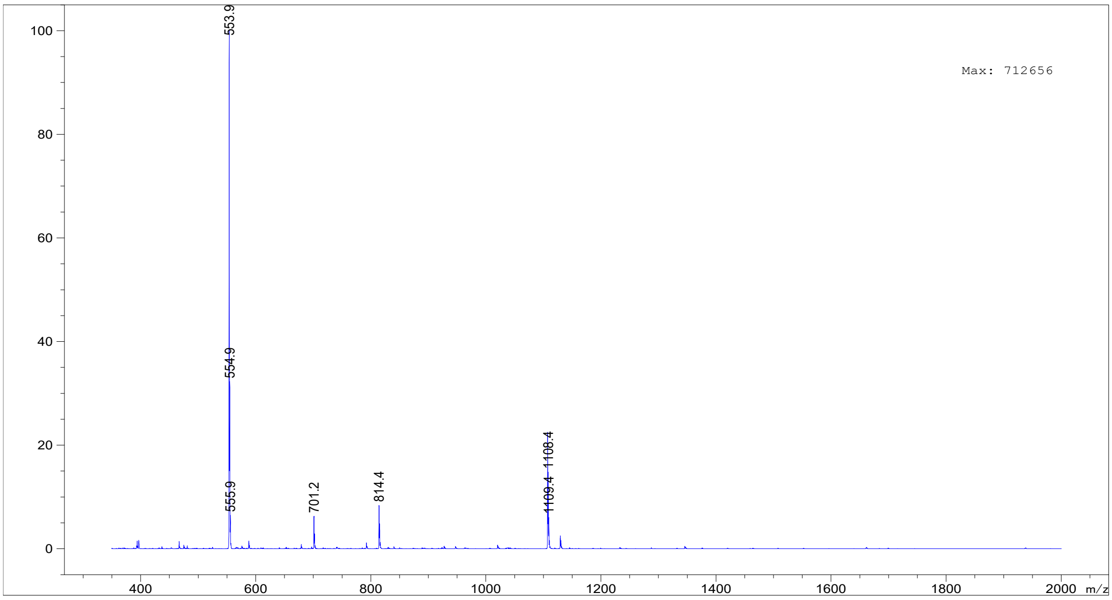

107.Compound **CAYW:** obsvd. [M+ H^{+}] = 542.0

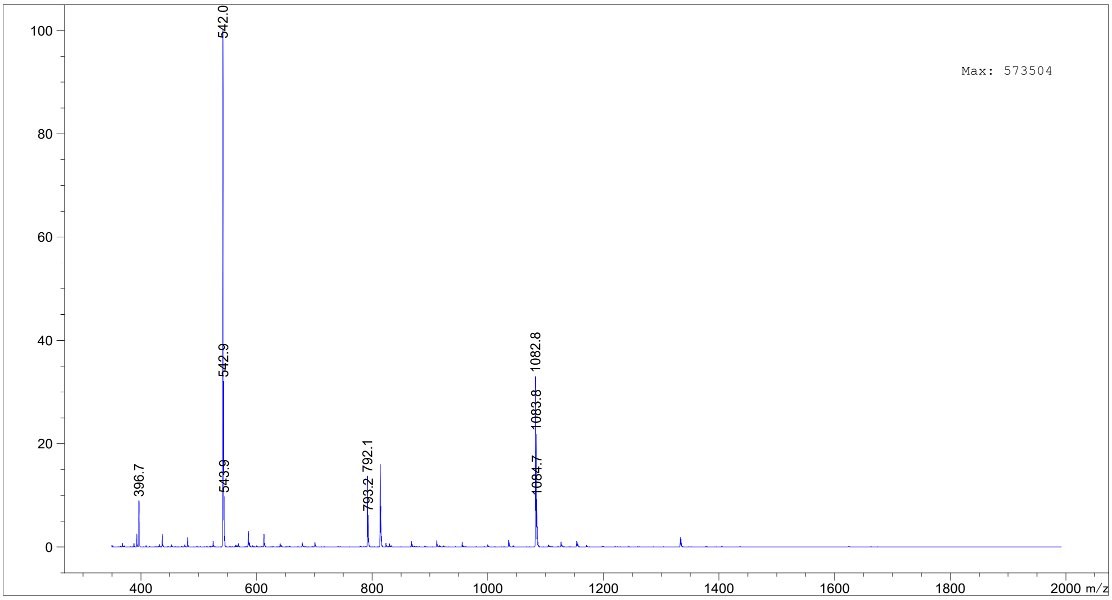

108.Compound **PWWY:** obsvd. [M+ H^{+}] = 651.4

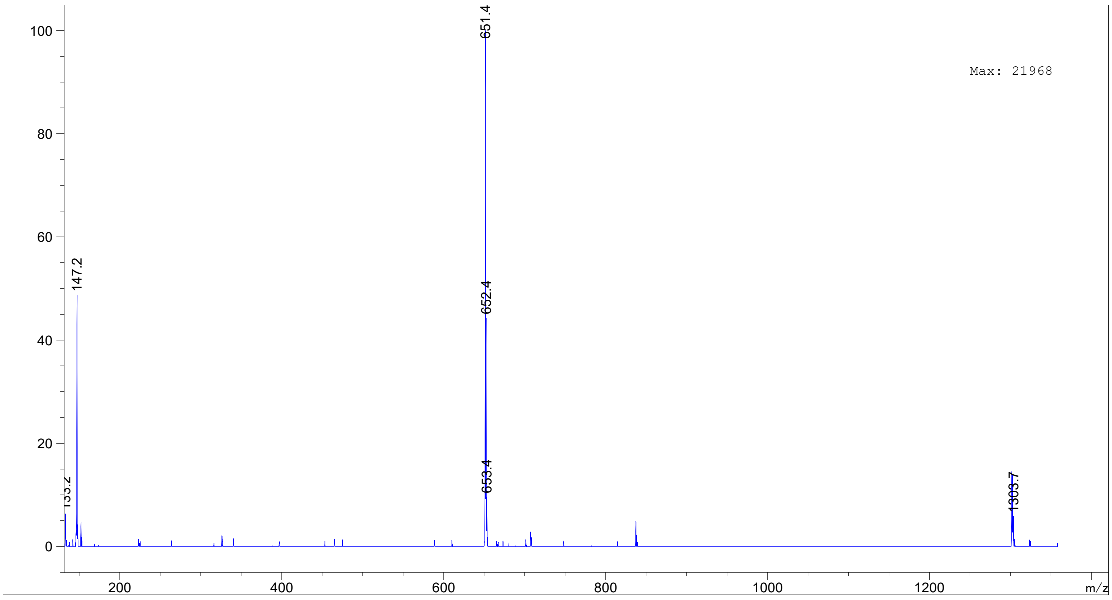

109.Compound **AAAA:** obsvd. [M+ H^{+}] = 303.3

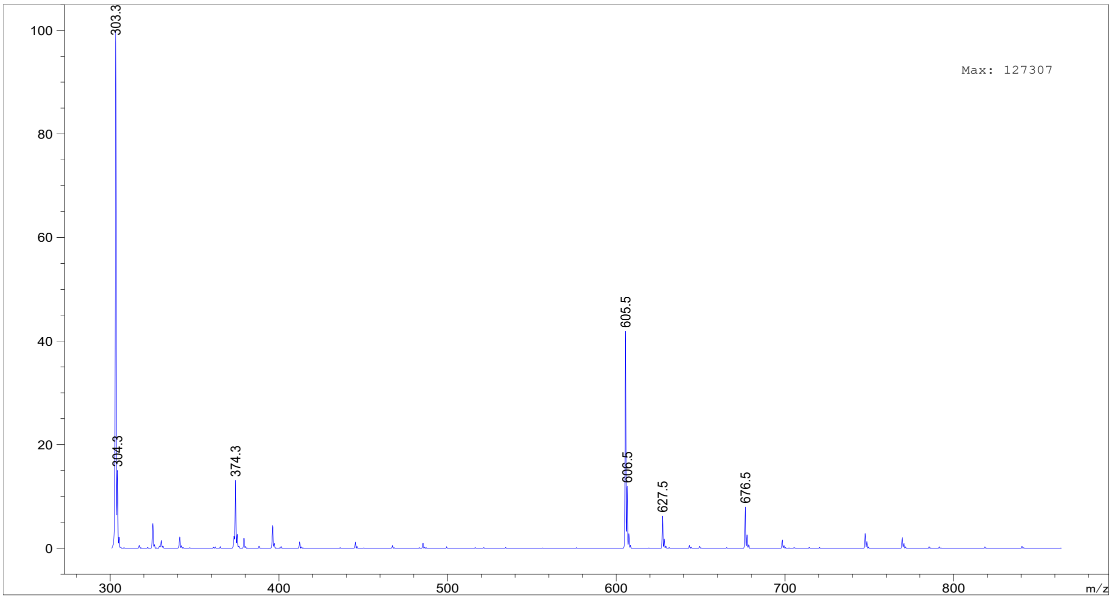

110.Compound **DDDD:** obsvd. $[M+ H^{+}] = 479.0$

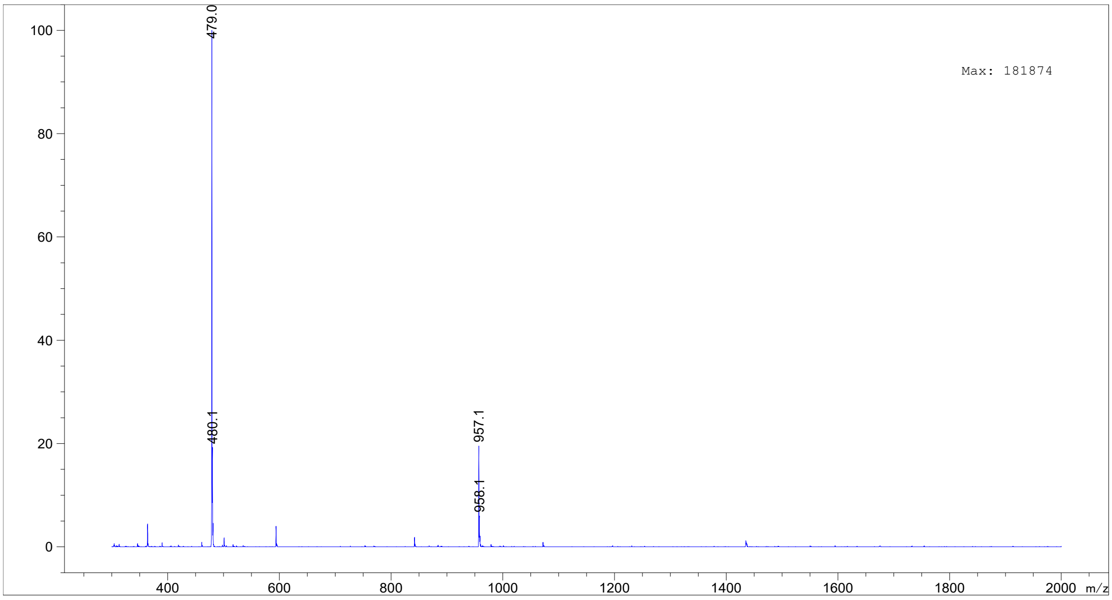

111.Compound **GGGG:** obsvd. $[M+ H^{+}] = 247.2$

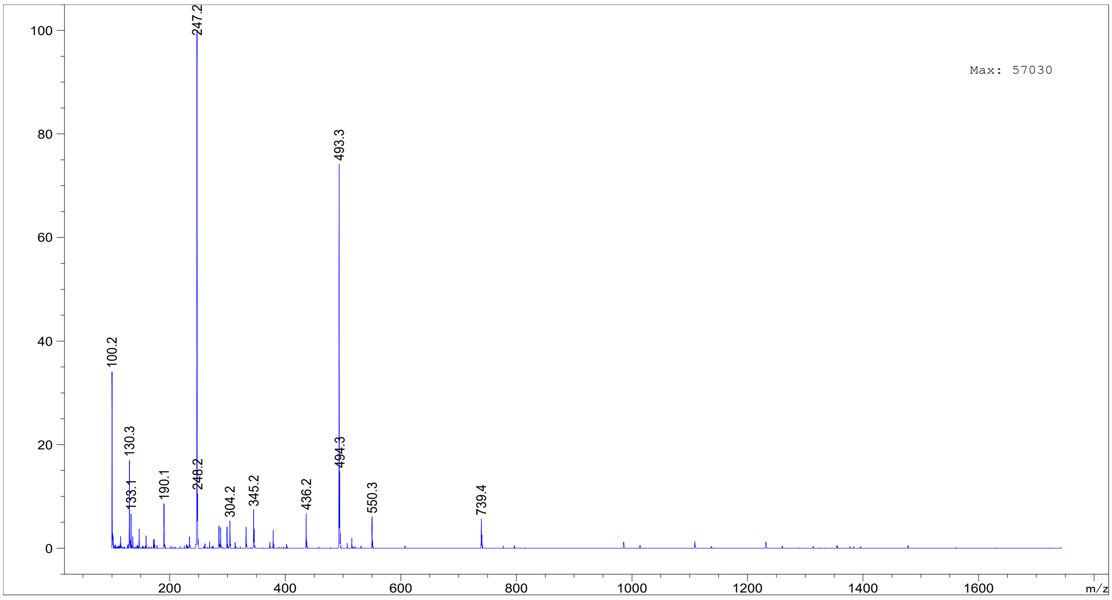

112.Compound **HHHH:** obsvd. [M+ H^+] = 567.4

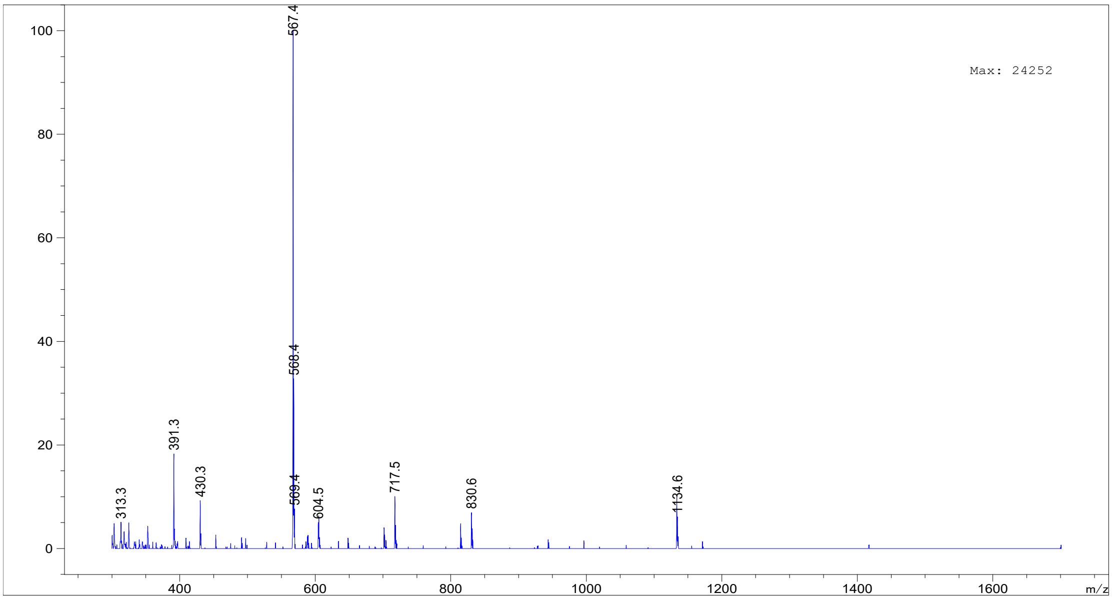

113.Compound **KKKK:** obsvd. [M+ H^+] = 531.5

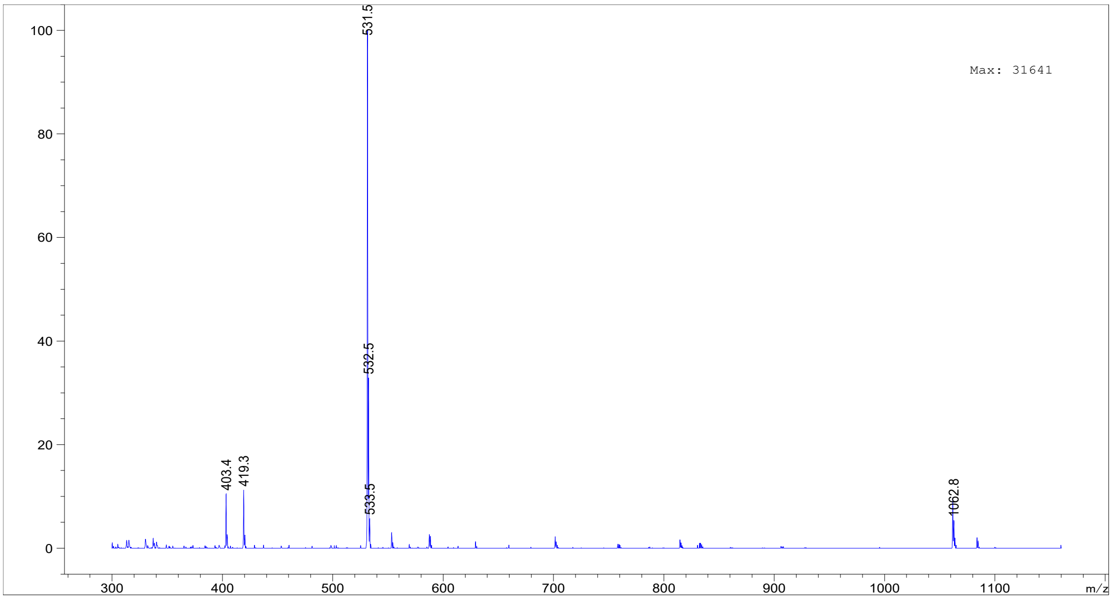

114.Compound **RRRR:** obsvd. [M+ H$^+$] = 643.6

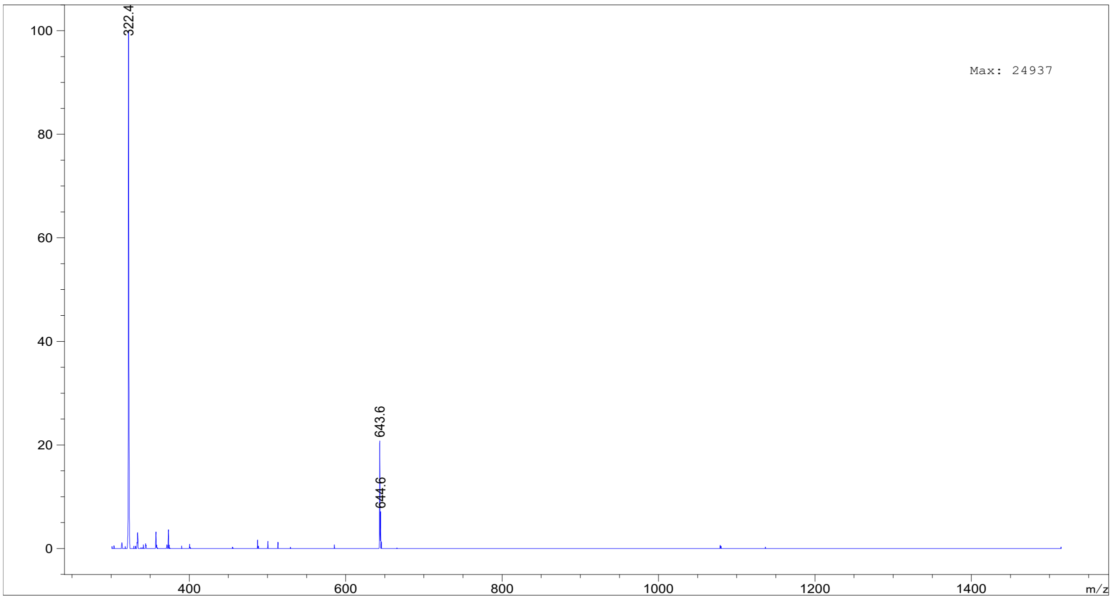

115.Compound **YYYY:** obsvd. [M+ H$^+$] = 671.4

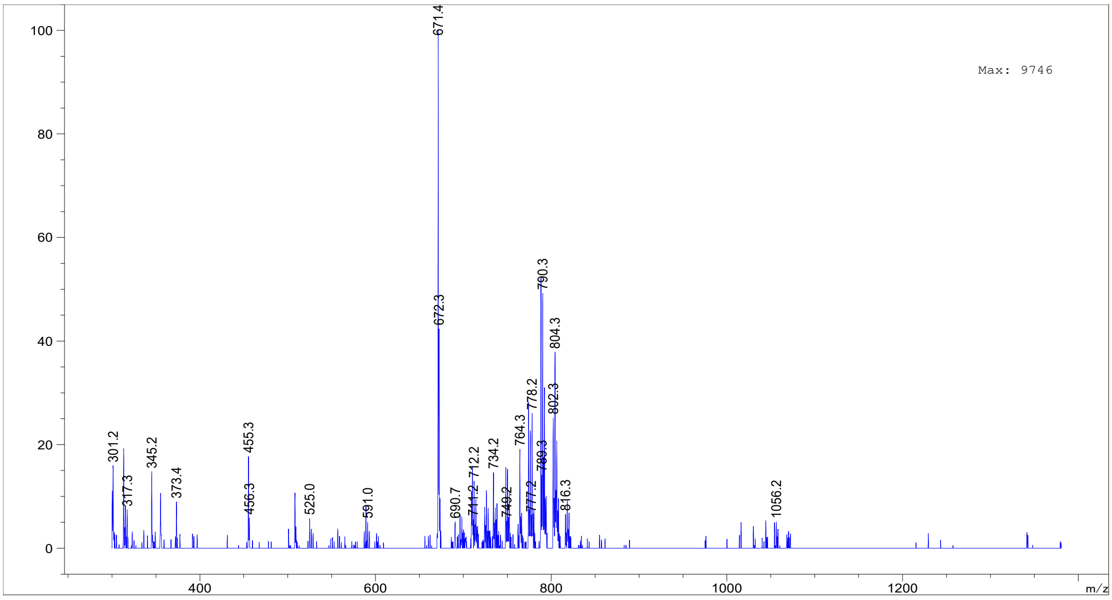

116.Compound **WFHP:** obsvd. [M+ H$^+$] = 586.2

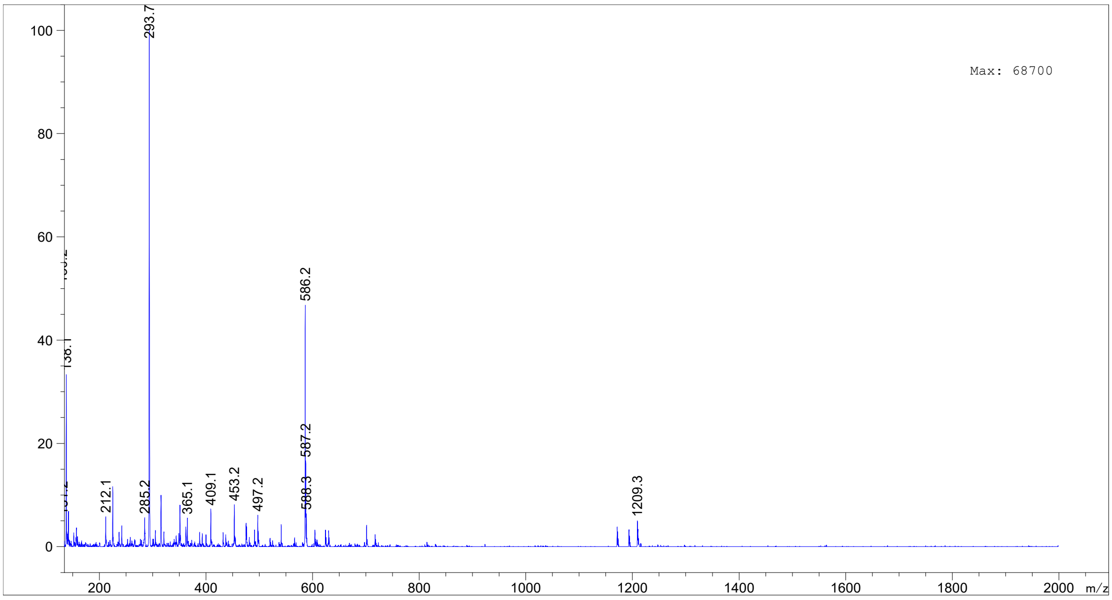

117.Compound **EFFK:** obsvd. [M − H$^-$] = 568.2

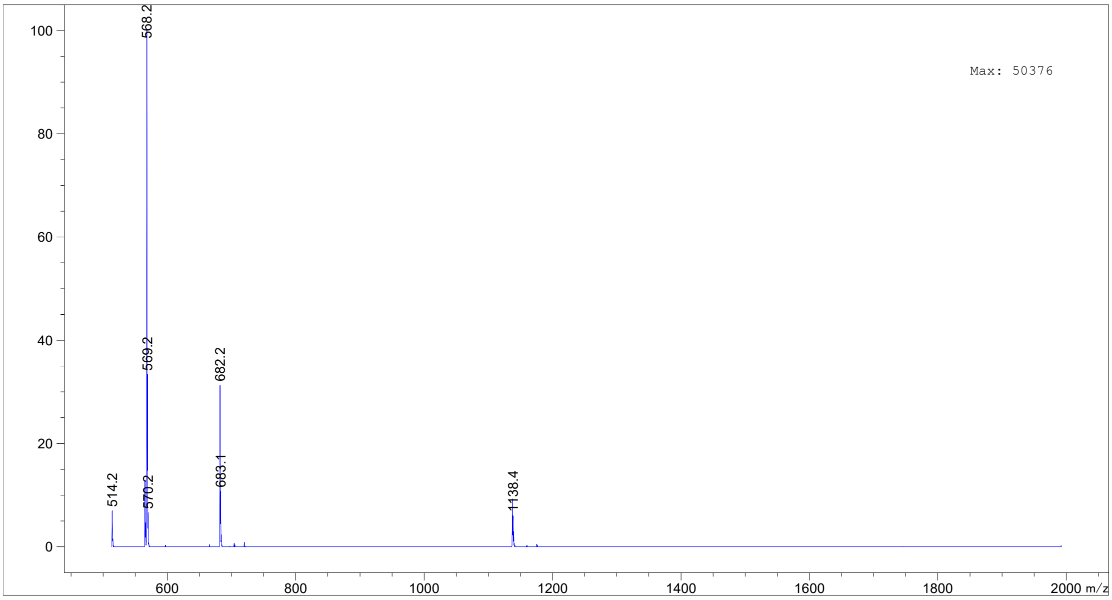

118.Compound **EEKK:** obsvd. $[M-H^-] = 531.2$

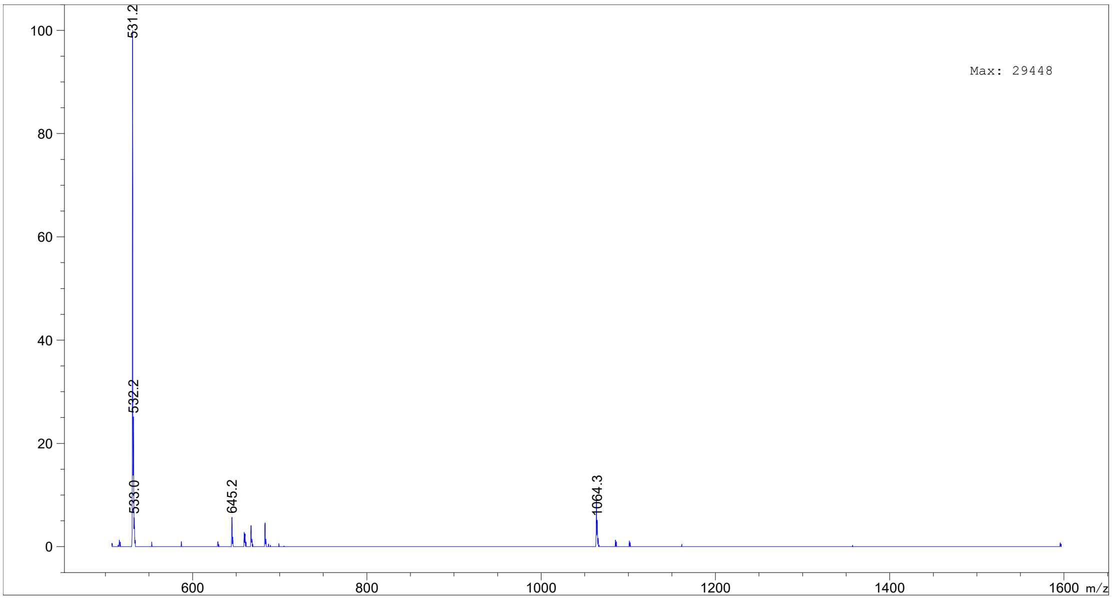

119.Compound **LGVF:** obsvd. $[M+H^+] = 435.2$

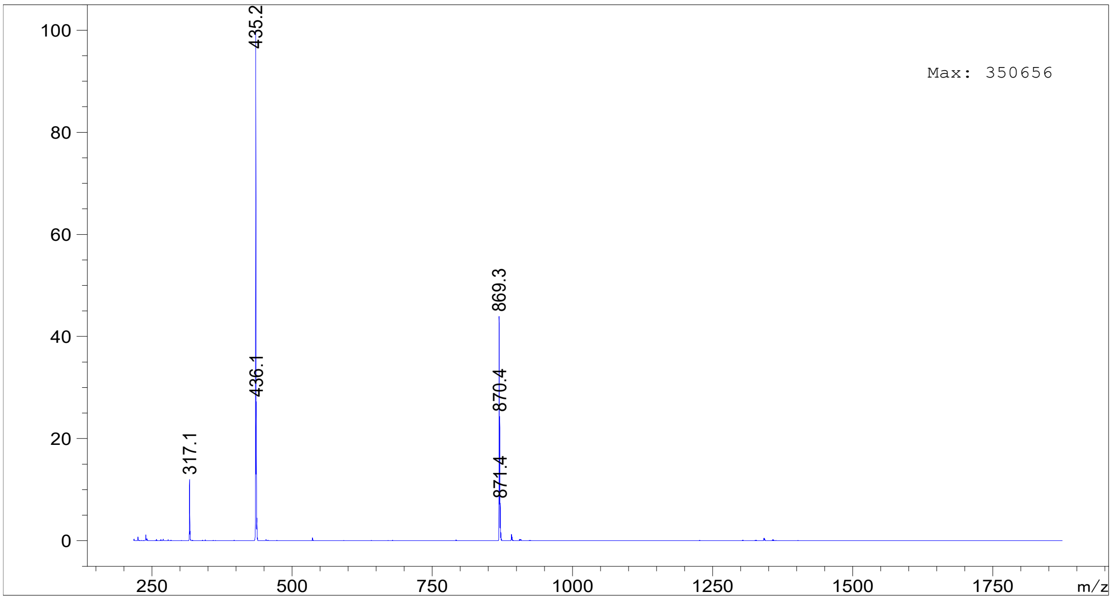

120.Compound **RWVF:** obsvd. [M+ H^+] = 607.2

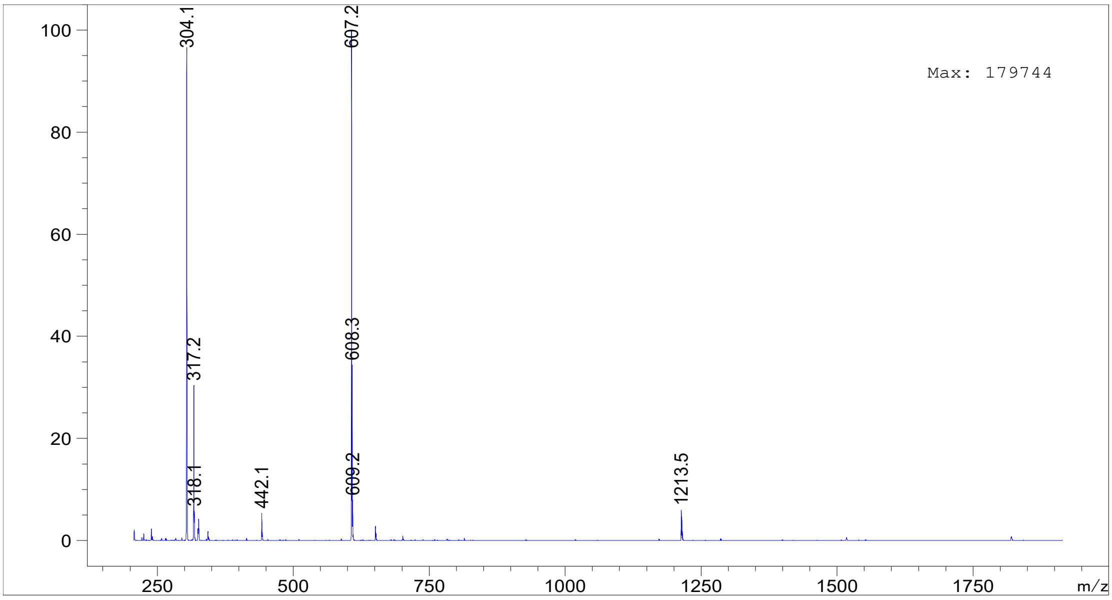

121.Compound **VALV:** obsvd. [M+TFA^-] = 512.6

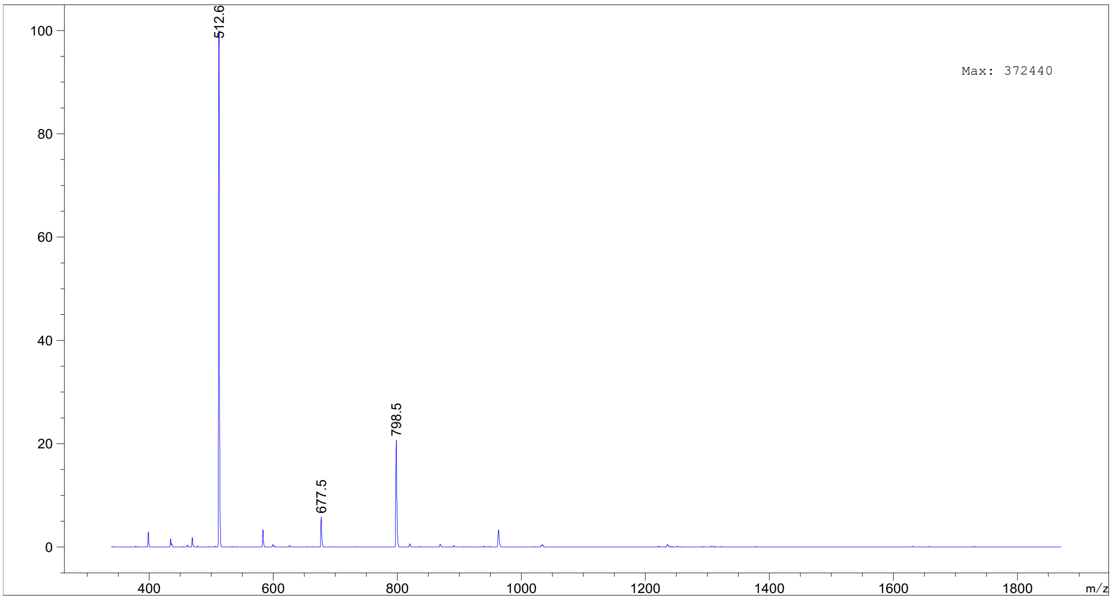

122.Compound **LRFH:** obsvd. [M+ H$^+$] = 572.0

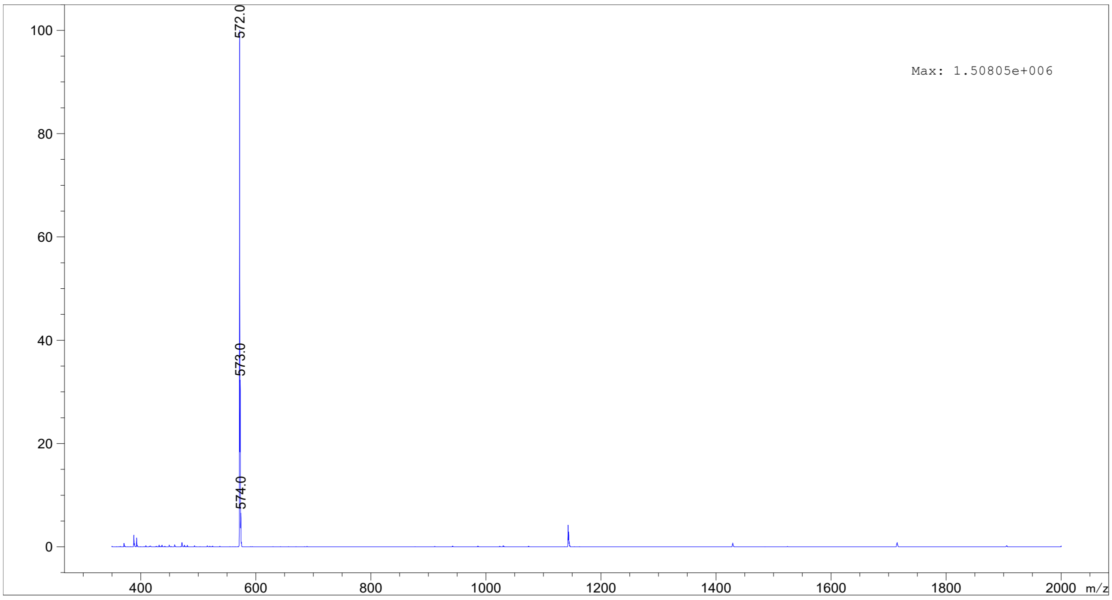

123.Compound **YAYY:** obsvd. [M+ H$^+$] = 578.9

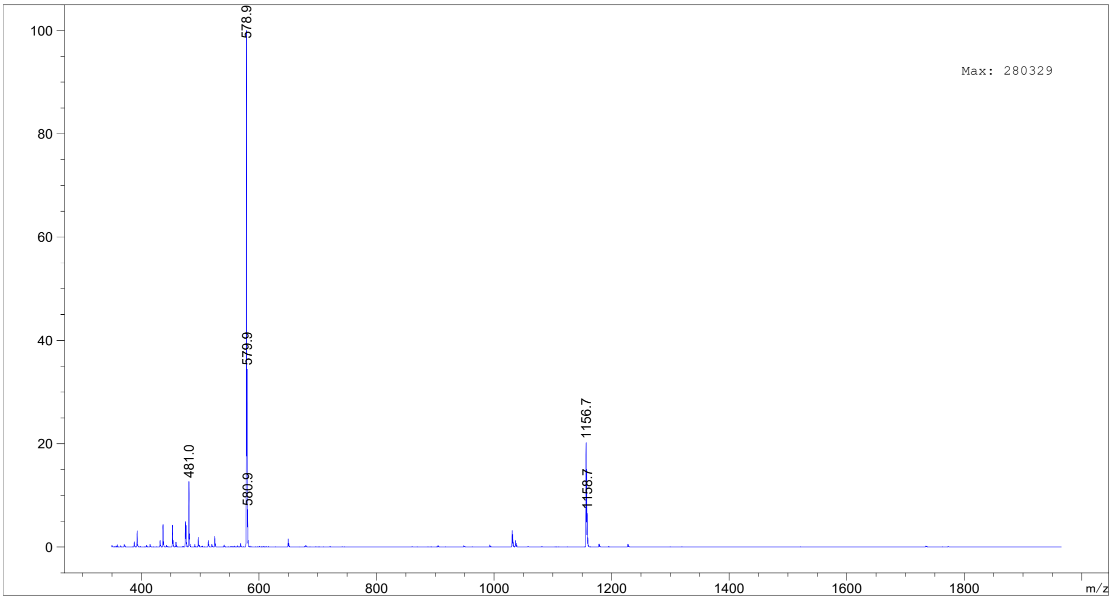

124.Compound **PYYI:** obsvd. [M+ H$^+$] = 555.0

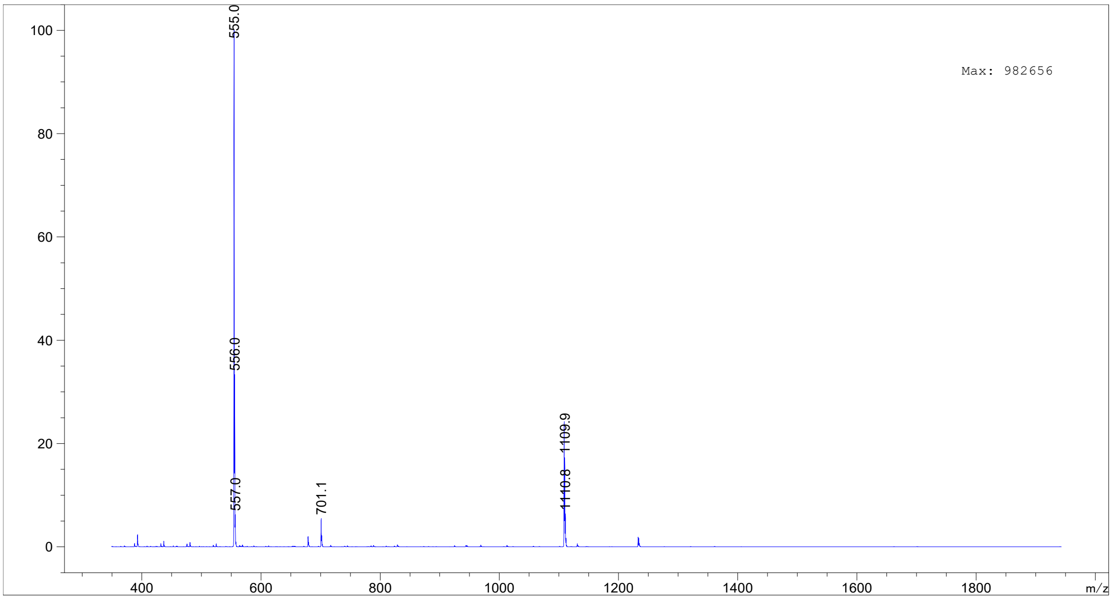

125.Compound **YPYI:** obsvd. [M+ H$^+$] = 555.0

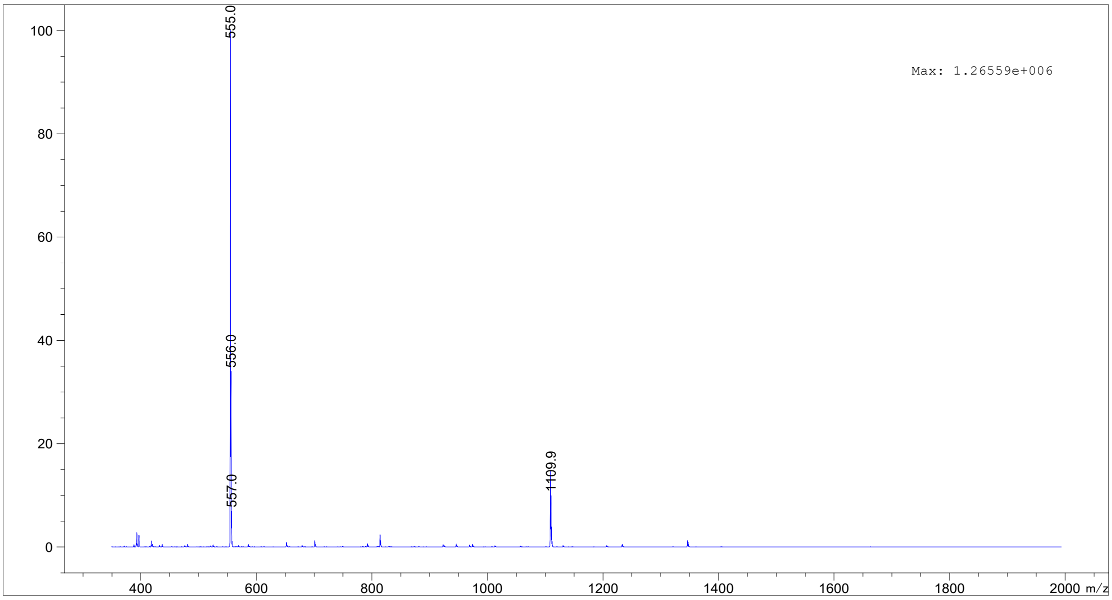

126.Compound **PGWW:** obsvd. [M+ H^{+}] = 545.0

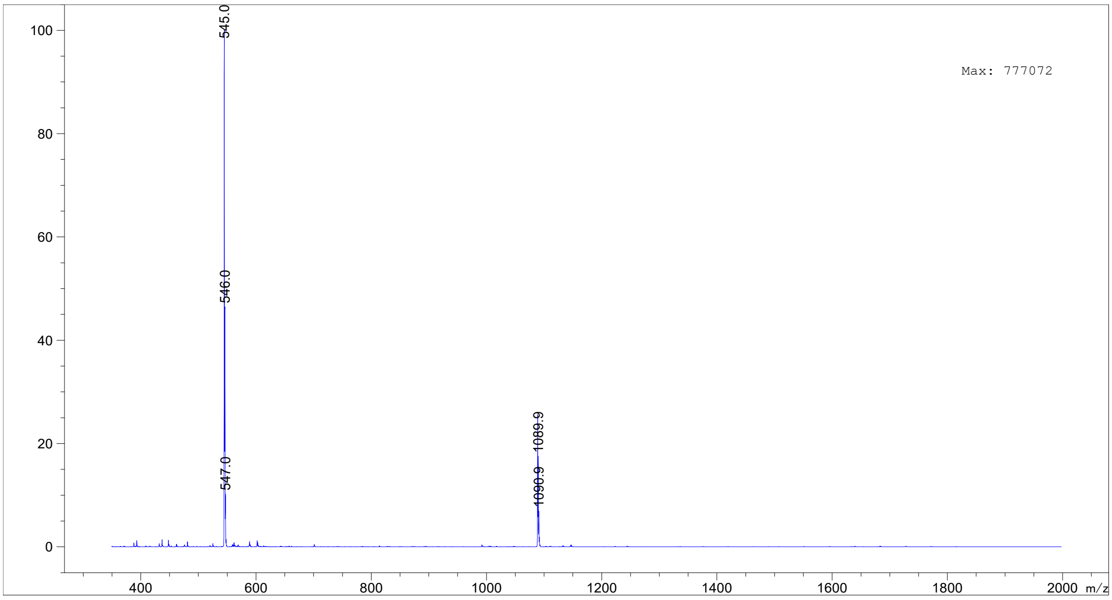

127.Compound **VYTF:** obsvd. [M+ H^{+}] = 529.0

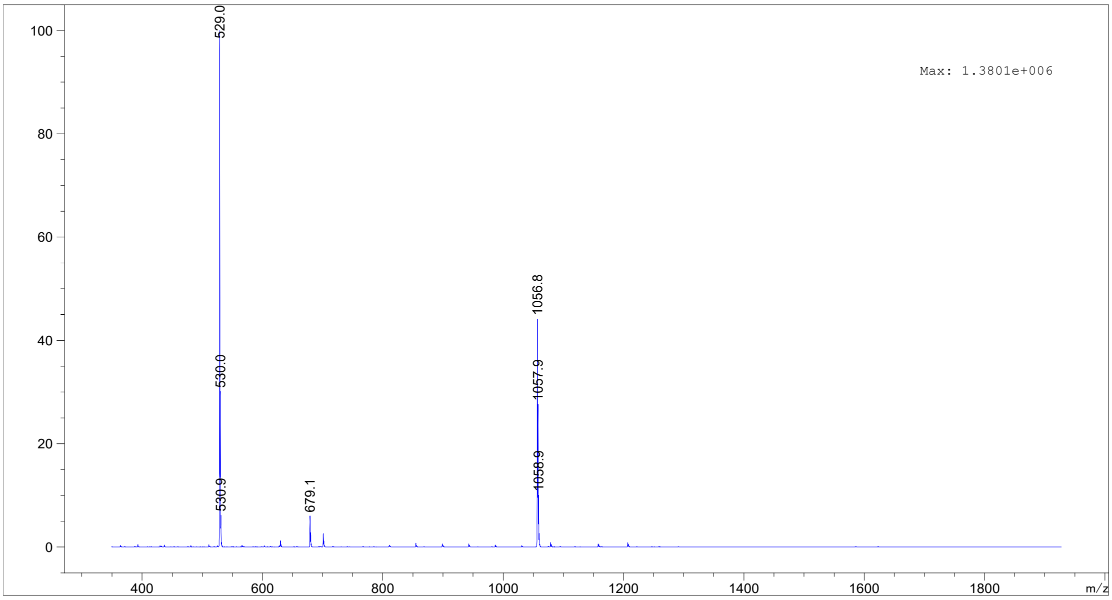

128.Compound **LHII:** obsvd. [M+ H^+] = 495.1

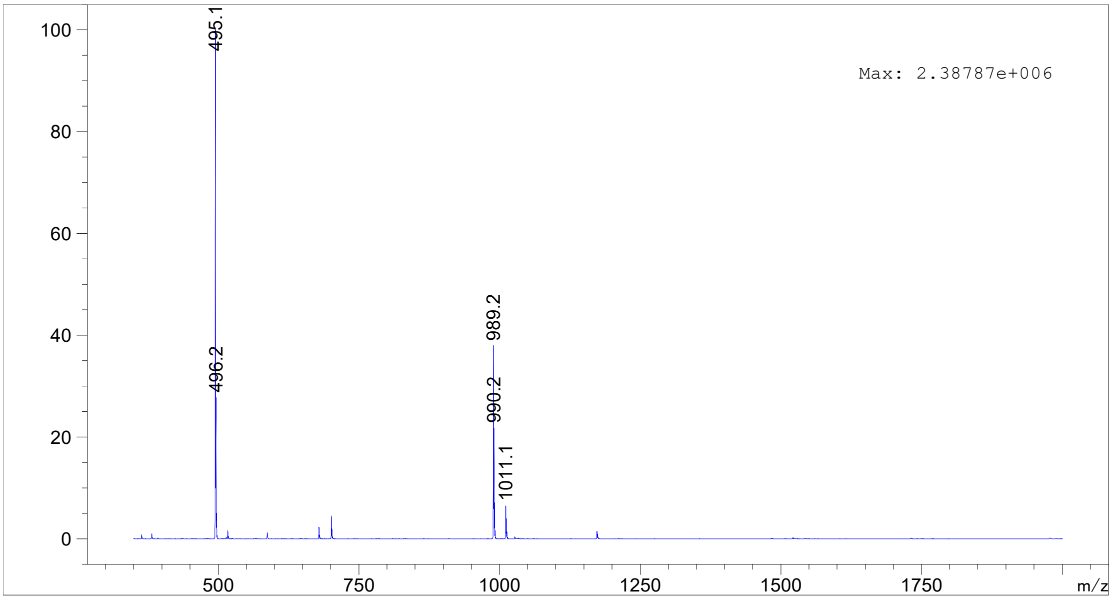

129.Compound **QHII:** obsvd. [M+ H^+] = 510.1

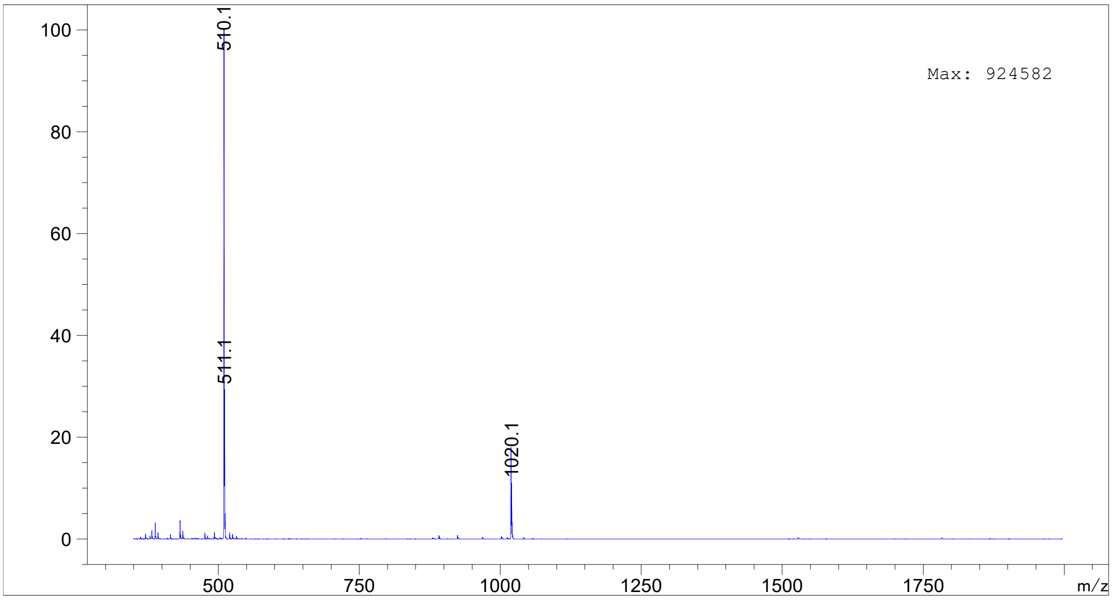

130.Compound **LTWF:** obsvd. $[M+ H^{+}]$ = 566.0

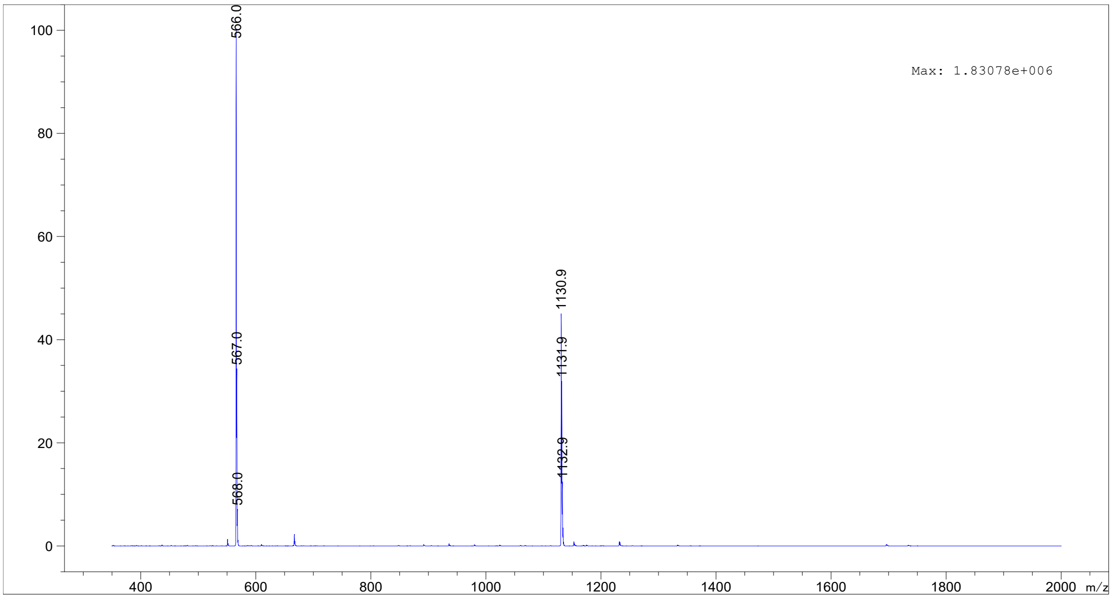

131.Compound **LWFI:** obsvd. $[M+ H^{+}]$ = 578.0

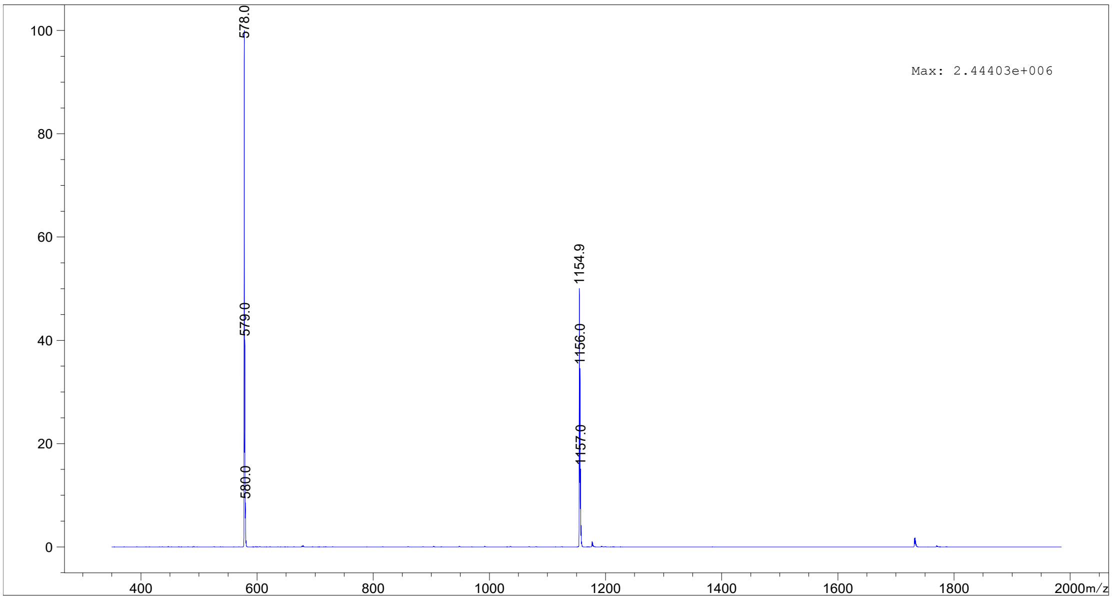

132.Compound **IHFV:** obsvd. [M+ H^+] = 515.3

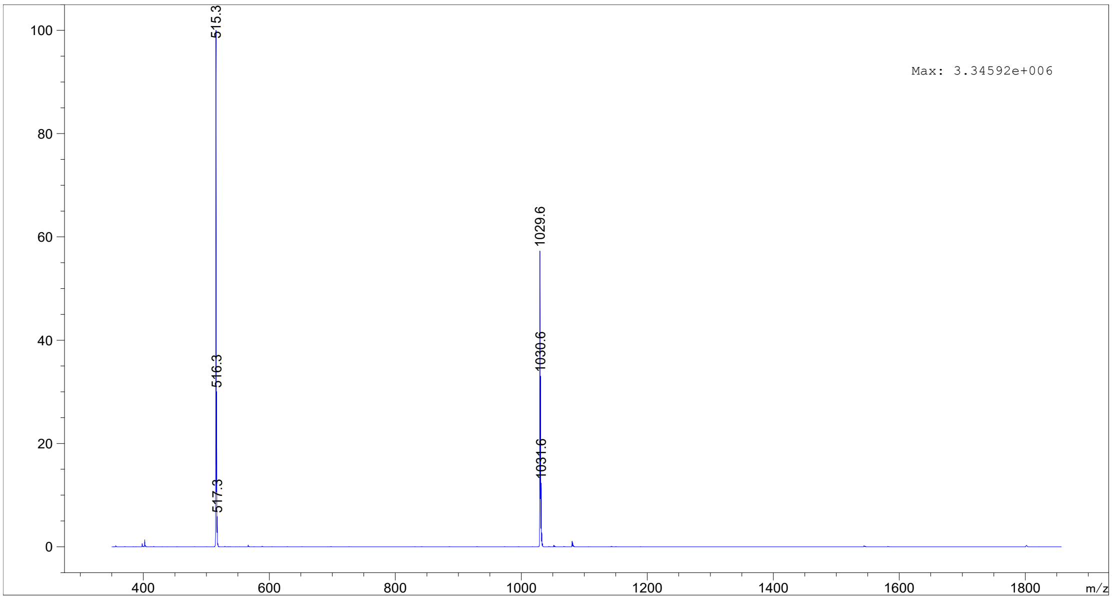

133.Compound **LVPF:** obsvd. [M+ H^+] = 475.3

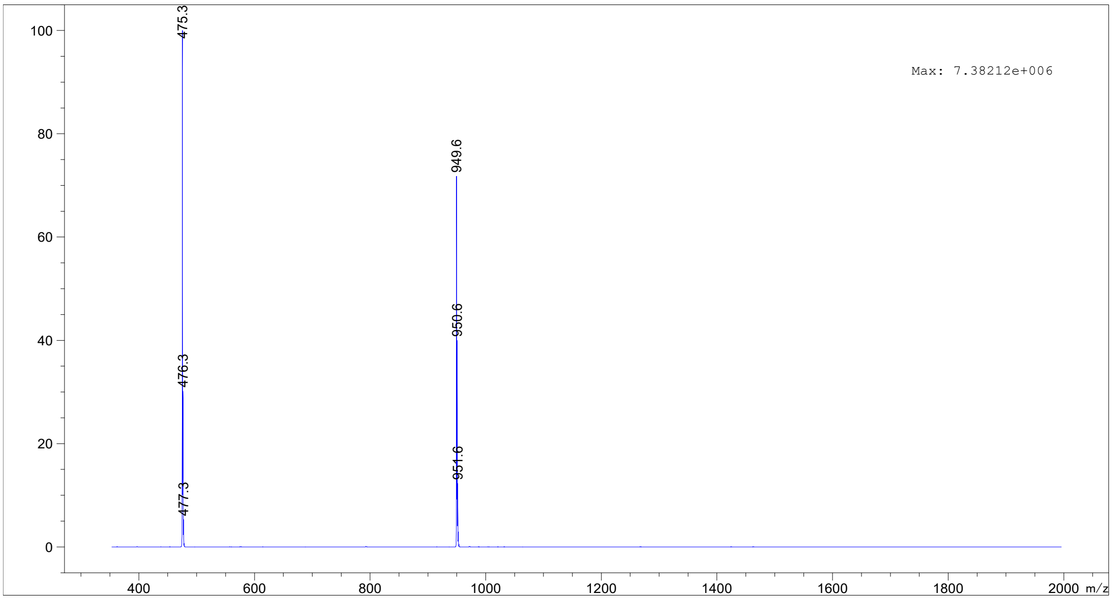

134.Compound **FMYS:** obsvd. [M+ H$^+$] = 547.0

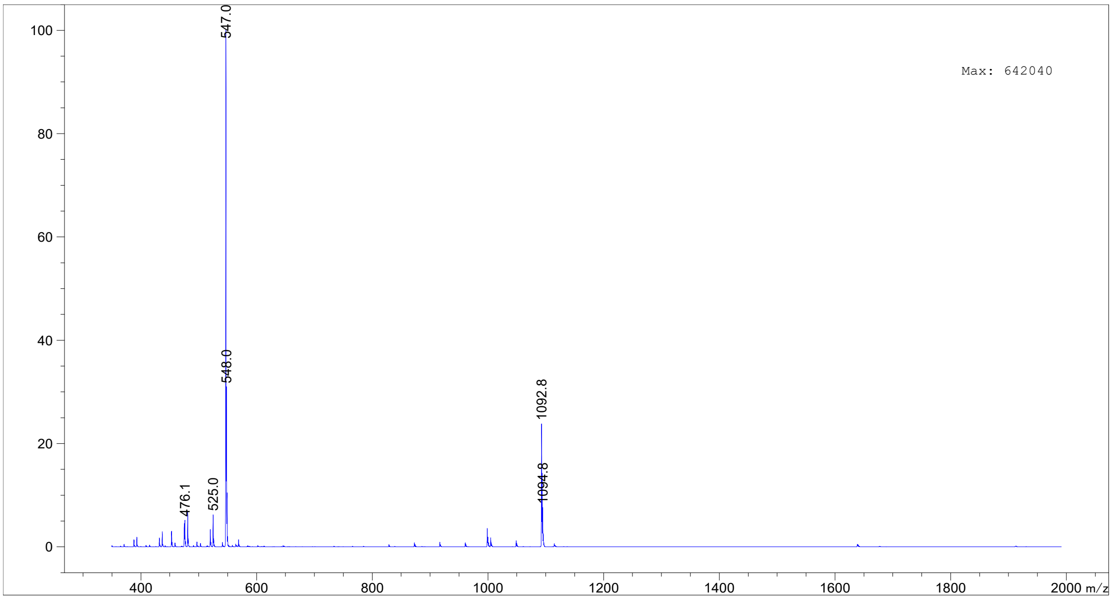

135.Compound **KFLL:** obsvd. [M+ H$^+$] = 520.1

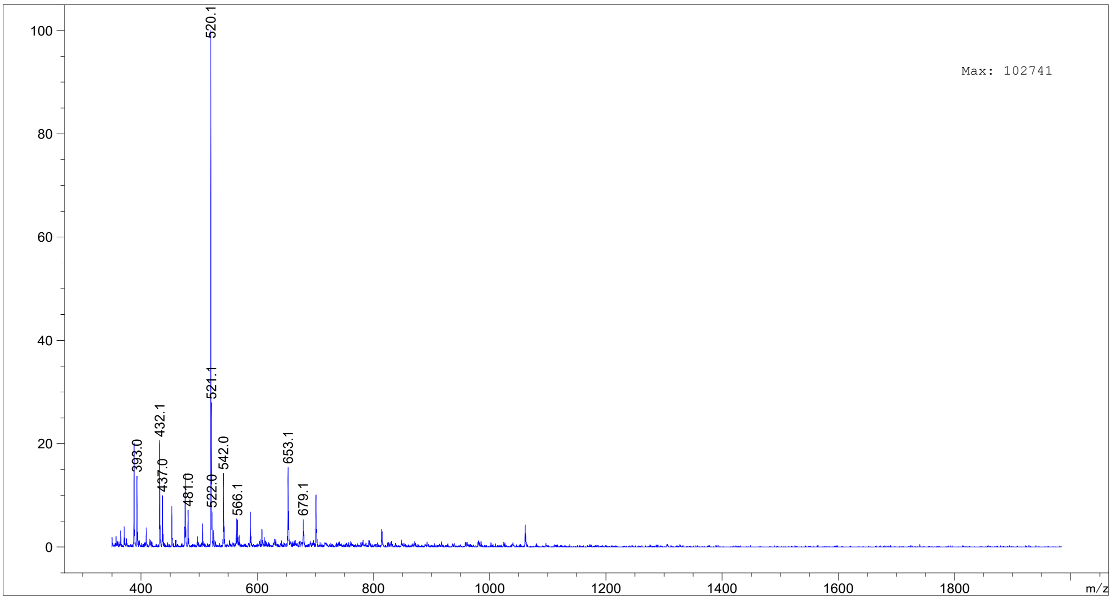

136.Compound **VFGV:** obsvd. [M+ H^+] = 421.1

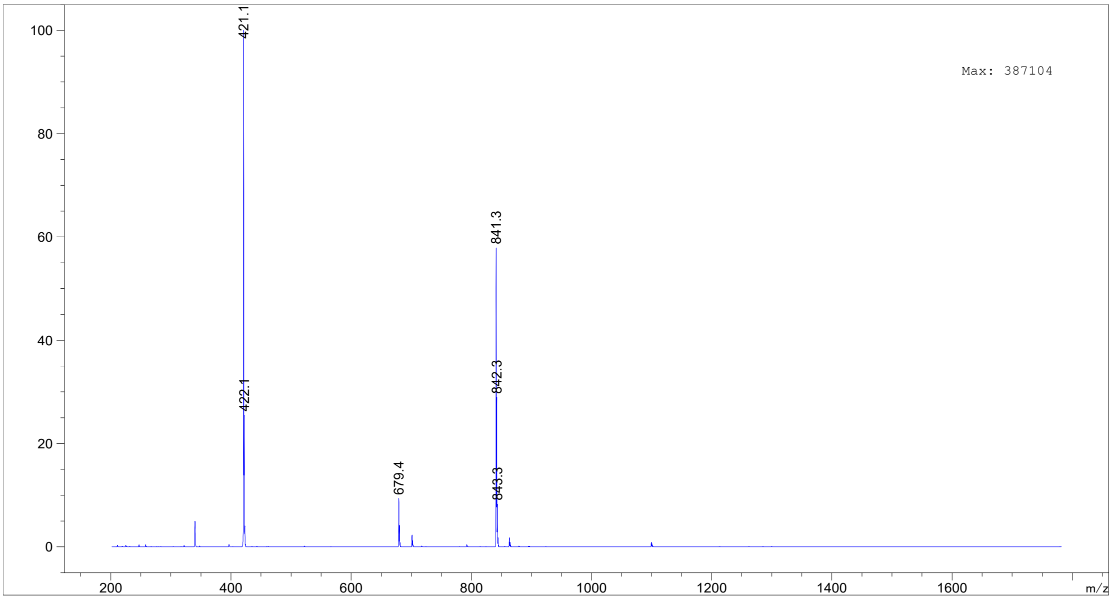

137.Compound **CCCC:** obsvd. [M+ H^+] = 431.1

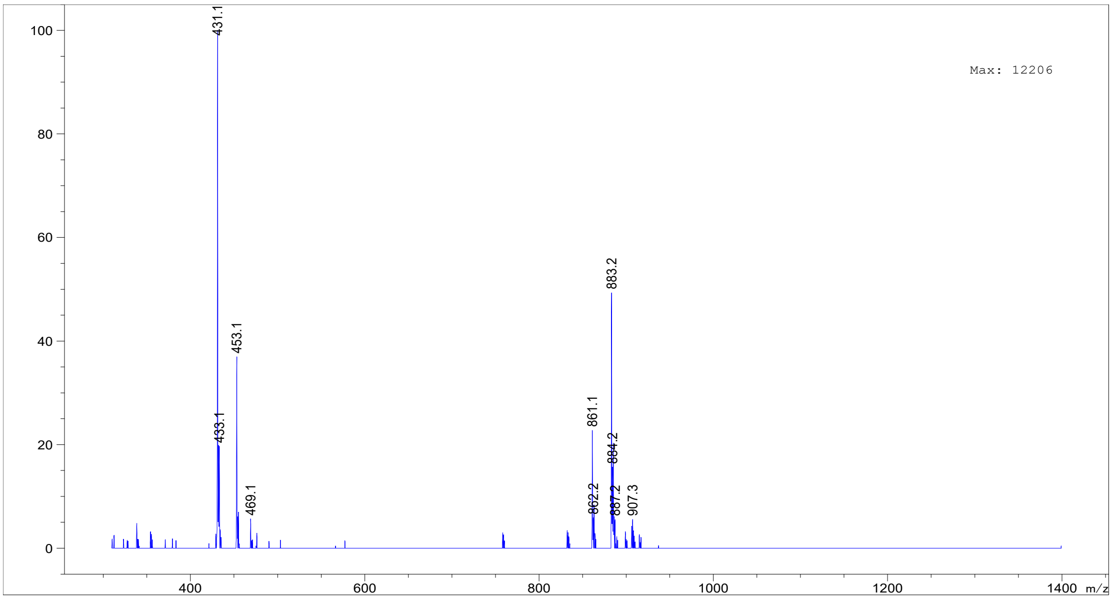

138.Compound **EEEE:** obsvd. [M+ H$^+$] = 535.3

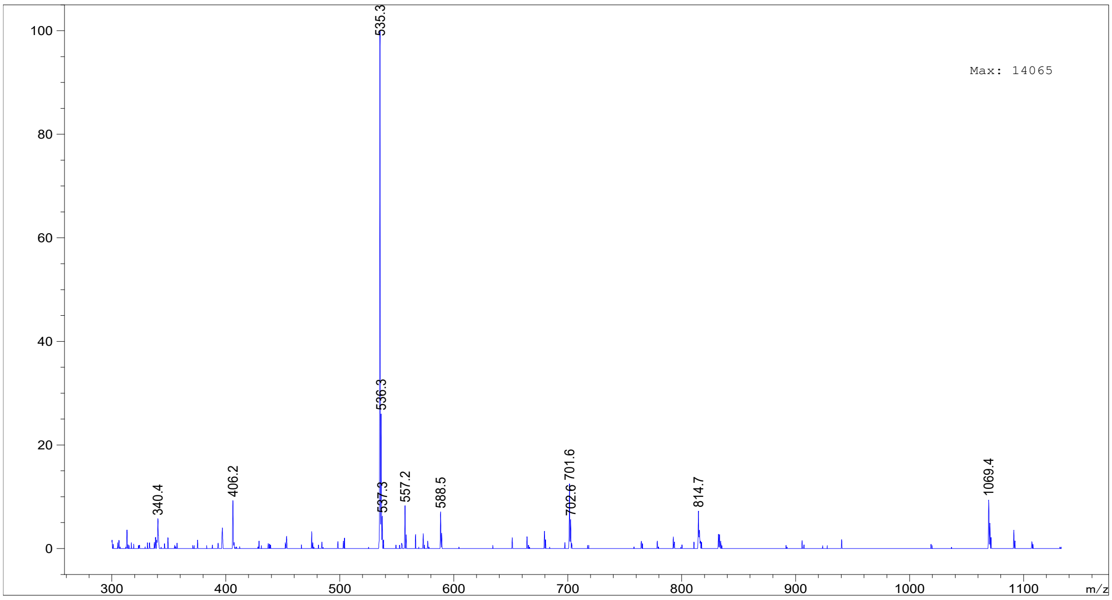

139.Compound **PPPP:** obsvd. [M+ H$^+$] = 407.2

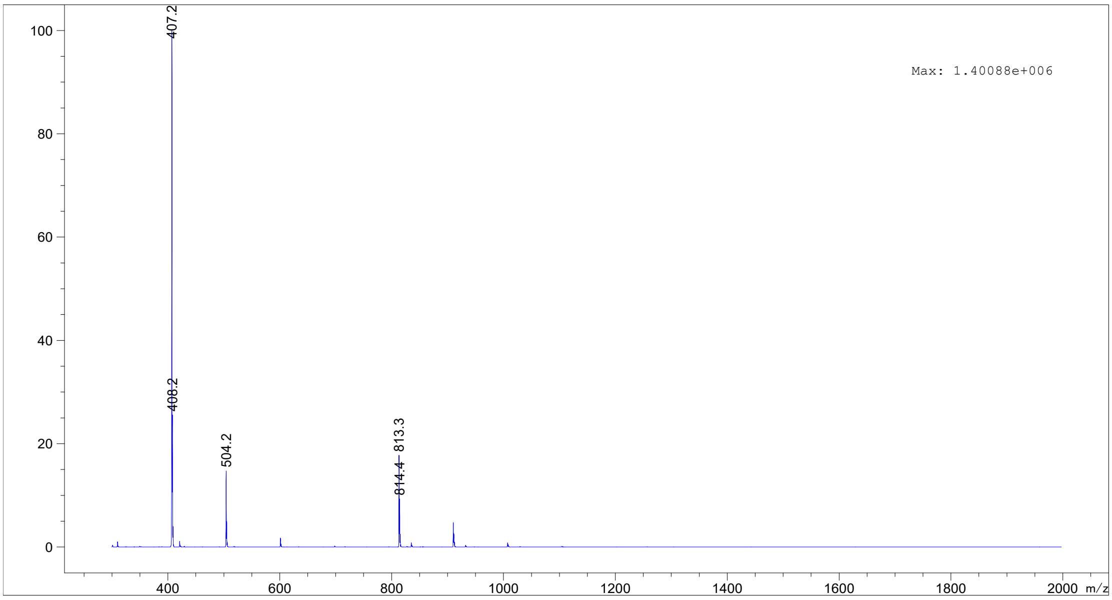

140.Compound **QQQQ:** obsvd. [M+ H^+] = 531.2

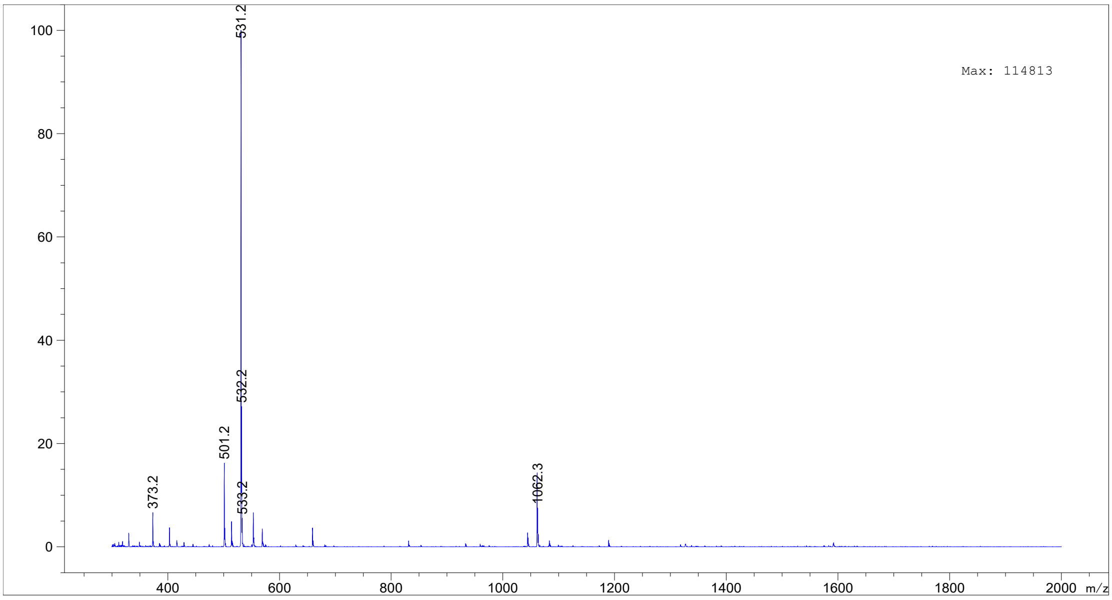

141.Compound **SSSS:** obsvd. [M+ H^+] = 367.2

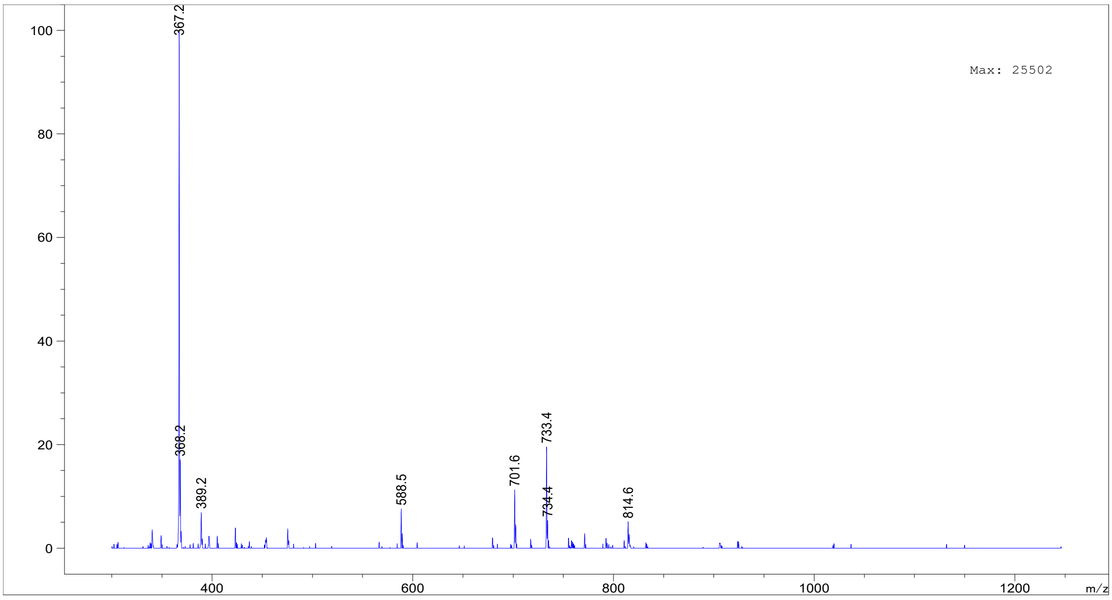

142.Compound **TTTT:** obsvd. [M+ H^+] = 423.4

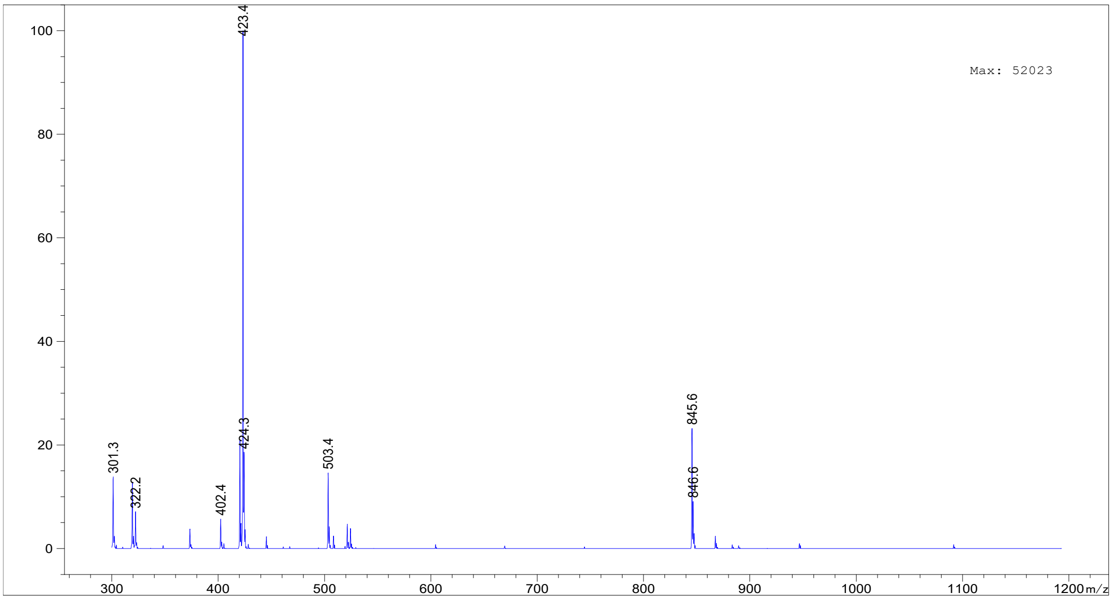

143.Compound **WWWW:** obsvd. [M+ H^+] = 763.5

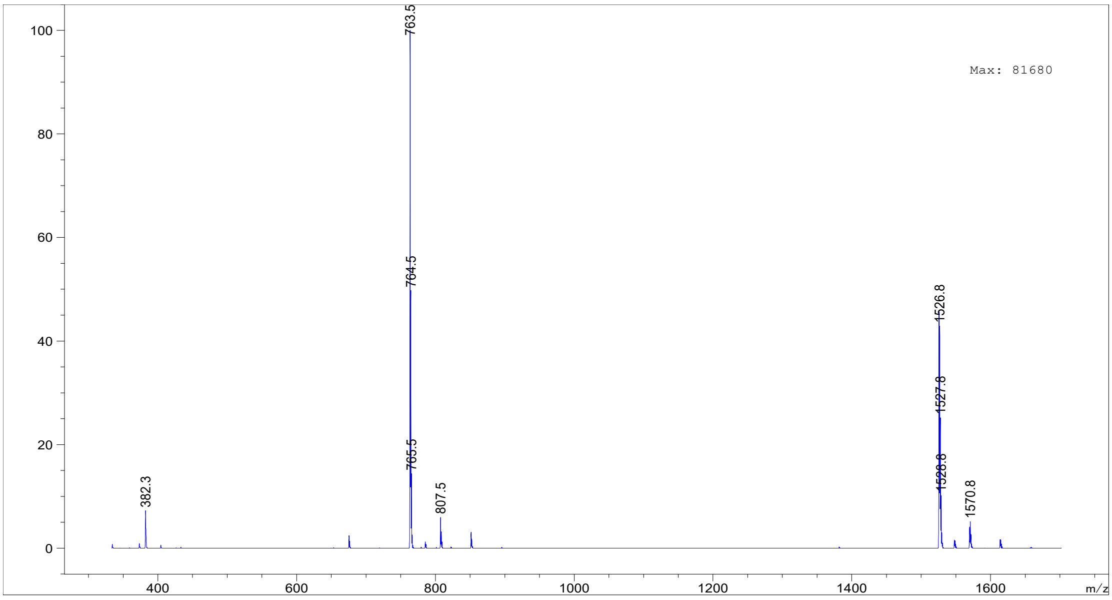

144.Compound **CWFT:** obsvd. [M+ H^+] = 555.9

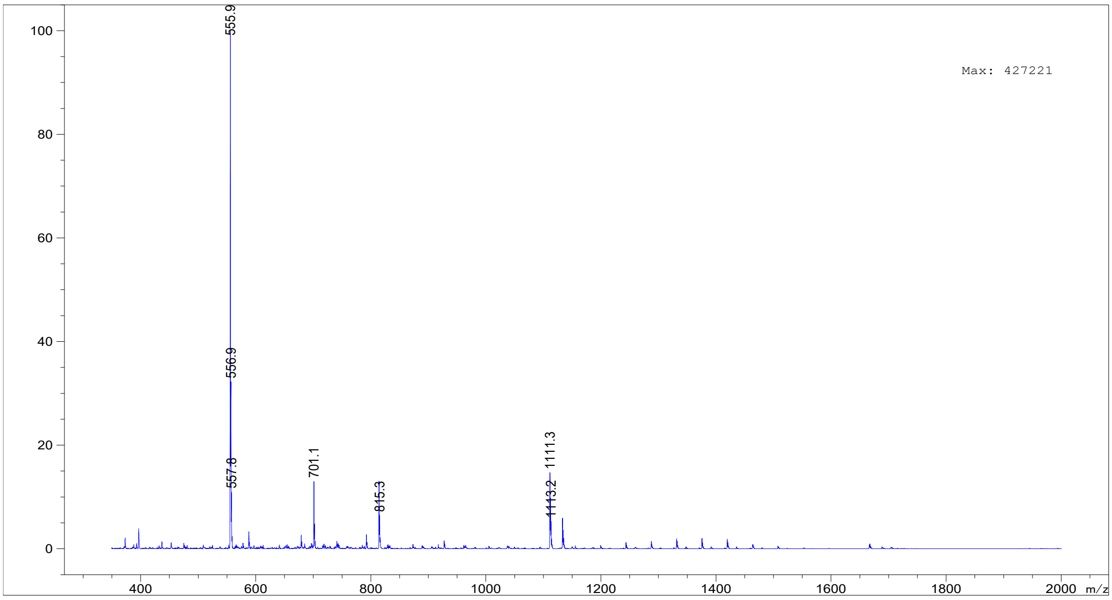

145.Compound **IRVW:** obsvd. [M+ H^+] = 572.9

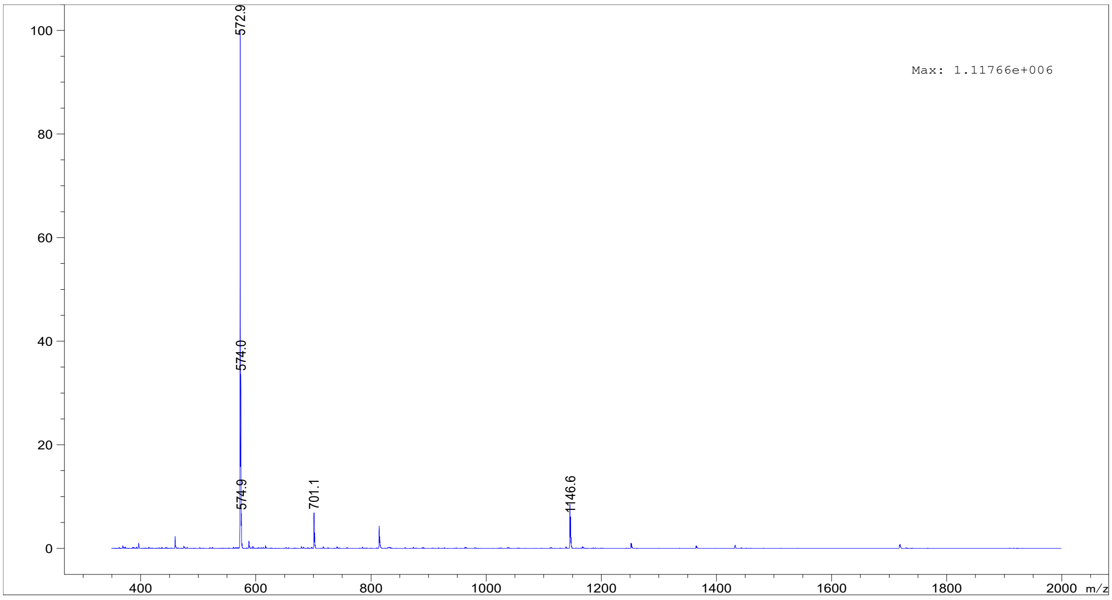

146.Compound **NVFH:** obsvd. $[M+H^{+}] = 516.2$

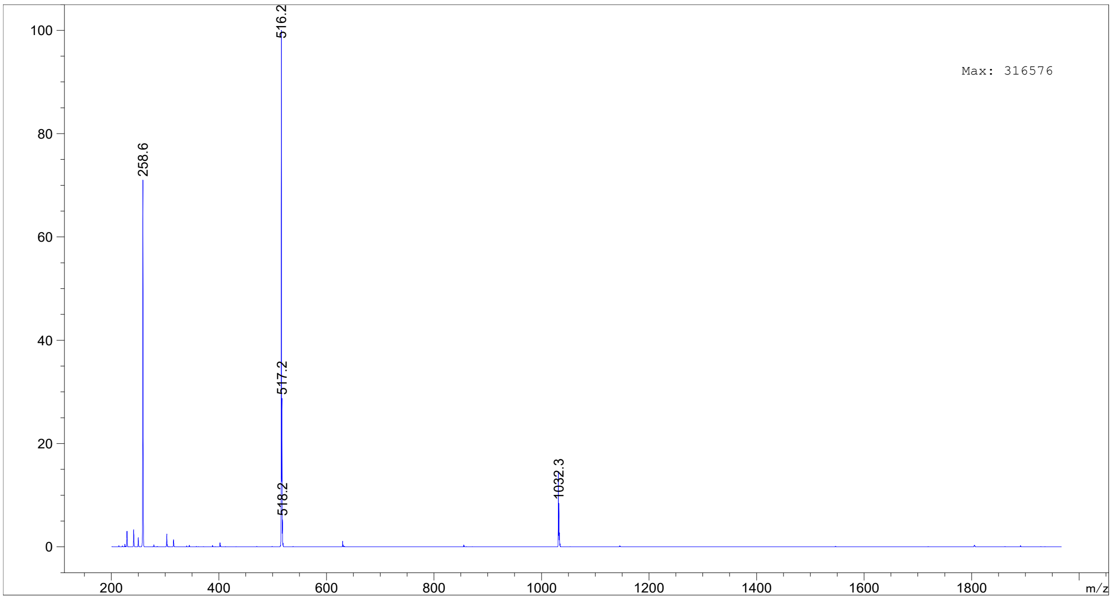

147.Compound **VFHN:** obsvd. $[M+H^{+}] = 516.2$

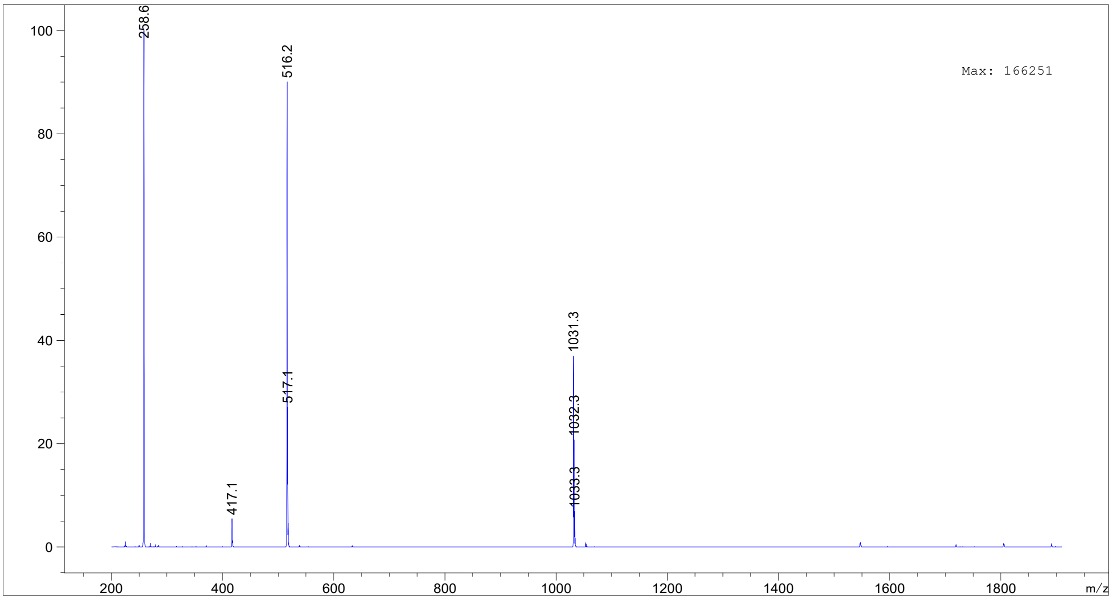

148.Compound **CFLW:** obsvd. [M+ H^+] = 567.9

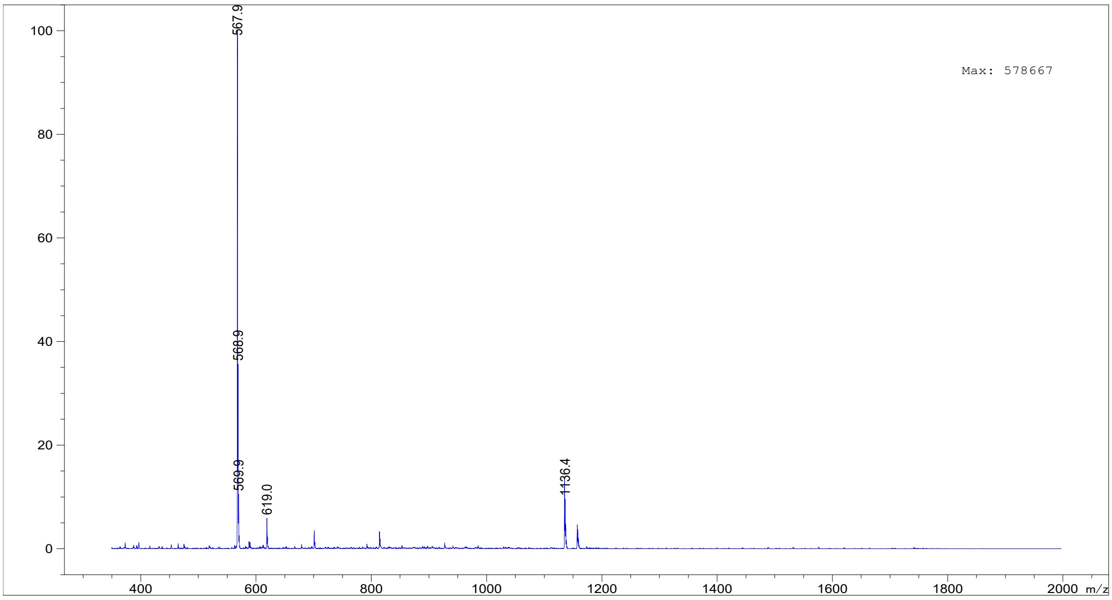

149.Compound **MMYY:** obsvd. [M+ H^+] = 606.9

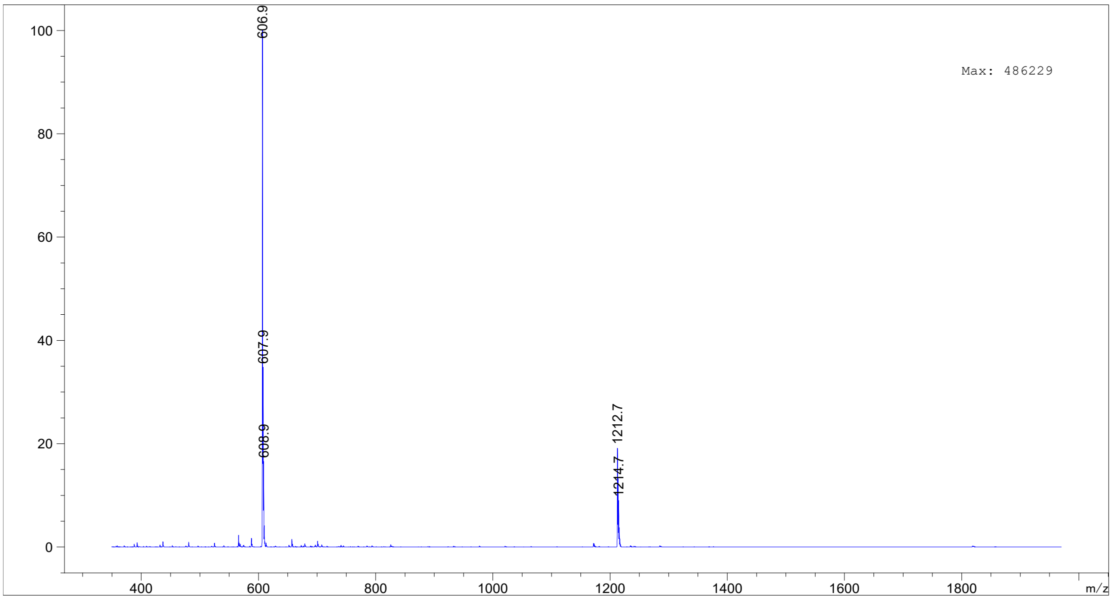

150.Compound **GLLY:** obsvd. [M+ H$^+$] = 464.5

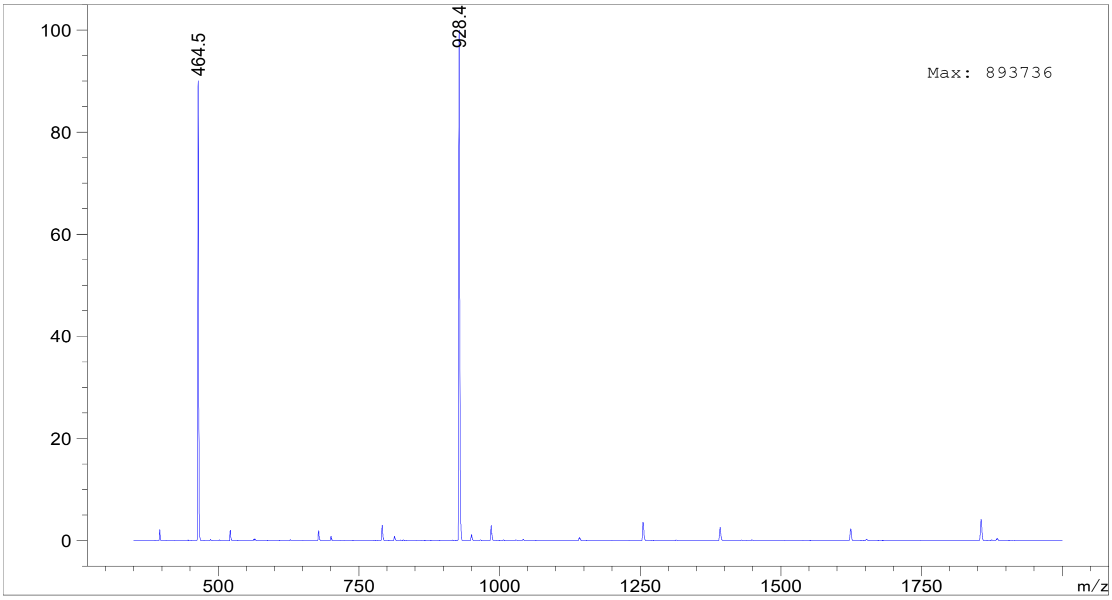

151.Compound **KMLF:** obsvd. [M+ H$^+$] = 538.0

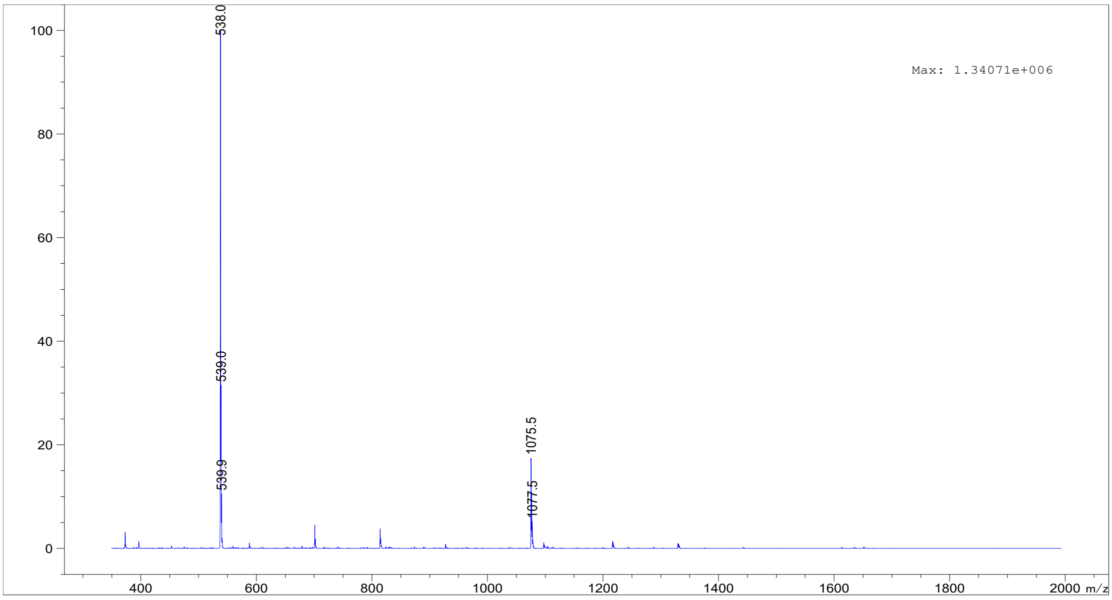

152.Compound **IWII:** obsvd. [M+ H^{+}] = 544.0

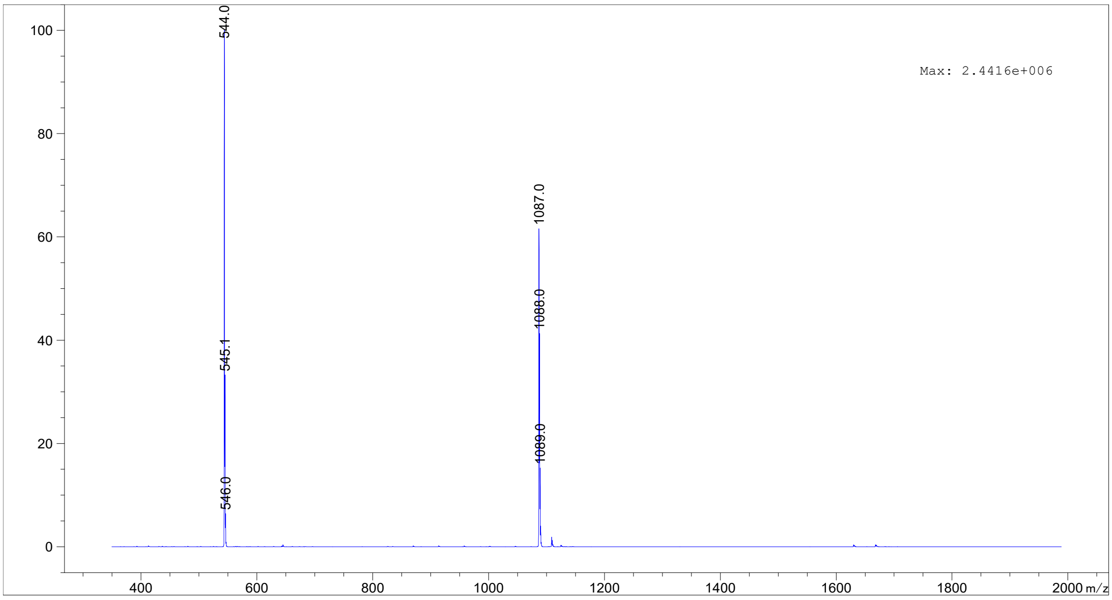

153.Compound **WVFI:** obsvd. [M+ H^{+}] = 564.0

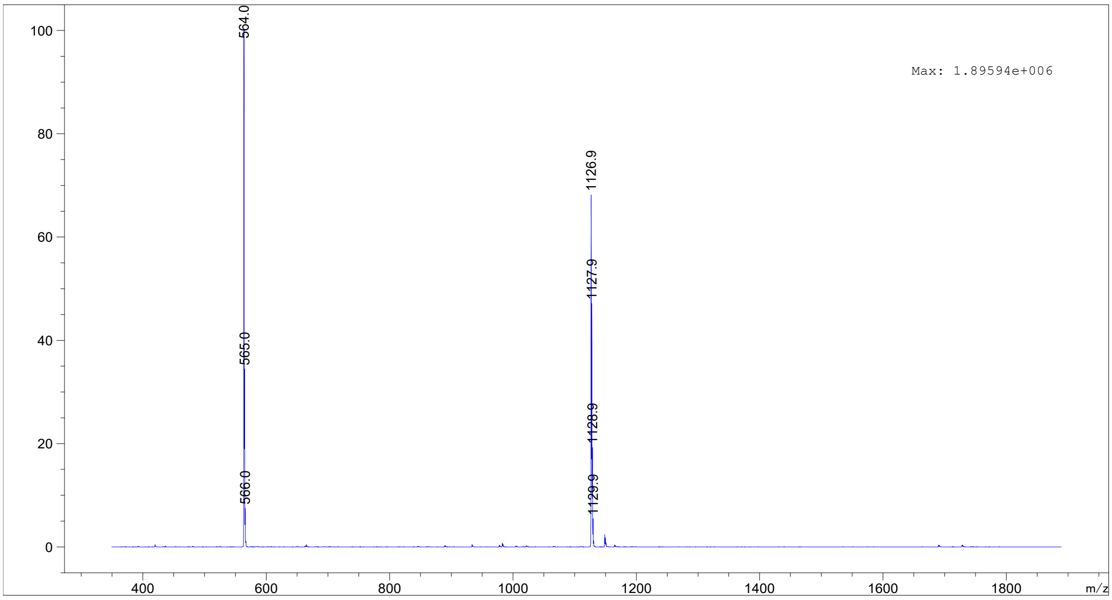

154.Compound **IIIR:** obsvd. [M+ H^+] = 514.4

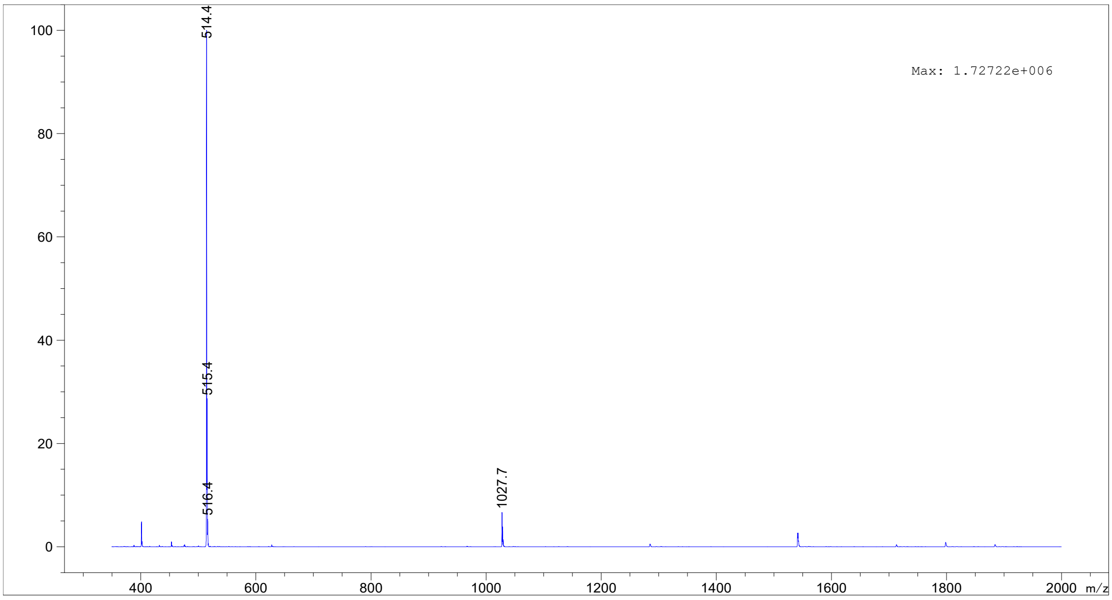

155.Compound **AVIQ:** obsvd. [M+ H^+] = 429.9

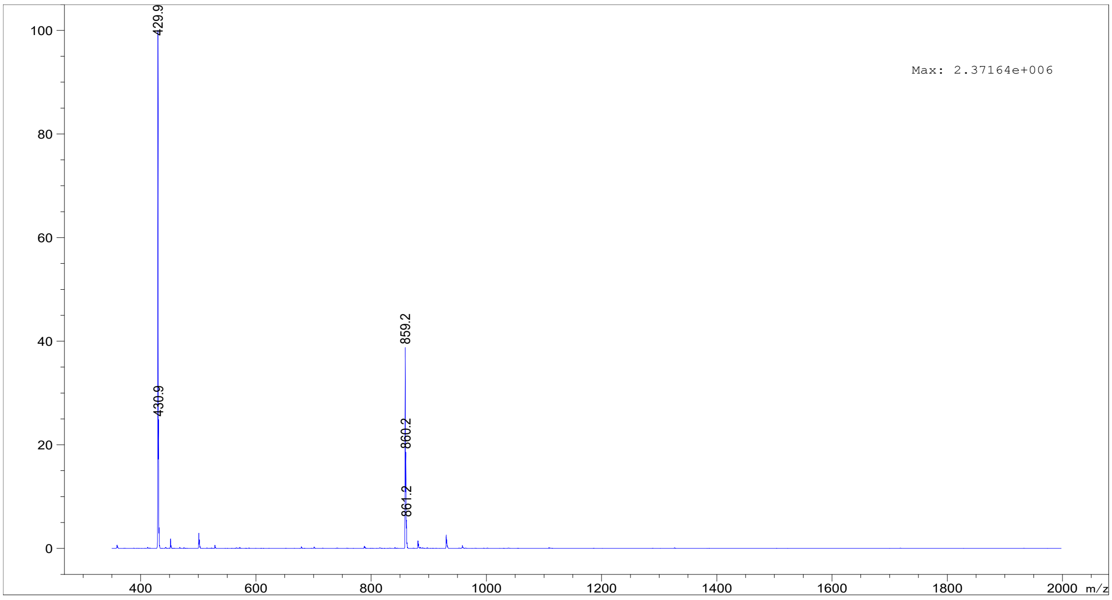

156.Compound **DALV:** obsvd. [M+ H$^+$] = 416.9

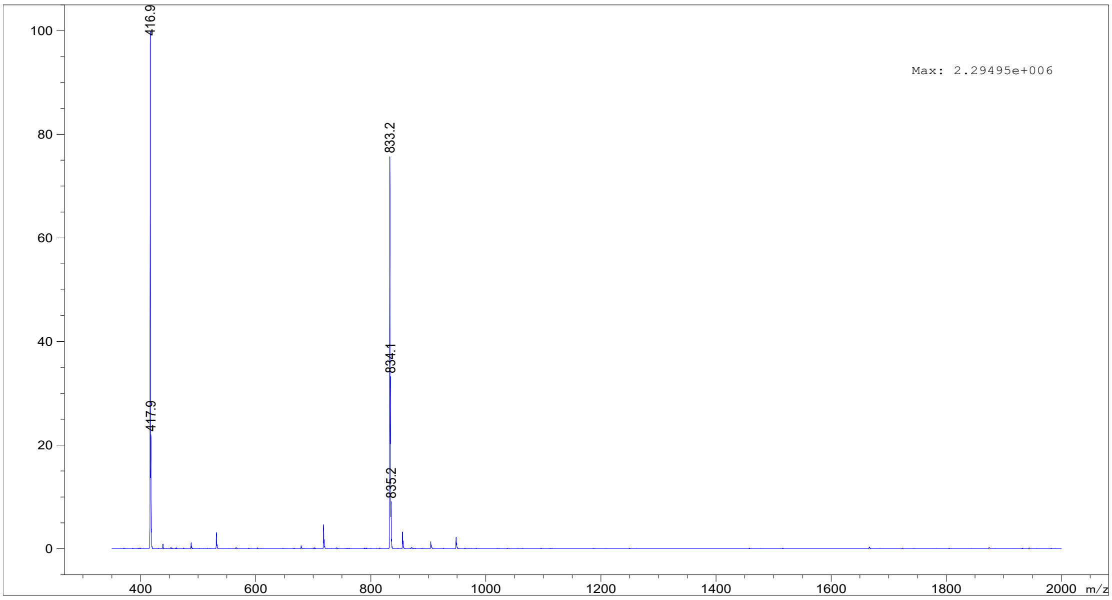

157.Compound **GVNM:** obsvd. [M+ H$^+$] = 419.9

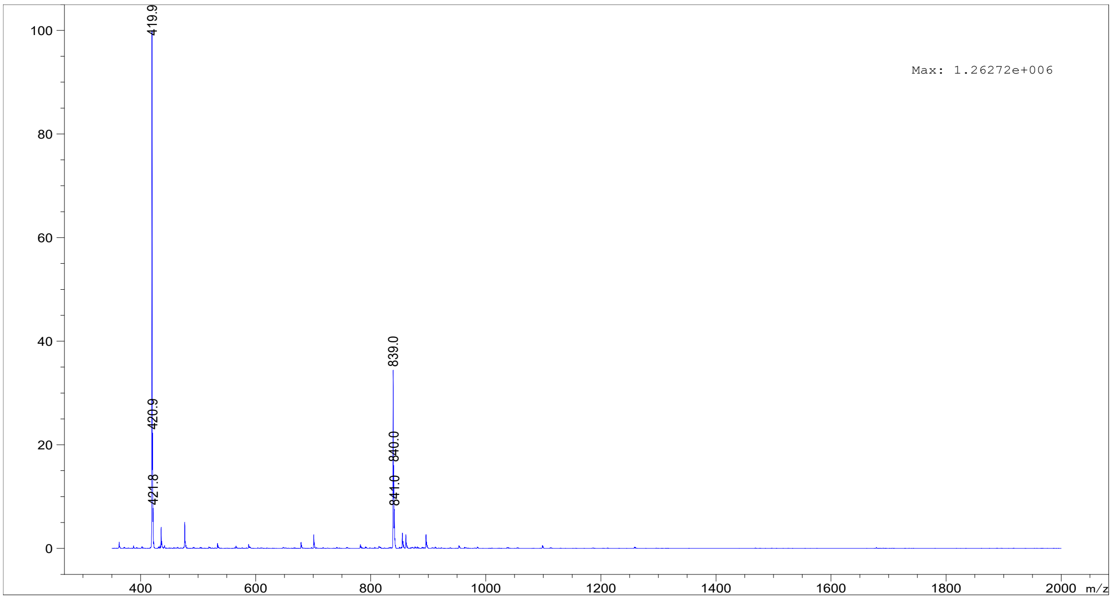

158.Compound **LALR:** obsvd. $[M+ H^{+}] = 472.0$

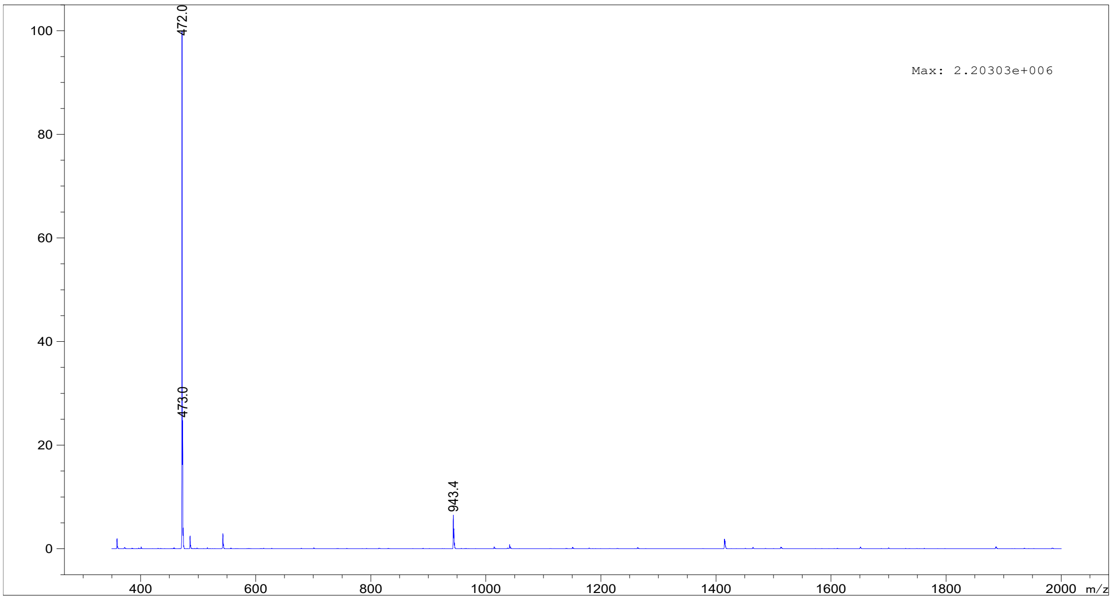

159.Compound **LLRQ:** obsvd. $[M+ H^{+}] = 528.9$

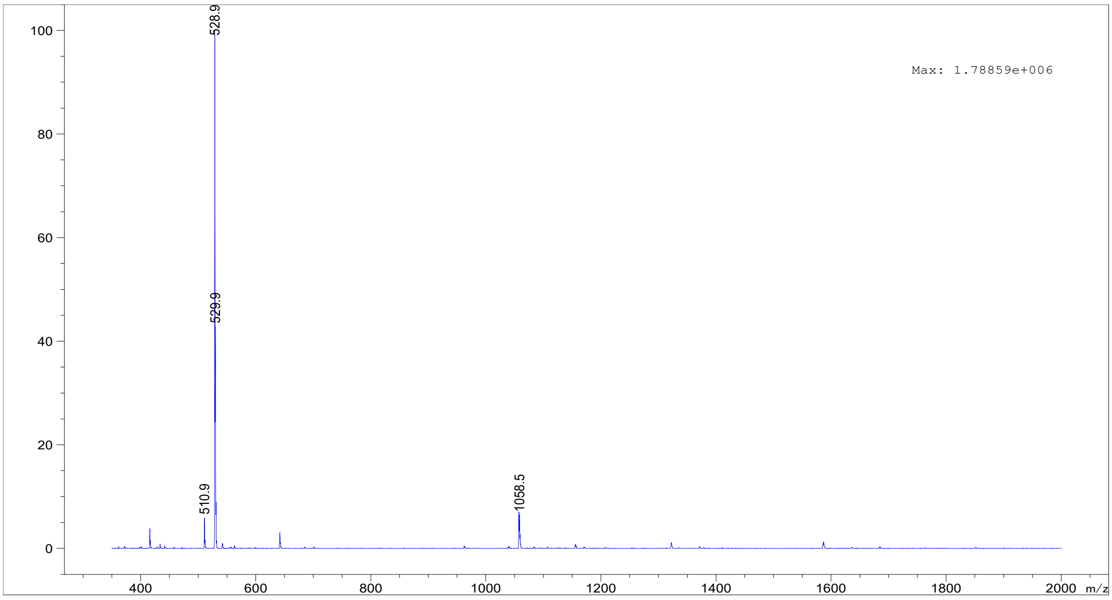

160.Compound **MVQG:** obsvd. [M+ H$^+$] = 433.9

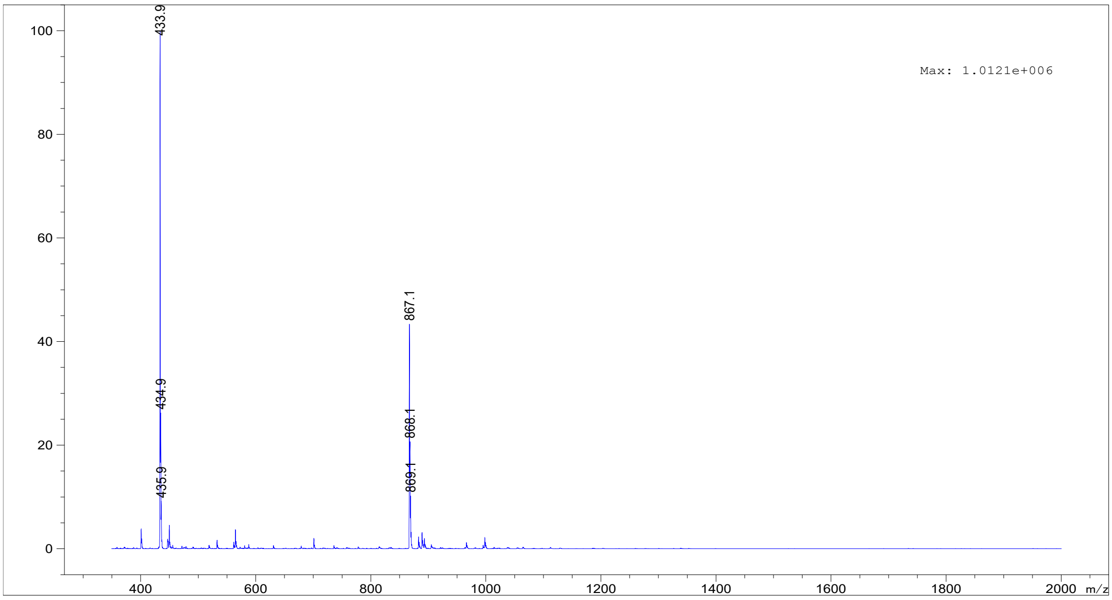

161.Compound **VALE:** obsvd. [M+ H$^+$] = 430.9

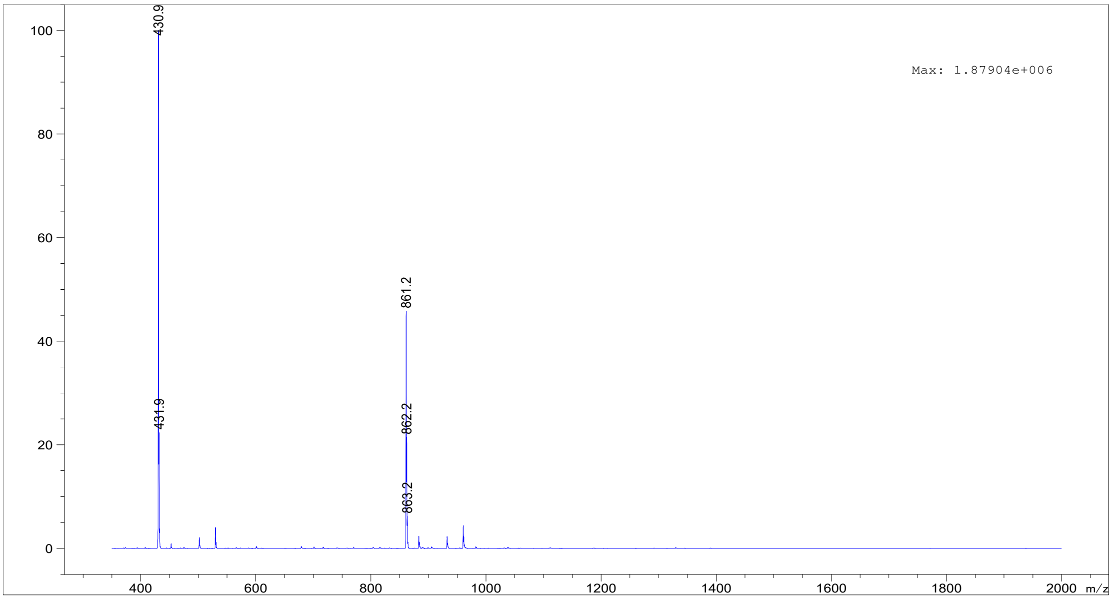

162.Compound **FAFP:** obsvd. [M+ H^+] = 481.0

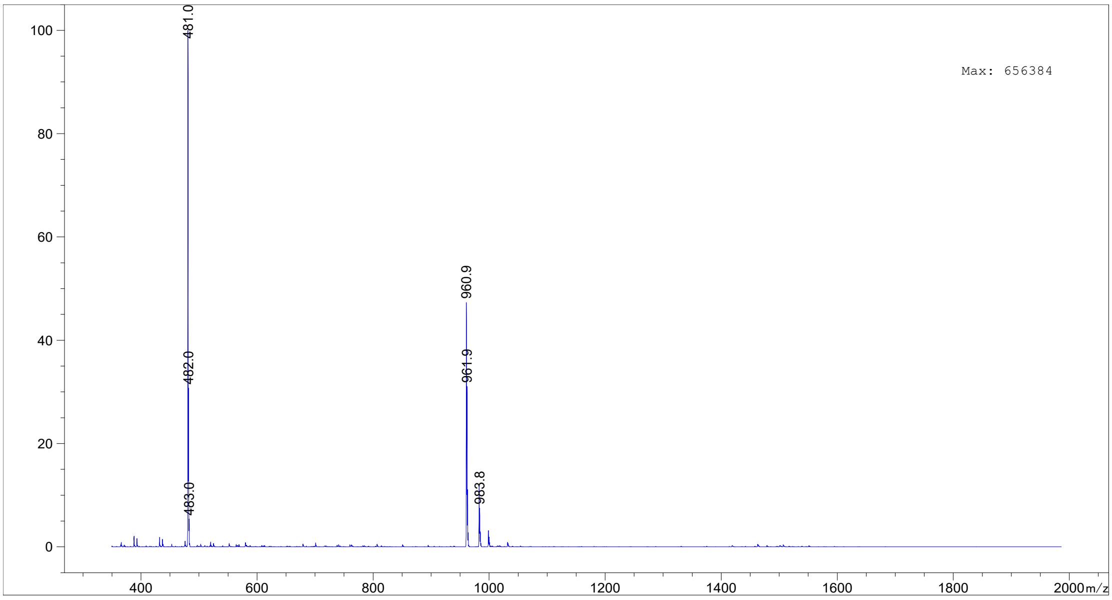

163.Compound **PWWC:** obsvd. [M+ H^+] = 590.8

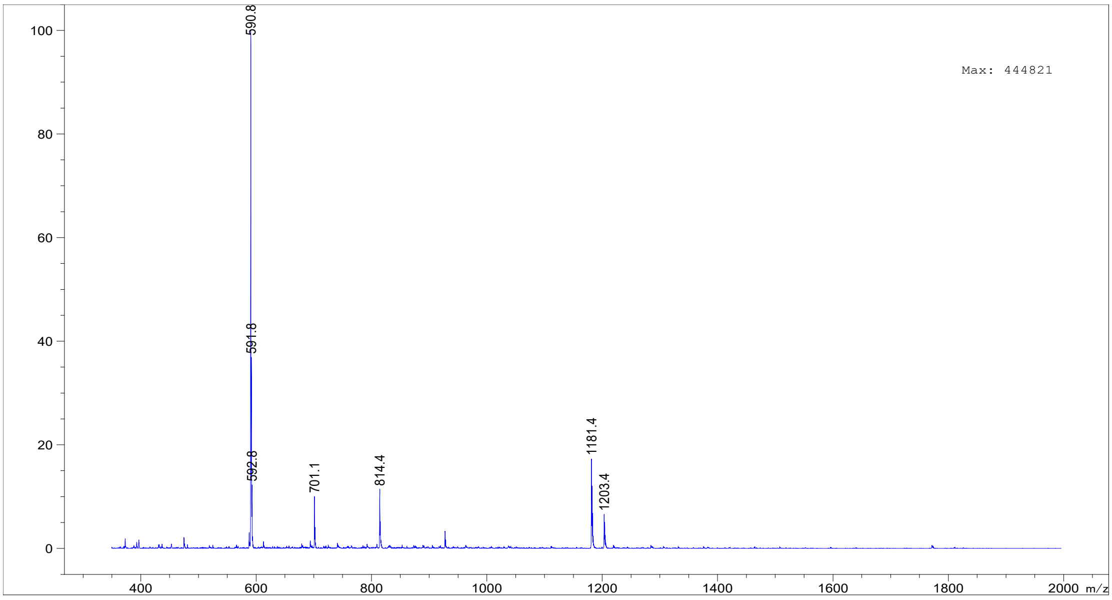

164.Compound **TRFS:** obsvd. [M+ H^+] = 509.9

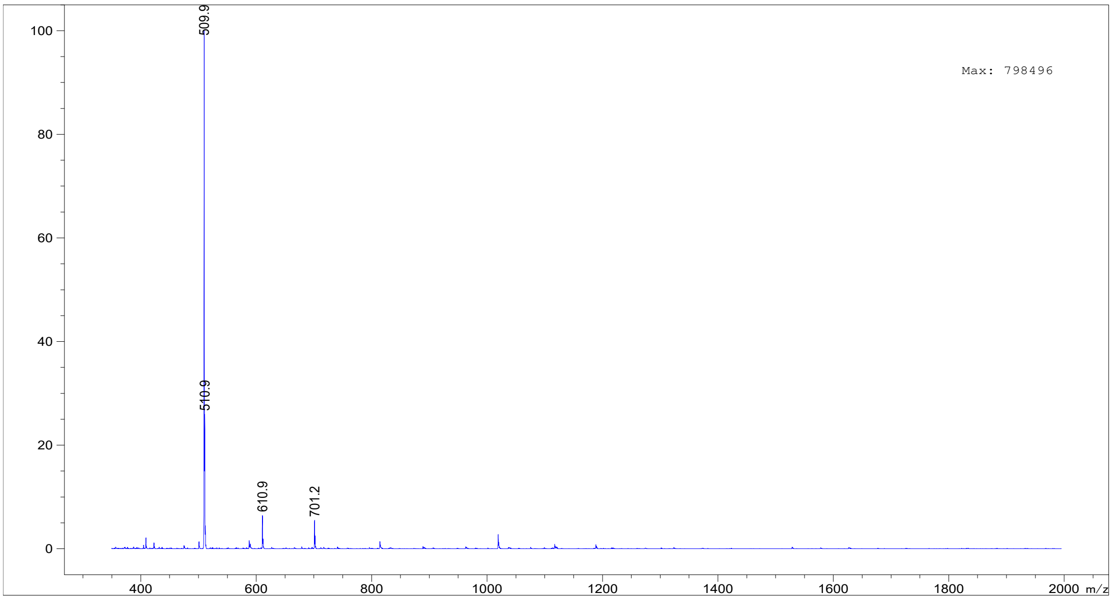

165.Compound **NNNN:** obsvd. [M+ H^+] = 475.3

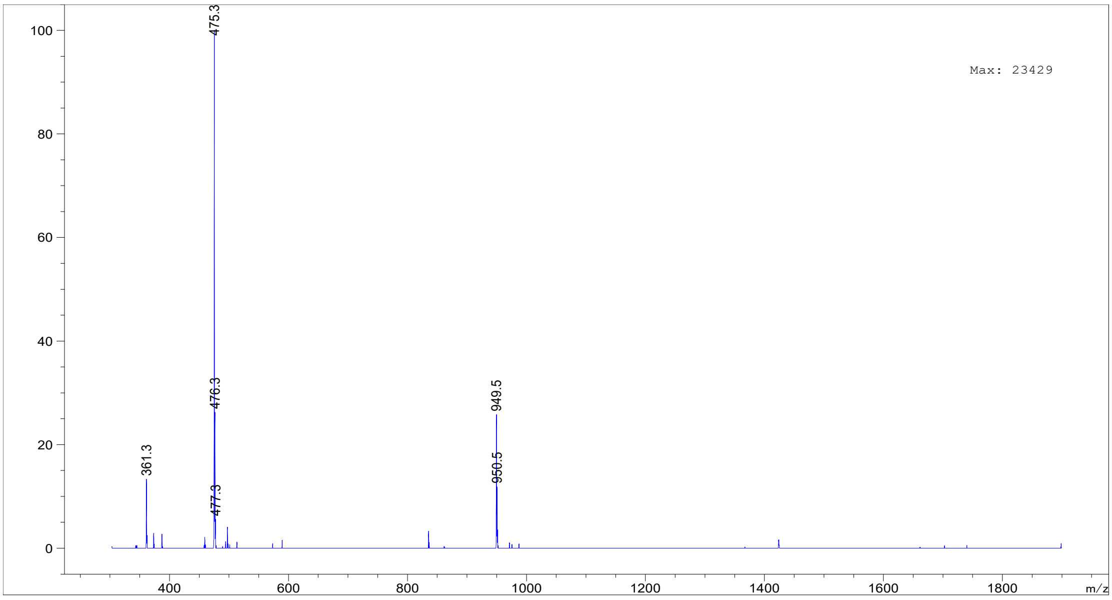

475.3
476.3
477.3
361.3
949.5
950.5
Max: 23429
m/z
