# Supplementary material for: Accelerating the prediction and discovery of peptide hydrogels with human-in-the-loop
Source: Nat Commun. 2023 Jun 30;14:3880. doi: 10.1038/s41467-023-39648-2 (PMC10313671; doi:10.1038/s41467-023-39648-2)
Supplement: Supplementary file 8 — Supplementary Data 5 [file 41467_2023_39648_MOESM8_ESM.pdf]

1.Compound **LWFW**: ^{1}H NMR (500 MHZ, DMSO-d6) δ 10.86 (d, J = 2.4 Hz, 1H), 10.78 (d, J = 2.4 Hz, 1H), 8.58 (d, J = 8.2 Hz, 1H), 8.31 (d, J = 7.5 Hz, 1H), 8.18 (d, J = 8.1 Hz, 1H), 8.00 (d, J = 5.3 Hz, 3H), 7.62 (d, J = 7.9 Hz, 1H), 7.55 (d, J = 7.9 Hz, 1H), 7.32 (dd, J = 13.2, 8.1 Hz, 2H), 7.21 (m, 4H), 7.16 (m, 2H), 7.12 (d, J = 2.3 Hz, 1H), 7.05 (m, 2H), 6.98 (td, J = 7.5, 7.0, 1.0 Hz, 1H), 6.94 (m, 1H), 4.61 (m, 2H), 4.52 (td, J = 7.4, 5.9 Hz, 1H), 3.70 (m, 1H), 3.38 (m, 1H), 3.18 (dd, J = 14.8, 5.9 Hz, 1H), 3.06 (m, 3H), 2.92 (dd, J = 14.9, 8.9 Hz, 1H), 2.80 (dd, J = 14.1, 9.2 Hz, 1H), 1.58 (m, 1H), 1.46 (t, J = 7.2 Hz, 2H), 0.84 (dd, J = 6.5, 3.8 Hz, 6H).

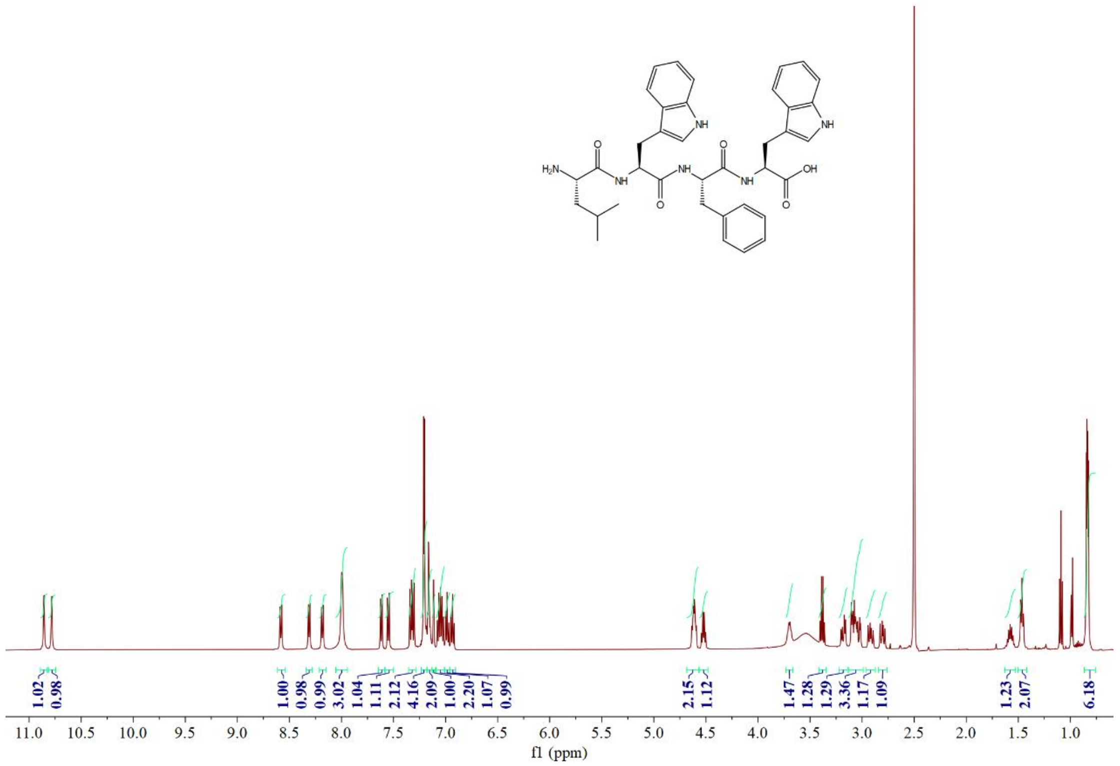

2.Compound **WVFY**: ^{1}H NMR (500 MHZ, DMSO-d6) δ 10. 97 (d, J = 2.5 Hz, 1H), 8.56 (d, J = 8.9 Hz, 1H), 8.19 (t, J = 8.3 Hz, 2H), 7.92 (td, J = 5.4 Hz, 3H), 7.68 (d, J = 7.9 Hz, 1H), 7.36 (d, J = 8.1 Hz, 1H), 7.26 (m, 2H), 7.21 (t, J = 7.6 Hz, 2H), 7.09 (m, 3H), 7.01 (d, J = 8.4 Hz, 2H), 6.92 (m, 1H), 6.64 (d, J = 8.4 Hz, 2H), 4.63

(m, 1H), 4.29 (td, J = 7.9, 5.5 Hz, 1H), 4.26 (dd, J = 8.9, 6.6 Hz, 1H), 4.09 (dt, J = 9.6, 5.1 Hz, 1H), 3.04 (ddd, J = 22.5, 14.5, 4.5 Hz, 2H), 2.93 (m, 2H), 2.79 (ddd, J = 25.8, 14.0, 8.9 Hz, 2H), 1.96(m, 1H), 0.83 (m, 6H).

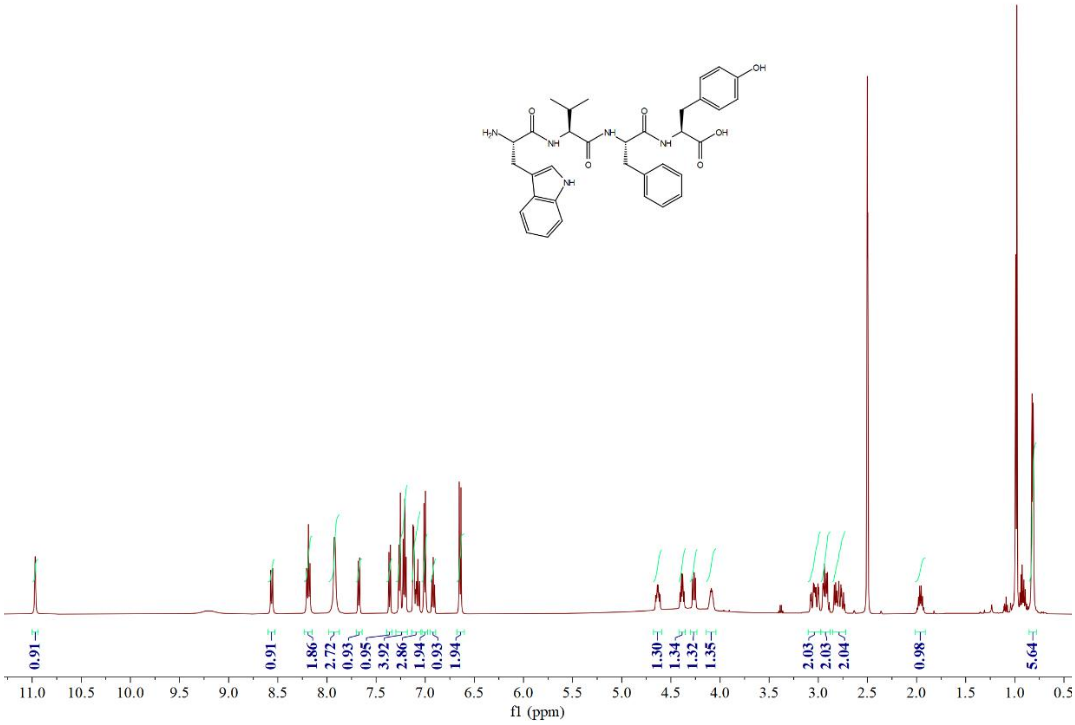

3.Compound **PVFY**: ^{1}H NMR (500 MHZ, DMSO-d6) δ ^{1}H NMR (500 MHZ, DMSO-d6) δ 9.11 (d, *J* = 5.5 Hz, 1H), 8.48 (dd, *J* = 11.4, 5.9 Hz, 1H), 8.42 (d, *J* = 9.0 Hz, 1H), 8.21 (d, *J* = 7.8 Hz, 1H), 8.14 (d, *J* = 8.5 Hz, 1H), 7.19 (m, 5H), 7.00 (d, *J* = 8.5 Hz, 2H), 6.64 (d, *J* = 8.4 Hz, 2H), 4.59 (td, *J* = 10.2, 8.5, 4.1 Hz, 1H), 4.37 (td, *J* = 8.0, 5.4 Hz, 1H), 4.18 (m, 2H), 3.19 (m, 2H). 2.86 (m, 4H), 2.18 (m, 1H), 1.85 (m, 3H), 1.61 (m, 1H), 0.77 (t, *J* = 6.9 Hz, 6H).

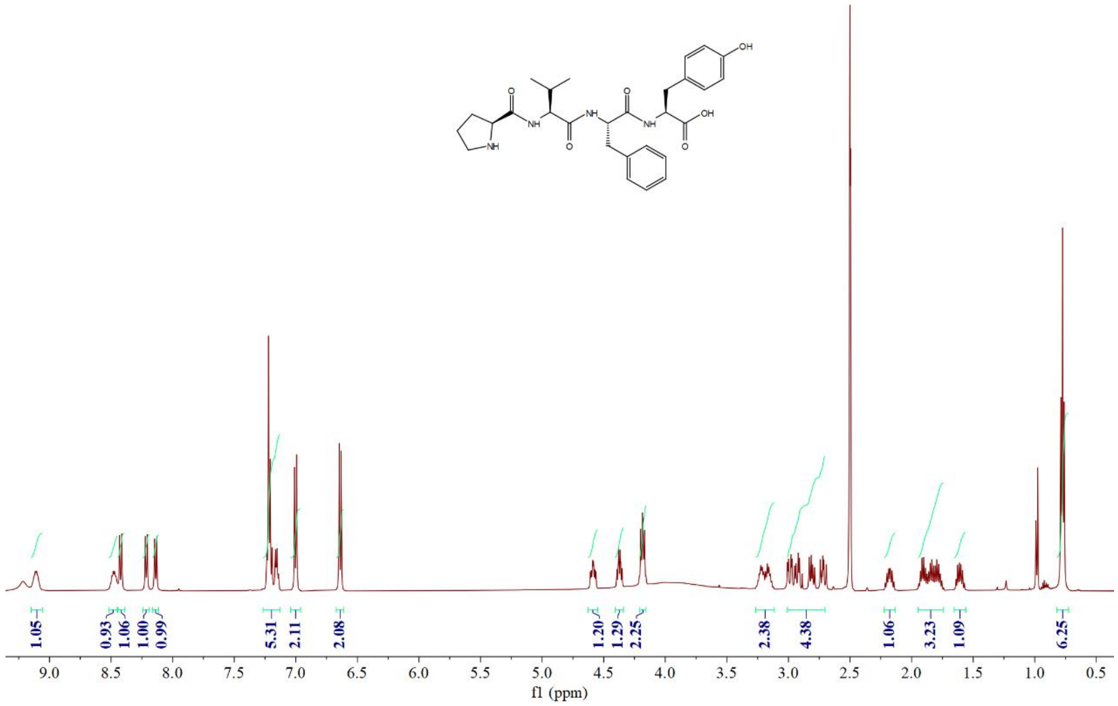

4.Compound **WAYF**: ^{1}H NMR (500 MHZ, DMSO-d6) δ 10.97 (d, J = 2.5 Hz, 1H), 9.14 (s, 1H), 8.74 (d, J = 7.5 Hz, 1H), 8.26 (s, 1H), 8.02 (d, J = 8.2 Hz, 1H), 7.94 (d, J = 5.4 Hz, 3H), 7.67 (d, J = 7.9 Hz, 1H), 7.36 (d, J =8.1 Hz, 1H), 7.23 (m, 5H), 7.17 (d, J = 2.5 Hz, 1H), 7.08 (ddd, J = 8.2, 7.0, 1.1 Hz, 1H), 7.04 (d, J = 8.5 Hz, 2H), 6.92 (ddd, J = 8.0, 6.9, 1.0 Hz, 1H), 6.62 (d, J = 8.4 Hz, 2H), 4.46 (m, 2H), 4.37 (dt, J = 7.0 Hz, 1H), 4.02 (dt, J = 9.7, 4.9 Hz, 1H), 3.18 (dd, J = 14.9, 4.6 Hz, 1H), 3.06 (dd, J = 13.9, 5.3 Hz, 1H), 2.95 (m, 3H), 2.67 (dd, J = 14.1, 9.0 Hz, 1H), 1.20 (d, J = 7.0 Hz, 3H).

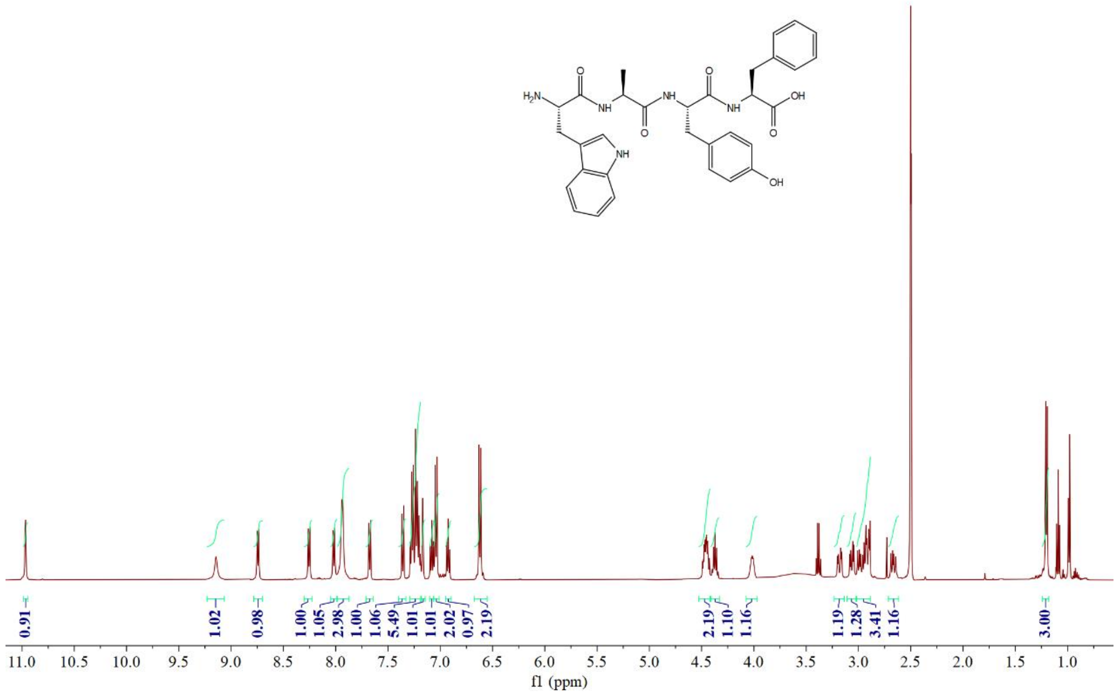

5.Compound **WEFF**: ^{1}H NMR (500 MHZ, DMSO-d6) δ 10.98 (d, J = 2.5 Hz, 1H), 8.71 (d, J = 8.1 Hz, 1H), 8.40 (d, J = 7.8 Hz, 1H), 8.13 (d, J = 8.2 Hz, 1H), 7.94 (m, 3H), 7.66 (d, J = 7.9 Hz, 1H), 7.36 (d, J = 8.1 Hz, 1H), 7.24 (m, 10H), 7.14 (m, 2H), 7.07 (ddd, J = 8.1, 6.9, 1.1 Hz, 1H), 6.92 (m, 1H), 4.60 (m, 1H), 4.48 (td, J = 8.2, 5.4 Hz, 1H), 4.36 (td, J = 8.1, 5.2 Hz, 1H), 4.03 (dt, J = 9.7, 5.0 Hz, 1H), 3.07 (m, 3H), 2.93 (m, 3H), 2.76 (m, 2H), 2.23 (m, 2H), 1.88 (m, 1H), 1.75 (m, 1H).

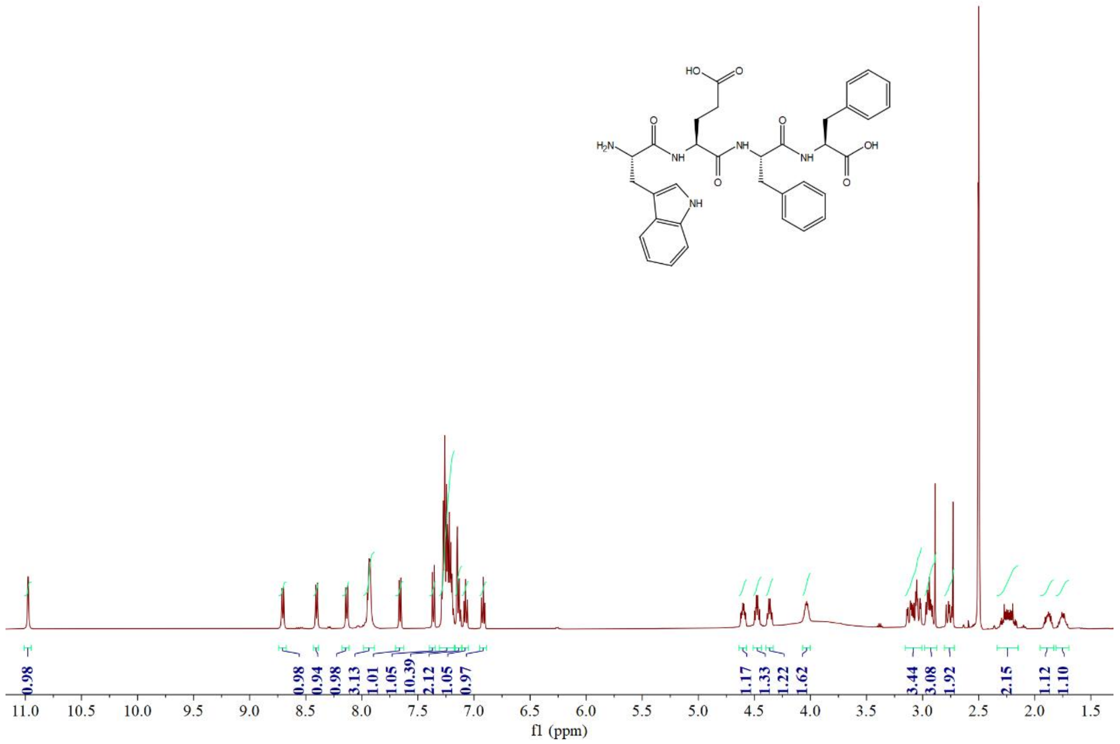

6.Compound **FKFF**: ^{1}H NMR (500 MHZ, DMSO-d6) δ 8.54 (d, J = 8.2 Hz, 1H), 8.41 (d, J = 7.8 Hz, 1H), 8.13 (d, J = 8.3 Hz, 1H), 8.08 (s, 3H), 7.22 (m, 15H), 4.59 (m, 1H), 4.45 (td, J = 8.3, 5.3 Hz, 1H), 4.30 (td, J = 8.2, 5.4 Hz, 1H), 4.03 (s, 1H), 2.93 (m, 6H), 1.52 (m, 4H), 1.24 (m, 2H).

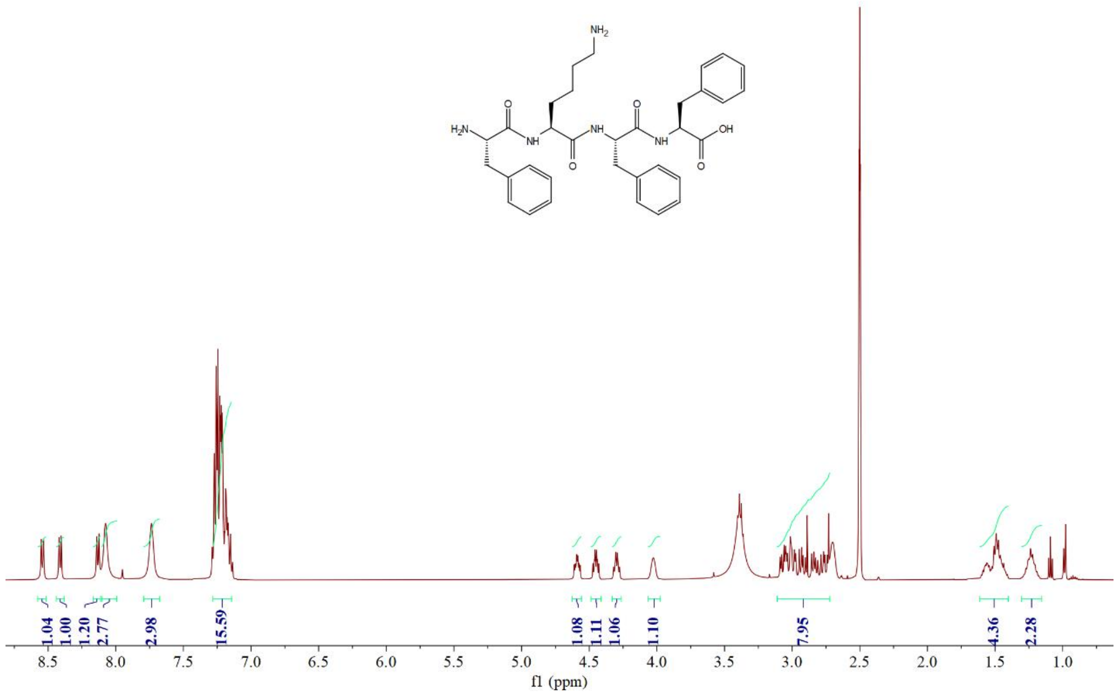

7.Compound **FKFW**: ^{1}H NMR (500 MHZ, DMSO-d6) δ 10.87 (d, J = 2.4 Hz, 1H), 8.56 (d, J = 8.2 Hz, 1H), 8.40 (d, J = 7.5 Hz, 1H), 8.12 (d, J = 8.2 Hz, 1H), 8.07 (s, 3H), 7.71 (s, 3H), 7.53 (d, J = 7.9 Hz, 1H), 7.34 (d, J = 8.1 Hz, 1H), 7.21 (m, 11H), 7.06 (m, 1H), 6.98 (t, J = 7.4 Hz, 1H), 4.62 (m, 1H), 4.49 (td, J = 7.5, 5.7 Hz, 1H), 4.30 (td, J = 8.2, 5.4 Hz, 1H), 4.03 (s, 1H), 3.17 (dd, J = 14.7, 5.7 Hz, 1H), 3.05 (m, 3H), 2.81 (m, 2H), 2.72 (d, J = 12.1 Hz, 2H), 1.59 (m,1H), 1.48 (m, 3H), 1.24 (dd, J = 10.3, 6.2 Hz, 2H).

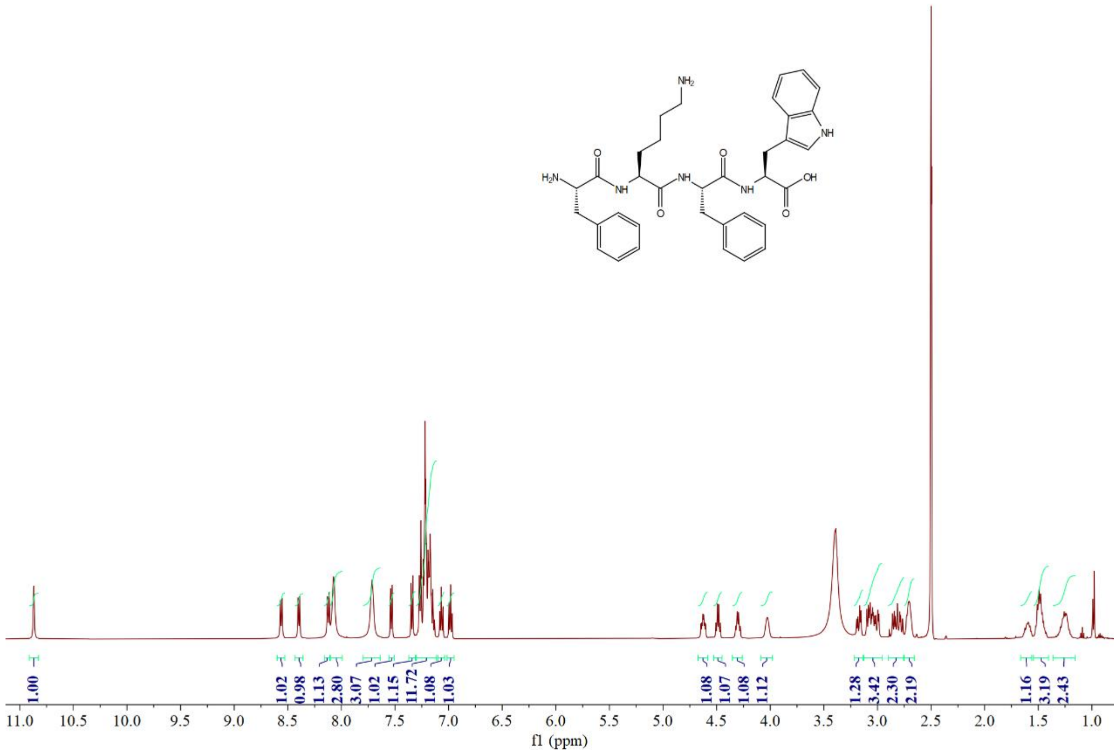

8.Compound **WKFF**: ^{1}H NMR (500 MHZ, DMSO-d6) δ 10.99 (D, J = 2.5 Hz, 1H), 8.70 (d, J = 8.2 Hz, 1H), 8.41 (d, J = 7.8 Hz, 1H), 8.12 (d, J = 8.2 Hz, 1H), 7.95 (d, J = 3.3 Hz, 3H), 7.95 (t, J = 3.5 Hz, 3H), 7.73 (t, J = 5.7 Hz, 3H), 7.67 (d, J = 8.0 Hz, 1H), 7.37 (d, J = 8.1 Hz, 1H), 7.24 (m, 10H), 7.08 (ddd, J = 8.1, 6.9, 1.1 Hz, 1H), 6.93 (m, 1H), 4.61 (td, J = 8.8, 4.3 Hz, 1H), 4.40 (td, J = 8.3, 5.2 Hz, 1H), 4.33 (td, J = 8.2, 5.4 Hz, 1H), 4.02 (m, 1H), 3.05 (m, 2H), 2.92 (m, 3H), 2.76 (m, 2H), 1.60 (m, 2H), 1.49 (m, 3H), 1.24 (m, 3H).

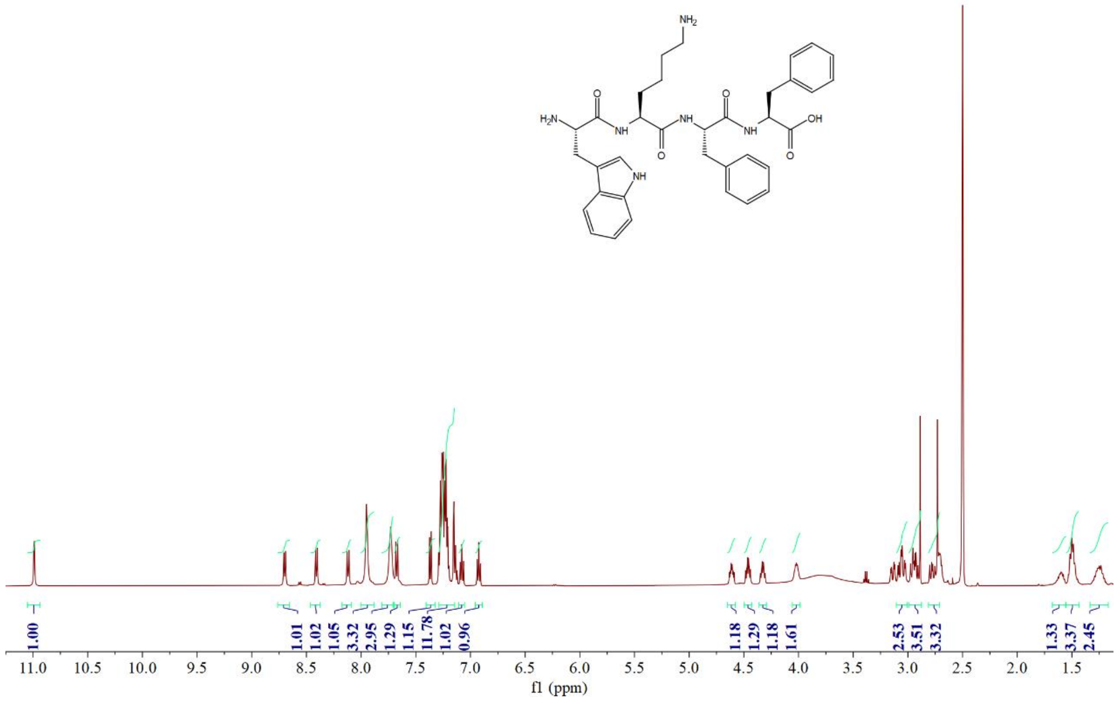

9.Compound **YAWF**: ^{1}H NMR (500 MHz, DMSO-d6) δ 8.37 (d, J = 7.6 Hz, 1H), 8.11 (t, J = 7.4 Hz, 2H), 7.58 (d, J = 7.9 Hz, 1H), 7.33 – 7.26 (m, 1H), 7.26 – 7.17 (m, 5H), 7.15 – 6.93 (m, 5H), 6.74 – 6.61 (m, 2H), 4.55 (td, J = 8.3, 5.0 Hz, 1H), 4.39 (td, J = 7.8, 5.4 Hz, 1H), 4.31 (q, J = 7.1 Hz, 1H), 3.70 (dd, J = 8.9, 4.5 Hz, 1H), 3.38 (q, J = 7.0 Hz, 2H), 3.09 (ddd, J = 28.0, 14.3, 5.2 Hz, 2H), 2.95 – 2.89 (m, 2H), 1.15 (d, J = 7.0 Hz, 3H).

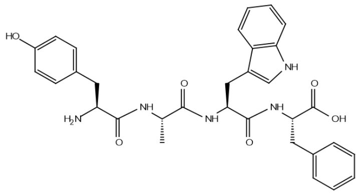

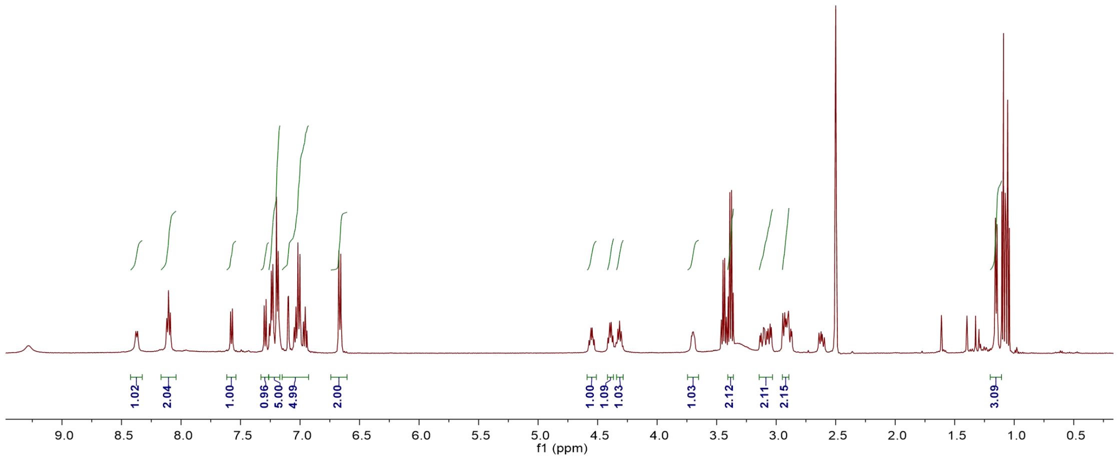

10.Compound **FFFF**: ^{1}H NMR (500 MHZ, DMSO-d6) δ 8.63 (d, J = 8.3 Hz, 1H), 8.39 (d, J = 7.8 Hz, 1H), 8.35 (d, J = 8.3 Hz, 1H), 7.96 (d, J = 5.7 Hz, 3H), 7.22 (m, 20H), 4.61 (m, 2H), 4.49 (td, J = 8.2, 5.3 Hz, 1H), 3.90 (dt, J = 9.5 Hz, 4.9 Hz, 1H), 2.89 (m, 8H).

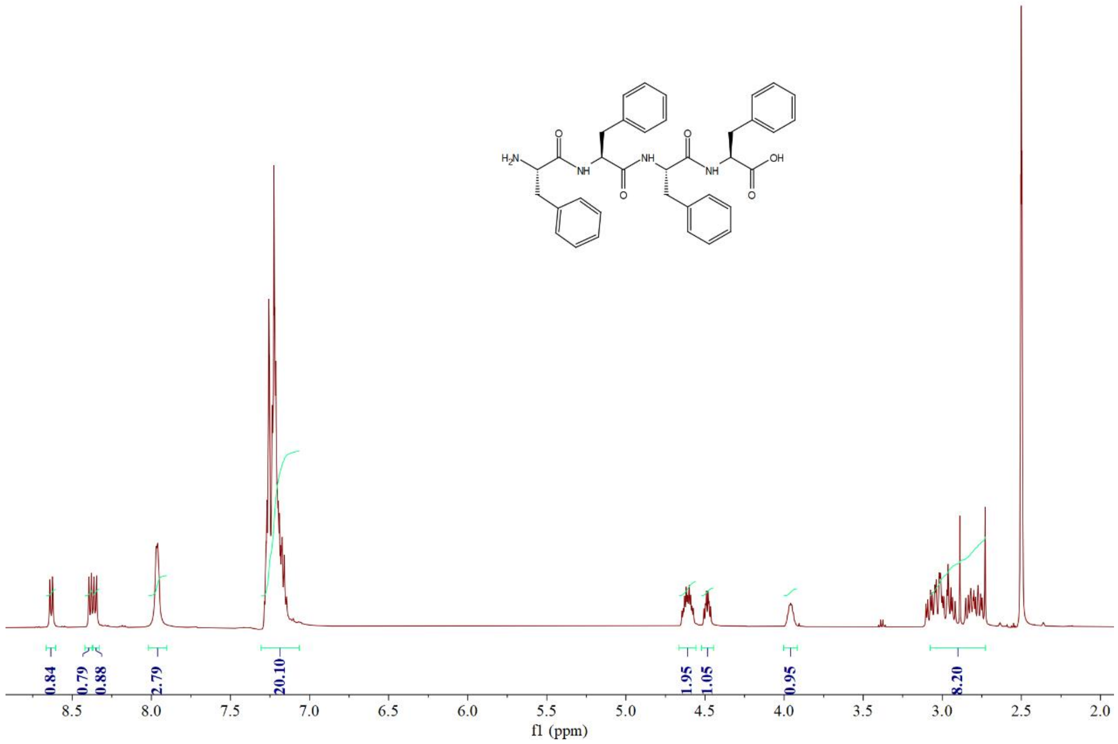

11.Compound **IIII**: ^{1}H NMR (500 MHZ, DMSO-d6) δ 8.35 (d, J = 8.7 Hz, 1H), 8.12 (d, J = 9.1 Hz, 1H), 7.93 (d, J = 8.3 Hz, 1H), 4.29 (dt, J = 12.8, 8.6 Hz, 2H), 4.15 (dd, J = 8.3, 6.3 Hz, 1H), 3.72 (d, J = 5.5 Hz, 1H), 1.72 (m, 4H), 1.44 (m, 4H), 1.20 (m, 1H), 0.82 (m, 24H).

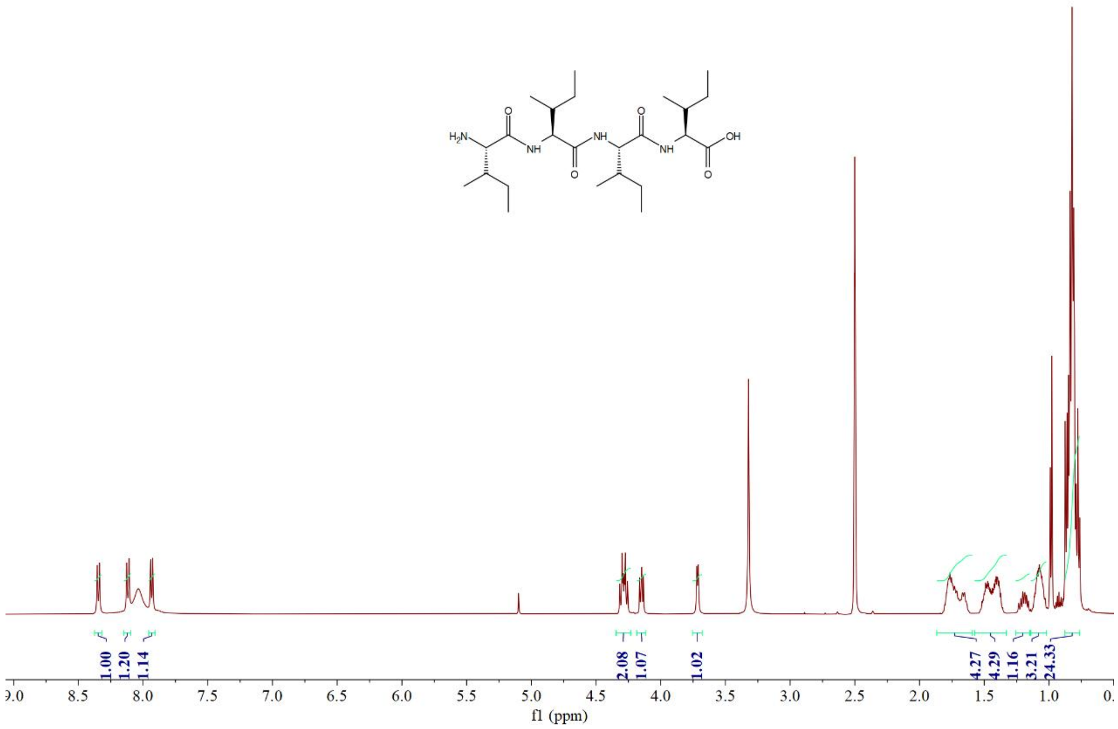

12.Compound **LLLL**: ^{1}H NMR (500 MHZ, DMSO-d6) δ 8.56 (d, J = 8.4 Hz, 1H), 8.15 (d, J = 8.5 Hz, 1H), 8.08 (d, J = 5.3 Hz, 3H), 8.00 (d, J = 8.0 Hz, 1H), 4.43 (td, J = 8.7, 5.9 Hz, 1H), 4.35 (td, J = 8.9, 5.8 Hz, 1H), 4.21 (m, 1H), 3.77 (td, J = 6.4 Hz, 1H), 1.60 (m, 3H), 1.47 (m, 6H), 0.86 (m, 24H).

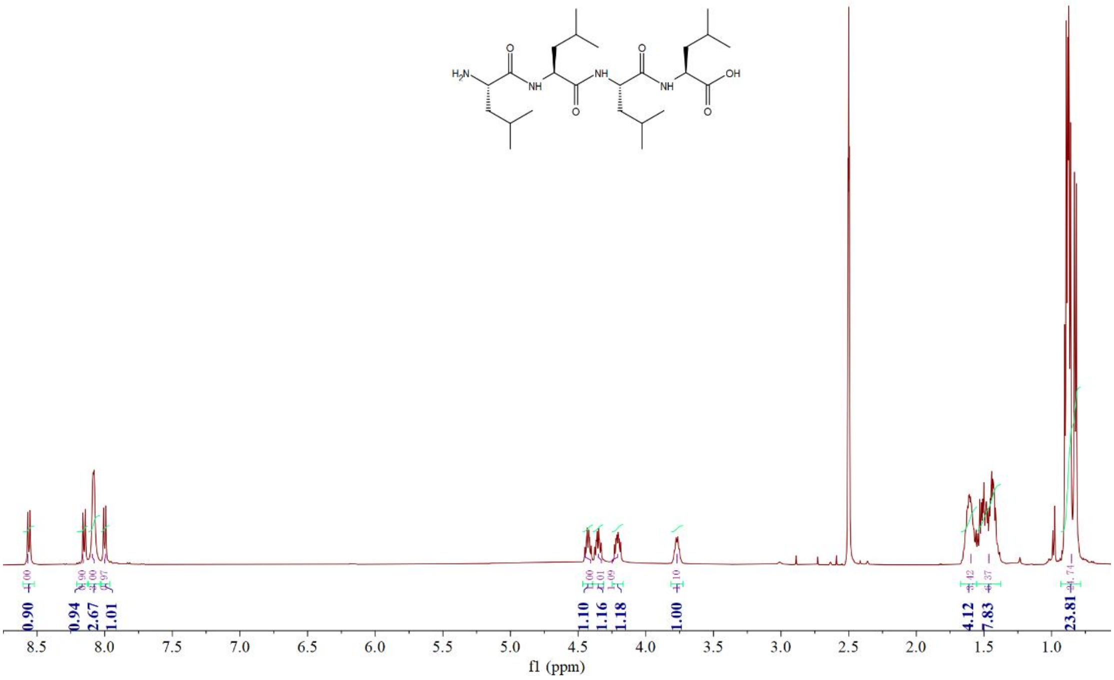

13.Compound **VVVV**: ^{1}H NMR (500 MHZ, DMSO-d6) δ 8.35 (d, J = 8.5 Hz, 1H), 8.05 (m, 4H), 7.87 (d, J = 8.3 Hz, 1H), 4.27 (m, 2H), 4.13 (dd, J = 8.3, 5.8 Hz, 1H), 3.71 (td, J = 5.6 Hz, 1H), 1.99 (m, 4H), 0.87 (m, 24H).

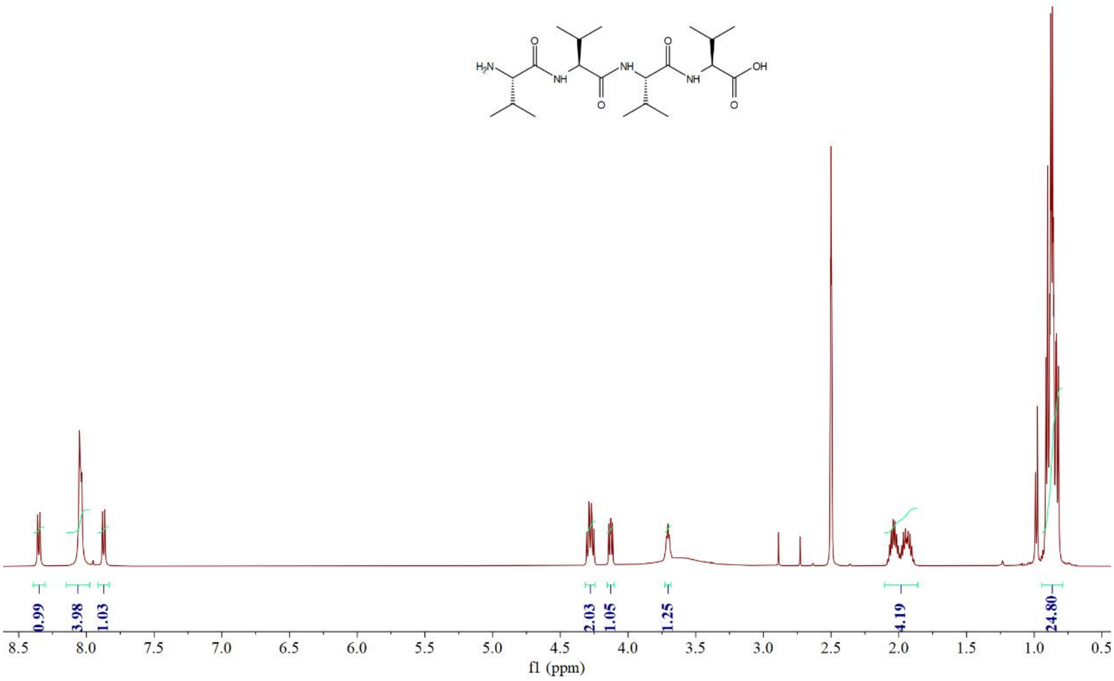

14.Compound **MMMM**: ^{1}H NMR (500 MHz, DMSO-d6) δ 8.58 (d, J = 7.7 Hz, 1H), 8.23 (d, J = 7.8 Hz, 1H), 8.20 (d, J = 7.7 Hz, 1H), 4.40 (q, J = 7.5 Hz, 1H), 4.31 (ddq, J = 21.4, 8.5, 4.4, 3.7 Hz, 2H), 3.81 (d, J = 6.3 Hz, 1H), 2.49 – 2.40 (m, 8H), 2.04 (d, J = 7.3 Hz, 12H), 1.97 – 1.78 (m, 8H).

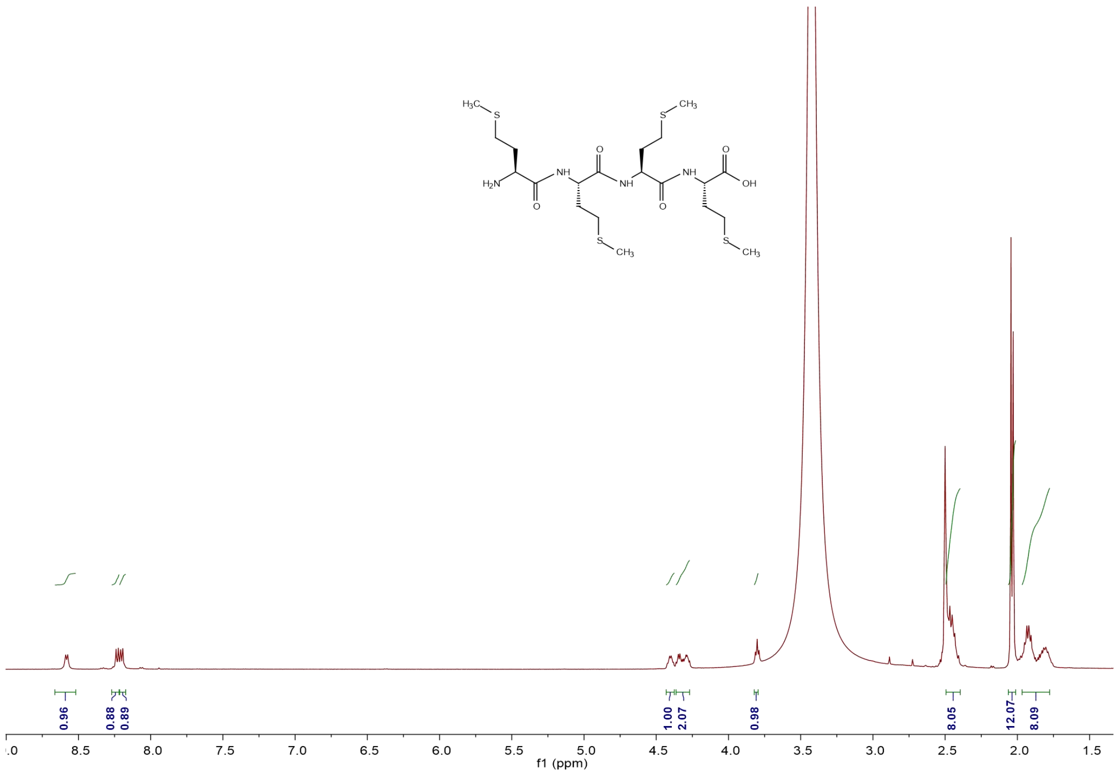

15.Compound **CHFW**: ^{1}H NMR (500 MHz, DMSO-d6) δ 8.97 – 8.87 (m, 1H), 8.59 (d, J = 7.6 Hz, 1H), 8.24 (d, J = 8.1 Hz, 1H), 7.61 – 7.49 (m, 1H), 7.35 – 7.17 (m, 7H), 7.13 – 6.94 (m, 2H), 4.72 – 4.48 (m, 3H), 3.97 (d, J = 5.8 Hz, 1H), 3.22 – 2.75 (m, 8H).

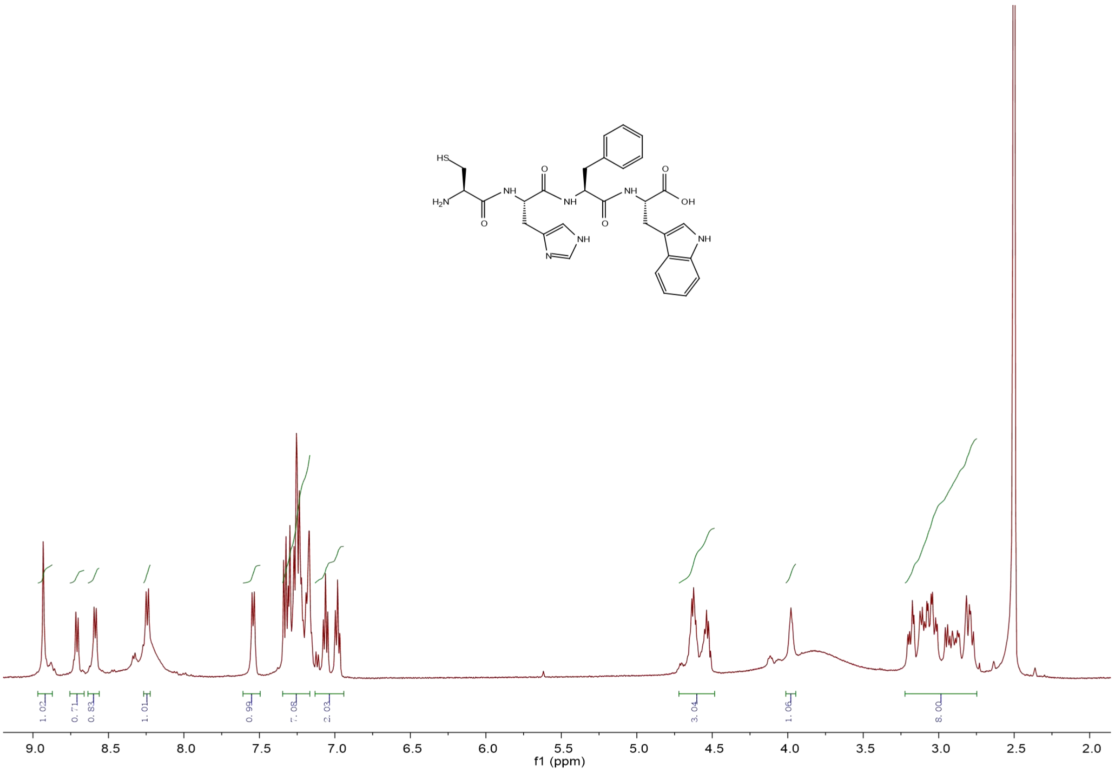

16.Compound **DVFW**:^{1}H NMR (500 MHz, DMSO-d6) δ 8.35 (d, J = 8.8 Hz, 1H), 8.24 (d, J = 7.6 Hz, 1H), 8.10 (t, J = 6.1 Hz, 3H), 7.52 (d, J = 7.9 Hz, 1H), 7.33 (d, J = 8.1 Hz, 1H), 7.18 (dd, J = 40.0, 3.5 Hz, 6H), 7.06 (t, J = 7.5 Hz, 1H), 6.98 (t, J = 7.5 Hz, 1H), 4.61 (td, J = 9.0, 4.3 Hz, 1H), 4.50 (q, J = 6.9 Hz, 1H), 4.17 (dd, J = 8.9, 6.4 Hz, 2H), 3.16 (dd, J = 14.7, 5.9 Hz, 1H), 3.11 – 2.98 (m, 2H), 2.75 (dd, J = 14.2, 9.9 Hz, 1H), 2.66 – 2.55 (m, 2H), 1.93 (h, J = 6.7 Hz, 1H), 0.77 (dd, J = 14.9, 6.8 Hz, 6H).

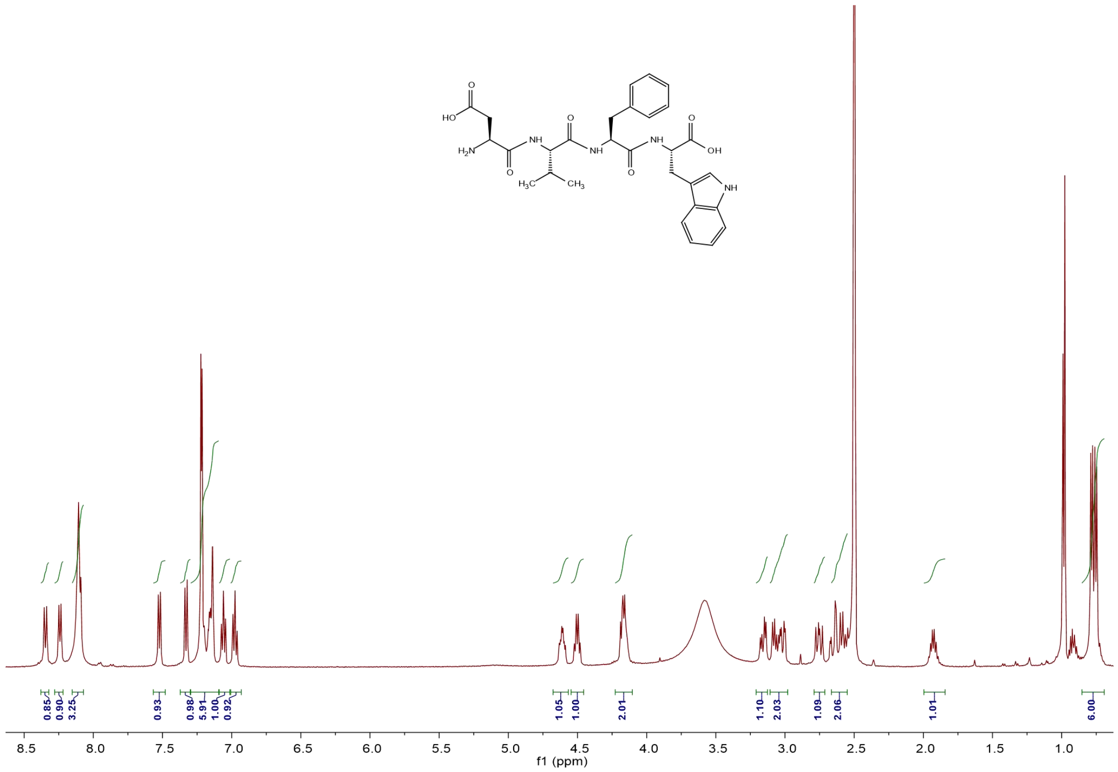

17.Compound **NWWF**: ^{1}H NMR (500 MHZ, DMSO-d6) δ 10.80 (dd, *J* = 19.6, 2.4 Hz, 2H), 8.55 (d, *J* = 7.8 Hz, 1H), 8.34 (d, *J* = 8.0 Hz, 1H), 8.20 (d, *J* = 7.7 Hz, 1H), 7.96 (d, *J* = 5.5 Hz, 3H), 7.66 (m, 2H), 7.59 (d, *J* = 7.9 Hz, 1H), 7.18 (m, 16H), 4.58 (m, 2H), 4.47 (td, *J* = 8.0, 5.5 Hz, 1H), 3.96 (m, 1H), 2.91 (m, 8H).

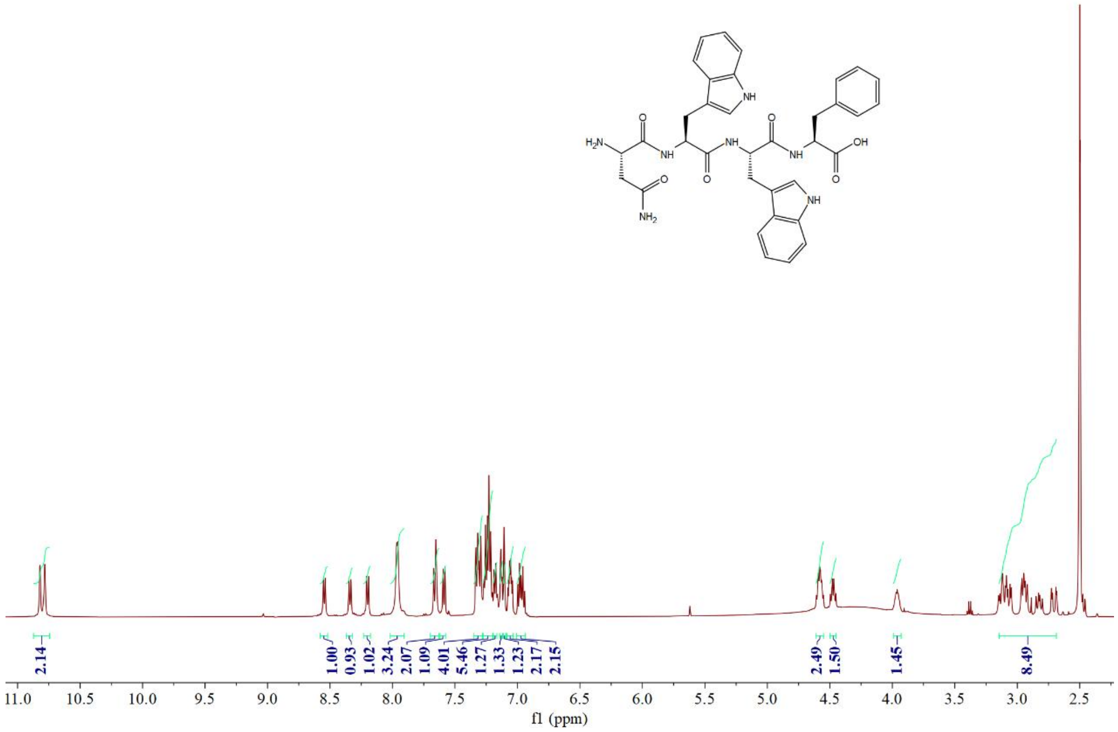

18.Compound **LSFW**: ^{1}H NMR (500 MHZ, DMSO-d6) δ 10.87 (d, *J* = 2.5 Hz, 1H), 8.57 (d, *J* = 8.0 Hz, 1H), 8.37 (d, *J* = 7.6 Hz, 1H), 8.07 (dd, *J* = 18.6, 6.8 Hz, 4H), 7.53 (d, *J* = 7.9 Hz, 1H), 7.34 (d, *J* = 8.1 Hz, 1H), 7.18 (m, 6H), 7.07 (m, 1H), 6.99 (m, 1H), 4.58 (td, *J* = 8.7, 4.1 Hz, 1H), 4.47 (td, *J* = 7.7, 5.6 Hz, 1H), 4.39 (dt, *J* = 8.2, 6.2 Hz, 1H), 3.81 (m, 1H), 3.55 (m, 4H), 2.99 (m, 4H), 1.46 (t, *J* = 7.5 Hz, 2H), 0.98 (d, *J* = 6.7 Hz, 1H), 0.84 (m, 6H).

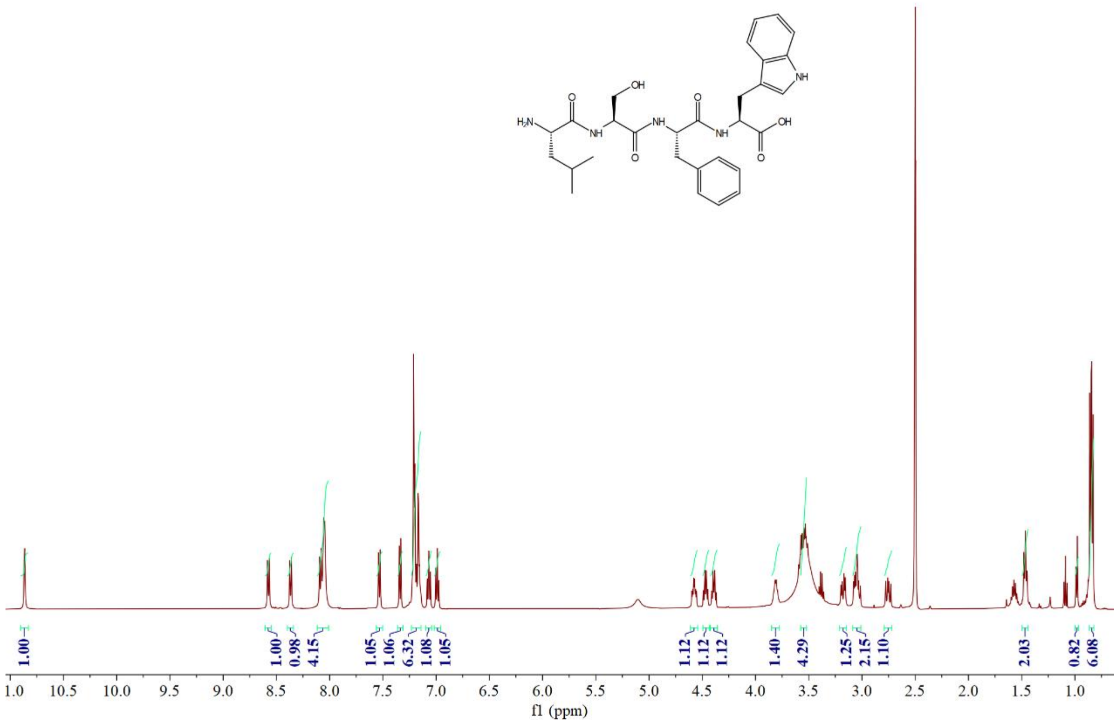

19.Compound **LFWS**: ^{1}H NMR (500 MHZ, DMSO-d6) δ 10.79 (d, *J* = 2.4 Hz, 1H), 8.60 (d, *J* = 8.1 Hz, 1H), 8.28 (d, *J* = 8.0 Hz, 1H), 8.20 (d, *J* = 7.7 Hz, 1H), 7.61 (d, *J* = 7.9 Hz, 1H), 7.31 (d, *J* = 8.1 Hz, 1H), 7.25 (d, *J* = 4.4 Hz, 4H), 7.18 (m, 2H), 7.05 (td, *J* = 8.1, 6.9, 1.2 Hz, 1H), 6.96 (td, *J* = 7.5, 7.0, 1.0 Hz, 1H), 4.63 (m, 3H), 4.30 (m, 1H), 3.35 (m, 8H), 1.52 (m, 2H), 0.98 (d, *J* = 6.7 Hz, 6H), 0.92 (m, 1H).

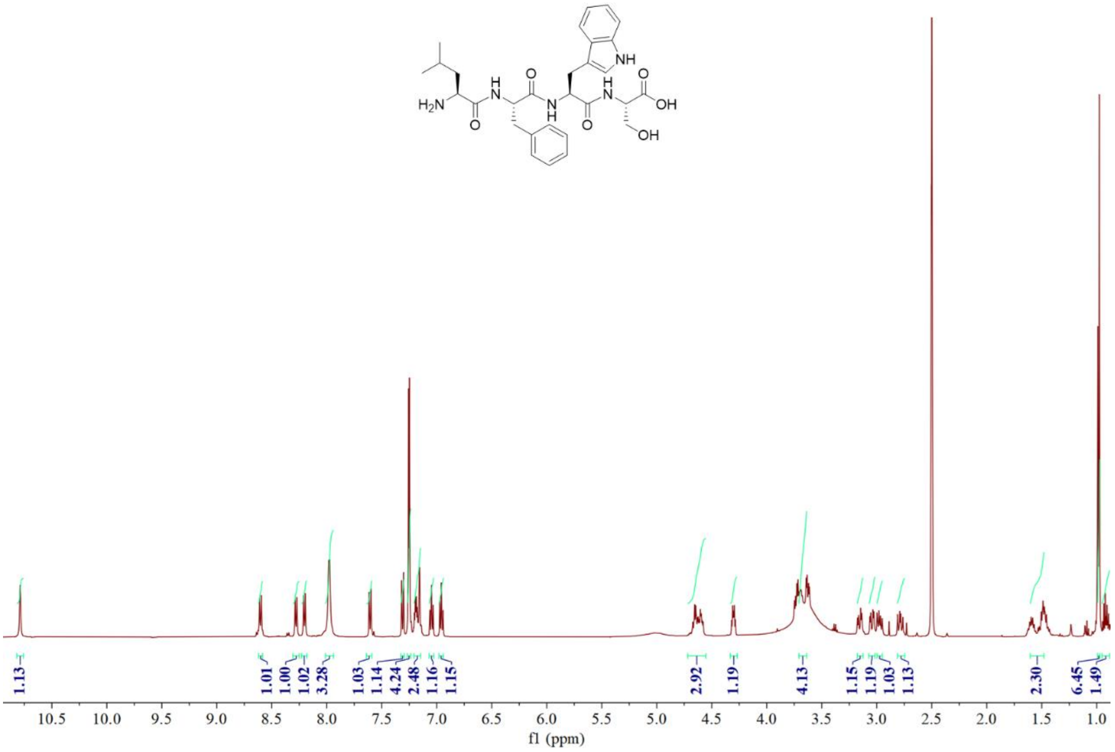

20.Compound **CWHY**: ^{1}H NMR (500 MHz, DMSO-d6) δ 9.24 (s, 1H), 8.94 (d, *J* = 10.6 Hz, 1H), 8.81 (dd, *J* = 21.0, 8.3 Hz, 1H), 8.65 – 8.57 (m, 1H), 8.46 – 8.10 (m, 3H), 7.65 (dd, *J* = 18.5, 7.8 Hz, 1H), 7.31 (dd, *J* = 20.8, 8.9 Hz, 2H), 7.12 – 7.00 (m, 4H), 6.95 (dd, *J* = 14.2, 6.9 Hz, 1H), 6.65 (d, *J* = 7.9 Hz, 2H), 4.67 (dd, *J* = 15.2, 8.2 Hz, 2H), 4.41 – 4.34 (m, 1H), 4.06 (d, *J* = 4.0 Hz, 1H), 3.27 (d, *J* = 12.5 Hz, 1H), 3.16 – 3.02 (m, 3H), 2.94 (dd, *J* = 29.1, 20.5 Hz, 4H), 2.83 (d, *J* = 9.2 Hz, 1H).

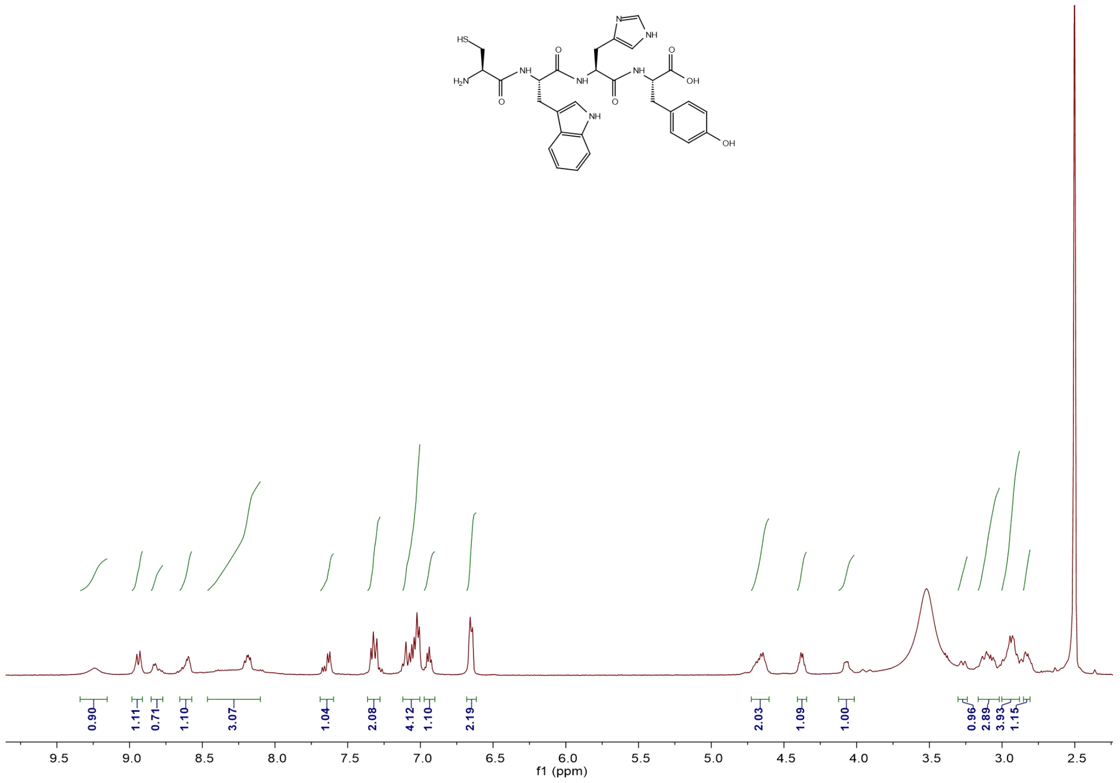

21.Compound **WALW**: ^{1}H NMR (500 MHZ, DMSO-d6) δ 11.00 (d, *J* = 2.5 Hz, 1H), 10.86 (d, *J* = 2.4 Hz, 1H), 8.80 (d, *J* = 7.5 Hz, 1H), 8.08 (dd, *J* = 7.9, 2.0 Hz, 2H), 7.97 (d, *J* = 5.3 Hz, 3H), 7.70 (d, *J* = 7.9 Hz, 1H), 7.52 (d, *J* = 7.9 Hz, 1H), 7.37 (d, *J* = 8.1 Hz, 1H), 7.33 (d, *J* = 8.1 Hz, 1H), 7.21 (d, *J* = 2.4 Hz, 1H), 7.05 (m, 4H), 4.48 (td, *J* = 7.6, 5.5 Hz, 1H), 4.39 (m, 2H), 4.02 (m, 1H), 3.19 (m, 2H), 3.03 (m, 2H), 1.63 (m, 1H), 1.45 (m, 2H), 0.88 (d, *J* = 6.6 Hz, 3H), 0.86 (d, *J* = 6.5 Hz, 2H).

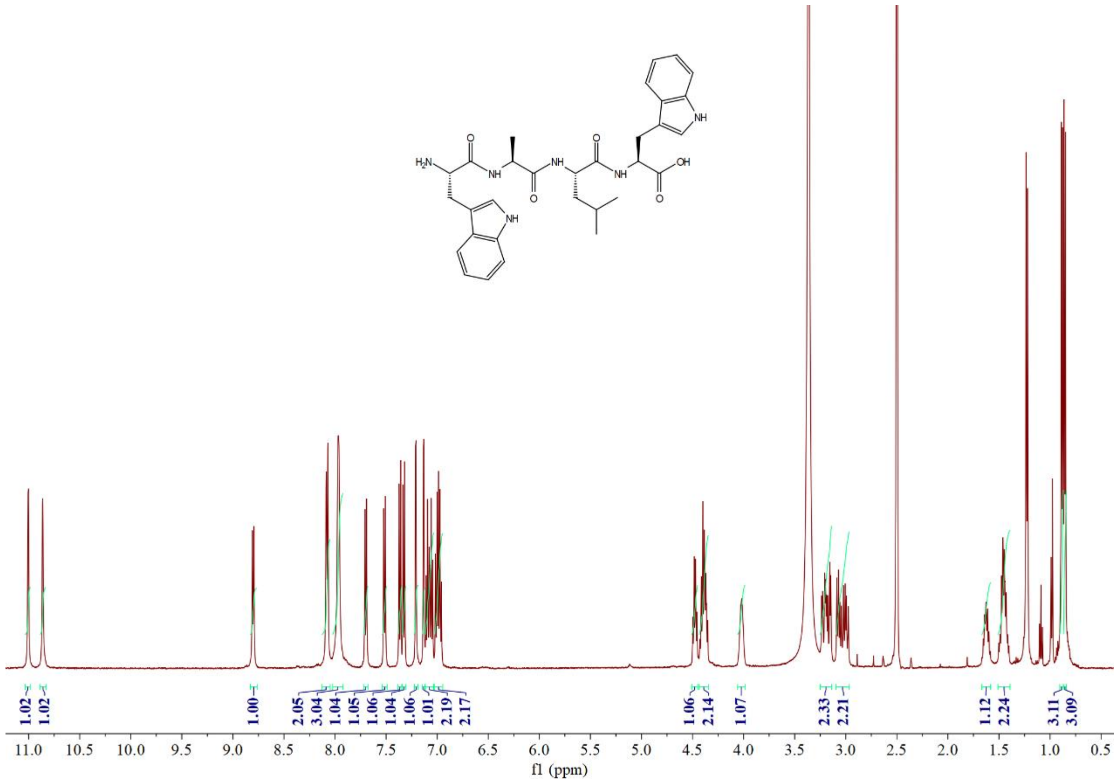

22.Compound **FFYY**: ^{1}H NMR (500 MHZ, DMSO-d6) δ 9.22 (s, 1H), 9.16 (s, 1H), 8.65 (d, *J* = 8.3 Hz, 1H), 8.28 (dd, *J* = 10.0, 8.0 Hz, 2H), 7.97 (d, *J* = 5.2 Hz, 3H), 7.21 (m, 10H), 7.04 (m, 4H), 6.64 (m, 4H), 4.60 (td, *J* = 8.7, 4.2 Hz, 1H), 4.53 (td, *J* = 8.7, 4.4 Hz, 1H), 4.38 (td, *J* = 7.9, 5.5 Hz, 1H), 3.96 (m,, 1H), 2.89 (m, 8H).

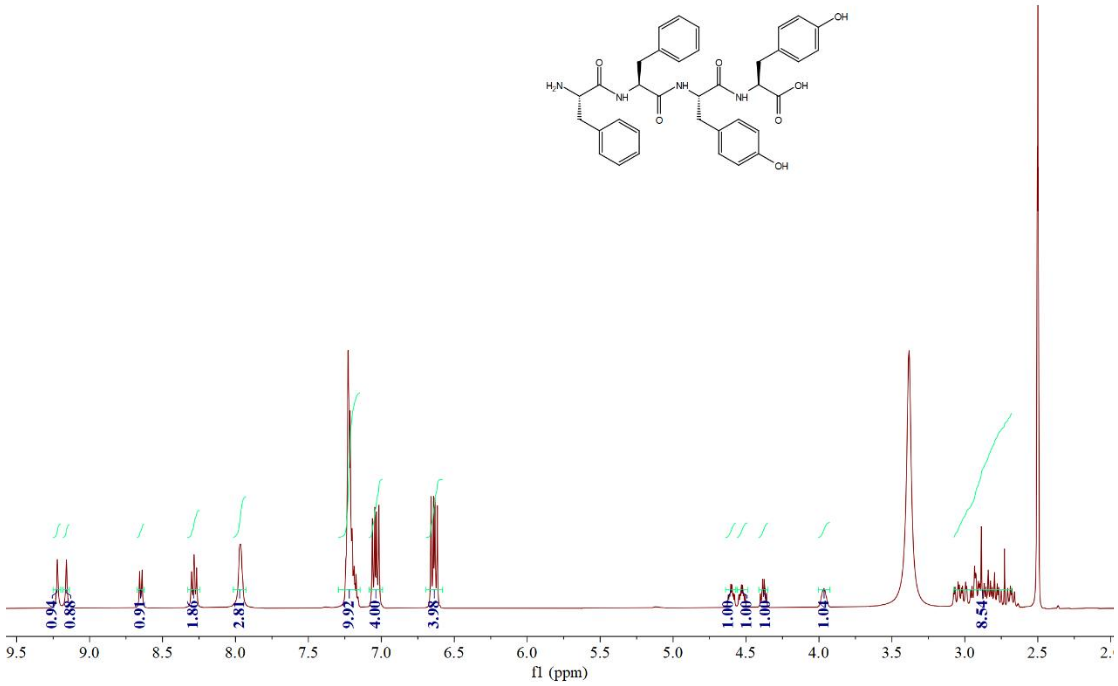

23.Compound **WYFF**: ^{1}H NMR (500 MHZ, DMSO-d6) δ 10.99 (d, *J* = 2.4 Hz, 1H), 8.71 (d, J = 8.2 Hz, 1H), 8.37 (d, J = 7.8 Hz, 1H), 8.29 (d, *J* = 8.2 Hz, 1H), 7.86 (d, *J* = 5.4 Hz, 3H), 7.70 (d, *J* = 7.9 Hz, 1H), 7.36 (d, *J* = 8.1 Hz, 1H), 7.22 (m, 11H), 7.08 (t, *J* = 7.6 Hz, 1H), 7.02 (d, *J* = 8.5 Hz, 2H), 6.94 (t, *J* = 7.5 Hz, 1H), 6.62 (d, *J* = 8.4 Hz, 2H), 4.62 (td, *J* = 8.7, 4.5 Hz, 1H), 4.51 (m, 2H), 3.94 (dt, *J* = 9.8, 4.9 Hz, 1H), 2.93 (m, 8H).

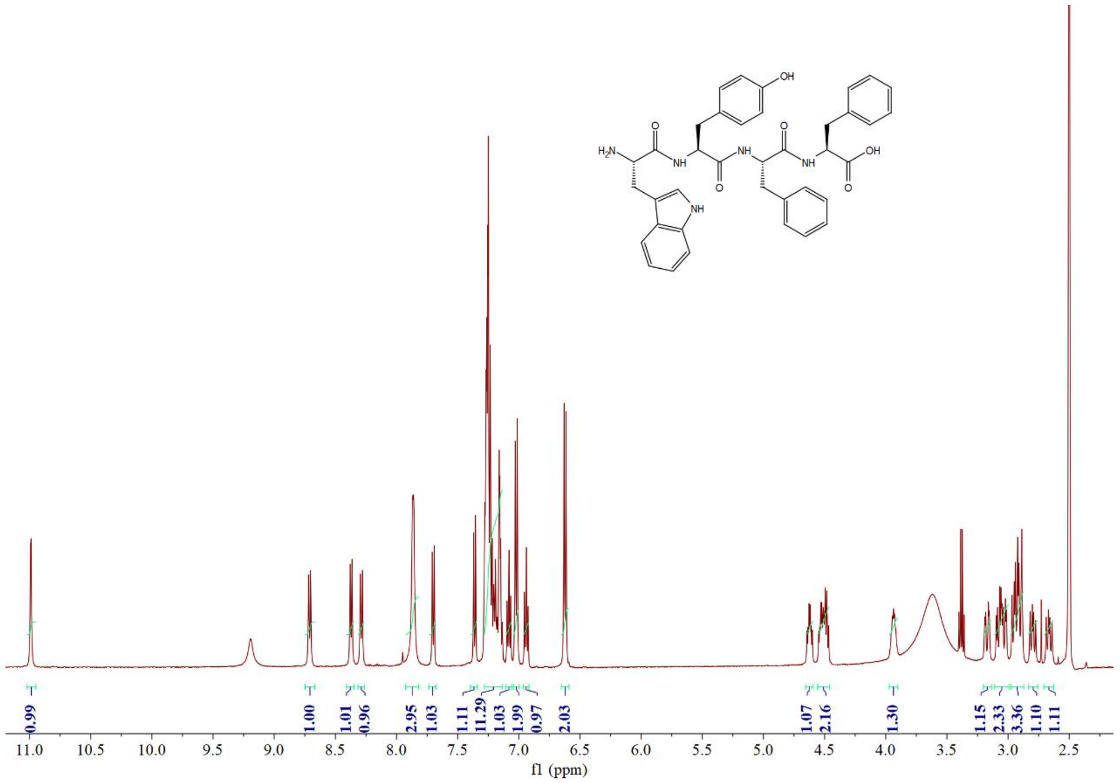

24.Compound **LVFF**: ^{1}H NMR (500 MHZ, DMSO-d6) δ 8.36 (d, *J* = 9.0 Hz, 1H), 8.30 (d, *J* = 7.8 Hz, 1H), 8.16 (d, *J* = 8.4 Hz, 1H), 8.04 (d, *J* = 5.3 Hz, 3H), 7.23 (m, 10H), 4.59 (td, *J* = 10.0, 8.4, 4.1 Hz, 1H), 4.46 (td, *J* = 8.2, 5.2 Hz, 1H), 4.20 (dd, *J* = 9.0, 6.9 Hz, 1H), 3.83 (m, 1H), 1.53 (m, 4H), 0.81 (m, 12H).

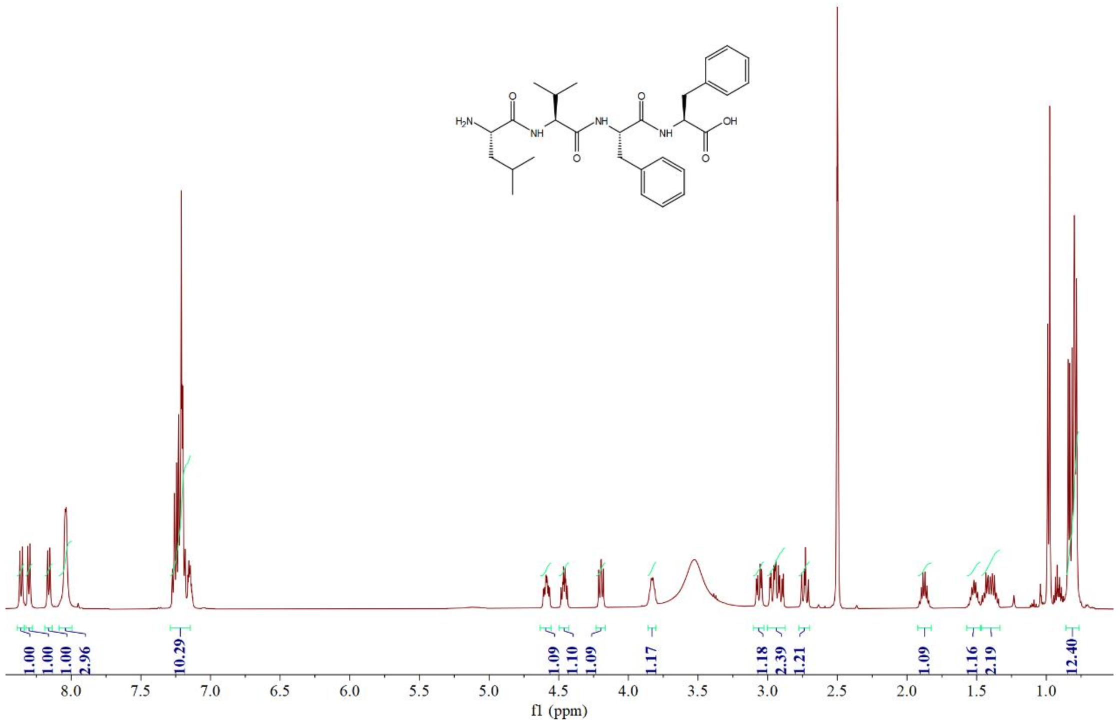

25.Compound **GFFY**: ^{1}H NMR (500 MHZ, DMSO-d6) δ 8.50 (d, *J* = 8.5 Hz, 1H), 8.39 (d, *J* = 8.3 Hz, 1H), 8.30 (d, *J* = 7.7 Hz, 1H), 7.87 (t, *J* = 5.9 Hz, 3H), 7.22 (m, 10H), 7.03 (m, 2H), 6.66 (m, 2H), 4.59 (m, 2H), 4.40 (m, 1H), 2.91 (m, 8H).

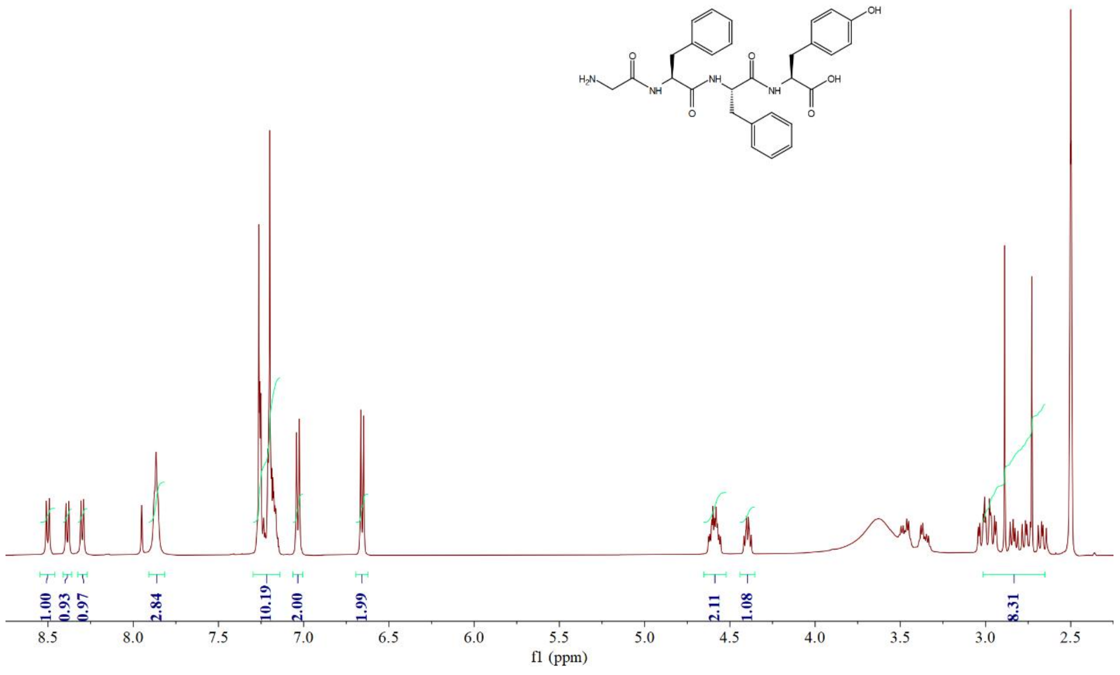

26.Compound **TAVF**: ^{1}H NMR (500 MHZ, DMSO-d6) δ 8.60 (d, *J* = 7.3 Hz, 1H), 8.21 (d, *J* = 7.8 Hz, 1H), 8.06 (d, *J* = 5.2 Hz, 3H), 7.89 (d, J = 9.1 Hz, 1H), 7.23 (m, 5H), 5.49 (s, 1H), 4.44 (m, 2H), 4.16 (dd, *J* = 9.1, 6.9 Hz, 1H), 3.77 (t, *J* = 6.9 Hz, 1H), 3.54 (t, *J* = 6.1 Hz, 1H), 3.05 (dd, *J* = 14.0, 5.1 Hz, 1H), 2.89 (dd, *J* = 14.0, 9.2 Hz, 1H), 1.92 (m, 1H), 1.16 (dd, *J* = 9.1, 6.6 Hz, 6H), 0.80 (dd, *J* = 11.8, 6.8 Hz, 6H).

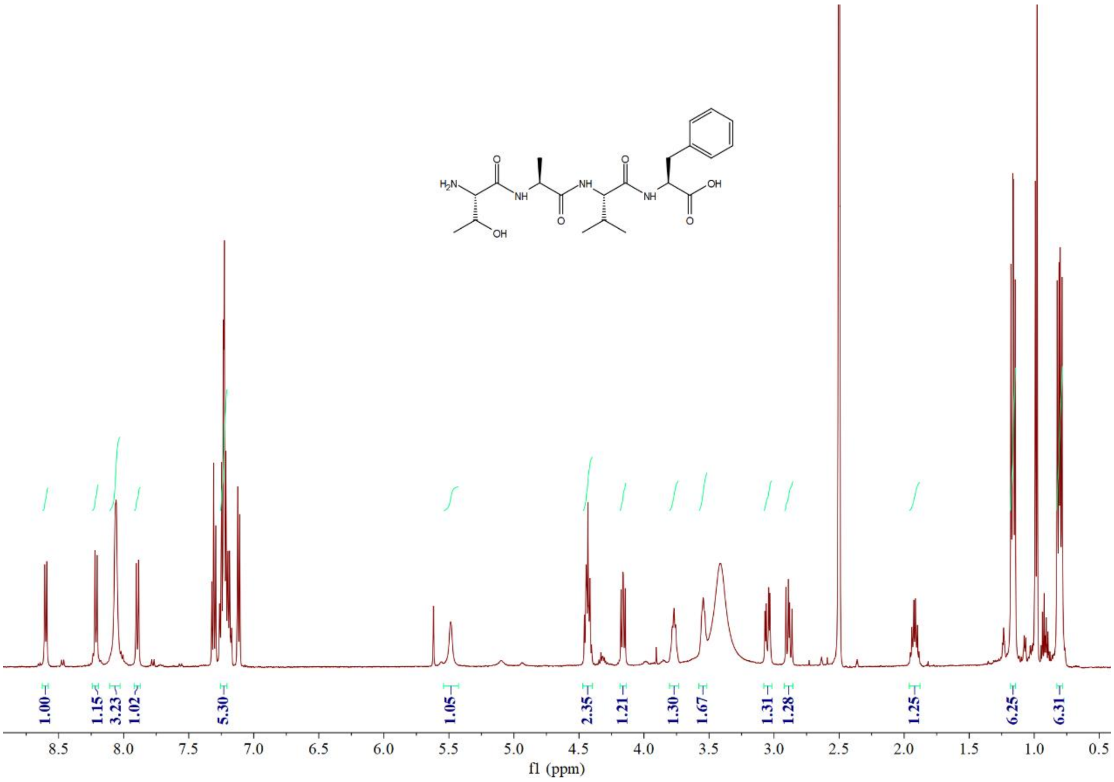

27.Compound **VFMM**: ^{1}H NMR (500 MHZ, DMSO-d6) δ 8.55 (d, *J* = 7.9 Hz, 1H), 8.35 (d, *J* f= 8.0 Hz, 1H), 8.23 (d, *J* = 7.7 Hz, 1H), 7.97 (s, 3H), 7.27 (m, 4H), 7.21 (m, 1H), 4.65 (m, 1H), 4.35 (m, 2H), 3.58 (s, 1H), 3.00 (td, *J* = 14.1, 4.4 Hz, 1H), 2.80 (m, 1H) 2.45 (m, 3H), 2.10 (m, 1H), 2.03 (d, *J* = 7.4 Hz, 6H), 1.85 (m, 4H), 0.93 (d, *J* = 6.9 Hz, 3H), 0.87 (d, *J* = 6.9 Hz, 3H).

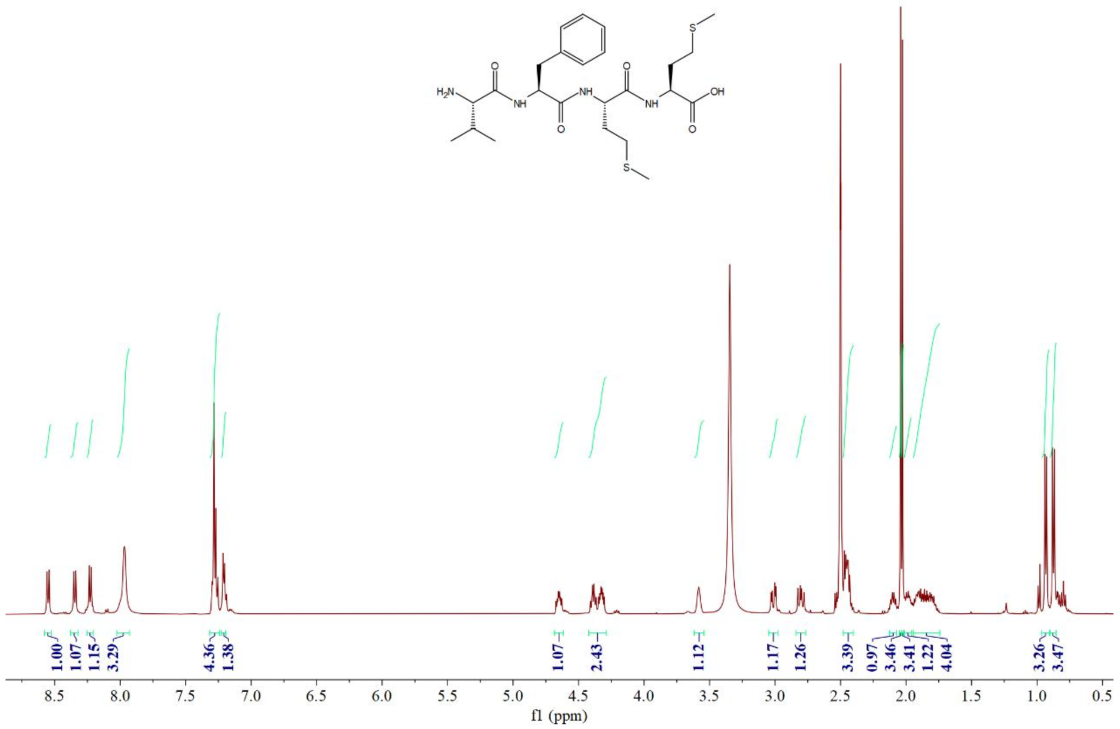

28.Compound **YVFA**: ^{1}H NMR (500 MHZ, DMSO-d6) δ 9.33 (s, 1H), 8.40 (d, *J* = 8.9 Hz, 1H), 8.28 (d, *J* = 7.3 Hz, 1H), 8.18 (d, *J* = 8.3 Hz, 1H), 7.94 (s, 3H), 7.22 (m, 5H), 6.98 (d, *J* = 8.4 Hz, 2H), 6.67 (d, *J* = 8.4 Hz, 2H), 4.62 (m, 1H), 4.22 (m, 2H), 4.00 (s, 1H), 3.05 (dd, *J* = 14.1, 4.3 Hz, 1H), 2.82 (m, 2H), 2.68 (dd, *J* = 14.4, 8.5 Hz, 1H), 1.93 (m, 1H), 1.28 (d, *J* = 7.3 Hz, 3H), 0.83 (t, *J* = 7.1 Hz, 6H).

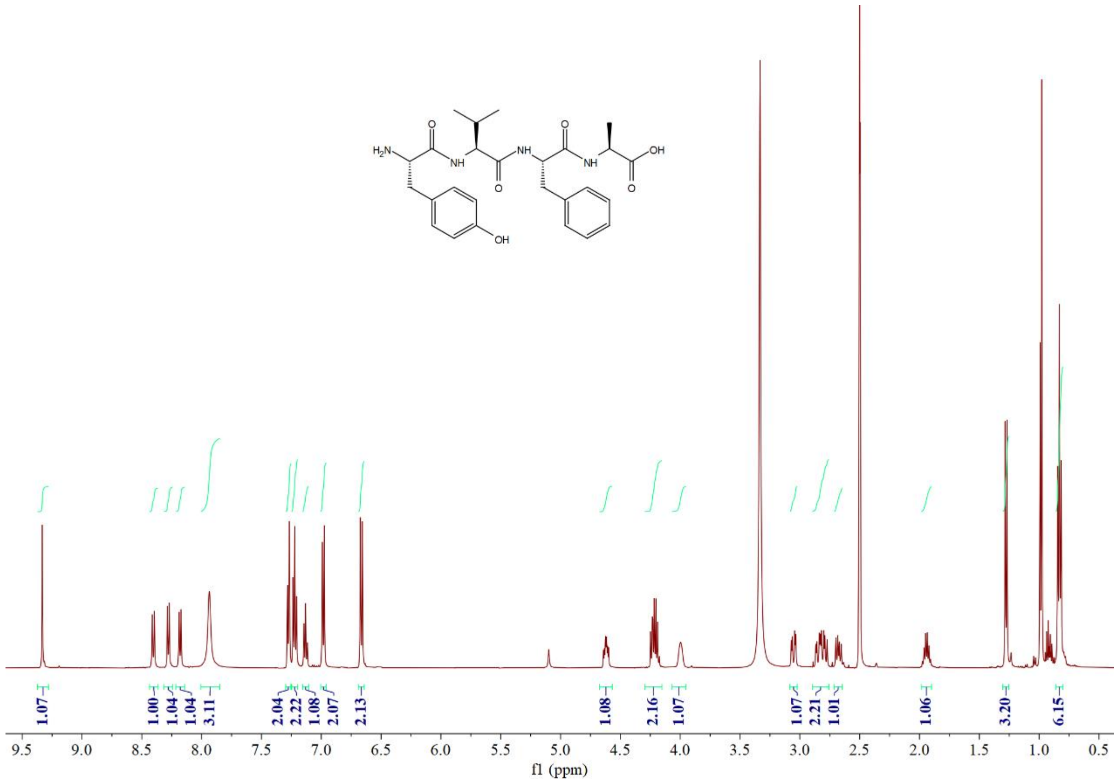

29.Compound **LVFG**: ^{1}H NMR (500 MHZ, DMSO-d6) δ 8.34 (m, 2H), 8.20 (d, *J* = 8.4 Hz, 1H), 8.04 (d, *J* = 5.2 Hz, 3H), 7.22 (m, 4H), 7.16 (m, 1H), 4.60 (m, 1H), 4.21 (dd, *J* = 8.9, 6.9 Hz, 1H), 3.83 (m, 1H), 3.78 (dd, *J* = 11.9, 5.8 Hz, 2H), 3.02 (dd, *J* = 14.1, 4.3 Hz, 1H), 2.79 (dd, *J* = 14.1, 9.8 Hz, 1H), 1.92 (m, 1H), 1.54 (m, 1H), 1.42 (m, 2H), 0.92 (m, 1H), 0.84 (m, 12H).

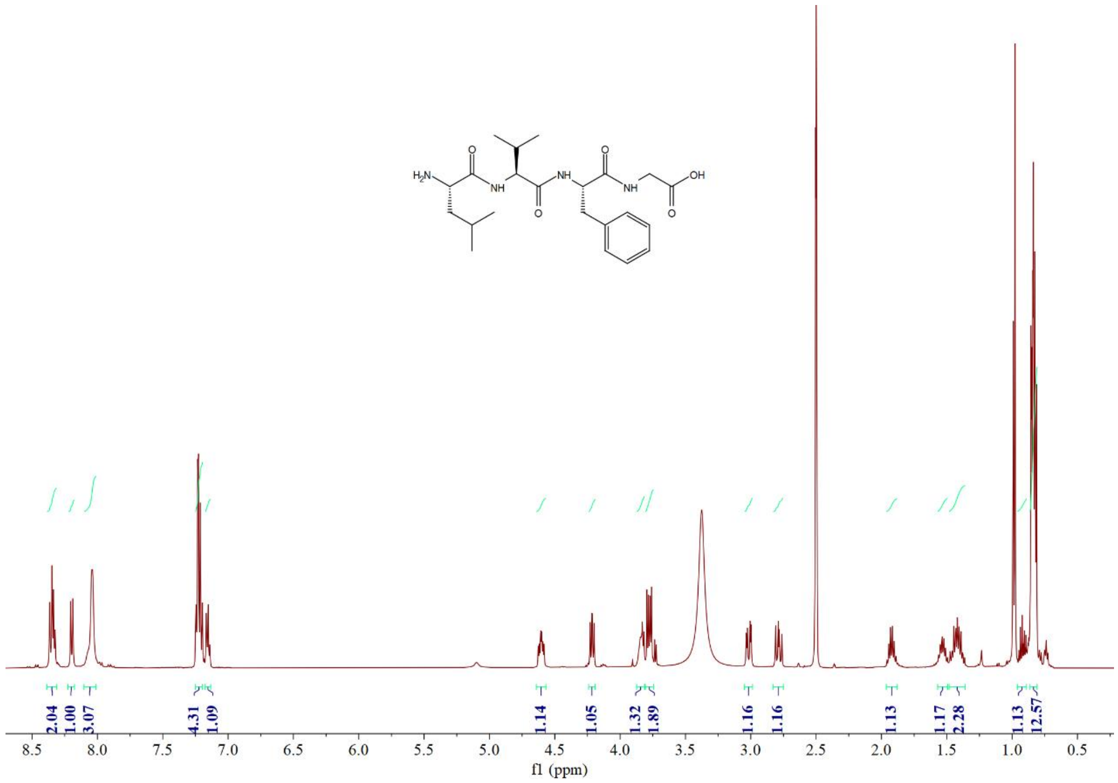

30.Compound **MVFP**: ^{1}H NMR (500 MHZ, DMSO-d6) δ 8.35 (m, 2H), 8.11 (d, *J* = 6.1 Hz, 3H), 7.24 (m, 5H), 4.68 (td, *J* = 8.3, 4.9 Hz, 1H), 4.23 (ddd, *J* = 8.6, 5.5, 3.2 Hz, 2H), 3.90 (s, 1H), 3.66 (dt, *J* = 9.9, 6.7 Hz, 1H), 3.45 (dt, *J* = 9.7, 6.3 Hz, 1H), 2.99 (dd, *J* = 14.1, 5.0 Hz, 1H), 2.80 (m, 1H), 2.39 (t, *J* = 8.1 Hz, 2H), 2.14 (m, 1H), 2.03 (d, *J* = 4.6 Hz, 1H), 1.99 (m, 3H), 1.88 (m, 6H), 0.83 (dd, *J* = 12.6, 6.7 Hz, 6H).

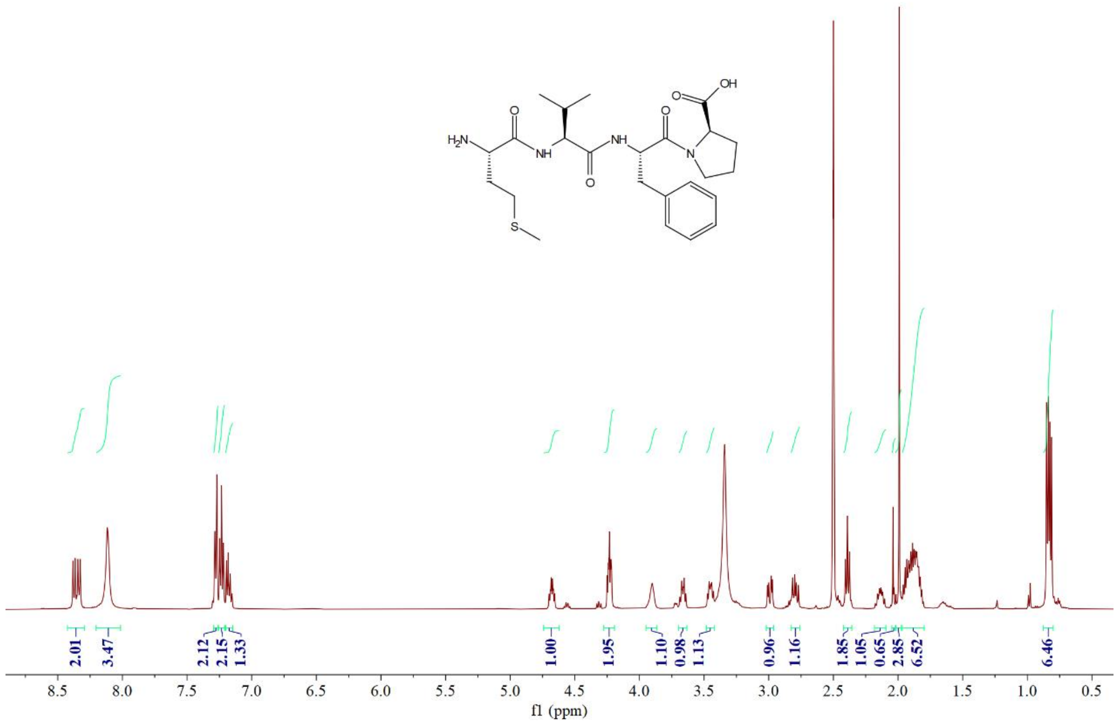

31.Compound **FMLF**: ^{1}H NMR (500 MHZ, DMSO-d6) δ 8.65 (d, *J* = 8.1 Hz, 1H), 8.13 (m, 5H), 7.24 (m, 10H), 4.43 (m, 2H), 4.35 (m, 1H), 4.08 (m, 1H), 3.06 (m, 2H), 2.91 (m, 2H), 2.41 (m, 2H), 2.02 (s, 3H), 1.87 (m, 1H), 1.75 (m, 1H), 1.59 (m, 1H), 1.42 (t, *J* = 7.3 Hz, 2H), 0.89 (d, *J* = 6.6 Hz, 3H), 0.85 (d, *J* = 6.5 Hz, 3H).

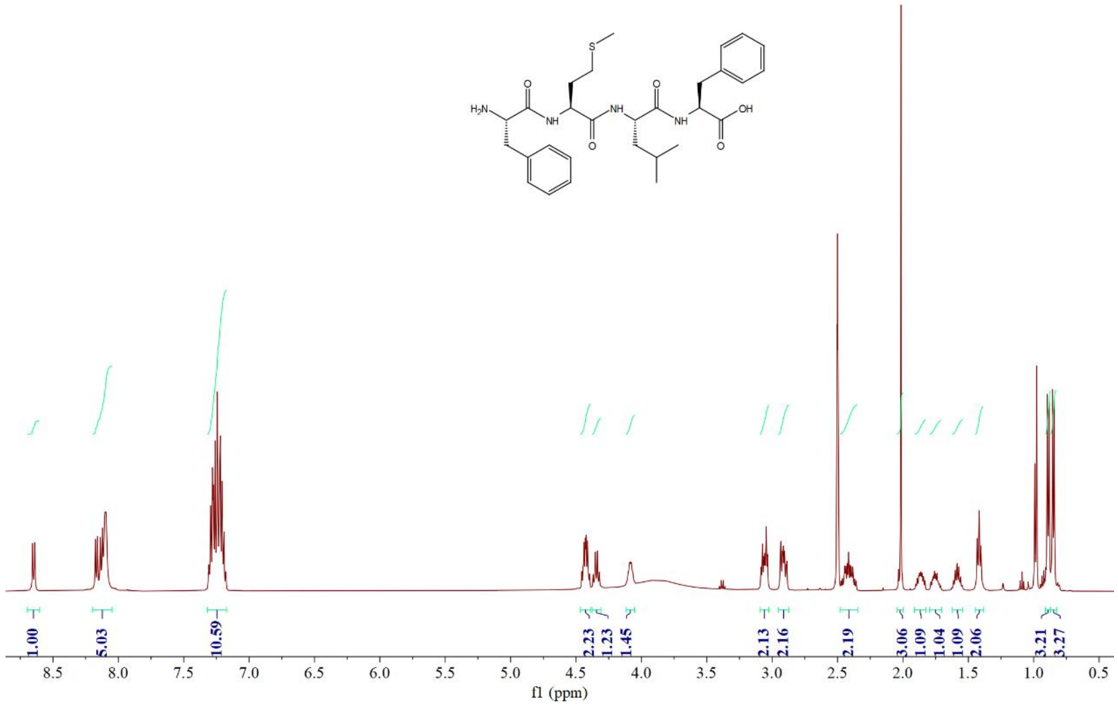

32.Compound **LLLS**: ^{1}H NMR (500 MHZ, DMSO-d6) δ 8.57 (d, *J* = 8.3 Hz, 1H), 8.16 (d, *J* = 8.5 Hz, 1H), 8.08 (d, *J* = 5.4 Hz, 3H), 7.93 (d, *J* = 7.7 Hz, 1H), 4.42 (m, 3H), 4.23 (m, 1H), 3.77 (dd, *J* = 6.4 Hz, 1H), 3.70 (dd, *J* = 10.9, 5.2 Hz, 1H), 3.61 (dd, *J* = 10.9, 4.2 Hz, 1H), 1.52 (m, 10H), 0.88 (m, 15H), 0.82 (d, *J* = 6.5 Hz, 3H).

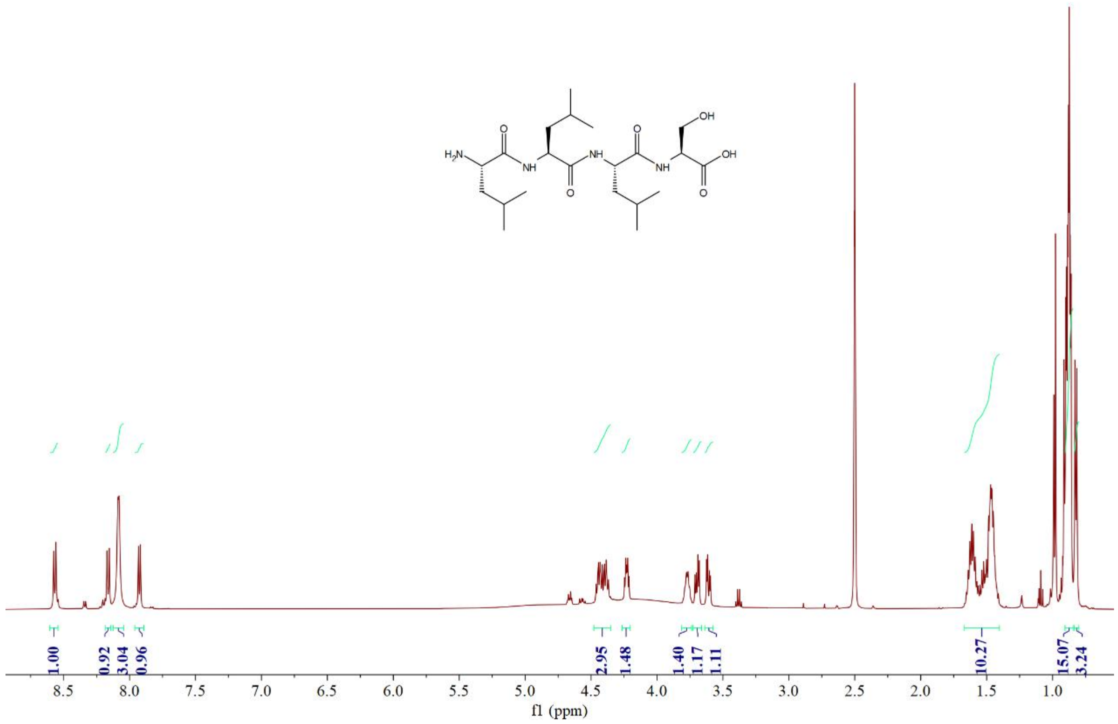

33.Compound **IFYT**: ^{1}H NMR (500 MHZ, DMSO-d6) δ 9.11 (S, 1H), 8.45 (d, *J* = 8.4 Hz, 1H), 8.31 (d, *J* = 8.2 Hz, 1H), 7.96 (m, 3H), 7.22 (m, 5H). 7.05 (d, *J* = 8.4 Hz, 2H), 6.60 (d, *J* = 8.4 Hz, 2H), 4.63 (m, 2H), 4.25 (m, 1H), 4.17 (m, 1H), 3.59 (m, 1H), 2.98 (ddd, *J* = 19.7, 14.3, 4.1 Hz, 2H), 2.75 (ddd, J = 37.1, 14.2, 9.4 Hz, 2H), 1.78 (m, 1H), 1.37 (m, 1H), 1.07 (d, *J* = 6.3 Hz, 3H), 1.00 (m, 1H), 0.80 (m, 6H).

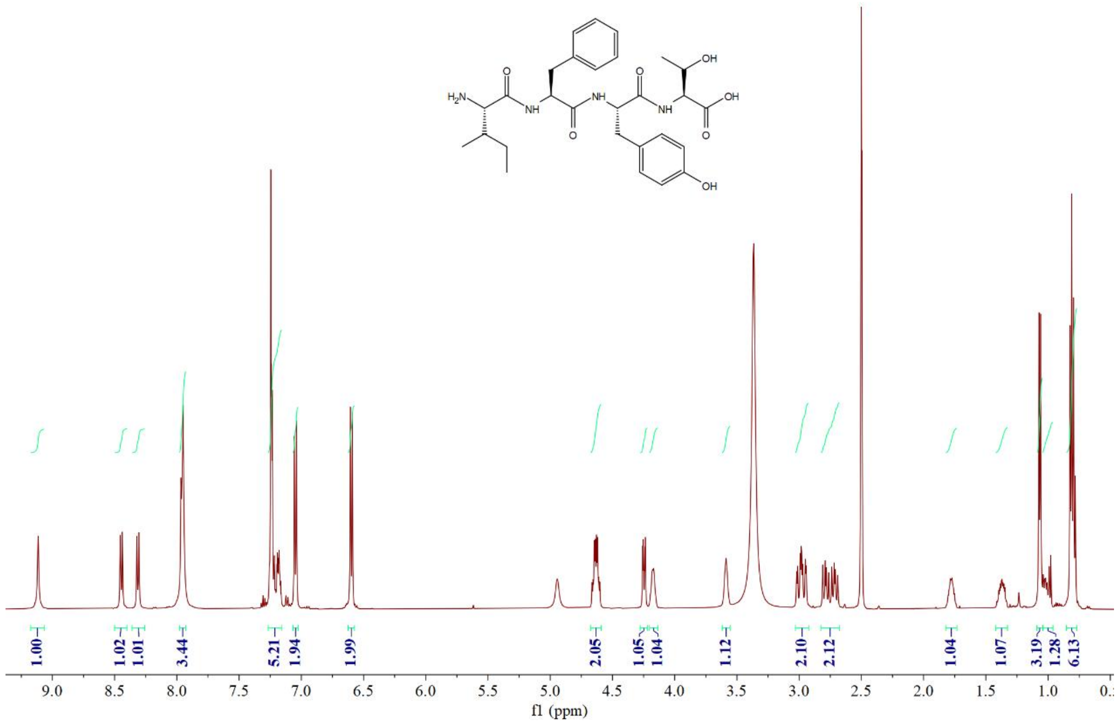

34.Compound **LLSL**: ^{1}H NMR (500 MHZ, DMSO-d6) δ 8.55 (d, *J* = 8.3 Hz, 1H), 8.11 (d, *J* = 7.8 Hz, 1H), 8.07 (d, *J* = 5.1 Hz, 3H), 7.98 (d, *J* = 8.1 Hz, 1H), 4.46 (m, 1H), 4.30 (m, 1H), f4.24 (m, 1H), 3.79 (m, 1H), 1.54 (m, 10H), 0.89 (m, 15H), 0.83 (d, *J* = 6.5 Hz, 3H).

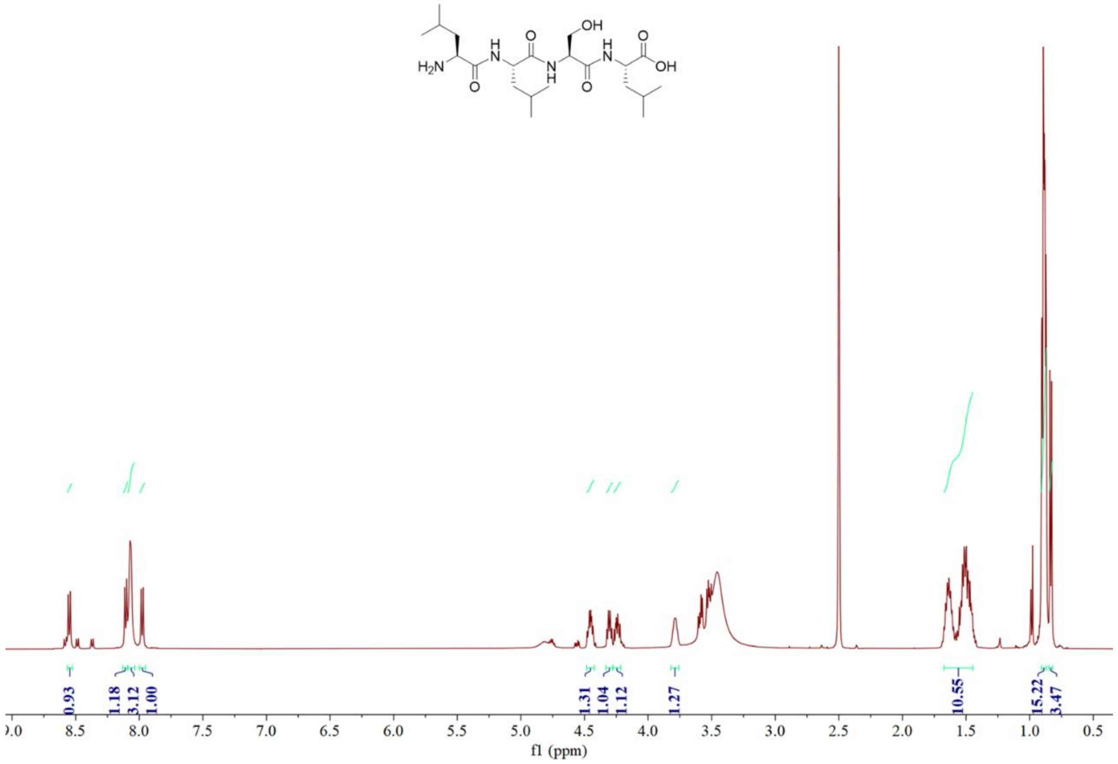

35.Compound **WLVW**: ^{1}H NMR (500 MHZ, DMSO-d6) δ 11.00 (d, *J* = 2.5 Hz, 1H), 10.83 (d, *J* = 2.4 Hz, 1H), 8.75 (d, *J* = 8.0 Hz, 1H), 8.19 (d, *J* = 7.4 Hz, 1H), 7.94 (dd, *J* = 13.6, 7.2 Hz, 4H), 7.74 (d, *J* = 7.9 Hz, 1H), 7.51 (d, *J* = 7.9 Hz, 1H), 7.37 (d, J = 8.1 Hz, 1H), 7.33 (d, *J* = 8.0 Hz, 1H), 7.21 (d, *J* = 2.5 Hz, 1H), 7.14 (d, *J* = 2.4 Hz, 1H), 7.04 (m, 4H), 4.48 (m, 2H), 4.27 (dd, *J* = 9.0, 6.8 Hz, 1H), 4.04 (m, 1H), 3.25 (dd, *J* = 14.9, 4.5 Hz, 1H), 3.15 (dd, *J* = 14.8, 5.8 Hz, 1H), 3.02 (m, 2H), 1.99 (m, 1H), 1.64 (m, 1H), 0.90 (d, *J* = 6.6 Hz, 3H), 0.85 (m, 9H).

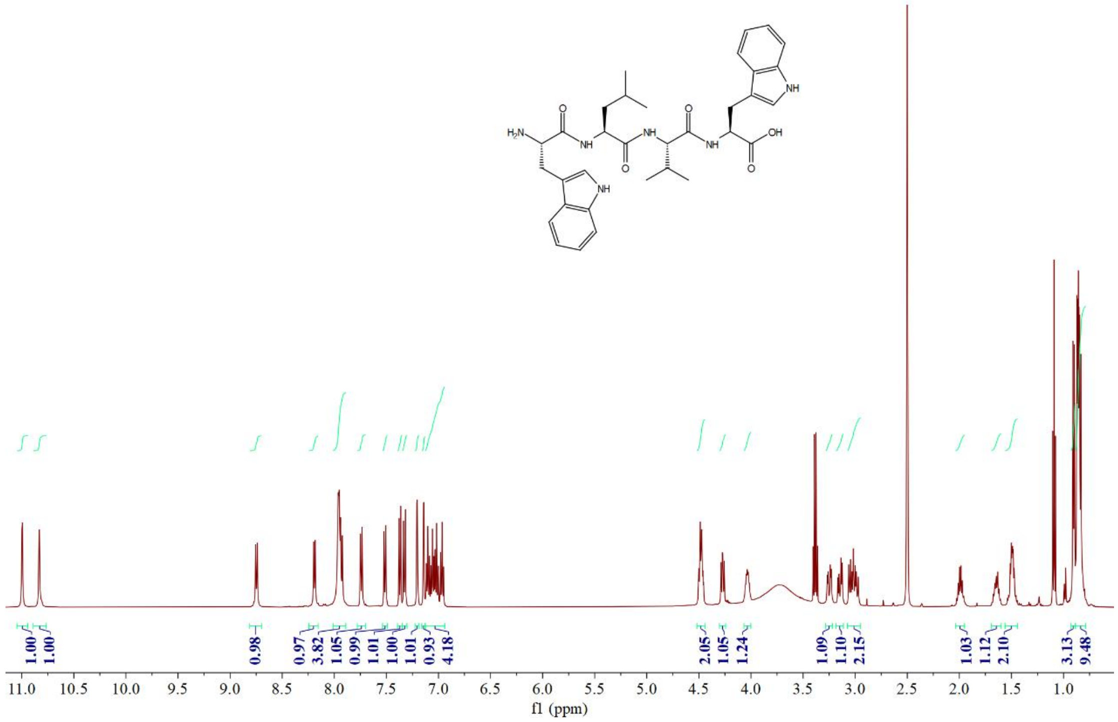

36.Compound **LWWF**: ^{1}H NMR (500 MHZ, DMSO-d6) δ 8.60 (d, J = 8.1 Hz, 1H), 8.25 (d, J = 7.7 Hz, 1H), 8.15 (d, J = 7.9 Hz, 1H), 7.99 (s, 2H), 7.64 (t, J = 8.8 Hz, 1H), 7.55 (d, J = 7.8 Hz, 1H), 7.32 (t, J = 7.4 Hz, 2H), 7.23 (dd, J = 12.5, 7.0 Hz, 4H), 7.17 (t, J = 7.0 Hz, 1H), 7.09 (d, J = 7.8 Hz, 2H), 7.05 (td, J = 7.6, 3.5 Hz, 2H), 6.95 (t, J = 7.3 Hz, 2H), 4.61 (dt, J = 12.9, 7.7 Hz, 2H), 4.47 (dd, J = 13.5, 7.7 Hz, 1H), 3.69 (s, 1H), 3.14 – 3.04 (m, 3H), 2.97 – 2.88 (m, 3H), 1.59 (dd, J = 14.0, 6.9 Hz, 1H), 1.52 – 1.43 (m, 2H), 0.86 – 0.81 (m, 6H).

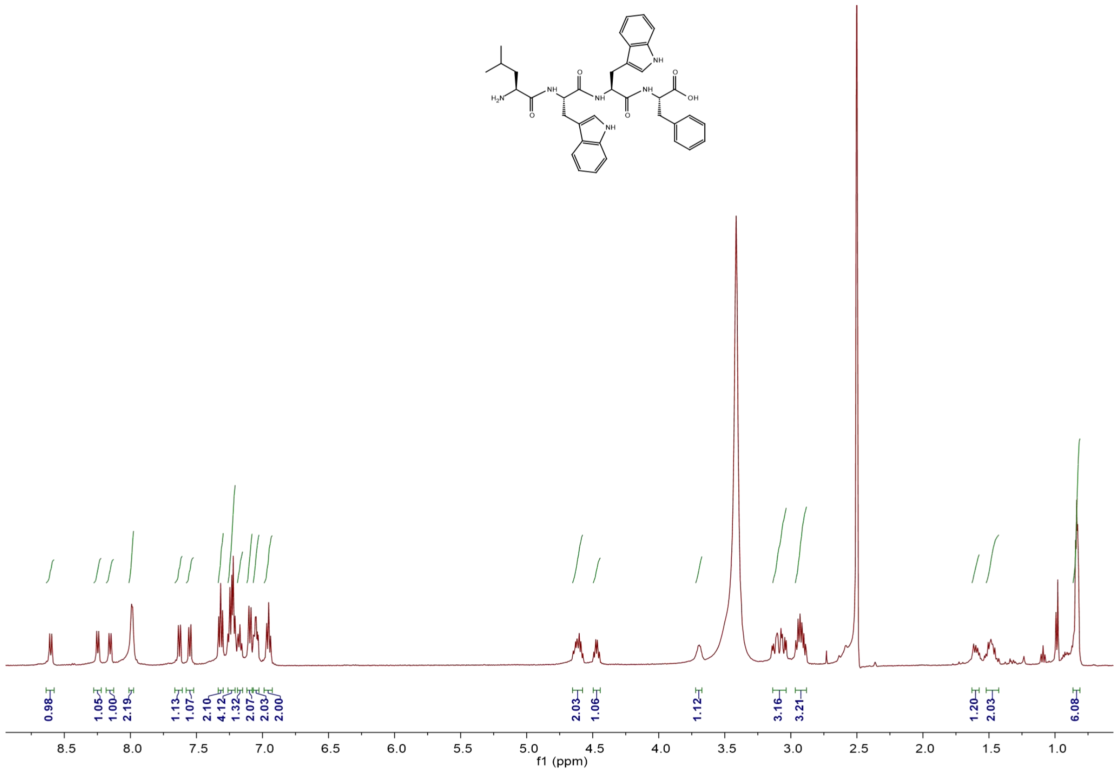

37.Compound **VFVF**: ^{1}H NMR (500 MHz, DMSO) δ 8.51 (d, *J* = 7.9 Hz, 1H), 8.23 (d, *J* = 7.7 Hz, 1H), 8.11 (d, *J* = 9.1 Hz, 1H), 7.96 (d, *J* = 3.8 Hz, 2H), 7.29 – 7.17 (m, 10H), 4.69 (td, *J* = 9.1, 4.6 Hz, 1H), 4.46 – 4.42 (m, 1H), 4.23 – 4.19 (m, 1H), 3.61 – 3.55 (m, 1H), 3.06 (dd, *J* = 14.0, 5.1 Hz, 1H), 2.94 – 2.86 (m, 2H), 2.76 (dd, *J* = 14.0, 10.0 Hz, 1H), 2.10 (dt, *J* = 13.5, 6.8 Hz, 1H), 1.93 (dq, *J* = 13.3, 6.6 Hz, 1H), 0.92 (d, *J* = 6.9 Hz, 3H), 0.87 – 0.78 (m, 9H).

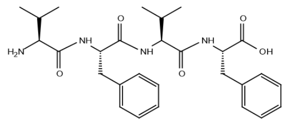

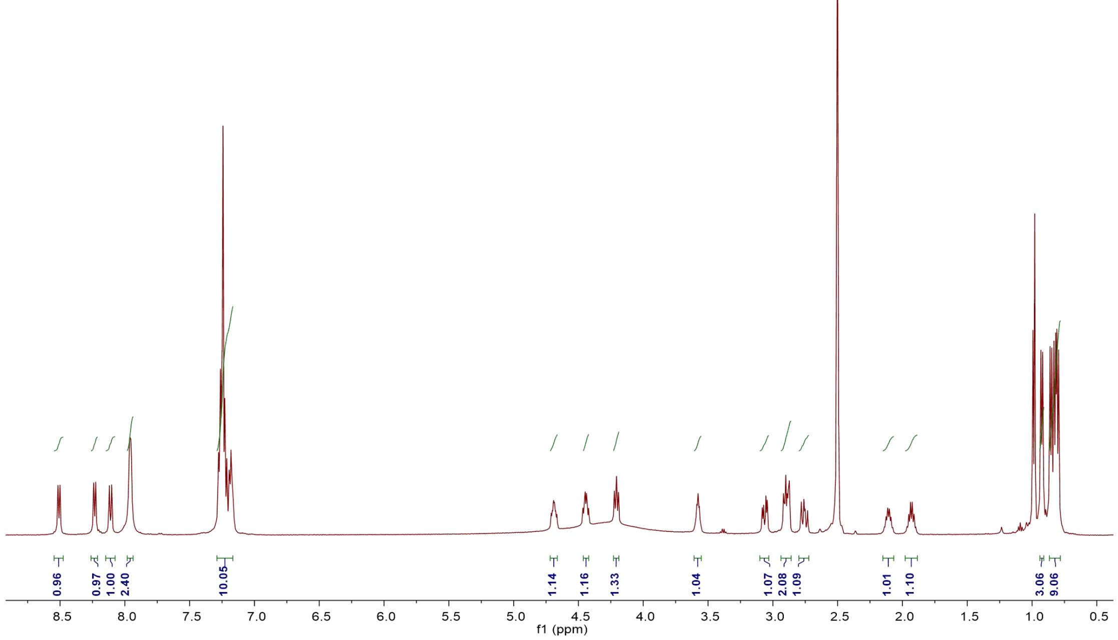

38.Compound **MMVV**: ^{1}H NMR (500 MHz, DMSO) δ 8.64 (d, *J* = 7.7 Hz, 1H), 8.16 (s, 2H), 8.03 (d, *J* = 8.8 Hz, 1H), 7.97 (d, *J* = 8.2 Hz, 1H), 4.49 (dd, *J* = 13.2, 8.0 Hz, 1H), 4.31 – 4.25 (m, 1H), 4.13 (dd, *J* = 7.9, 6.0 Hz, 1H), 3.88 (d, *J* = 4.3 Hz, 1H), 2.04 (s, 6H), 2.02 – 1.85 (m, 5H), 1.85 – 1.74 (m, 1H), 0.99 (d, *J* = 6.7 Hz, 2H), 0.95 – 0.78 (m, 14H).

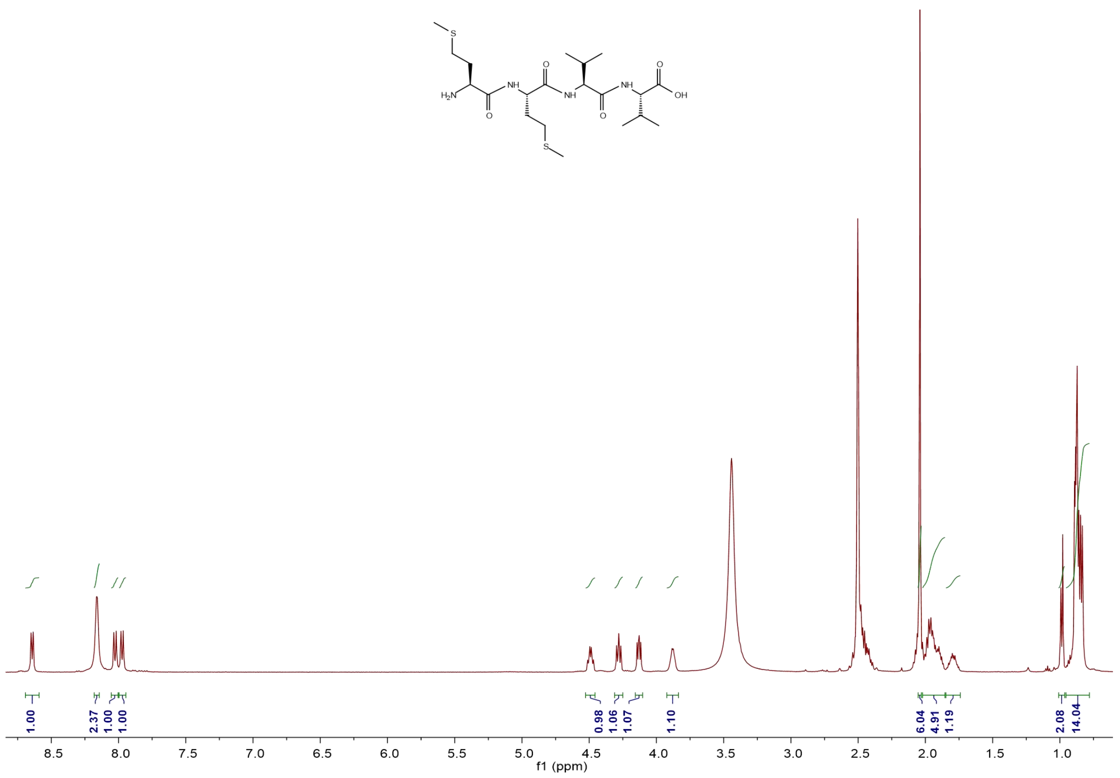

39.Compound **LVAI**: ^{1}H NMR (500 MHz, DMSO) δ 8.49 (d, J = 8.8 Hz, 1H), 8.14 (d, J = 7.3 Hz, 1H), 8.09 (s, 2H), 7.90 (d, J = 8.4 Hz, 1H), 4.41 – 4.34 (m, 1H), 4.28 – 4.23 (m, 1H), 4.20 – 4.15 (m, 1H), 3.89 (d, J = 5.2 Hz, 1H), 1.97 (dq, J = 13.4, 6.6 Hz, 1H), 1.77 (s, 1H), 1.62 (dt, J = 13.1, 6.4 Hz, 1H), 1.52 (t, J = 6.4 Hz, 2H), 1.43 – 1.36 (m, 1H), 1.19 (t, J = 7.6 Hz, 4H), 0.90 – 0.83 (m, 18H).

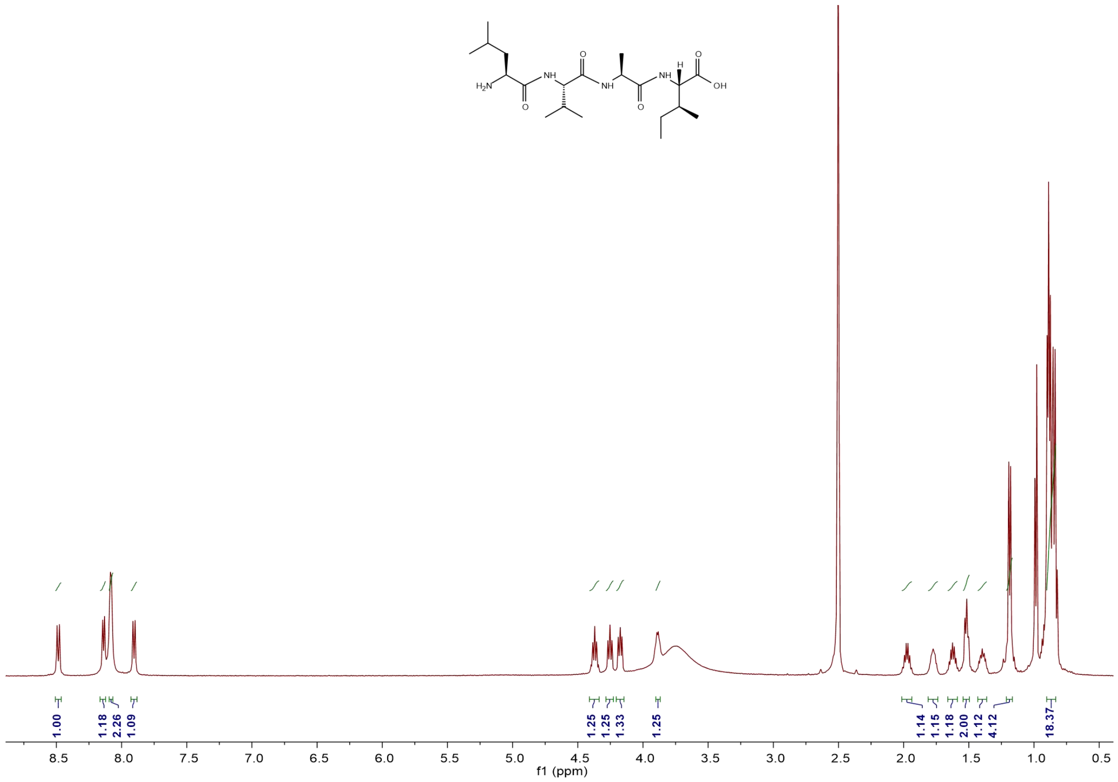

40.Compound **WAVV**: ^{1}H NMR (500 MHz, DMSO-d6) δ 8.59 (d, J = 7.3 Hz, 1H), 7.96 (d, J = 8.9 Hz, 1H), 7.88 (d, J = 8.2 Hz, 1H), 7.65 (d, J = 7.9 Hz, 1H), 7.35 (d, J = 8.1 Hz, 1H), 7.20 (d, J = 2.1 Hz, 1H), 7.08 (t, J = 7.5 Hz, 1H), 6.99 (t, J = 7.5 Hz, 1H), 4.46 (p, J = 7.0 Hz, 1H), 4.28 (dd, J = 8.7, 6.9 Hz, 1H), 4.11 (dd, J = 8.1, 5.6 Hz, 1H), 3.84 (dd, J = 8.4, 4.6 Hz, 1H), 3.18 (dd, J = 14.6, 4.3 Hz, 1H), 2.93 (dd, J = 14.7, 8.7 Hz, 1H), 2.08 – 1.94 (m, 2H), 1.22 (d, J = 7.0 Hz, 3H), 0.90 – 0.83 (m, 12H).

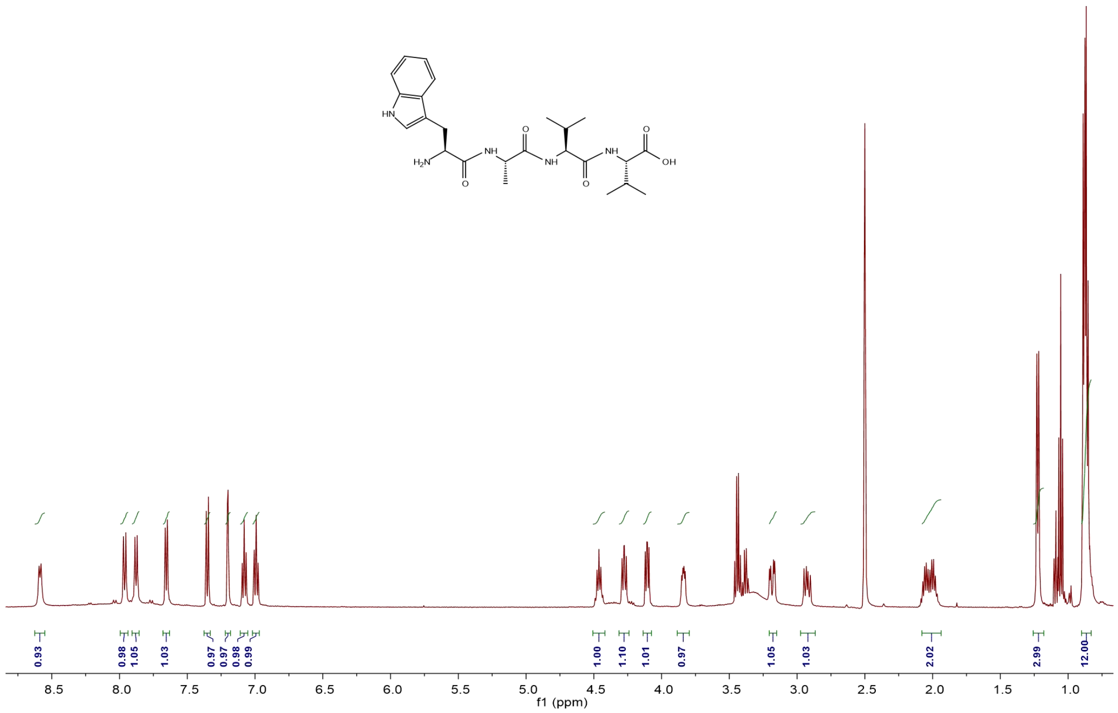

41.Compound **MMLL:** ^{1}H NMR (500 MHz, DMSO-d6) δ 8.59 (d, J = 7.9 Hz, 1H), 8.13 (dd, J = 29.5, 8.2 Hz, 2H), 4.42 (q, J = 7.7 Hz, 1H), 4.35 (q, J = 7.8 Hz, 1H), 4.22 (td, J = 9.1, 5.0 Hz, 1H), 3.83 (t, J = 6.2 Hz, 1H), 2.44 (ddd, J = 17.1, 8.6, 4.7 Hz, 4H), 2.13 – 1.81 (m, 10H), 1.62 (dd, J = 13.4, 6.8 Hz, 2H), 1.55 – 1.41 (m, 4H), 0.90 – 0.81 (m, 12H).

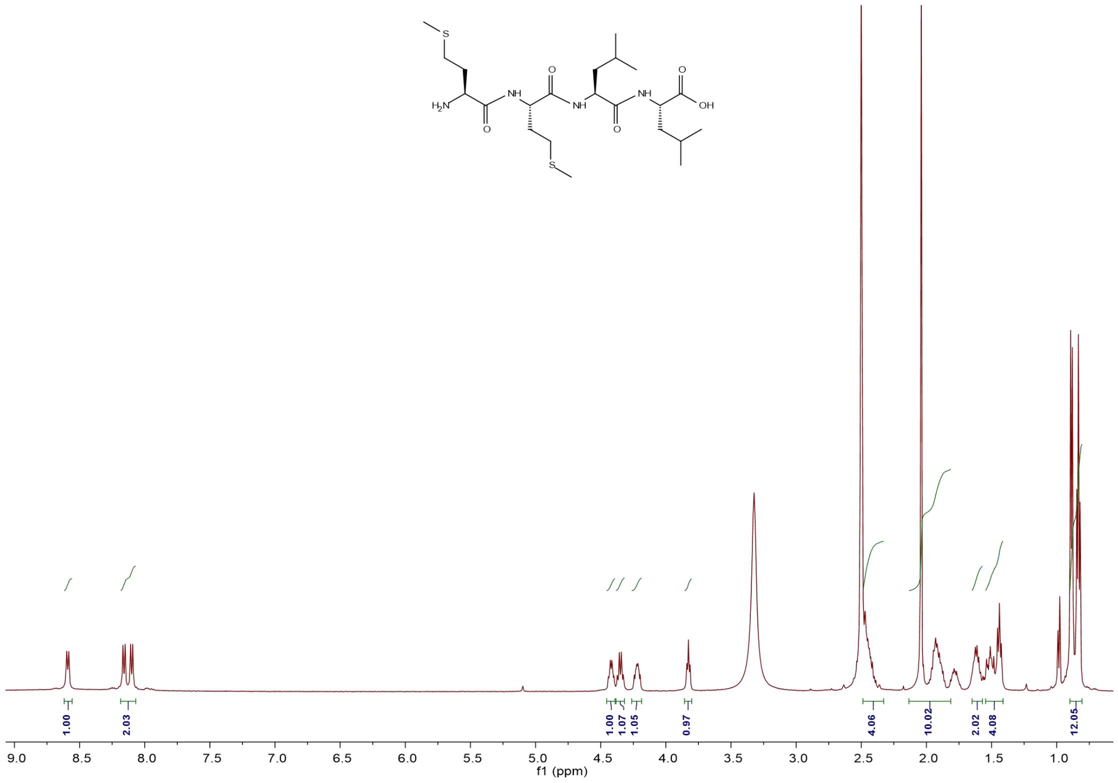

42.Compound **SWYF:** ^{1}H NMR (500 MHz, DMSO-d6) δ 9.16 (s, 1H), 8.54 (d, *J* = 7.9 Hz, 1H), 8.31 (d, *J* = 7.5 Hz, 1H), 8.04 (d, *J* = 7.1 Hz, 3H), 7.61 (d, *J* = 7.8 Hz, 1H), 7.32 – 7.20 (m, 6H), 7.09 – 6.94 (m, 5H), 6.63 (d, *J* = 8.2 Hz, 2H), 5.48 (s, 1H), 4.56 (dd, *J* = 13.0, 8.6 Hz, 1H), 4.51 – 4.44 (m, 2H), 3.74 (d, *J* = 7.8 Hz, 2H), 3.12 – 3.04 (m, 2H), 2.96 – 2.83 (m, 4H), 2.67 (dd, *J* = 14.0, 9.2 Hz, 1H).

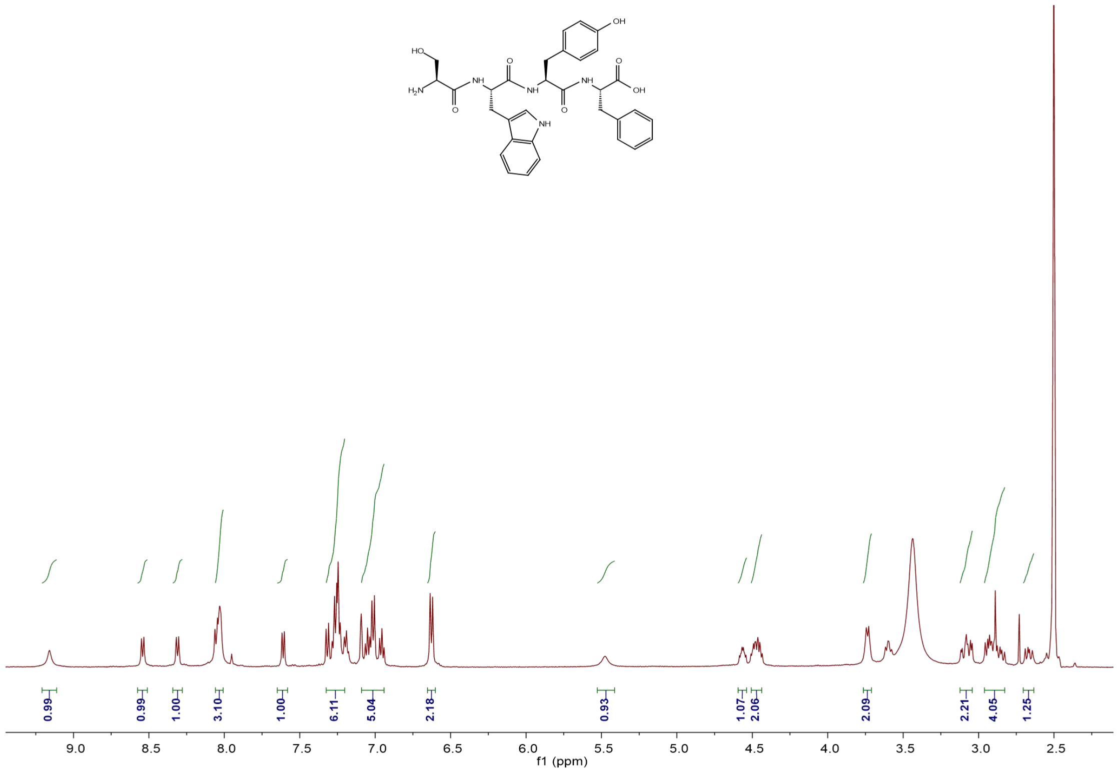

43.Compound **WWMF**: ^{1}H NMR (500 MHz, DMSO-d6) δ 8.84 (d, J = 7.8 Hz, 1H), 8.32 (d, J = 8.0 Hz, 1H), 8.20 (d, J = 7.7 Hz, 1H), 7.91 (s, 2H), 7.73 (d, J = 7.9 Hz, 1H), 7.66 (d, J = 7.9 Hz, 1H), 7.36 – 7.23 (m, 6H), 7.03 (ddt, J = 52.0, 31.2, 7.4 Hz, 5H), 4.69 (td, J = 8.3, 4.9 Hz, 1H), 4.46 (qd, J = 8.6, 5.0 Hz, 2H), 3.97 (s, 1H), 3.27 (dd, J = 15.0, 4.2 Hz, 2H), 3.16 – 2.91 (m, 6H), 2.02 (s, 5H).

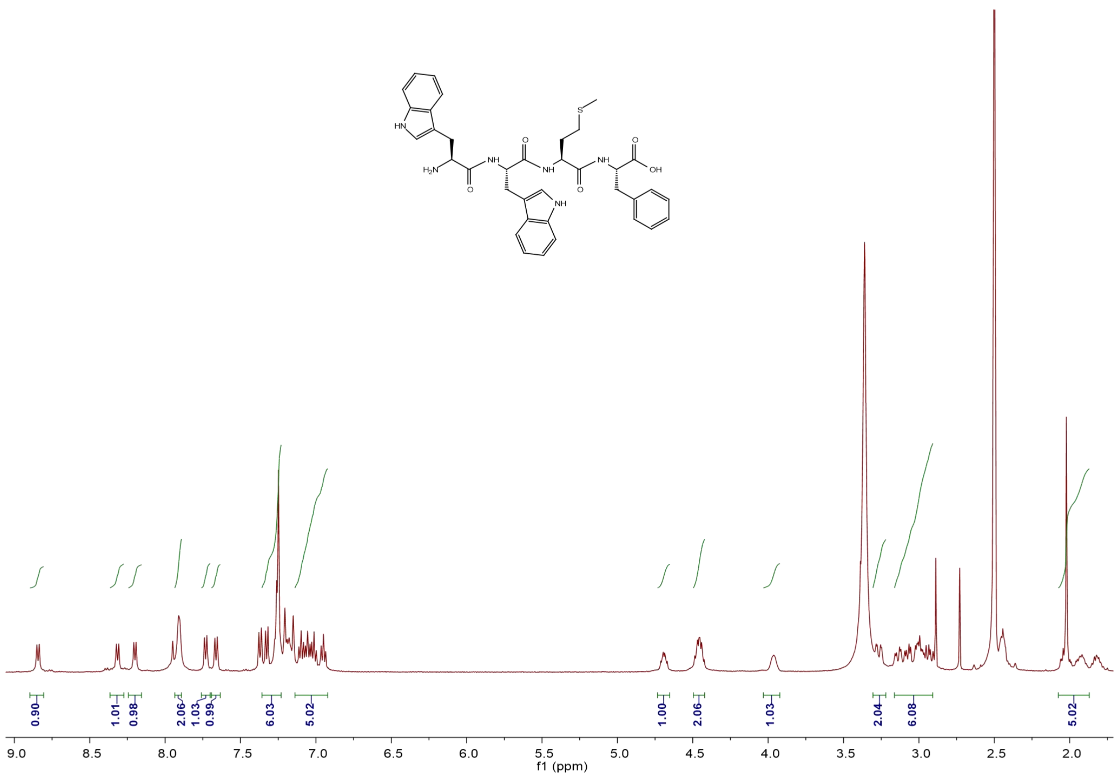

44.Compound **SYFF**: ^{1}H NMR (500 MHz, DMSO-d6) δ 8.44 (d, J = 8.3 Hz, 1H), 8.36 (d, J = 7.8 Hz, 1H), 8.17 (d, J = 8.3 Hz, 1H), 7.30 – 7.19 (m, 10H), 6.99 (d, J = 8.0 Hz, 2H), 6.61 (d, J = 8.0 Hz, 2H), 4.56 (td, J = 8.8, 4.5 Hz, 1H), 4.45 (dtd, J = 13.4, 8.9, 8.4, 4.9 Hz, 2H), 3.79 – 3.64 (m, 2H), 3.56 (dd, J = 11.0, 7.0 Hz, 1H), 3.10 – 2.99 (m, 2H), 2.98 – 2.71 (m, 4H).

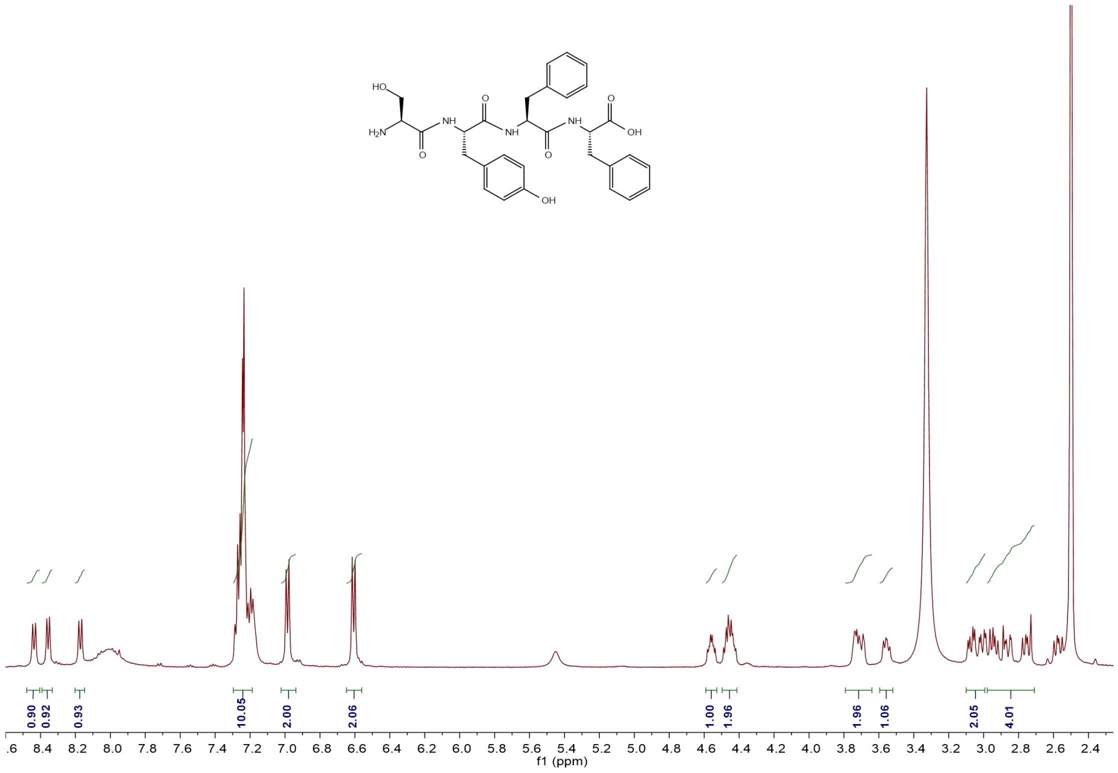

45.Compound **WFMM**: ^{1}H NMR (500 MHz, DMSO-d6) δ 8.85 (d, J = 8.0 Hz, 1H), 8.39 (d, J = 7.8 Hz, 1H), 8.28 (d, J = 7.8 Hz, 1H), 7.92 (d, J = 5.5 Hz, 1H), 7.71 (d, J = 7.9 Hz, 1H), 7.39 – 7.23 (m, 6H), 7.10 (t, J = 7.6 Hz, 1H), 7.01 (t, J = 7.4 Hz, 1H), 4.67 (td, J = 8.5, 4.4 Hz, 1H), 4.44 (td, J = 7.9, 5.3 Hz, 1H), 4.35 (td, J = 8.7, 4.4 Hz, 1H), 3.98 (dd, J = 9.6, 5.1 Hz, 1H), 3.26 (dd, J = 15.0, 4.4 Hz, 2H), 3.10 – 2.99 (m, 2H), 2.92 – 2.71 (m, 2H), 2.05 (d, J = 3.7 Hz, 6H), 2.00 – 1.92 (m, 2H), 1.91 – 1.84 (m, 2H).

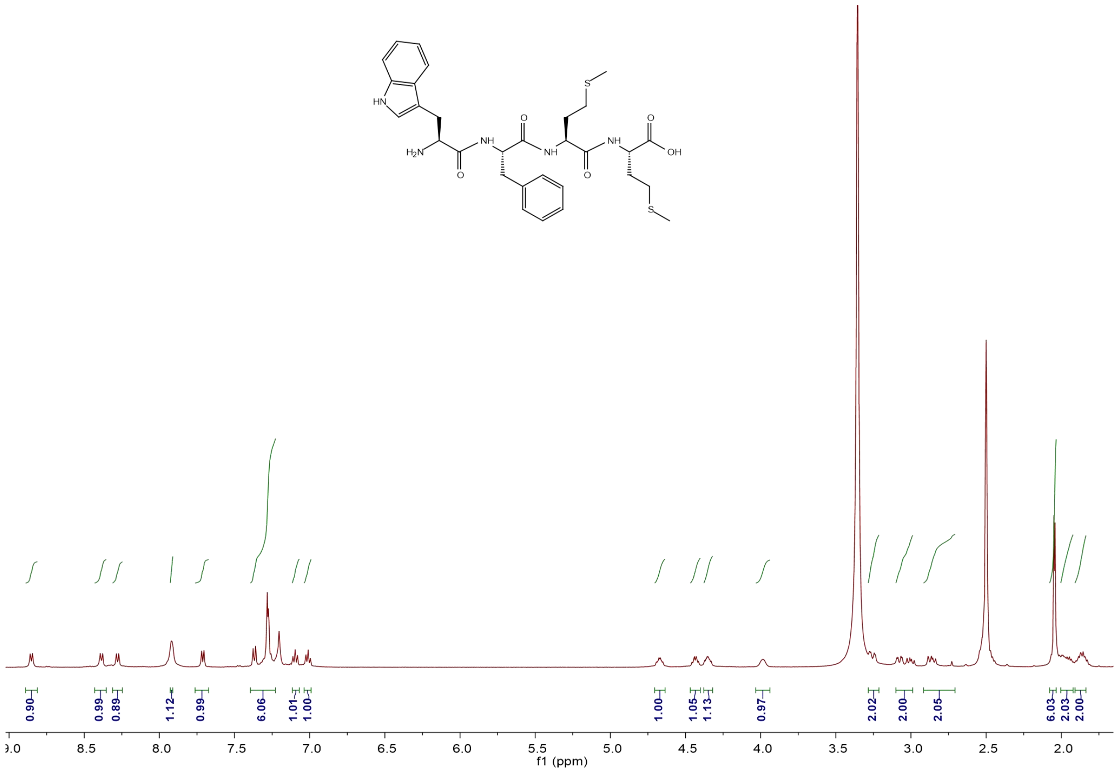

46.Compound **CWFS**: ^{1}H NMR (500 MHz, DMSO-d6) δ 8.52 (d, J = 7.9 Hz, 1H), 8.31 (dd, J = 8.1, 3.2 Hz, 2H), 7.65 (d, J = 7.9 Hz, 1H), 7.30 (dd, J = 19.5, 8.0 Hz, 4H), 7.14 – 6.93 (m, 5H), 4.69 (dt, J = 8.8, 4.5 Hz, 1H), 4.60 (td, J = 8.6, 4.6 Hz, 1H), 3.76 (dd, J = 11.0, 5.3 Hz, 2H), 3.66 (dt, J = 10.9, 4.8 Hz, 2H), 3.10 (td, J = 15.3, 14.9, 4.5 Hz, 3H), 2.98 – 2.85 (m, 3H).

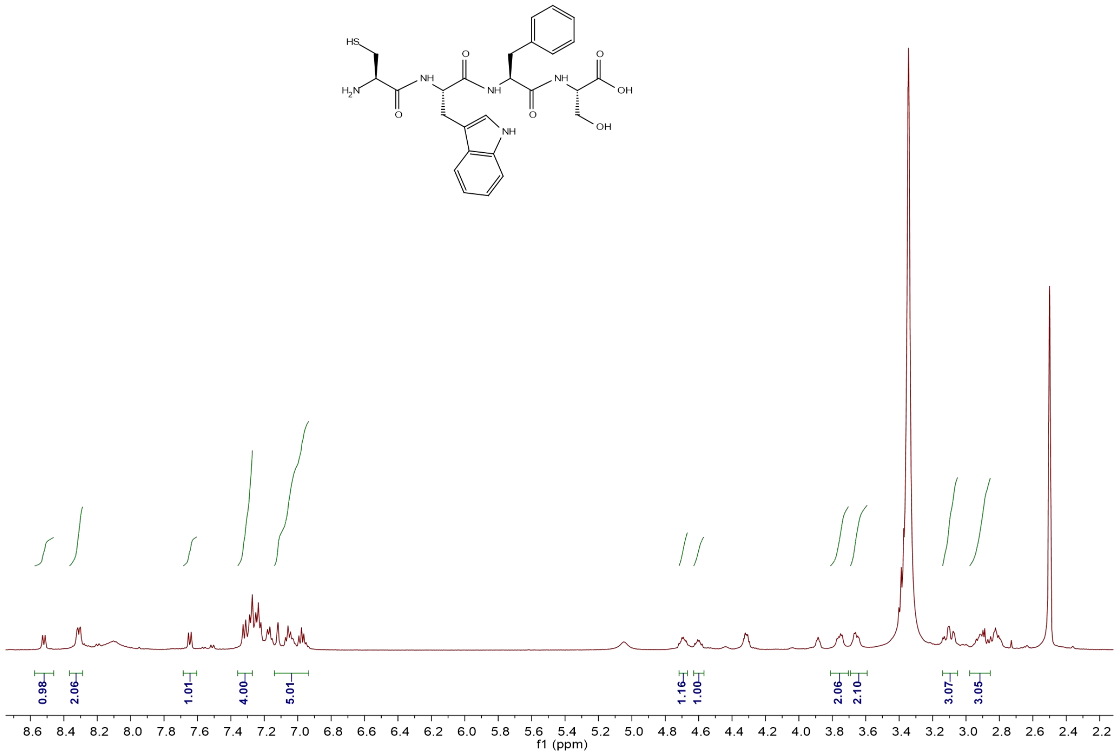

47.Compound **PWYW**: ^{1}H NMR (500 MHz, DMSO-d6) δ 9.15 (s, 1H), 8.54 (d, J = 8.5 Hz, 1H), 8.29 (d, J = 7.6 Hz, 1H), 8.09 (d, J = 8.2 Hz, 1H), 7.58 (dd, J = 27.0, 7.9 Hz, 2H), 7.32 (dd, J = 12.8, 8.0 Hz, 2H), 7.17 (s, 1H), 7.10 – 6.93 (m, 7H), 6.60 (d, J = 8.0 Hz, 2H), 4.58 (td, J = 9.0, 4.6 Hz, 1H), 4.51 (q, J = 6.5 Hz, 2H), 3.96 (d, J = 7.4 Hz, 1H), 3.24 – 3.03 (m, 6H), 2.96 – 2.86 (m, 2H), 2.15 (s, 1H), 1.70 (qd, J = 14.4, 13.9, 6.4 Hz, 3H).

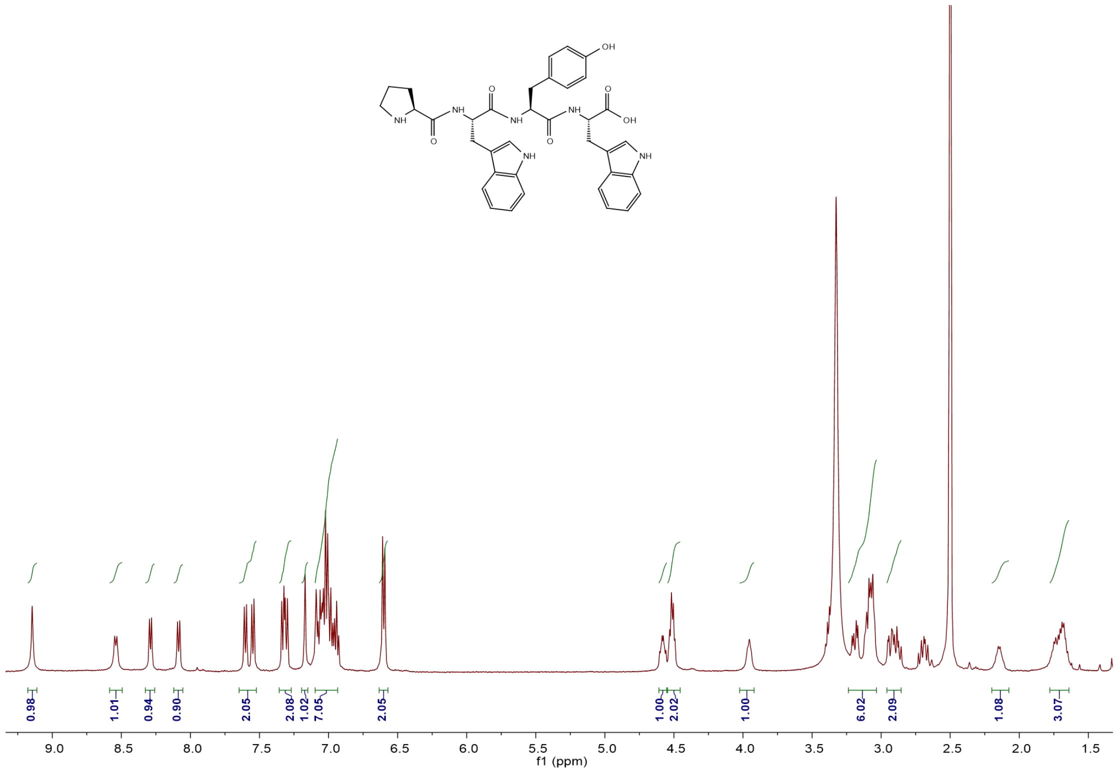

48.Compound **KVWL**: ^{1}H NMR (500 MHz, DMSO-d6) δ 8.49 (d, J = 9.2 Hz, 1H), 8.29 (dd, J = 18.4, 8.1 Hz, 2H), 7.70 (d, J = 7.8 Hz, 1H), 7.32 (d, J = 8.0 Hz, 1H), 7.19 (d, J = 2.3 Hz, 1H), 7.10 – 7.04 (m, 1H), 6.99 (t, J = 7.4 Hz, 1H), 4.65 – 4.57 (m, 1H), 4.37 (dd, J = 9.2, 5.4 Hz, 1H), 4.30 (ddd, J = 10.5, 8.1, 4.6 Hz, 1H), 3.83 (t, J = 6.4 Hz, 1H), 3.09 (dd, J = 14.8, 4.7 Hz, 1H), 2.94 – 2.87 (m, 1H), 2.72 (dd, J = 10.4, 5.5 Hz, 2H), 2.02 (dq, J = 13.3, 6.7 Hz, 1H), 1.72 – 1.61 (m, 3H), 1.52 (dtq, J = 13.6, 9.5, 4.3 Hz, 4H), 1.32 (q, J = 8.1 Hz, 2H), 0.89 – 0.76 (m, 12H).

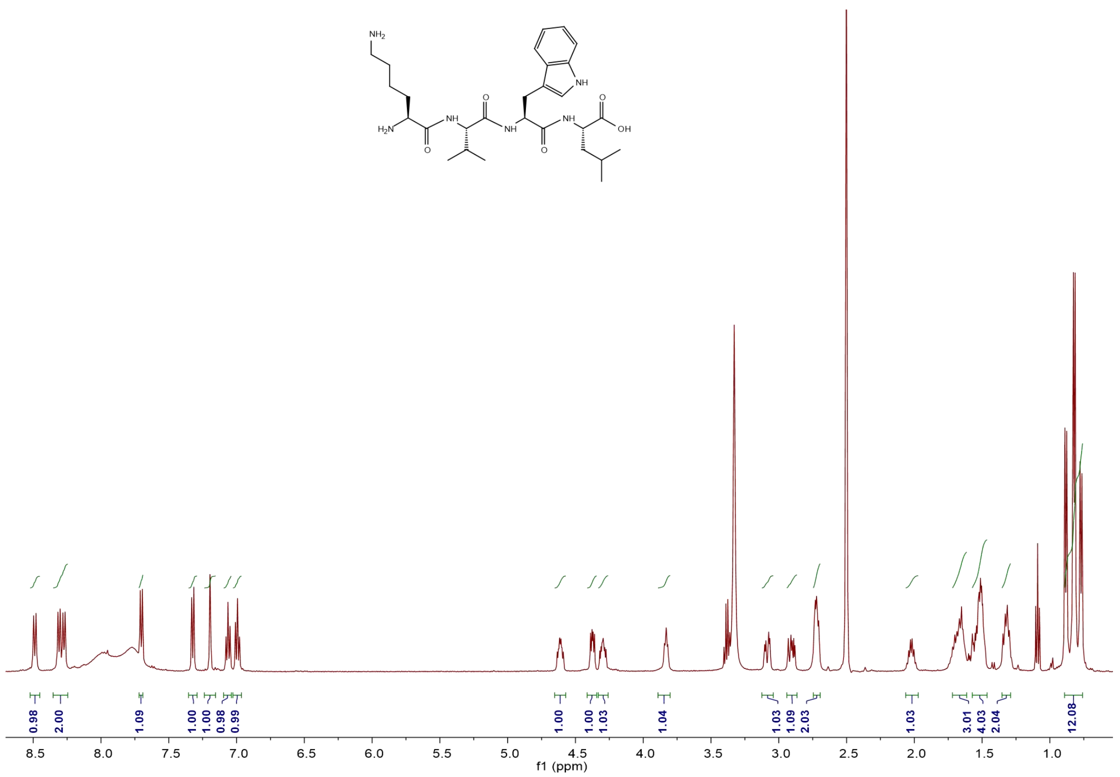

49.Compound **FYAY**: ^{1}H NMR (500 MHz, DMSO) δ 9.20 (s, 2H), 8.56 (d, *J* = 7.9 Hz, 1H), 8.24 (d, *J* = 7.4 Hz, 1H), 8.00 (t, *J* = 16.5 Hz, 1H), 7.36 – 7.16 (m, 5H), 7.01 (dt, *J* = 34.9, 17.4 Hz, 4H), 6.61 (dd, *J* = 29.9, 7.6 Hz, 4H), 4.53 (dd, *J* = 12.5, 8.7 Hz, 1H), 4.42 – 4.26 (m, 2H), 3.87 (s, 1H), 3.08 (dd, *J* = 14.0, 3.9 Hz, 1H), 2.87 (dtd, *J* = 22.1, 14.0, 6.7 Hz, 4H), 2.67 (dt, *J* = 21.0, 10.5 Hz, 1H), 1.21 (d, *J* = 7.0 Hz, 3H).

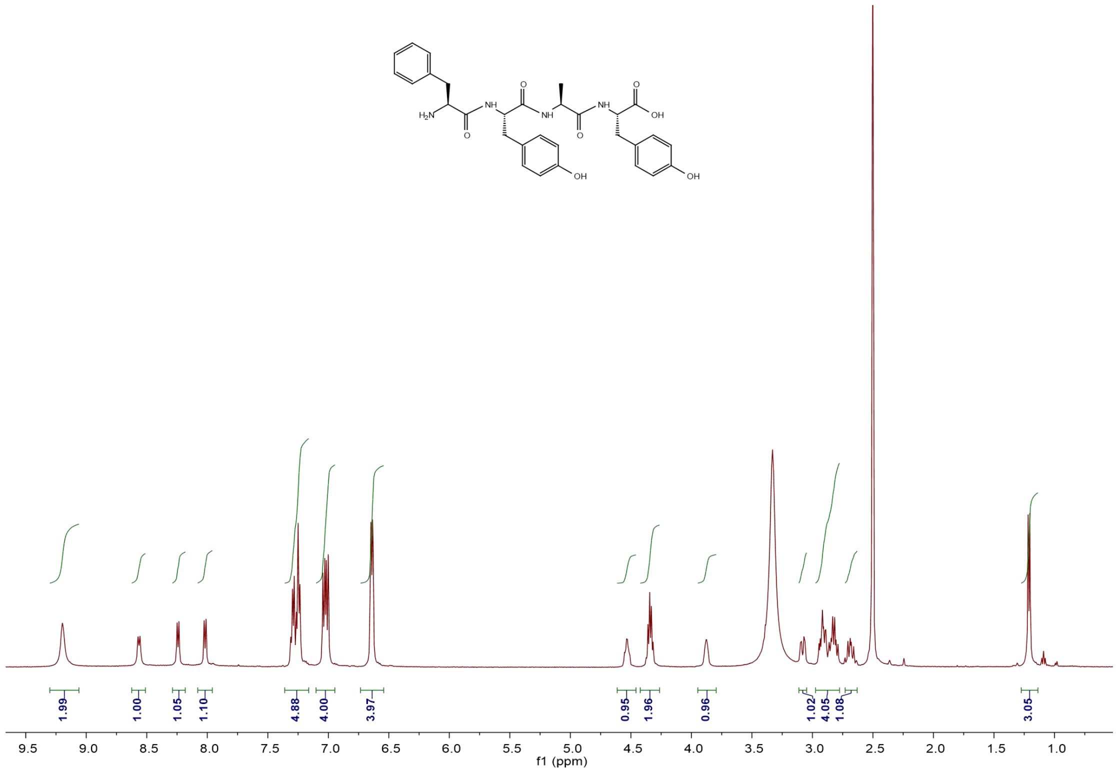

50.Compound **KLLF**: 1H NMR (500 MHz, DMSO-d6) δ 8.57 (d, J = 8.4 Hz, 1H), 8.17 (d, J = 8.3 Hz, 1H), 8.04 – 7.92 (m, 2H), 7.30 – 7.14 (m, 5H), 4.39 (q, J = 8.4 Hz, 2H), 4.28 (q, J = 8.7 Hz, 1H), 3.78 (t, J = 6.1 Hz, 1H), 3.05 (dd, J = 13.9, 5.1 Hz, 1H), 2.94 – 2.88 (m, 1H), 2.76– 2.69 (m, 2H), 1.69 (dt, J = 13.1, 6.7 Hz, 2H), 1.55 (dq, J = 21.7, 7.2 Hz, 4H), 1.41 (ddt, J = 19.9, 13.7, 6.9 Hz, 4H), 1.31 (dt, J = 15.2, 7.4 Hz, 2H), 0.98 – 0.77 (m, 12H).

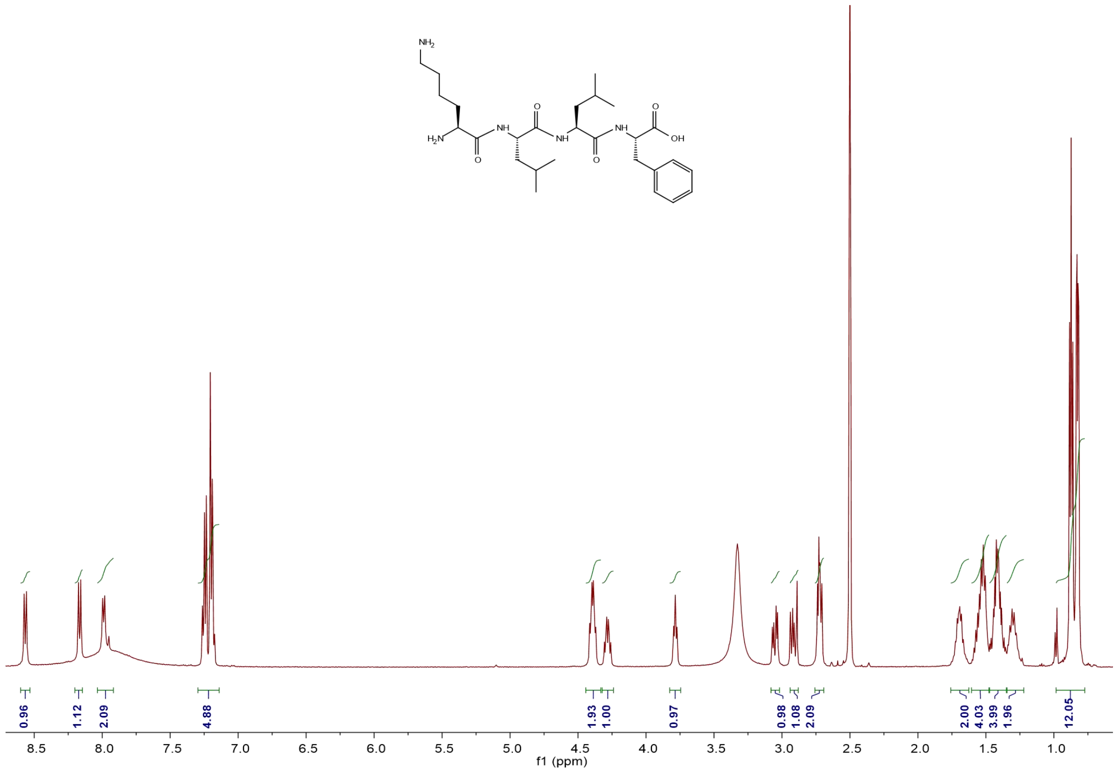

51.Compound **SAWF**: ^{1}H NMR (500 MHz, DMSO-d6) δ 8.51 (d, J = 7.6 Hz, 1H), 8.23 (d, J = 7.7 Hz, 1H), 8.05 (d, J = 8.1 Hz, 1H), 7.59 (d, J = 7.9 Hz, 1H), 7.31 (d, J = 8.1 Hz, 1H), 7.28 – 7.19 (m, 5H), 7.11 (d, J = 2.3 Hz, 1H), 7.08 – 7.03 (m, 1H), 6.97 (dd, J = 14.9, 1.0 Hz, 1H), 4.55 (td, J = 8.4, 4.9 Hz, 1H), 4.44 (td, J = 8.0, 5.3 Hz, 1H), 4.32 (p, J = 7.1 Hz, 1H), 3.81 (dd, J = 6.7, 4.3 Hz, 1H), 3.69 (dd, J = 11.3, 4.3 Hz, 1H), 3.60 (dd, J = 11.3, 6.8 Hz, 1H), 3.08 (ddd, J = 23.0, 14.4, 5.1 Hz, 2H), 2.91 (ddd, J = 18.5, 14.4, 8.7 Hz, 2H), 1.16 (d, J = 7.0 Hz, 3H).

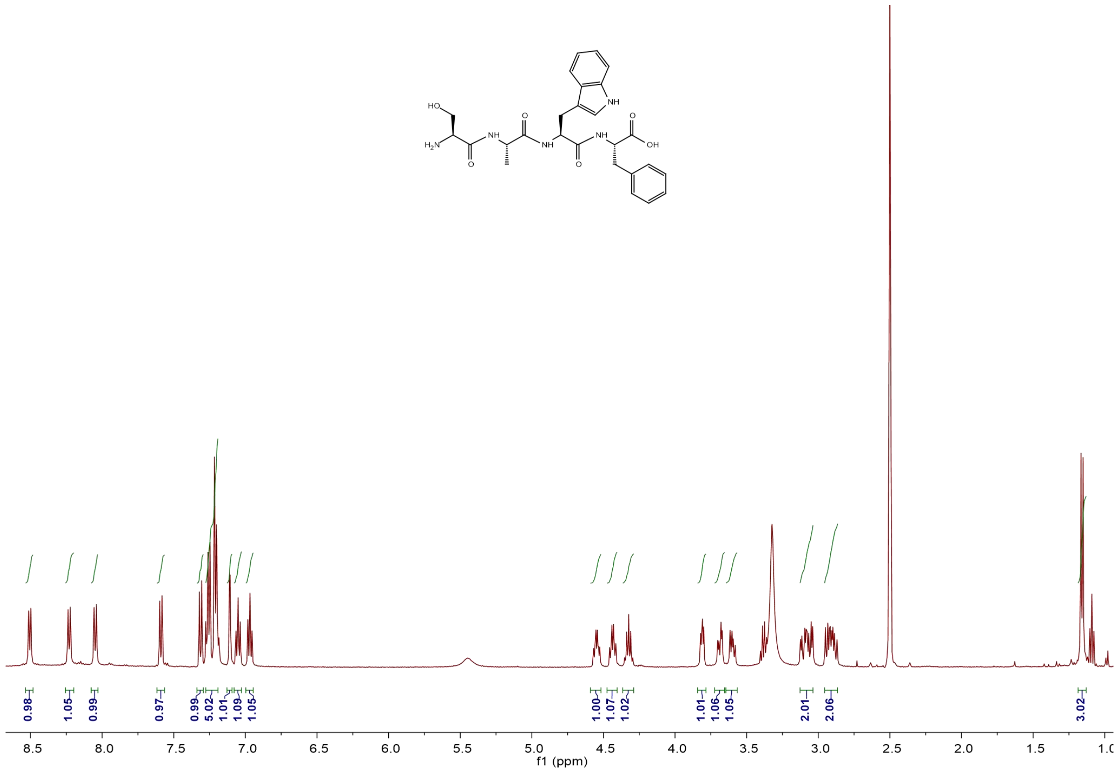

52.Compound **TGFF**: ^{1}H NMR (500 MHz, DMSO-d6) δ 8.56 (t, J = 5.4 Hz, 1H), 8.42 (d, J = 7.8 Hz, 1H), 8.20 (d, J = 8.5 Hz, 1H), 7.31 – 7.15 (m, 10H), 4.56 (td, J = 9.4, 4.0 Hz, 1H), 4.48 –4.38 (m, 1H), 3.87 – 3.79 (m, 1H), 3.79 – 3.65 (m, 2H), 3.55 (d, J = 6.6 Hz, 1H), 3.08 (dd, J = 13.9, 5.1 Hz, 1H), 3.00 (dd, J = 13.8, 3.9 Hz, 1H), 2.92 (dd, J = 13.9, 9.0 Hz, 1H), 2.69 (dd, J = 13.7, 10.0 Hz, 1H), 1.11 (dd, J = 14.9, 6.6 Hz, 3H).

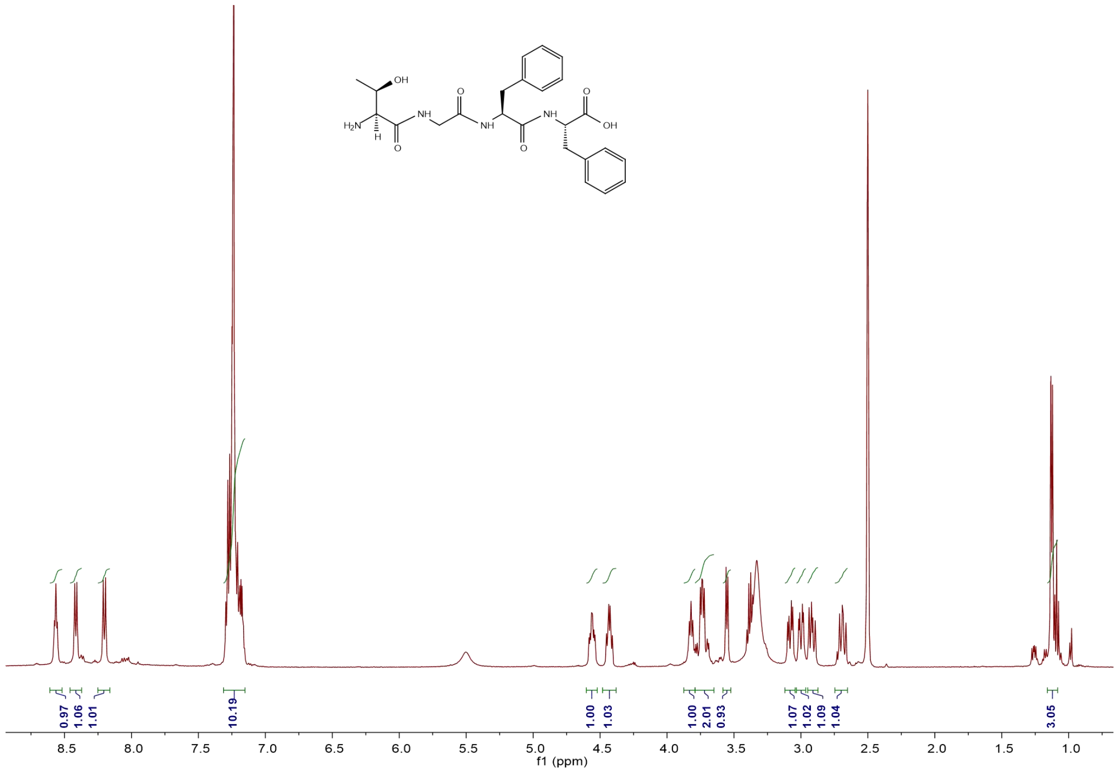

53.Compound **VAFY**: ^{1}H NMR (500 MHz, DMSO-d6) δ 8.42 (d, J = 7.7 Hz, 1H), 8.20 (d, J = 7.8 Hz, 1H), 8.10 (d, J = 8.4 Hz, 1H), 8.01 (d, J = 5.4 Hz, 2H), 7.24 – 7.13 (m, 5H), 7.01 (d, J = 8.4 Hz, 2H), 6.65 (d, J = 8.4 Hz, 2H), 4.55 (td, J = 8.9, 4.4 Hz, 1H), 4.42 – 4.30 (m, 2H), 3.38 (d, J = 7.0 Hz, 1H), 2.97 (ddd, J = 34.9, 14.0, 4.8 Hz, 2H), 2.78 (ddd, J = 28.2, 14.0, 8.9 Hz, 2H), 1.96 (h, J = 6.8 Hz, 1H), 1.17 (d, J = 7.0 Hz, 3H), 0.84 (dd, J = 6.9, 4.8 Hz, 6H).

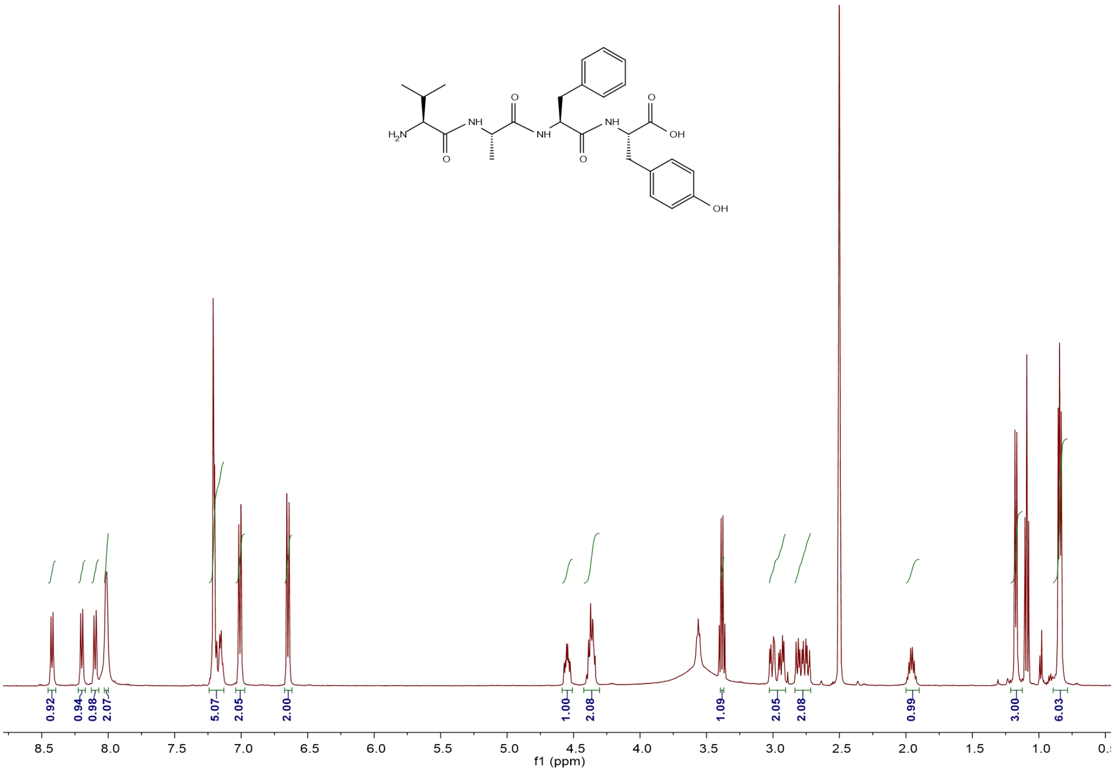

54.Compound **SSFF**: ^{1}H NMR (500 MHz, DMSO) δ 8.49 (ddd, *J* = 52.1, 36.3, 8.0 Hz, 2H), 8.04 (d, *J* = 8.1 Hz, 3H), 7.37 – 7.09 (m, 10H), 5.48 (s, 1H), 5.08 (s, 1H), 4.63 – 4.32 (m, 3H), 3.94 – 3.43 (m, 5H), 3.05 (ddd, *J* = 17.5, 14.1, 4.7 Hz, 2H), 2.96 – 2.86 (m, 1H), 2.74 (dd, *J* = 22.9, 13.2 Hz, 1H).

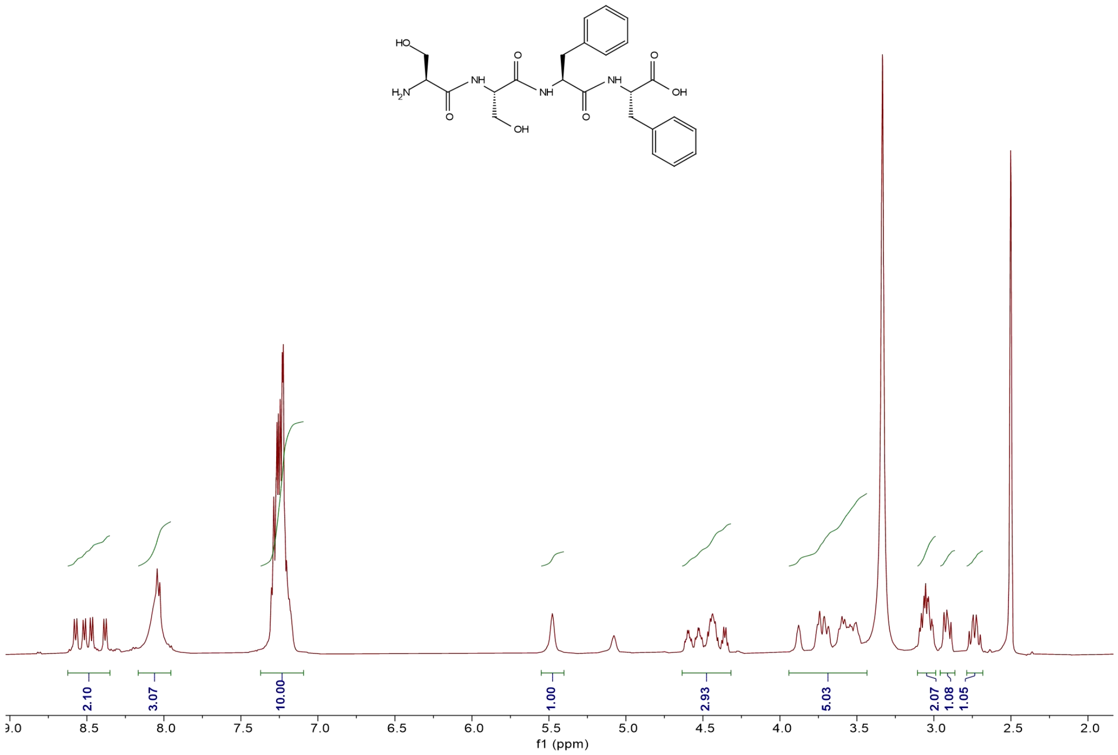

55.Compound **CTYF**: ^{1}H NMR (500 MHz, DMSO-d6) δ 8.36 (dd, J = 16.4, 8.0 Hz, 1H), 7.85 (d, J = 8.1 Hz, 1H), 7.24 (tt, J = 15.0, 7.4 Hz, 5H), 7.05 – 6.93 (m, 2H), 6.68 – 6.52 (m, 2H), 4.51 – 4.33 (m, 2H), 4.24 (dd, J = 8.2, 4.4 Hz, 1H), 4.08 – 3.85 (m, 2H), 3.05 (dd, J = 13.9, 5.9 Hz, 1H), 2.96 – 2.79 (m, 4H), 2.65 (dd, J = 14.3, 8.5 Hz, 1H), 1.04 – 0.90 (m, 3H).

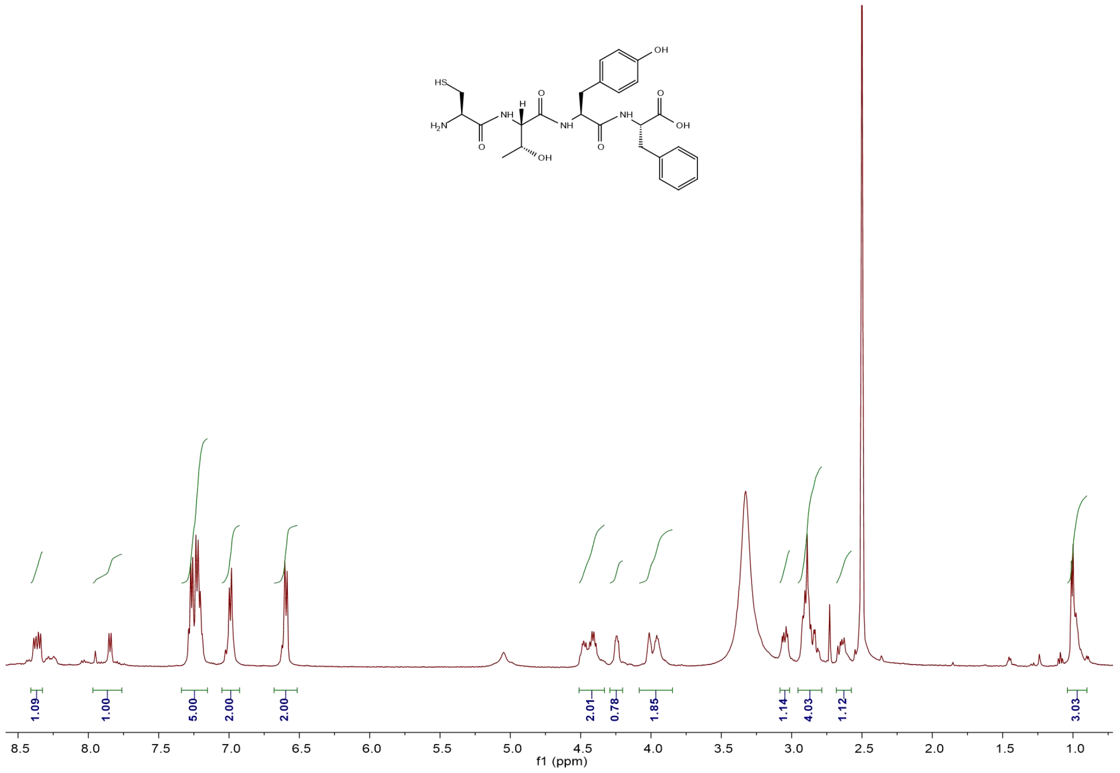

56.Compound **FSYV**: ^{1}H NMR (500 MHz, DMSO-d6) δ 8.67 (d, J = 8.0 Hz, 1H), 8.09 – 8.00 (m, 4H), 7.24 (p, J = 3.6 Hz, 5H), 7.06 (d, J = 8.4 Hz, 2H), 6.62 (d, J = 8.4 Hz, 2H), 4.57 (td, J = 8.5, 4.3 Hz, 1H), 4.41 (q, J = 6.1 Hz, 1H), 4.13 (dd, J = 8.4, 5.9 Hz, 1H), 3.62 – 3.51 (m, 3H), 3.07 (dd, J = 14.2, 4.6 Hz, 1H), 2.97 (dd, J = 14.0, 4.0 Hz, 1H), 2.86 (dd, J = 14.2, 8.6 Hz, 1H), 2.71 (dd, J = 13.9, 9.0 Hz, 1H), 2.05 (dq, J = 13.5, 6.8 Hz, 1H), 0.88 (dd, J = 6.7, 4.4 Hz, 6H).

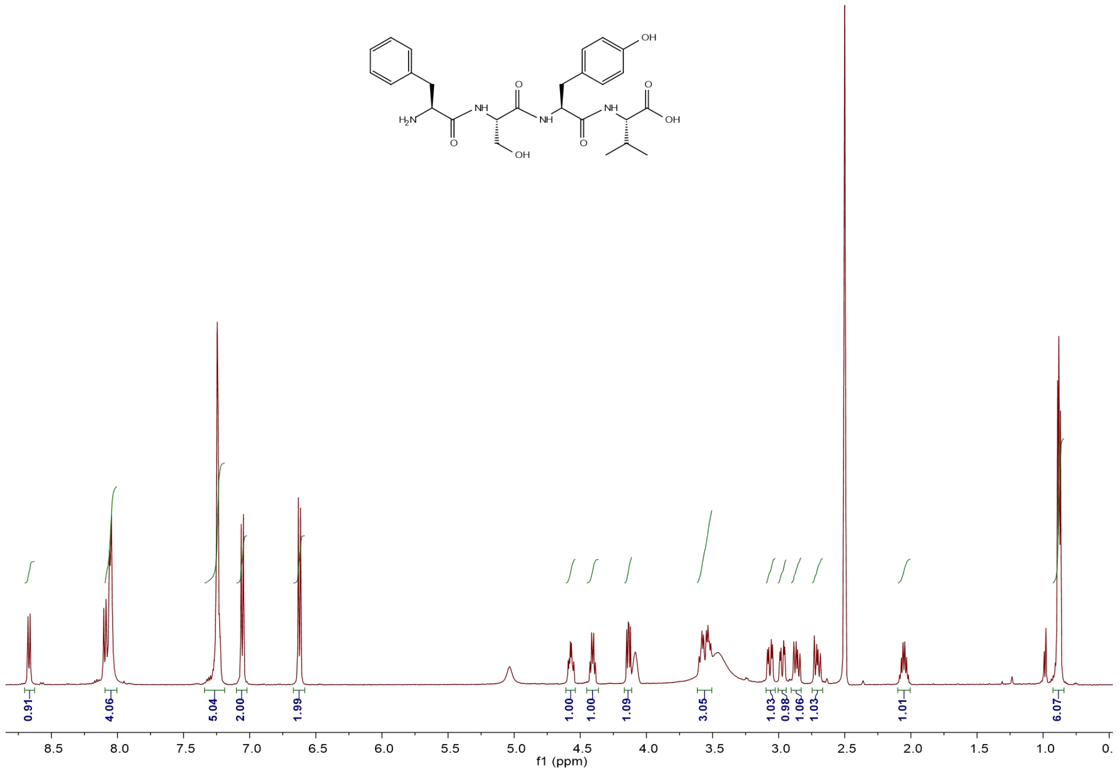

57.Compound **GVLF**: ^{1}H NMR (500 MHz, DMSO) δ 8.35 (d, *J* = 8.9 Hz, 1H), 8.04 (t, *J* = 35.7 Hz, 2H), 7.33 – 7.10 (m, 5H), 4.44 (dd, *J* = 13.4, 8.1 Hz, 1H), 4.33 (dt, *J* = 15.3, 8.5 Hz, 2H), 3.71 – 3.52 (m, 2H), 3.04 (dd, *J* = 14.1, 5.0 Hz, 1H), 2.92 (dd, *J* = 14.0, 8.8 Hz, 1H), 1.93 (td, *J* = 13.4, 6.8 Hz, 1H), 1.55 (td, *J* = 13.0, 6.5 Hz, 1H), 1.41 (d, *J* = 6.7 Hz, 2H), 1.06 – 0.55 (m, 12H).

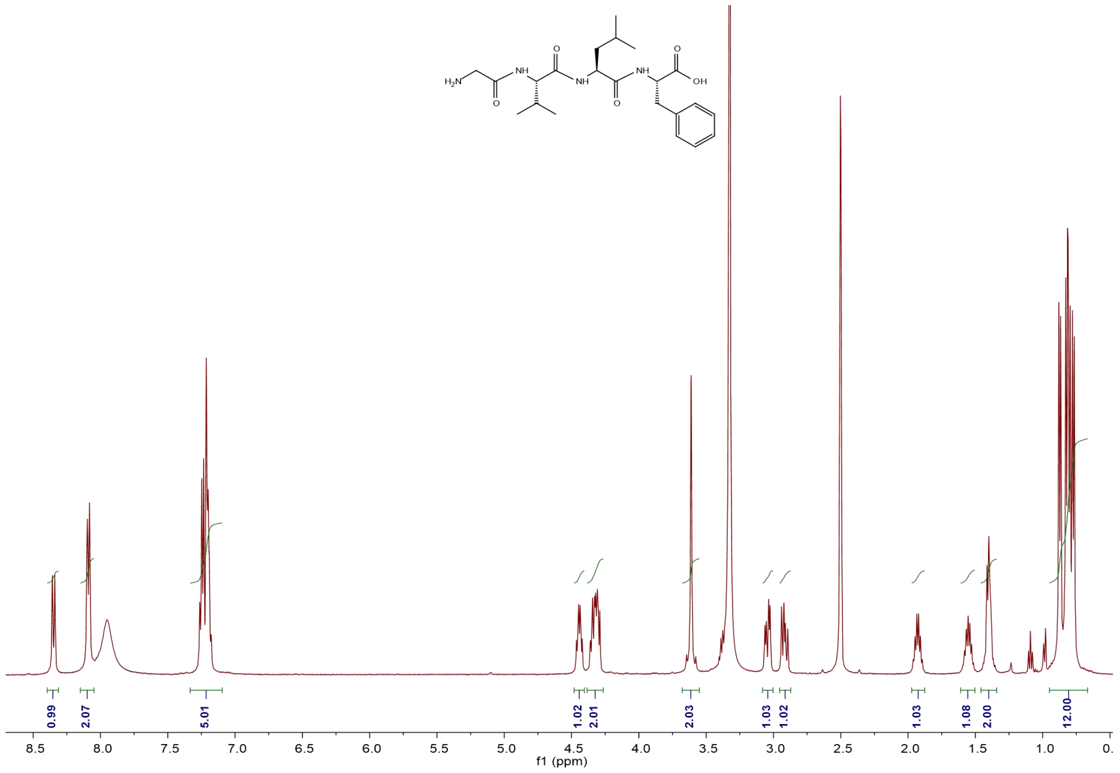

58.Compound **STYF**: ^{1}H NMR (500 MHz, DMSO) δ 9.16 (s, 1H), 8.39 (dd, *J* = 18.8, 8.1 Hz, 2H), 8.08 (s, 2H), 7.80 (d, *J* = 8.2 Hz, 1H), 7.24 (tt, *J* = 14.5, 7.3 Hz, 5H), 7.02 (t, *J* = 13.5 Hz, 2H), 6.61 (d, *J* = 8.1 Hz, 2H), 5.48 (s, 1H), 5.07 (s, 1H), 4.50 – 4.38 (m, 2H), 4.27 (dd, *J* = 8.2, 4.1 Hz, 1H), 4.01 – 3.92 (m, 2H), 3.75 – 3.67 (m, 2H), 3.05 (dd, *J* = 13.9, 5.3 Hz, 1H), 2.91 (dd, *J* = 12.1, 7.4 Hz, 2H), 2.63 (dd, *J* = 13.8, 9.0 Hz, 1H), 1.02 – 0.96 (m, 3H).

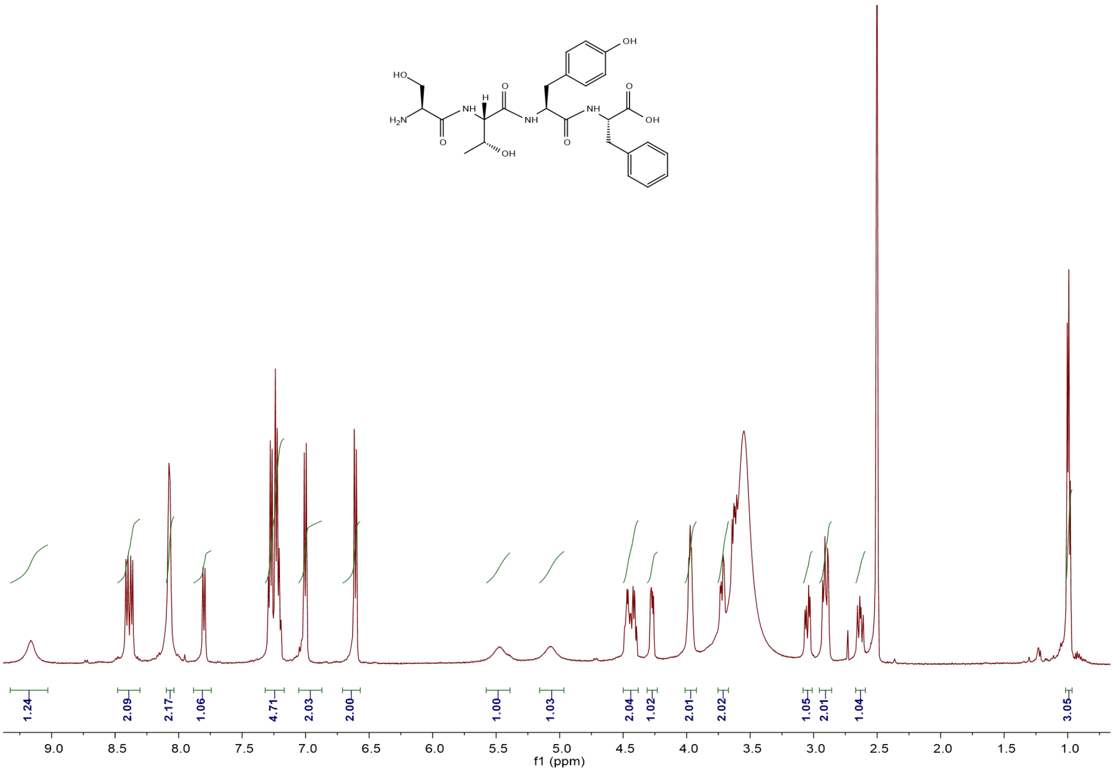

59.Compound **ILVV**: ^{1}H NMR (500 MHz, DMSO-d6) δ 8.43 (d, J = 8.1 Hz, 1H), 8.06 (dd, J = 18.4, 7.2 Hz, 3H), 4.49 (q, J = 7.8 Hz, 1H), 4.28 (t, J = 8.1 Hz, 1H), 4.11 (dd, J = 8.2, 5.8 Hz, 1H), 3.65 (d, J = 5.5 Hz, 1H), 2.01 (ddq, J = 39.3, 13.8, 6.8 Hz, 2H), 1.78 (q, J = 5.7 Hz, 1H), 1.61 (dp, J = 13.3, 6.6 Hz, 1H), 1.52 – 1.40 (m, 3H), 0.94 – 0.79 (m, 24H).

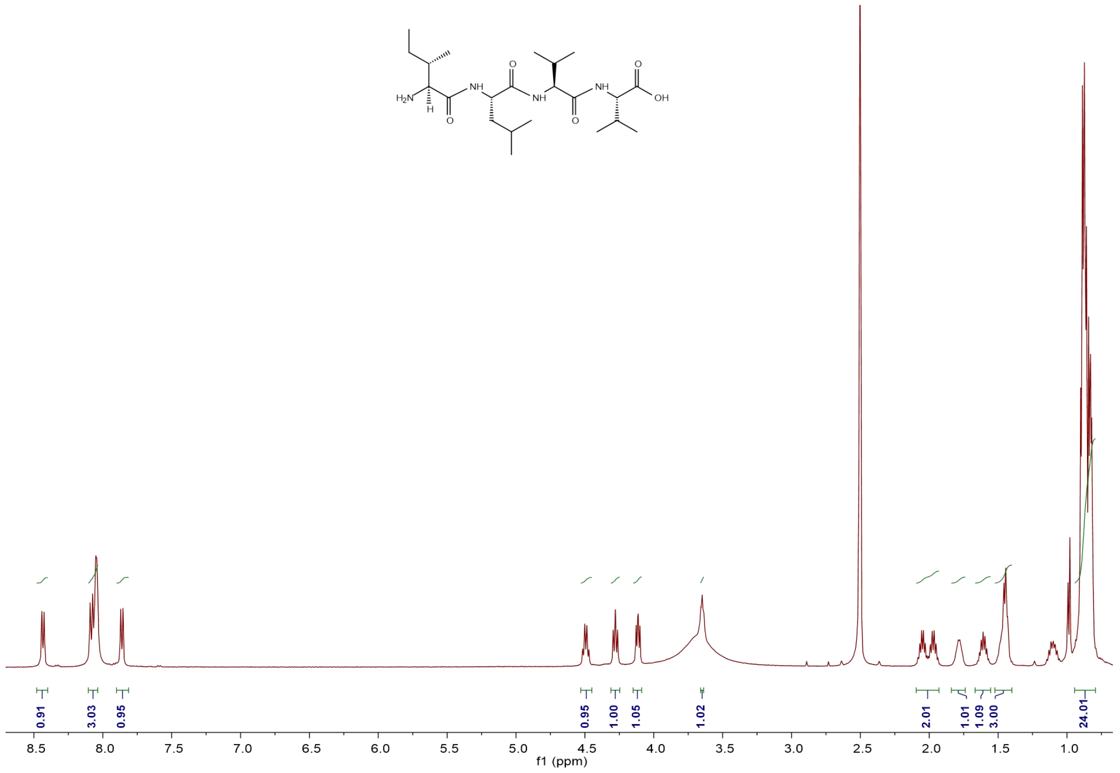

60.Compound **IVII**: ^{1}H NMR (500 MHz, DMSO) δ 12.65 (d, *J* = 114.3 Hz, 1H), 8.35 (d, *J* = 8.7 Hz, 1H), 8.09 – 8.04 (m, 3H), 7.94 (d, *J* = 8.3 Hz, 1H), 4.27 (t, *J* = 8.1 Hz, 2H), 4.18 – 4.11 (m, 1H), 3.78 – 3.70 (m, 1H), 1.90 (dq, *J* = 13.7, 6.7 Hz, 1H), 1.81 – 1.69 (m, 3H), 1.50 – 1.37 (m, 3H), 1.24 – 1.16 (m, 1H), 1.09 (dt, *J* = 13.4, 6.9 Hz, 2H), 0.90 – 0.78 (m, 24H).

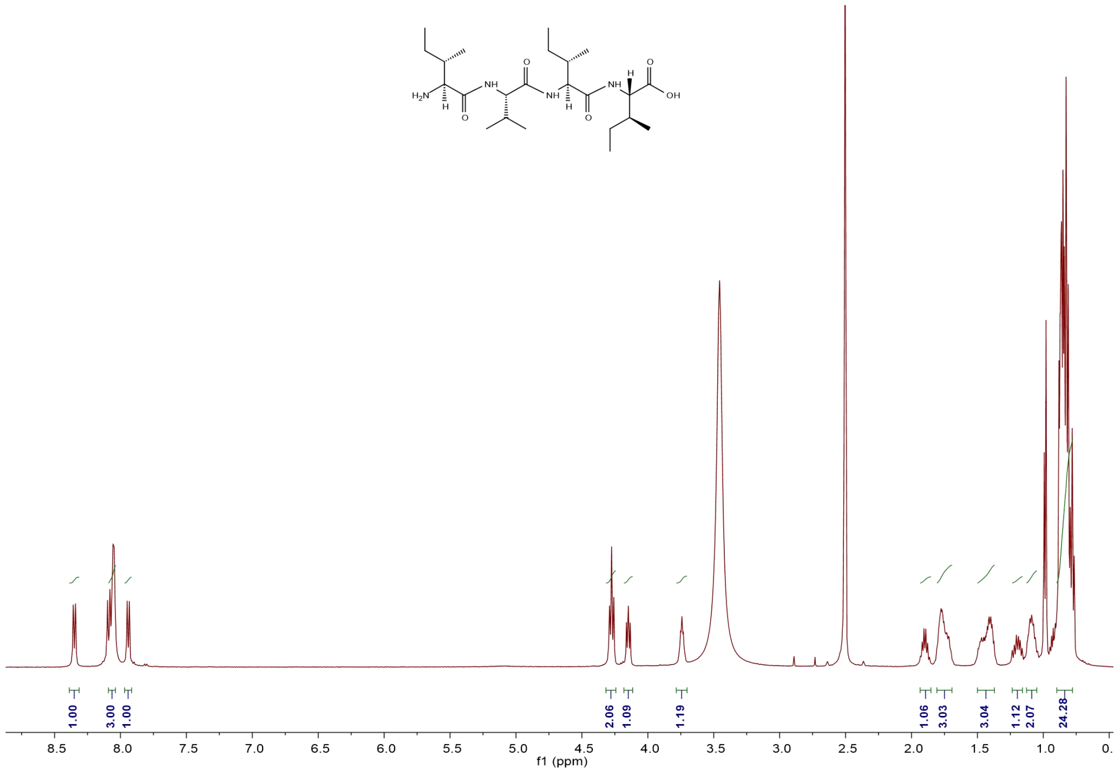

61.Compound **LVII**: ^{1}H NMR (500 MHz, DMSO) δ 12.53 (s, 1H), 8.50 (d, *J* = 8.8 Hz, 1H), 8.10 (s, 2H), 8.04 (d, *J* = 8.8 Hz, 1H), 7.94 (d, *J* = 8.2 Hz, 1H), 4.27 (td, *J* = 8.4, 3.1 Hz, 2H), 4.18 – 4.12 (m, 1H), 3.87 (s, 1H), 1.92 (dq, *J* = 13.7, 6.7 Hz, 1H), 1.82 – 1.68 (m, 2H), 1.60 (dt, *J* = 12.9, 6.5 Hz, 1H), 1.52 – 1.48 (m, 1H), 1.39 (dd, *J* = 11.2, 6.6 Hz, 2H), 1.20 (dt, *J* = 13.9, 7.6 Hz, 1H), 1.12 – 1.04 (m, 1H), 0.99 (d, *J* = 6.8 Hz, 1H), 0.85 (ddd, *J* = 17.7, 11.4, 5.4 Hz, 24H).

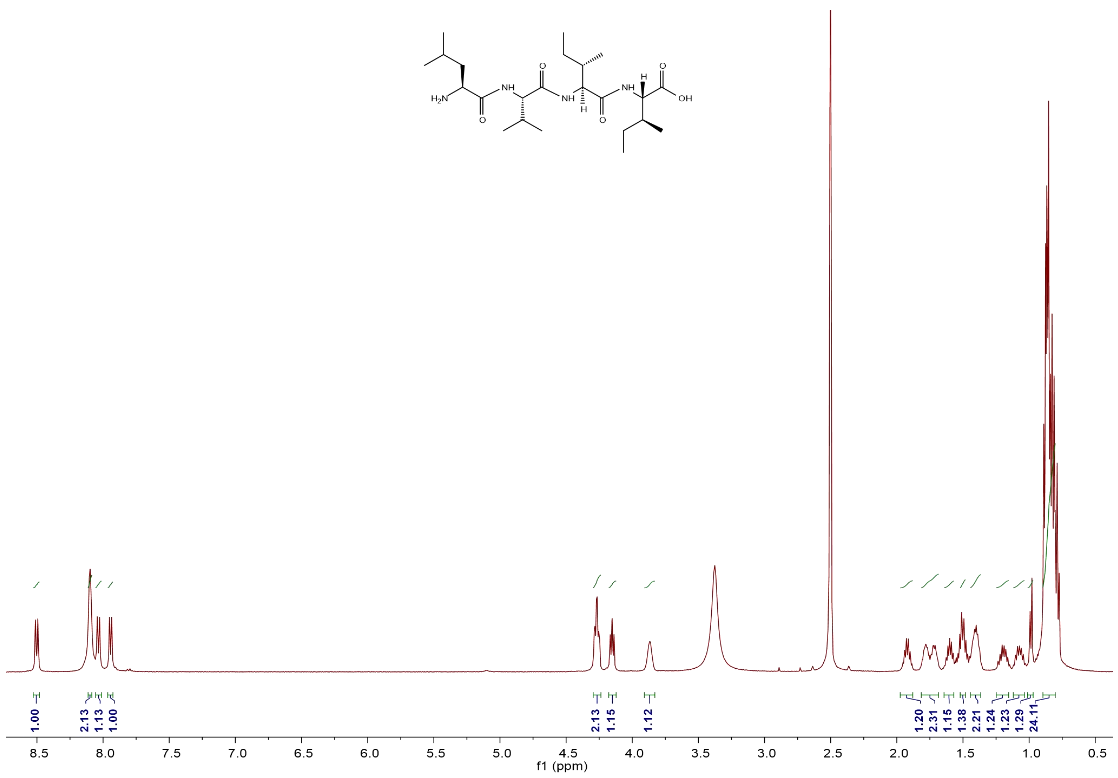

62.Compound **LVLI**: ^{1}H NMR (500 MHz, DMSO-d6) δ 8.54 (d, J = 8.9 Hz, 1H), 8.10 (d, J = 5.2 Hz, 2H), 7.83 (d, J = 8.4 Hz, 1H), 4.42 (dt, J = 9.4, 4.6 Hz, 1H), 4.22 (t, J = 8.3 Hz, 1H), 4.16 (dd, J = 8.3, 6.0 Hz, 1H), 3.87 (p, J = 6.2 Hz, 1H), 1.93 (h, J = 6.9 Hz, 1H), 1.77 (p, J = 6.4 Hz, 1H), 1.63 – 1.35 (m, 7H), 1.23 – 1.13 (m, 1H), 0.95 – 0.77 (m, 24H).

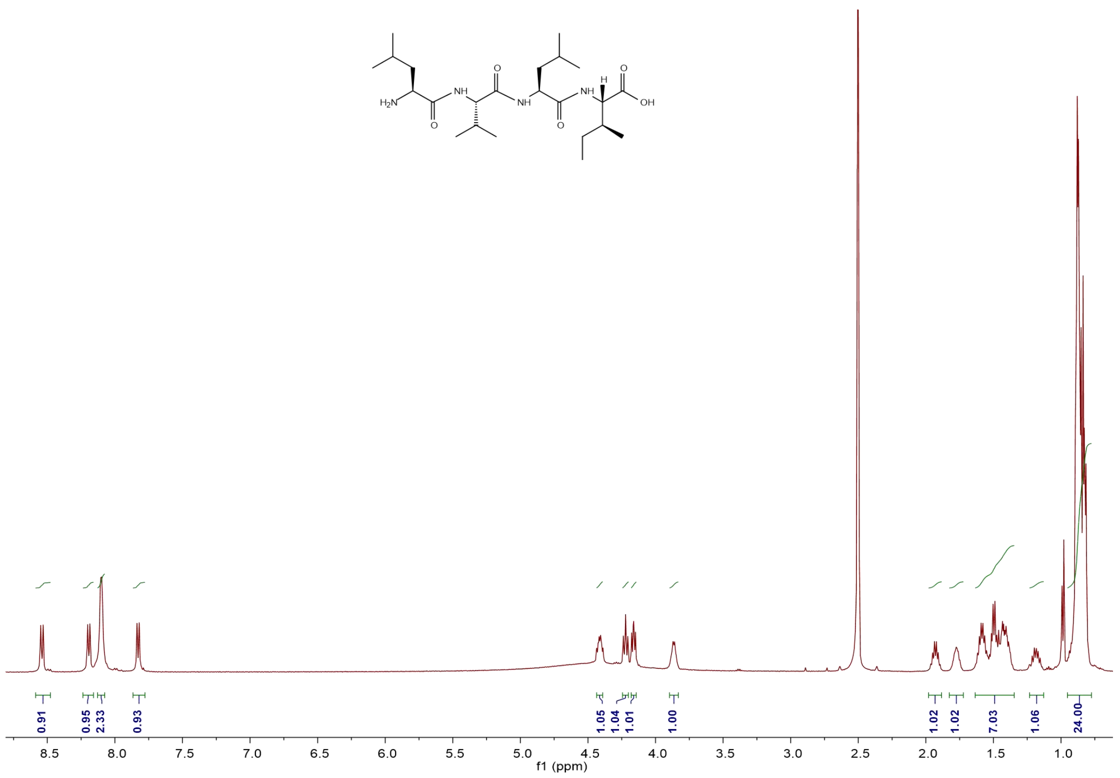

63.Compound **SLVV**: ^{1}H NMR (500 MHz, DMSO) δ 8.53 (d, *J* = 7.9 Hz, 1H), 8.34 (d, *J* = 7.5 Hz, 1H), 8.19 (d, *J* = 7.9 Hz, 1H), 8.10 – 7.92 (m, 1H), 7.59 (s, 1H), 7.33 – 7.19 (m, 5H), 7.19 – 7.14 (m, 1H), 7.01 – 6.82 (m, 1H), 5.06 (s, 1H), 4.69 – 4.58 (m, 1H), 4.39 – 4.22 (m, 2H), 3.74 (dd, *J* = 10.4, 5.4 Hz, 2H), 3.64 (dd, *J* = 10.8, 3.9 Hz, 1H), 3.55 (d, *J* = 7.1 Hz, 1H), 3.13 – 2.99 (m, 3H), 2.76 (dt, *J* = 17.4, 8.8 Hz, 1H), 1.51 – 1.43 (m, 2H), 1.09 – 0.93 (m, 3H).

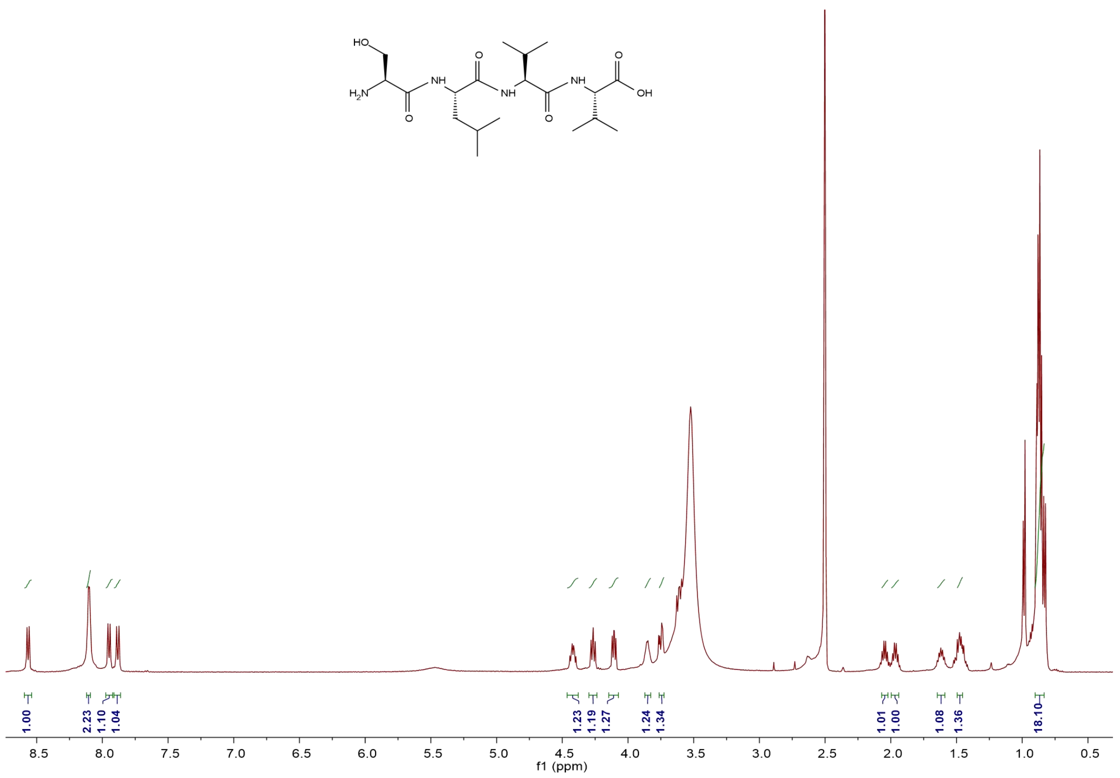

64.Compound **VILI:** ^{1}H NMR (500 MHz, DMSO) δ 12.61 (s, 1H), 8.39 (d, *J* = 8.7 Hz, 1H), 8.24 (d, *J* = 8.4 Hz, 1H), 8.05 (s, 2H), 7.80 (d, *J* = 8.4 Hz, 1H), 4.45 – 4.39 (m, 1H), 4.24 (t, *J* = 8.5 Hz, 1H), 4.16 (dd, *J* = 8.0, 6.3 Hz, 1H), 3.67 (d, *J* = 5.0 Hz, 1H), 2.01 (dd, *J* = 12.7, 6.5 Hz, 1H), 1.77 (s, 1H), 1.68 (d, *J* = 7.1 Hz, 1H), 1.58 (d, *J* = 13.2 Hz, 1H), 1.48 (dd, *J* = 16.2, 7.6 Hz, 2H), 1.40 (dd, *J* = 13.4, 4.6 Hz, 2H), 1.17 (dd, *J* = 13.0, 6.7 Hz, 1H), 1.07 (dd, *J* = 13.4, 7.4 Hz, 1H), 0.85 (ddd, *J* = 18.0, 13.4, 5.6 Hz, 24H).

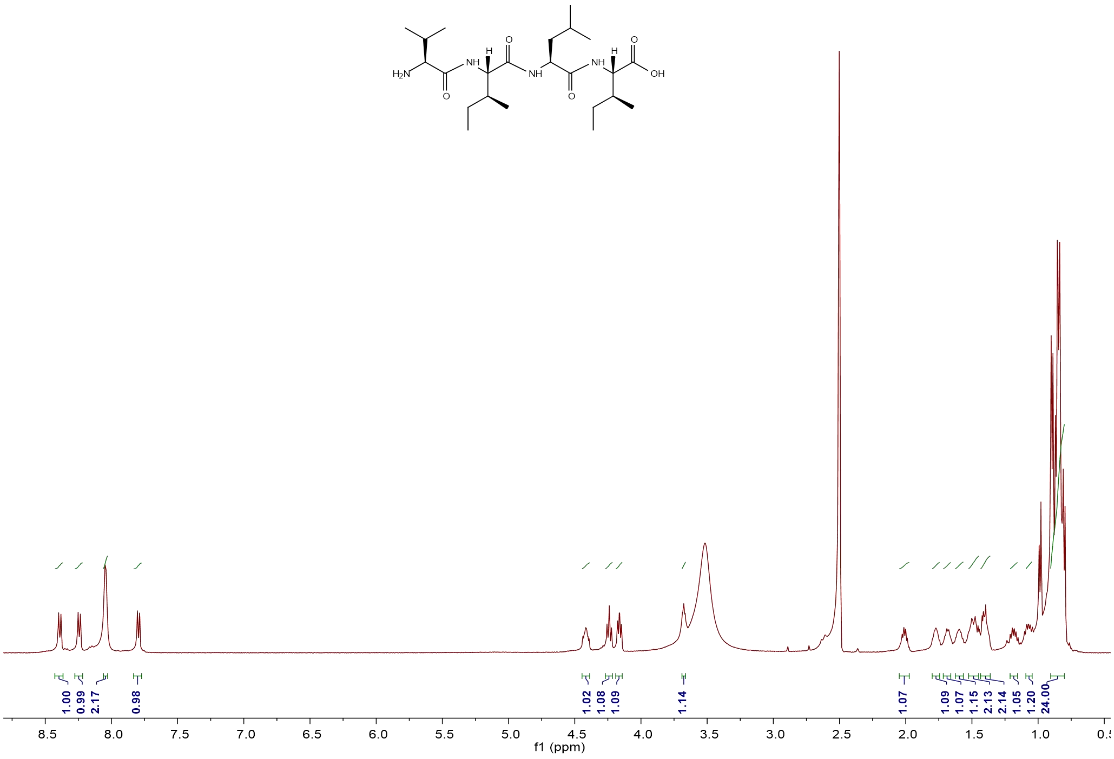

65.Compound **VLVL**: ^{1}H NMR (500 MHz, DMSO) δ 12.49 (s, 1H), 8.42 (d, *J* = 7.9 Hz, 1H), 8.05 (dd, *J* = 13.6, 6.6 Hz, 4H), 4.46 (q, *J* = 7.6 Hz, 1H), 4.25 – 4.13 (m, 2H), 2.05 (dq, *J* = 13.4, 6.8 Hz, 1H), 1.94 (dq, *J* = 13.9, 6.8 Hz, 1H), 1.60 (tt, *J* = 11.9, 5.9 Hz, 2H), 1.54 – 1.40 (m, 4H), 0.99 (d, *J* = 6.7 Hz, 1H), 0.87 (dtd, *J* = 18.4, 10.3, 6.1 Hz, 24H).

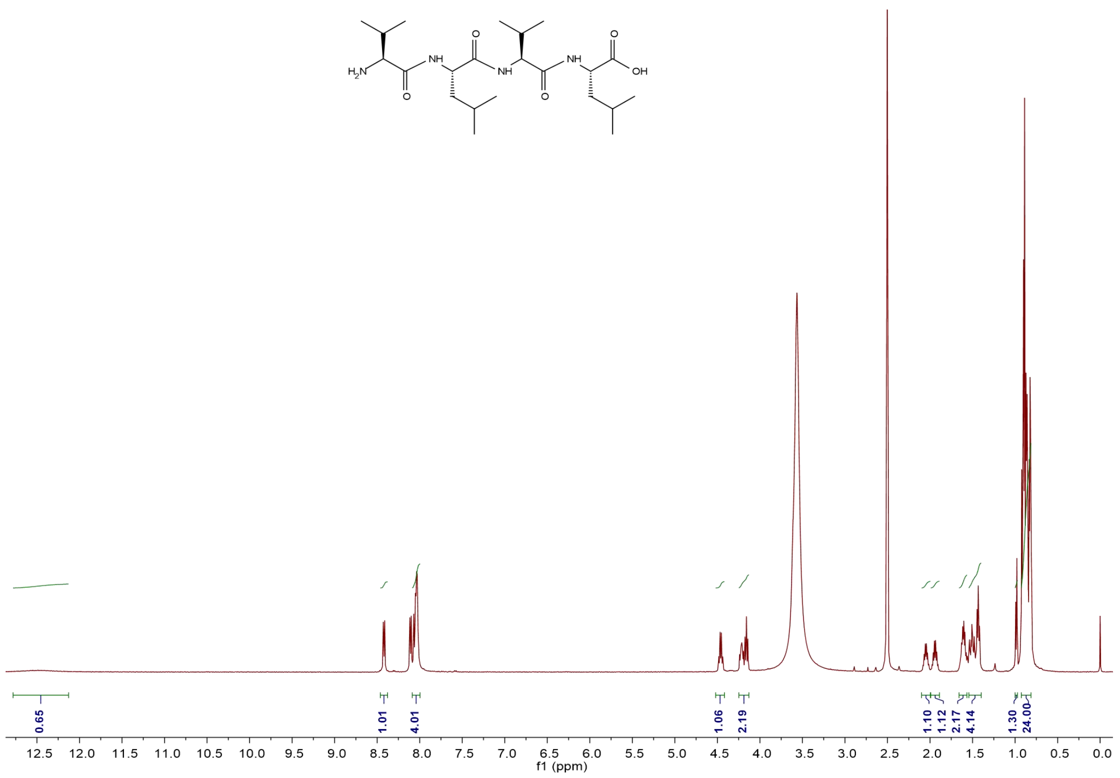

66.Compound **GVFY**: ^{1}H NMR (500 MHz, DMSO-d6) δ 8.27 (d, J = 9.1 Hz, 1H), 8.17 (dd, J = 21.1, 8.0 Hz, 2H), 7.29 – 7.13 (m, 5H), 7.00 (d, J = 8.4 Hz, 2H), 6.64 (d, J = 8.4 Hz, 2H), 4.56 (td, J = 9.5, 4.4 Hz, 1H), 4.36 (q, J = 7.8 Hz, 1H), 4.27 (dd, J = 9.0, 6.2 Hz, 1H), 3.62 – 3.49 (m, 2H), 3.01 – 2.87 (m, 2H), 2.82 (dd, J = 14.0, 8.0 Hz, 1H), 2.73 (dd, J = 13.8, 10.1 Hz, 1H), 1.92 (dq, J = 13.1, 6.6 Hz, 1H), 0.76 (dd, J = 21.5, 6.8 Hz, 6H).

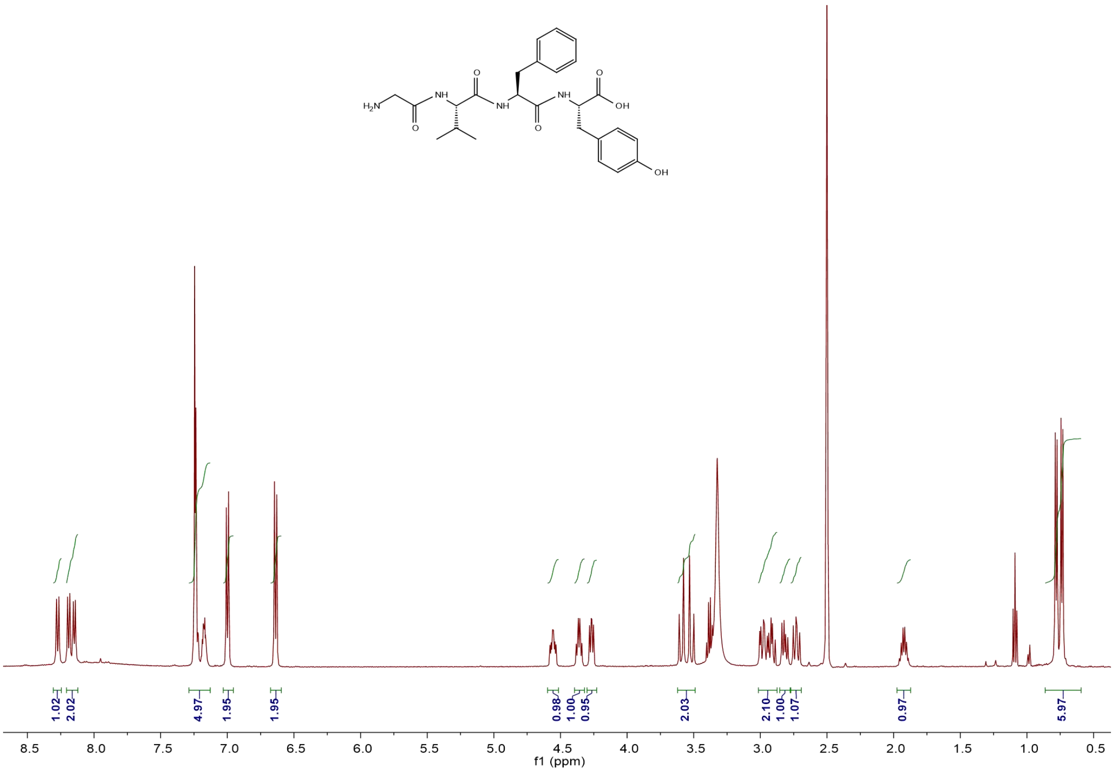

67.Compound **IMVV**: ^{1}H NMR (500 MHz, DMSO) δ 8.49 (d, *J* = 7.9 Hz, 1H), 7.99 (dd, *J* = 47.9, 8.5 Hz, 2H), 4.52 (dd, *J* = 13.0, 7.4 Hz, 1H), 4.27 (t, *J* = 7.8 Hz, 1H), 4.19 – 4.06 (m, 1H), 3.60 (d, *J* = 5.3 Hz, 1H), 2.48 – 2.34 (m, 2H), 2.04 (d, *J* = 7.3 Hz, 3H), 1.97 (dd, *J* = 13.5, 6.7 Hz, 1H), 1.89 (dd, *J* = 14.4, 9.3 Hz, 1H), 1.77 (s, 2H), 1.47 (s, 1H), 1.17 – 0.94 (m, 2H), 0.91 – 0.79 (m, 18H).

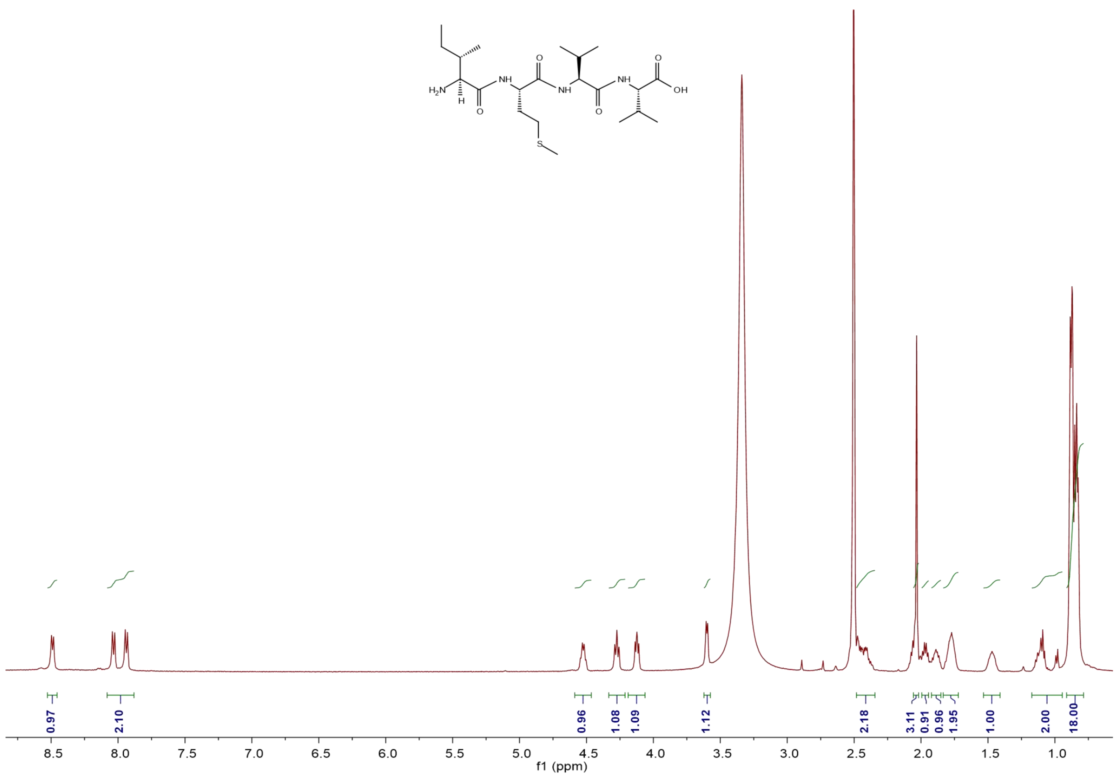

68.Compound **LTLF**: ^{1}H NMR (500 MHz, DMSO) δ 8.57 (d, *J* = 8.4 Hz, 1H), 8.17 (d, *J* = 7.7 Hz, 1H), 8.08 (d, *J* = 2.6 Hz, 2H), 7.96 (d, *J* = 8.4 Hz, 1H), 7.30 – 7.16 (m, 5H), 4.96 (s, 1H), 4.46 – 4.27 (m, 3H), 3.99 – 3.85 (m, 2H), 3.04 (dd, *J* = 14.0, 5.2 Hz, 1H), 2.89 (dd, *J* = 13.6, 9.4 Hz, 1H), 1.56 (tdt, *J* = 35.3, 13.8, 6.8 Hz, 4H), 1.44 – 1.32 (m, 2H), 1.04 (d, *J* = 6.2 Hz, 3H), 0.86 (ddd, *J* = 26.5, 15.6, 6.4 Hz, 12H).

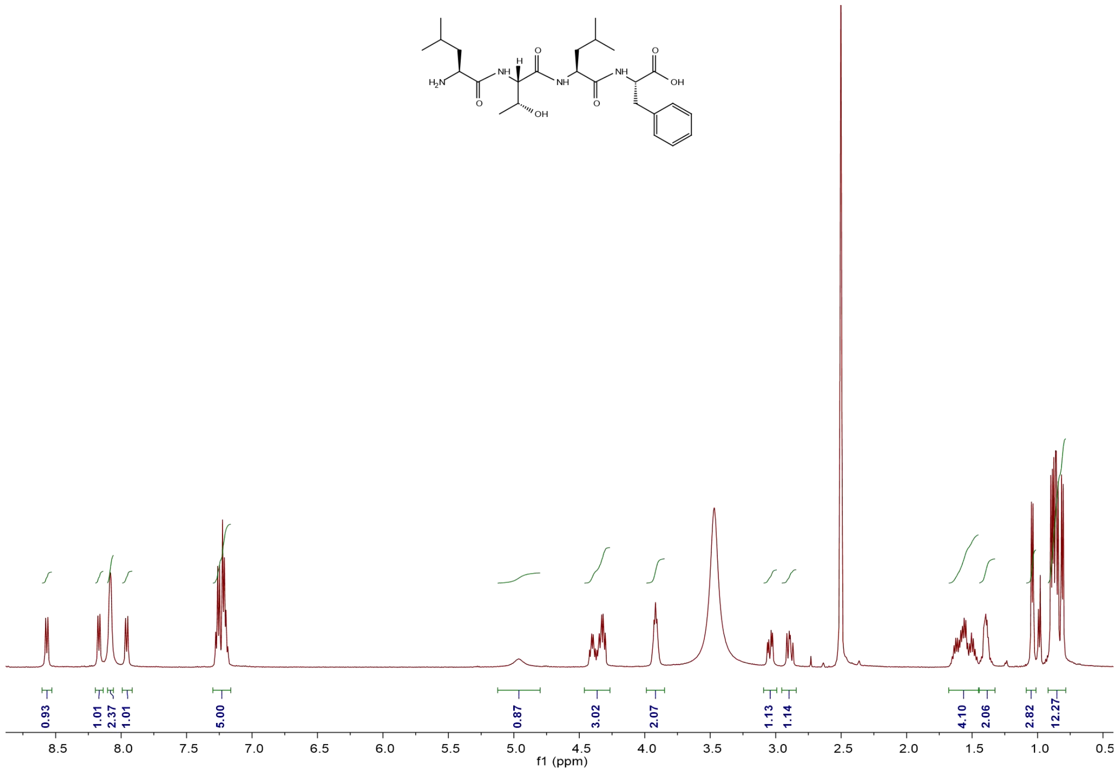

69.Compound **LTLI**: ^{1}H NMR (500 MHz, DMSO) δ 8.61 (d, *J* = 8.3 Hz, 1H), 8.10 (s, 3H), 8.01 (d, *J* = 8.3 Hz, 1H), 7.94 (d, *J* = 8.2 Hz, 1H), 4.92 (s, 1H), 4.41 (dd, *J* = 15.4, 7.7 Hz, 1H), 4.36 – 4.29 (m, 1H), 4.18 – 4.10 (m, 1H), 3.97 – 3.86 (m, 2H), 1.76 (d, *J* = 12.0 Hz, 1H), 1.66 – 1.39 (m, 7H), 1.22 – 1.15 (m, 1H), 1.08 (d, *J* = 6.1 Hz, 3H), 0.93 – 0.80 (m, 18H).

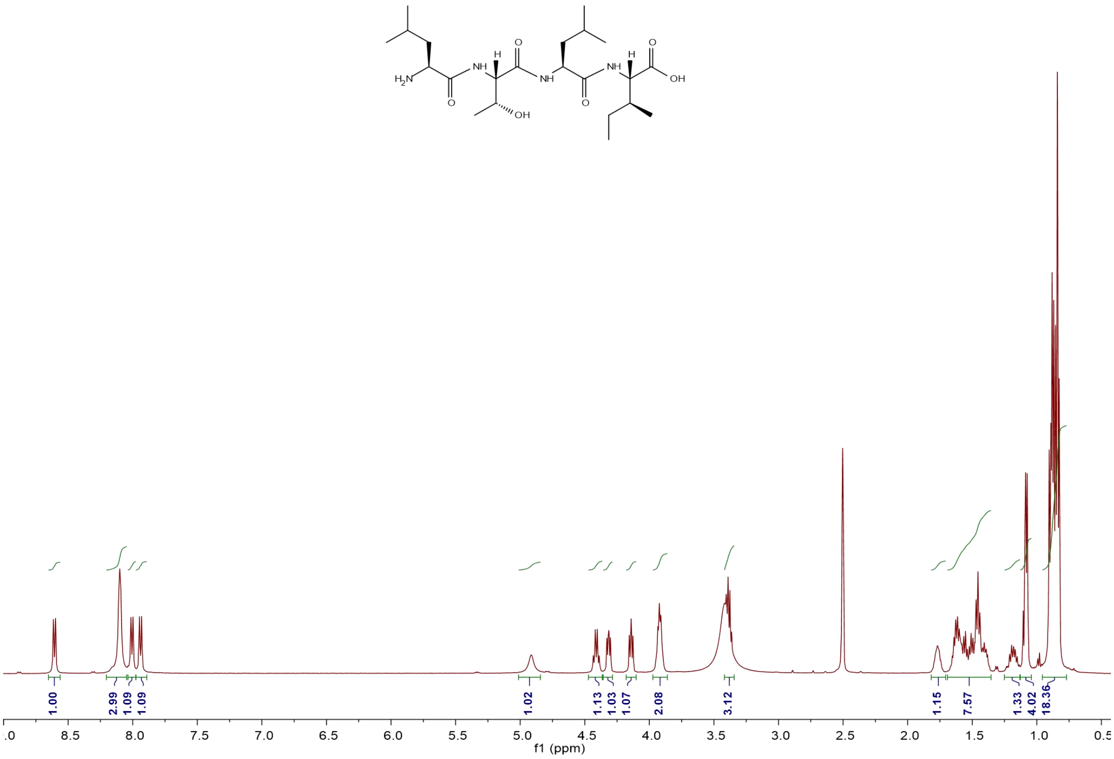

70.Compound **LWFF**: ^{1}H NMR (500 MHz, DMSO-d6) δ 8.50 (d, J = 8.2 Hz, 1H), 8.25 (d, J = 7.6 Hz, 1H), 8.20 (d, J = 8.2 Hz, 1H), 7.60 (d, J = 7.9 Hz, 1H), 7.31 (d, J = 8.1 Hz, 1H), 7.29 – 7.13 (m, 10H), 7.10 – 6.99 (m, 2H), 6.95 (t, J = 7.4 Hz, 1H), 4.57 (dtd, J = 12.8, 8.5, 4.8 Hz, 2H), 4.44 (td, J = 7.9, 5.4 Hz, 1H), 3.62 (dd, J = 8.5, 5.7 Hz, 1H), 3.39 (s, 1H), 3.10 – 2.99 (m, 3H), 2.96 – 2.87 (m, 2H), 2.78 (dd, J = 14.0, 9.3 Hz, 1H), 1.57 (dp, J = 13.3, 6.5 Hz, 1H), 1.42 (dt, J = 12.6, 7.5 Hz, 2H), 0.83 (t, J = 6.9 Hz, 6H).

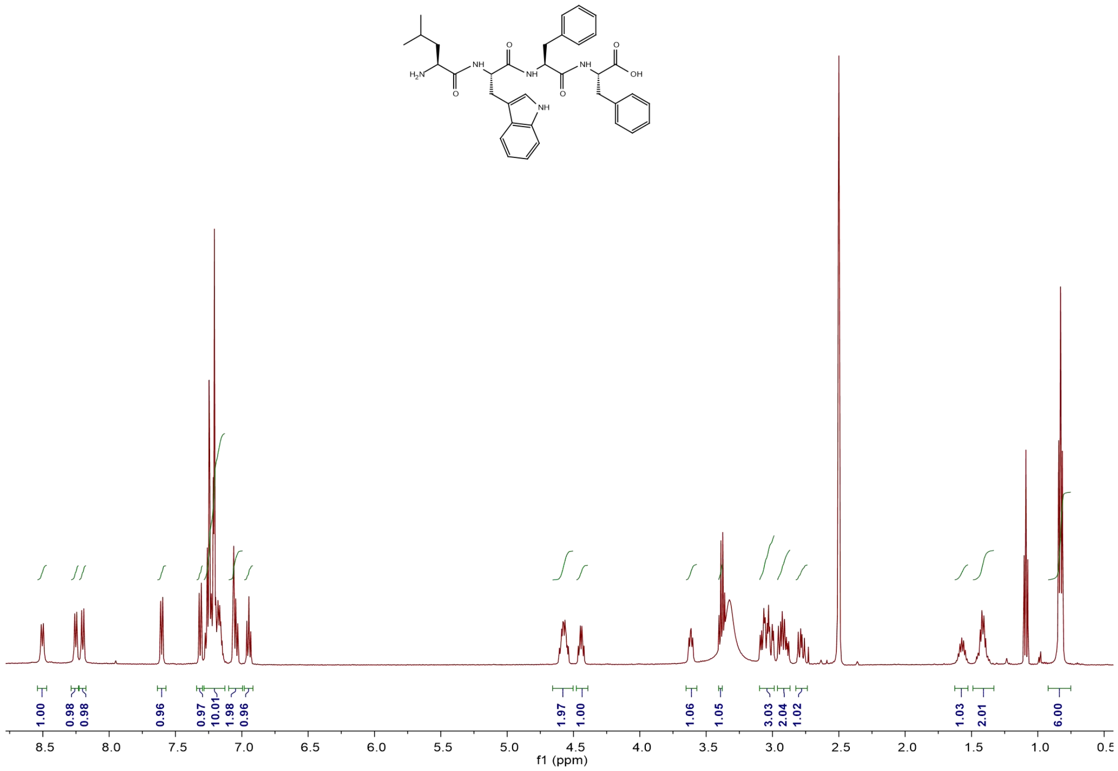

71.Compound **IQII**: ^{1}H NMR (600 MHz, DMSO-d6) δ 8.52 (d, J = 7.9 Hz, 1H), 8.04 (d, J = 8.9 Hz, 1H), 8.00 (d, J = 8.1 Hz, 1H), 7.22 (d, J = 2.4 Hz, 1H), 6.81 (d, J = 2.4 Hz, 1H), 4.40 (q, J = 7.6 Hz, 1H), 4.29 (t, J = 8.3 Hz, 1H), 4.15 (dd, J = 8.1, 6.1 Hz, 1H), 3.65 (d, J = 5.6 Hz, 1H), 2.10 (dddd, J = 37.6, 15.2, 10.1, 5.9 Hz, 2H), 1.83 – 1.69 (m, 5H), 1.49 (dtd, J = 13.8, 7.3, 3.6 Hz, 1H), 1.41 (dqd, J = 14.7, 7.0, 3.8 Hz, 2H), 1.19 (ddd, J = 13.5, 8.7, 7.0 Hz, 1H), 1.13 – 1.05 (m, 2H), 0.89 – 0.77 (m, 18H).

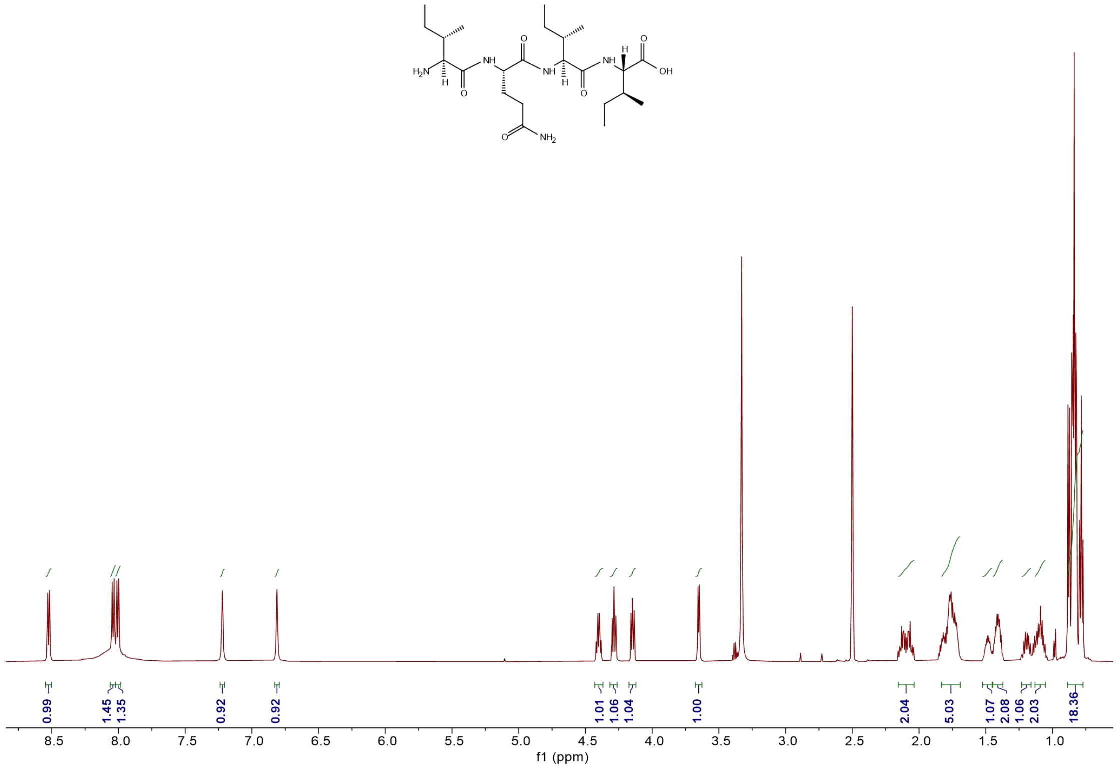

72.Compound **LVIF**: ^{1}H NMR (500 MHz, DMSO) δ 8.47 (d, *J* = 8.8 Hz, 1H), 8.20 (d, *J* = 7.8 Hz, 1H), 8.09 (s, 2H), 7.94 (d, *J* = 9.0 Hz, 1H), 7.32 – 7.06 (m, 5H), 4.46 (dd, *J* = 13.4, 8.5 Hz, 1H), 4.30 – 4.15 (m, 2H), 3.86 (s, 1H), 3.05 (dd, *J* = 14.2, 4.8 Hz, 1H), 2.89 (dd, *J* = 14.0, 9.4 Hz, 1H), 1.87 (dq, *J* = 13.5, 6.7 Hz, 1H), 1.67 (d, *J* = 7.7 Hz, 1H), 1.59 (dt, *J* = 13.0, 6.4 Hz, 1H), 1.49 (qd, *J* = 13.6, 7.0 Hz, 2H), 1.35 (d, *J* = 7.8 Hz, 1H), 1.04 – 0.98 (m, 1H), 0.96 – 0.54 (m, 18H).

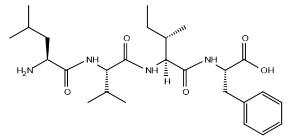

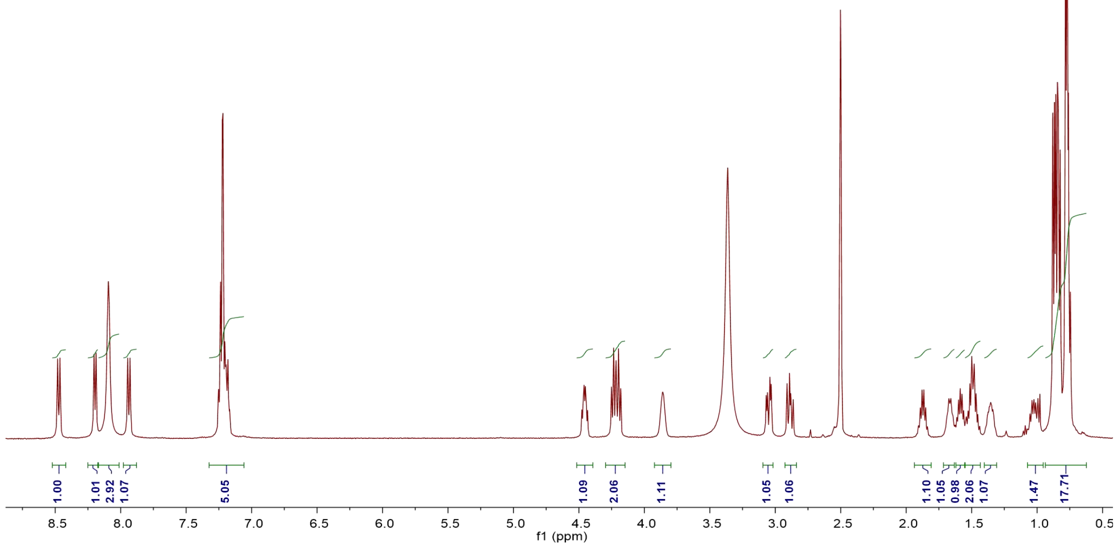

73.Compound **LVWF**: ^{1}H NMR (500 MHz, DMSO-d6) δ 8.32 (d, J = 8.6 Hz, 1H), 8.13 (dd, J = 20.1, 7.9 Hz, 2H), 7.56 (d, J = 7.9 Hz, 1H), 7.30 (d, J = 8.1 Hz, 1H), 7.25 – 7.17 (m, 5H), 7.05 (dd, J = 15.7, 8.4 Hz, 2H), 6.95 (t, J = 7.5 Hz, 1H), 4.59 (q, J = 8.1 Hz, 1H), 4.42 (q, J = 7.7 Hz, 1H), 4.23 – 4.18 (m, 1H), 3.74 (t, J = 6.4 Hz, 1H), 3.05 (td, J = 14.3, 12.6, 5.1 Hz, 2H), 2.91 (dd, J = 14.3, 7.8 Hz, 2H), 1.93 (dq, J = 13.4, 7.0, 6.6 Hz, 1H), 1.42 (dd, J = 10.2, 4.0 Hz, 2H), 0.87 – 0.77 (m, 12H).

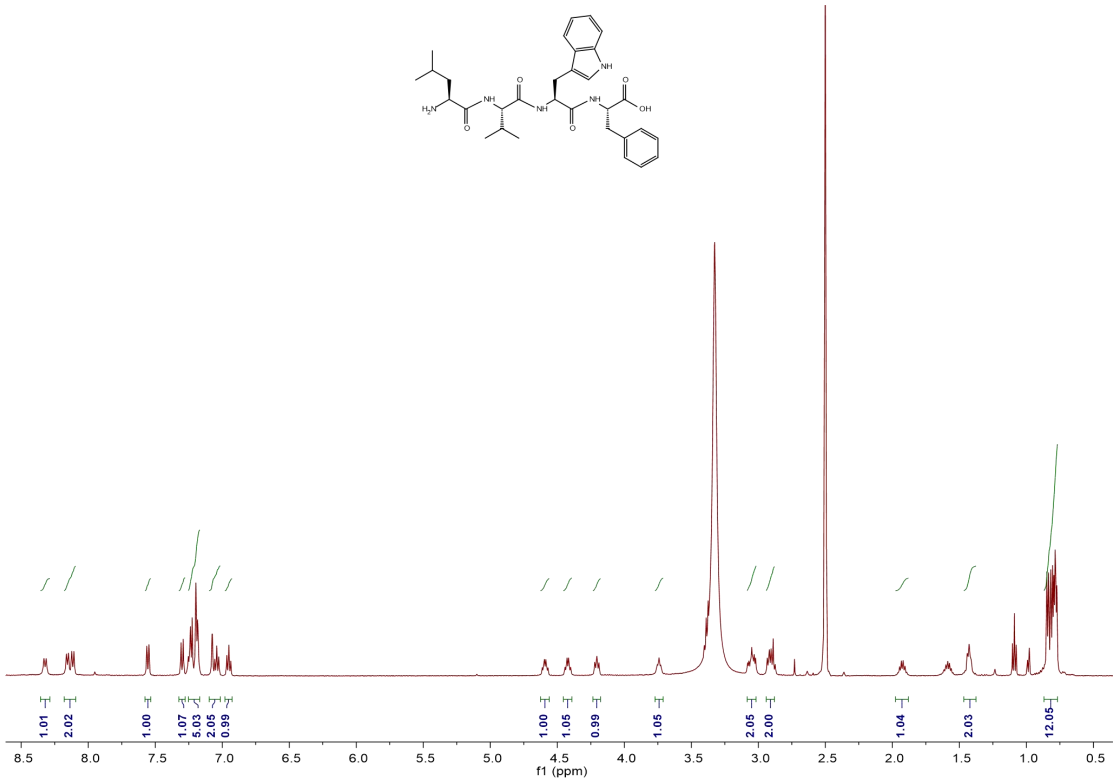

74.Compound **WVII**: ^{1}H NMR (500 MHz, DMSO-d6) δ 10.98 (s, 1H), 8.61 (d, J = 8.7 Hz, 1H), 8.05 (d, J = 8.7 Hz, 1H), 7.96 (d, J = 8.2 Hz, 1H), 7.68 (d, J = 7.9 Hz, 1H), 7.36 (d, J = 8.1 Hz, 1H), 7.22 – 7.18 (m, 1H), 7.09 (t, J = 7.4 Hz, 1H), 7.00 (t, J = 7.4 Hz, 1H), 4.36 – 4.27 (m, 2H), 4.16 (dd, J = 8.1, 6.4 Hz, 1H), 4.06 (dd, J = 7.8, 4.5 Hz, 1H), 3.38 (q, J = 7.0 Hz, 3H), 3.19 (dd, J = 14.8, 4.4 Hz, 1H), 2.98 (dd, J = 14.8, 8.7 Hz, 1H), 1.77 (dp, J = 21.3, 6.8 Hz, 2H), 1.51 – 1.36 (m, 2H), 1.21 – 1.06 (m, 4H), 0.88 – 0.80 (m, 18H).

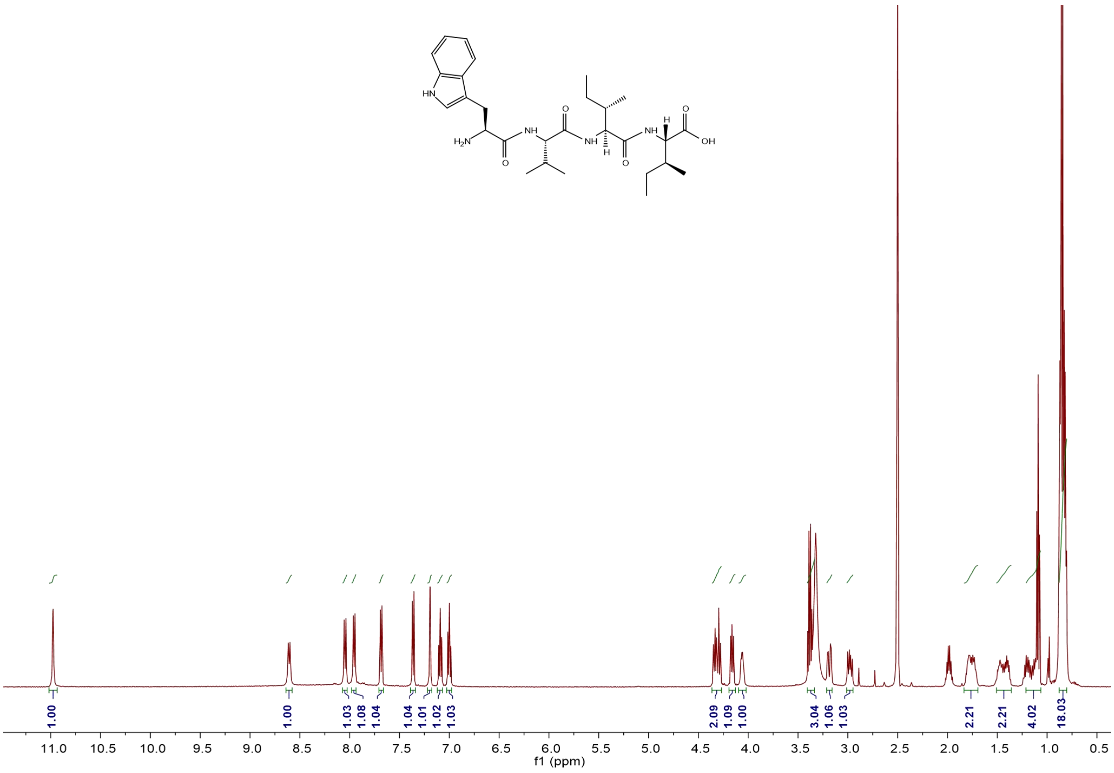

75.Compound **LVIY**: ^{1}H NMR (500 MHz, DMSO) δ 9.18 (s, 1H), 8.48 (d, *J* = 8.8 Hz, 1H), 8.10 (d, *J* = 7.7 Hz, 4H), 6.99 (d, *J* = 8.1 Hz, 2H), 6.62 (d, *J* = 8.1 Hz, 2H), 4.36 (dd, *J* = 13.3, 8.1 Hz, 1H), 4.22 (dt, *J* = 17.3, 8.3 Hz, 2H), 3.86 (s, 1H), 2.91 (dd, *J* = 14.3, 5.0 Hz, 1H), 2.83 – 2.71 (m, 1H), 1.89 (dq, *J* = 13.8, 6.9 Hz, 1H), 1.56 (dddd, *J* = 31.2, 21.3, 19.2, 7.6 Hz, 4H), 1.42 – 1.28 (m, 1H), 1.12 – 0.95 (m, 1H), 0.90 – 0.75 (m, 18H).

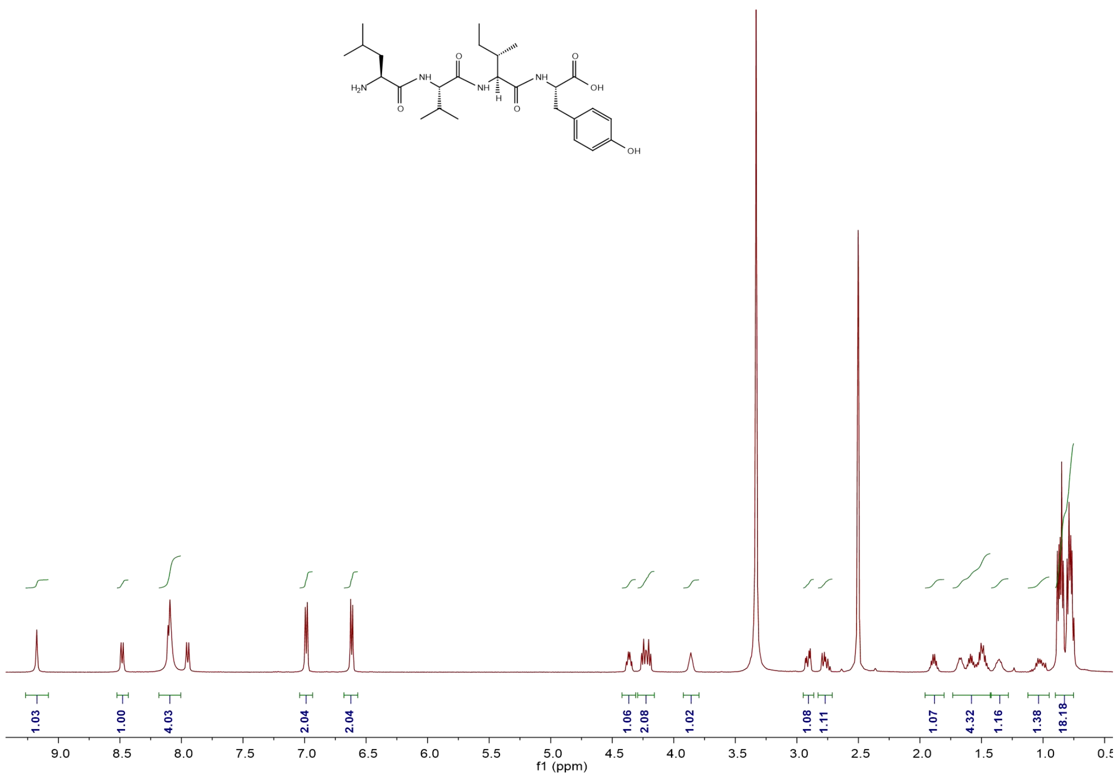

76.Compound **QMVV**: ^{1}H NMR (600 MHz, DMSO-d6) δ 8.65 (d, J = 7.8 Hz, 1H), 8.15 (d, J = 5.3 Hz, 3H), 8.01 (dd, J = 8.6, 6.3 Hz, 2H), 7.39 (d, J = 2.4 Hz, 1H), 6.99 – 6.95 (m, 1H), 4.48 (td, J = 8.1, 5.0 Hz, 1H), 4.26 (dd, J = 8.7, 7.2 Hz, 1H), 4.13 (dd, J = 8.3, 5.8 Hz, 1H), 3.83 (p, J = 5.9 Hz, 1H), 2.48 – 2.39 (m, 2H), 2.20 (td, J = 7.5, 4.2 Hz, 2H), 2.03 (s, 4H), 1.97 (dt, J = 13.7, 6.8 Hz, 1H), 1.89 (q, J = 7.5 Hz, 3H), 1.79 (dtd, J = 13.6, 9.0, 8.6, 5.1 Hz, 1H), 0.89 – 0.83 (m, 12H).

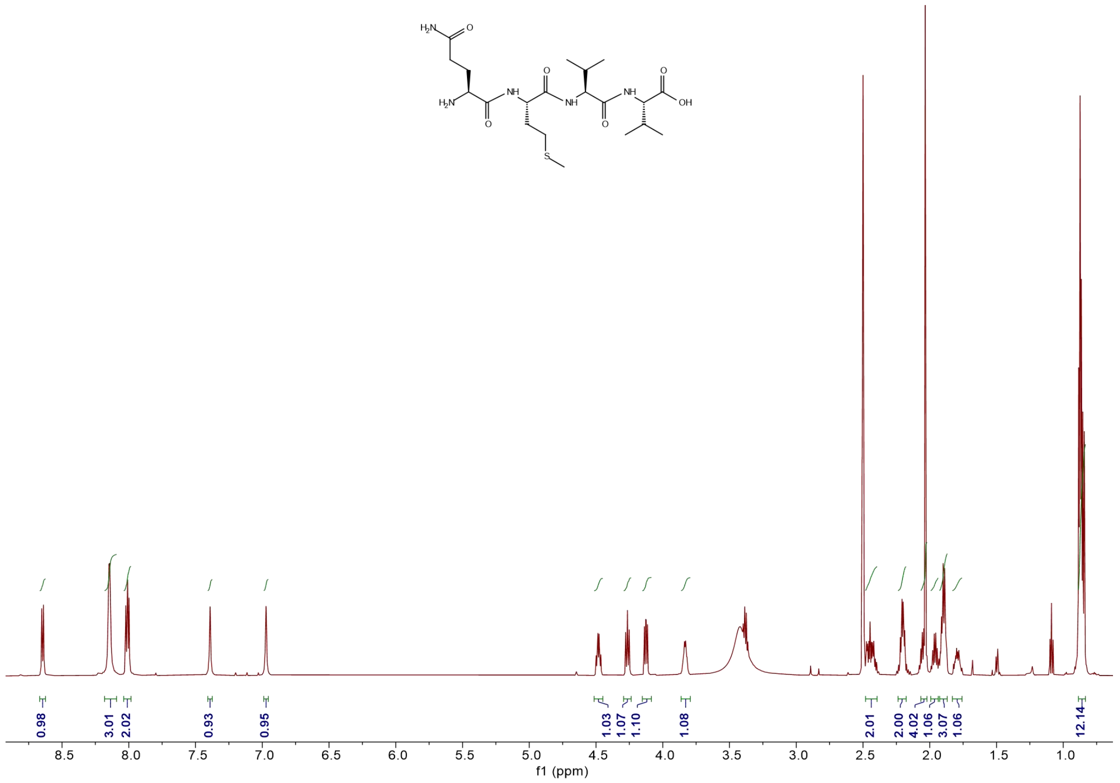

77.Compound **WTIF**: ^{1}H NMR (600 MHz, DMSO-d6) δ 8.80 (d, J = 8.2 Hz, 1H), 8.33 – 8.23 (m, 1H), 7.98 (d, J = 5.4 Hz, 2H), 7.75 (d, J = 7.9 Hz, 1H), 7.37 (d, J = 8.0 Hz, 1H), 7.27 – 7.17 (m, 6H), 7.09 (t, J = 7.5 Hz, 1H), 7.00 (t, J = 7.4 Hz, 1H), 4.48 – 4.35 (m, 2H), 4.29 (dd, J = 8.9, 6.4 Hz, 1H), 4.18 (dq, J = 9.7, 5.3 Hz, 1H), 3.98 (p, J = 6.2 Hz, 1H), 3.38 (q, J = 7.0 Hz, 1H), 3.25 (dd, J = 14.8, 4.7 Hz, 1H), 3.04 (dtd, J = 14.9, 9.6, 8.9, 4.4 Hz, 2H), 2.91 – 2.86 (m, 1H), 1.75 (dtt, J = 10.0, 6.7, 4.1 Hz, 1H), 1.34 (dtq, J = 15.6, 8.2, 5.2, 4.2 Hz, 1H), 1.10 – 0.98 (m, 6H), 0.86 – 0.79 (m, 3H).

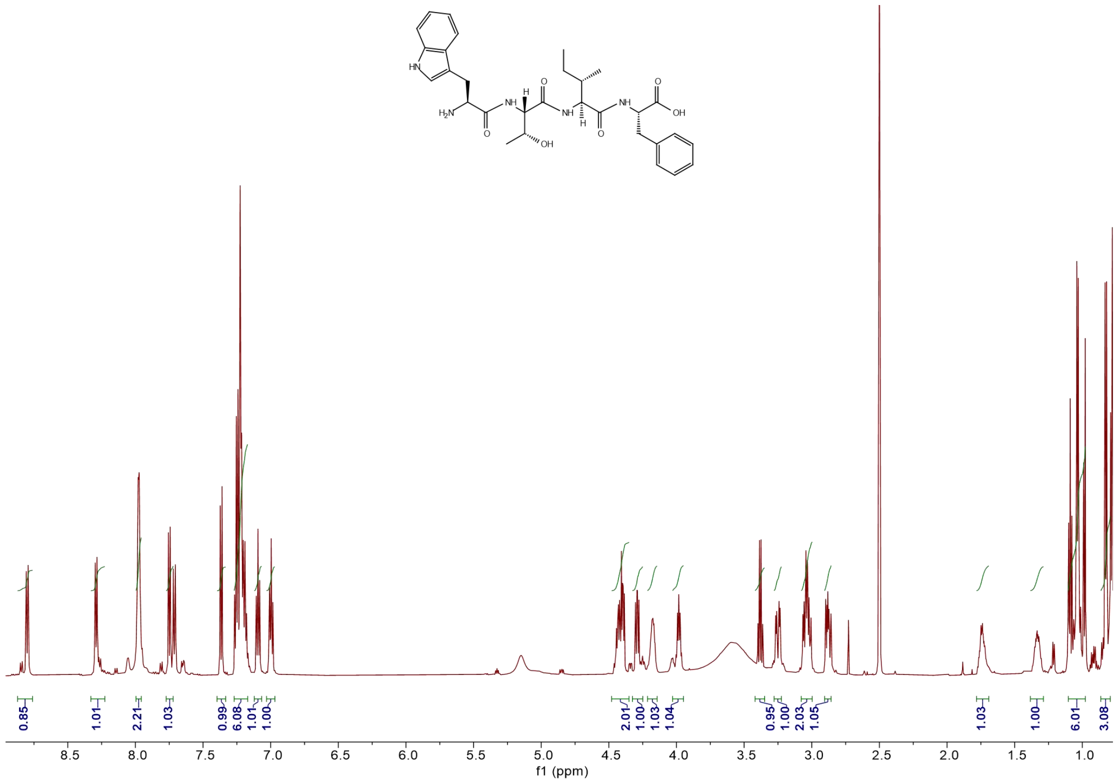

78.Compound **IVIY**: ^{1}H NMR (500 MHz, DMSO) δ 12.56 (s, 1H), 9.17 (s, 1H), 8.32 (d, *J* = 8.7 Hz, 1H), 8.09 – 7.96 (m, 4H), 6.99 (d, *J* = 8.3 Hz, 2H), 6.61 (d, *J* = 8.3 Hz, 2H), 4.35 (dt, *J* = 13.5, 6.8 Hz, 1H), 4.21 (dq, *J* = 13.3, 8.5 Hz, 2H), 3.74 (s, 1H), 2.93 – 2.88 (m, 1H), 2.81 – 2.72 (m, 1H), 1.93 – 1.82 (m, 1H), 1.76 (d, *J* = 6.1 Hz, 1H), 1.68 (d, *J* = 5.9 Hz, 1H), 1.51 – 1.41 (m, 1H), 1.37 (dd, *J* = 11.6, 6.1 Hz, 1H), 1.07 (dd, *J* = 17.7, 5.8 Hz, 2H), 0.88 – 0.75 (m, 18H).

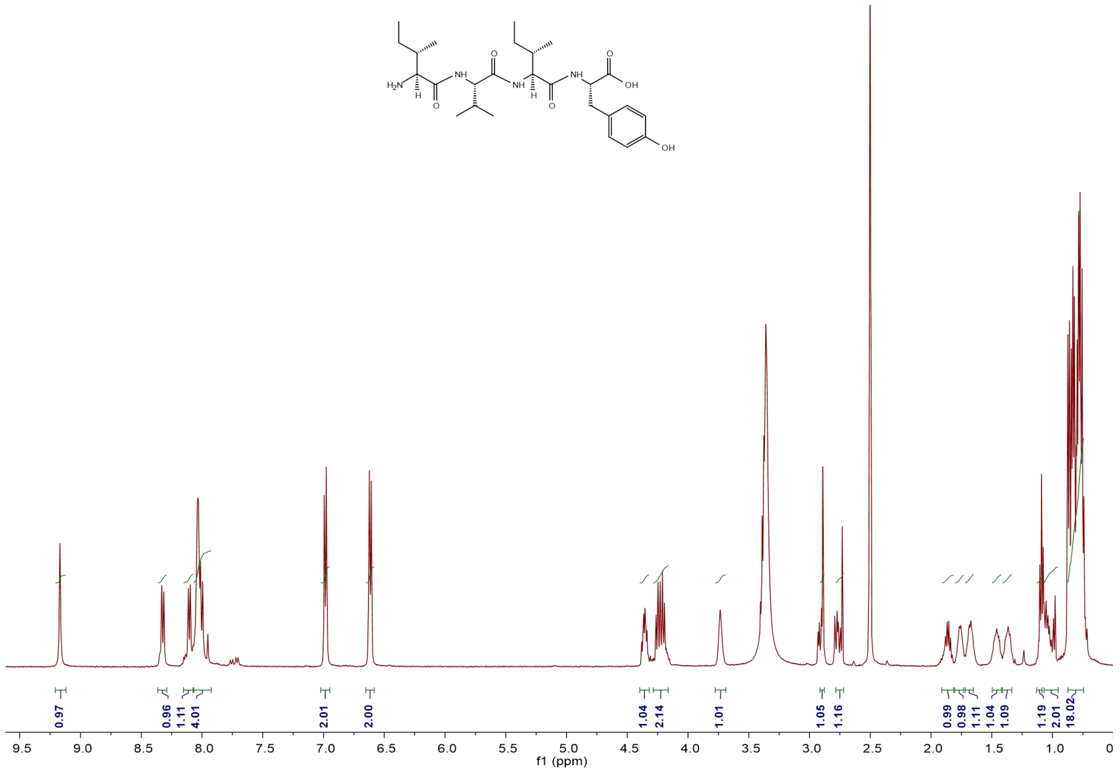

79.Compound **NTIF**: ^{1}H NMR (500 MHz, DMSO) δ 8.47 (t, *J* = 11.3 Hz, 1H), 8.23 (t, *J* = 10.5 Hz, 1H), 8.07 (d, *J* = 3.8 Hz, 3H), 7.75 – 7.62 (m, 2H), 7.22 (dd, *J* = 15.9, 9.2 Hz, 5H), 5.06 (s, 1H), 4.42 (dd, *J* = 13.6, 8.5 Hz, 1H), 4.32 (dt, *J* = 15.4, 7.7 Hz, 1H), 4.26 – 4.18 (m, 2H), 3.93 (dd, *J* = 11.3, 6.0 Hz, 1H), 3.05 (dd, *J* = 14.0, 5.3 Hz, 1H), 2.94 – 2.82 (m, 2H), 2.75 – 2.66 (m, 1H), 2.56 – 2.52 (m, 1H), 1.00 (dd, *J* = 11.4, 6.6 Hz, 4H), 0.82 – 0.71 (m, 7H).

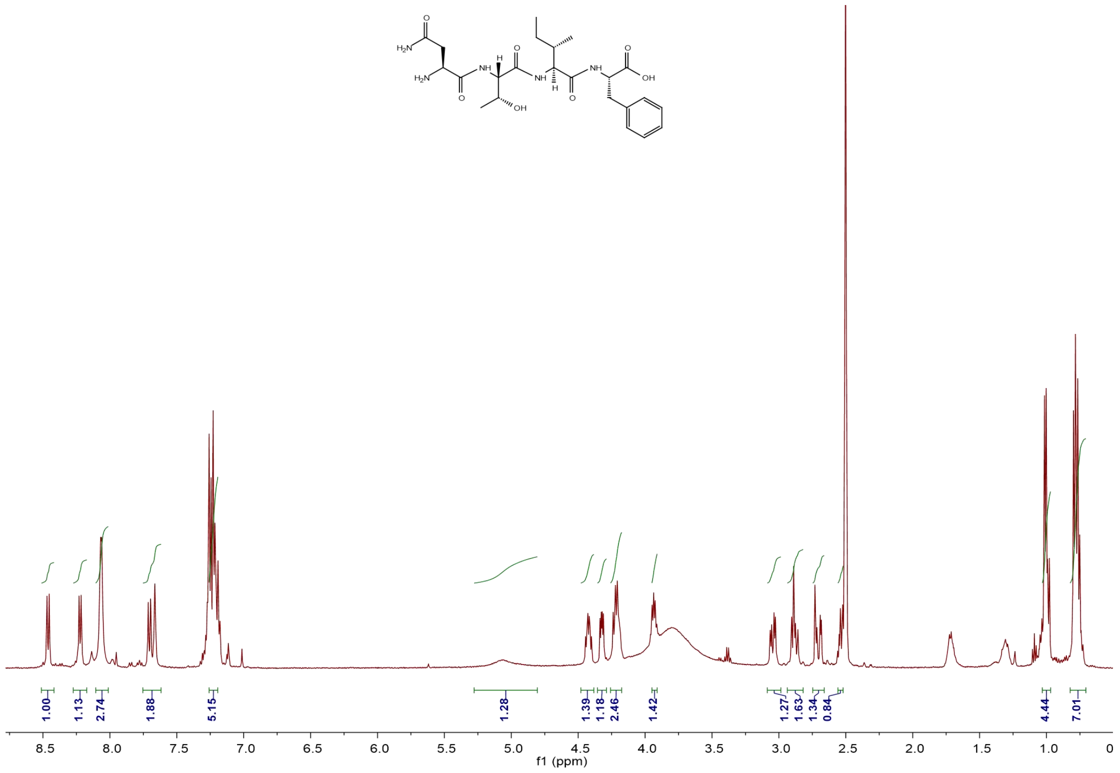

80.Compound **FVIY**: ^{1}H NMR (500 MHz, DMSO) δ 9.17 (s, 1H), 8.43 (d, *J* = 8.8 Hz, 1H), 8.10 (d, *J* = 7.7 Hz, 1H), 7.97 (d, *J* = 9.0 Hz, 1H), 7.26 (dt, *J* = 24.1, 6.9 Hz, 5H), 6.99 (d, *J* = 8.1 Hz, 2H), 6.62 (d, *J* = 8.1 Hz, 2H), 4.46 – 4.17 (m, 3H), 4.04 (s, 1H), 3.04 (dd, *J* = 13.9, 4.5 Hz, 1H), 2.89 (ddd, *J* = 20.4, 14.3, 6.3 Hz, 2H), 2.81 – 2.70 (m, 1H), 1.90 (tt, *J* = 16.6, 8.4 Hz, 1H), 1.69 (d, *J* = 6.5 Hz, 1H), 1.41 (d, *J* = 7.5 Hz, 1H), 1.14 – 0.95 (m, 1H), 0.80 (dd, *J* = 8.5, 5.1 Hz, 12H).

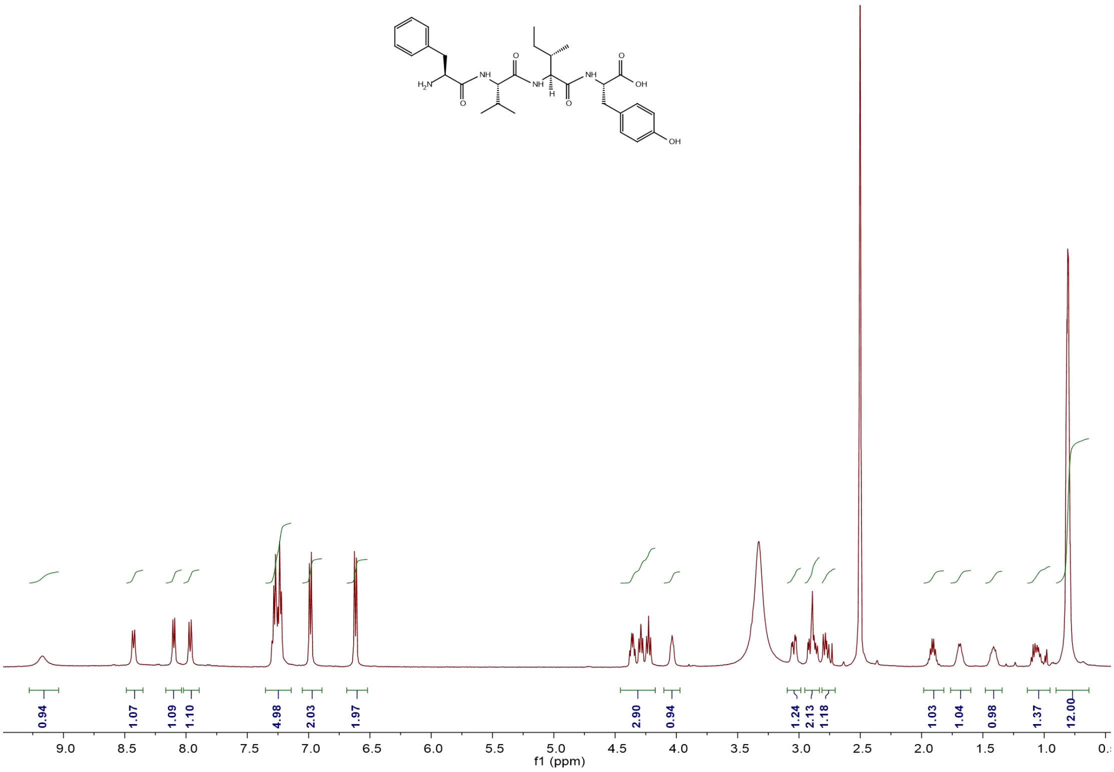

81.Compound **LLLF**: ^{1}H NMR (500 MHz, DMSO) δ 8.52 (t, *J* = 16.6 Hz, 1H), 8.32 (d, *J* = 7.8 Hz, 1H), 8.06 (s, 3H), 7.33 – 7.12 (m, 5H), 4.42 (ddd, *J* = 20.3, 14.2, 8.5 Hz, 2H), 3.76 (s, 1H), 3.06 (dd, *J* = 14.0, 4.8 Hz, 1H), 2.90 (dd, *J* = 14.0, 9.2 Hz, 1H), 1.60 (dtd, *J* = 19.5, 13.2, 6.6 Hz, 2H), 1.52 – 1.37 (m, 4H), 1.14 – 1.05 (m, 1H), 0.96 – 0.75 (m, 12H).

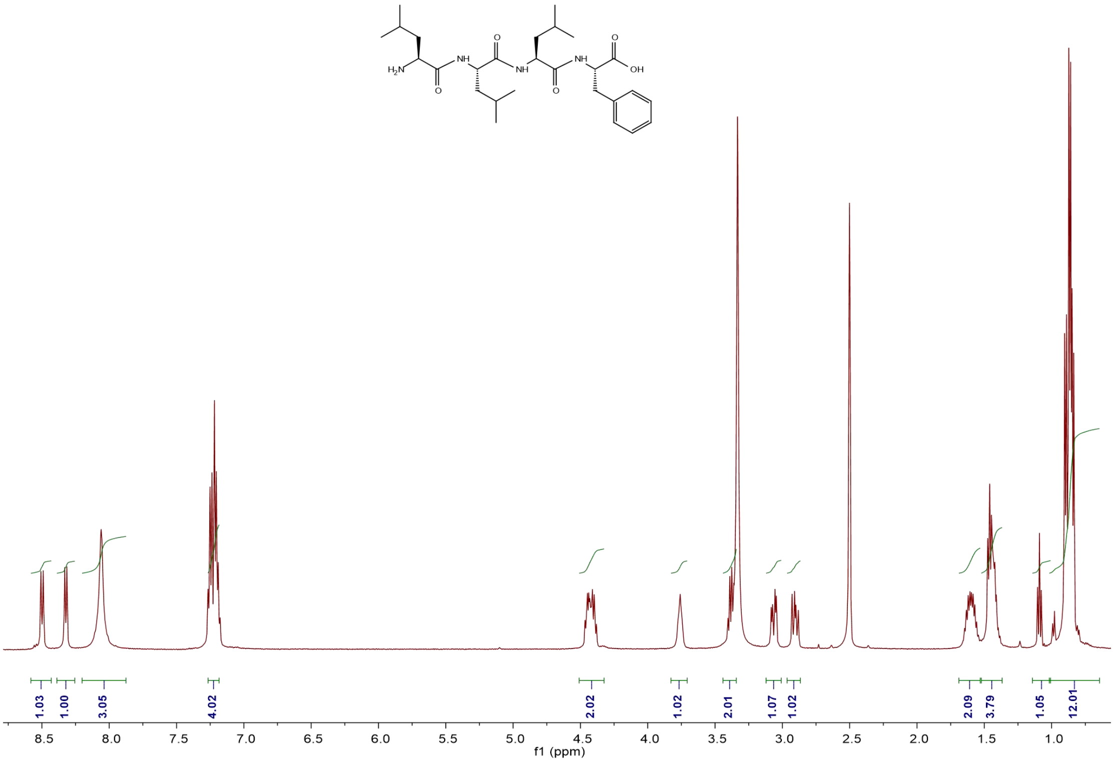

82.Compound **LVHF**: ^{1}H NMR (500 MHz, DMSO) δ 8.50 – 8.35 (m, 2H), 8.24 (dd, *J* = 20.9, 7.7 Hz, 2H), 7.22 (dq, *J* = 14.6, 7.5 Hz, 6H), 7.09 (s, 1H), 4.59 (dd, *J* = 14.0, 7.2 Hz, 1H), 4.44 (dd, *J* = 13.1, 8.0 Hz, 1H), 4.21 (t, *J* = 7.6 Hz, 1H), 3.88 (t, *J* = 7.0 Hz, 1H), 3.06 (dd, *J* = 14.0, 4.8 Hz, 2H), 3.00 – 2.82 (m, 4H), 1.92 (dq, *J* = 13.2, 6.5 Hz, 1H), 1.59 (td, *J* = 12.8, 6.3 Hz, 1H), 1.48 (t, *J* = 6.9 Hz, 2H), 0.88 – 0.81 (m, 12H).

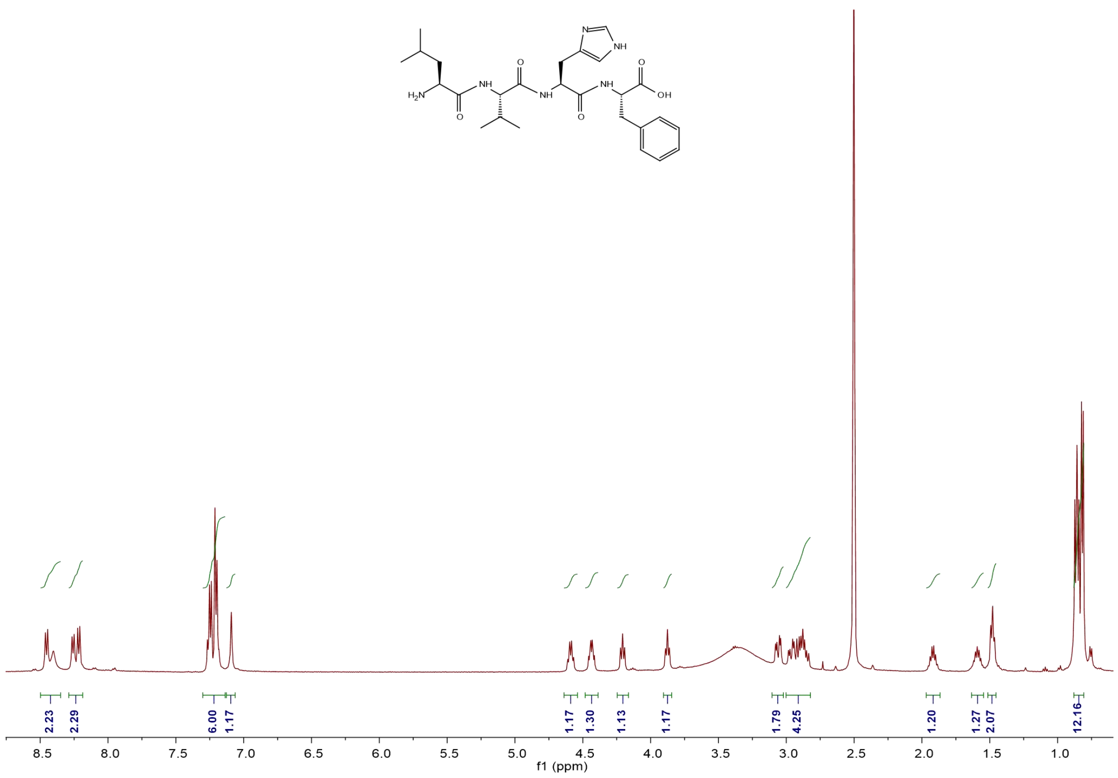

83.Compound **PIFY**: ^{1}H NMR (500 MHz, DMSO) δ 12.71 (s, 1H), 9.21 (s, 1H), 8.43 (d, *J* = 8.9 Hz, 1H), 8.15 (dd, *J* = 26.1, 8.1 Hz, 2H), 7.26 – 7.13 (m, 5H), 7.00 (d, *J* = 8.1 Hz, 2H), 6.64 (d, *J* = 8.1 Hz, 2H), 4.60 (td, *J* = 9.5, 4.1 Hz, 1H), 4.38 (dd, *J* = 13.4, 7.7 Hz, 1H), 4.25 – 4.12 (m, 2H), 3.19 (dd, *J* = 13.9, 7.4 Hz, 2H), 2.96 (ddd, *J* = 19.3, 14.0, 4.5 Hz, 2H), 2.77 (ddd, *J* = 24.1, 13.8, 9.2 Hz, 2H), 2.16 (td, *J* = 14.8, 7.6 Hz, 1H), 1.92 – 1.73 (m, 2H), 1.60 (ddd, *J* = 20.3, 16.0, 6.8 Hz, 2H), 1.35 (dd, *J* = 20.0, 12.3 Hz, 1H), 1.07 – 0.92 (m, 1H), 0.74 (dq, *J* = 20.8, 7.1 Hz, 6H).

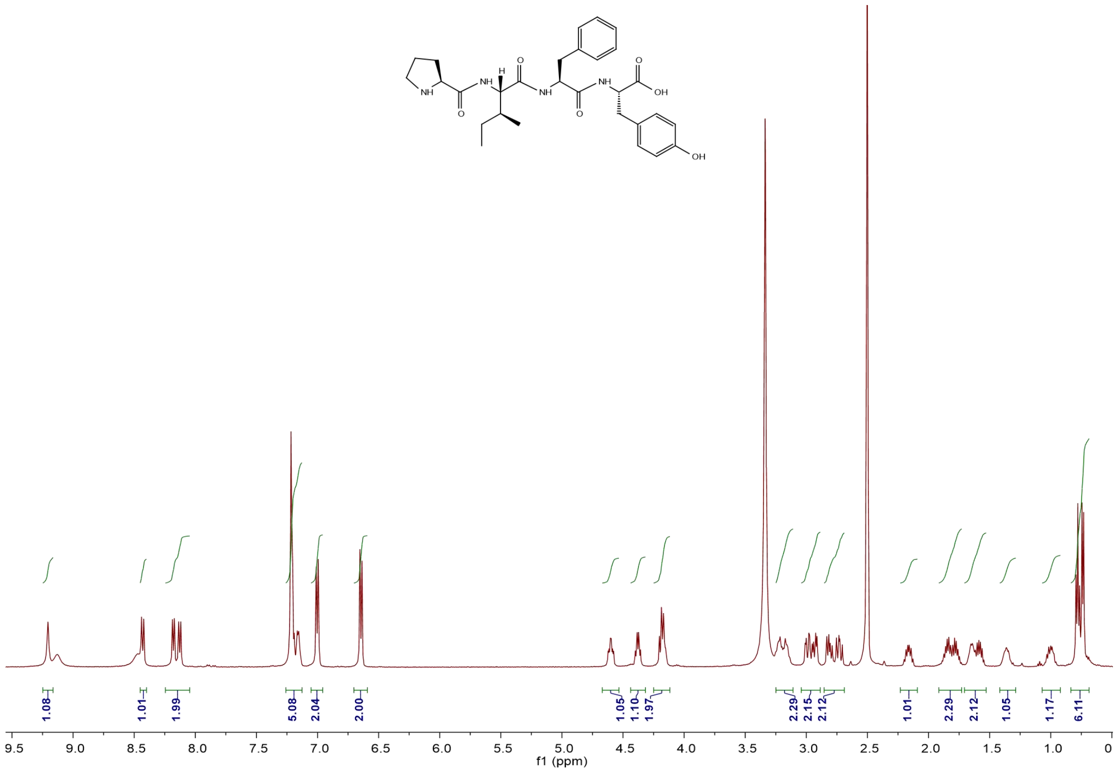

84.Compound **WIIF**: ^{1}H NMR (600 MHz, DMSO-d6) δ 8.20 (d, J = 7.8 Hz, 1H), 7.99 (d, J = 9.1 Hz, 1H), 7.72 (d, J = 7.9 Hz, 1H), 7.37 (d, J = 8.0 Hz, 1H), 7.27 – 7.16 (m, 6H), 7.10 (t, J = 7.5 Hz, 1H), 7.02 (q, J = 7.3, 6.6 Hz, 1H), 4.46 (td, J = 8.5, 5.1 Hz, 1H), 4.35 – 4.21 (m, 2H), 4.09 (dd, J = 8.9, 4.7 Hz, 1H), 3.17 (dd, J = 15.0, 4.7 Hz, 1H), 3.08 – 2.85 (m, 3H), 1.70 (ddq, J = 14.5, 7.4, 3.7 Hz, 2H), 1.52 – 1.37 (m, 2H), 1.12 – 1.02 (m, 2H), 0.86 – 0.73 (m, 12H).

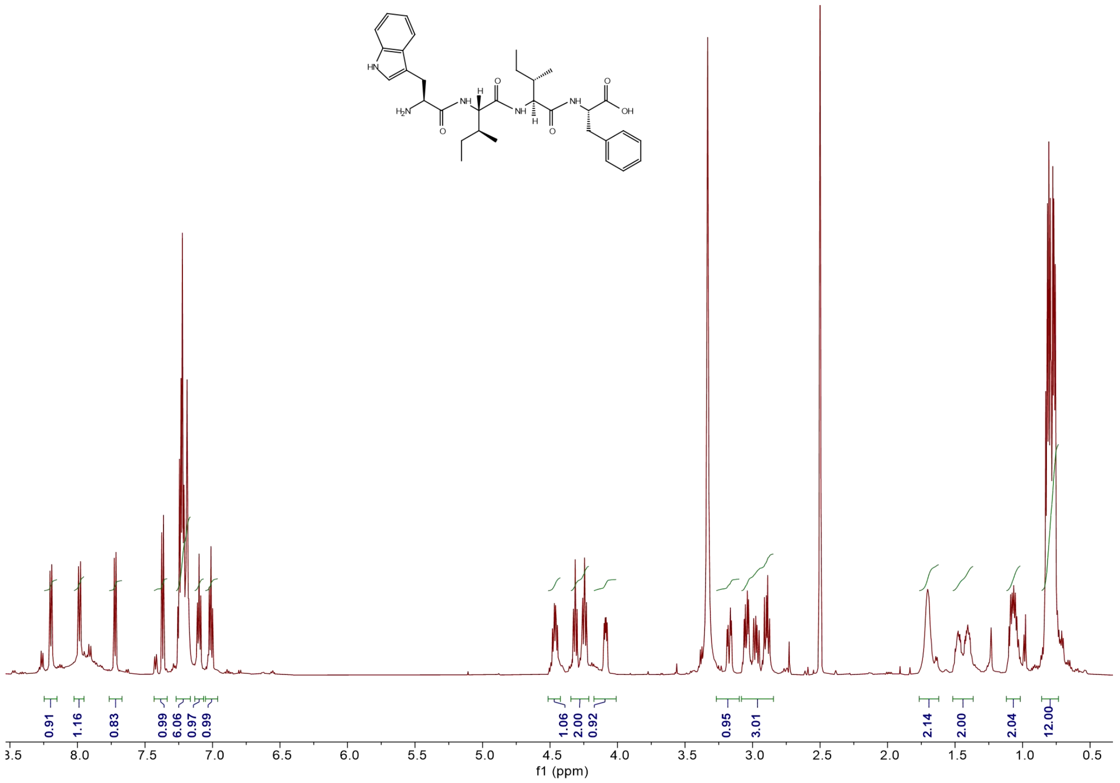

85.Compound **IFIY**: ^{1}H NMR (500 MHz, DMSO) δ 9.19 (s, 1H), 8.52 (d, J = 8.2 Hz, 1H), 8.14 (t, J = 9.5 Hz, 2H), 7.96 (s, 2H), 7.30 – 7.16 (m, 5H), 7.02 (d, J = 8.1 Hz, 2H), 6.64 (d, J = 8.1 Hz, 2H), 4.69 (dt, J = 29.7, 16.9 Hz, 1H), 4.34 (dd, J = 13.6, 7.9 Hz, 1H), 4.24 (dd, J = 16.4, 8.2 Hz, 1H), 3.60 (s, 1H), 2.96 – 2.87 (m, 2H), 2.83 – 2.74 (m, 2H), 1.84 (d, J = 4.6 Hz, 1H), 1.70 (d, J = 6.6 Hz, 1H), 1.40 (d, J = 7.0 Hz, 2H), 1.11 – 1.02 (m, 2H), 0.89 (t, J = 8.4 Hz, 3H), 0.83 – 0.76 (m, 9H).

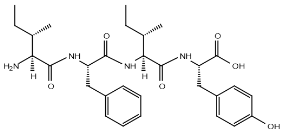

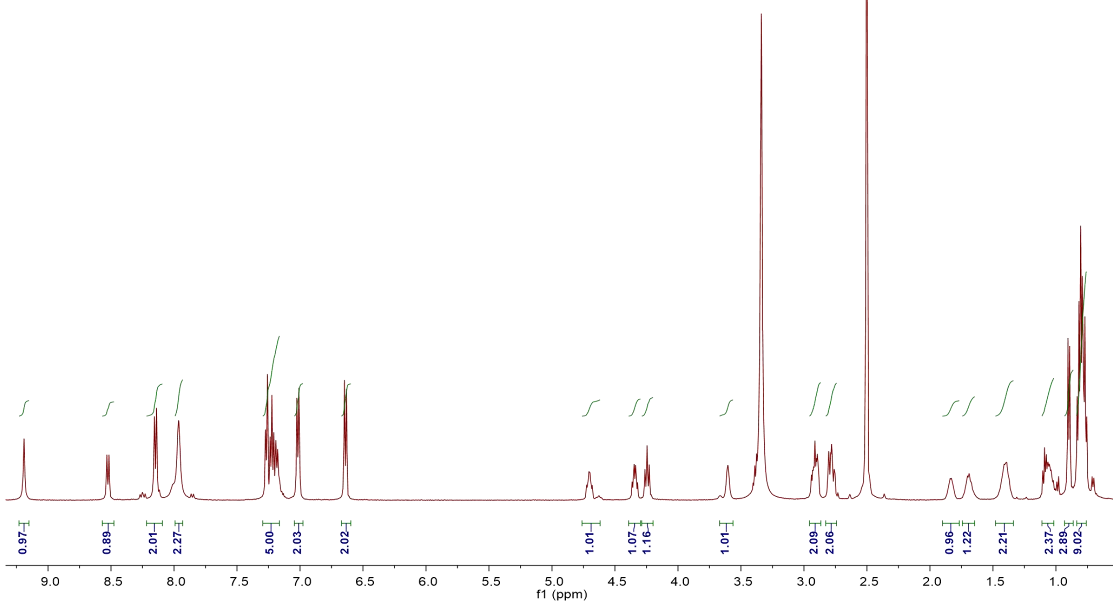

86.Compound **IVFW**: ^{1}H NMR (500 MHz, DMSO) δ 8.29 – 8.12 (m, 3H), 7.52 (d, *J* = 7.8 Hz, 1H), 7.33 (d, *J* = 8.0 Hz, 1H), 7.18 (dt, *J* = 18.8, 8.9 Hz, 6H), 7.06 (t, *J* = 7.5 Hz, 1H), 6.97 (t, *J* = 7.4 Hz, 1H), 4.62 (td, *J* = 9.0, 4.1 Hz, 1H), 4.50 (dd, *J* = 13.7, 7.0 Hz, 1H), 4.21 (dd, *J* = 20.4, 12.4 Hz, 1H), 3.63 (d, *J* = 5.3 Hz, 1H), 3.16 (dd, *J* = 14.7, 5.9 Hz, 1H), 3.03 (ddd, *J* = 18.0, 14.3, 5.6 Hz, 2H), 2.82 – 2.70 (m, 1H), 1.89 (dt, *J* = 13.8, 6.9 Hz, 1H), 1.67 (s, 1H), 1.38 (s, 1H), 1.02 – 0.96 (m, 1H), 0.78 (ddd, *J* = 26.6, 19.4, 9.9 Hz, 12H).

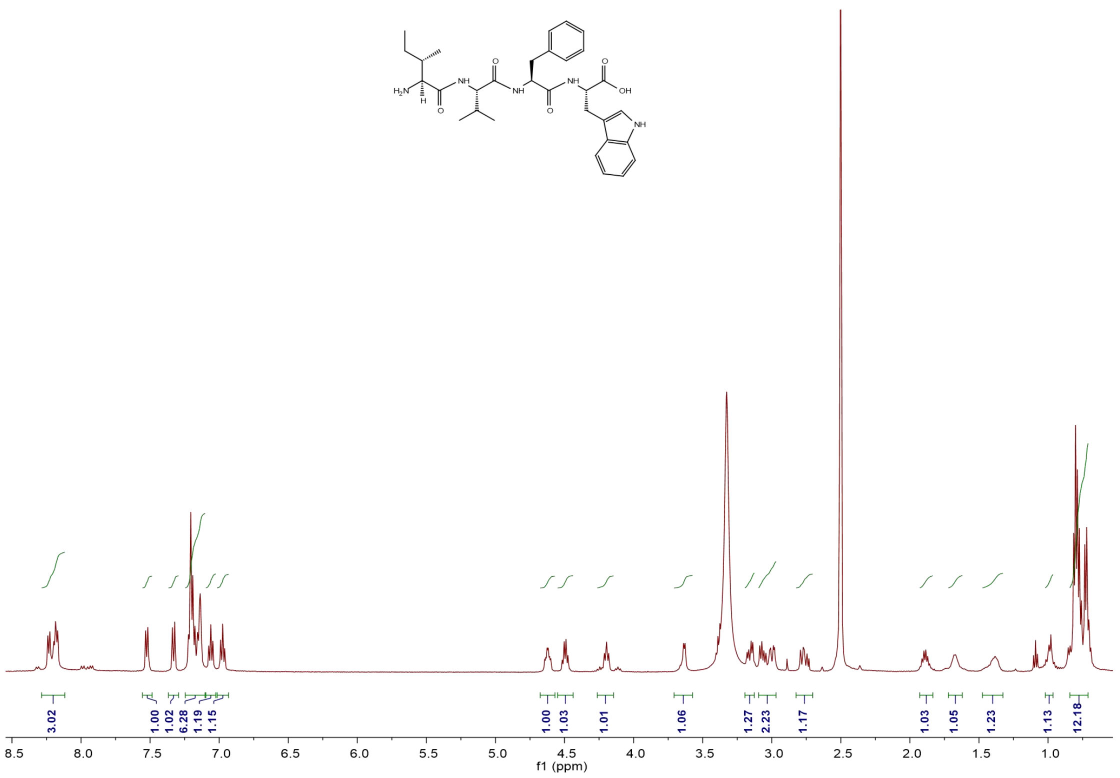

87.Compound **VLVV**: ^{1}H NMR (500 MHz, DMSO) δ 8.45 (d, *J* = 8.0 Hz, 1H), 8.05 (s, 3H), 7.86 (d, *J* = 8.2 Hz, 1H), 4.47 (dd, *J* = 15.1, 7.7 Hz, 1H), 4.27 (t, *J* = 8.1 Hz, 1H), 4.12 (dd, *J* = 8.0, 5.9 Hz, 1H), 3.64 – 3.59 (m, 1H), 2.05 (dq, *J* = 13.2, 6.6 Hz, 2H), 1.96 (dt, *J* = 13.7, 6.8 Hz, 1H), 1.61 (td, *J* = 13.2, 6.6 Hz, 1H), 1.45 (t, *J* = 7.1 Hz, 2H), 0.88 (tdd, *J* = 15.0, 9.0, 5.8 Hz, 24H).

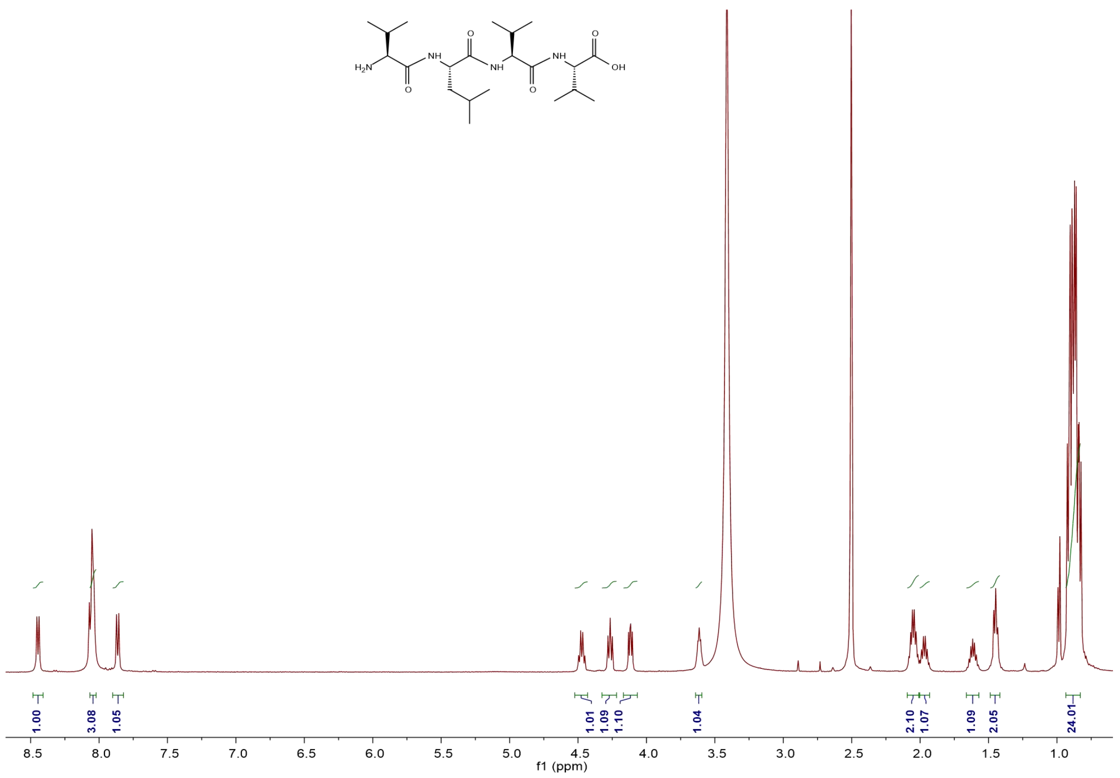

88.Compound **HLLF**: ^{1}H NMR (500 MHz, DMSO) δ 8.61 (d, *J* = 7.3 Hz, 1H), 8.39 (d, *J* = 8.3 Hz, 1H), 8.03 (d, *J* = 7.8 Hz, 1H), 7.34 (s, 1H), 7.29 – 7.15 (m, 5H), 4.44 (dd, *J* = 13.3, 8.0 Hz, 1H), 4.33 (dd, *J* = 15.0, 7.4 Hz, 2H), 4.14 (t, *J* = 5.8 Hz, 1H), 3.14 (d, *J* = 5.2 Hz, 2H), 3.06 (dd, *J* = 13.9, 5.0 Hz, 1H), 2.92 (dd, *J* = 15.0, 9.9 Hz, 1H), 1.60 (ddd, *J* = 19.9, 13.0, 6.4 Hz, 2H), 1.44 (dd, *J* = 14.8, 8.0 Hz, 4H), 0.93 – 0.77 (m, 12H).

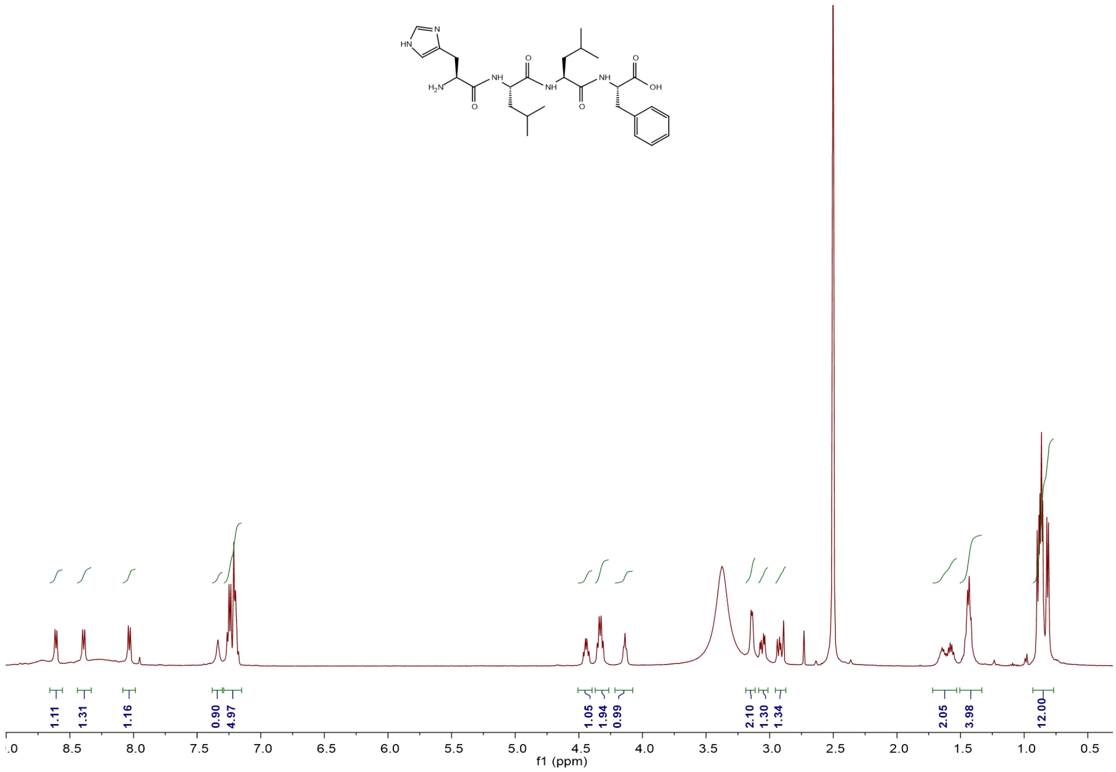

89.Compound **IVIF**: ^{1}H NMR (500 MHz, DMSO) δ 8.31 (d, J = 8.6 Hz, 1H), 8.20 (d, J = 7.8 Hz, 1H), 8.08 – 7.99 (m, 3H), 7.27 – 7.15 (m, 5H), 4.45 (dd, J = 13.4, 8.4 Hz, 1H), 4.28 – 4.17 (m, 2H), 3.76 – 3.71 (m, 1H), 3.05 (dd, J = 14.2, 4.8 Hz, 1H), 2.89 (dd, J = 14.2, 9.3 Hz, 1H), 1.91 – 1.80 (m, 1H), 1.76 (d, J = 5.6 Hz, 1H), 1.67 (d, J = 6.4 Hz, 1H), 1.46 (dd, J = 13.5, 7.3 Hz, 1H), 1.36 (d, J = 7.5 Hz, 1H), 1.06 (ddd, J = 21.3, 15.1, 8.3 Hz, 2H), 0.88 – 0.74 (m, 18H).

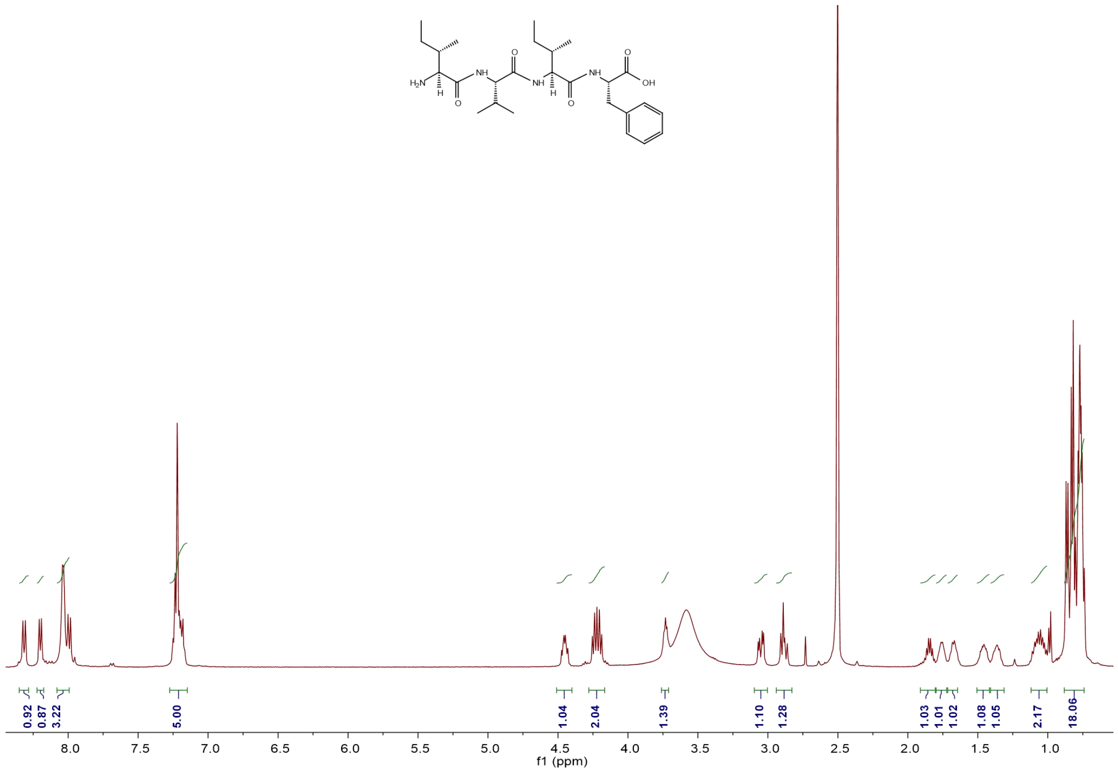

90.Compound **LLHF**: ^{1}H NMR (500 MHz, DMSO) δ 8.63 (s, 1H), 8.54 (d, *J* = 7.7 Hz, 1H), 8.28 (d, *J* = 8.0 Hz, 1H), 8.19 (t, *J* = 10.1 Hz, 1H), 7.29 – 7.24 (m, 2H), 7.24 – 7.10 (m, 5H), 4.58 (dd, *J* = 14.2, 7.4 Hz, 1H), 4.43 (dd, *J* = 12.9, 8.0 Hz, 1H), 4.34 (dd, *J* = 13.4, 8.8 Hz, 1H), 3.78 (t, *J* = 7.0 Hz, 1H), 3.08 – 2.99 (m, 2H), 2.94 – 2.86 (m, 2H), 1.68 – 1.57 (m, 2H), 1.53 – 1.34 (m, 4H), 0.87 (dd, *J* = 13.9, 6.6 Hz, 12H).

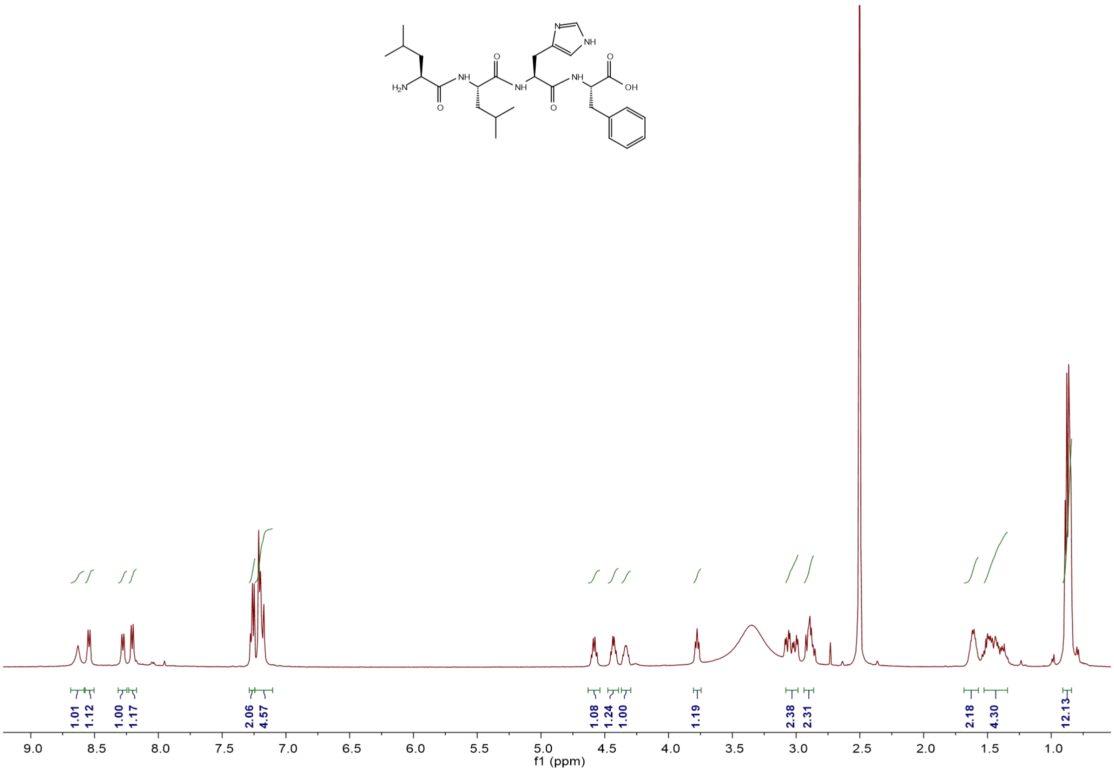

91.Compound **LLLY**: ^{1}H NMR (500 MHz, DMSO) δ 12.66 (s, 1H), 9.20 (s, 1H), 8.56 (d, J = 8.4 Hz, 1H), 8.13 (d, J = 8.5 Hz, 1H), 8.08 (s, 2H), 7.90 (d, J = 7.6 Hz, 1H), 6.98 (d, J = 8.0 Hz, 2H), 6.64 (d, J = 8.0 Hz, 2H), 4.42 (q, J = 7.5 Hz, 1H), 4.37 – 4.30 (m, 2H), 3.77 (d, J = 5.0 Hz, 1H), 2.98 – 2.85 (m, 2H), 1.63 – 1.37 (m, 9H), 0.86 (ddd, J = 33.9, 16.8, 4.5 Hz, 18H).

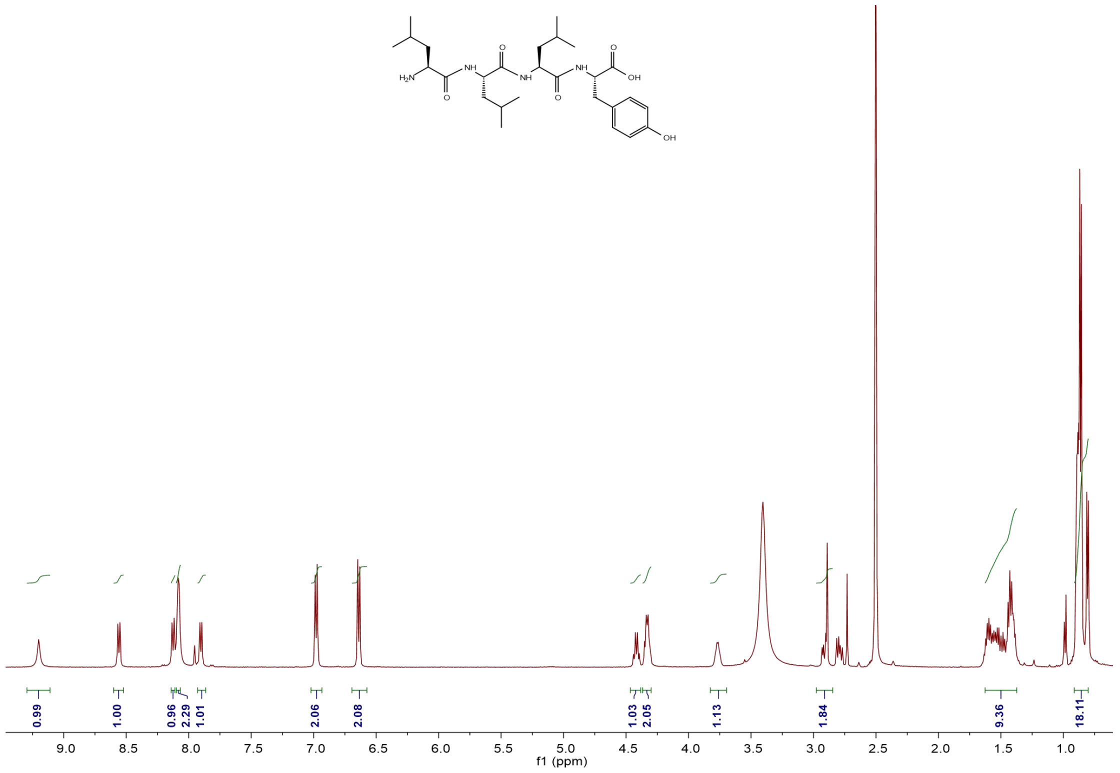

92.Compound **CIYF**: ^{1}H NMR (500 MHz, DMSO) δ 8.34 (t, J = 13.2 Hz, 1H), 8.24 (t, J = 11.3 Hz, 1H), 8.18 (s, 2H), 8.05 – 7.91 (m, 1H), 7.27 (ddd, J = 24.0, 15.9, 7.1 Hz, 5H), 7.08 – 6.93 (m, 2H), 6.61 (dd, J = 16.7, 8.0 Hz, 2H), 4.59 – 4.39 (m, 2H), 4.39 – 4.12 (m, 1H), 4.00 (s, 1H), 3.05 (dd, J = 13.9, 5.3 Hz, 1H), 2.97 – 2.79 (m, 4H), 2.70 – 2.58 (m, 1H), 1.66 (d, J = 6.2 Hz, 1H), 1.35 (d, J = 8.2 Hz, 1H), 1.02 (dd, J = 29.5, 7.4 Hz, 2H), 0.83 – 0.65 (m, 6H).

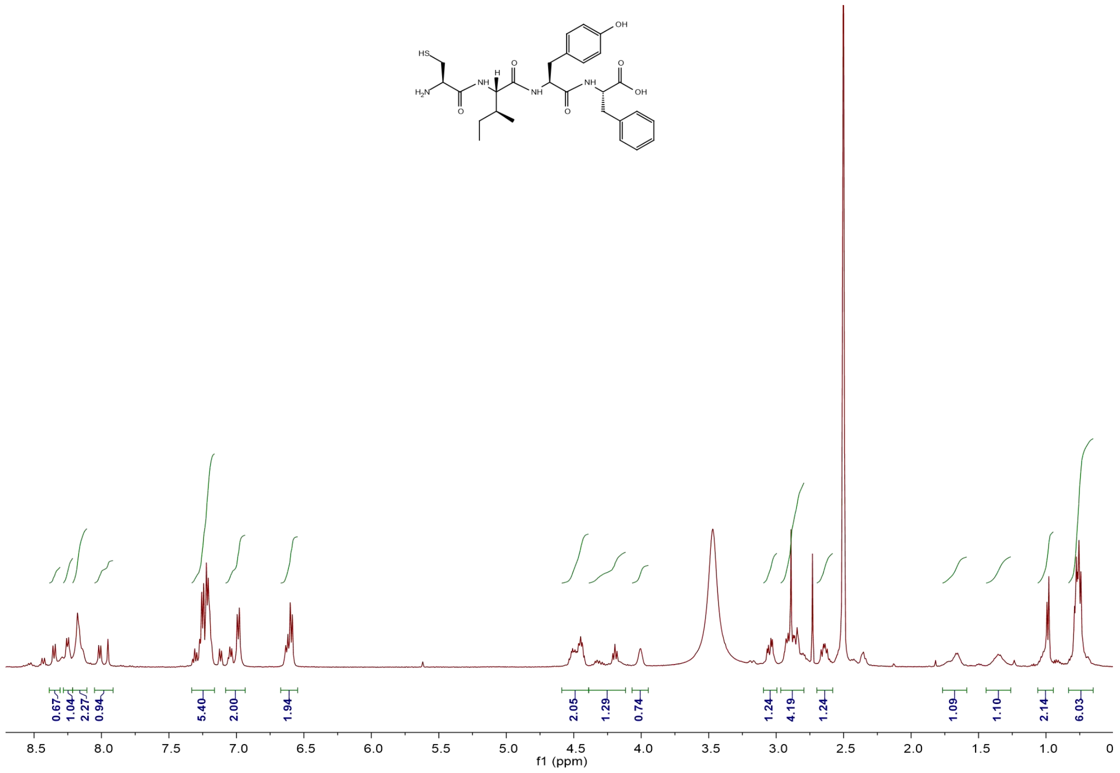

93.Compound **PVFW**: ^{1}H NMR (500 MHz, DMSO) δ 8.37 (d, *J* = 8.6 Hz, 1H), 8.25 (d, *J* = 7.5 Hz, 1H), 8.12 (d, *J* = 8.4 Hz, 1H), 7.53 (d, *J* = 7.8 Hz, 1H), 7.33 (d, *J* = 8.1 Hz, 1H), 7.21 (q, *J* = 7.6 Hz, 4H), 7.17 – 7.12 (m, 2H), 7.06 (t, *J* = 7.5 Hz, 1H), 6.98 (t, *J* = 7.4 Hz, 1H), 4.61 (td, *J* = 9.4, 4.2 Hz, 1H), 4.50 (dd, *J* = 13.5, 6.8 Hz, 1H), 4.16 (dd, *J* = 19.7, 11.9 Hz, 2H), 3.16 (dt, *J* = 16.8, 8.4 Hz, 3H), 3.05 (ddd, *J* = 17.7, 14.4, 5.6 Hz, 2H), 2.78 – 2.71 (m, 1H), 2.16 (dd, *J* = 12.7, 6.9 Hz, 1H), 1.91 (dt, *J* = 13.6, 6.8 Hz, 1H), 1.84 – 1.72 (m, 2H), 1.68 – 1.58 (m, 1H), 0.77 (t, *J* = 7.2 Hz, 6H).

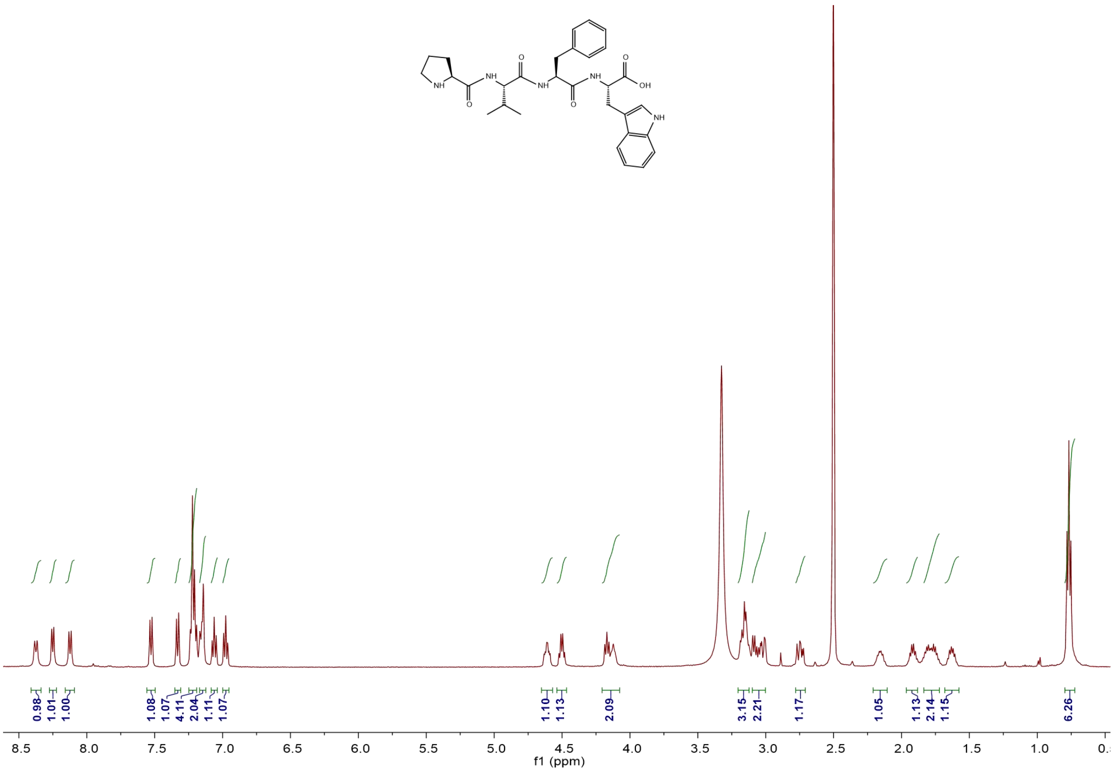

94.Compound **PVII**: ^{1}H NMR (500 MHz, DMSO) δ 8.55 (d, J = 8.6 Hz, 1H), 7.96 (t, J = 9.5 Hz, 2H), 4.33 – 4.20 (m, 3H), 4.19 – 4.11 (m, 1H), 3.24 (dd, J = 11.9, 5.9 Hz, 1H), 3.18 (dd, J = 16.2, 9.3 Hz, 1H), 2.34 – 2.22 (m, 1H), 1.97 (td, J = 13.6, 6.9 Hz, 1H), 1.93 – 1.65 (m, 5H), 1.49 – 1.34 (m, 2H), 1.19 (dt, J = 14.1, 7.7 Hz, 1H), 1.13 – 1.02 (m, 1H), 0.82 (ddd, J = 18.7, 15.4, 7.9 Hz, 18H).

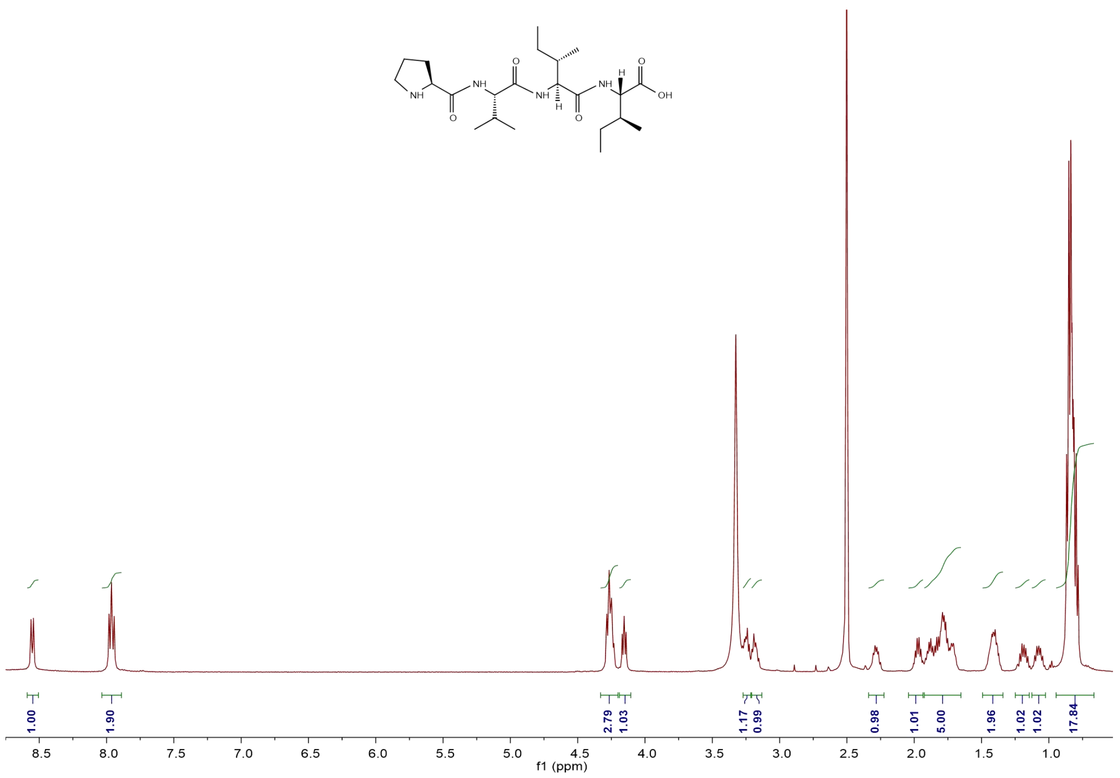

95.Compound **PWYY**: ^{1}H NMR (500 MHZ, DMSO-d6) δ 10.80 (d, J = 2.4 Hz, 1H), 9.21 (s, 1H), 9.15 (s, 1H), 8.61 (d, J = 8.3 Hz, 1H), 8.22 (d, J = 7.7 Hz, 1H), 8.11 (d, J = 8.2 Hz, 1H), 7.63 (d, J = 7.9 Hz, 1H), 7.32 (D, J = 8.1 Hz, 1H), 7.05 (m, 6H), 6.96 (ddd, J = 7.4, 6.9, 1.0 Hz, 1H), 6.66 (d, J = 8.4 Hz, 2H), 6.61 (d, J = 8.4 Hz, 2H), 4.60 (td, J = 8.9, 4.5 Hz, 1H), 4.49 (td, J = 8.8, 4.2 Hz, 1H), 4.38 (td, J = 7.9, 5.5 Hz, 1H), 4.02 (d, J = 7.1 Hz, 1H), 3.12 (m, 4H), 2.88 (m, 5H), 2.67 (dt, J = 14.1, 9.5 Hz, 1H), 2.20 (td, J = 15.5, 12.6, 7.4 Hz, 1H), 1.76 (m, 4H).

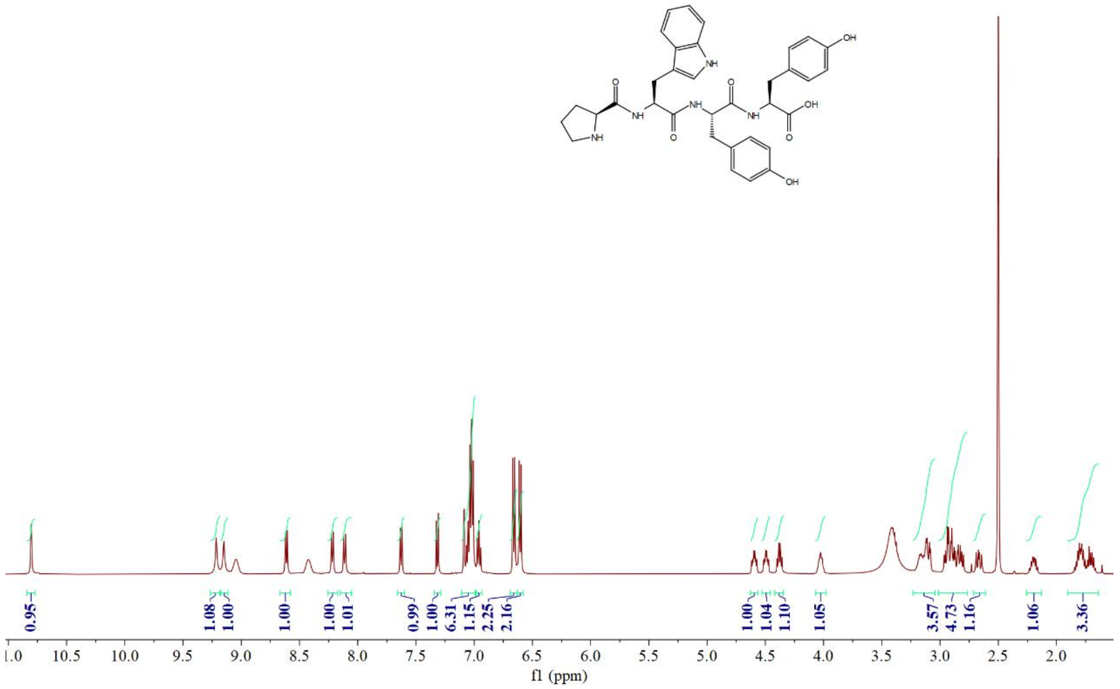

96.Compound **WAFY**: ^{1}H NMR (500 MHZ, DMSO-d6) δ 10.98 (d, J = 2.4 Hz, 1H), 9.22 (s, 1H), 8.74 (d, J = 7.6 Hz, 1H), 8.23 (d, J = 7.8 Hz, 1H), 8.11 (d, J = 8.3 Hz, 1H), 7.94 (m, 3H), 7.68 (d, J = 7.9 Hz, 1H), 7.36 (D, J = 8.1 Hz, 1H), 7.24 (m, 5H), 7.15 (m, 2H), 7.08 (ddd, J = 8.1, 6.9, 1.1 Hz, 1H), 7.02 (m, 2H), 6.94 (ddd, J = 8.0, 7.0, 1,0 Hz, 1H), 6.66 (m, 2H), 4.58 (td, J = 8.8, 4.5 Hz, 1H), 4.37 (td, J = 8.3, 7.8, 5.2 Hz, 2H), 4.01 (m, 1H), 3.14 (dd, J = 14.9, 4.6 Hz, 1H), 3.04 (dd, J = 14.0, 4.5 Hz, 1H), 2.95 (m, 2H), 2.79 (m, 2H), 1.21 (d, J = 7.0 Hz, 3H).

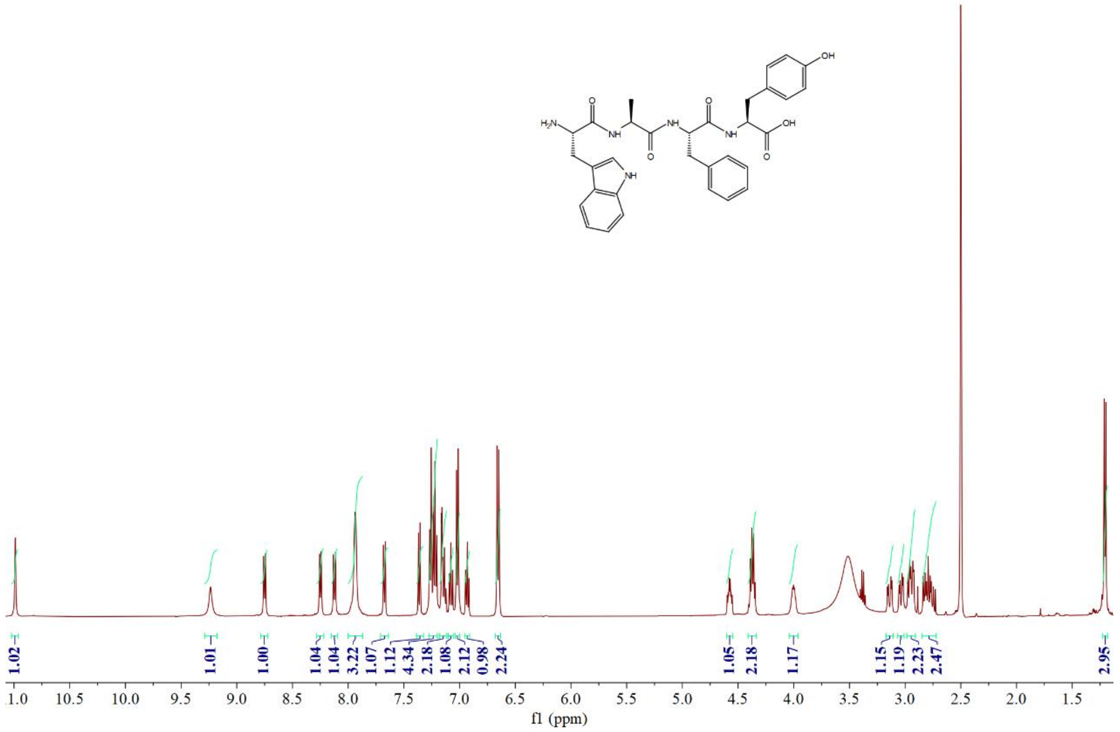

97.Compound **FHLF**: ^{1}H NMR (500 MHz, DMSO-d6) δ 8.65 (d, J = 7.8 Hz, 1H), 8.53 (d, J = 7.8 Hz, 1H), 8.17 (d, J = 8.2 Hz, 1H), 7.67 (s, 1H), 7.31 – 7.17 (m, 10H), 6.86 (s, 1H), 4.58 (q, J = 7.3 Hz, 1H), 4.44 (td, J = 8.4, 5.4 Hz, 1H), 4.33 (td, J = 9.3, 8.7, 4.8 Hz, 1H), 4.06 (dd, J = 7.7, 5.3 Hz, 1H), 3.07 (dt, J = 12.9, 6.0 Hz, 2H), 3.00 – 2.83 (m, 4H), 1.56 (dq, J = 12.9, 6.6 Hz, 1H), 1.50 – 1.35 (m, 2H), 0.85 (dd, J = 22.2, 6.5 Hz, 6H).

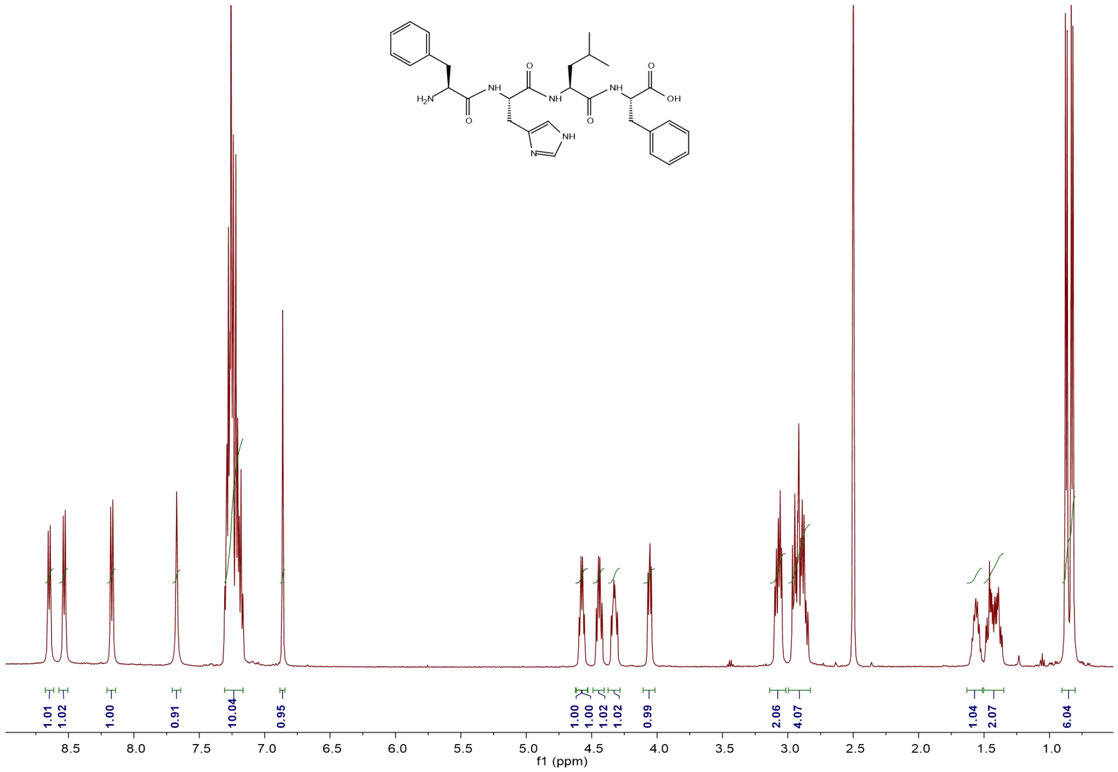

98.Compound **LIEM**: ^{1}H NMR (500 MHz, DMSO) δ 8.50 (d, J = 8.6 Hz, 1H), 8.17 (dt, J = 18.9, 9.4 Hz, 2H), 8.08 (d, J = 3.2 Hz, 2H), 4.33 – 4.24 (m, 3H), 3.85 (d, J = 5.4 Hz, 1H), 2.49 – 2.39 (m, 2H), 2.23 (dd, J = 16.5, 6.9 Hz, 2H), 2.06 – 1.99 (m, 3H), 1.97 (d, J = 8.1 Hz, 1H), 1.92 – 1.81 (m, 2H), 1.78 – 1.68 (m, 2H), 1.60 (dt, J = 13.0, 6.6 Hz, 1H), 1.54 – 1.47 (m, 3H), 1.12 – 1.04 (m, 1H), 0.90 – 0.83 (m, 12H).

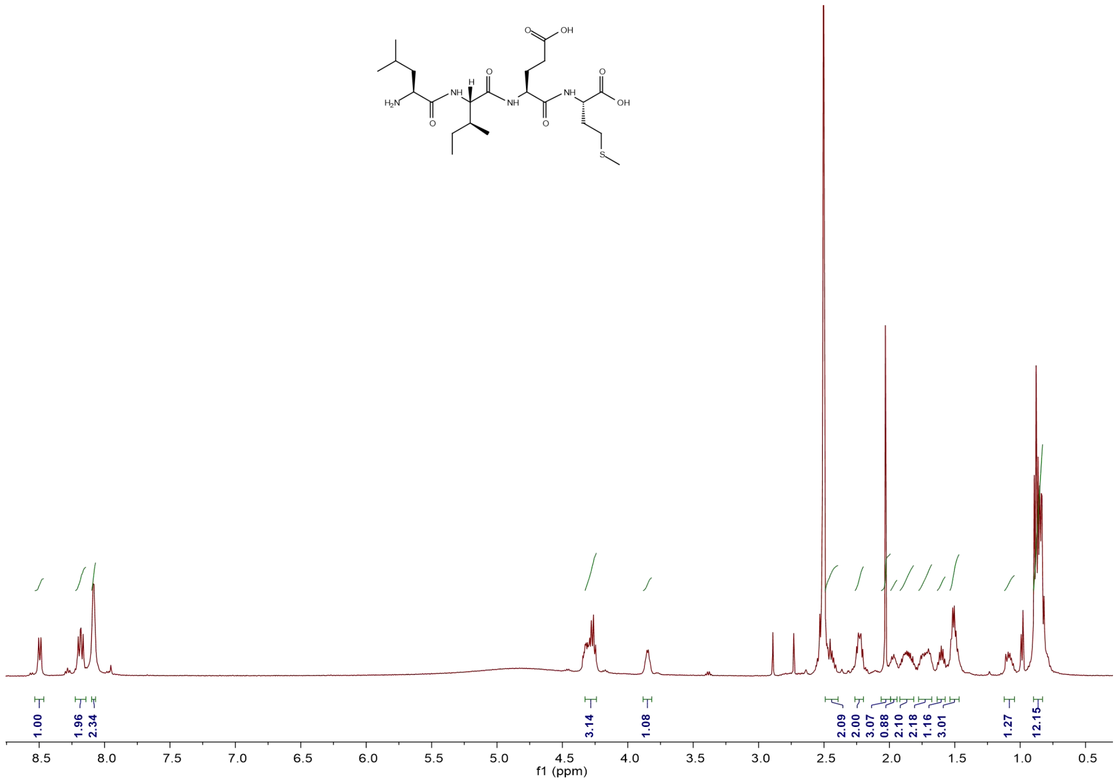

99.Compound **PMFY**: ^{1}H NMR (500 MHz, DMSO) δ 9.14 (s, 1H), 8.59 (d, *J* = 8.1 Hz, 1H), 8.49 (s, 1H), 8.28 (d, *J* = 7.6 Hz, 1H), 8.07 (t, *J* = 13.5 Hz, 1H), 7.29 – 7.17 (m, 5H), 7.01 (d, *J* = 8.0 Hz, 2H), 6.66 (d, *J* = 8.0 Hz, 2H), 4.55 (td, *J* = 9.1, 4.1 Hz, 1H), 4.37 (d, *J* = 7.2 Hz, 2H), 4.22 – 4.10 (m, 1H), 3.23 – 3.12 (m, 2H), 3.02 (dd, *J* = 13.8, 3.6 Hz, 1H), 2.98 – 2.87 (m, 2H), 2.86 – 2.69 (m, 3H), 2.27 – 2.13 (m, 1H), 2.00 (d, *J* = 9.0 Hz, 3H), 1.89 – 1.71 (m, 5H).

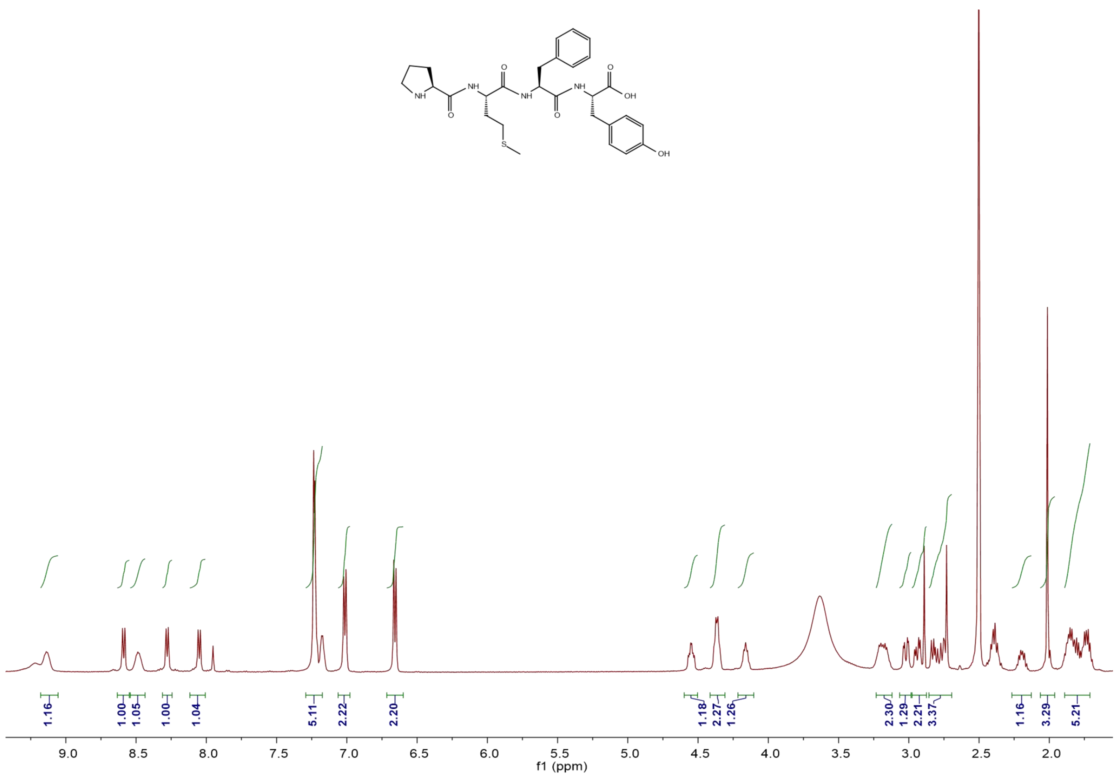

100.Compound **FCWW**: ^{1}H NMR (500 MHz, DMSO-d6) δ 8.66 (d, J = 8.1 Hz, 1H), 8.28 (dd, J = 17.9, 7.7 Hz, 2H), 7.64 (d, J = 7.9 Hz, 1H), 7.52 (d, J = 7.9 Hz, 1H), 7.35 – 7.13 (m, 9H), 7.05 (q, J = 8.0 Hz, 2H), 6.97 (t, J = 7.5 Hz, 2H), 4.63 (td, J = 8.2, 4.8 Hz, 1H), 4.49 (dq, J = 19.4, 6.9 Hz, 2H), 4.11 – 3.99 (m, 1H), 3.16 (dt, J = 14.9, 5.9 Hz, 2H), 3.10 – 3.05 (m, 1H), 2.97 (dd, J = 15.7, 6.1 Hz, 1H), 2.90 – 2.80 (m, 2H), 2.72 (d, J = 7.9 Hz, 2H).

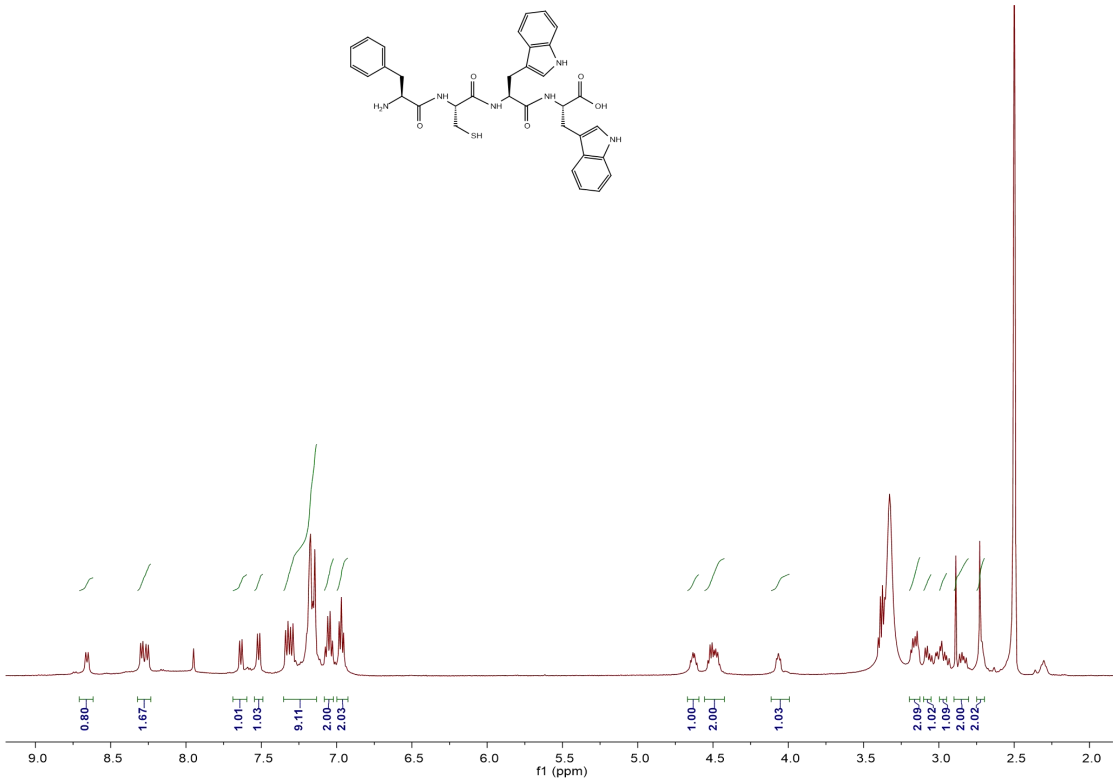

101.Compound **FGWW**: ^{1}H NMR (500 MHz, DMSO-d6) δ 8.59 (t, J = 5.5 Hz, 1H), 8.39 (d, J = 7.6 Hz, 1H), 8.16 (d, J = 8.3 Hz, 1H), 7.63 (d, J = 7.9 Hz, 1H), 7.54 (d, J = 7.9 Hz, 1H), 7.35 – 7.22 (m, 7H), 7.15 (dd, J = 15.8, 2.3 Hz, 2H), 7.06 (q, J = 7.0 Hz, 2H), 6.98 (q, J = 7.2 Hz, 2H), 4.64 (td, J = 8.6, 4.4 Hz, 1H), 4.51 (q, J = 7.6 Hz, 1H), 4.04 (s, 1H), 3.85 (dd, J = 16.8, 5.7 Hz, 1H), 3.62 (d, J = 5.2 Hz, 1H), 3.17 (ddd, J = 22.3, 14.7, 4.9 Hz, 2H), 3.06 (dt, J = 13.2, 6.5 Hz, 2H), 2.94 – 2.85 (m, 2H).

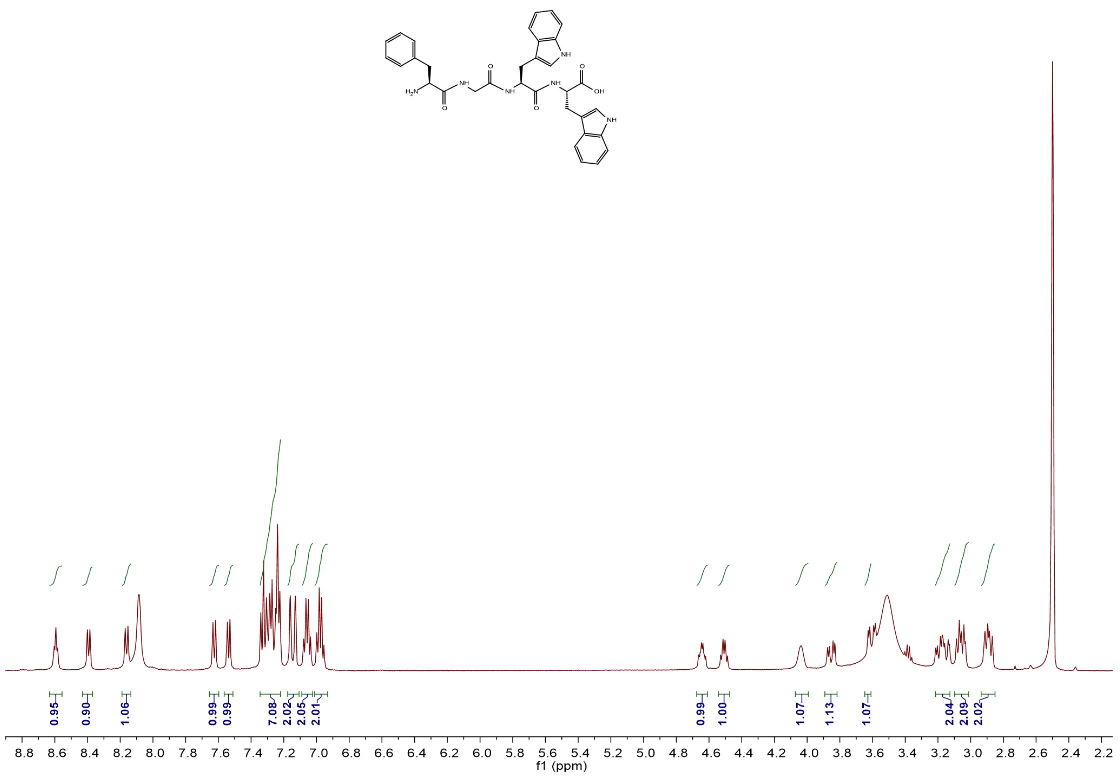

102.Compound **VWIT**: ^{1}H NMR (500 MHZ, DMSO-d6) δ 10.80 (d, *J* = 2.4 Hz, 1H), 8.54 (d, *J* = 7.9 Hz, 1H), 8.30 (d, *J* = 8.9 Hz, 1H), 7.97 (d, *J* = 5.5 Hz, 3H), 7.79 (d, *J* = 8.5 Hz, 1H), 7.67 (d, *J* = 7.9 Hz, 1H), 7.32 (d, *J* = 8.2 Hz, 1H), 7.16 (d, *J* = 2.4 Hz, 1H), 7.06 (m, 1H), 6.98 (m, 1H), 4.74 (m, 1H), 4.34 (t, *J* = 8.4 Hz, 1H), 4.23 (dd, *J* = 8.5, 3.2 Hz, 1H), 4.15 (m, 1H), 3.59 (t, *J* = 5.4 Hz, 1H), 3.10 (dd, *J* = 14.8, 5.0 Hz, 1H), 2.94 (dd, *J* = 14.9, 9.2 Hz, 1H), 2.09 (m, 1H), 1.80 (m, 1H), 1.46 (m, 1H), .05 (d, *J* = 6.4 Hz, 3H), 0.94 (d, *J* = 6.9 Hz, 3H), 0.87 (dd, *J* = 9.5, 6.8 Hz, 6H), 0.80 (t, *J* = 7.4 Hz, 3H).

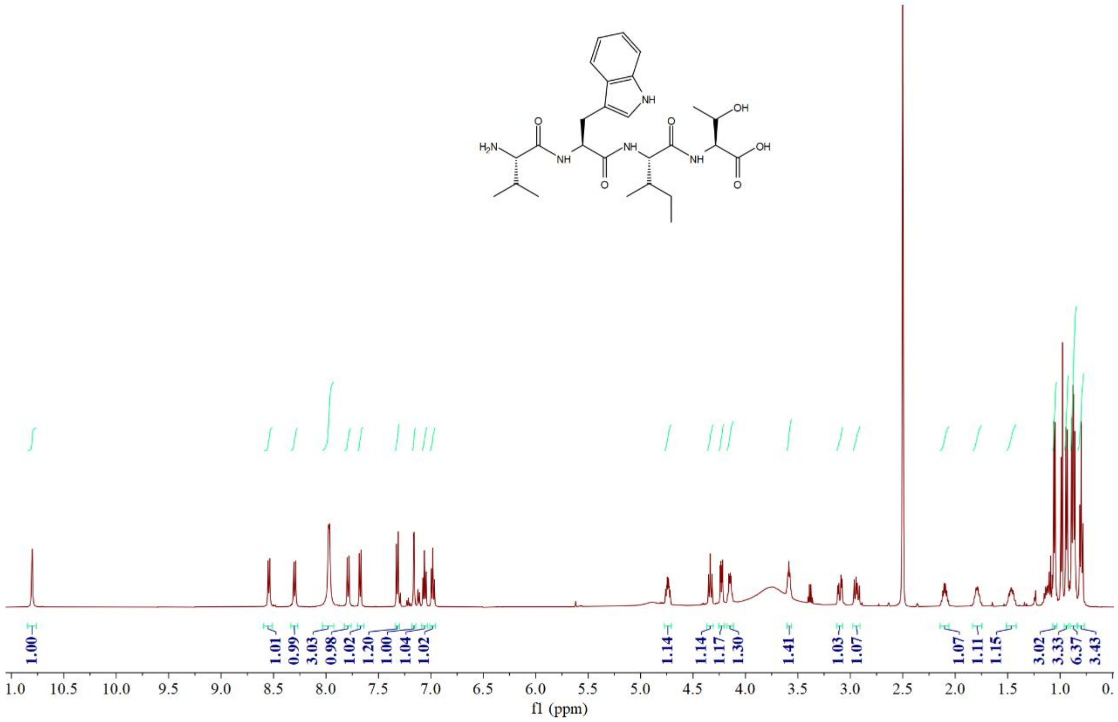

103.Compound **WWCP**: ^{1}H NMR (500 MHz, DMSO-d6) δ 8.82 (d, J = 7.5 Hz, 1H), 8.56 (d, J = 8.0 Hz, 1H), 7.71 (d, J = 8.0 Hz, 1H), 7.59 (d, J = 7.9 Hz, 1H), 7.35 (dd, J = 21.6, 8.1 Hz, 2H), 7.18 – 6.87 (m, 6H), 4.70 (q, J = 7.1 Hz, 1H), 4.63 (q, J = 7.2 Hz, 1H), 4.26 – 4.16 (m, 1H), 3.99 (dd, J = 9.2, 4.7 Hz, 1H), 3.26 – 3.19 (m, 2H), 3.17 – 2.96 (m, 4H), 2.82 (dd, J = 13.4, 7.2 Hz, 2H), 1.95 – 1.67 (m, 4H).

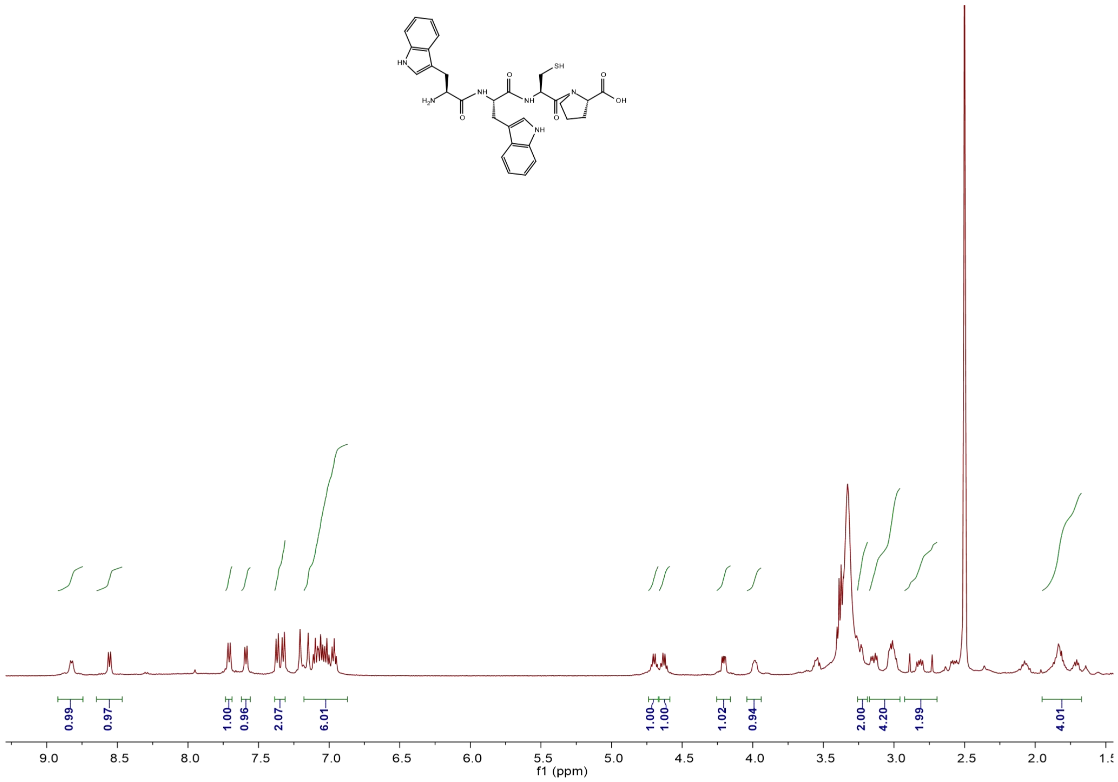

104.Compound **IWPW**: ^{1}H NMR (500 MHz, DMSO) δ 10.92 (s, 1H), 10.85 (s, 1H), 8.75 (d, *J* = 7.5 Hz, 1H), 8.03 (s, 2H), 7.58 (t, *J* = 7.9 Hz, 1H), 7.56 – 7.51 (m, 1H), 7.32 (dd, *J* = 19.0, 10.2 Hz, 3H), 7.19 (s, 1H), 7.08 – 6.96 (m, 5H), 4.78 (dd, *J* = 13.9, 7.3 Hz, 1H), 4.50 (dd, *J* = 13.5, 7.1 Hz, 1H), 4.42 (dd, *J* = 8.4, 3.5 Hz, 1H), 3.63 (s, 1H), 3.60 – 3.55 (m, 1H), 3.14 (dd, *J* = 10.0, 5.4 Hz, 2H), 3.10 – 3.07 (m, 1H), 2.97 (dd, *J* = 14.7, 7.7 Hz, 1H), 1.97 (dd, *J* = 11.7, 8.1 Hz, 1H), 1.81 – 1.74 (m, 3H), 1.49 – 1.40 (m, 2H), 1.12 – 1.08 (m, 1H), 0.84 (dd, *J* = 15.4, 7.8 Hz, 6H).

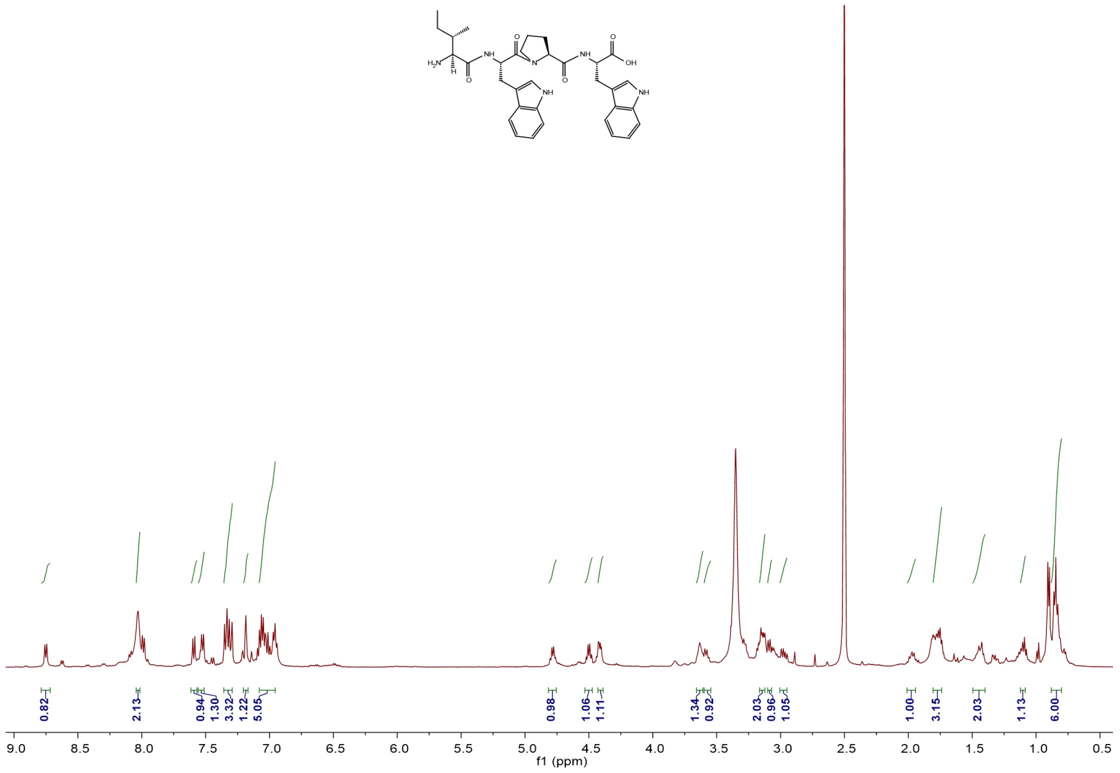

105.Compound **WPYY**: ^{1}H NMR (500 MHz, DMSO) δ 10.98 (t, *J* = 39.4 Hz, 2H), 9.15 (s, 1H), 8.09 (d, *J* = 7.3 Hz, 1H), 8.02 (d, *J* = 8.9 Hz, 1H), 7.79 (d, *J* = 8.0 Hz, 1H), 7.61 (d, *J* = 7.8 Hz, 1H), 7.36 (d, *J* = 7.6 Hz, 2H), 7.01 (dd, *J* = 17.4, 9.0 Hz, 5H), 6.61 (dt, *J* = 10.8, 7.4 Hz, 6H), 4.46 (dd, *J* = 12.9, 8.2 Hz, 1H), 4.32 (d, *J* = 5.9 Hz, 1H), 4.19 (d, *J* = 6.7 Hz, 1H), 4.13 (d, *J* = 5.6 Hz, 1H), 3.06 – 3.02 (m, 2H), 2.95 (d, *J* = 9.2 Hz, 2H), 2.86 – 2.82 (m, 2H), 2.74 – 2.68 (m, 2H), 1.97 (d, *J* = 11.7 Hz, 1H), 1.75 (d, *J* = 5.3 Hz, 3H).

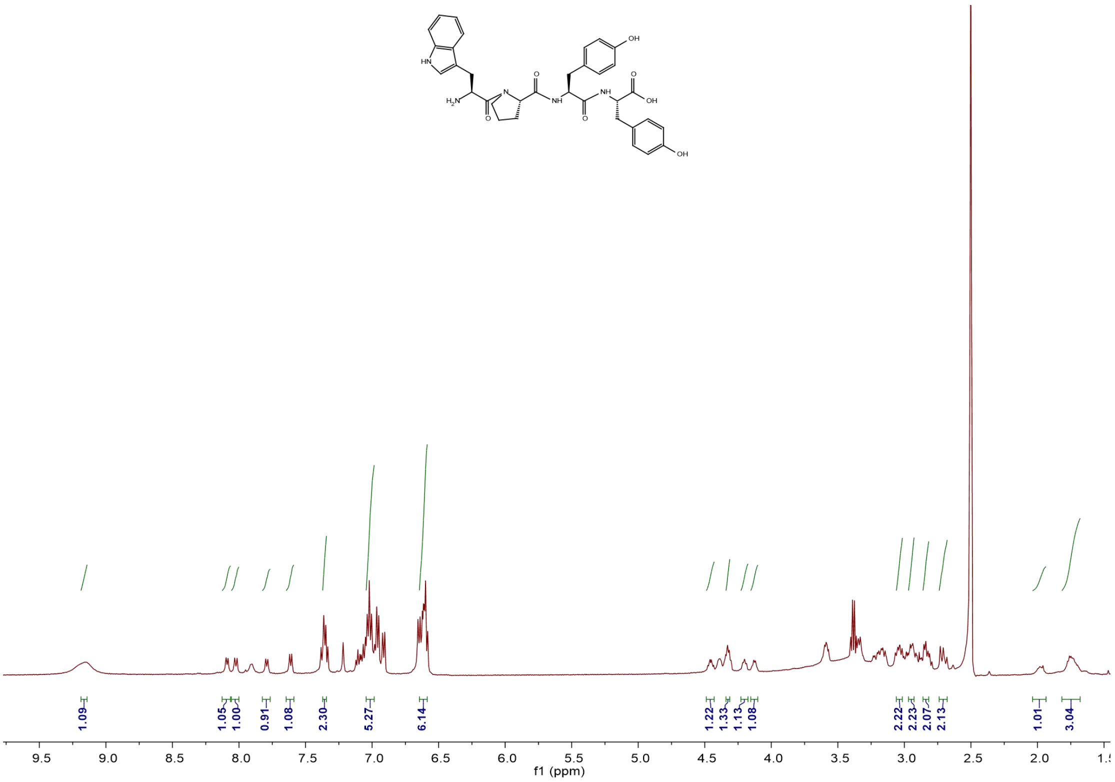

106.Compound **VSWY**: ^{1}H NMR (500 MHz, DMSO) δ 9.22 (s, 1H), 8.43 (d, *J* = 7.7 Hz, 1H), 8.12 (dd, *J* = 17.7, 7.8 Hz, 2H), 7.53 (d, *J* = 7.9 Hz, 1H), 7.31 (d, *J* = 8.0 Hz, 1H), 7.09 (s, 1H), 7.04 (t, *J* = 7.6 Hz, 1H), 7.01 – 6.87 (m, 4H), 6.65 (d, *J* = 8.2 Hz, 3H), 5.24 (s, 1H), 4.56 (dd, *J* = 12.6, 8.2 Hz, 1H), 4.40 (dd, *J* = 13.1, 6.2 Hz, 1H), 4.30 (dd, *J* = 13.4, 7.3 Hz, 1H), 3.62 – 3.57 (m, 2H), 3.55 – 3.52 (m, 1H), 2.93 – 2.88 (m, 2H), 2.77 (dd, *J* = 14.0, 7.9 Hz, 1H), 1.99 (dd, *J* = 12.7, 6.4 Hz, 1H), 1.09 (d, *J* = 5.1 Hz, 1H), 0.86 (dd, *J* = 12.1, 6.8 Hz, 6H).

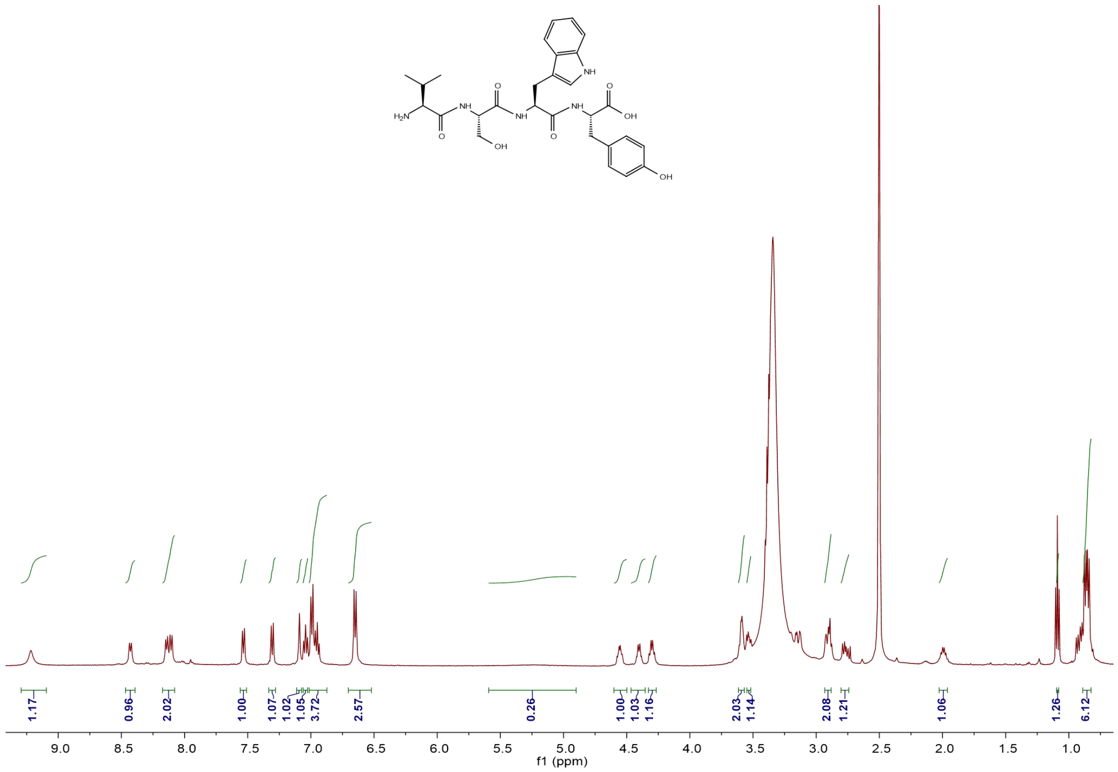

107.Compound **CAYW**: ^{1}H NMR (500 MHz, DMSO) δ 12.72 (s, 1H), 10.85 (s, 1H), 9.10 (d, *J* = 23.8 Hz, 1H), 8.55 (t, *J* = 8.3 Hz, 1H), 8.42 – 8.21 (m, 2H), 8.18 (s, 2H), 7.55 (d, *J* = 7.5 Hz, 1H), 7.31 (t, *J* = 10.1 Hz, 1H), 7.13 (dd, *J* = 15.8, 7.9 Hz, 1H), 7.07 (t, *J* = 7.4 Hz, 1H), 7.00 (t, *J* = 7.3 Hz, 1H), 6.87 – 6.68 (m, 2H), 6.56 – 6.37 (m, 2H), 4.54 – 4.41 (m, 2H), 4.32 (dd, *J* = 19.0, 12.0 Hz, 1H), 3.96 (s, 1H), 3.17 (dd, *J* = 14.5, 4.6 Hz, 2H), 3.01 – 2.88 (m, 2H), 2.66 (dd, *J* = 38.8, 29.2 Hz, 2H), 1.25 – 1.12 (m, 3H), 1.07 – 0.90 (m, 1H).

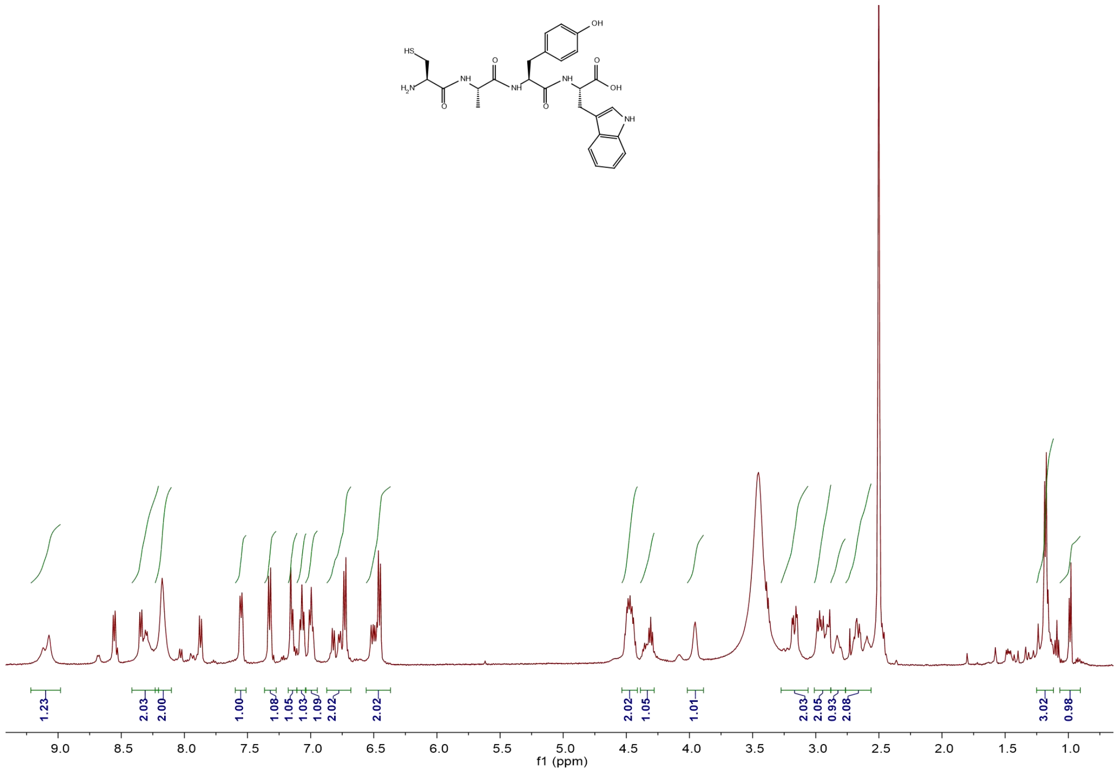

108.Compound **PWWY**: ^{1}H NMR (500 MHZ, DMSO-d6) δ 10.79 (d, J = 2.5 Hz, 2H), 9.21 (s, 1H), 8.59 (d, J = 8.2 Hz, 1H), 8.21 (d, J = 7.6 Hz, 1H), 8.16 (d, J = 8.0 Hz, 1H), 7.62 (d, J = 7.9 Hz, 1H), 7.58 Hz (d, J = 7.9 Hz, 1H), 7.31 (d, J = 8.1 Hz, 2H), 7.06 (m, 6H), 6.90 (td, J = 7.3, 4.9 Hz, 2H), 6.65 (d, J = 8.4 Hz, 1H), 4.61 (m, 2H), 4.39 (td, J = 7.7, 5.6 Hz, 1H), 3.99 (t, J = 7.1 Hz, 1H), 3.11 (m, 4H), 2.90 (m, 4H), 2.14 (dt, J = 13.9, 7.9 Hz, 1H), 1.71 (m, 4H).

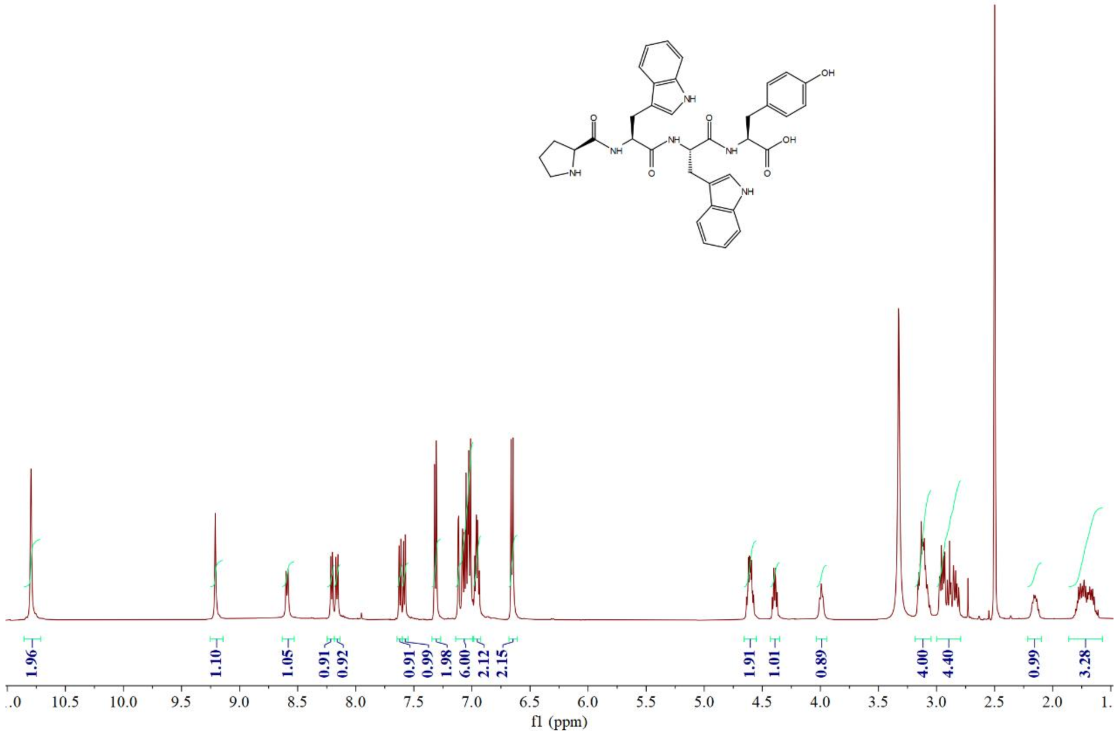

109.Compound **AAAA**: ^{1}H NMR (500 MHz, DMSO-d6) δ 8.53 (d, J = 7.5 Hz, 1H), 8.14 (d, J = 7.3 Hz, 1H), 8.04 (t, J = 7.1 Hz, 4H), 4.36 (p, J = 7.1 Hz, 1H), 4.29 (p, J = 7.1 Hz, 1H), 4.19 (p, J = 7.3 Hz, 1H), 1.33 (d, J = 6.9 Hz, 3H), 1.30 – 1.15 (m, 9H).

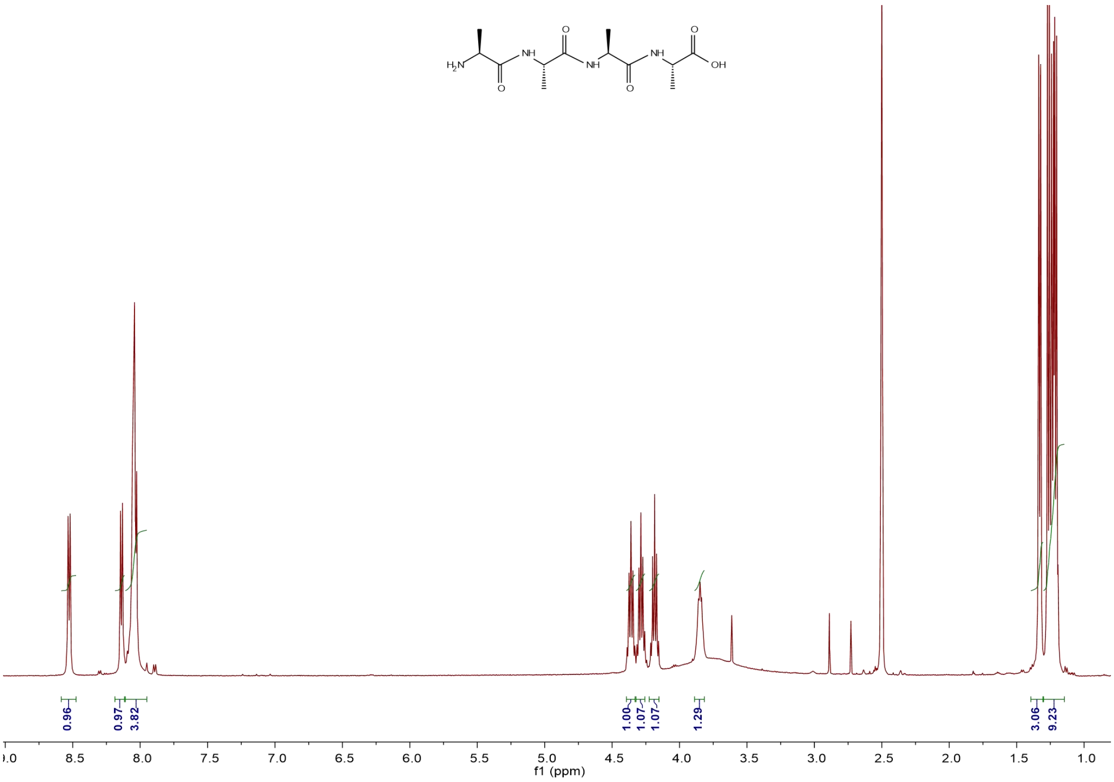

110.Compound **DDDD**: ^{1}H NMR (500 MHz, DMSO-d6) δ 8.76 (d, J = 7.5 Hz, 1H), 8.26 (d, J = 7.9 Hz, 1H), 8.19 – 8.12 (m, 3H), 8.00 (d, J = 8.1 Hz, 1H), 4.59 – 4.50 (m, 3H), 4.06 (d, J = 7.1 Hz, 1H), 2.92 – 2.88 (m, 1H), 2.78 (dd, J = 16.9, 4.2 Hz, 1H), 2.73 – 2.59 (m, 4H), 2.58 – 2.50 (m, 2H).

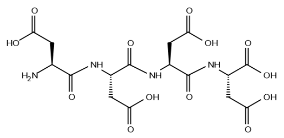

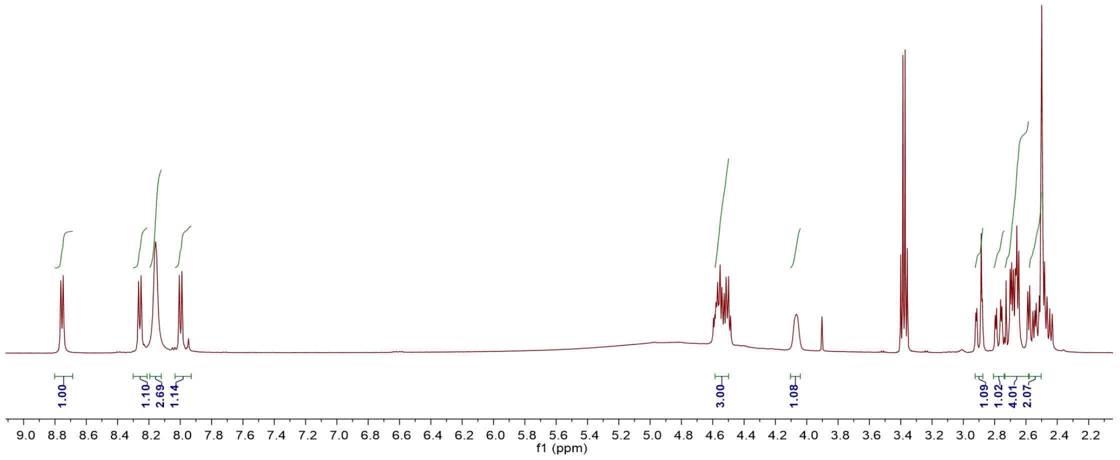

111.Compound **GGGG**: ^{1}H NMR (500 MHz, DMSO-d6) δ 8.62 (t, J = 5.7 Hz, 1H), 8.29 (t, J = 6.0 Hz, 1H), 8.19 (q, J = 5.4, 4.9 Hz, 1H), 8.00 (d, J = 7.4 Hz, 3H), 3.85 (d, J = 5.6 Hz, 2H), 3.76 (dd, J = 5.9, 1.7 Hz, 4H), 3.61 (q, J = 5.7 Hz, 2H).

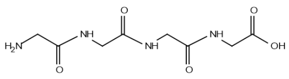

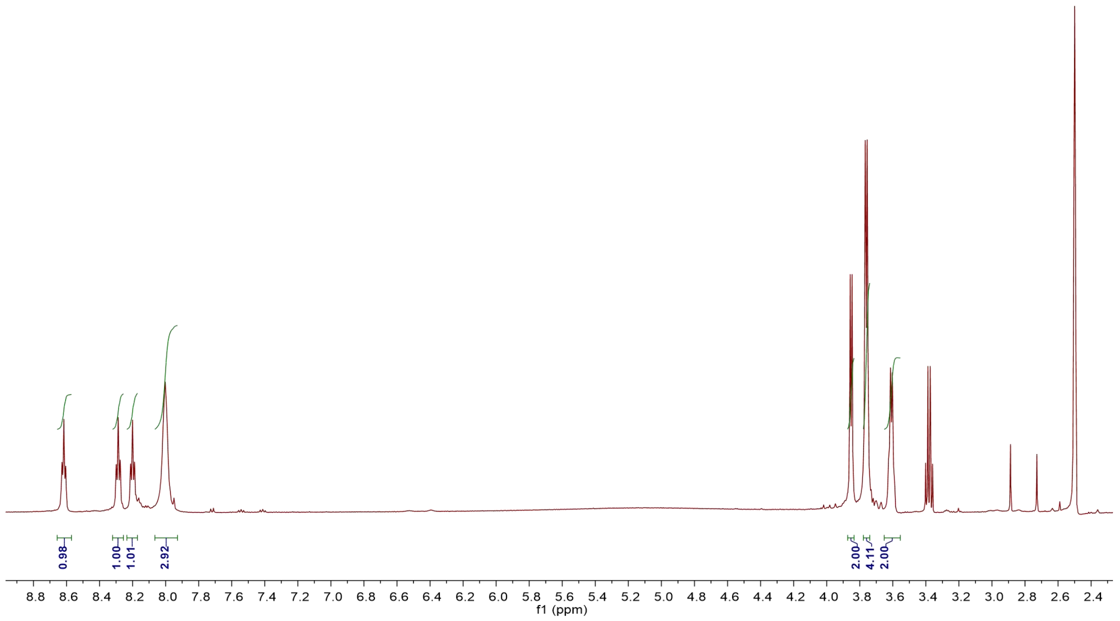

112.Compound **HHHH**: ^{1}H NMR (500 MHZ, DMSO-d6) δ 8.96 (m, 3H), 8.90 (d, J = 7.4 Hz, 1H), 8.73 (d, J = 7.7 Hz, 1H), 8.64 (d, J = 7.8 Hz, 1H), 7.36 (d, J = 4.7 Hz, 2H), 7.31 (d, J = 6.3 Hz, 2H), 4.62 (m, 3H), 4.19 (t, J = 6.5 Hz, 1H), 3.09 (m, 8H).

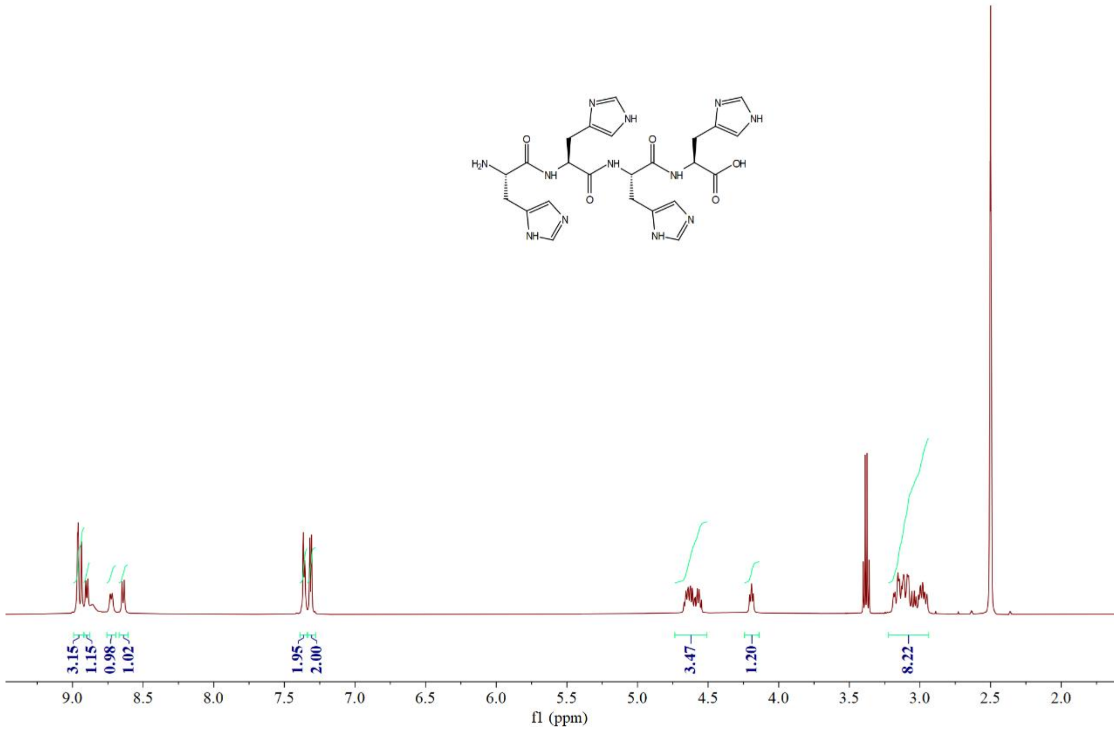

113.Compound **KKKK**: ^{1}H NMR (500 MHZ, DMSO-d6) δ 8.58 (d, J= 7.8 Hz, 1H), 8.18 (m, 4H), 8.10 (d, J = 7.9 Hz, 1H), 7.82 (s, 8H), 4.27 (m, 2H), 4.13 (td, J = 8.3, 5.3 Hz, 1H), 3.80 (td, J = 6.0 Hz, 1H), 3.80 (td, J = 6.0 Hz, 1H), 2.75 (dt, J = 11.8, 5.5 Hz, 8H), 1.53 (m, 16H), 1.34 (m, 8H).

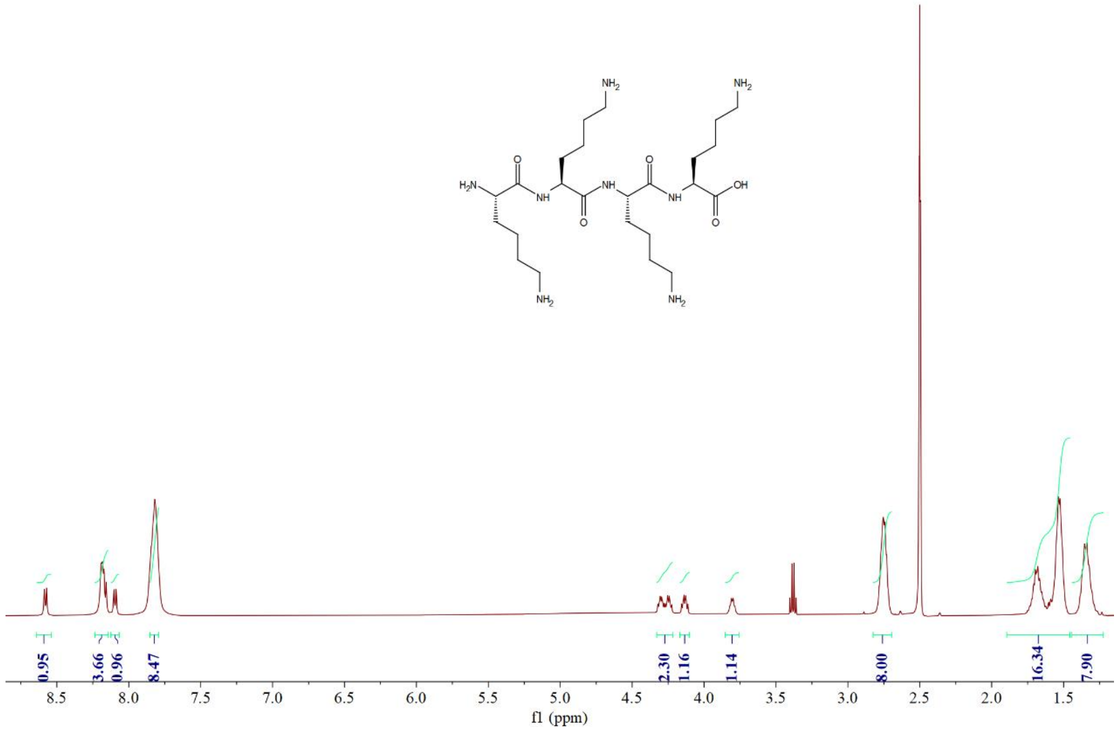

114.Compound **RRRR**: ^{1}H NMR (500 MHz, DMSO-d6) δ 8.54 (d, J = 7.4 Hz, 1H), 8.20 (d, J = 7.3 Hz, 1H), 8.12 (s, 3H), 7.58 (d, J = 5.7 Hz, 4H), 4.28 (dt, J = 32.5, 6.8 Hz, 2H), 4.13 (q, J = 7.5 Hz, 1H), 3.84 (s, 1H), 3.17 – 3.03 (m, 8H), 1.71 (s, 5H), 1.54 (q, J = 8.1, 7.6 Hz, 11H).

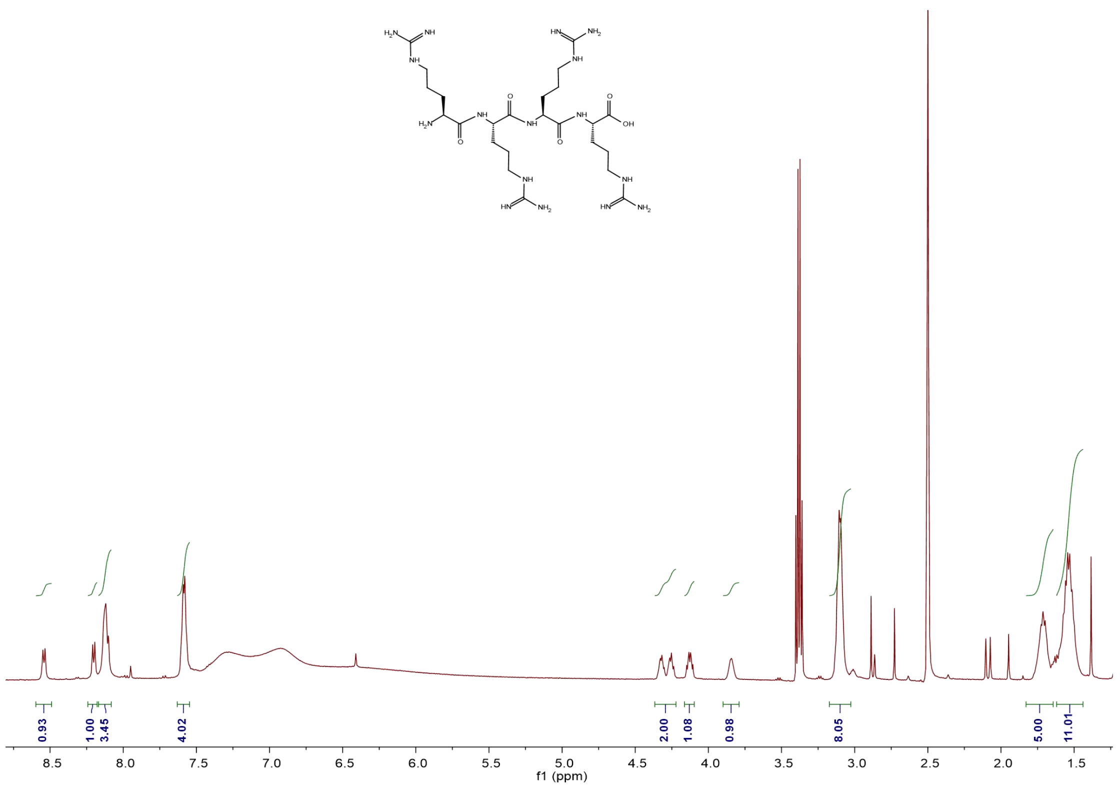

115.Compound **YYYY**: ^{1}H NMR (500 MHz, DMSO-d6) δ 9.16 (d, J = 33.8 Hz, 4H), 8.58 (d, J = 8.3 Hz, 1H), 8.19 (dd, J = 7.9, 4.0 Hz, 2H), 7.87 (d, J = 5.1 Hz, 3H), 7.10 – 6.96 (m, 8H), 6.72 – 6.58 (m, 8H), 4.51 (ddd, J = 10.3, 8.6, 5.4 Hz, 2H), 4.37 (td, J = 7.7, 5.7 Hz, 1H), 3.85 (dt, J = 9.3, 4.9 Hz, 1H), 2.99 – 2.87 (m, 4H), 2.83 – 2.61 (m, 4H).

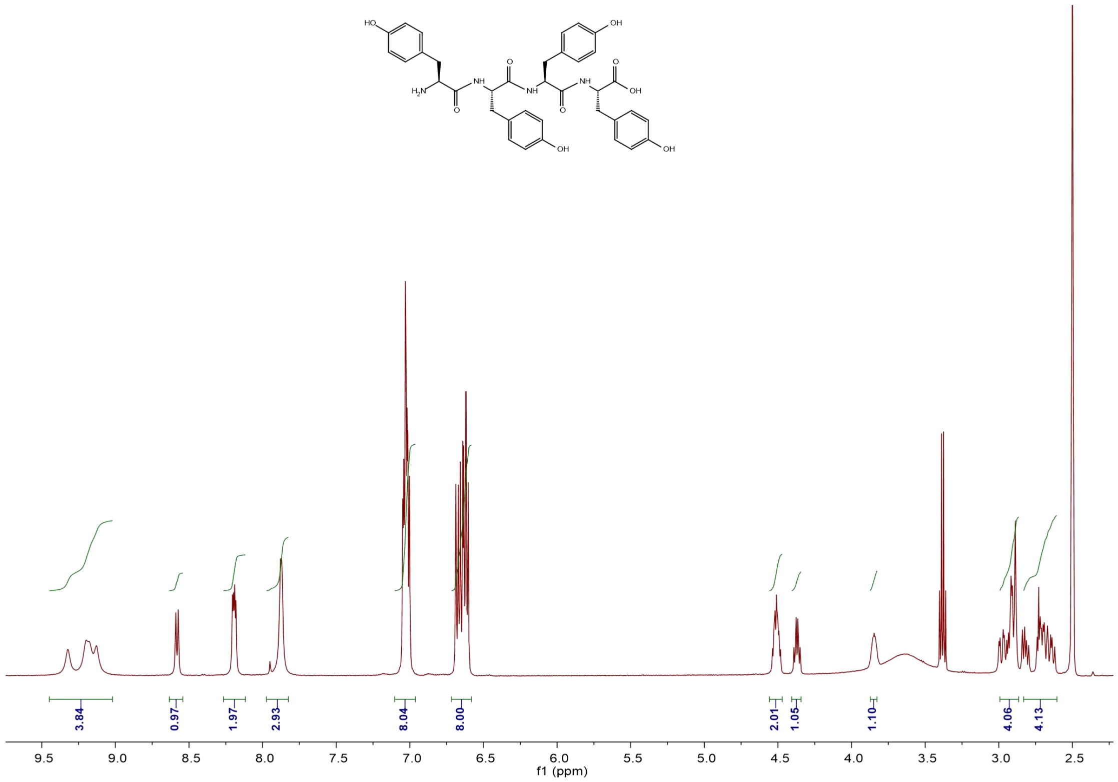

116.Compound **WFHP**: ^{1}H NMR (500 MHz, DMSO) δ 8.96 (s, 1H), 8.90 (d, *J* = 7.5 Hz, 1H), 8.73 (d, *J* = 8.2 Hz, 1H), 7.85 (d, *J* = 85.6 Hz, 3H), 7.71 (d, *J* = 7.9 Hz, 1H), 7.37 (dd, *J* = 19.6, 11.5 Hz, 2H), 7.30 – 7.20 (m, 6H), 7.10 (t, *J* = 7.5 Hz, 1H), 6.98 (t, *J* = 7.5 Hz, 1H), 4.89 (dd, *J* = 14.9, 7.4 Hz, 1H), 4.61 (dd, *J* = 13.6, 7.7 Hz, 1H), 4.22 (dd, *J* = 8.5, 3.4 Hz, 1H), 4.02 (d, *J* = 3.8 Hz, 1H), 3.22 (dd, *J* = 14.8, 3.8 Hz, 2H), 3.12 – 2.85 (m, 6H), 2.17 – 2.08 (m, 1H), 1.85 (dd, *J* = 10.7, 4.3 Hz, 3H).

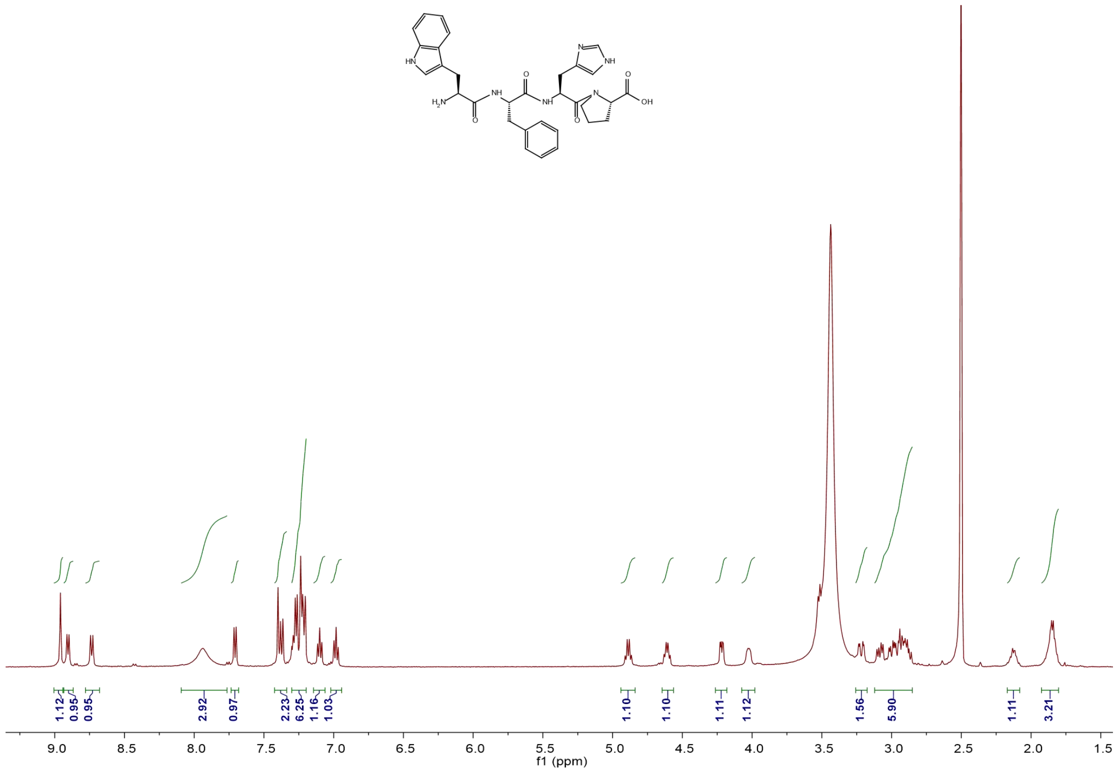

117.Compound **EFFK**: ^{1}H NMR (500 MHz, DMSO) δ 12.68 (s, 1H), 12.34 (s, 1H), 8.53 (d, *J* = 8.0 Hz, 1H), 8.39 (d, *J* = 8.1 Hz, 1H), 8.29 (d, *J* = 7.6 Hz, 1H), 8.05 (s, 2H), 7.73 (s, 2H), 7.25 (t, *J* = 6.5 Hz, 8H), 7.19 (dd, *J* = 11.9, 6.6 Hz, 2H), 4.64 – 4.57 (m, 2H), 4.18 (dd, *J* = 13.3, 8.0 Hz, 1H), 3.74 (s, 1H), 3.04 (td, *J* = 13.9, 3.8 Hz, 2H), 2.76 (dd, *J* = 23.0, 12.4 Hz, 4H), 2.34 – 2.27 (m, 2H), 1.95 – 1.86 (m, 2H), 1.74 (dt, *J* = 13.0, 6.6 Hz, 1H), 1.63 – 1.53 (m, 3H), 1.36 (dd, *J* = 14.9, 7.5 Hz, 2H).

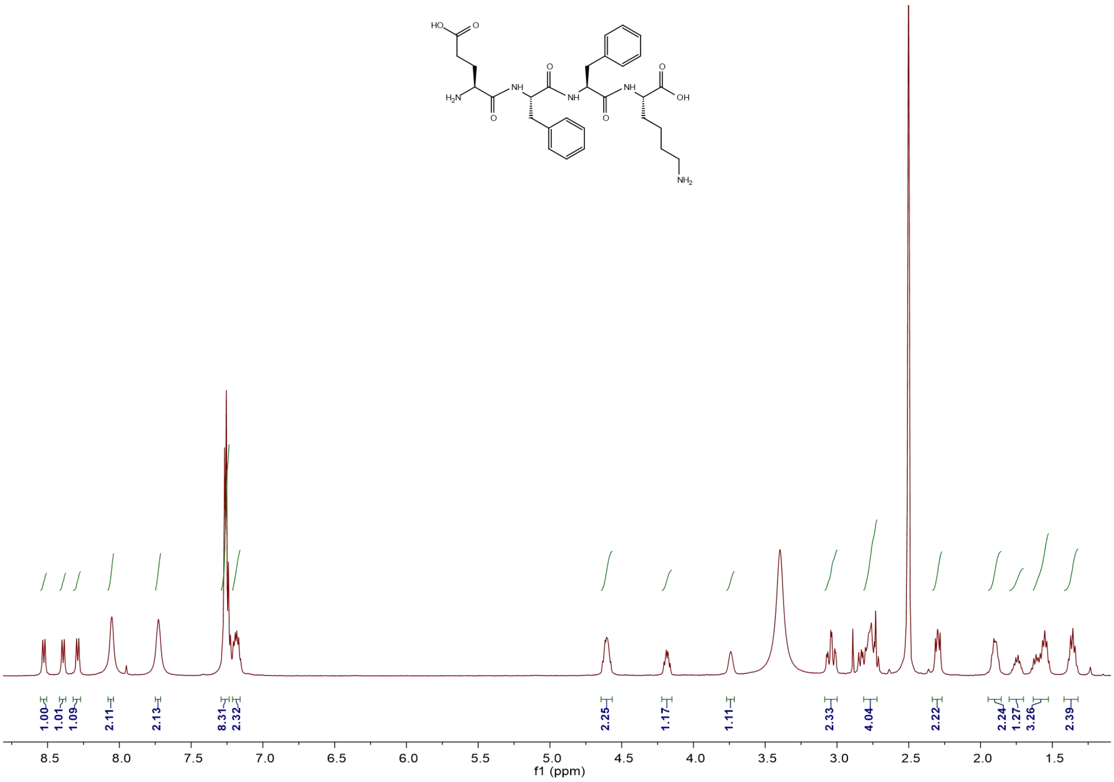

118.Compound **EEKK**: ^{1}H NMR (500 MHz, DMSO-d6) δ 8.59 (d, J = 7.6 Hz, 1H), 8.14 (dd, J = 17.5, 7.7 Hz, 2H), 4.26 (t, J = 7.1 Hz, 1H), 4.12 (q, J = 7.6 Hz, 1H), 3.81 (t, J = 6.4 Hz, 1H), 2.81 – 2.69 (m, 4H), 2.38 – 2.21 (m, 4H), 1.89 (h, J = 8.1 Hz, 4H), 1.73 – 1.46 (m, 8H), 1.35 (p, J = 7.6 Hz, 4H).

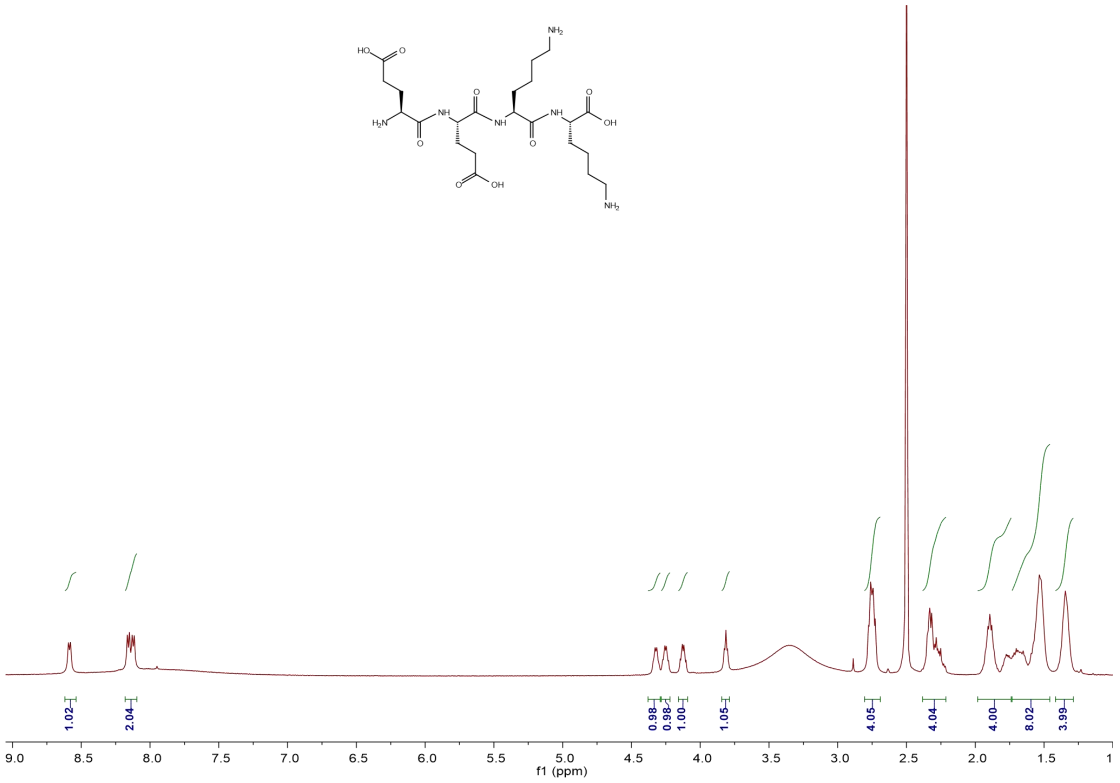

119.Compound **LGVF**: ^{1}H NMR (500 MHZ, DMSO-d6) δ 8.68 (t, *J* = 5.7 Hz, 1H), 8.32 (d, *J* = 7.7 Hz, 1H), 8.09 (d, *J* = 4.7 Hz, 3H, 7.90 (d, *J* = 9.1 Hz, 1H), 7.24 (m, 5H), 4.40 (m, 1H), 4.25 (dd, *J* = 9.1, 6.6 Hz, 1H), 3.91 (dd, *J* = 16.6, 5.8 Hz, 1H), 3.77 (dd, *J* = 16.6, 5.5 Hz, 1H), 3.04 (dd, *J* = 13.9, 5.2 Hz, 1H), 2.88 (dd, *J* = 13.9, 9.2 Hz, 1H), 1.94 (m, 1H), 1.68 (m, 1H), 1.55 (m, 2H), 0.89 (dd, *J* = 9.0, 6.5 Hz, 6H), 0.84 (d, *J* = 6.8 Hz, 3H), 0.79 (d, *J* = 6.8 Hz, 3H).

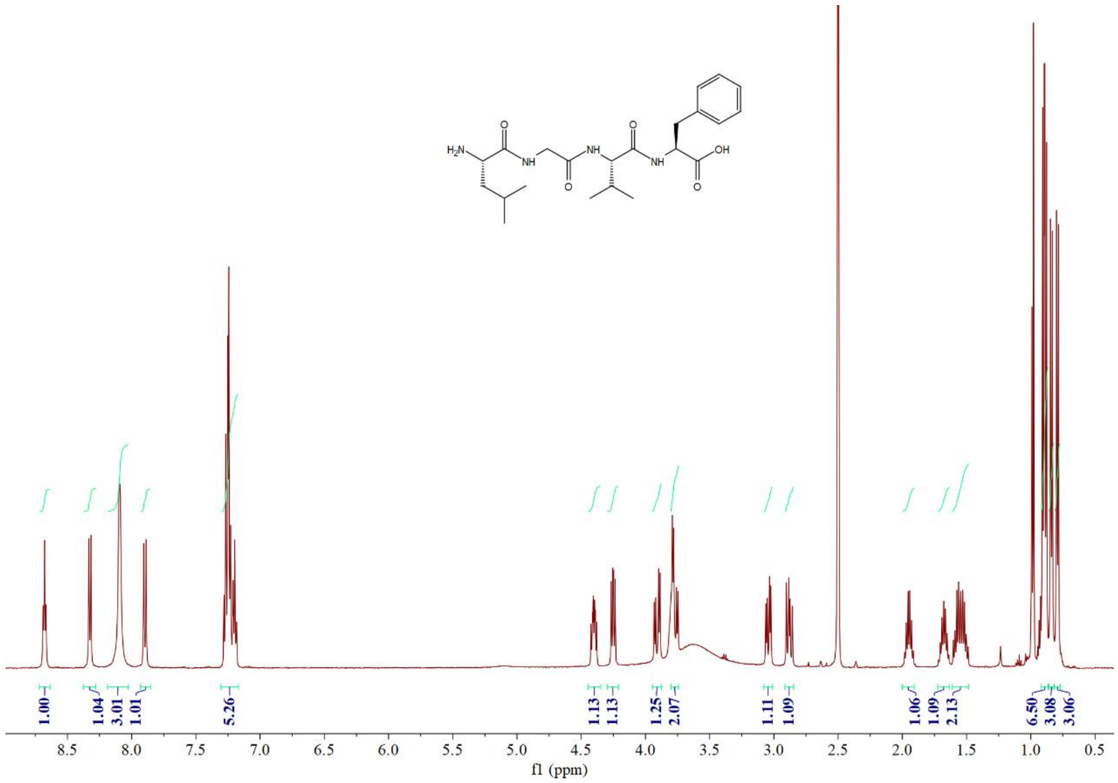

120.Compound **RWVF**: ^{1}H NMR (500 MHZ, DMSO-d6) δ 10.82 (d, *J* = 2.4 Hz, 1H), 8.57 (d, *J* = 7.7 Hz, 1H), 8.15 (dd, *J* = 15.6, 8.3 Hz, 2H), 7.65 (d, *J* = 7.9 Hz, 1H), 7.18 (m, 9H), 4.71 (td, *J* = 8.4, 4.8 Hz, 1H), 4.44 (td, *J* = 8.2, 5.3 Hz, 1H), 4.14 (s, 1H), 3.74 (t, *J* = 6.3 Hz, 1H), 3.06 (dt, *J* = 15.0, 5.6 Hz, 4H), 2.93 (td, *J* = 13.9, 9.0 Hz, 2H), 1.97 (m, 1H), 1.69 (m, 2H), 1.50 (m, 2H), 0.80 (t, *J* = 6.3 Hz, 6H).

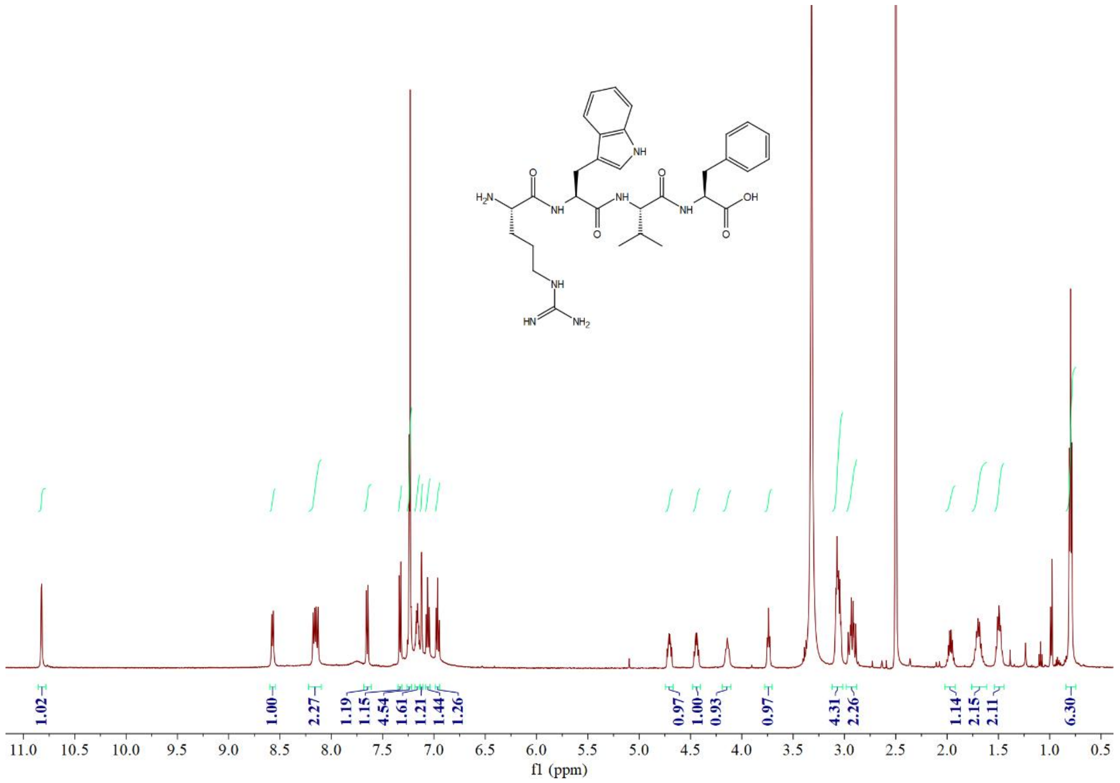

121.Compound **VALV**: ^{1}H NMR (500 MHz, DMSO) δ 8.55 (d, *J* = 7.5 Hz, 1H), 8.14 (d, *J* = 8.3 Hz, 1H), 8.06 (s, 2H), 7.82 (d, *J* = 8.5 Hz, 1H), 4.46 – 4.36 (m, 2H), 4.13 (dd, *J* = 8.2, 5.9 Hz, 1H), 3.59 (s, 1H), 2.09 – 1.99 (m, 2H), 1.62 (dq, *J* = 12.8, 6.6 Hz, 1H), 1.49 – 1.42 (m, 2H), 1.23 (d, *J* = 7.0 Hz, 3H), 0.96 – 0.83 (m, 18H).

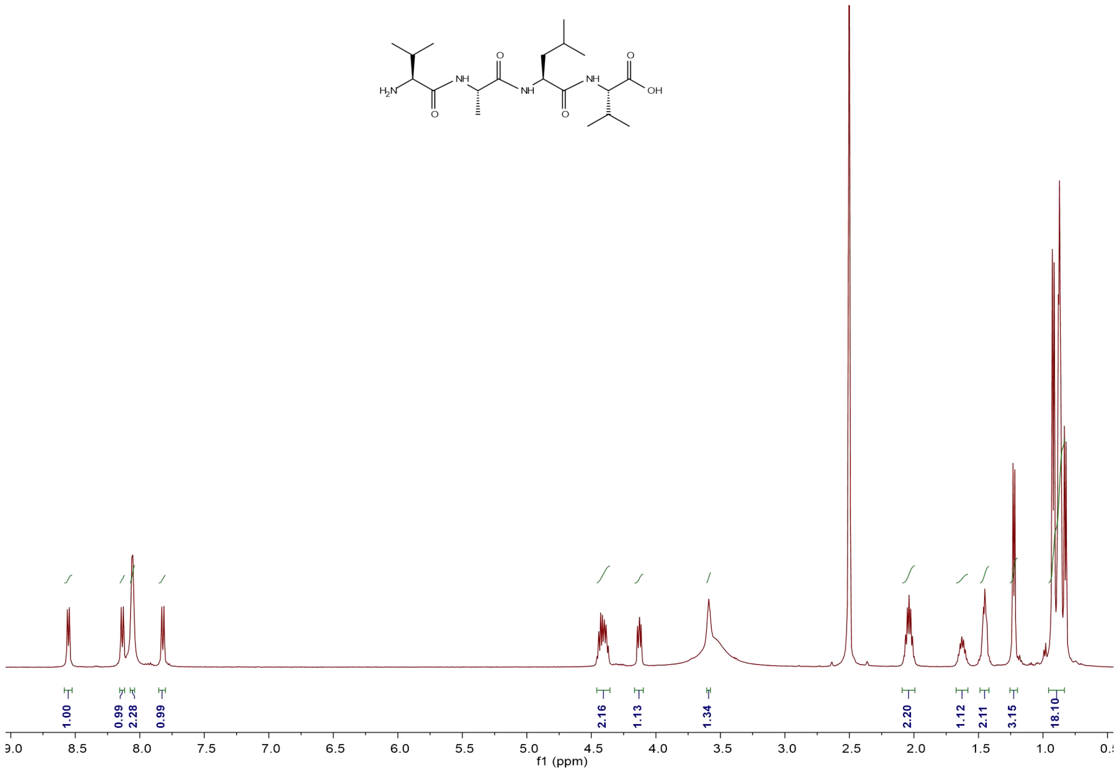

122.Compound **LRFH**: ^{1}H NMR (500 MHz, DMSO) δ 8.64 – 8.46 (m, 3H), 8.40 (d, *J* = 7.3 Hz, 1H), 8.17 (s, 1H), 7.23 (d, *J* = 11.0 Hz, 6H), 7.17 (s, 1H), 4.43 (d, *J* = 7.1 Hz, 2H), 4.35 (s, 1H), 3.78 (s, 1H), 3.09 (d, *J* = 4.7 Hz, 3H), 3.03 – 2.90 (m, 3H), 2.86 – 2.78 (m, 1H), 1.72 (s, 1H), 1.61 – 1.49 (m, 3H), 1.43 (t, *J* = 18.2 Hz, 3H), 0.83 (dd, *J* = 10.9, 6.4 Hz, 6H).

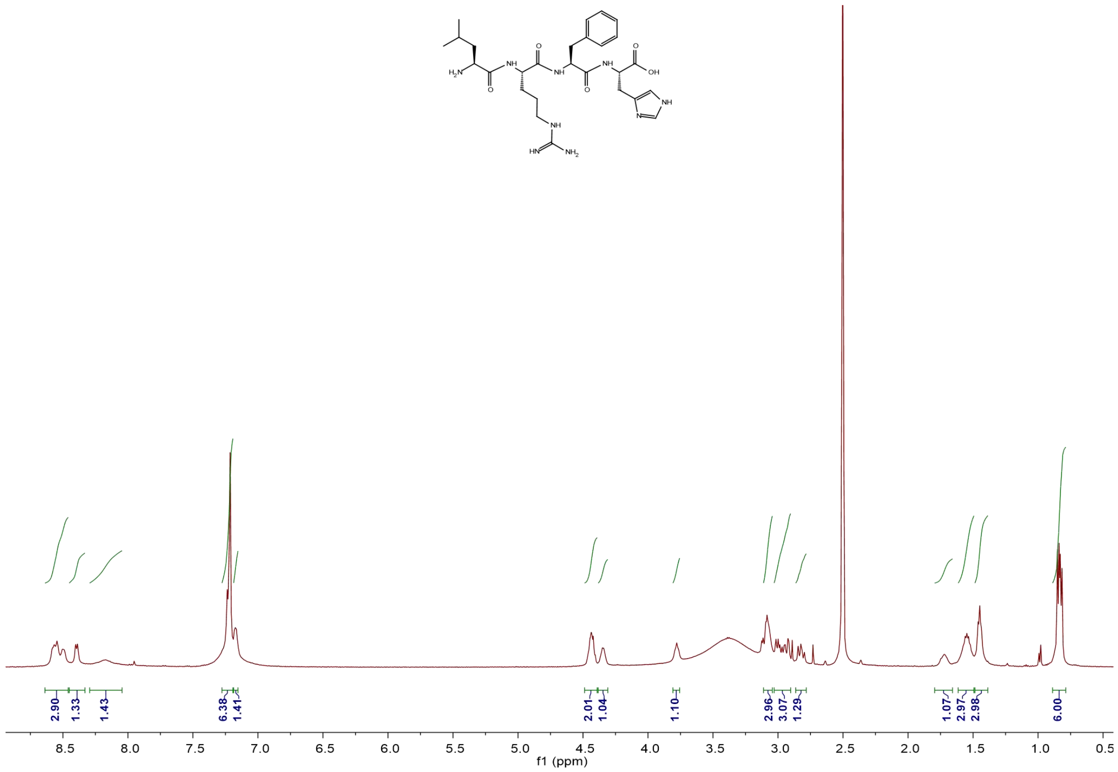

123.Compound **YAYY**: ^{1}H NMR (500 MHz, DMSO) δ 9.37 – 9.02 (m, 3H), 8.36 (d, *J* = 6.9 Hz, 1H), 8.04 (dd, *J* = 14.1, 7.9 Hz, 2H), 7.00 (dd, *J* = 11.3, 5.7 Hz, 6H), 6.71 – 6.55 (m, 6H), 4.42 (dd, *J* = 13.2, 8.5 Hz, 1H), 4.30 (dd, *J* = 13.7, 7.1 Hz, 2H), 3.69 (s, 1H), 3.38 (t, *J* = 5.9 Hz, 2H), 2.98 – 2.86 (m, 3H), 2.81 (dd, *J* = 13.9, 8.0 Hz, 1H), 2.70 – 2.58 (m, 2H), 1.09 (t, *J* = 7.0 Hz, 3H).

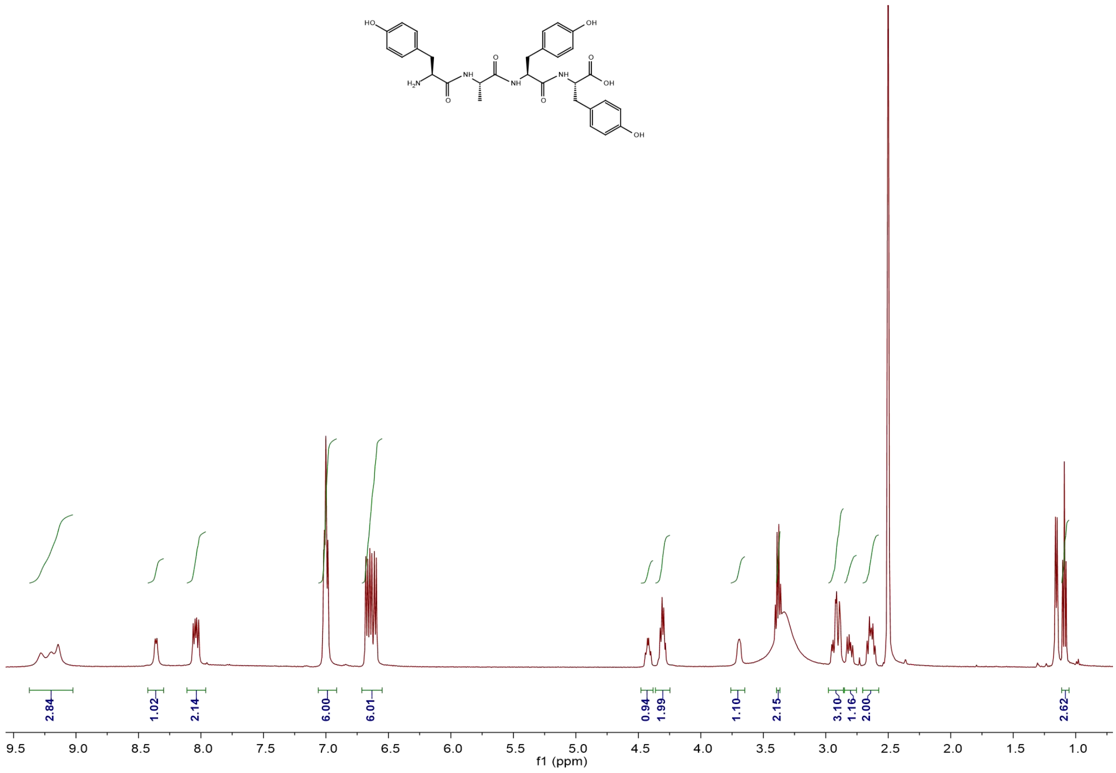

124.Compound **PYYI**: ^{1}H NMR (500 MHz, DMSO) δ 9.18 (d, *J* = 9.9 Hz, 2H), 8.55 (d, *J* = 8.5 Hz, 1H), 8.13 (dd, *J* = 36.7, 8.2 Hz, 2H), 7.03 (dd, *J* = 23.9, 8.1 Hz, 4H), 6.62 (d, *J* = 6.2 Hz, 4H), 4.52 (dtd, *J* = 36.6, 9.0, 4.0 Hz, 2H), 4.27 – 4.17 (m, 1H), 4.01 (t, *J* = 7.5 Hz, 1H), 3.19 – 3.07 (m, 2H), 2.95 – 2.87 (m, 2H), 2.74 – 2.56 (m, 2H), 2.27 – 2.16 (m, 1H), 1.87 – 1.65 (m, 4H), 1.49 – 1.37 (m, 1H), 1.20 (dt, *J* = 21.7, 7.7 Hz, 1H), 1.09 (t, *J* = 7.0 Hz, 1H), 0.87 (t, *J* = 7.8 Hz, 6H).

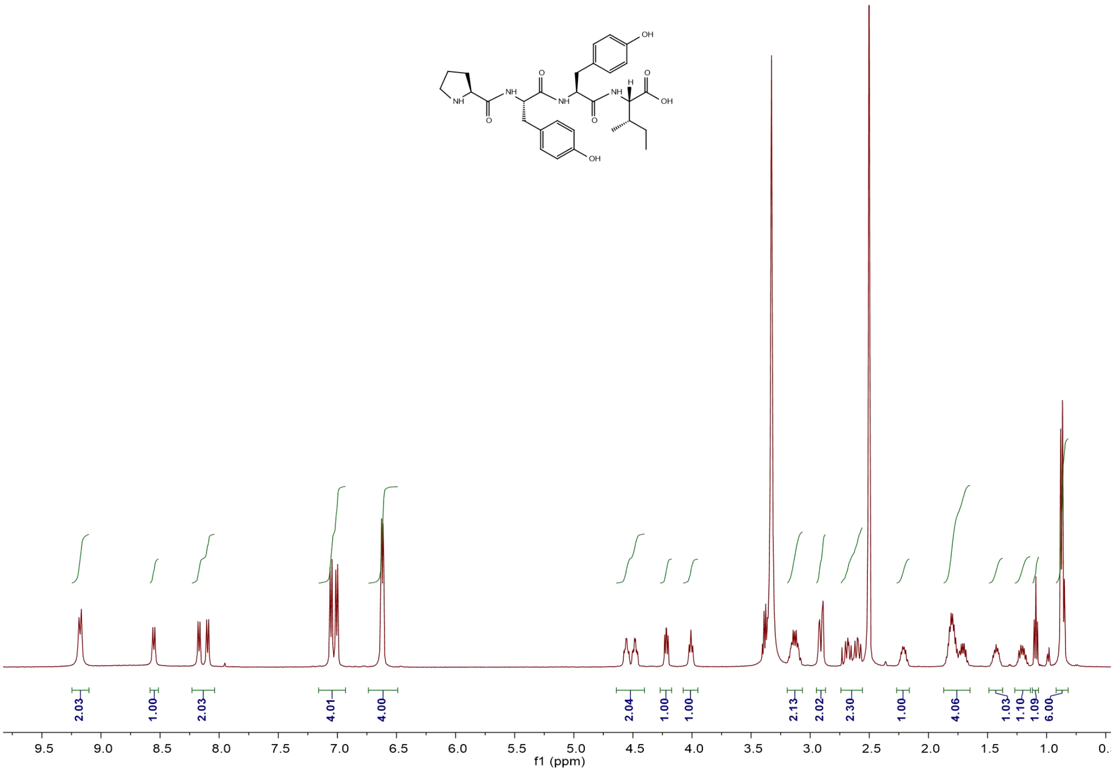

125.Compound **YPYI**: ^{1}H NMR (500 MHz, DMSO) δ 9.40 (d, *J* = 39.2 Hz, 1H), 9.17 (d, *J* = 16.5 Hz, 1H), 8.14 (dd, *J* = 35.1, 8.4 Hz, 1H), 8.00 (t, *J* = 19.0 Hz, 2H), 7.82 (d, *J* = 7.9 Hz, 1H), 7.14 (d, *J* = 8.1 Hz, 1H), 7.05 (t, *J* = 9.0 Hz, 2H), 6.92 (d, *J* = 8.1 Hz, 1H), 6.75 – 6.58 (m, 4H), 4.56 (dd, *J* = 12.6, 7.4 Hz, 1H), 4.47 – 4.36 (m, 1H), 4.19 (t, *J* = 6.9 Hz, 2H), 3.62 – 3.54 (m, 1H), 3.16 – 3.05 (m, 1H), 3.00 – 2.86 (m, 2H), 2.77 (ddd, *J* = 29.3, 21.0, 12.1 Hz, 2H), 2.01 (dd, *J* = 27.2, 23.0 Hz, 1H), 1.76 (dd, *J* = 15.8, 9.7 Hz, 3H), 1.69 – 1.51 (m, 1H), 1.43 (dd, *J* = 21.2, 14.4 Hz, 1H), 1.25 – 1.14 (m, 1H), 0.86 (t, *J* = 7.5 Hz, 6H).

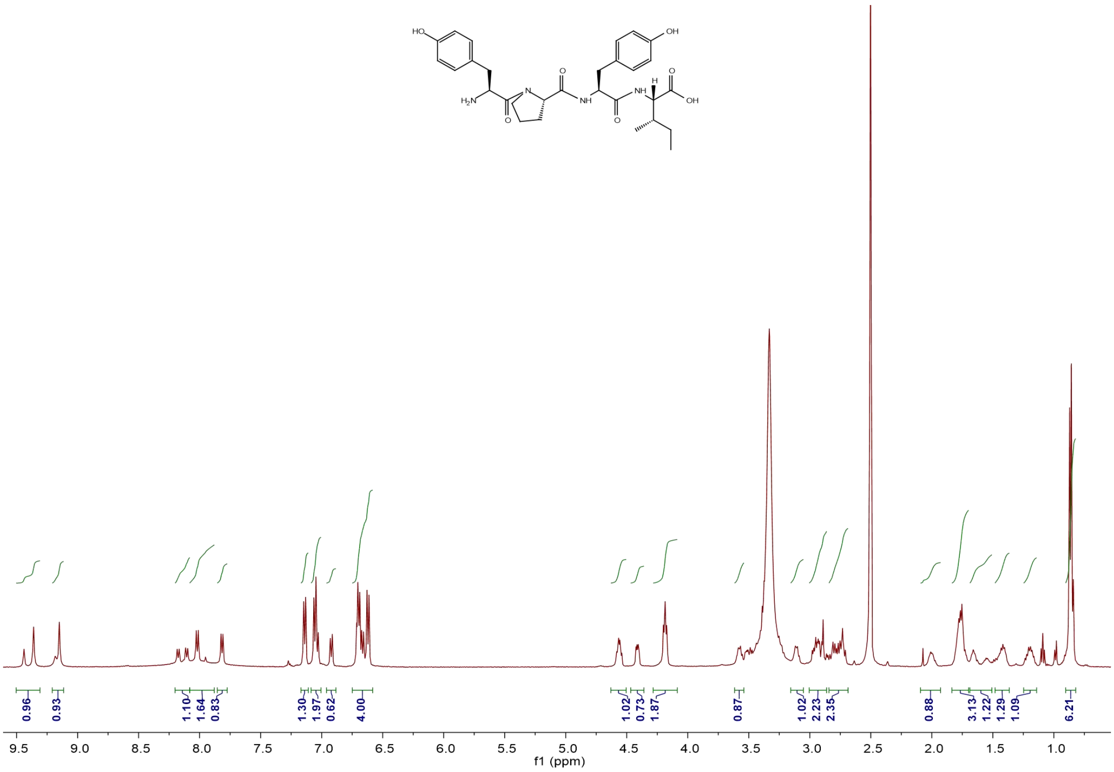

126.Compound **PGWW**: ^{1}H NMR (500 MHz, DMSO) δ 9.18 (s, 1H), 8.72 – 8.43 (m, 2H), 8.36 (dd, *J* = 16.1, 8.0 Hz, 1H), 8.11 (d, *J* = 8.1 Hz, 1H), 7.69 – 7.46 (m, 2H), 7.31 (dt, *J* = 25.6, 12.9 Hz, 2H), 7.21 – 6.88 (m, 6H), 4.76 – 4.43 (m, 2H), 4.15 (s, 1H), 3.88 – 3.63 (m, 2H), 3.26 – 3.00 (m, 5H), 2.90 (dd, *J* = 14.6, 9.2 Hz, 1H), 2.32 – 2.17 (m, 1H), 1.94 – 1.74 (m, 2H), 1.44 – 1.21 (m, 1H), 0.97 (t, *J* = 11.3 Hz, 1H).

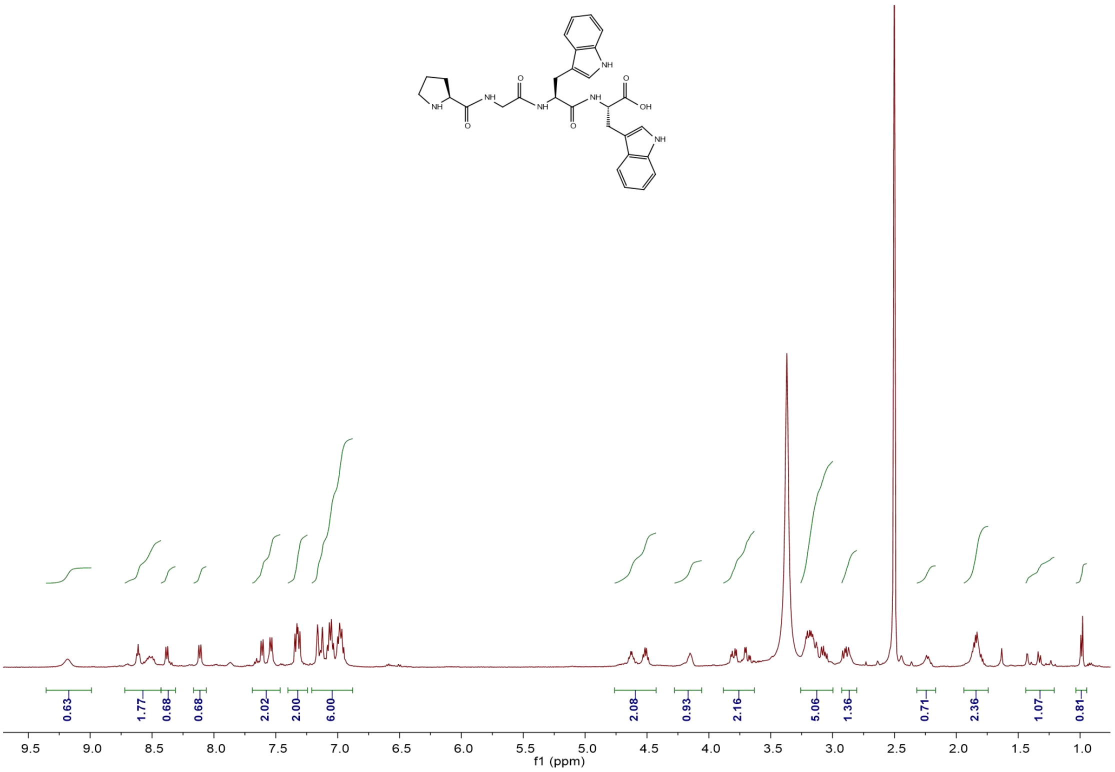

127.Compound **VYTF**: ^{1}H NMR (500 MHz, DMSO) δ 9.19 (s, 1H), 8.48 (d, *J* = 8.1 Hz, 1H), 8.12 (d, *J* = 8.5 Hz, 1H), 8.03 – 7.79 (m, 4H), 7.30 – 7.15 (m, 5H), 7.10 (d, *J* = 8.3 Hz, 2H), 6.64 (d, *J* = 8.3 Hz, 2H), 4.87 (s, 1H), 4.66 (td, *J* = 9.4, 4.0 Hz, 1H), 4.49 (dd, *J* = 13.3, 7.4 Hz, 1H), 4.00 – 3.93 (m, 1H), 3.61 – 3.55 (m, 1H), 3.05 (dd, *J* = 13.9, 5.5 Hz, 1H), 2.98 – 2.85 (m, 2H), 2.67 (dd, *J* = 14.1, 10.0 Hz, 1H), 2.11 (dq, *J* = 13.3, 6.8 Hz, 1H), 1.01 – 0.79 (m, 9H).

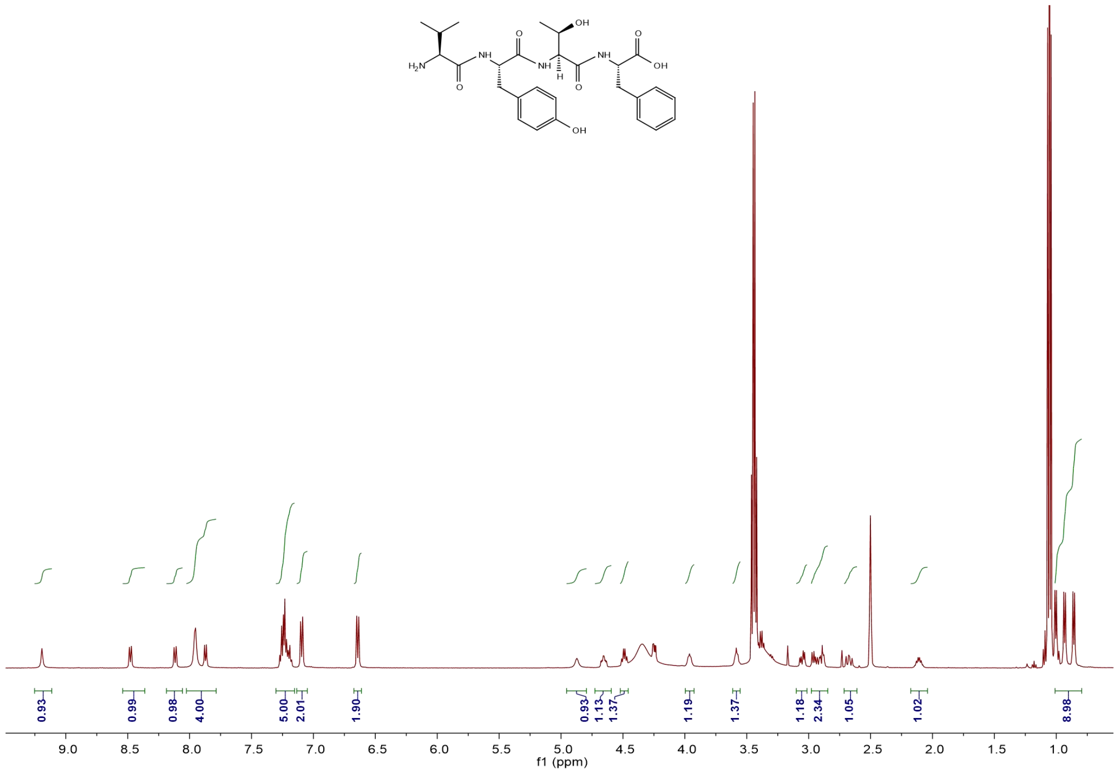

128.Compound **LHII**: ^{1}H NMR (600 MHz, DMSO-d6) δ 8.86 (d, J = 8.0 Hz, 1H), 8.16 (d, J = 8.2 Hz, 1H), 8.04 (d, J = 8.3 Hz, 1H), 7.27 (s, 1H), 4.76 (q, J = 7.1 Hz, 1H), 4.26 (t, J = 7.8 Hz, 1H), 4.16 (dd, J = 8.2, 6.1 Hz, 1H), 3.81 (t, J = 7.2 Hz, 1H), 3.04 (dd, J = 15.4, 6.3 Hz, 1H), 2.95 (dd, J = 15.3, 7.2 Hz, 1H), 1.82 – 1.68 (m, 2H), 1.62 (dp, J = 13.3, 6.6 Hz, 1H), 1.57 – 1.47 (m, 2H), 1.41 (dddd, J = 27.1, 11.7, 9.0, 5.6 Hz, 2H), 1.22 – 1.13 (m, 1H), 1.10 – 1.07 (m, 1H), 0.90 – 0.77 (m, 18H).

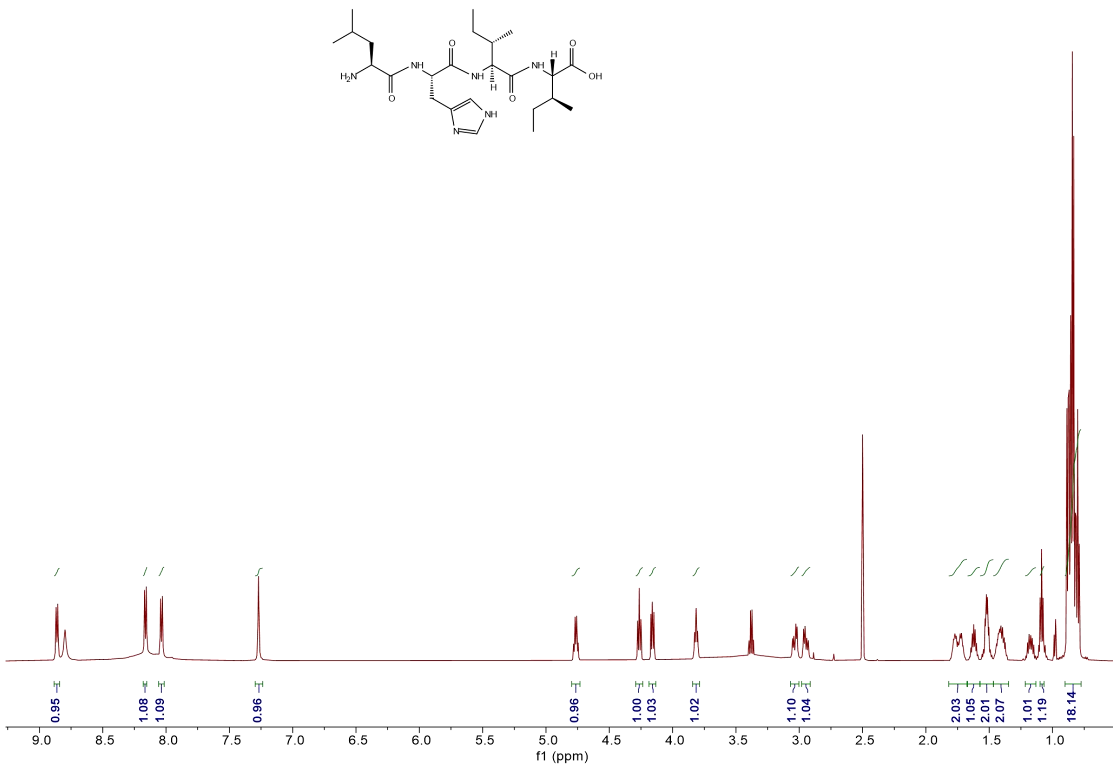

129.Compound **QHII**: ^{1}H NMR (500 MHz, DMSO) δ 8.91 (d, J = 10.6 Hz, 1H), 8.79 (d, J = 7.8 Hz, 1H), 8.23 (dt, J = 20.6, 10.3 Hz, 3H), 8.08 (d, J = 8.0 Hz, 1H), 7.40 (s, 1H), 7.30 (s, 1H), 6.97 (s, 1H), 4.75 (dd, J = 13.9, 7.0 Hz, 1H), 4.22 (dt, J = 44.9, 7.1 Hz, 2H), 3.86 (t, J = 6.0 Hz, 1H), 3.05 – 2.96 (m, 2H), 2.18 (dt, J = 15.8, 7.4 Hz, 2H), 1.97 – 1.85 (m, 2H), 1.75 (dd, J = 17.9, 5.5 Hz, 2H), 1.46 – 1.36 (m, 2H), 1.11 (ddd, J = 23.8, 16.5, 8.9 Hz, 2H), 0.83 (dt, J = 14.6, 7.7 Hz, 12H).

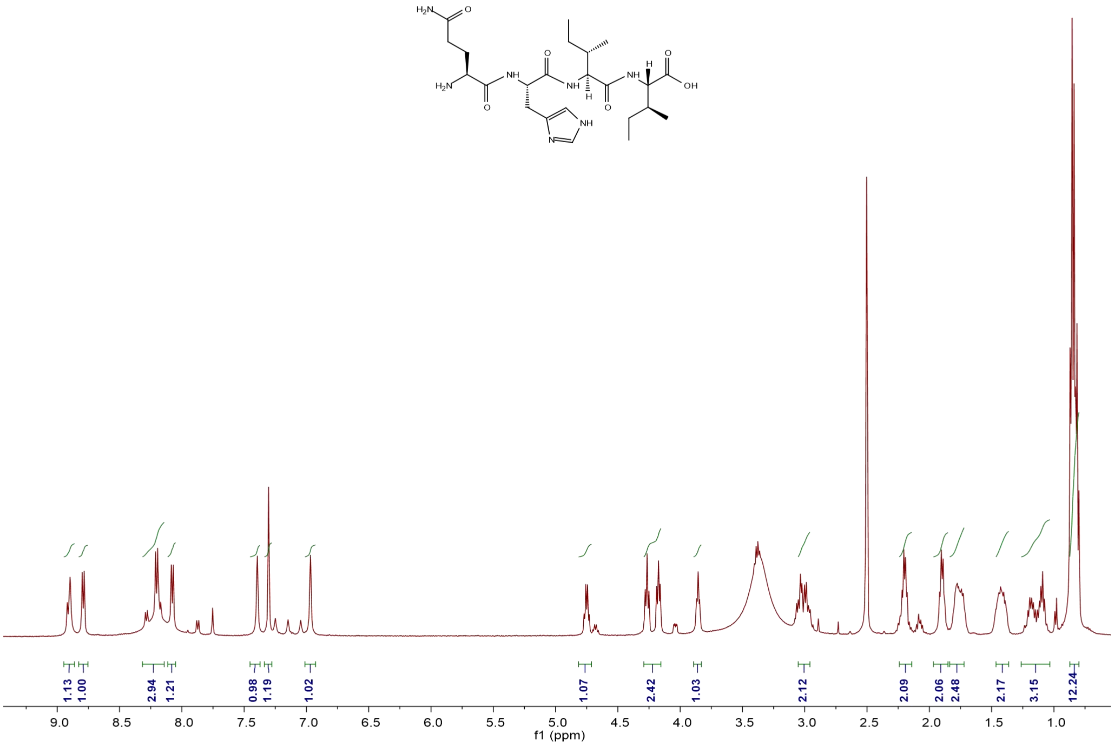

130.Compound **LTWF**: ^{1}H NMR (500 MHz, DMSO) δ 8.44 (d, J = 8.2 Hz, 1H), 8.25 (d, J = 7.6 Hz, 1H), 7.95 (d, J = 7.6 Hz, 1H), 7.54 (d, J = 7.8 Hz, 1H), 7.25 (ddd, J = 30.4, 17.5, 7.8 Hz, 6H), 7.09 (s, 1H), 7.04 (t, J = 7.5 Hz, 1H), 6.94 (t, J = 7.4 Hz, 1H), 4.57 (dd, J = 12.5, 7.8 Hz, 1H), 4.39 (dd, J = 13.3, 7.4 Hz, 1H), 4.29 (dt, J = 25.4, 12.7 Hz, 1H), 4.02 – 3.89 (m, 1H), 3.85 (t, J = 6.8 Hz, 1H), 3.13 (dd, J = 14.8, 4.4 Hz, 1H), 3.05 (dd, J = 13.7, 5.4 Hz, 1H), 2.90 (dd, J = 15.4, 6.4 Hz, 2H), 1.62 (dd, J = 13.0, 6.7 Hz, 1H), 1.55 – 1.43 (m, 2H), 1.00 (dd, J = 13.6, 6.6 Hz, 3H), 0.85 (dd, J = 11.6, 6.4 Hz, 6H).

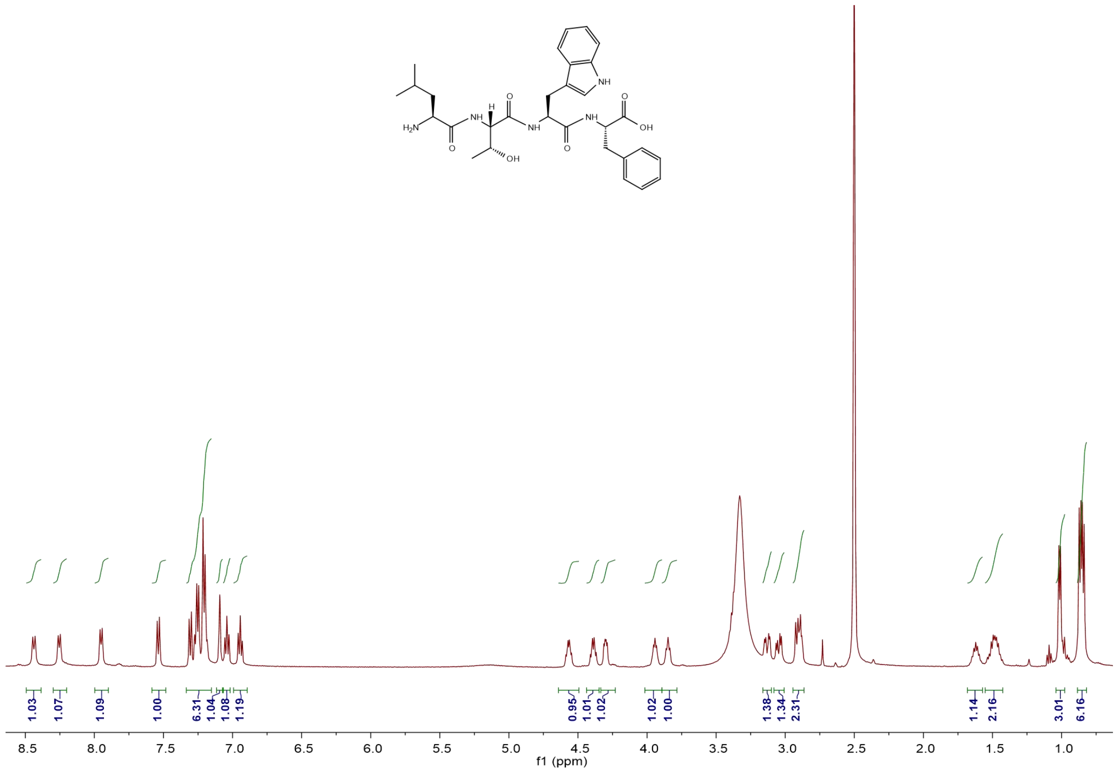

131.Compound **LWFI**: ^{1}H NMR (500 MHz, DMSO) δ 8.52 (d, J = 7.5 Hz, 1H), 8.24 (d, J = 8.0 Hz, 1H), 8.06 (d, J = 8.2 Hz, 1H), 7.60 (d, J = 7.9 Hz, 1H), 7.32 (d, J = 8.1 Hz, 1H), 7.23 (d, J = 4.1 Hz, 4H), 7.18 (d, J = 4.3 Hz, 1H), 7.11 (s, 1H), 7.05 (t, J = 7.5 Hz, 1H), 6.97 (t, J = 7.4 Hz, 1H), 4.71 – 4.52 (m, 2H), 4.27 – 4.13 (m, 1H), 3.60 (d, J = 5.7 Hz, 1H), 3.10 – 3.00 (m, 2H), 2.97 – 2.88 (m, 1H), 2.83 (dd, J = 13.9, 9.4 Hz, 1H), 1.77 (d, J = 31.0 Hz, 1H), 1.66 – 1.51 (m, 1H), 1.48 – 1.36 (m, 3H), 1.28 – 1.15 (m, 1H), 0.90 – 0.81 (m, 12H).

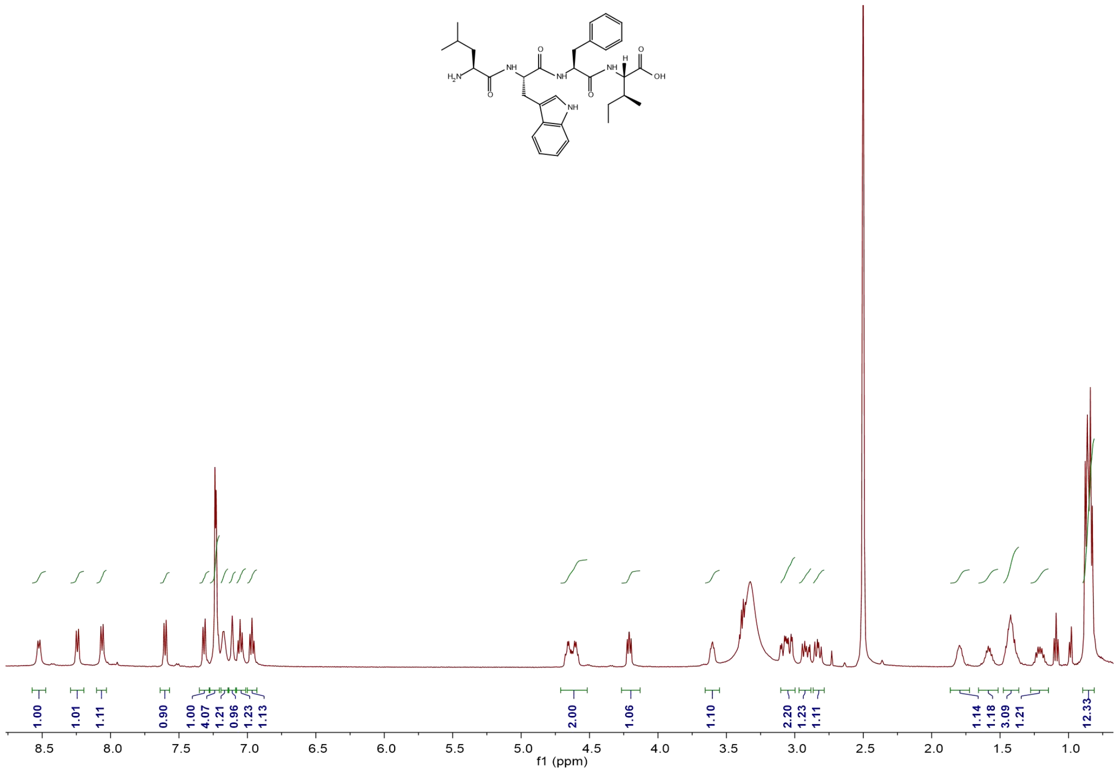

132.Compound **IHFV**: ^{1}H NMR (500 MHz, DMSO) δ 8.96 (s, 1H), 8.61 (d, *J* = 8.2 Hz, 1H), 8.38 (d, *J* = 8.7 Hz, 1H), 8.31 (d, *J* = 7.5 Hz, 1H), 8.02 (d, *J* = 61.3 Hz, 3H), 7.40 – 7.12 (m, 7H), 4.76 – 4.62 (m, 2H), 4.21 (dd, *J* = 8.3, 6.0 Hz, 1H), 3.64 (d, *J* = 5.0 Hz, 1H), 3.07 – 2.94 (m, 3H), 2.80 (dd, *J* = 14.0, 10.0 Hz, 1H), 2.14 – 2.02 (m, 1H), 1.72 (d, *J* = 6.1 Hz, 1H), 1.47 – 1.35 (m, 1H), 1.09 – 0.99 (m, 1H), 0.93 – 0.87 (m, 6H), 0.78 (dt, *J* = 15.6, 8.0 Hz, 6H).

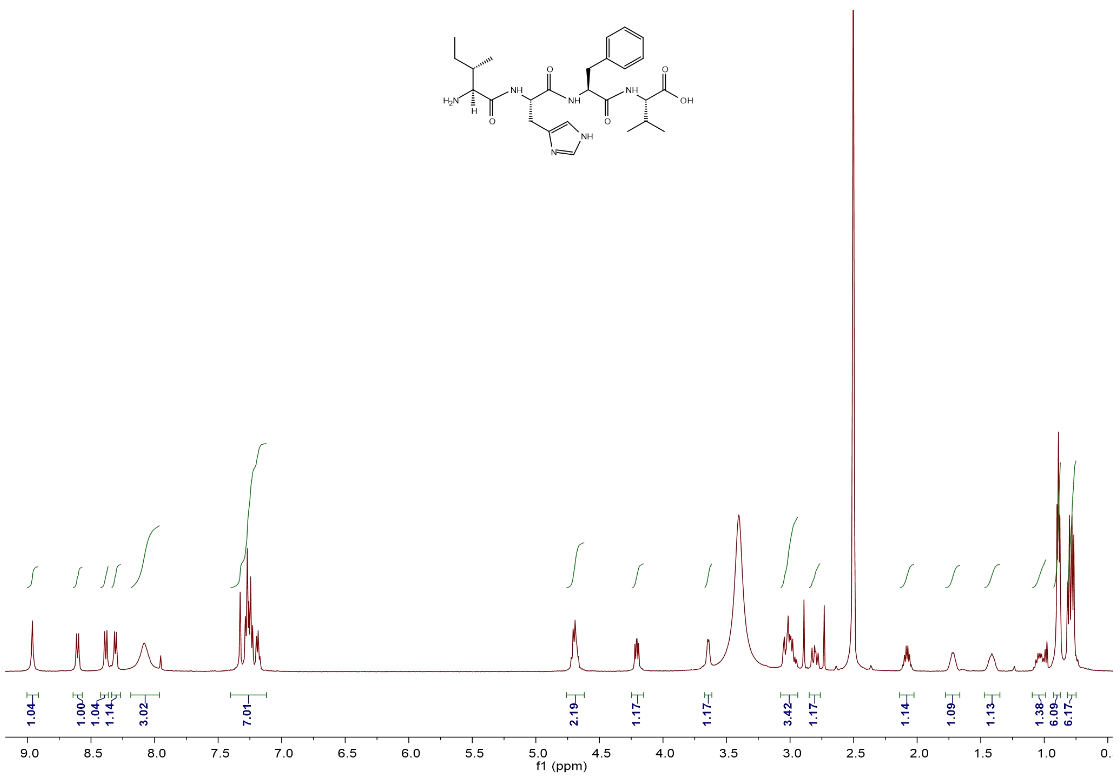

133.Compound **LVPF**: ^{1}H NMR (500 MHz, DMSO) δ 8.61 (d, *J* = 8.4 Hz, 1H), 8.10 – 8.01 (m, 3H), 7.33 – 7.15 (m, 5H), 4.40 – 4.33 (m, 2H), 3.85 (d, *J* = 5.2 Hz, 1H), 3.68 (dd, *J* = 15.8, 6.7 Hz, 1H), 3.61 – 3.55 (m, 1H), 3.42 – 3.32 (m, 1H), 3.01 (dd, *J* = 13.8, 5.5 Hz, 1H), 2.96 – 2.87 (m, 1H), 2.04 – 1.94 (m, 2H), 1.90 (dd, *J* = 13.5, 7.5 Hz, 1H), 1.82 (dd, *J* = 10.9, 5.2 Hz, 2H), 1.62 (ddt, *J* = 19.6, 13.0, 6.4 Hz, 1H), 1.50 (t, *J* = 6.9 Hz, 2H), 1.09 (t, *J* = 7.0 Hz, 1H), 0.90 (ddd, *J* = 11.1, 9.9, 5.2 Hz, 12H).

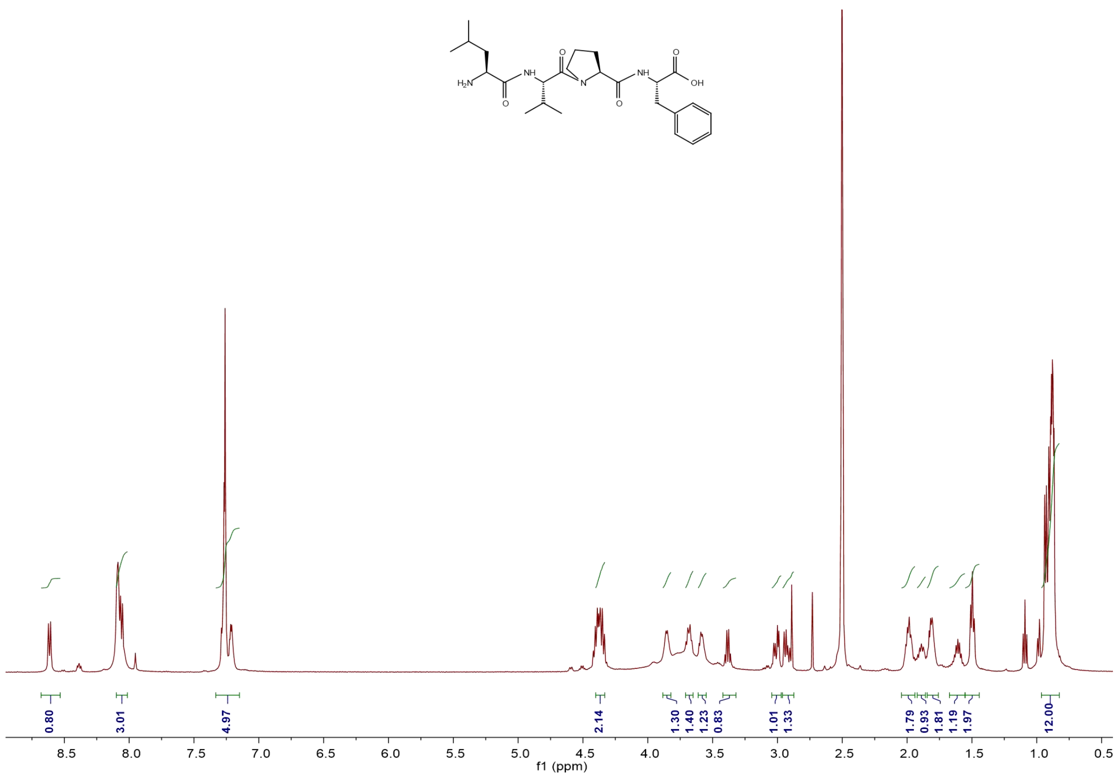

134.Compound **FMYS**: ^{1}H NMR (500 MHz, DMSO) δ 9.15 (s, 1H), 8.60 (t, J = 14.8 Hz, 1H), 8.25 (d, J = 7.8 Hz, 1H), 8.08 (d, J = 7.3 Hz, 3H), 7.20 (dd, J = 19.2, 4.2 Hz, 5H), 7.09 (d, J = 8.1 Hz, 2H), 6.63 (d, J = 8.1 Hz, 2H), 5.01 (s, 1H), 4.55 (td, J = 8.8, 3.9 Hz, 1H), 4.40 (dd, J = 13.2, 8.1 Hz, 1H), 4.32 – 4.23 (m, 1H), 4.05 (s, 1H), 3.77 – 3.67 (m, 2H), 3.63 (dd, J = 10.7, 3.9 Hz, 2H), 3.00 (ddd, J = 17.2, 13.9, 4.0 Hz, 2H), 2.87 (dd, J = 14.0, 8.0 Hz, 1H), 2.75 – 2.62 (m, 1H), 2.03 (s, 3H), 1.90 (dt, J = 15.0, 5.3 Hz, 1H), 1.74 (dt, J = 18.9, 11.5 Hz, 1H).

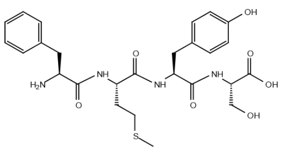

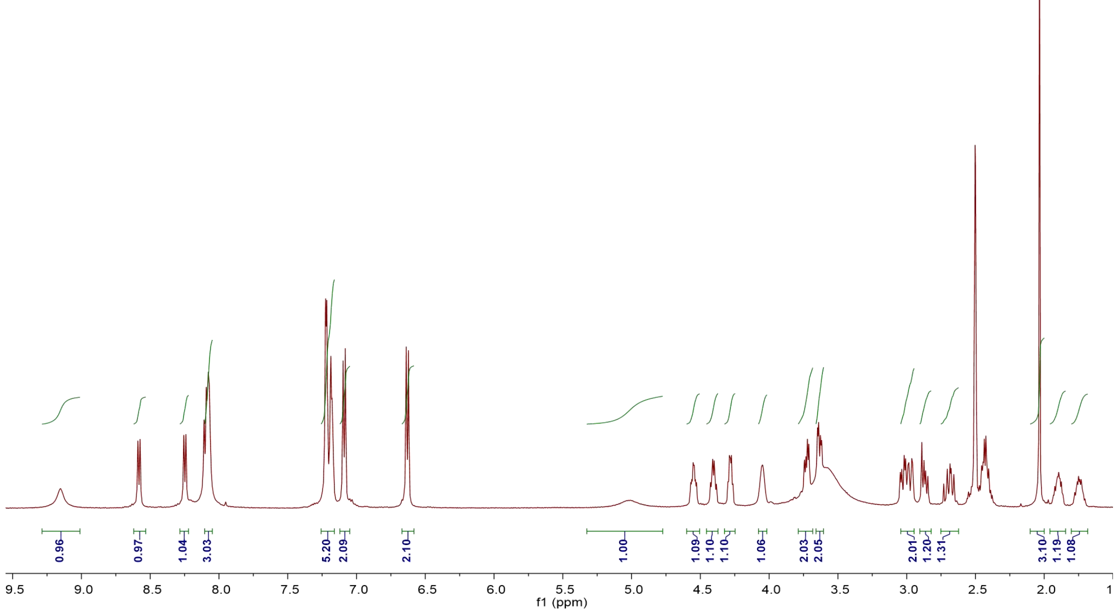

135.Compound **KFLL**: ^{1}H NMR (500 MHz, DMSO) δ 8.61 (d, *J* = 8.0 Hz, 1H), 8.15 (dd, *J* = 23.9, 8.4 Hz, 2H), 7.86 (s, 4H), 7.36 – 7.12 (m, 5H), 4.71 (dd, *J* = 12.8, 8.5 Hz, 1H), 4.22 (dd, *J* = 17.1, 10.0 Hz, 2H), 3.73 (t, *J* = 5.8 Hz, 1H), 3.38 (dd, *J* = 15.9, 8.9 Hz, 1H), 3.01 (dd, *J* = 14.0, 4.0 Hz, 1H), 2.79 (dd, *J* = 13.9, 9.6 Hz, 1H), 2.76 – 2.68 (m, 2H), 1.97 (dt, *J* = 13.3, 6.7 Hz, 1H), 1.74 – 1.60 (m, 3H), 1.60 – 1.43 (m, 4H), 1.31 (s, 2H), 1.13 – 0.96 (m, 1H), 1.00 – 0.73 (m, 12H).

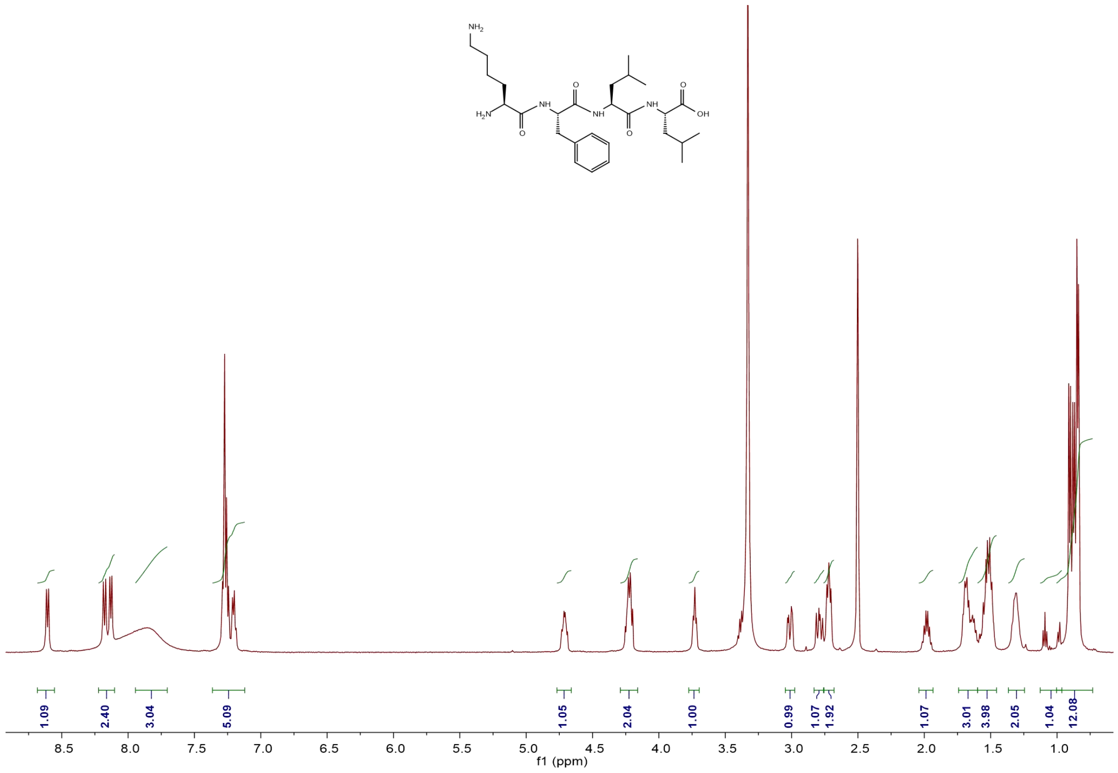

136.Compound **VFGV**: ^{1}H NMR (500 MHZ, DMSO-d6) δ 8.57 (d, *J* = 8.0 Hz, 1H), 8.39 (t, *J* = 5.7 Hz, 1H), 7.96 (m, 4H), 7.27 (m, 4H), 7.21 (m, 1H), 4.65 (td, *J* = 8.7, 4.6 Hz, 1H), 4.18 (dd, *J* = 8.6, 5.7 Hz, 1H), 3.79 (m, 2H), 3.60 (t, *J* = 5.3 Hz, 2H), 3.04 (dd, *J* = 14.1, 4.6 Hz, 1H), 2.84 (m, 1H), 2.08 (m, 2H), 0.87 (dd, *J* = 6.8, 3.8 Hz, 12H).

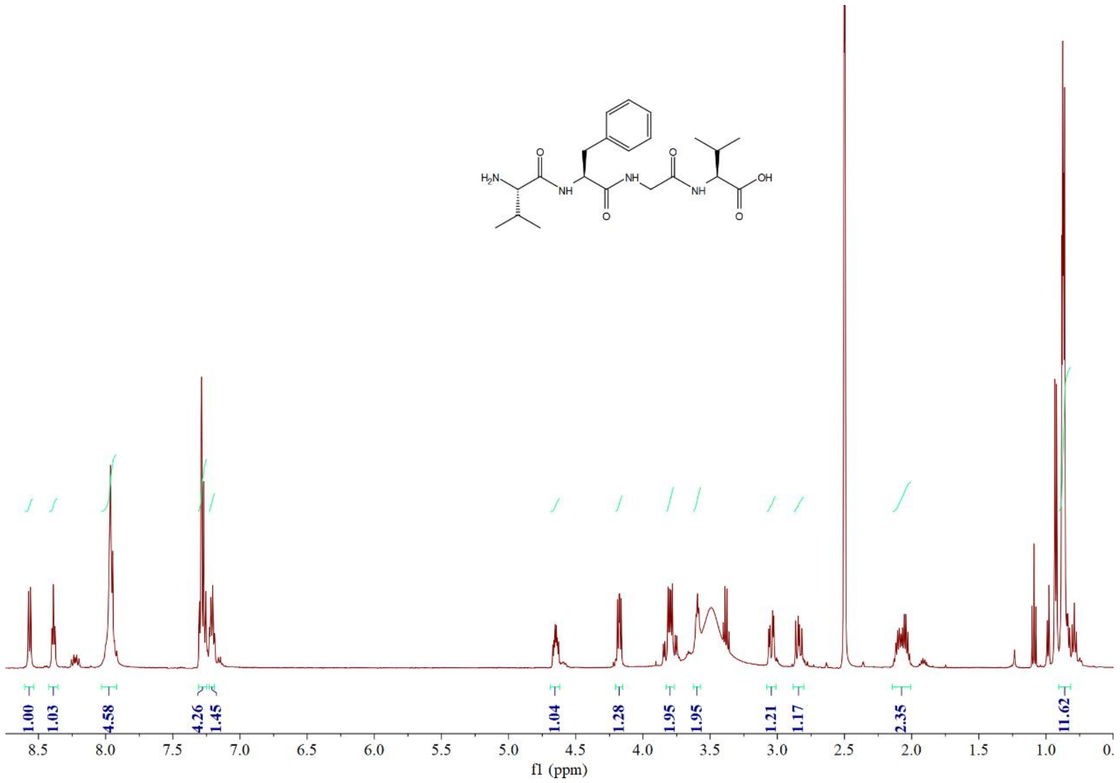

137.Compound **CCCC**: ^{1}H NMR (500 MHz, DMSO-d6) δ 8.75 (d, J = 7.7 Hz, 1H), 8.40 (d, J = 7.8 Hz, 1H), 8.34 (d, J = 7.8 Hz, 1H), 8.24 (s, 2H), 7.31 (t, J = 7.6 Hz, 1H), 7.12 (dd, J = 7.4, 1.7 Hz, 1H), 4.53 (td, J = 7.6, 4.9 Hz, 1H), 4.48 (td, J = 7.7, 4.9 Hz, 1H), 4.41 (td, J = 7.4, 4.4 Hz, 1H), 4.08 (t, J = 5.5 Hz, 1H), 2.93 – 2.84 (m, 4H), 2.80 – 2.70 (m, 4H).

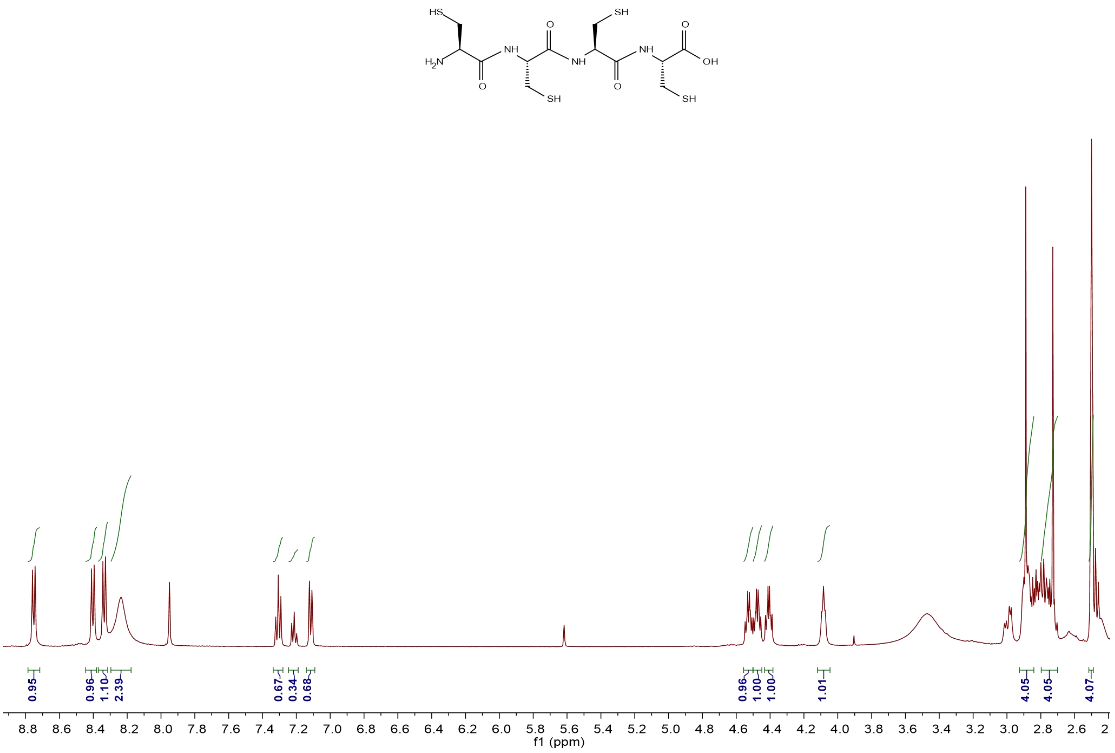

138.Compound **EEEE**: ^{1}H NMR (500 MHZ, DMSO-d6) δ 8.60 (d, J = 7.5 Hz, 1H), 8.23 (d, J = 7.5 Hz, 1H), 8.19 (d, J = 7.6 Hz, 1H), 8.12 (d, J = 5.2 Hz, 3H), 4.34 (td, J = 8.0, 5.3 Hz, 1H), 4.27 (td, J = 7.9, 5.5 Hz, 1H), 4.18 (td, J = 7.5, 5.2 Hz), 3.83 (m, 1H), 2.29 (m, 8H), 1.87 (m, 8H).

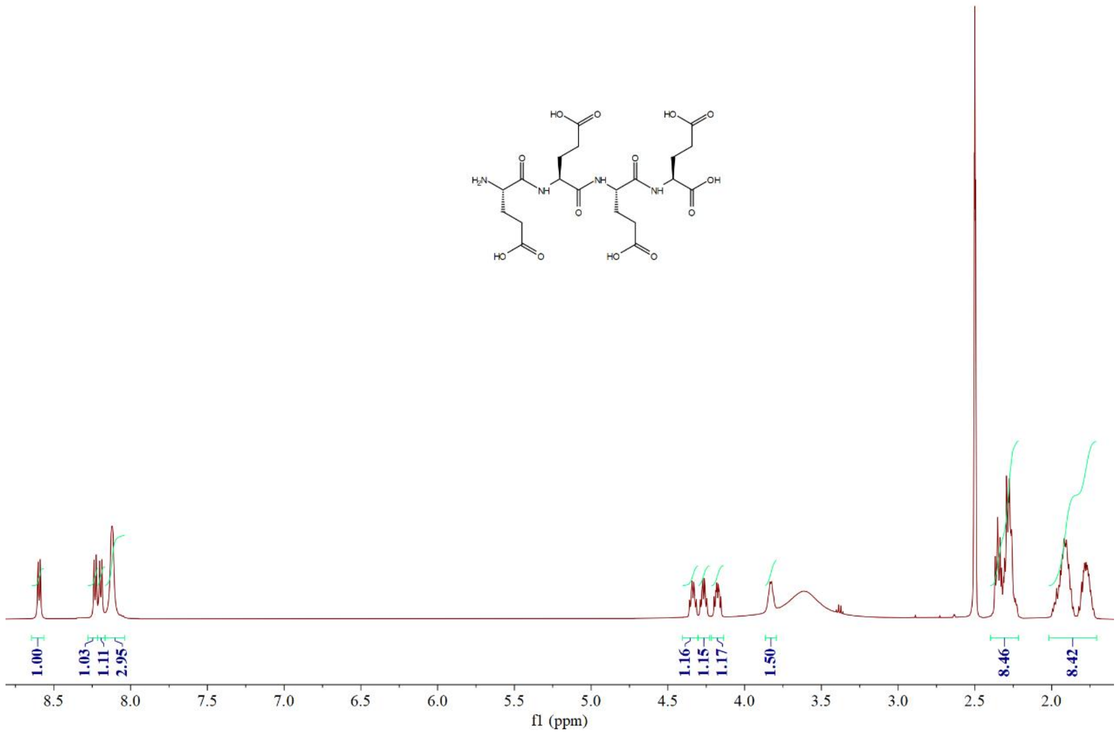

139.Compound **PPPP**: ^{1}H NMR (500 MHz, DMSO-d6) δ 4.66 (dd, J = 8.6, 4.6 Hz, 1H), 4.61 – 4.54 (m, 1H), 4.49 (t, J = 7.0 Hz, 1H), 4.20 (dt, J = 8.7, 5.3 Hz, 1H), 3.71 – 3.62 (m, 1H), 3.61 – 3.46 (m, 2H), 3.24 – 3.14 (m, 1H), 2.28 – 2.10 (m, 2H), 1.98 – 1.77 (m, 7H).

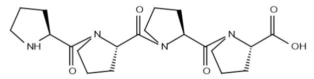

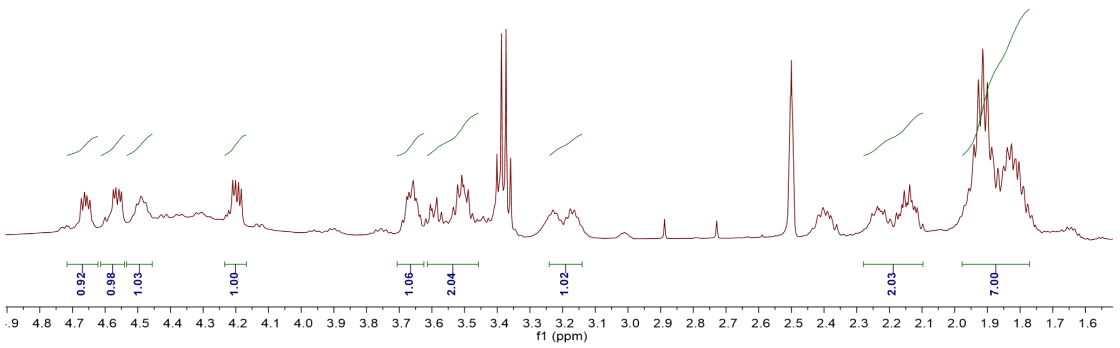

140.Compound **QQQQ**: ^{1}H NMR (500 MHZ, DMSO-d6) δ 8.63 (d, J = 7.6 Hz, 1H), 8.27 (d, J = 7.6 Hz, 1H), 8.18 (d, J = 7.8 Hz, 1H), 8.15 (d, J = 5.4 Hz, 3H), 7.43 (d, J = 2.4 Hz, 1H), 7.25 (m, 6H), 6.99 (m, 1H), 6.82 (m, 1H), 6.79 (m, 2H), 4.29 (m, 2H), 4.16 (m, 1H), 3.83 (m, 1H), 2.06 (m, 16H).

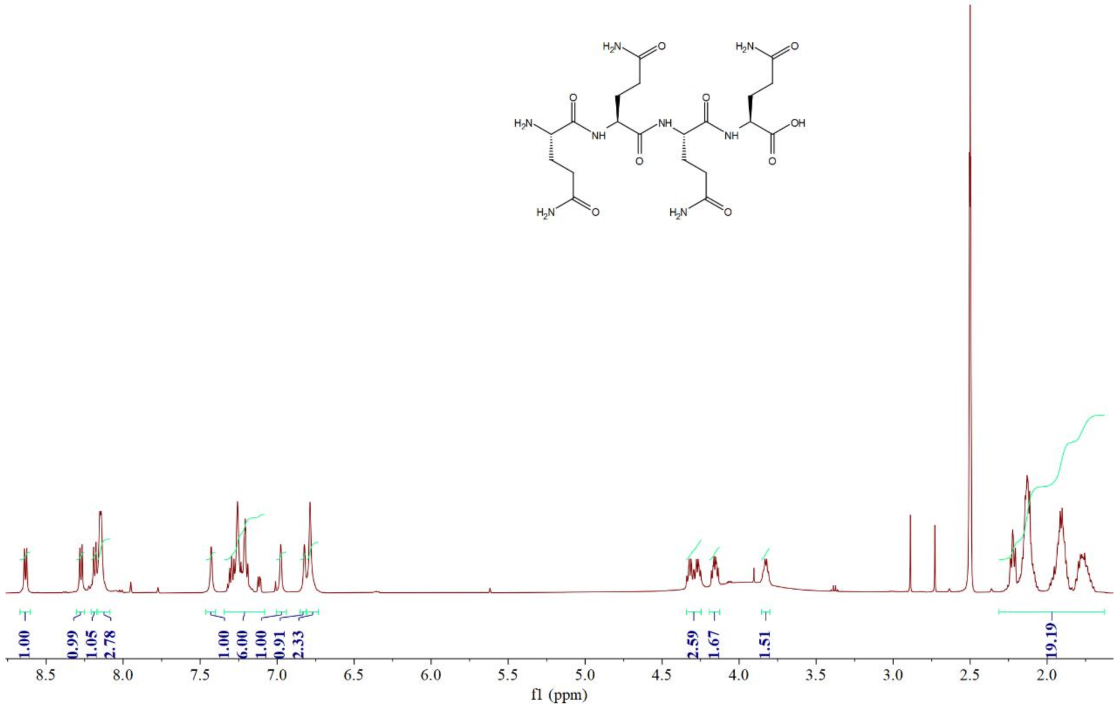

141.Compound **SSSS**: ^{1}H NMR (500 MHZ, DMSO-d6) δ 8.62 (d, J = 7.8 Hz, 1H), 8.02 (d, J = 7.2 Hz, 2H), 5.49 (d, J = 5.0 Hz, 1H), 5.00 (m, 3H), 4.45 (dt, J = 7.9, 5.8 Hz, 1H), 4.39 (dt, J = 8.0, 5.5 Hz, 1H), 4.27 (dt, J = 7.8, 4.7 Hz, 1H), 3.92 (dd, J = 6.5, 4.3 Hz, 1H), 3.61 (m, 8H).

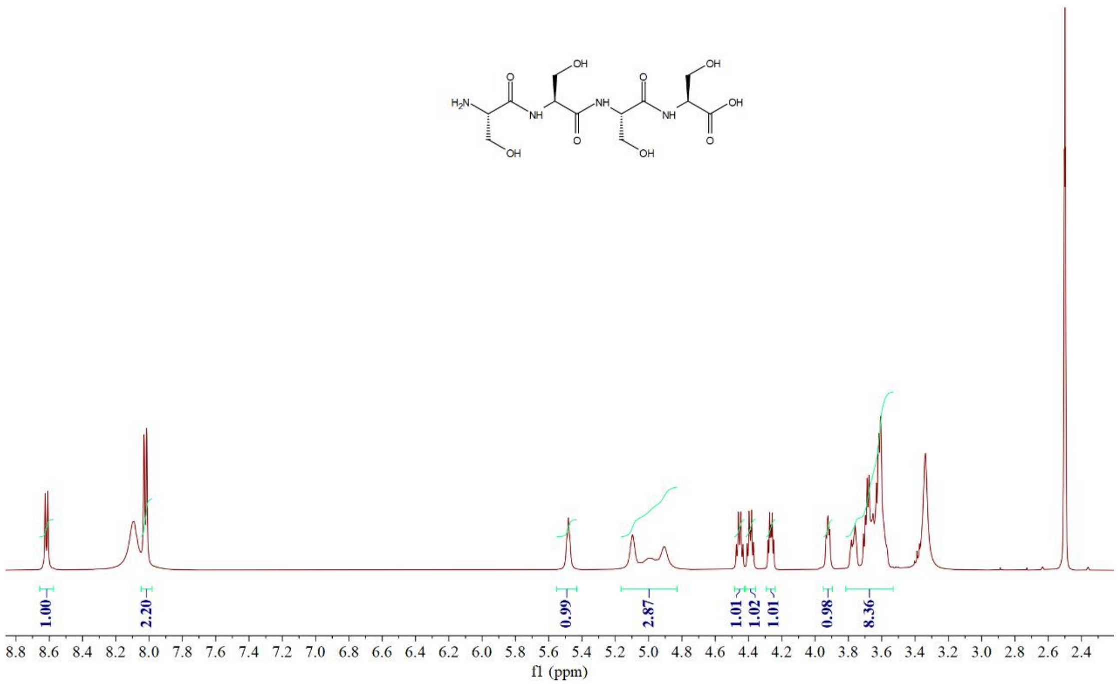

142.Compound **TTTT**: ^{1}H NMR (500 MHz, DMSO-d6) δ 8.53 (d, J = 8.1 Hz, 1H), 8.06 (d, J = 5.4 Hz, 2H), 7.87 (d, J = 8.4 Hz, 1H), 7.77 (d, J = 8.7 Hz, 1H), 4.42 (dd, J = 8.2, 4.5 Hz, 1H), 4.36 (dd, J = 8.3, 3.8 Hz, 1H), 4.21 (dd, J = 8.7, 3.1 Hz, 1H), 4.14 (dt, J = 7.9, 4.0 Hz, 1H), 4.01 (ddd, J = 10.6, 6.5, 4.2 Hz, 2H), 3.84 (q, J = 6.5 Hz, 1H), 3.76 (t, J = 6.0 Hz, 1H), 1.24 – 1.01 (m, 12H).

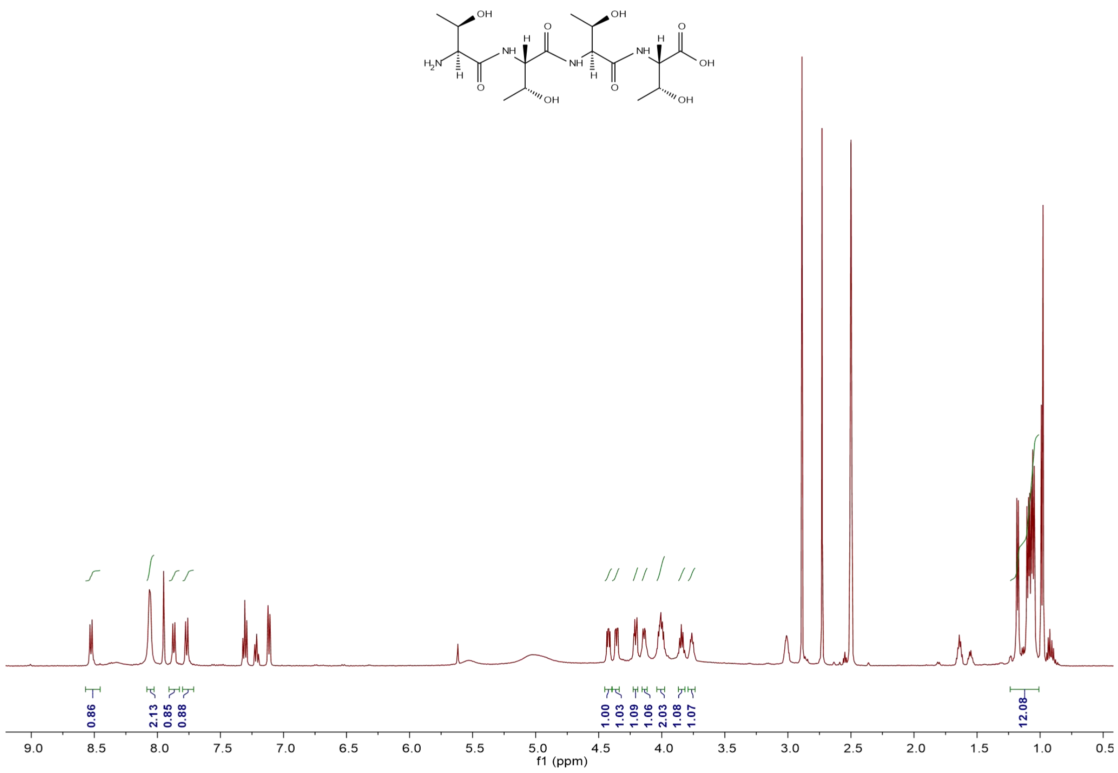

143.Compound **WWWW**: ^{1}H NMR (500 MHz, DMSO-d6) δ 10.94 (d, J = 2.6 Hz, 1H), 10.84 (s, 1H), 10.79 (t, J = 2.7 Hz, 2H), 8.86 – 8.76 (m, 1H), 8.28 – 8.18 (m, 1H), 7.85 (d, J = 5.6 Hz, 3H), 7.69 – 7.52 (m, 4H), 7.36 – 7.29 (m, 4H), 7.16 (dq, J = 7.0, 3.5 Hz, 4H), 7.09 – 6.88 (m, 8H), 4.68 (dtd, J = 16.9, 8.1, 4.9 Hz, 2H), 4.54 (q, J = 7.1 Hz, 1H), 3.94 (d, J = 7.6 Hz, 1H), 3.19 (ddt, J = 18.7, 9.9, 4.1 Hz, 4H), 3.08 – 2.90 (m, 4H).

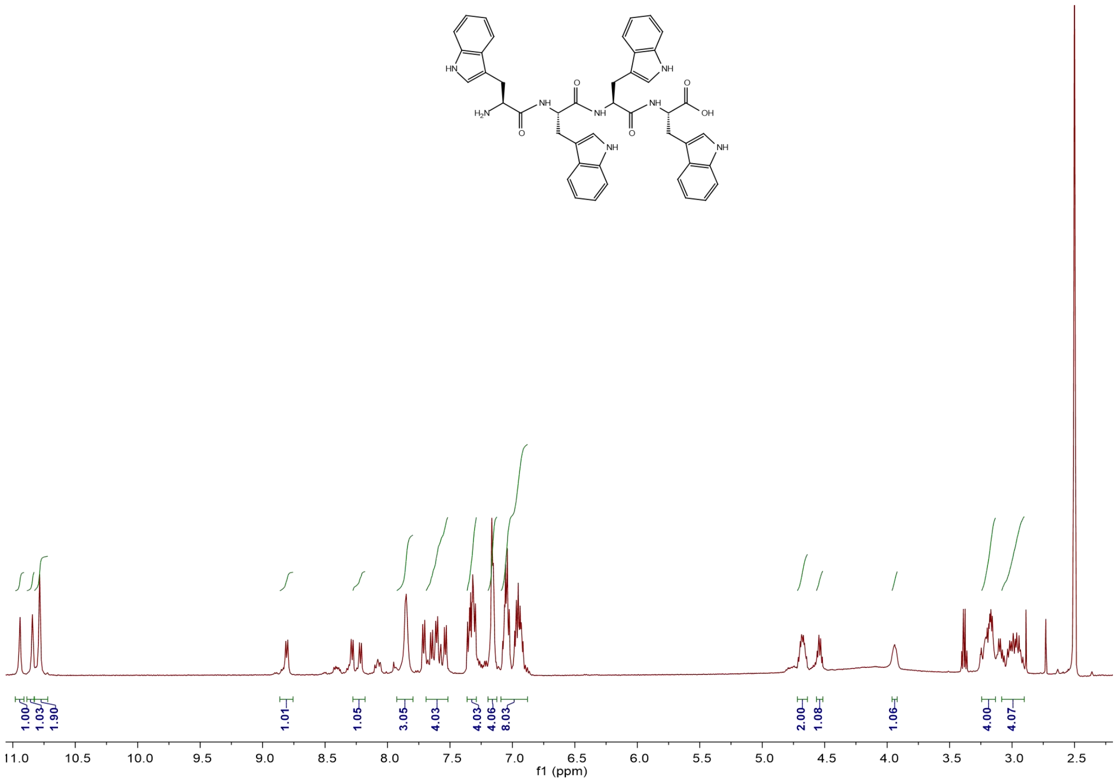

144.Compound **CWFT**: ^{1}H NMR (500 MHz, DMSO-d6) δ 8.54 (d, J = 8.1 Hz, 1H), 8.37 (d, J = 8.2 Hz, 1H), 8.07 (d, J = 8.7 Hz, 1H), 7.65 (d, J = 7.9 Hz, 1H), 7.33 (s, 1H), 7.31 – 7.10 (m, 7H), 7.06 (t, J = 7.5 Hz, 1H), 6.98 (t, J = 7.4 Hz, 1H), 4.75 (td, J = 8.9, 4.1 Hz, 1H), 4.60 (td, J = 8.7, 4.4 Hz, 1H), 4.26 (dd, J = 8.7, 3.1 Hz, 1H), 4.22 – 4.16 (m, 1H), 3.88 (s, 1H), 3.12 (ddd, J = 15.0, 11.9, 4.5 Hz, 2H), 2.88 (ddd, J = 32.9, 15.2, 8.4 Hz, 4H), 1.08 (d, J = 6.4 Hz, 3H).

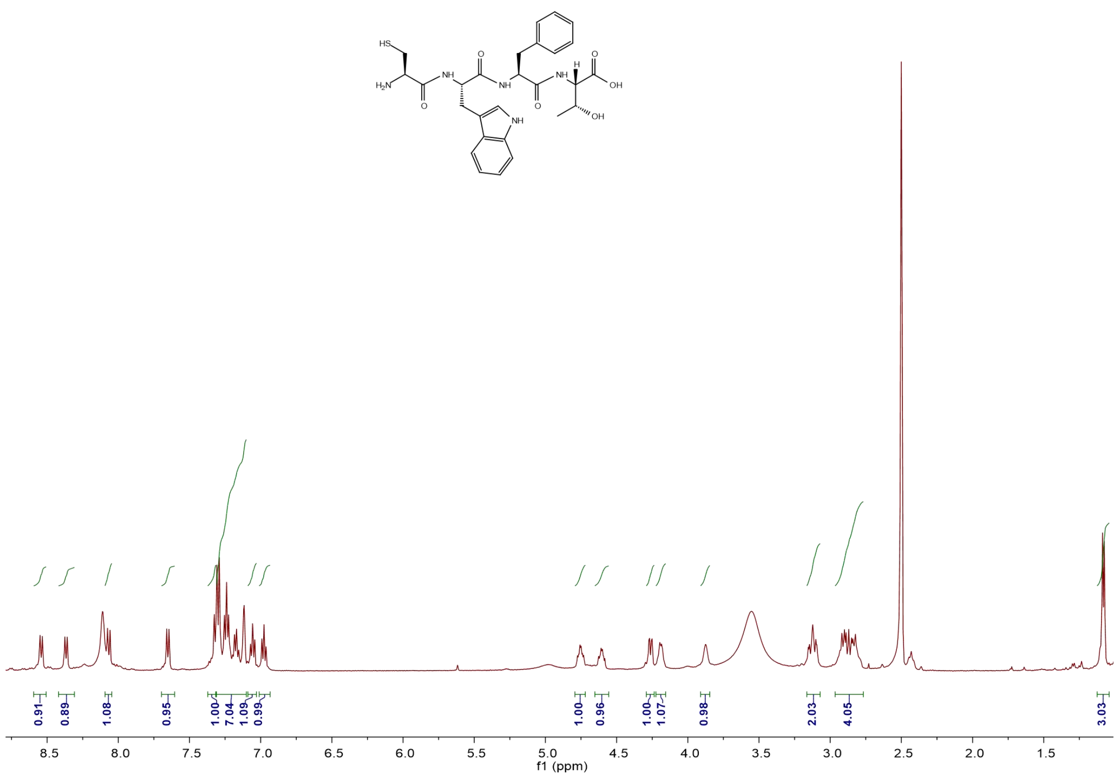

145.Compound **IRVW**: ^{1}H NMR (500 MHz, DMSO-d6) δ 8.48 (d, J = 7.8 Hz, 1H), 7.52 (d, J = 7.9 Hz, 1H), 7.32 (d, J = 8.1 Hz, 1H), 7.13 (d, J = 2.3 Hz, 1H), 7.05 (t, J = 7.5 Hz, 1H), 6.96 (t, J = 7.5 Hz, 1H), 4.47 – 4.41 (m, 1H), 4.37 (d, J = 7.3 Hz, 1H), 4.19 (t, J = 7.8 Hz, 1H), 3.65 (d, J = 5.6 Hz, 1H), 3.20 – 2.99 (m, 5H), 1.99 (h, J = 7.1 Hz, 1H), 1.77 (d, J = 9.1 Hz, 1H), 1.68 (s, 1H), 1.56 – 1.42 (m, 4H), 0.87 (d, J = 6.9 Hz, 3H), 0.81 (q, J = 7.1 Hz, 9H).

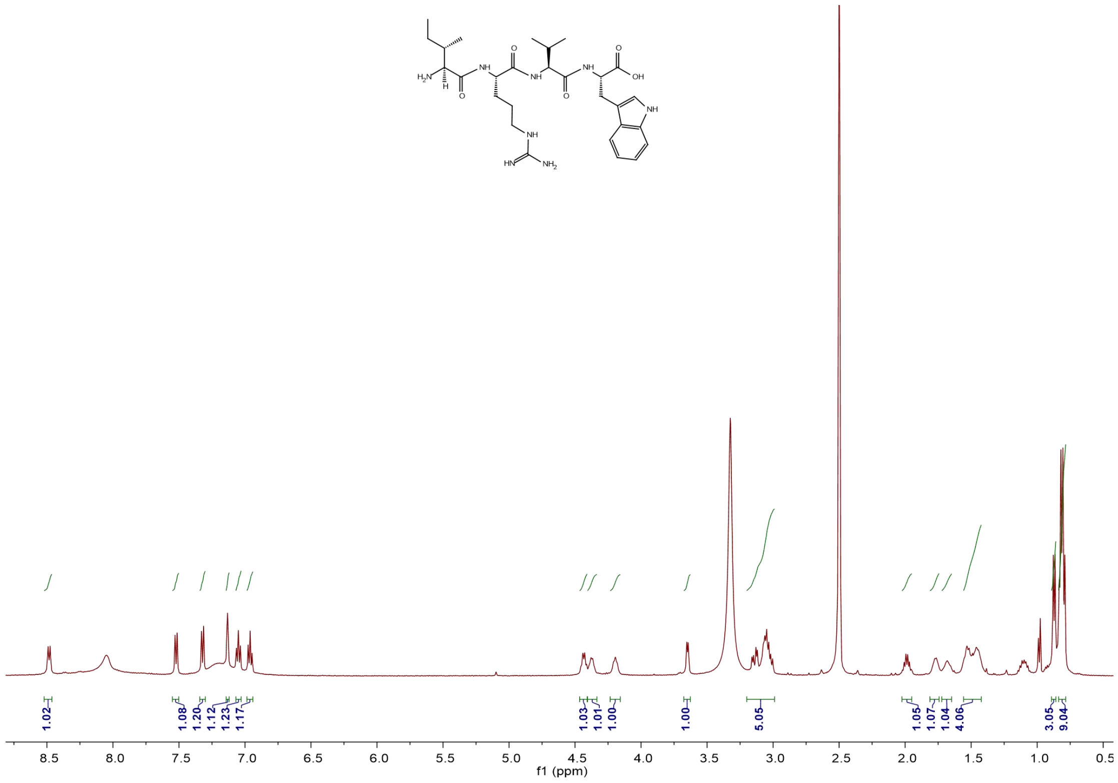

146.Compound **NVFH**: ^{1}H NMR (500 MHZ, DMSO-d6) δ 8.97 (d, *J* = 1.4 Hz, 1H), 8.42 (d, *J* = 8.0 Hz, 1H), 8.33 (d, *J* = 8.6 Hz, 1H), 8.18 (d, *J* = 8.0 Hz, 1H), 7.68 (d, *J* = 2.2 Hz, 1H), 7.35 (d, *J* = 1.3 Hz, 1H), 7.30 (d, *J* = 2.2 Hz, 1H), 7.24 (m, 4H), 7.18 (m, 1H), 4.57 (m, 1H), 4.50 (m, 1H), 4.18 (dd, *J* = 8.5, 5.8 Hz, 2H), 3.14 (dd, *J* = 15.3, 5.5 Hz, 1H), 2.98 (m, 2H), 2.78 (dd, *J* = 14.1, 9.5 Hz, 1H), 2.56 (m, 2H), 1.94 (m, 1H), 0.79 (d, *J* = 6.8 Hz, 3H), 0.73 (d, *J* = 6.8 Hz, 3H).

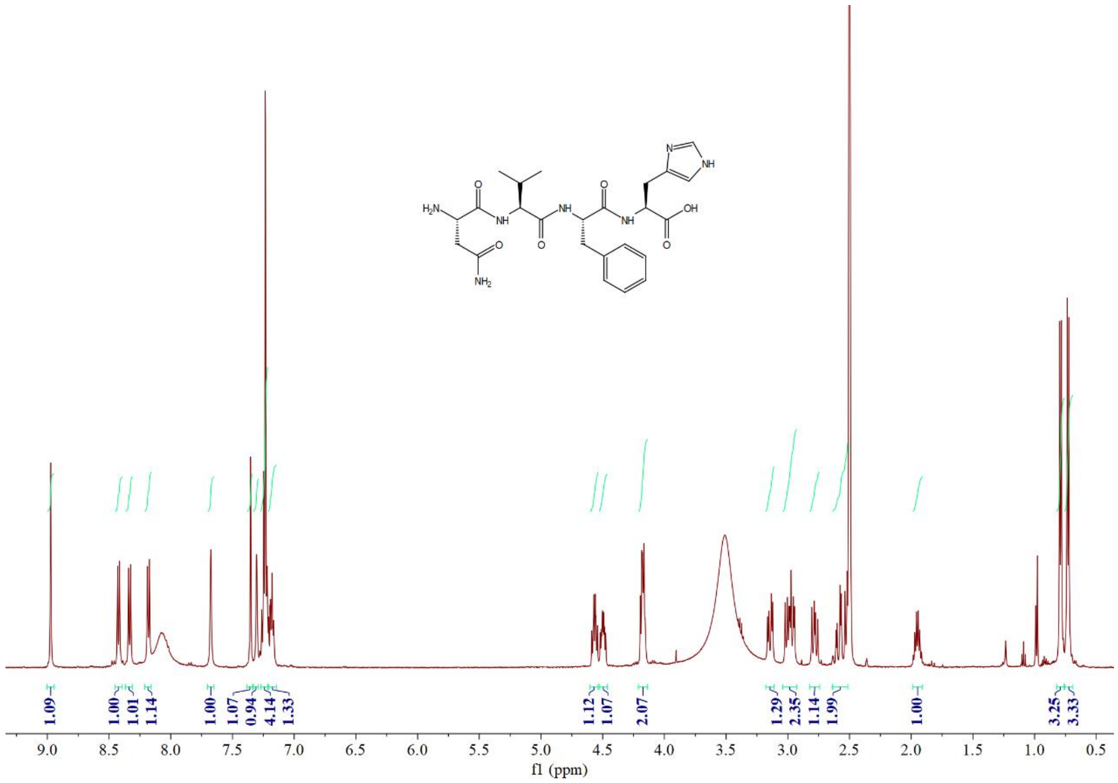

147.Compound **VFHN**: ^{1}H NMR (500 MHZ, DMSO-d6) δ 8.94 (d, *J* = 1.4 Hz, 1H), 8.57 (dd, *J* = 13.4, 8.0 Hz, 2H), 8.23 (d, *J* = 8.0 Hz, 1H), 7.46 (d, *J* = 2.4 Hz, 1H), 7.33 (m, 1H), 7.27 (d, *J* = 4.3 Hz, 4H), 7.21 (m, 1H), 6.97 (d, *J* = 2.4 Hz, 1H), 4.69 (td, *J* = 8.2, 5.6 Hz, 1H), 4.60 (m, 1H), 4.53 (dt, *J* = 8.0, 5.9 Hz, 1H), 3.59 (d, *J* = 5.2 Hz, 1H), 3.06 (m, 2H), 2.87 (m, 2H), 2.55 (d, *J* = 6.0 Hz, 2H), 2.06 (m, 1H), 0.89 (d, *J* = 6.9 Hz, 3H), 0.83 (d, *J* = 6.9 Hz, 3H).

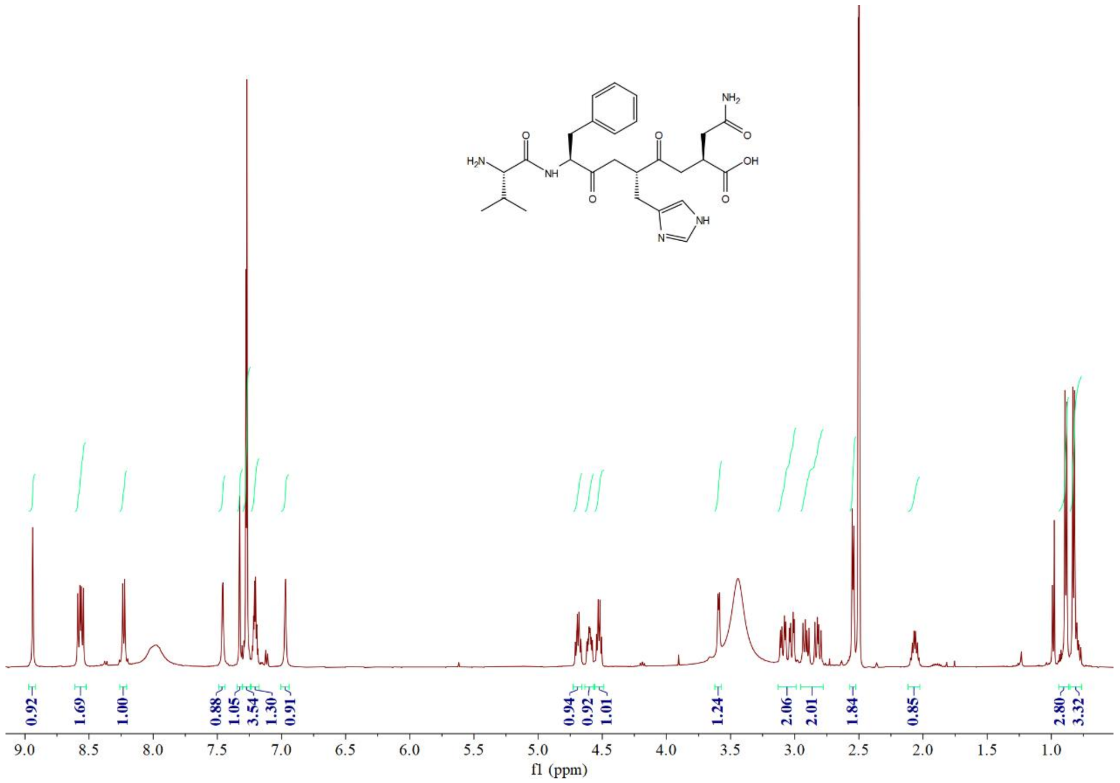

148.Compound **CFLW**: ^{1}H NMR (500 MHz, DMSO) δ 8.55 (d, *J* = 8.1 Hz, 1H), 8.31 (d, *J* = 8.4 Hz, 1H), 8.16 (d, *J* = 7.6 Hz, 1H), 7.53 (d, *J* = 7.9 Hz, 1H), 7.32 (d, *J* = 8.0 Hz, 1H), 7.28 (d, *J* = 7.4 Hz, 2H), 7.25 – 7.21 (m, 2H), 7.16 (dd, *J* = 14.0, 6.7 Hz, 4H), 7.06 (t, *J* = 7.5 Hz, 1H), 6.98 (t, *J* = 7.4 Hz, 1H), 4.64 – 4.57 (m, 1H), 4.48 (d, *J* = 6.0 Hz, 1H), 4.42 – 4.38 (m, 1H), 3.93 – 3.85 (m, 1H), 3.17 (dd, *J* = 14.7, 5.3 Hz, 2H), 3.10 – 2.98 (m, 4H), 2.84 (dd, *J* = 14.6, 4.0 Hz, 1H), 2.78 – 2.71 (m, 1H), 1.60 (dd, *J* = 13.3, 6.6 Hz, 1H), 1.45 (s, 1H), 0.88 (d, *J* = 6.5 Hz, 3H), 0.83 (d, *J* = 6.5 Hz, 3H).

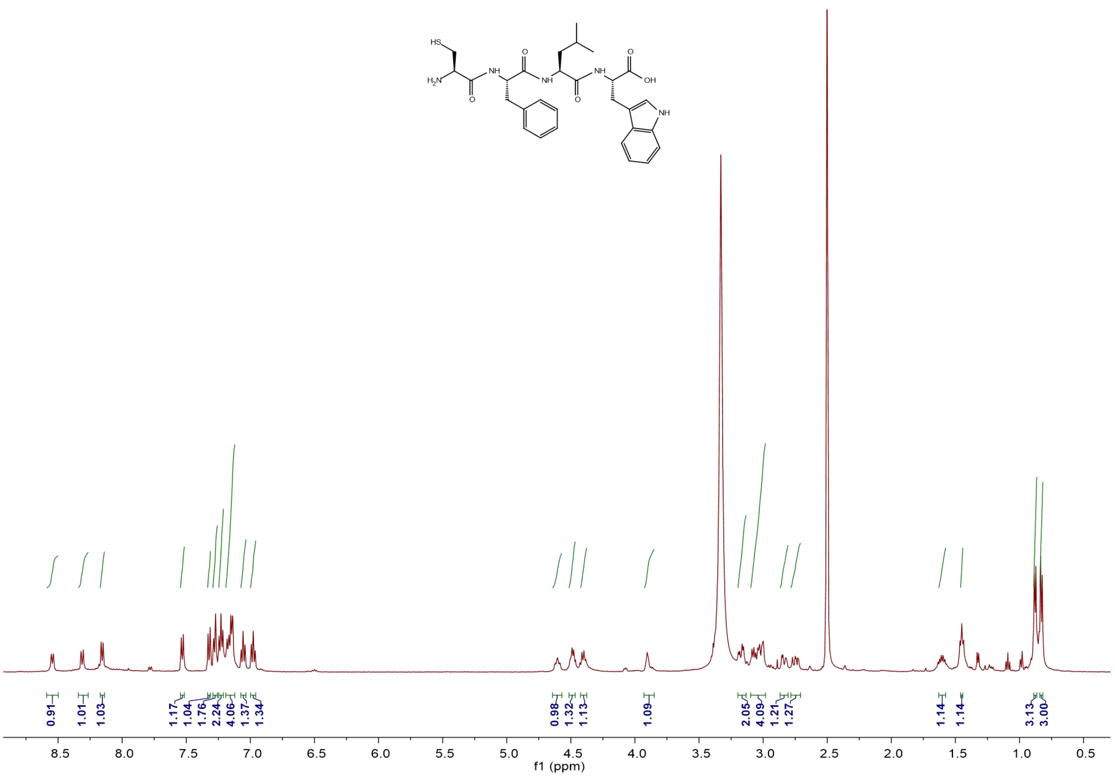

149.Compound **MMYY**: ^{1}H NMR (500 MHz, DMSO) δ 9.18 (d, *J* = 25.7 Hz, 2H), 8.46 (d, *J* = 6.8 Hz, 1H), 8.17 (d, *J* = 7.6 Hz, 1H), 8.03 (d, *J* = 8.1 Hz, 1H), 7.00 (d, *J* = 7.8 Hz, 4H), 6.63 (dd, *J* = 19.8, 8.2 Hz, 4H), 4.45 (td, *J* = 8.6, 4.6 Hz, 1H), 4.40 – 4.29 (m, 2H), 3.74 (s, 1H), 2.97 – 2.86 (m, 2H), 2.81 (dd, *J* = 13.9, 7.9 Hz, 1H), 2.65 (dd, *J* = 14.0, 9.4 Hz, 1H), 2.43 – 2.34 (m, 2H), 2.02 (t, *J* = 8.5 Hz, 6H), 1.89 (d, *J* = 5.1 Hz, 3H), 1.80 – 1.69 (m, 1H).

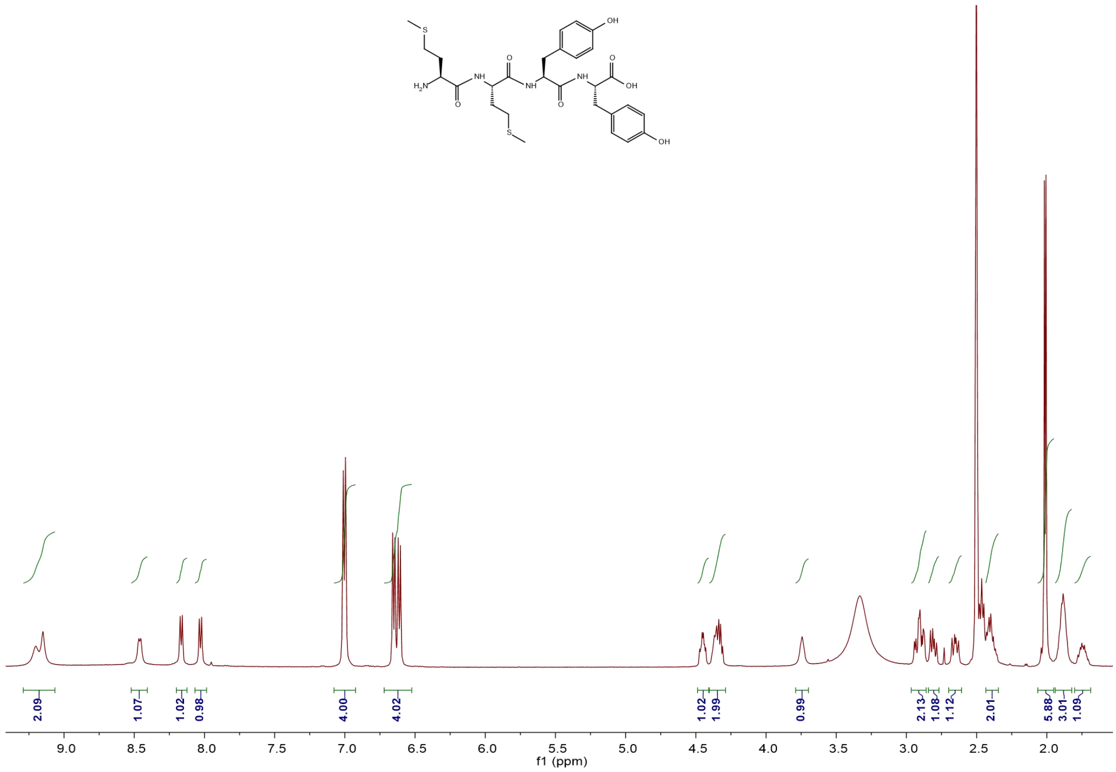

150.Compound **GLLY**: ^{1}H NMR (500 MHz, DMSO-d6) δ 9.20 (s, 1H), 8.45 (d, J = 8.3 Hz, 1H), 7.91 (d, J = 7.6 Hz, 1H), 6.98 (d, J = 8.1 Hz, 2H), 6.64 (d, J = 8.0 Hz, 2H), 4.42 (q, J = 7.7 Hz, 1H), 4.36 – 4.27 (m, 2H), 3.57 (s, 2H), 2.91 (dd, J = 14.4, 5.6 Hz, 1H), 2.80 (dd, J = 14.1, 8.1 Hz, 1H), 1.57 (dt, J = 13.5, 6.7 Hz, 2H), 1.42 (q, J = 7.4 Hz, 4H), 0.89 – 0.80 (m, 12H).

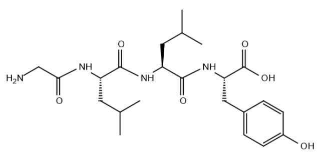

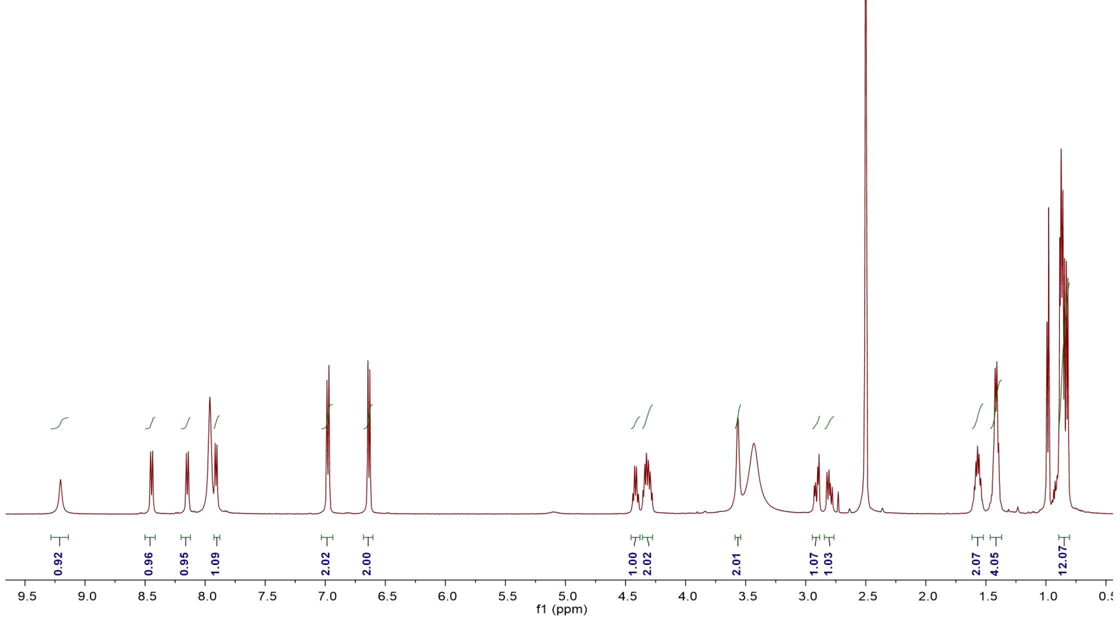

151.Compound **KMLF**: 1H NMR (500 MHz, DMSO-d6) δ 8.59 (d, J = 7.9 Hz, 1H), 8.10 (t, J = 8.9 Hz, 3H), 7.93 – 7.70 (m, 3H), 7.23 (dd, J = 23.6, 7.2 Hz, 5H), 4.42 (dtd, J = 17.6, 8.3, 5.0 Hz, 2H), 4.32 (q, J = 7.8 Hz, 1H), 3.79 (t, J = 6.5 Hz, 1H), 3.06 (dd, J = 14.0, 5.2 Hz, 1H), 2.94 – 2.89 (m, 1H), 2.73 (q, J = 7.1, 6.0 Hz, 2H), 2.44 (ddd, J = 21.8, 10.3, 5.5 Hz, 2H), 2.03 (s, 3H), 1.88 (ddt, J = 15.3, 10.9, 5.4 Hz, 1H), 1.76 (qd, J = 9.2, 4.7 Hz, 1H), 1.67 (q, J = 7.4 Hz, 2H), 1.54 (dp, J = 23.5, 7.1 Hz, 4H), 1.41 (t, J = 7.3 Hz, 3H), 1.32 (t, J = 7.8 Hz, 2H), 0.84 (dd, J = 27.4, 6.5 Hz, 6H).

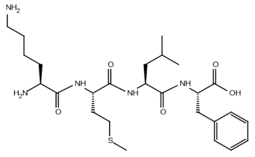

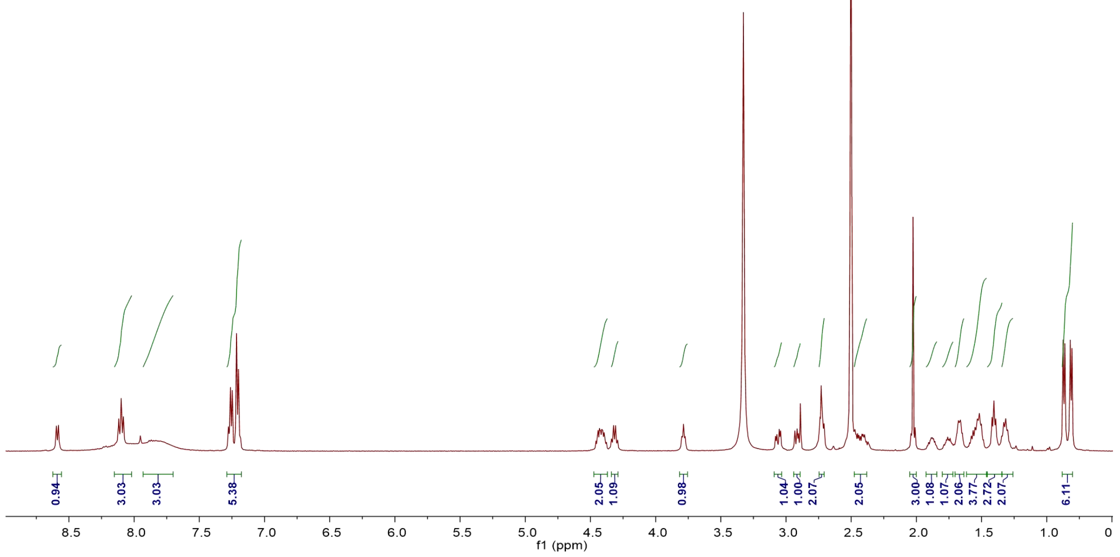

152.Compound **IWII**: ^{1}H NMR (500 MHz, DMSO) δ 8.49 (d, *J* = 7.8 Hz, 1H), 8.23 (d, *J* = 8.9 Hz, 1H), 7.92 (t, *J* = 10.0 Hz, 1H), 7.65 (d, *J* = 7.8 Hz, 1H), 7.31 (t, *J* = 9.3 Hz, 1H), 7.16 (s, 1H), 7.06 (t, *J* = 7.5 Hz, 1H), 6.96 (dd, *J* = 14.8, 7.5 Hz, 1H), 4.74 (dd, *J* = 13.4, 8.2 Hz, 1H), 4.31 (dd, *J* = 16.6, 8.3 Hz, 1H), 4.20 – 4.12 (m, 1H), 3.54 (d, *J* = 4.4 Hz, 1H), 3.08 (dd, *J* = 14.8, 5.0 Hz, 1H), 2.94 (dd, *J* = 14.8, 8.7 Hz, 1H), 1.80 (t, *J* = 18.9 Hz, 3H), 1.43 (dd, *J* = 15.1, 9.4 Hz, 3H), 1.14 – 0.99 (m, 3H), 0.88 – 0.78 (m, 18H).

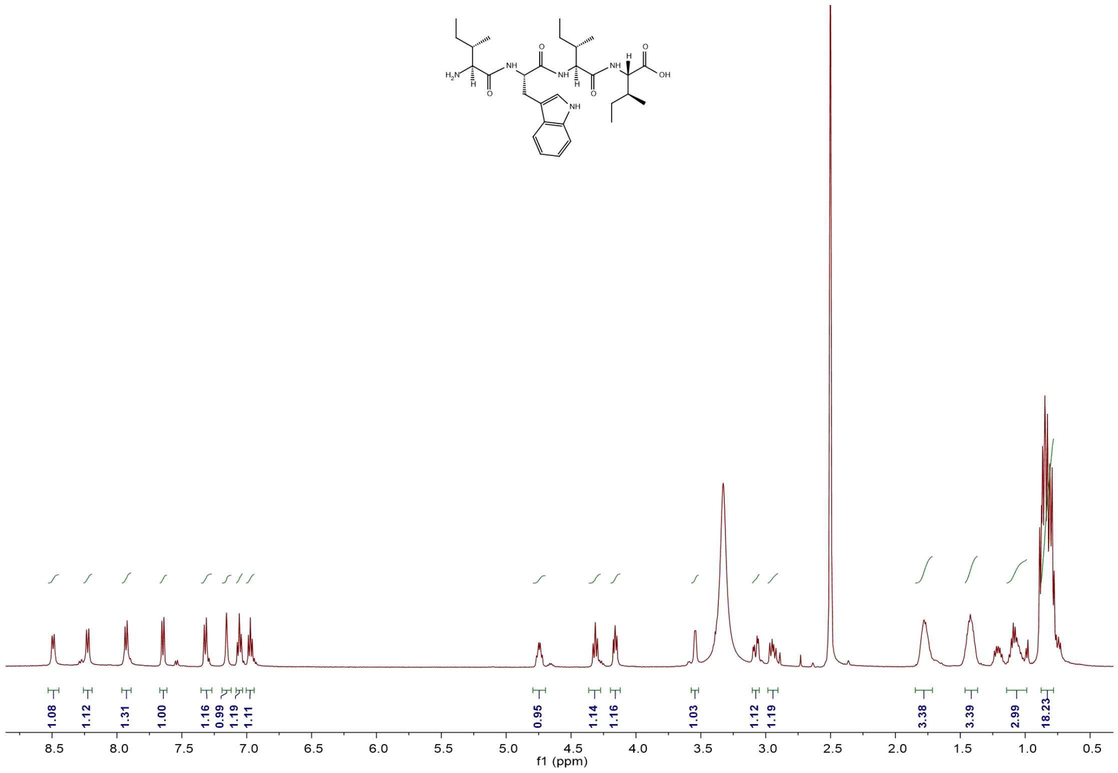

153.Compound **WVFI**: ^{1}H NMR (500 MHz, DMSO) δ 8.50 (d, J = 8.6 Hz, 1H), 8.23 (d, J = 8.1 Hz, 1H), 8.05 (d, J = 8.4 Hz, 1H), 7.64 (d, J = 8.0 Hz, 1H), 7.39 – 7.20 (m, 5H), 7.15 (d, J = 9.8 Hz, 2H), 7.06 (t, J = 7.5 Hz, 1H), 6.89 (t, J = 7.4 Hz, 1H), 4.70 (td, J = 9.0, 4.6 Hz, 1H), 4.30 – 4.15 (m, 2H), 3.99 (s, 1H), 3.05 (ddd, J = 29.0, 14.3, 4.0 Hz, 2H), 2.91 (dd, J = 14.3, 8.5 Hz, 1H), 2.80 (dd, J = 13.8, 10.0 Hz, 1H), 1.98 (dq, J = 13.2, 6.7 Hz, 1H), 1.80 (t, J = 13.1 Hz, 1H), 1.48 – 1.35 (m, 1H), 1.19 (dt, J = 21.1, 7.6 Hz, 1H), 0.91 – 0.72 (m, 12H).

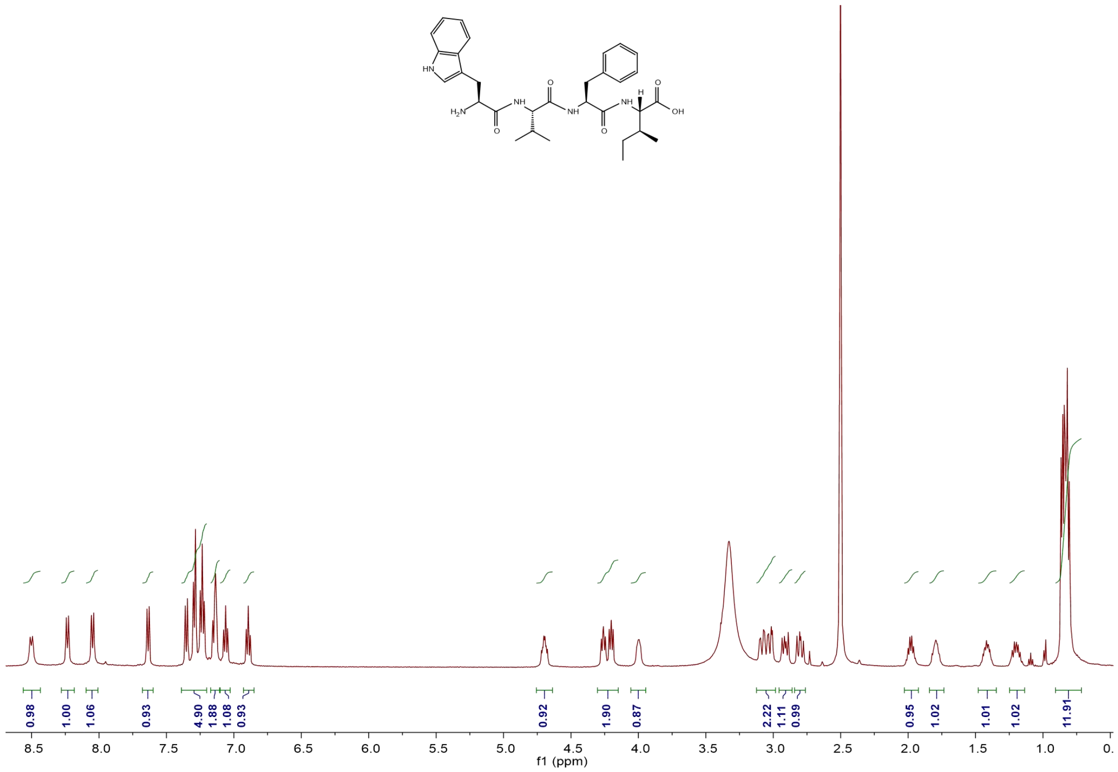

154.Compound **IIIR**: ^{1}H NMR (500 MHz, DMSO) δ 8.45 – 8.31 (m, 1H), 8.24 – 8.05 (m, 2H), 7.80 (t, *J* = 70.3 Hz, 2H), 7.24 (s, 4H), 4.27 (t, *J* = 8.5 Hz, 1H), 4.19 (t, *J* = 8.5 Hz, 1H), 4.12 (d, *J* = 5.8 Hz, 1H), 3.65 (d, *J* = 4.3 Hz, 1H), 3.08 (s, 2H), 1.74 (d, *J* = 20.9 Hz, 4H), 1.65 – 1.57 (m, 1H), 1.55 – 1.36 (m, 5H), 1.15 – 1.01 (m, 3H), 0.90 – 0.74 (m, 18H).

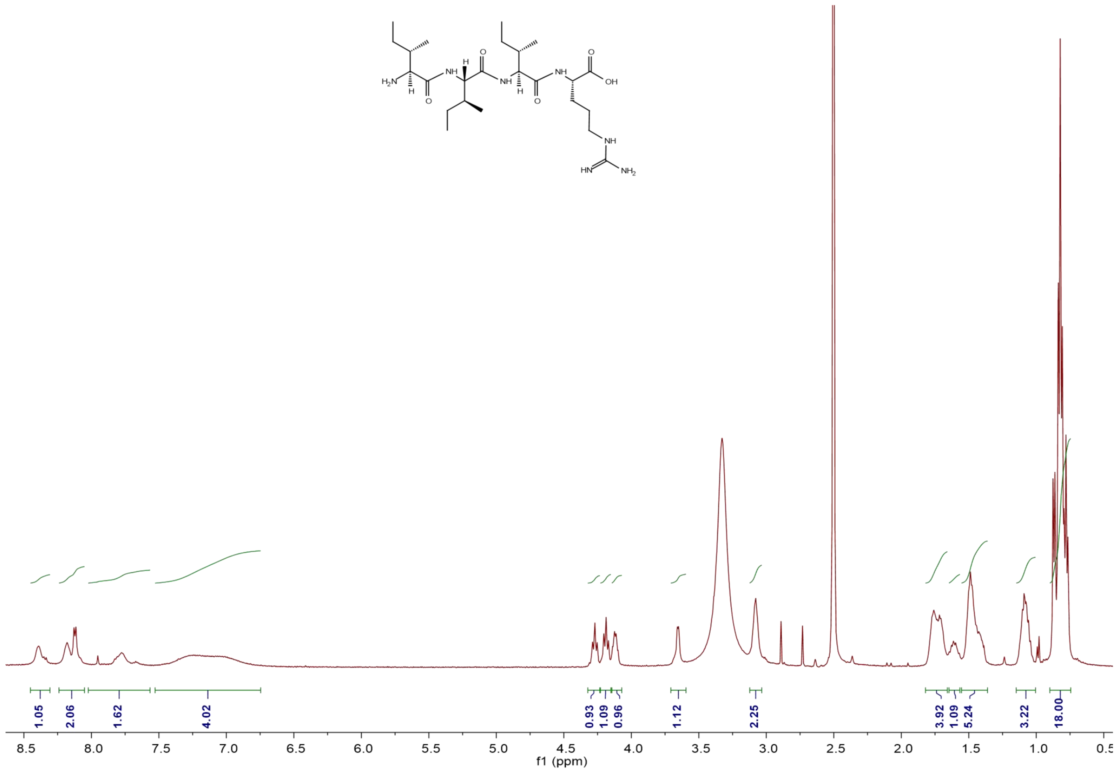

155.Compound **AVIQ**: ^{1}H NMR (500 MHz, DMSO) δ 8.39 (d, J = 8.8 Hz, 1H), 8.20 (d, J = 7.3 Hz, 1H), 8.04 (d, J = 3.7 Hz, 2H), 7.86 (d, J = 8.6 Hz, 1H), 7.22 (s, 1H), 6.77 (s, 1H), 4.27 – 4.19 (m, 2H), 4.14 (dd, J = 13.4, 7.7 Hz, 1H), 3.96 – 3.89 (m, 1H), 2.19 – 2.05 (m, 2H), 2.00 – 1.88 (m, 2H), 1.83 – 1.68 (m, 2H), 1.42 (d, J = 7.4 Hz, 1H), 1.09 (t, J = 7.1 Hz, 1H), 0.98 – 0.75 (m, 15H).

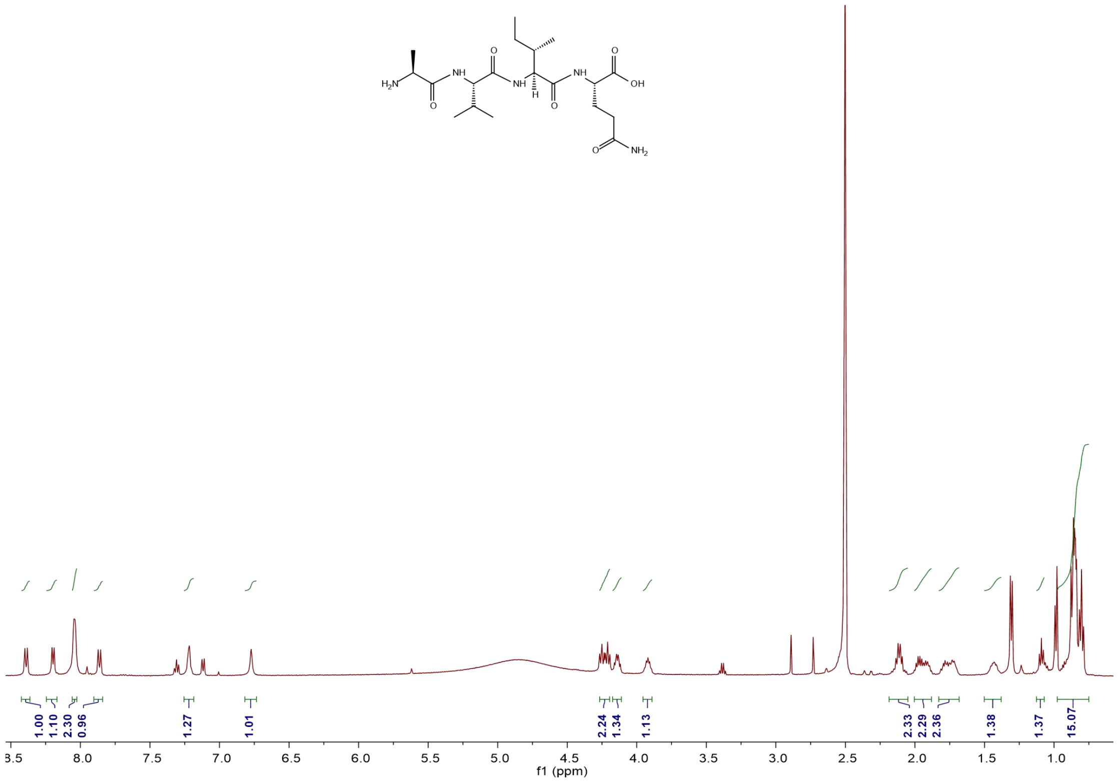

156.Compound **DALV**: ^{1}H NMR (500 MHz, DMSO) δ 8.61 (d, J = 7.4 Hz, 1H), 8.15 (s, 3H), 8.06 (d, J = 8.1 Hz, 1H), 7.83 (d, J = 8.5 Hz, 1H), 4.36 (dd, J = 15.8, 8.3 Hz, 2H), 4.12 (dd, J = 14.2, 7.9 Hz, 2H), 2.82 (dd, J = 17.7, 3.4 Hz, 1H), 2.64 (dd, J = 17.8, 8.9 Hz, 1H), 2.04 (dq, J = 13.1, 6.5 Hz, 1H), 1.62 (dt, J = 13.1, 6.6 Hz, 1H), 1.46 (t, J = 7.2 Hz, 2H), 0.87 (ddd, J = 12.8, 9.8, 6.9 Hz, 15H).

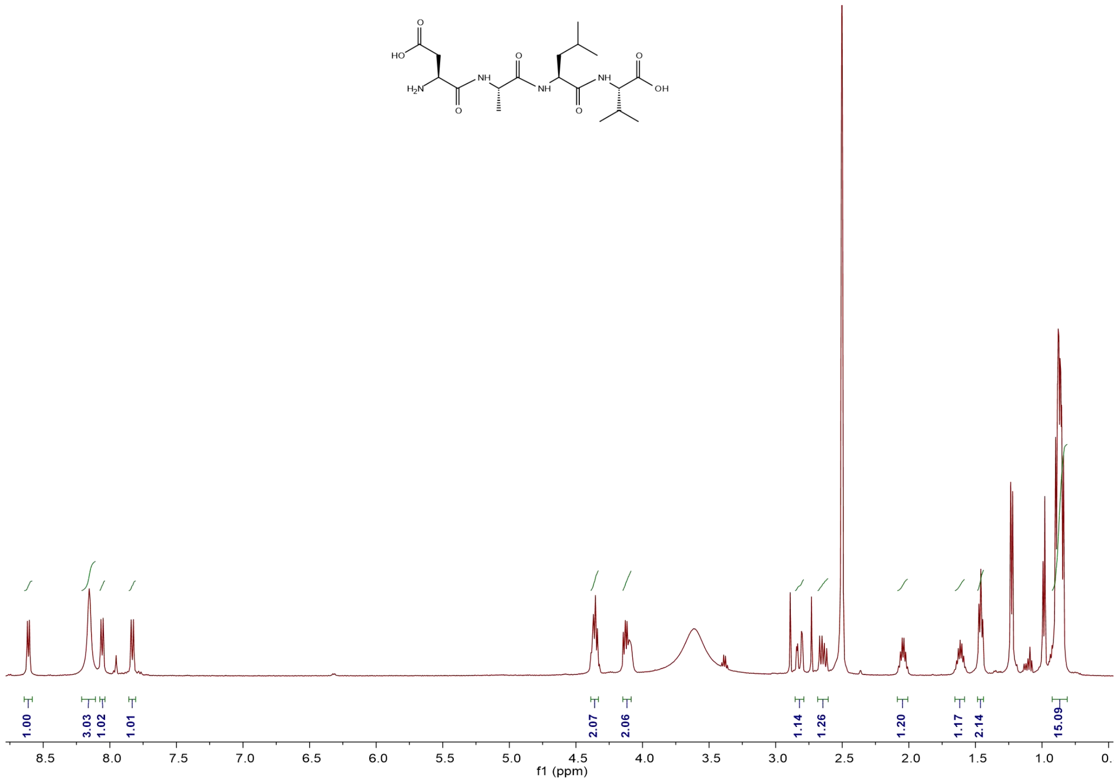

157.Compound **GVNM**: ^{1}H NMR (500 MHz, DMSO) δ 8.45 (d, *J* = 9.1 Hz, 1H), 8.32 (d, *J* = 7.3 Hz, 1H), 8.05 (d, *J* = 7.8 Hz, 1H), 7.96 (s, 2H), 7.38 – 7.29 (m, 1H), 6.91 (s, 1H), 4.57 (dd, *J* = 12.7, 7.9 Hz, 2H), 4.39 – 4.27 (m, 3H), 3.63 (dd, *J* = 16.1, 10.6 Hz, 2H), 2.46 – 2.38 (m, 2H), 2.01 (d, *J* = 14.6 Hz, 3H), 1.95 (dd, *J* = 13.0, 6.8 Hz, 2H), 1.87 – 1.78 (m, 1H), 0.89 – 0.81 (m, 6H).

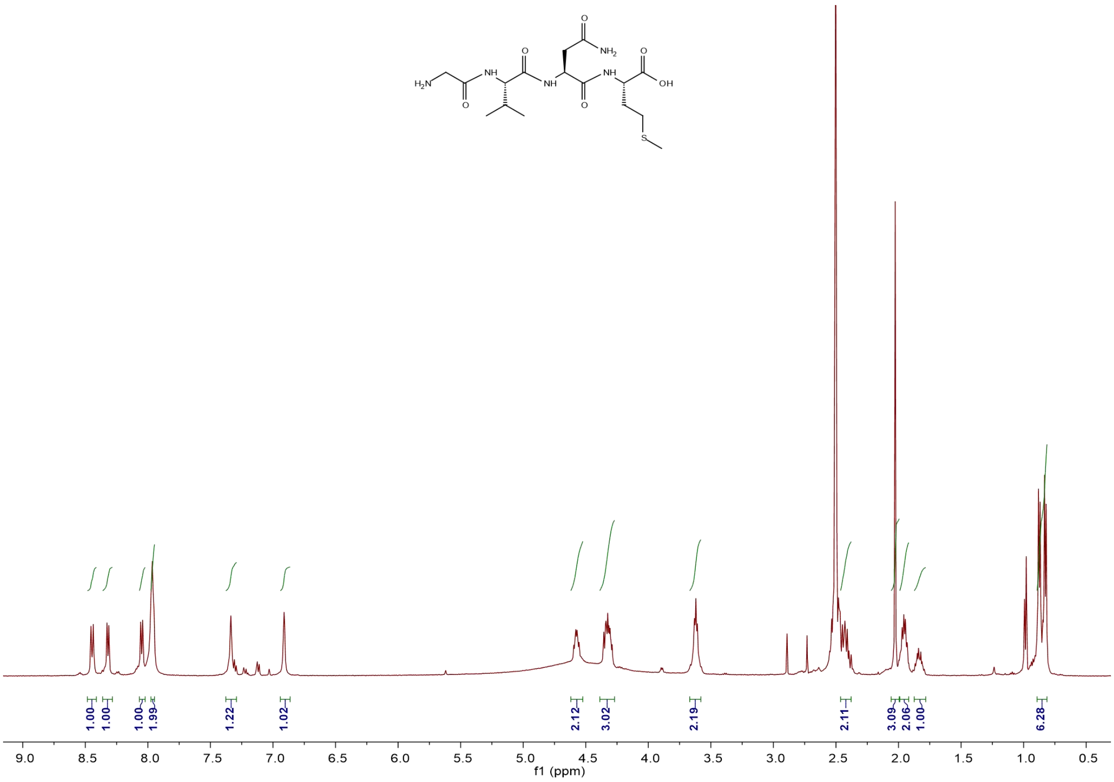

158.Compound **LALR**: ^{1}H NMR (500 MHz, DMSO) δ 8.70 (d, J = 6.2 Hz, 1H), 8.20 – 8.05 (m, 2H), 7.73 (s, 1H), 7. 24 (s, 4H), 4.40 (d, J = 6.5 Hz, 1H), 4.32 (dd, J = 14.6, 8.1 Hz, 1H), 4.14 (d, J = 4.9 Hz, 1H), 3.76 (s, 1H), 3.10 (s, 2H), 1.74 (d, J = 7.2 Hz, 1H), 1.63 (d, J = 6.5 Hz, 2H), 1.49 (dd, J = 22.9, 16.0 Hz, 5H), 1.23 (d, J = 6.2 Hz, 2H), 1.03 – 0.78 (m, 15H).

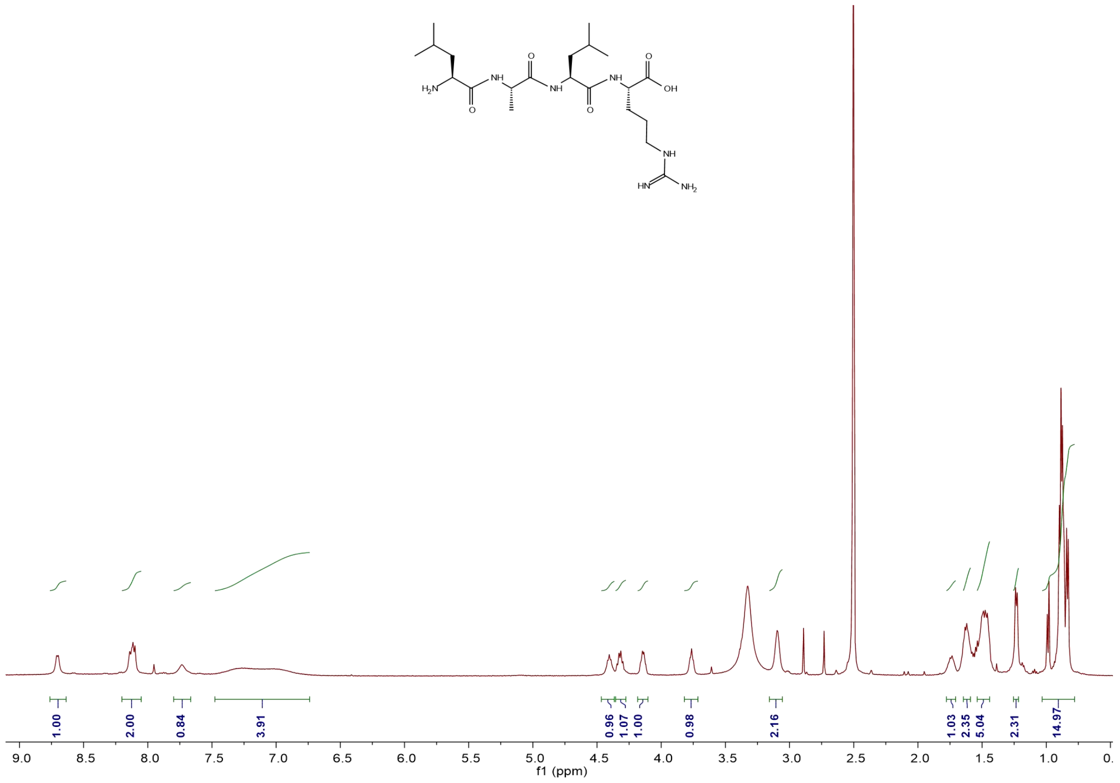

159.Compound **LLRQ**: ^{1}H NMR (500 MHz, DMSO-d6) δ 8.55 (d, J = 8.0 Hz, 1H), 8.10 (dd, J = 16.3, 6.4 Hz, 4H), 7.55 (t, J = 5.8 Hz, 1H), 7.24 (d, J = 9.8 Hz, 2H), 7.12 (d, J = 7.0 Hz, 1H), 6.79 (s, 1H), 4.40 (q, J = 7.7 Hz, 1H), 4.27 (q, J = 7.5, 7.0 Hz, 1H), 4.15 (q, J = 7.6 Hz, 1H), 3.79 (s, 1H), 3.38 (q, J = 7.0 Hz, 1H), 3.09 (q, J = 6.4 Hz, 2H), 2.11 (dd, J = 9.4, 6.2 Hz, 1H), 1.97 – 1.90 (m, 1H), 1.79 (dt, J = 15.1, 4.7 Hz, 1H), 1.67 – 1.62 (m, 2H), 1.50 (ddt, J = 19.5, 14.3, 7.0 Hz, 8H), 0.89 (t, J = 6.6 Hz, 12H).

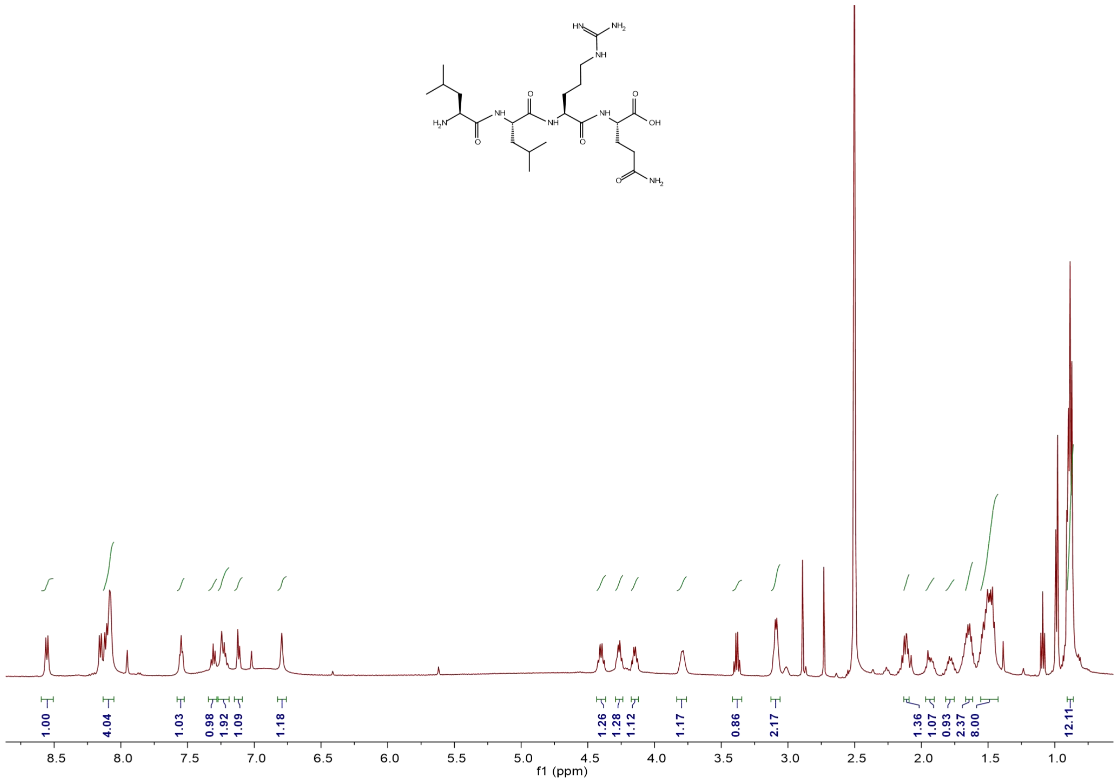

160.Compound **MVQG**: ^{1}H NMR (500 MHz, DMSO) δ 8.41 (d, J = 8.2 Hz, 1H), 8.17 (dd, J = 16.7, 9.2 Hz, 4H), 7.25 – 7.16 (m, 1H), 6.77 (s, 1H), 4.30 – 4.21 (m, 2H), 3.95 (d, J = 5.3 Hz, 2H), 3.81 (dd, J = 17.5, 5.9 Hz, 2H), 3.72 (d, J = 5.5 Hz, 2H), 3.69 (d, J = 5.5 Hz, 2H), 2.11 (t, J = 8.0 Hz, 2H), 2.05 (s, 3H), 1.96 (d, J = 6.4 Hz, 1H), 1.87 (dd, J = 14.4, 6.9 Hz, 1H), 1.80 – 1.73 (m, 1H), 0.88 (dd, J = 13.7, 6.7 Hz, 6H).

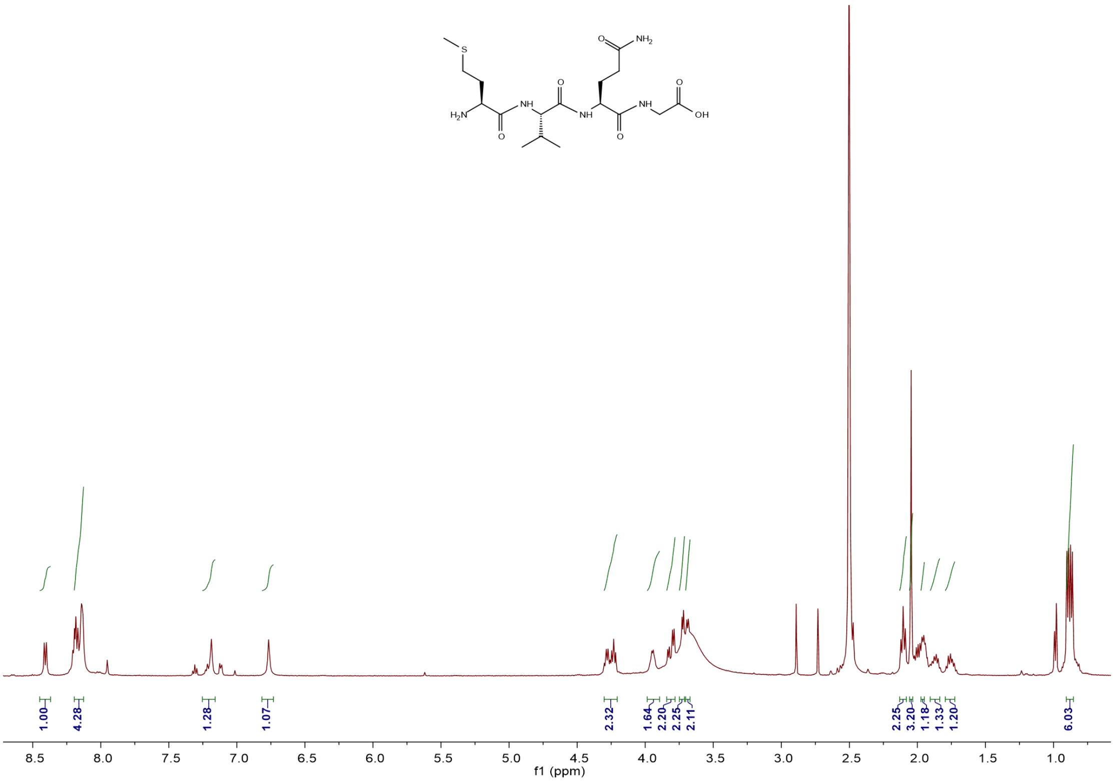

161.Compound **VALE**: ^{1}H NMR (500 MHz, DMSO) δ 8.53 (d, J = 7.4 Hz, 1H), 8.07 (d, J = 16.4 Hz, 4H), 4.47 – 4.38 (m, 1H), 4.37 – 4.27 (m, 2H), 4.22 – 4.16 (m, 1H), 3.58 (d, J = 5.0 Hz, 2H), 2.27 (d, J = 7.9 Hz, 2H), 2.04 – 1.95 (m, 2H), 1.82 – 1.75 (m, 1H), 1.63 (td, J = 13.2, 6.6 Hz, 1H), 0.93 – 0.82 (m, 15H).

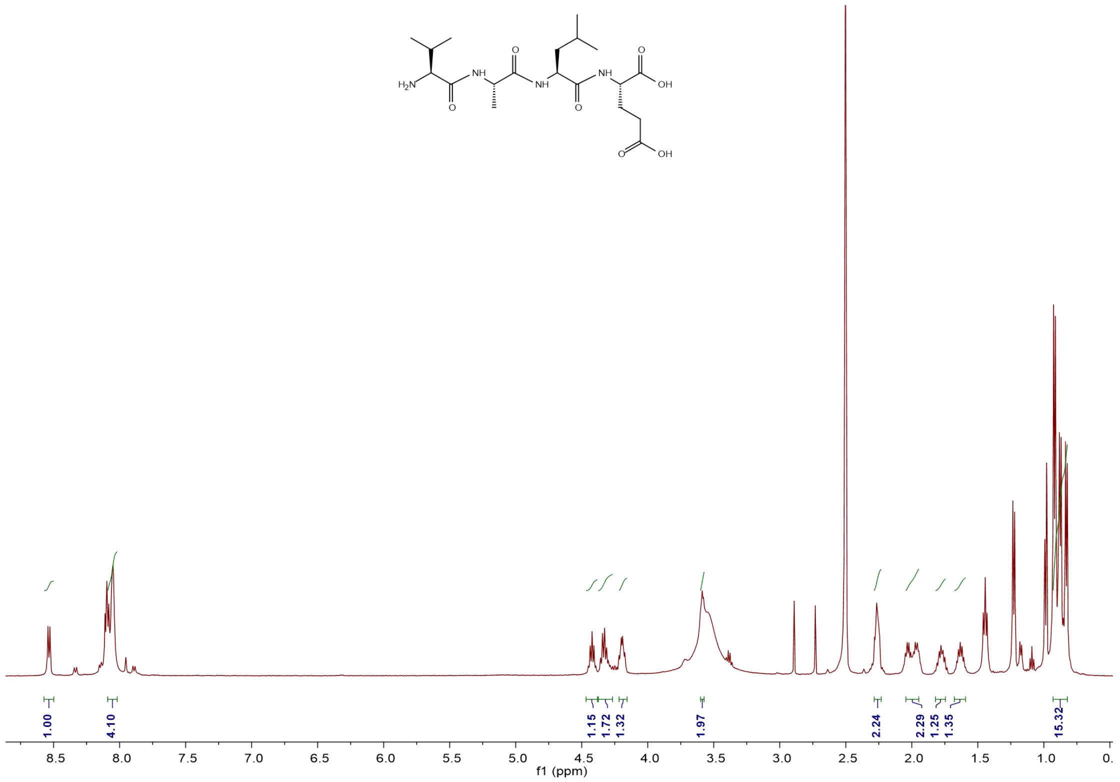

162.Compound **FAFP**: ^{1}H NMR (500 MHz, DMSO) δ 12.51 (s, 1H), 8.60 (dd, *J* = 18.1, 7.5 Hz, 1H), 8.31 (d, *J* = 7.9 Hz, 1H), 8.06 (s, 2H), 7.24 (ddt, *J* = 28.4, 13.8, 7.2 Hz, 10H), 4.65 (dt, *J* = 36.0, 18.0 Hz, 1H), 4.44 – 4.32 (m, 1H), 4.25 (dd, *J* = 8.6, 4.4 Hz, 1H), 4.02 (s, 1H), 3.64 (dd, *J* = 16.1, 7.2 Hz, 1H), 3.47 (dd, *J* = 15.8, 6.5 Hz, 1H), 3.12 – 2.96 (m, 2H), 2.91 – 2.75 (m, 2H), 2.14 (dt, *J* = 15.8, 7.4 Hz, 1H), 1.85 (ddd, *J* = 54.3, 31.7, 24.9 Hz, 3H), 1.19 (t, *J* = 10.0 Hz, 3H).

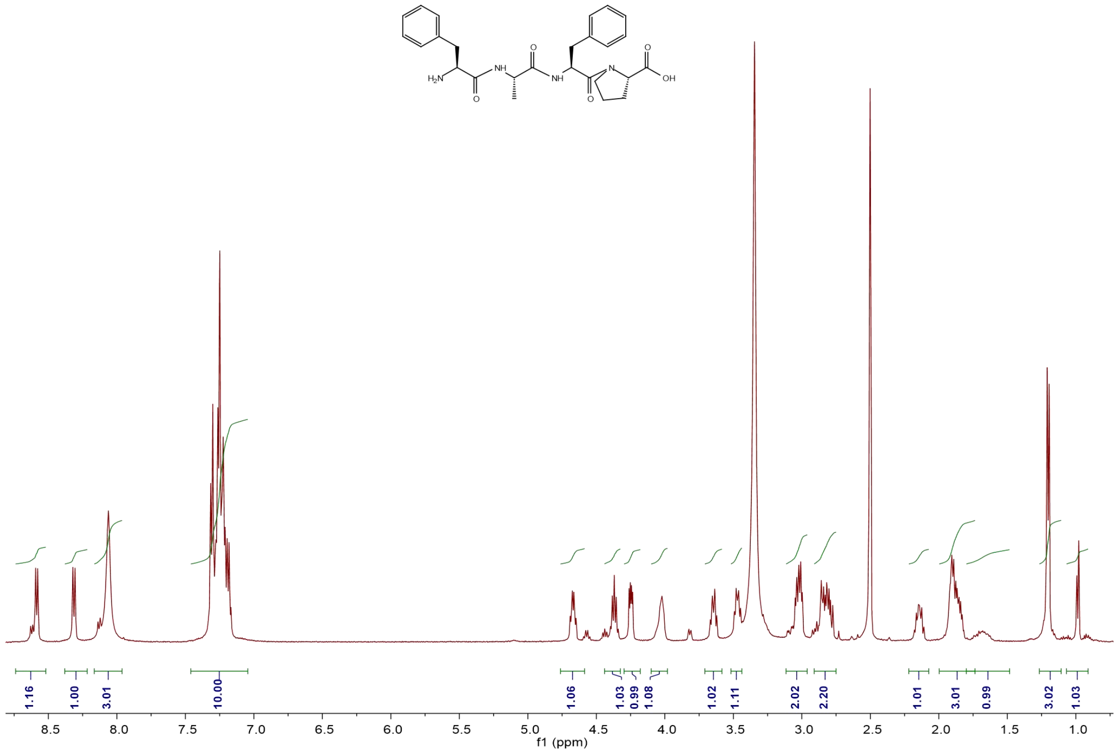

163.Compound **PWWC**: ^{1}H NMR (500 MHz, DMSO) δ 9.05 (s, 1H), 8.64 (d, *J* = 8.2 Hz, 1H), 8.29 (dt, *J* = 28.2, 14.1 Hz, 2H), 7.62 (dd, *J* = 15.6, 7.9 Hz, 2H), 7.32 (d, *J* = 8.1 Hz, 2H), 7.17 – 7.12 (m, 2H), 7.06 (t, *J* = 7.5 Hz, 2H), 6.97 (td, *J* = 7.5, 2.7 Hz, 2H), 4.69 – 4.60 (m, 2H), 4.47 (dd, *J* = 12.1, 7.0 Hz, 1H), 4.03 (t, *J* = 7.2 Hz, 1H), 3.20 – 3.13 (m, 4H), 3.01 (dd, *J* = 14.8, 8.7 Hz, 1H), 2.89 (dd, *J* = 9.2, 5.6 Hz, 2H), 2.80 (dd, *J* = 14.3, 7.0 Hz, 1H), 2.23 – 2.16 (m, 1H), 1.78 (dt, *J* = 13.6, 6.9 Hz, 2H), 1.72 – 1.66 (m, 1H), 1.09 (t, *J* = 7.0 Hz, 1H).

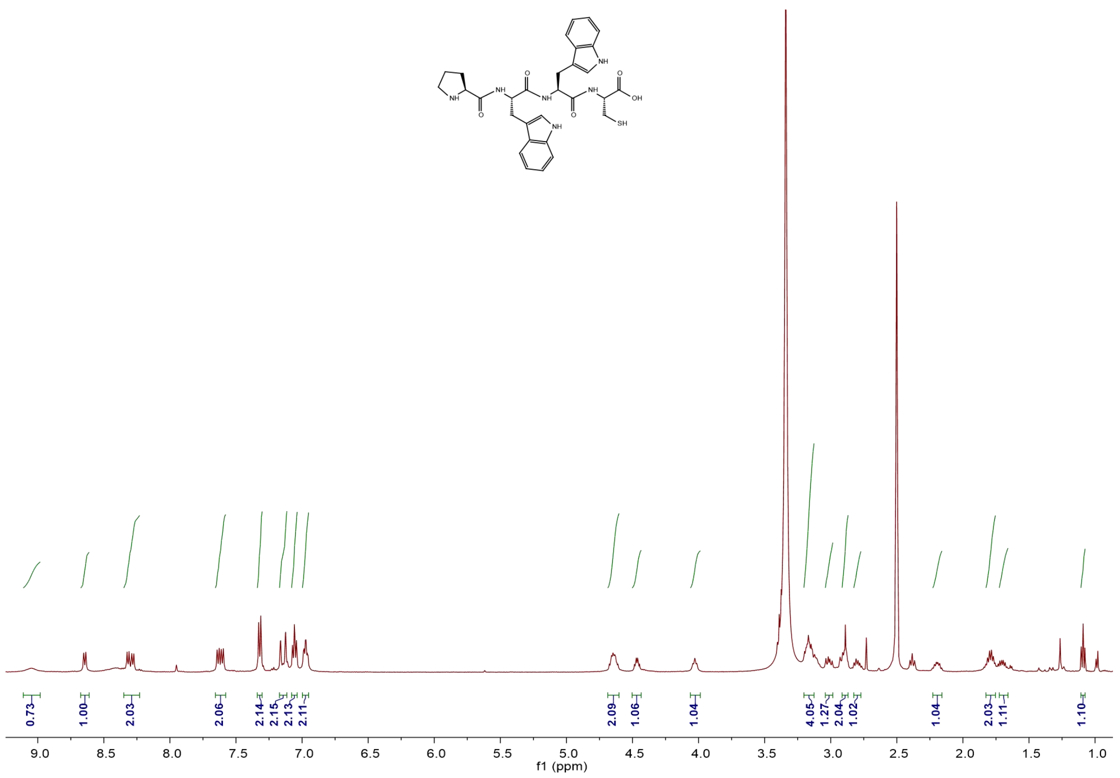

164.Compound **TRFS**: ^{1}H NMR (500 MHz, DMSO-d6) δ 8.52 (d, J = 8.0 Hz, 1H), 8.34 (d, J = 7.8 Hz, 1H), 8.19 (d, J = 8.3 Hz, 1H), 7.59 (s, 1H), 7.28 – 7.17 (m, 5H), 7.16 – 7.07 (m, 1H), 5.52 (s, 1H), 4.64 (td, J = 9.2, 3.9 Hz, 1H), 4.37 – 4.22 (m, 2H), 3.74 (dt, J = 10.2, 6.0 Hz, 2H), 3.64 (dd, J = 10.9, 4.1 Hz, 1H), 3.55 (d, J = 7.2 Hz, 1H), 3.07 (p, J = 6.8, 5.5 Hz, 3H), 2.77 (dd, J = 14.1, 10.0 Hz, 1H), 1.66 – 1.37 (m, 4H), 1.07 – 0.95 (m, 3H).

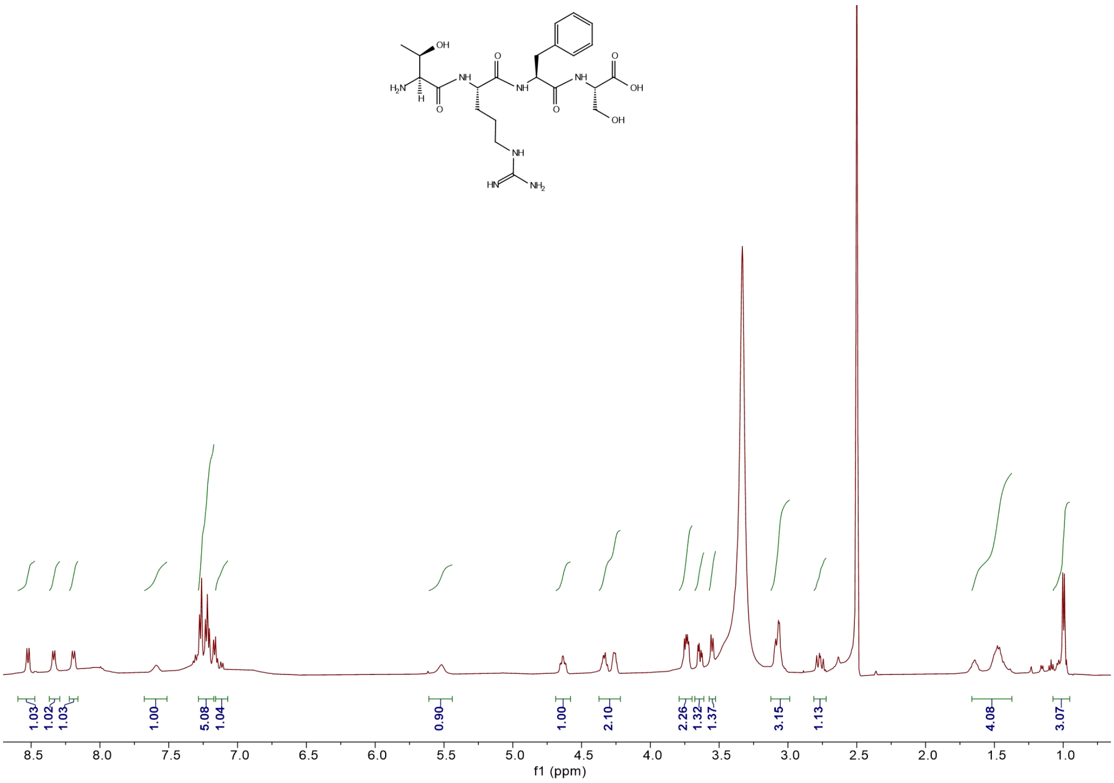

165.Compound **NNNN**: ^{1}H NMR (500 MHz, DMSO-d6) δ 8.66 (d, J = 7.7 Hz, 1H), 8.24 – 7.89 (m, 5H), 7.74 – 7.64 (m, 1H), 7.43 – 7.23 (m, 4H), 6.95 – 6.87 (m, 2H), 4.56 (dtd, J = 10.5, 7.8, 5.1 Hz, 2H), 4.49 – 4.44 (m, 1H), 4.07 (dd, J = 9.1, 4.6 Hz, 1H), 2.65 – 2.51 (m, 6H), 2.47 – 2.34 (m, 2H).

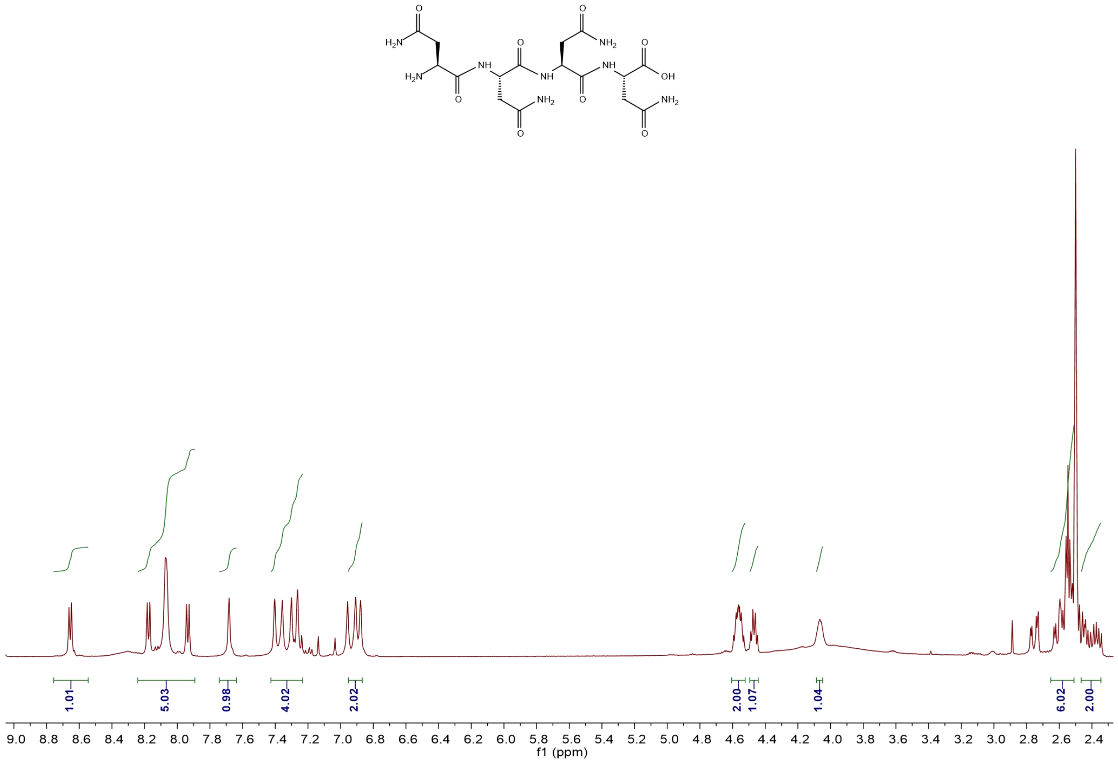
1.01
5.03
0.98
4.02
2.02
2.00
1.07
1.04
6.02
2.00
9.0 8.8 8.6 8.4 8.2 8.0 7.8 7.6 7.4 7.2 7.0 6.8 6.6 6.4 6.2 6.0 5.8 5.6 5.4 5.2 5.0 4.8 4.6 4.4 4.2 4.0 3.8 3.6 3.4 3.2 3.0 2.8 2.6 2.4
f1 (ppm)
