# Supplementary material for: Accelerating the prediction and discovery of peptide hydrogels with human-in-the-loop
Source: Nat Commun. 2023 Jun 30;14:3880. doi: 10.1038/s41467-023-39648-2 (PMC10313671; doi:10.1038/s41467-023-39648-2)
Supplement: Supplementary file 9 — Supplementary Data 6 [file 41467_2023_39648_MOESM9_ESM.pdf]

Hydrogels:

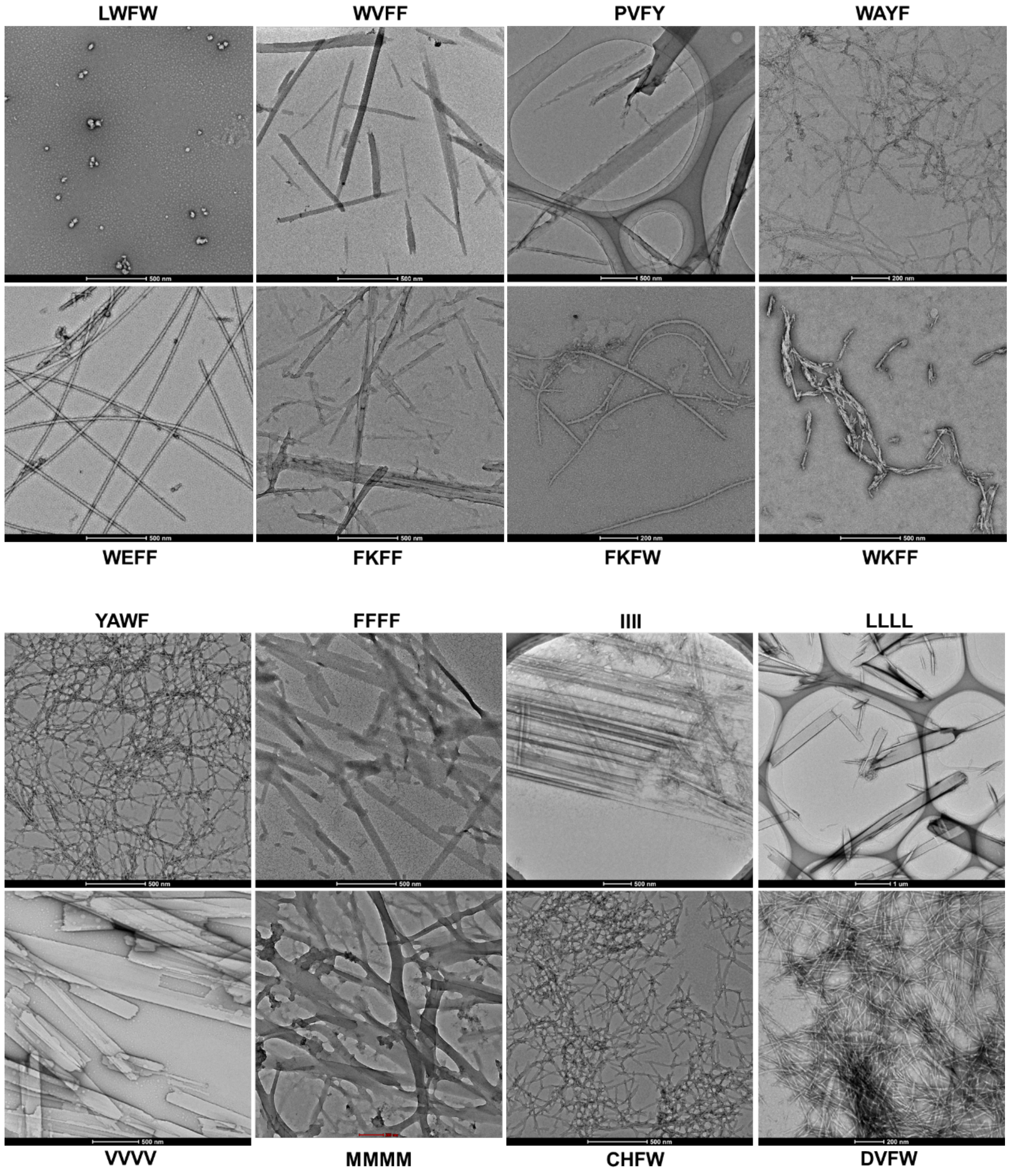
LWFW
WVFF
PVFY
WAYF
WEFF
FKFF
FKFW
WKFF
YAWF
FFFF
IIII
LLLL
VVVV
MMMM
CHFW
DVFW

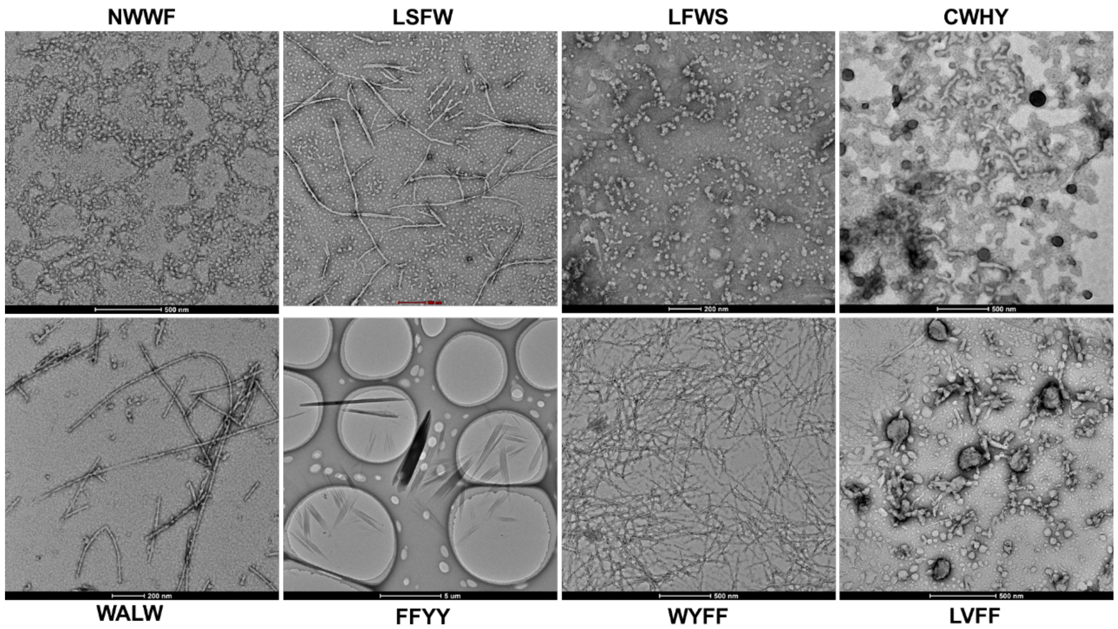
NWWF
LSFW
LFWS
CWHY
WALW
FFYY
WYFF
LVFF

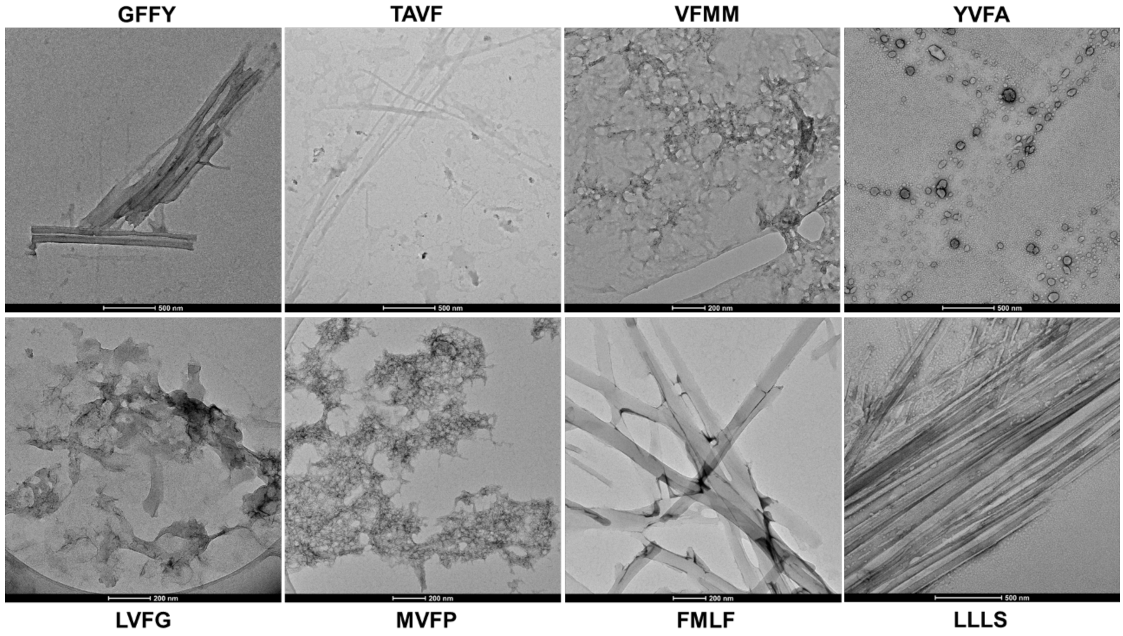
GFFY
TAVF
VFMM
YVFA
LVFG
MVFP
FMLF
LLLS

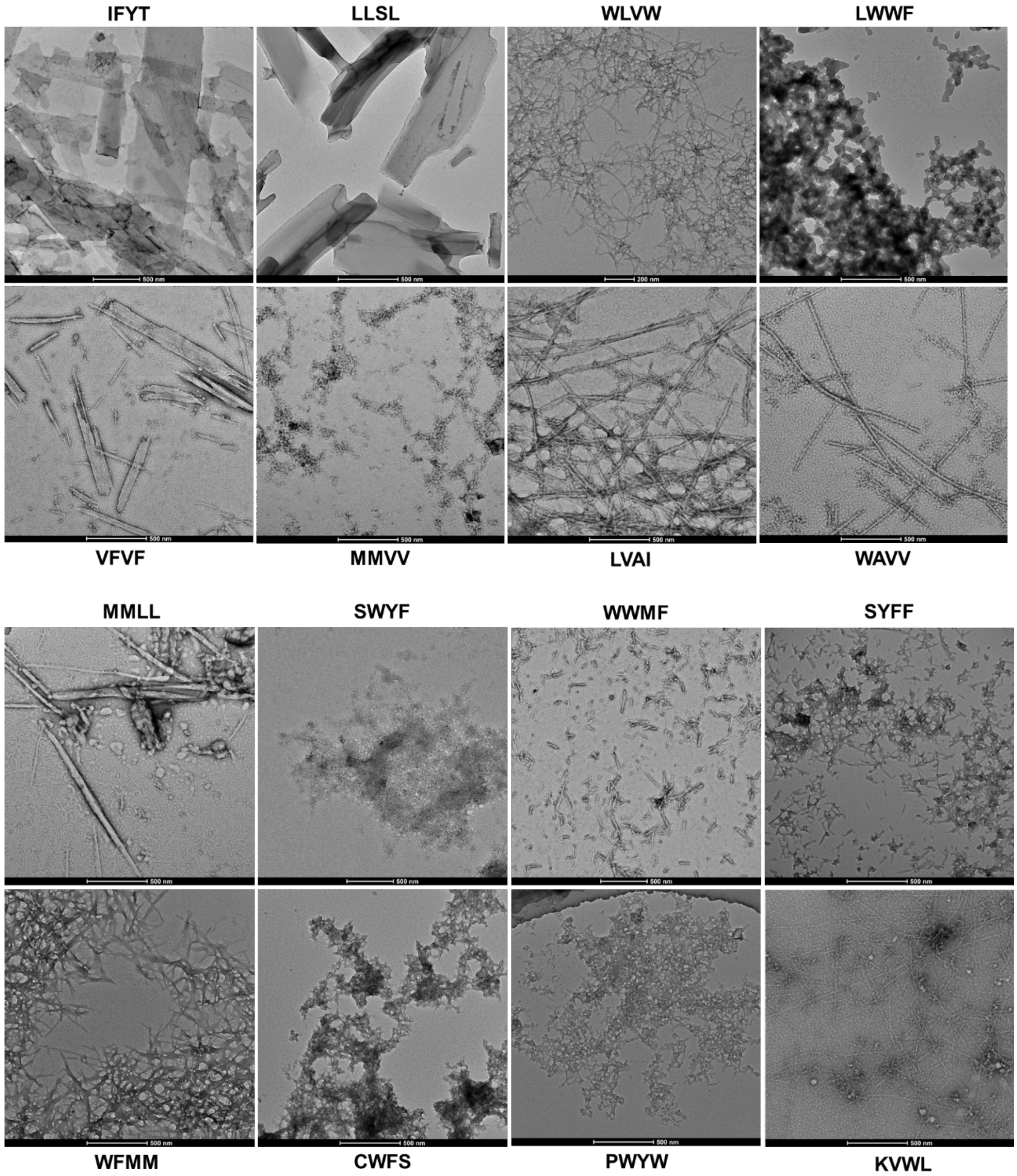
IFYT
LLSL
WLVW
LWWF
VFVF
MMVV
LVAI
WAVV
MMLL
SWYF
WWMF
SYFF
WFMM
CWFS
PWYW
KVWL

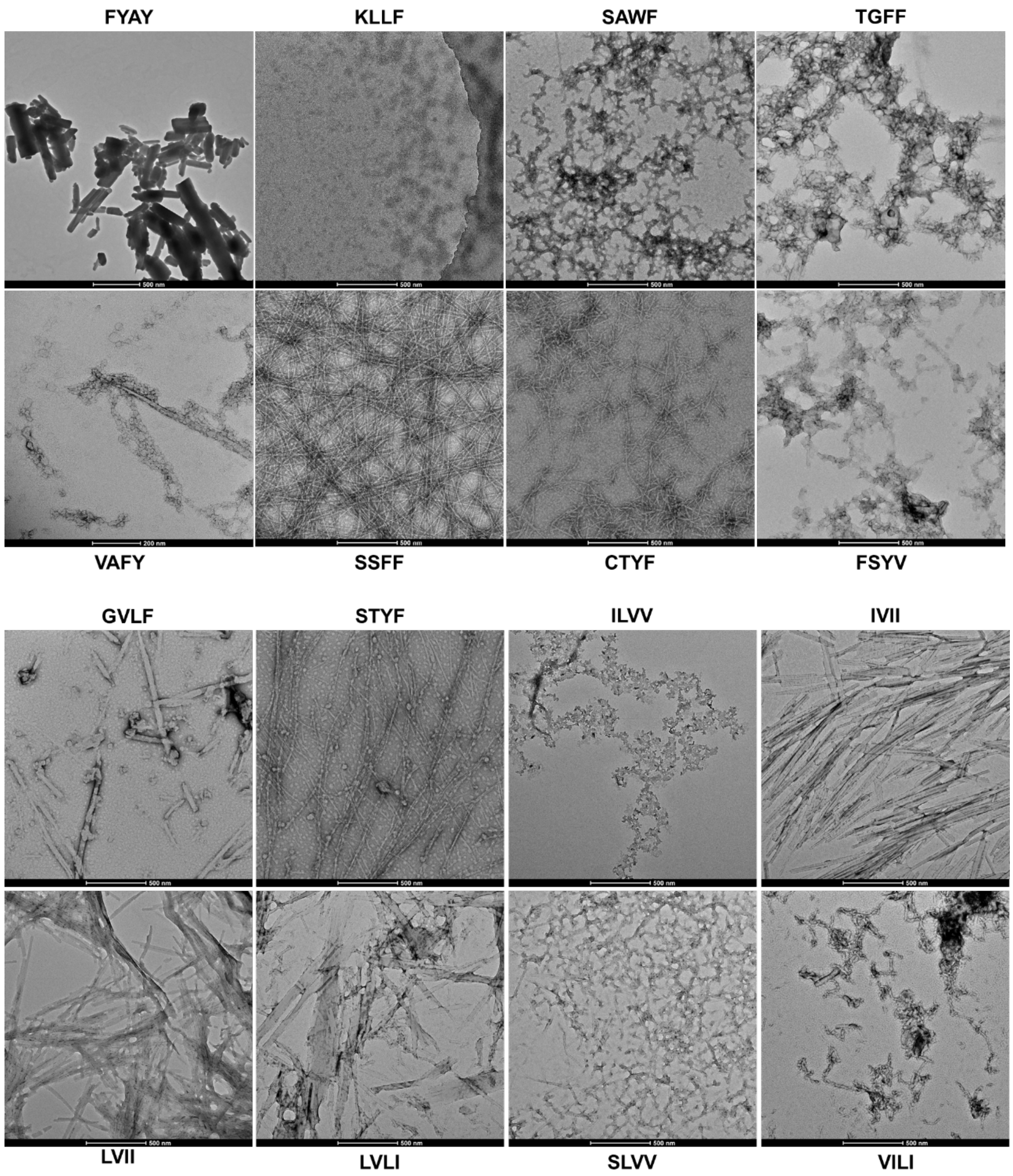
FYAY
KLLF
SAWF
TGFF
VAFY
SSFF
CTYF
FSYV
GVLF
STYF
ILVV
IVII
LVII
LVLI
SLVV
VILI

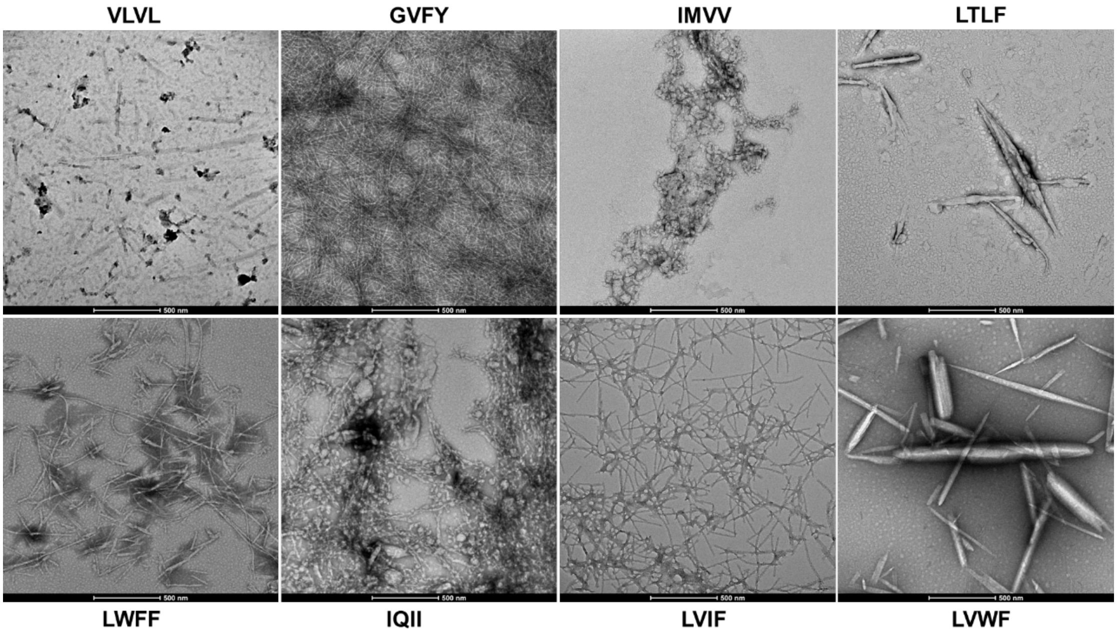
VLVL
GVFY
IMVV
LTLF
LWFF
IQII
LVIF
LVWF

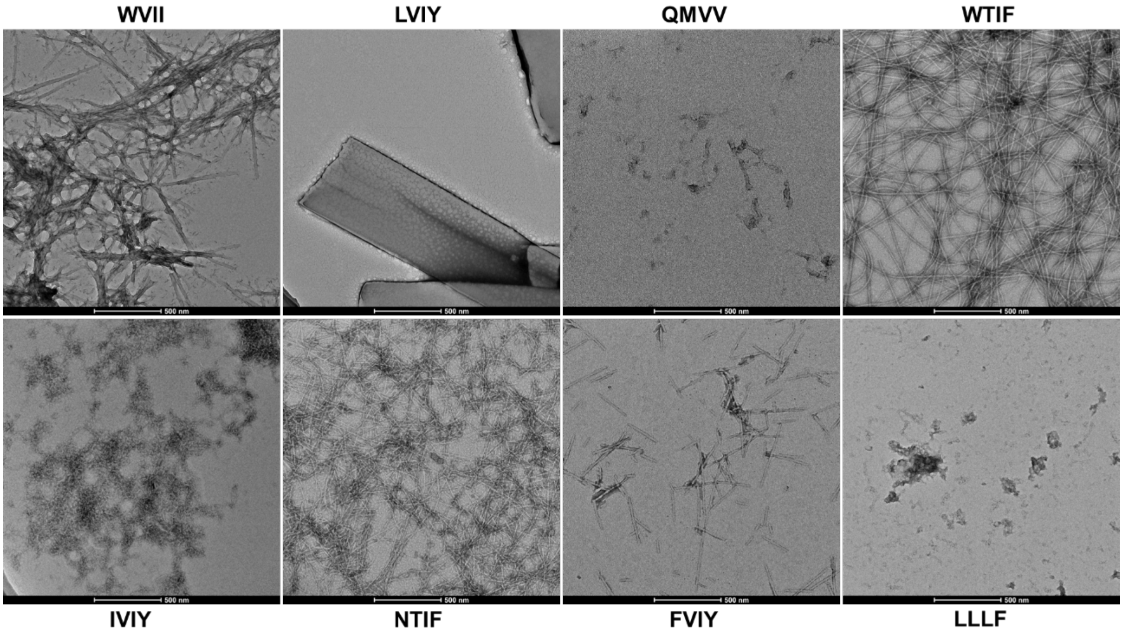
WVII
LVIY
QMVV
WTIF
IVIY
NTIF
FVIY
LLLF

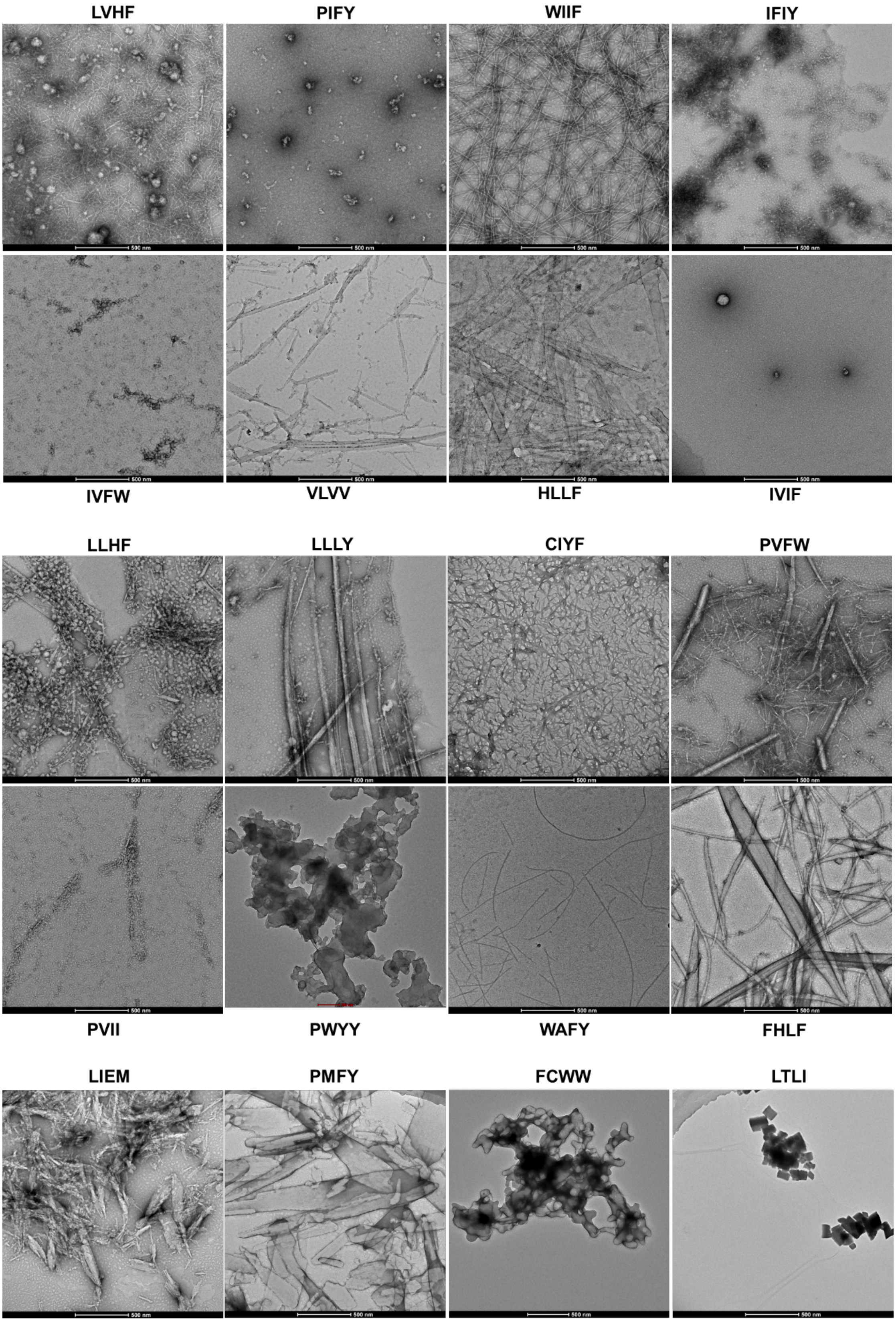
LVHF
PIFY
WIIF
IFIY
IVFW
VLVV
HLLF
IVIF
LLHF
LLLY
CIYF
PVFW
PVII
PWYY
WAFY
FHLF
LIEM
PMFY
FCWW
LTLI

Non-Hydrogels:

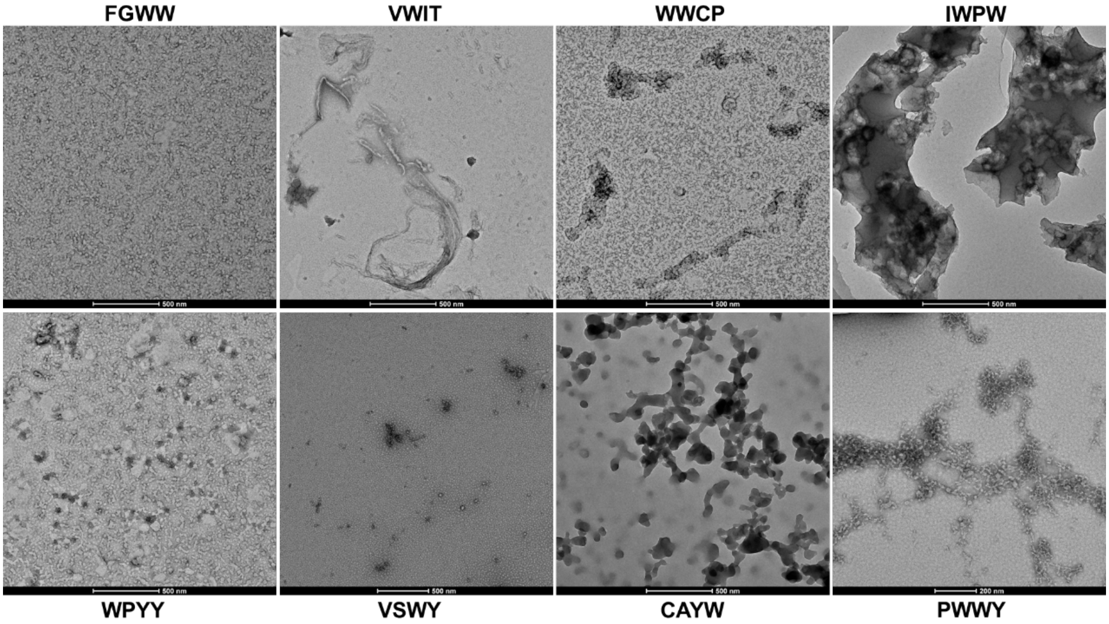

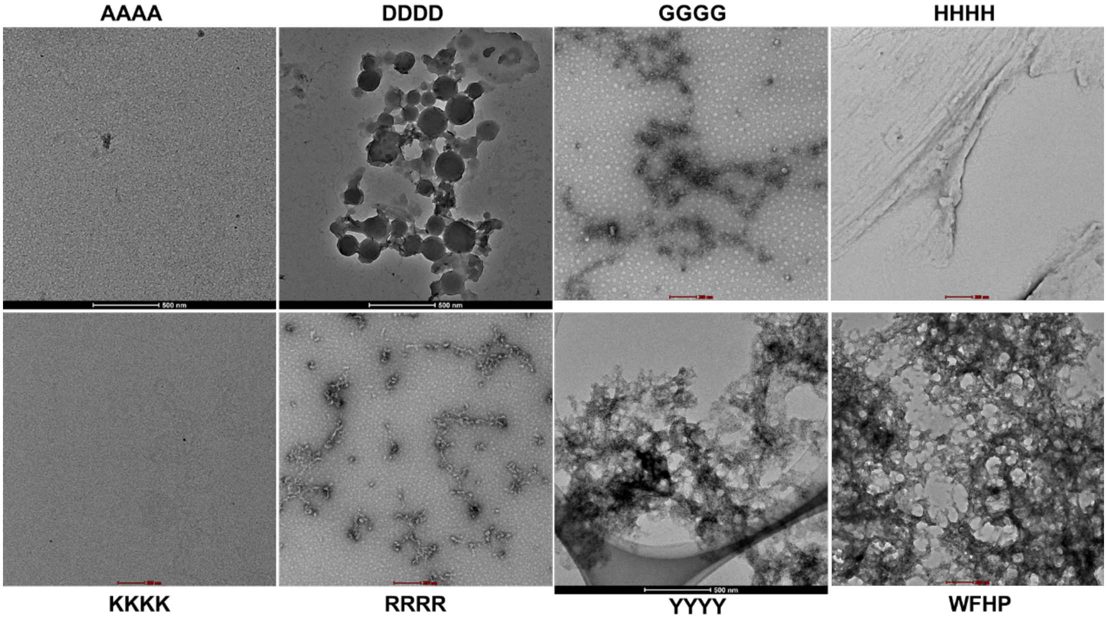

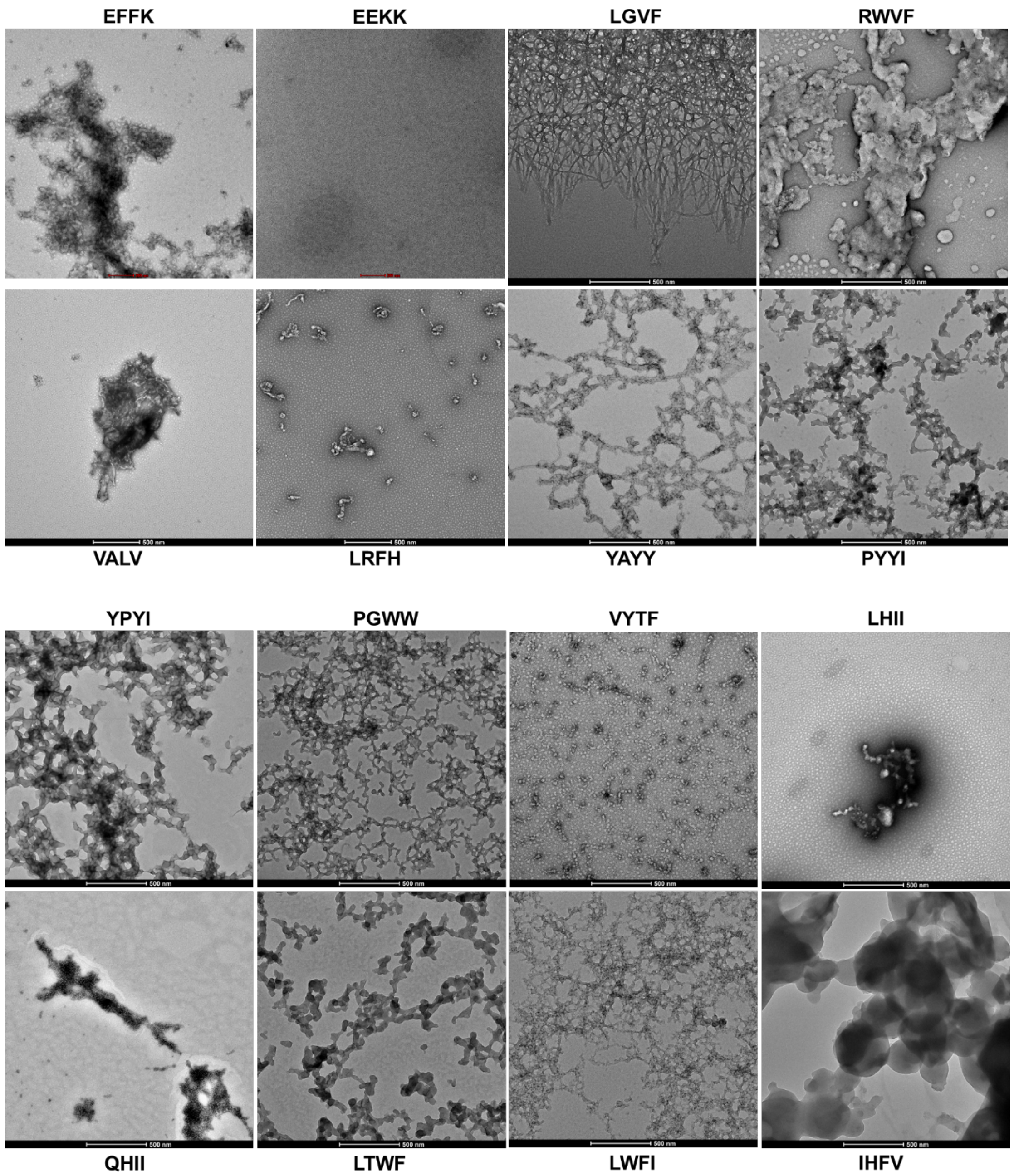
EFFK
EEKK
LGVF
RWVF
500 nm
VALV
LRFH
YAYY
PYYI
YPYI
PGWW
VYTF
LHII
QHII
LTWF
LWFI
IHFV

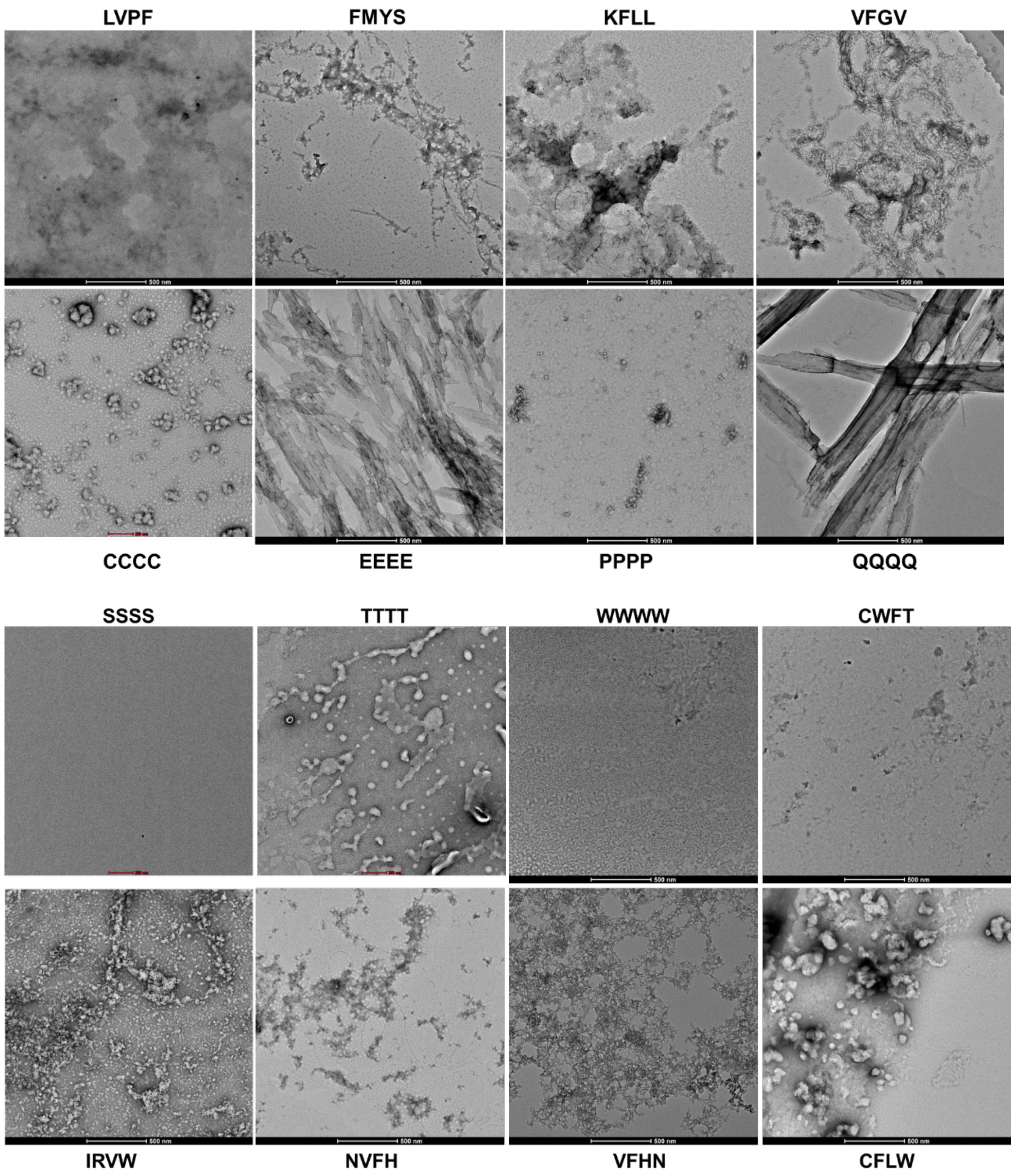
LVPF
FMYS
KFLL
VFGV
CCCC
EEEE
PPPP
QQQQ
SSSS
TTTT
WWWW
CWFT
IRVW
NVFH
VFHN
CFLW

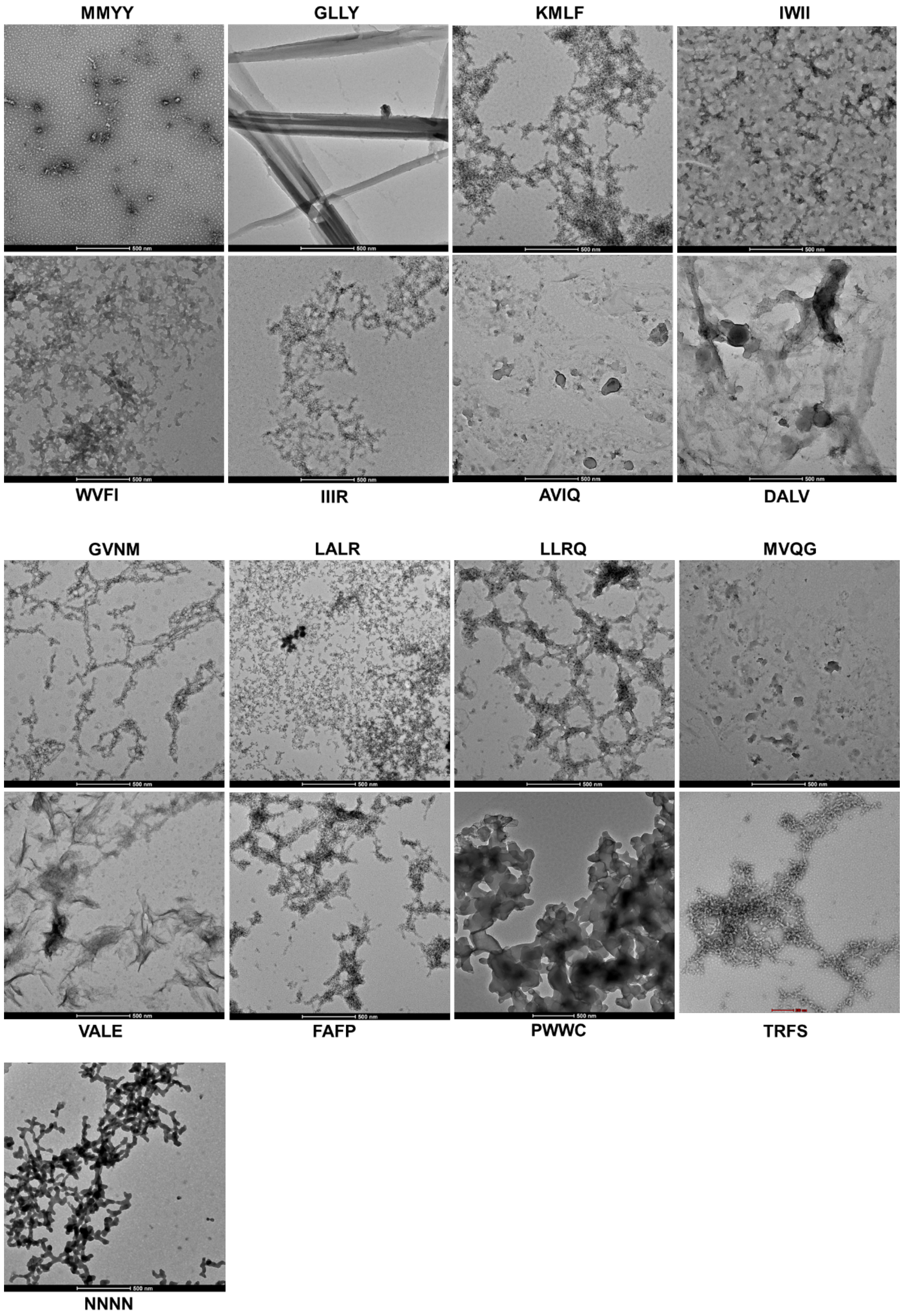
MMYY
GLLY
KMLF
IWII
WVFI
IIIR
AVIQ
DALV
GVNM
LALR
LLRQ
MVQG
VALE
FAFP
PWWC
TRFS
NNNN
